# Supplementary figures and images for: The CLEC3B inhibits cellular proliferation and metastasis of cholangiocarcinoma through Wnt/β-catenin pathway (part 1 of 5)
Source: PeerJ. 2024 Nov 13;12:e18497. doi: 10.7717/peerj.18497 (PMC11568818; doi:10.7717/peerj.18497)

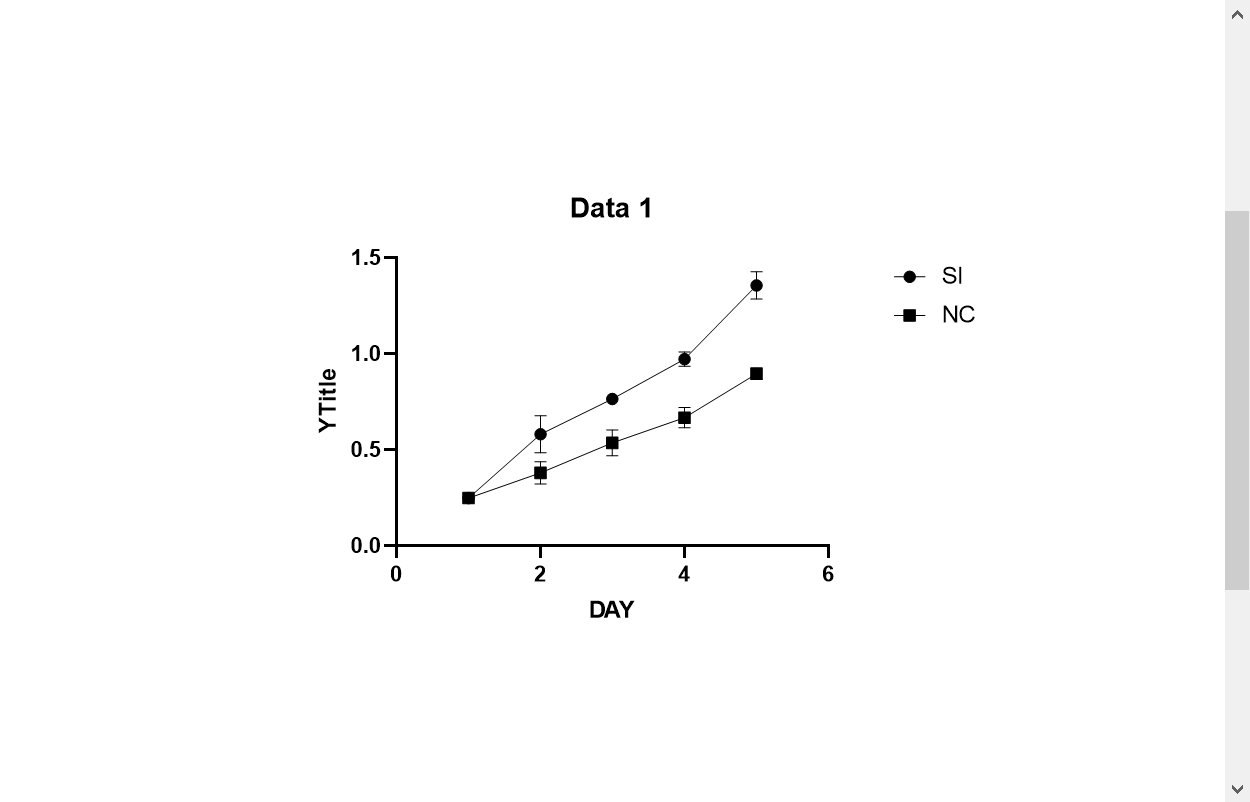

Supplement: Supplemental Information 3 — CCK8 assay of QBC939 cells, NC OE raw data, and original statistical map. [file peerj-12-18497-s003.zip › CCK8 qbc939 clec3b knock down/图.png]

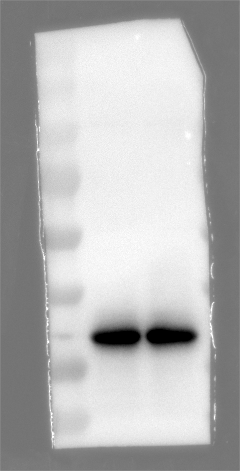

Supplement: Supplemental Information 4 — Western blot original strip, quantitative gray value and statistical map. [file peerj-12-18497-s004.zip › In all Figure , all the original western blot images, original gray value data and statistical graphs were obtained(In addition to overexpression and knock-down validation bands)/HUCCT1/hucct1 over expression clec3b(nc oe) and knock down clec3b (sicon si185)/2023.12.27 hucct clec3b nc oe sicon si/be]

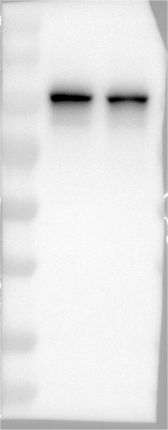

Supplement: Supplemental Information 4 — Western blot original strip, quantitative gray value and statistical map. [file peerj-12-18497-s004.zip › In all Figure , all the original western blot images, original gray value data and statistical graphs were obtained(In addition to overexpression and knock-down validation bands)/HUCCT1/hucct1 over expression clec3b(nc oe) and knock down clec3b (sicon si185)/2023.12.27 hucct clec3b nc oe sicon si/be]

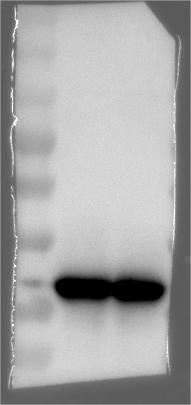

Supplement: Supplemental Information 4 — Western blot original strip, quantitative gray value and statistical map. [file peerj-12-18497-s004.zip › In all Figure , all the original western blot images, original gray value data and statistical graphs were obtained(In addition to overexpression and knock-down validation bands)/HUCCT1/hucct1 over expression clec3b(nc oe) and knock down clec3b (sicon si185)/2023.12.27 hucct clec3b nc oe sicon si/be]

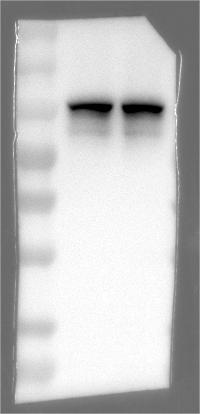

Supplement: Supplemental Information 4 — Western blot original strip, quantitative gray value and statistical map. [file peerj-12-18497-s004.zip › In all Figure , all the original western blot images, original gray value data and statistical graphs were obtained(In addition to overexpression and knock-down validation bands)/HUCCT1/hucct1 over expression clec3b(nc oe) and knock down clec3b (sicon si185)/2023.12.27 hucct clec3b nc oe sicon si/be]

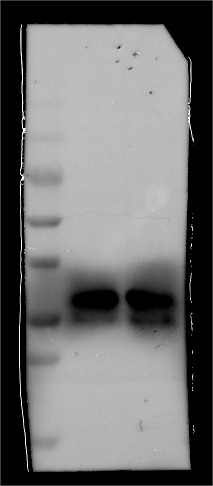

Supplement: Supplemental Information 4 — Western blot original strip, quantitative gray value and statistical map. [file peerj-12-18497-s004.zip › In all Figure , all the original western blot images, original gray value data and statistical graphs were obtained(In addition to overexpression and knock-down validation bands)/HUCCT1/hucct1 over expression clec3b(nc oe) and knock down clec3b (sicon si185)/2023.12.27 hucct clec3b nc oe sicon si/cm]

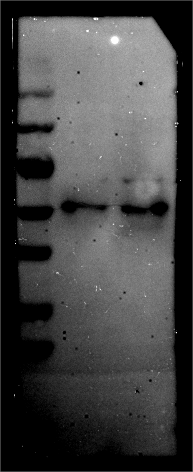

Supplement: Supplemental Information 4 — Western blot original strip, quantitative gray value and statistical map. [file peerj-12-18497-s004.zip › In all Figure , all the original western blot images, original gray value data and statistical graphs were obtained(In addition to overexpression and knock-down validation bands)/HUCCT1/hucct1 over expression clec3b(nc oe) and knock down clec3b (sicon si185)/2023.12.27 hucct clec3b nc oe sicon si/cm]

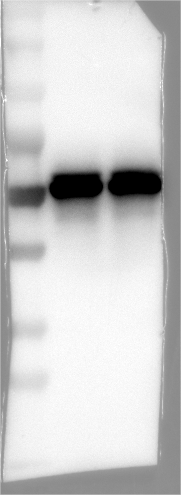

Supplement: Supplemental Information 4 — Western blot original strip, quantitative gray value and statistical map. [file peerj-12-18497-s004.zip › In all Figure , all the original western blot images, original gray value data and statistical graphs were obtained(In addition to overexpression and knock-down validation bands)/HUCCT1/hucct1 over expression clec3b(nc oe) and knock down clec3b (sicon si185)/2023.12.27 hucct clec3b nc oe sicon si/cy]

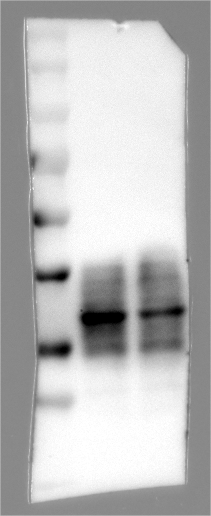

Supplement: Supplemental Information 4 — Western blot original strip, quantitative gray value and statistical map. [file peerj-12-18497-s004.zip › In all Figure , all the original western blot images, original gray value data and statistical graphs were obtained(In addition to overexpression and knock-down validation bands)/HUCCT1/hucct1 over expression clec3b(nc oe) and knock down clec3b (sicon si185)/2023.12.27 hucct clec3b nc oe sicon si/cy]

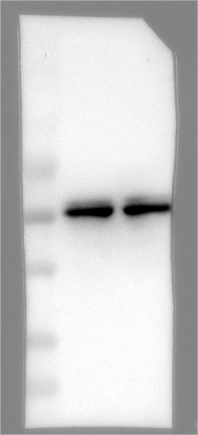

Supplement: Supplemental Information 4 — Western blot original strip, quantitative gray value and statistical map. [file peerj-12-18497-s004.zip › In all Figure , all the original western blot images, original gray value data and statistical graphs were obtained(In addition to overexpression and knock-down validation bands)/HUCCT1/hucct1 over expression clec3b(nc oe) and knock down clec3b (sicon si185)/2023.12.27 hucct clec3b nc oe sicon si/cy]

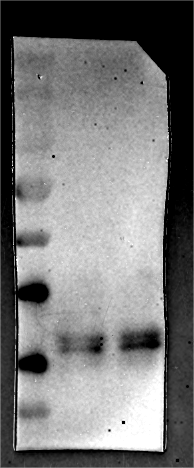

Supplement: Supplemental Information 4 — Western blot original strip, quantitative gray value and statistical map. [file peerj-12-18497-s004.zip › In all Figure , all the original western blot images, original gray value data and statistical graphs were obtained(In addition to overexpression and knock-down validation bands)/HUCCT1/hucct1 over expression clec3b(nc oe) and knock down clec3b (sicon si185)/2023.12.27 hucct clec3b nc oe sicon si/cy]

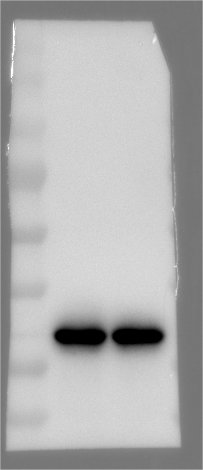

Supplement: Supplemental Information 4 — Western blot original strip, quantitative gray value and statistical map. [file peerj-12-18497-s004.zip › In all Figure , all the original western blot images, original gray value data and statistical graphs were obtained(In addition to overexpression and knock-down validation bands)/HUCCT1/hucct1 over expression clec3b(nc oe) and knock down clec3b (sicon si185)/2023.12.27 hucct clec3b nc oe sicon si/gs]

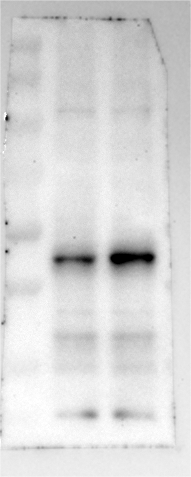

Supplement: Supplemental Information 4 — Western blot original strip, quantitative gray value and statistical map. [file peerj-12-18497-s004.zip › In all Figure , all the original western blot images, original gray value data and statistical graphs were obtained(In addition to overexpression and knock-down validation bands)/HUCCT1/hucct1 over expression clec3b(nc oe) and knock down clec3b (sicon si185)/2023.12.27 hucct clec3b nc oe sicon si/gs]

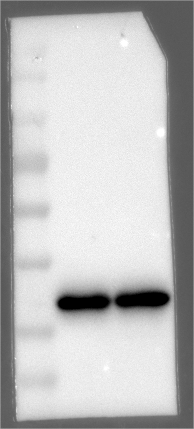

Supplement: Supplemental Information 4 — Western blot original strip, quantitative gray value and statistical map. [file peerj-12-18497-s004.zip › In all Figure , all the original western blot images, original gray value data and statistical graphs were obtained(In addition to overexpression and knock-down validation bands)/HUCCT1/hucct1 over expression clec3b(nc oe) and knock down clec3b (sicon si185)/2024.1.20. HUCCT CLEC3B nc oe sicon si/HU]

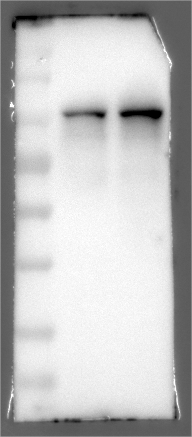

Supplement: Supplemental Information 4 — Western blot original strip, quantitative gray value and statistical map. [file peerj-12-18497-s004.zip › In all Figure , all the original western blot images, original gray value data and statistical graphs were obtained(In addition to overexpression and knock-down validation bands)/HUCCT1/hucct1 over expression clec3b(nc oe) and knock down clec3b (sicon si185)/2024.1.20. HUCCT CLEC3B nc oe sicon si/HU]

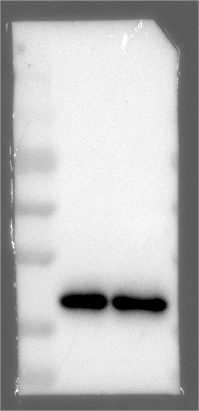

Supplement: Supplemental Information 4 — Western blot original strip, quantitative gray value and statistical map. [file peerj-12-18497-s004.zip › In all Figure , all the original western blot images, original gray value data and statistical graphs were obtained(In addition to overexpression and knock-down validation bands)/HUCCT1/hucct1 over expression clec3b(nc oe) and knock down clec3b (sicon si185)/2024.1.20. HUCCT CLEC3B nc oe sicon si/HU]

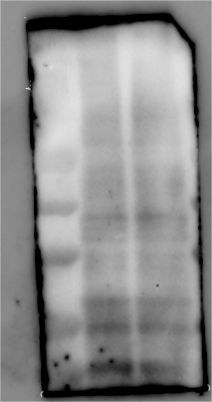

Supplement: Supplemental Information 4 — Western blot original strip, quantitative gray value and statistical map. [file peerj-12-18497-s004.zip › In all Figure , all the original western blot images, original gray value data and statistical graphs were obtained(In addition to overexpression and knock-down validation bands)/HUCCT1/hucct1 over expression clec3b(nc oe) and knock down clec3b (sicon si185)/2024.1.20. HUCCT CLEC3B nc oe sicon si/HU]

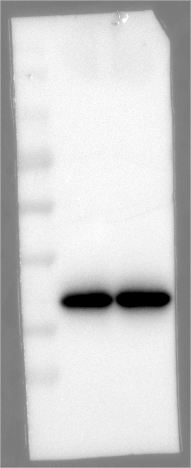

Supplement: Supplemental Information 4 — Western blot original strip, quantitative gray value and statistical map. [file peerj-12-18497-s004.zip › In all Figure , all the original western blot images, original gray value data and statistical graphs were obtained(In addition to overexpression and knock-down validation bands)/HUCCT1/hucct1 over expression clec3b(nc oe) and knock down clec3b (sicon si185)/2024.1.20. HUCCT CLEC3B nc oe sicon si/HU]

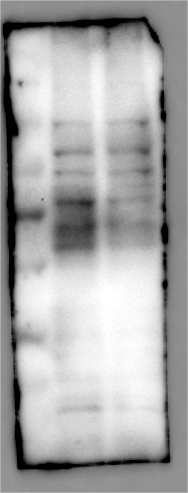

Supplement: Supplemental Information 4 — Western blot original strip, quantitative gray value and statistical map. [file peerj-12-18497-s004.zip › In all Figure , all the original western blot images, original gray value data and statistical graphs were obtained(In addition to overexpression and knock-down validation bands)/HUCCT1/hucct1 over expression clec3b(nc oe) and knock down clec3b (sicon si185)/2024.1.20. HUCCT CLEC3B nc oe sicon si/HU]

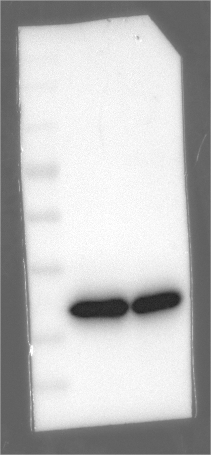

Supplement: Supplemental Information 4 — Western blot original strip, quantitative gray value and statistical map. [file peerj-12-18497-s004.zip › In all Figure , all the original western blot images, original gray value data and statistical graphs were obtained(In addition to overexpression and knock-down validation bands)/HUCCT1/hucct1 over expression clec3b(nc oe) and knock down clec3b (sicon si185)/2024.1.20. HUCCT CLEC3B nc oe sicon si/hu]

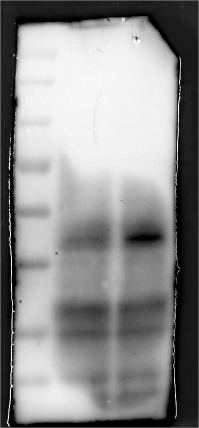

Supplement: Supplemental Information 4 — Western blot original strip, quantitative gray value and statistical map. [file peerj-12-18497-s004.zip › In all Figure , all the original western blot images, original gray value data and statistical graphs were obtained(In addition to overexpression and knock-down validation bands)/HUCCT1/hucct1 over expression clec3b(nc oe) and knock down clec3b (sicon si185)/2024.1.20. HUCCT CLEC3B nc oe sicon si/hu]

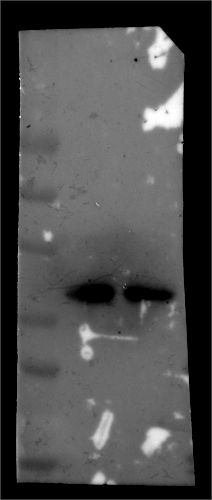

Supplement: Supplemental Information 4 — Western blot original strip, quantitative gray value and statistical map. [file peerj-12-18497-s004.zip › In all Figure , all the original western blot images, original gray value data and statistical graphs were obtained(In addition to overexpression and knock-down validation bands)/HUCCT1/hucct1 over expression clec3b(nc oe) and knock down clec3b (sicon si185)/2024.1.23 HUCCT CLEC3B NC OE SICON SI/cmy]

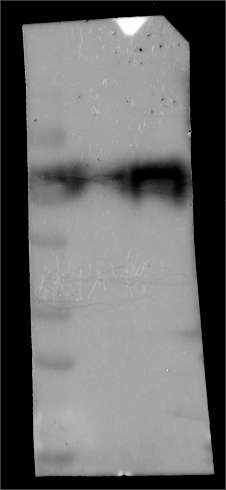

Supplement: Supplemental Information 4 — Western blot original strip, quantitative gray value and statistical map. [file peerj-12-18497-s004.zip › In all Figure , all the original western blot images, original gray value data and statistical graphs were obtained(In addition to overexpression and knock-down validation bands)/HUCCT1/hucct1 over expression clec3b(nc oe) and knock down clec3b (sicon si185)/2024.1.23 HUCCT CLEC3B NC OE SICON SI/cmy]

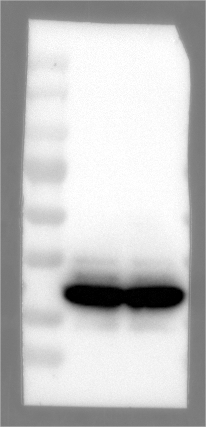

Supplement: Supplemental Information 4 — Western blot original strip, quantitative gray value and statistical map. [file peerj-12-18497-s004.zip › In all Figure , all the original western blot images, original gray value data and statistical graphs were obtained(In addition to overexpression and knock-down validation bands)/HUCCT1/hucct1 over expression clec3b(nc oe) and knock down clec3b (sicon si185)/2024.1.23 HUCCT CLEC3B NC OE SICON SI/huc]

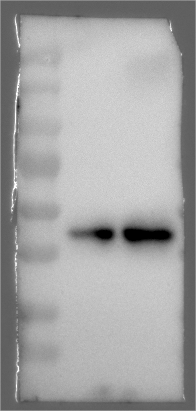

Supplement: Supplemental Information 4 — Western blot original strip, quantitative gray value and statistical map. [file peerj-12-18497-s004.zip › In all Figure , all the original western blot images, original gray value data and statistical graphs were obtained(In addition to overexpression and knock-down validation bands)/HUCCT1/hucct1 over expression clec3b(nc oe) and knock down clec3b (sicon si185)/2024.1.23 HUCCT CLEC3B NC OE SICON SI/huc]

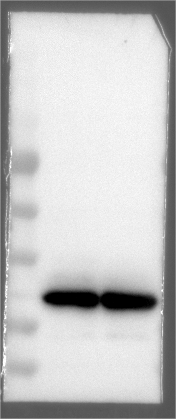

Supplement: Supplemental Information 4 — Western blot original strip, quantitative gray value and statistical map. [file peerj-12-18497-s004.zip › In all Figure , all the original western blot images, original gray value data and statistical graphs were obtained(In addition to overexpression and knock-down validation bands)/HUCCT1/hucct1 over expression clec3b(nc oe) and knock down clec3b (sicon si185)/2024.1.23 HUCCT CLEC3B NC OE SICON SI/huc]

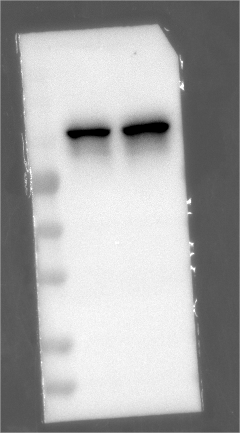

Supplement: Supplemental Information 4 — Western blot original strip, quantitative gray value and statistical map. [file peerj-12-18497-s004.zip › In all Figure , all the original western blot images, original gray value data and statistical graphs were obtained(In addition to overexpression and knock-down validation bands)/HUCCT1/hucct1 over expression clec3b(nc oe) and knock down clec3b (sicon si185)/2024.1.23 HUCCT CLEC3B NC OE SICON SI/huc]

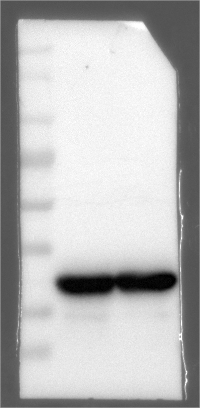

Supplement: Supplemental Information 4 — Western blot original strip, quantitative gray value and statistical map. [file peerj-12-18497-s004.zip › In all Figure , all the original western blot images, original gray value data and statistical graphs were obtained(In addition to overexpression and knock-down validation bands)/HUCCT1/hucct1 over expression clec3b(nc oe) and knock down clec3b (sicon si185)/2024.1.23 HUCCT CLEC3B NC OE SICON SI/HUC]

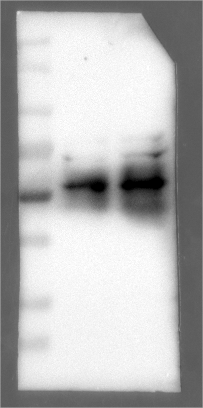

Supplement: Supplemental Information 4 — Western blot original strip, quantitative gray value and statistical map. [file peerj-12-18497-s004.zip › In all Figure , all the original western blot images, original gray value data and statistical graphs were obtained(In addition to overexpression and knock-down validation bands)/HUCCT1/hucct1 over expression clec3b(nc oe) and knock down clec3b (sicon si185)/2024.1.23 HUCCT CLEC3B NC OE SICON SI/HUC]

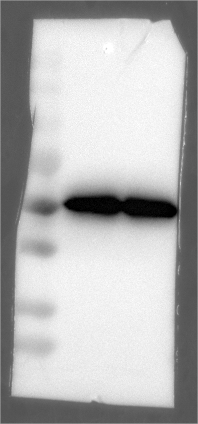

Supplement: Supplemental Information 4 — Western blot original strip, quantitative gray value and statistical map. [file peerj-12-18497-s004.zip › In all Figure , all the original western blot images, original gray value data and statistical graphs were obtained(In addition to overexpression and knock-down validation bands)/HUCCT1/hucct1 over expression clec3b(nc oe) and knock down clec3b (sicon si185)/2024.1.23 HUCCT CLEC3B NC OE SICON SI/HUC]

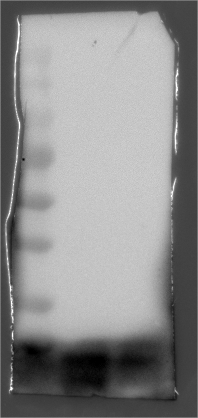

Supplement: Supplemental Information 4 — Western blot original strip, quantitative gray value and statistical map. [file peerj-12-18497-s004.zip › In all Figure , all the original western blot images, original gray value data and statistical graphs were obtained(In addition to overexpression and knock-down validation bands)/HUCCT1/hucct1 over expression clec3b(nc oe) and knock down clec3b (sicon si185)/2024.1.23 HUCCT CLEC3B NC OE SICON SI/HUC]

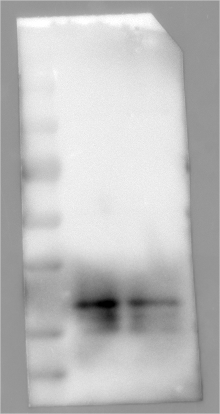

Supplement: Supplemental Information 4 — Western blot original strip, quantitative gray value and statistical map. [file peerj-12-18497-s004.zip › In all Figure , all the original western blot images, original gray value data and statistical graphs were obtained(In addition to overexpression and knock-down validation bands)/HUCCT1/hucct1 over expression clec3b(nc oe) and knock down clec3b (sicon si185)/2024.1.23 HUCCT CLEC3B NC OE SICON SI/HUC]

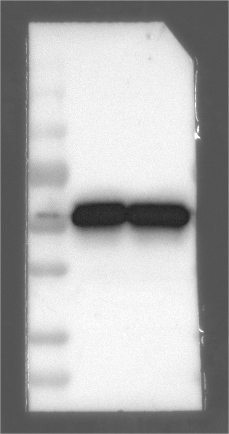

Supplement: Supplemental Information 4 — Western blot original strip, quantitative gray value and statistical map. [file peerj-12-18497-s004.zip › In all Figure , all the original western blot images, original gray value data and statistical graphs were obtained(In addition to overexpression and knock-down validation bands)/HUCCT1/hucct1 over expression clec3b(nc oe) and knock down clec3b (sicon si185)/2024.1.23 HUCCT CLEC3B NC OE SICON SI/HUC]

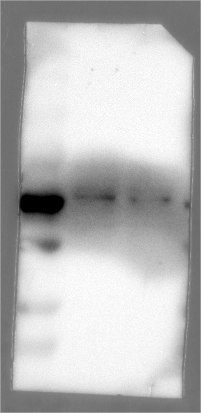

Supplement: Supplemental Information 4 — Western blot original strip, quantitative gray value and statistical map. [file peerj-12-18497-s004.zip › In all Figure , all the original western blot images, original gray value data and statistical graphs were obtained(In addition to overexpression and knock-down validation bands)/HUCCT1/hucct1 over expression clec3b(nc oe) and knock down clec3b (sicon si185)/2024.1.23 HUCCT CLEC3B NC OE SICON SI/HUC]

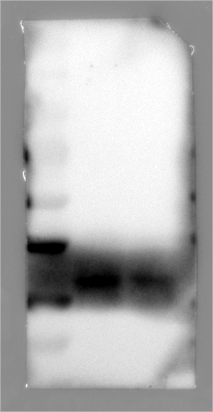

Supplement: Supplemental Information 4 — Western blot original strip, quantitative gray value and statistical map. [file peerj-12-18497-s004.zip › In all Figure , all the original western blot images, original gray value data and statistical graphs were obtained(In addition to overexpression and knock-down validation bands)/HUCCT1/hucct1 over expression clec3b(nc oe) and knock down clec3b (sicon si185)/2024.1.23 HUCCT CLEC3B NC OE SICON SI/HUC]

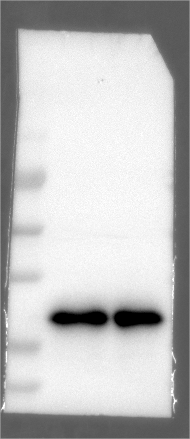

Supplement: Supplemental Information 4 — Western blot original strip, quantitative gray value and statistical map. [file peerj-12-18497-s004.zip › In all Figure , all the original western blot images, original gray value data and statistical graphs were obtained(In addition to overexpression and knock-down validation bands)/HUCCT1/hucct1 over expression clec3b(nc oe) and knock down clec3b (sicon si185)/2024.1.23 HUCCT CLEC3B NC OE SICON SI/HUC]

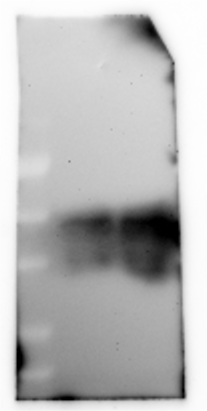

Supplement: Supplemental Information 4 — Western blot original strip, quantitative gray value and statistical map. [file peerj-12-18497-s004.zip › In all Figure , all the original western blot images, original gray value data and statistical graphs were obtained(In addition to overexpression and knock-down validation bands)/HUCCT1/hucct1 over expression clec3b(nc oe) and knock down clec3b (sicon si185)/2024.1.23 HUCCT CLEC3B NC OE SICON SI/HUC]

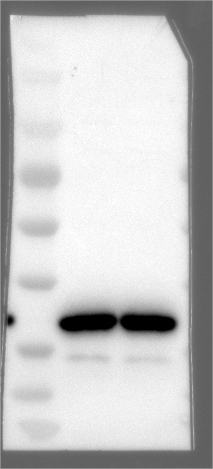

Supplement: Supplemental Information 4 — Western blot original strip, quantitative gray value and statistical map. [file peerj-12-18497-s004.zip › In all Figure , all the original western blot images, original gray value data and statistical graphs were obtained(In addition to overexpression and knock-down validation bands)/HUCCT1/hucct1 over expression clec3b(nc oe) and knock down clec3b (sicon si185)/2024.1.26 hucct clec3b nc oe sicon si/bei]

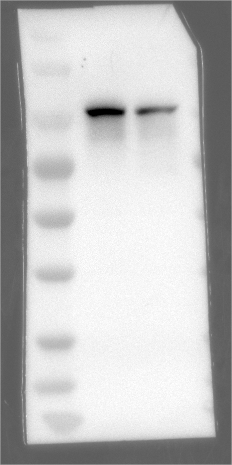

Supplement: Supplemental Information 4 — Western blot original strip, quantitative gray value and statistical map. [file peerj-12-18497-s004.zip › In all Figure , all the original western blot images, original gray value data and statistical graphs were obtained(In addition to overexpression and knock-down validation bands)/HUCCT1/hucct1 over expression clec3b(nc oe) and knock down clec3b (sicon si185)/2024.1.26 hucct clec3b nc oe sicon si/bei]

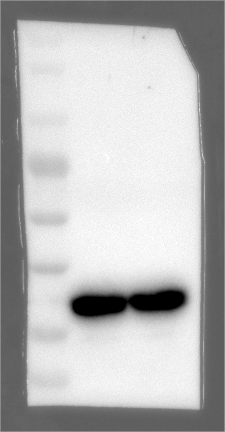

Supplement: Supplemental Information 4 — Western blot original strip, quantitative gray value and statistical map. [file peerj-12-18497-s004.zip › In all Figure , all the original western blot images, original gray value data and statistical graphs were obtained(In addition to overexpression and knock-down validation bands)/HUCCT1/hucct1 over expression clec3b(nc oe) and knock down clec3b (sicon si185)/2024.1.26 hucct clec3b nc oe sicon si/c-m]

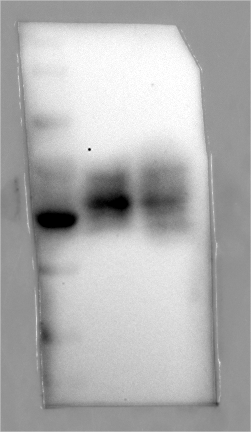

Supplement: Supplemental Information 4 — Western blot original strip, quantitative gray value and statistical map. [file peerj-12-18497-s004.zip › In all Figure , all the original western blot images, original gray value data and statistical graphs were obtained(In addition to overexpression and knock-down validation bands)/HUCCT1/hucct1 over expression clec3b(nc oe) and knock down clec3b (sicon si185)/2024.1.26 hucct clec3b nc oe sicon si/c-m]

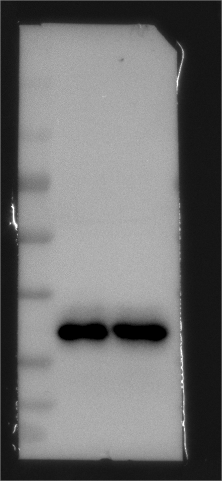

Supplement: Supplemental Information 4 — Western blot original strip, quantitative gray value and statistical map. [file peerj-12-18497-s004.zip › In all Figure , all the original western blot images, original gray value data and statistical graphs were obtained(In addition to overexpression and knock-down validation bands)/HUCCT1/hucct1 over expression clec3b(nc oe) and knock down clec3b (sicon si185)/2024.1.26 hucct clec3b nc oe sicon si/c-m]

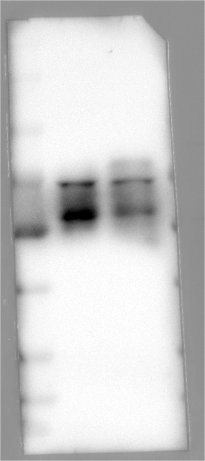

Supplement: Supplemental Information 4 — Western blot original strip, quantitative gray value and statistical map. [file peerj-12-18497-s004.zip › In all Figure , all the original western blot images, original gray value data and statistical graphs were obtained(In addition to overexpression and knock-down validation bands)/HUCCT1/hucct1 over expression clec3b(nc oe) and knock down clec3b (sicon si185)/2024.1.26 hucct clec3b nc oe sicon si/c-m]

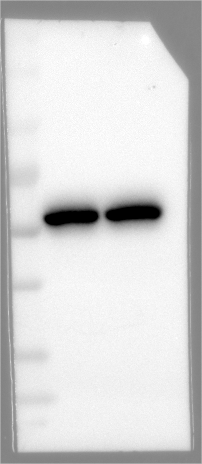

Supplement: Supplemental Information 4 — Western blot original strip, quantitative gray value and statistical map. [file peerj-12-18497-s004.zip › In all Figure , all the original western blot images, original gray value data and statistical graphs were obtained(In addition to overexpression and knock-down validation bands)/HUCCT1/hucct1 over expression clec3b(nc oe) and knock down clec3b (sicon si185)/2024.1.26 hucct clec3b nc oe sicon si/cyc]

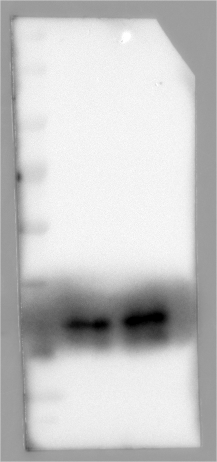

Supplement: Supplemental Information 4 — Western blot original strip, quantitative gray value and statistical map. [file peerj-12-18497-s004.zip › In all Figure , all the original western blot images, original gray value data and statistical graphs were obtained(In addition to overexpression and knock-down validation bands)/HUCCT1/hucct1 over expression clec3b(nc oe) and knock down clec3b (sicon si185)/2024.1.26 hucct clec3b nc oe sicon si/cyc]

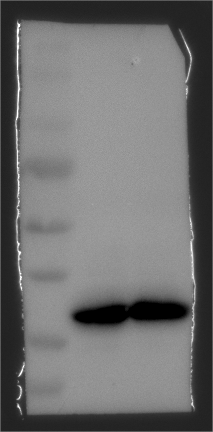

Supplement: Supplemental Information 4 — Western blot original strip, quantitative gray value and statistical map. [file peerj-12-18497-s004.zip › In all Figure , all the original western blot images, original gray value data and statistical graphs were obtained(In addition to overexpression and knock-down validation bands)/HUCCT1/hucct1 over expression clec3b(nc oe) and knock down clec3b (sicon si185)/2024.1.26 hucct clec3b nc oe sicon si/gsk]

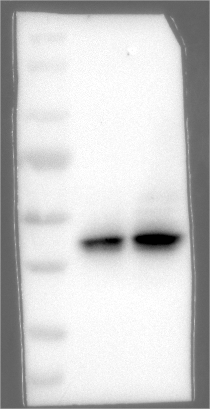

Supplement: Supplemental Information 4 — Western blot original strip, quantitative gray value and statistical map. [file peerj-12-18497-s004.zip › In all Figure , all the original western blot images, original gray value data and statistical graphs were obtained(In addition to overexpression and knock-down validation bands)/HUCCT1/hucct1 over expression clec3b(nc oe) and knock down clec3b (sicon si185)/2024.1.26 hucct clec3b nc oe sicon si/gsk]

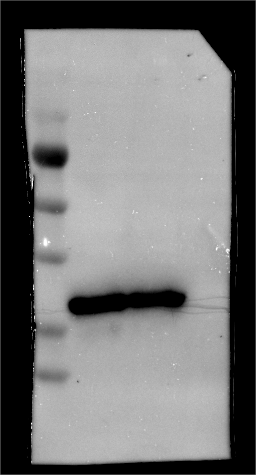

Supplement: Supplemental Information 4 — Western blot original strip, quantitative gray value and statistical map. [file peerj-12-18497-s004.zip › In all Figure , all the original western blot images, original gray value data and statistical graphs were obtained(In addition to overexpression and knock-down validation bands)/HUCCT1/hucct1 over expression clec3b(nc oe) and knock down clec3b (sicon si185)/2024.1.26 hucct clec3b nc oe sicon si/p-g]

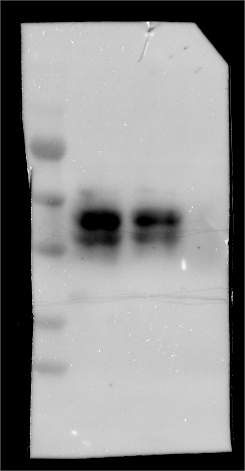

Supplement: Supplemental Information 4 — Western blot original strip, quantitative gray value and statistical map. [file peerj-12-18497-s004.zip › In all Figure , all the original western blot images, original gray value data and statistical graphs were obtained(In addition to overexpression and knock-down validation bands)/HUCCT1/hucct1 over expression clec3b(nc oe) and knock down clec3b (sicon si185)/2024.1.26 hucct clec3b nc oe sicon si/p-g]

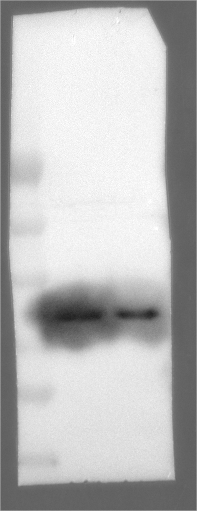

Supplement: Supplemental Information 4 — Western blot original strip, quantitative gray value and statistical map. [file peerj-12-18497-s004.zip › In all Figure , all the original western blot images, original gray value data and statistical graphs were obtained(In addition to overexpression and knock-down validation bands)/HUCCT1/hucct1 over expression clec3b(nc oe) and knock down clec3b (sicon si185)/2024.1.26 hucct clec3b nc oe sicon si/新建文]

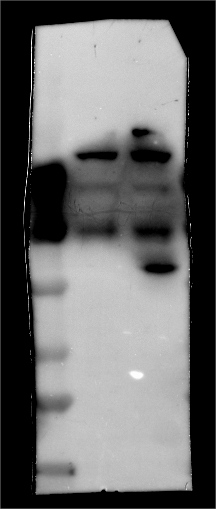

Supplement: Supplemental Information 4 — Western blot original strip, quantitative gray value and statistical map. [file peerj-12-18497-s004.zip › In all Figure , all the original western blot images, original gray value data and statistical graphs were obtained(In addition to overexpression and knock-down validation bands)/HUCCT1/hucct1 over expression clec3b(nc oe) and knock down clec3b (sicon si185)/2024.1.26 hucct clec3b nc oe sicon si/新建文]

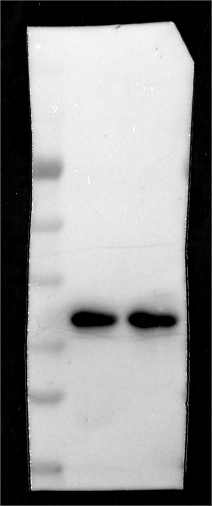

Supplement: Supplemental Information 4 — Western blot original strip, quantitative gray value and statistical map. [file peerj-12-18497-s004.zip › In all Figure , all the original western blot images, original gray value data and statistical graphs were obtained(In addition to overexpression and knock-down validation bands)/HUCCT1/hucct1 over expression clec3b(nc oe) and knock down clec3b (sicon si185)/2024.1.26 hucct clec3b nc oe sicon si/新建文]

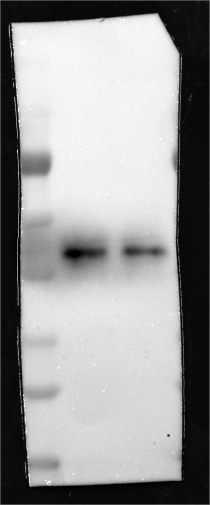

Supplement: Supplemental Information 4 — Western blot original strip, quantitative gray value and statistical map. [file peerj-12-18497-s004.zip › In all Figure , all the original western blot images, original gray value data and statistical graphs were obtained(In addition to overexpression and knock-down validation bands)/HUCCT1/hucct1 over expression clec3b(nc oe) and knock down clec3b (sicon si185)/2024.1.26 hucct clec3b nc oe sicon si/新建文]

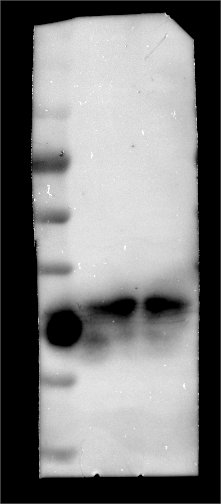

Supplement: Supplemental Information 4 — Western blot original strip, quantitative gray value and statistical map. [file peerj-12-18497-s004.zip › In all Figure , all the original western blot images, original gray value data and statistical graphs were obtained(In addition to overexpression and knock-down validation bands)/HUCCT1/hucct1 over expression clec3b(nc oe) and knock down clec3b (sicon si185)/2024.1.26 hucct clec3b nc oe sicon si/新建文]

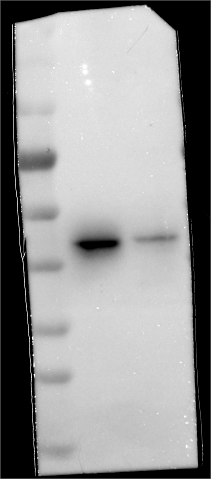

Supplement: Supplemental Information 4 — Western blot original strip, quantitative gray value and statistical map. [file peerj-12-18497-s004.zip › In all Figure , all the original western blot images, original gray value data and statistical graphs were obtained(In addition to overexpression and knock-down validation bands)/HUCCT1/hucct1 over expression clec3b(nc oe) and knock down clec3b (sicon si185)/2024.1.26 hucct clec3b nc oe sicon si/新建文]

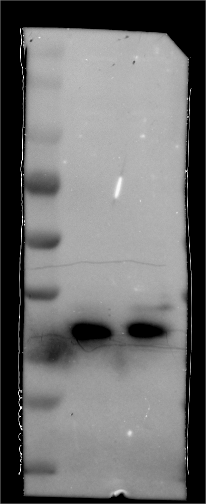

Supplement: Supplemental Information 4 — Western blot original strip, quantitative gray value and statistical map. [file peerj-12-18497-s004.zip › In all Figure , all the original western blot images, original gray value data and statistical graphs were obtained(In addition to overexpression and knock-down validation bands)/HUCCT1/hucct1 over expression clec3b(nc oe) and knock down clec3b (sicon si185)/2024.1.26 hucct clec3b nc oe sicon si/新建文]

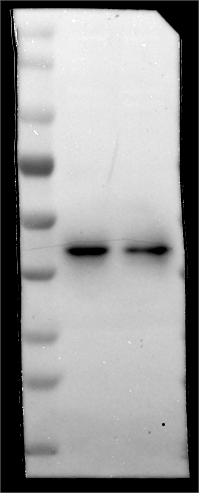

Supplement: Supplemental Information 4 — Western blot original strip, quantitative gray value and statistical map. [file peerj-12-18497-s004.zip › In all Figure , all the original western blot images, original gray value data and statistical graphs were obtained(In addition to overexpression and knock-down validation bands)/HUCCT1/hucct1 over expression clec3b(nc oe) and knock down clec3b (sicon si185)/2024.1.26 hucct clec3b nc oe sicon si/新建文]

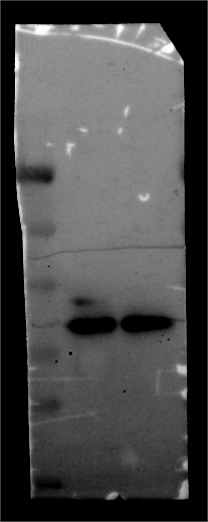

Supplement: Supplemental Information 4 — Western blot original strip, quantitative gray value and statistical map. [file peerj-12-18497-s004.zip › In all Figure , all the original western blot images, original gray value data and statistical graphs were obtained(In addition to overexpression and knock-down validation bands)/HUCCT1/hucct1 over expression clec3b(nc oe) and knock down clec3b (sicon si185)/2024.1.26 hucct clec3b nc oe sicon si/新建文]

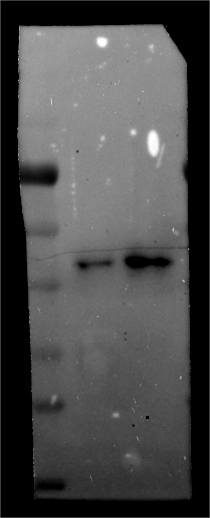

Supplement: Supplemental Information 4 — Western blot original strip, quantitative gray value and statistical map. [file peerj-12-18497-s004.zip › In all Figure , all the original western blot images, original gray value data and statistical graphs were obtained(In addition to overexpression and knock-down validation bands)/HUCCT1/hucct1 over expression clec3b(nc oe) and knock down clec3b (sicon si185)/2024.1.26 hucct clec3b nc oe sicon si/新建文]

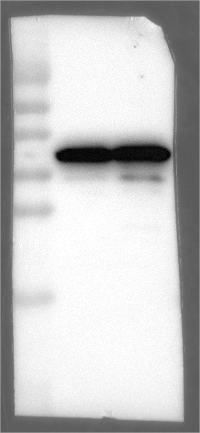

Supplement: Supplemental Information 4 — Western blot original strip, quantitative gray value and statistical map. [file peerj-12-18497-s004.zip › In all Figure , all the original western blot images, original gray value data and statistical graphs were obtained(In addition to overexpression and knock-down validation bands)/HUCCT1/hucct1 over expression clec3b(nc oe) and knock down clec3b (sicon si185)/2024.1.30 hucct clec3b/clec3b nc oe 第1张 G]

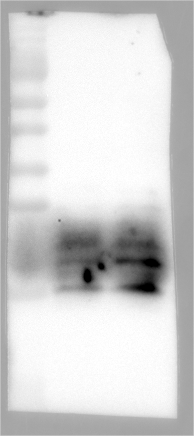

Supplement: Supplemental Information 4 — Western blot original strip, quantitative gray value and statistical map. [file peerj-12-18497-s004.zip › In all Figure , all the original western blot images, original gray value data and statistical graphs were obtained(In addition to overexpression and knock-down validation bands)/HUCCT1/hucct1 over expression clec3b(nc oe) and knock down clec3b (sicon si185)/2024.1.30 hucct clec3b/clec3b nc oe 第1张.p]

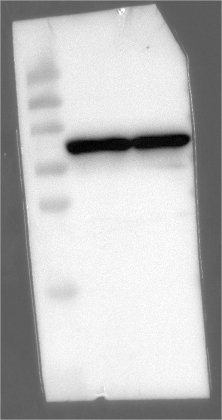

Supplement: Supplemental Information 4 — Western blot original strip, quantitative gray value and statistical map. [file peerj-12-18497-s004.zip › In all Figure , all the original western blot images, original gray value data and statistical graphs were obtained(In addition to overexpression and knock-down validation bands)/HUCCT1/hucct1 over expression clec3b(nc oe) and knock down clec3b (sicon si185)/2024.1.30 hucct clec3b/clec3b nc oe 第2张 G]

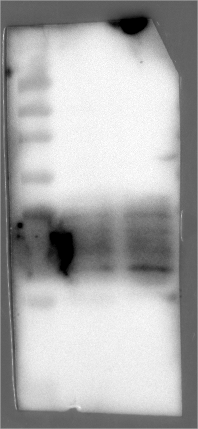

Supplement: Supplemental Information 4 — Western blot original strip, quantitative gray value and statistical map. [file peerj-12-18497-s004.zip › In all Figure , all the original western blot images, original gray value data and statistical graphs were obtained(In addition to overexpression and knock-down validation bands)/HUCCT1/hucct1 over expression clec3b(nc oe) and knock down clec3b (sicon si185)/2024.1.30 hucct clec3b/clec3b nc oe 第2张.p]

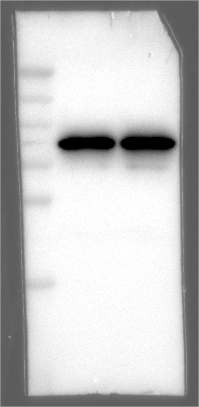

Supplement: Supplemental Information 4 — Western blot original strip, quantitative gray value and statistical map. [file peerj-12-18497-s004.zip › In all Figure , all the original western blot images, original gray value data and statistical graphs were obtained(In addition to overexpression and knock-down validation bands)/HUCCT1/hucct1 over expression clec3b(nc oe) and knock down clec3b (sicon si185)/2024.1.30 hucct clec3b/clec3b nc oe 第3张 G]

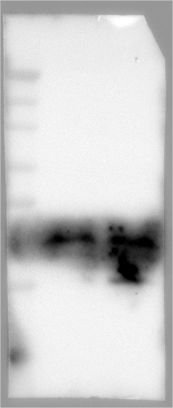

Supplement: Supplemental Information 4 — Western blot original strip, quantitative gray value and statistical map. [file peerj-12-18497-s004.zip › In all Figure , all the original western blot images, original gray value data and statistical graphs were obtained(In addition to overexpression and knock-down validation bands)/HUCCT1/hucct1 over expression clec3b(nc oe) and knock down clec3b (sicon si185)/2024.1.30 hucct clec3b/clec3b nc oe 第3张.p]

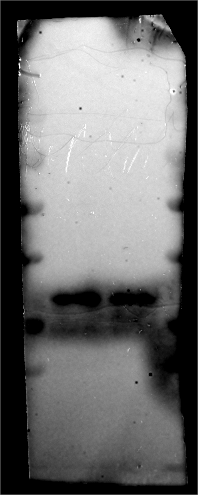

Supplement: Supplemental Information 4 — Western blot original strip, quantitative gray value and statistical map. [file peerj-12-18497-s004.zip › In all Figure , all the original western blot images, original gray value data and statistical graphs were obtained(In addition to overexpression and knock-down validation bands)/HUCCT1/hucct1 over expression clec3b(nc oe) and knock down clec3b (sicon si185)/2024.2.29 HUCCT clec3b nc oe sicon si185/]

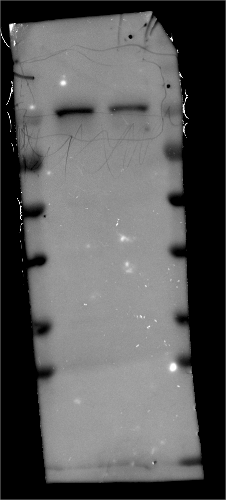

Supplement: Supplemental Information 4 — Western blot original strip, quantitative gray value and statistical map. [file peerj-12-18497-s004.zip › In all Figure , all the original western blot images, original gray value data and statistical graphs were obtained(In addition to overexpression and knock-down validation bands)/HUCCT1/hucct1 over expression clec3b(nc oe) and knock down clec3b (sicon si185)/2024.2.29 HUCCT clec3b nc oe sicon si185/]

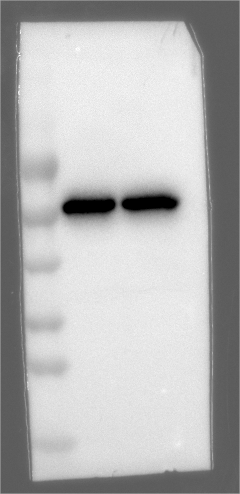

Supplement: Supplemental Information 4 — Western blot original strip, quantitative gray value and statistical map. [file peerj-12-18497-s004.zip › In all Figure , all the original western blot images, original gray value data and statistical graphs were obtained(In addition to overexpression and knock-down validation bands)/HUCCT1/hucct1 over expression clec3b(nc oe) and knock down clec3b (sicon si185)/2024.2.29 HUCCT clec3b nc oe sicon si185/]

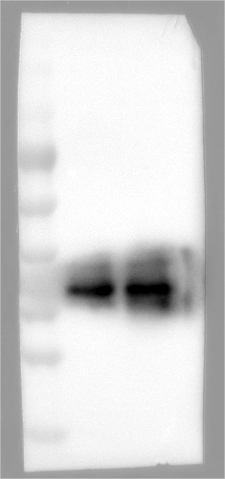

Supplement: Supplemental Information 4 — Western blot original strip, quantitative gray value and statistical map. [file peerj-12-18497-s004.zip › In all Figure , all the original western blot images, original gray value data and statistical graphs were obtained(In addition to overexpression and knock-down validation bands)/HUCCT1/hucct1 over expression clec3b(nc oe) and knock down clec3b (sicon si185)/2024.2.29 HUCCT clec3b nc oe sicon si185/]

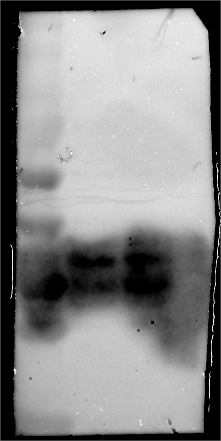

Supplement: Supplemental Information 4 — Western blot original strip, quantitative gray value and statistical map. [file peerj-12-18497-s004.zip › In all Figure , all the original western blot images, original gray value data and statistical graphs were obtained(In addition to overexpression and knock-down validation bands)/HUCCT1/hucct1 over expression clec3b(nc oe) and knock down clec3b (sicon si185)/2024.2.29 HUCCT clec3b nc oe sicon si185/]

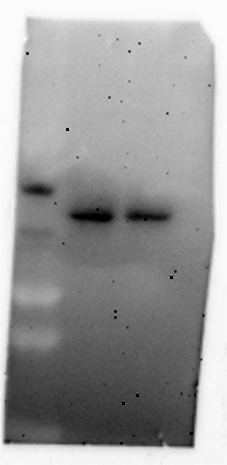

Supplement: Supplemental Information 4 — Western blot original strip, quantitative gray value and statistical map. [file peerj-12-18497-s004.zip › In all Figure , all the original western blot images, original gray value data and statistical graphs were obtained(In addition to overexpression and knock-down validation bands)/HUCCT1/hucct1 over expression clec3b(nc oe) and knock down clec3b (sicon si185)/2024.2.29 HUCCT clec3b nc oe sicon si185/]

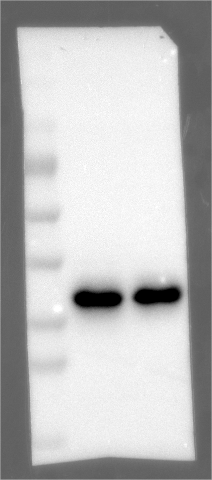

Supplement: Supplemental Information 4 — Western blot original strip, quantitative gray value and statistical map. [file peerj-12-18497-s004.zip › In all Figure , all the original western blot images, original gray value data and statistical graphs were obtained(In addition to overexpression and knock-down validation bands)/HUCCT1/hucct1 over expression clec3b(nc oe) and knock down clec3b (sicon si185)/2024.2.29 HUCCT clec3b nc oe sicon si185/]

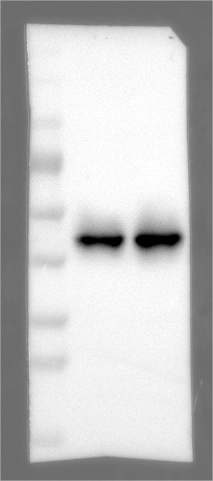

Supplement: Supplemental Information 4 — Western blot original strip, quantitative gray value and statistical map. [file peerj-12-18497-s004.zip › In all Figure , all the original western blot images, original gray value data and statistical graphs were obtained(In addition to overexpression and knock-down validation bands)/HUCCT1/hucct1 over expression clec3b(nc oe) and knock down clec3b (sicon si185)/2024.2.29 HUCCT clec3b nc oe sicon si185/]

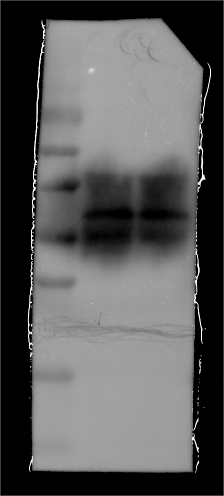

Supplement: Supplemental Information 4 — Western blot original strip, quantitative gray value and statistical map. [file peerj-12-18497-s004.zip › In all Figure , all the original western blot images, original gray value data and statistical graphs were obtained(In addition to overexpression and knock-down validation bands)/HUCCT1/hucct1 western blot bax bcl-2 n-cadherin e-cadherin/2024.4.12 hucct emt bax bcl-2/bax sicon si185 第2张 gapdh.png]

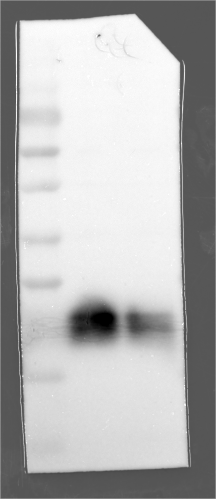

Supplement: Supplemental Information 4 — Western blot original strip, quantitative gray value and statistical map. [file peerj-12-18497-s004.zip › In all Figure , all the original western blot images, original gray value data and statistical graphs were obtained(In addition to overexpression and knock-down validation bands)/HUCCT1/hucct1 western blot bax bcl-2 n-cadherin e-cadherin/2024.4.12 hucct emt bax bcl-2/bax sicon si185 第2张.png]

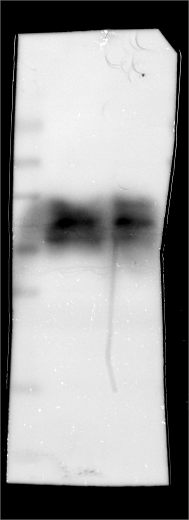

Supplement: Supplemental Information 4 — Western blot original strip, quantitative gray value and statistical map. [file peerj-12-18497-s004.zip › In all Figure , all the original western blot images, original gray value data and statistical graphs were obtained(In addition to overexpression and knock-down validation bands)/HUCCT1/hucct1 western blot bax bcl-2 n-cadherin e-cadherin/2024.4.12 hucct emt bax bcl-2/bcl-2 sicon si185 第1张 gapdh.png]

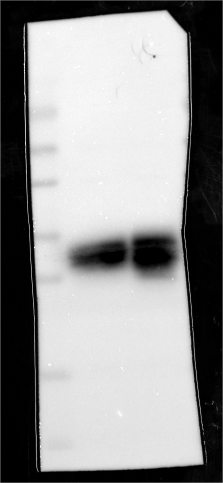

Supplement: Supplemental Information 4 — Western blot original strip, quantitative gray value and statistical map. [file peerj-12-18497-s004.zip › In all Figure , all the original western blot images, original gray value data and statistical graphs were obtained(In addition to overexpression and knock-down validation bands)/HUCCT1/hucct1 western blot bax bcl-2 n-cadherin e-cadherin/2024.4.12 hucct emt bax bcl-2/bcl-2 sicon si185 第1张.png]

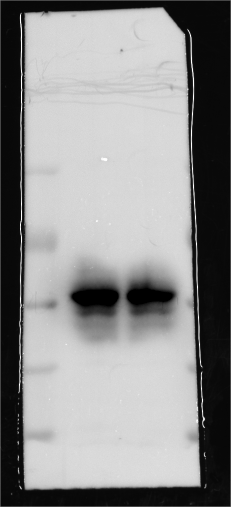

Supplement: Supplemental Information 4 — Western blot original strip, quantitative gray value and statistical map. [file peerj-12-18497-s004.zip › In all Figure , all the original western blot images, original gray value data and statistical graphs were obtained(In addition to overexpression and knock-down validation bands)/HUCCT1/hucct1 western blot bax bcl-2 n-cadherin e-cadherin/2024.4.12 hucct emt bax bcl-2/E-cad nc oe 第1张 tubulin.png]

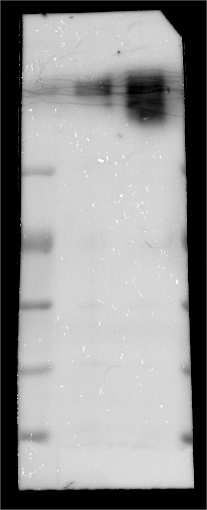

Supplement: Supplemental Information 4 — Western blot original strip, quantitative gray value and statistical map. [file peerj-12-18497-s004.zip › In all Figure , all the original western blot images, original gray value data and statistical graphs were obtained(In addition to overexpression and knock-down validation bands)/HUCCT1/hucct1 western blot bax bcl-2 n-cadherin e-cadherin/2024.4.12 hucct emt bax bcl-2/E-cad nc oe 第1张.png]

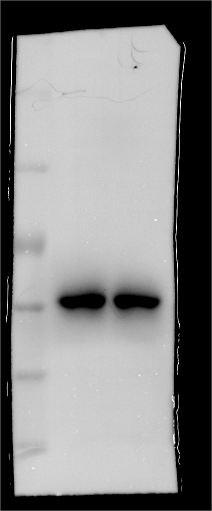

Supplement: Supplemental Information 4 — Western blot original strip, quantitative gray value and statistical map. [file peerj-12-18497-s004.zip › In all Figure , all the original western blot images, original gray value data and statistical graphs were obtained(In addition to overexpression and knock-down validation bands)/HUCCT1/hucct1 western blot bax bcl-2 n-cadherin e-cadherin/2024.4.12 hucct emt bax bcl-2/N-cad nc oe 第2张 tubulin.png]

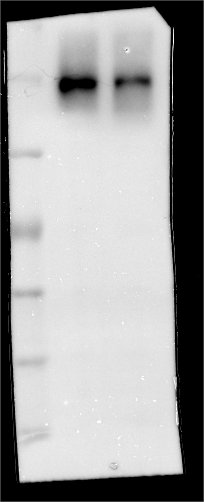

Supplement: Supplemental Information 4 — Western blot original strip, quantitative gray value and statistical map. [file peerj-12-18497-s004.zip › In all Figure , all the original western blot images, original gray value data and statistical graphs were obtained(In addition to overexpression and knock-down validation bands)/HUCCT1/hucct1 western blot bax bcl-2 n-cadherin e-cadherin/2024.4.12 hucct emt bax bcl-2/N-cad nc oe 第2张.png]

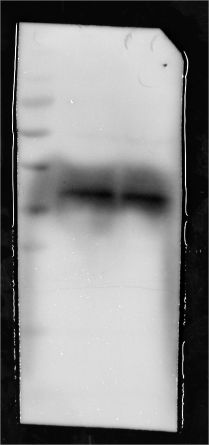

Supplement: Supplemental Information 4 — Western blot original strip, quantitative gray value and statistical map. [file peerj-12-18497-s004.zip › In all Figure , all the original western blot images, original gray value data and statistical graphs were obtained(In addition to overexpression and knock-down validation bands)/HUCCT1/hucct1 western blot bax bcl-2 n-cadherin e-cadherin/2024.4.13 hucct emt bax bcl-2/bax sicon si185 gapdh.png]

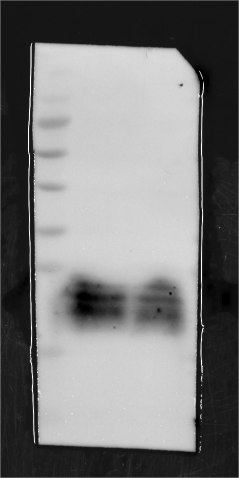

Supplement: Supplemental Information 4 — Western blot original strip, quantitative gray value and statistical map. [file peerj-12-18497-s004.zip › In all Figure , all the original western blot images, original gray value data and statistical graphs were obtained(In addition to overexpression and knock-down validation bands)/HUCCT1/hucct1 western blot bax bcl-2 n-cadherin e-cadherin/2024.4.13 hucct emt bax bcl-2/bax sicon si185.png]

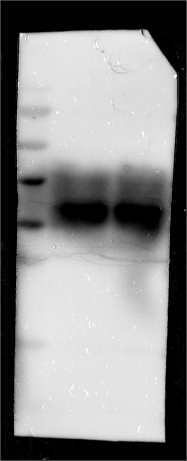

Supplement: Supplemental Information 4 — Western blot original strip, quantitative gray value and statistical map. [file peerj-12-18497-s004.zip › In all Figure , all the original western blot images, original gray value data and statistical graphs were obtained(In addition to overexpression and knock-down validation bands)/HUCCT1/hucct1 western blot bax bcl-2 n-cadherin e-cadherin/2024.4.13 hucct emt bax bcl-2/bcl-2 nc oe gapdh.png]

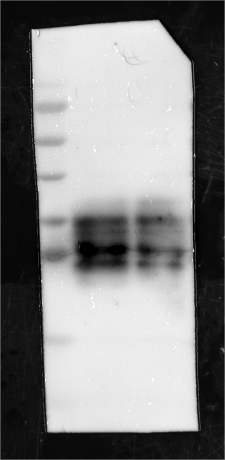

Supplement: Supplemental Information 4 — Western blot original strip, quantitative gray value and statistical map. [file peerj-12-18497-s004.zip › In all Figure , all the original western blot images, original gray value data and statistical graphs were obtained(In addition to overexpression and knock-down validation bands)/HUCCT1/hucct1 western blot bax bcl-2 n-cadherin e-cadherin/2024.4.13 hucct emt bax bcl-2/bcl-2 nc oe.png]

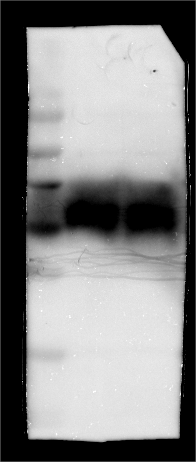

Supplement: Supplemental Information 4 — Western blot original strip, quantitative gray value and statistical map. [file peerj-12-18497-s004.zip › In all Figure , all the original western blot images, original gray value data and statistical graphs were obtained(In addition to overexpression and knock-down validation bands)/HUCCT1/hucct1 western blot bax bcl-2 n-cadherin e-cadherin/2024.4.13 hucct emt bax bcl-2/bcl-2 sicon si185 gapdh.png]

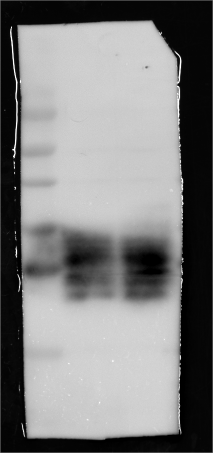

Supplement: Supplemental Information 4 — Western blot original strip, quantitative gray value and statistical map. [file peerj-12-18497-s004.zip › In all Figure , all the original western blot images, original gray value data and statistical graphs were obtained(In addition to overexpression and knock-down validation bands)/HUCCT1/hucct1 western blot bax bcl-2 n-cadherin e-cadherin/2024.4.13 hucct emt bax bcl-2/bcl-2 sicon si185.png]

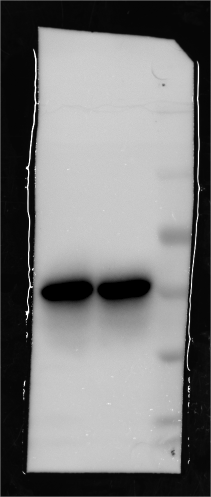

Supplement: Supplemental Information 4 — Western blot original strip, quantitative gray value and statistical map. [file peerj-12-18497-s004.zip › In all Figure , all the original western blot images, original gray value data and statistical graphs were obtained(In addition to overexpression and knock-down validation bands)/HUCCT1/hucct1 western blot bax bcl-2 n-cadherin e-cadherin/2024.4.13 hucct emt bax bcl-2/e-cad nc oe tubulin.png]

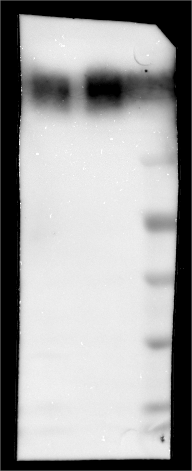

Supplement: Supplemental Information 4 — Western blot original strip, quantitative gray value and statistical map. [file peerj-12-18497-s004.zip › In all Figure , all the original western blot images, original gray value data and statistical graphs were obtained(In addition to overexpression and knock-down validation bands)/HUCCT1/hucct1 western blot bax bcl-2 n-cadherin e-cadherin/2024.4.13 hucct emt bax bcl-2/e-cad nc oe.png]

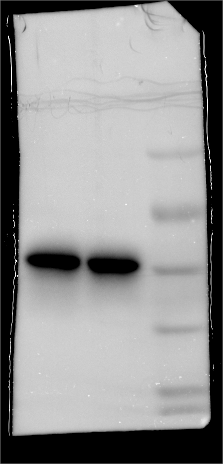

Supplement: Supplemental Information 4 — Western blot original strip, quantitative gray value and statistical map. [file peerj-12-18497-s004.zip › In all Figure , all the original western blot images, original gray value data and statistical graphs were obtained(In addition to overexpression and knock-down validation bands)/HUCCT1/hucct1 western blot bax bcl-2 n-cadherin e-cadherin/2024.4.13 hucct emt bax bcl-2/e-cad sicon si185 tubulin.png]

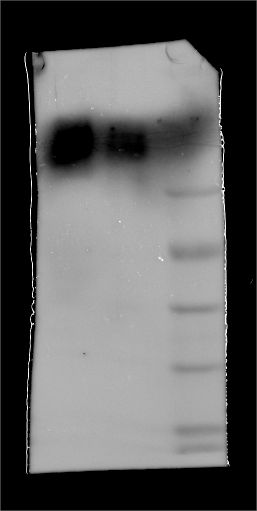

Supplement: Supplemental Information 4 — Western blot original strip, quantitative gray value and statistical map. [file peerj-12-18497-s004.zip › In all Figure , all the original western blot images, original gray value data and statistical graphs were obtained(In addition to overexpression and knock-down validation bands)/HUCCT1/hucct1 western blot bax bcl-2 n-cadherin e-cadherin/2024.4.13 hucct emt bax bcl-2/e-cad sicon si185.png]

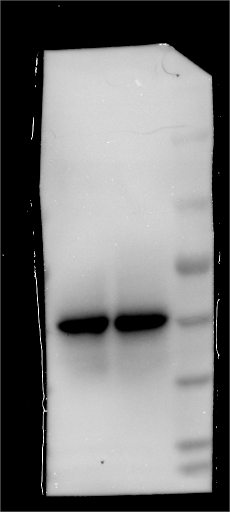

Supplement: Supplemental Information 4 — Western blot original strip, quantitative gray value and statistical map. [file peerj-12-18497-s004.zip › In all Figure , all the original western blot images, original gray value data and statistical graphs were obtained(In addition to overexpression and knock-down validation bands)/HUCCT1/hucct1 western blot bax bcl-2 n-cadherin e-cadherin/2024.4.13 hucct emt bax bcl-2/n-cad nc oe tubulin.png]

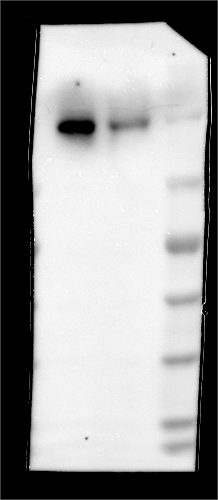

Supplement: Supplemental Information 4 — Western blot original strip, quantitative gray value and statistical map. [file peerj-12-18497-s004.zip › In all Figure , all the original western blot images, original gray value data and statistical graphs were obtained(In addition to overexpression and knock-down validation bands)/HUCCT1/hucct1 western blot bax bcl-2 n-cadherin e-cadherin/2024.4.13 hucct emt bax bcl-2/n-cad nc oe.png]

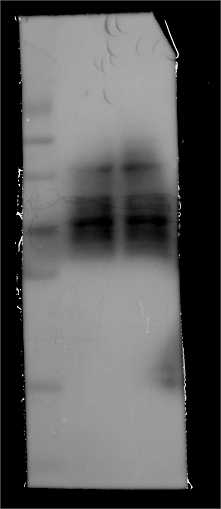

Supplement: Supplemental Information 4 — Western blot original strip, quantitative gray value and statistical map. [file peerj-12-18497-s004.zip › In all Figure , all the original western blot images, original gray value data and statistical graphs were obtained(In addition to overexpression and knock-down validation bands)/HUCCT1/hucct1 western blot bax bcl-2 n-cadherin e-cadherin/2024.4.15 hucct emt bax bcl-2/bax nc oe 第1张 gapdh.png]

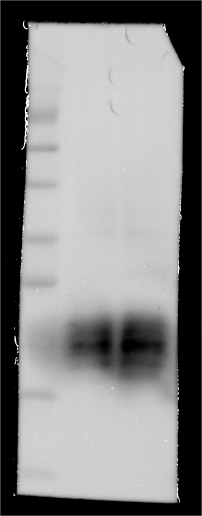

Supplement: Supplemental Information 4 — Western blot original strip, quantitative gray value and statistical map. [file peerj-12-18497-s004.zip › In all Figure , all the original western blot images, original gray value data and statistical graphs were obtained(In addition to overexpression and knock-down validation bands)/HUCCT1/hucct1 western blot bax bcl-2 n-cadherin e-cadherin/2024.4.15 hucct emt bax bcl-2/bax nc oe 第1张.png]

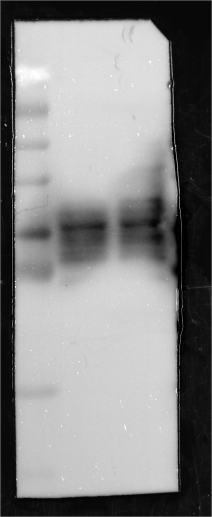

Supplement: Supplemental Information 4 — Western blot original strip, quantitative gray value and statistical map. [file peerj-12-18497-s004.zip › In all Figure , all the original western blot images, original gray value data and statistical graphs were obtained(In addition to overexpression and knock-down validation bands)/HUCCT1/hucct1 western blot bax bcl-2 n-cadherin e-cadherin/2024.4.15 hucct emt bax bcl-2/bax nc oe 第2张 gapdh.png]

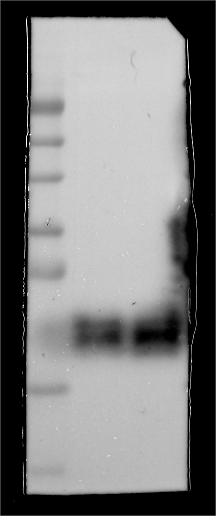

Supplement: Supplemental Information 4 — Western blot original strip, quantitative gray value and statistical map. [file peerj-12-18497-s004.zip › In all Figure , all the original western blot images, original gray value data and statistical graphs were obtained(In addition to overexpression and knock-down validation bands)/HUCCT1/hucct1 western blot bax bcl-2 n-cadherin e-cadherin/2024.4.15 hucct emt bax bcl-2/bax nc oe 第2张.png]

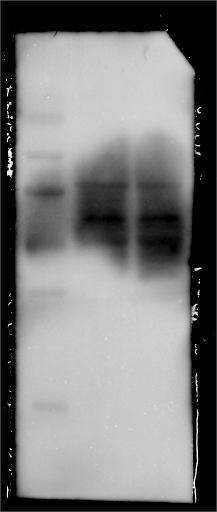

Supplement: Supplemental Information 4 — Western blot original strip, quantitative gray value and statistical map. [file peerj-12-18497-s004.zip › In all Figure , all the original western blot images, original gray value data and statistical graphs were obtained(In addition to overexpression and knock-down validation bands)/HUCCT1/hucct1 western blot bax bcl-2 n-cadherin e-cadherin/2024.4.15 hucct emt bax bcl-2/bcl-2 nc oe 第1张 gapdh.png]

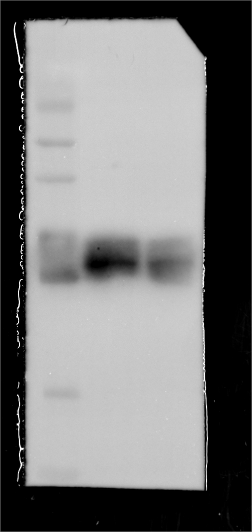

Supplement: Supplemental Information 4 — Western blot original strip, quantitative gray value and statistical map. [file peerj-12-18497-s004.zip › In all Figure , all the original western blot images, original gray value data and statistical graphs were obtained(In addition to overexpression and knock-down validation bands)/HUCCT1/hucct1 western blot bax bcl-2 n-cadherin e-cadherin/2024.4.15 hucct emt bax bcl-2/bcl-2 nc oe 第1张.png]

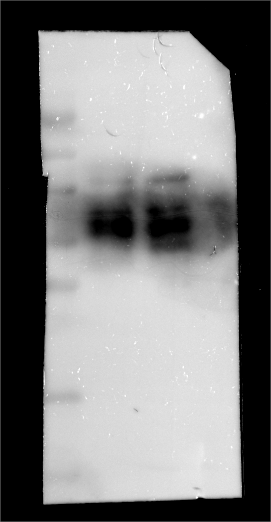

Supplement: Supplemental Information 4 — Western blot original strip, quantitative gray value and statistical map. [file peerj-12-18497-s004.zip › In all Figure , all the original western blot images, original gray value data and statistical graphs were obtained(In addition to overexpression and knock-down validation bands)/HUCCT1/hucct1 western blot bax bcl-2 n-cadherin e-cadherin/2024.4.15 hucct emt bax bcl-2/bcl-2 nc oe 第2张 gapdh.png]
