# Supplementary figures and images for: The CLEC3B inhibits cellular proliferation and metastasis of cholangiocarcinoma through Wnt/β-catenin pathway (part 2 of 5)
Source: PeerJ. 2024 Nov 13;12:e18497. doi: 10.7717/peerj.18497 (PMC11568818; doi:10.7717/peerj.18497)

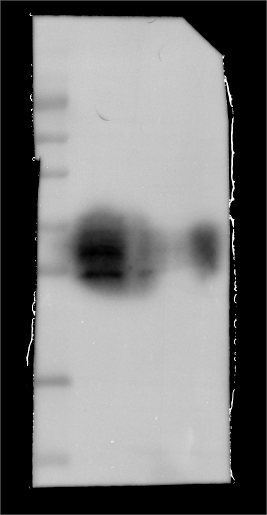

Supplement: Supplemental Information 4 — Western blot original strip, quantitative gray value and statistical map. [file peerj-12-18497-s004.zip › In all Figure , all the original western blot images, original gray value data and statistical graphs were obtained(In addition to overexpression and knock-down validation bands)/HUCCT1/hucct1 western blot bax bcl-2 n-cadherin e-cadherin/2024.4.15 hucct emt bax bcl-2/bcl-2 nc oe 第2张.png]

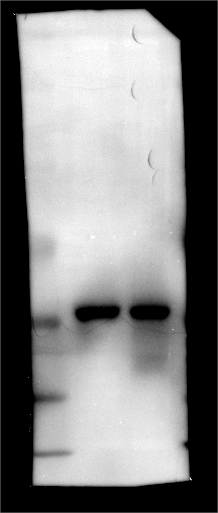

Supplement: Supplemental Information 4 — Western blot original strip, quantitative gray value and statistical map. [file peerj-12-18497-s004.zip › In all Figure , all the original western blot images, original gray value data and statistical graphs were obtained(In addition to overexpression and knock-down validation bands)/HUCCT1/hucct1 western blot bax bcl-2 n-cadherin e-cadherin/2024.4.15 hucct emt bax bcl-2/e-cad nc oe 第1张 tubulin.png]

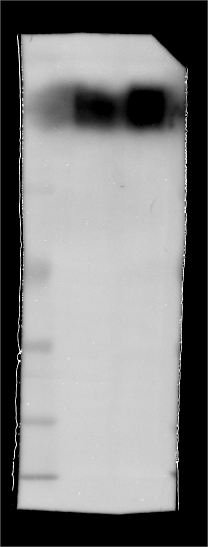

Supplement: Supplemental Information 4 — Western blot original strip, quantitative gray value and statistical map. [file peerj-12-18497-s004.zip › In all Figure , all the original western blot images, original gray value data and statistical graphs were obtained(In addition to overexpression and knock-down validation bands)/HUCCT1/hucct1 western blot bax bcl-2 n-cadherin e-cadherin/2024.4.15 hucct emt bax bcl-2/e-cad nc oe 第1张.png]

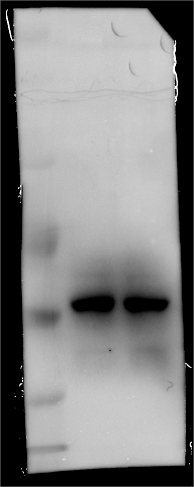

Supplement: Supplemental Information 4 — Western blot original strip, quantitative gray value and statistical map. [file peerj-12-18497-s004.zip › In all Figure , all the original western blot images, original gray value data and statistical graphs were obtained(In addition to overexpression and knock-down validation bands)/HUCCT1/hucct1 western blot bax bcl-2 n-cadherin e-cadherin/2024.4.15 hucct emt bax bcl-2/e-cad nc oe 第2张 tubulin.png]

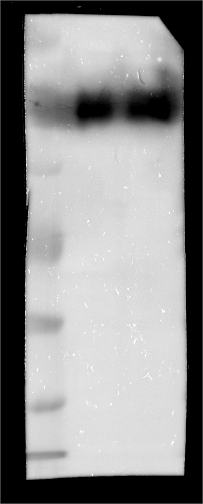

Supplement: Supplemental Information 4 — Western blot original strip, quantitative gray value and statistical map. [file peerj-12-18497-s004.zip › In all Figure , all the original western blot images, original gray value data and statistical graphs were obtained(In addition to overexpression and knock-down validation bands)/HUCCT1/hucct1 western blot bax bcl-2 n-cadherin e-cadherin/2024.4.15 hucct emt bax bcl-2/e-cad nc oe 第2张.png]

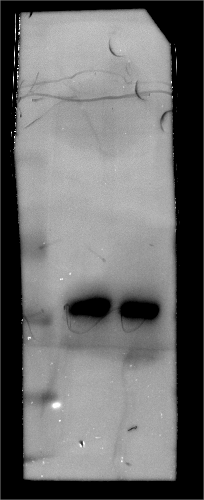

Supplement: Supplemental Information 4 — Western blot original strip, quantitative gray value and statistical map. [file peerj-12-18497-s004.zip › In all Figure , all the original western blot images, original gray value data and statistical graphs were obtained(In addition to overexpression and knock-down validation bands)/HUCCT1/hucct1 western blot bax bcl-2 n-cadherin e-cadherin/2024.4.15 hucct emt bax bcl-2/e-cad sicon si185 tubulin.png]

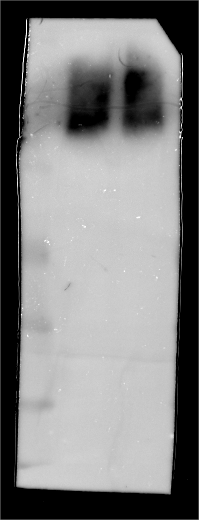

Supplement: Supplemental Information 4 — Western blot original strip, quantitative gray value and statistical map. [file peerj-12-18497-s004.zip › In all Figure , all the original western blot images, original gray value data and statistical graphs were obtained(In addition to overexpression and knock-down validation bands)/HUCCT1/hucct1 western blot bax bcl-2 n-cadherin e-cadherin/2024.4.15 hucct emt bax bcl-2/e-cad sicon si185.png]

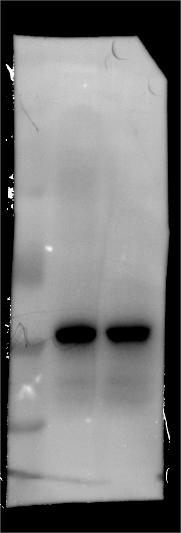

Supplement: Supplemental Information 4 — Western blot original strip, quantitative gray value and statistical map. [file peerj-12-18497-s004.zip › In all Figure , all the original western blot images, original gray value data and statistical graphs were obtained(In addition to overexpression and knock-down validation bands)/HUCCT1/hucct1 western blot bax bcl-2 n-cadherin e-cadherin/2024.4.15 hucct emt bax bcl-2/n-cad nc oe tubulin.png]

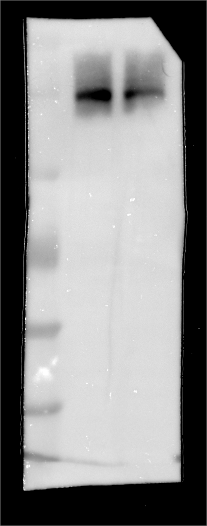

Supplement: Supplemental Information 4 — Western blot original strip, quantitative gray value and statistical map. [file peerj-12-18497-s004.zip › In all Figure , all the original western blot images, original gray value data and statistical graphs were obtained(In addition to overexpression and knock-down validation bands)/HUCCT1/hucct1 western blot bax bcl-2 n-cadherin e-cadherin/2024.4.15 hucct emt bax bcl-2/n-cad nc oe.png]

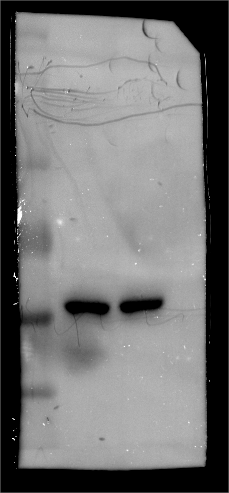

Supplement: Supplemental Information 4 — Western blot original strip, quantitative gray value and statistical map. [file peerj-12-18497-s004.zip › In all Figure , all the original western blot images, original gray value data and statistical graphs were obtained(In addition to overexpression and knock-down validation bands)/HUCCT1/hucct1 western blot bax bcl-2 n-cadherin e-cadherin/2024.4.15 hucct emt bax bcl-2/n-cad sicon si185 tubulin.png]

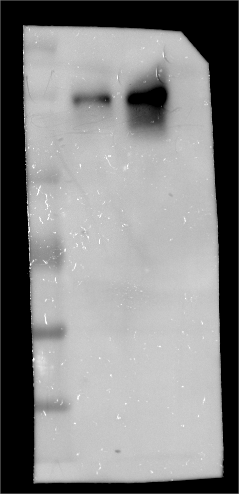

Supplement: Supplemental Information 4 — Western blot original strip, quantitative gray value and statistical map. [file peerj-12-18497-s004.zip › In all Figure , all the original western blot images, original gray value data and statistical graphs were obtained(In addition to overexpression and knock-down validation bands)/HUCCT1/hucct1 western blot bax bcl-2 n-cadherin e-cadherin/2024.4.15 hucct emt bax bcl-2/n-cad sicon si185.png]

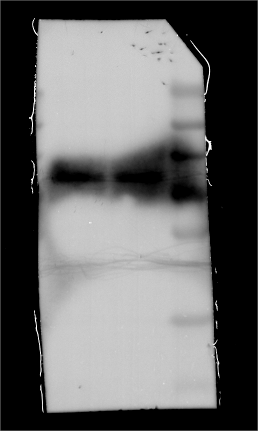

Supplement: Supplemental Information 4 — Western blot original strip, quantitative gray value and statistical map. [file peerj-12-18497-s004.zip › In all Figure , all the original western blot images, original gray value data and statistical graphs were obtained(In addition to overexpression and knock-down validation bands)/HUCCT1/hucct1 western blot bax bcl-2 n-cadherin e-cadherin/2024.4.17 hucct emt bax bcl-2/bax nc oe GAPDH.png]

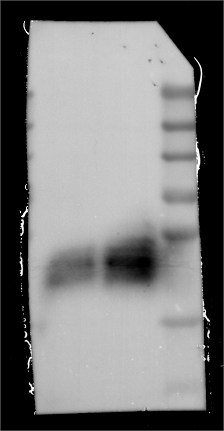

Supplement: Supplemental Information 4 — Western blot original strip, quantitative gray value and statistical map. [file peerj-12-18497-s004.zip › In all Figure , all the original western blot images, original gray value data and statistical graphs were obtained(In addition to overexpression and knock-down validation bands)/HUCCT1/hucct1 western blot bax bcl-2 n-cadherin e-cadherin/2024.4.17 hucct emt bax bcl-2/bax nc oe.png]

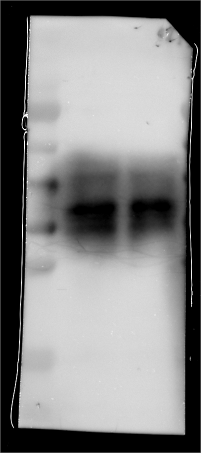

Supplement: Supplemental Information 4 — Western blot original strip, quantitative gray value and statistical map. [file peerj-12-18497-s004.zip › In all Figure , all the original western blot images, original gray value data and statistical graphs were obtained(In addition to overexpression and knock-down validation bands)/HUCCT1/hucct1 western blot bax bcl-2 n-cadherin e-cadherin/2024.4.17 hucct emt bax bcl-2/bcl-2 nc oe 第1张 GAPDH.png]

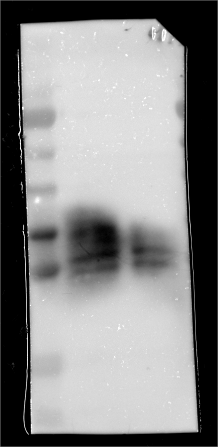

Supplement: Supplemental Information 4 — Western blot original strip, quantitative gray value and statistical map. [file peerj-12-18497-s004.zip › In all Figure , all the original western blot images, original gray value data and statistical graphs were obtained(In addition to overexpression and knock-down validation bands)/HUCCT1/hucct1 western blot bax bcl-2 n-cadherin e-cadherin/2024.4.17 hucct emt bax bcl-2/bcl-2 nc oe 第1张.png]

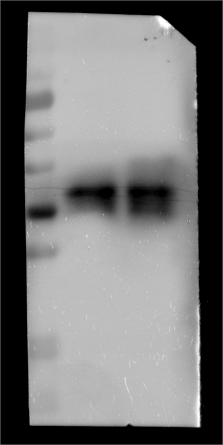

Supplement: Supplemental Information 4 — Western blot original strip, quantitative gray value and statistical map. [file peerj-12-18497-s004.zip › In all Figure , all the original western blot images, original gray value data and statistical graphs were obtained(In addition to overexpression and knock-down validation bands)/HUCCT1/hucct1 western blot bax bcl-2 n-cadherin e-cadherin/2024.4.17 hucct emt bax bcl-2/bcl-2 sicon si185 第1张 GAPDH.png]

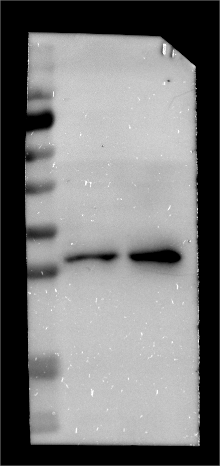

Supplement: Supplemental Information 4 — Western blot original strip, quantitative gray value and statistical map. [file peerj-12-18497-s004.zip › In all Figure , all the original western blot images, original gray value data and statistical graphs were obtained(In addition to overexpression and knock-down validation bands)/HUCCT1/hucct1 western blot bax bcl-2 n-cadherin e-cadherin/2024.4.17 hucct emt bax bcl-2/bcl-2 sicon si185 第1张.png]

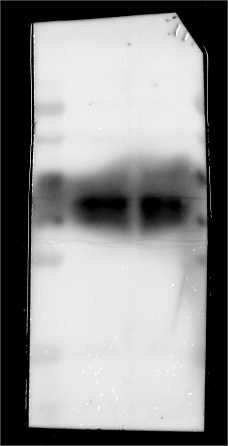

Supplement: Supplemental Information 4 — Western blot original strip, quantitative gray value and statistical map. [file peerj-12-18497-s004.zip › In all Figure , all the original western blot images, original gray value data and statistical graphs were obtained(In addition to overexpression and knock-down validation bands)/HUCCT1/hucct1 western blot bax bcl-2 n-cadherin e-cadherin/2024.4.17 hucct emt bax bcl-2/bcl-2 sicon si185 第2张 GAPDH.png]

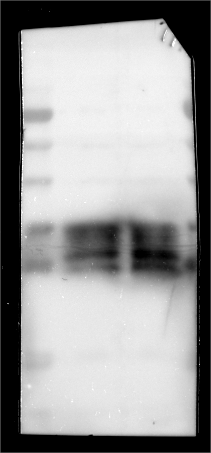

Supplement: Supplemental Information 4 — Western blot original strip, quantitative gray value and statistical map. [file peerj-12-18497-s004.zip › In all Figure , all the original western blot images, original gray value data and statistical graphs were obtained(In addition to overexpression and knock-down validation bands)/HUCCT1/hucct1 western blot bax bcl-2 n-cadherin e-cadherin/2024.4.17 hucct emt bax bcl-2/bcl-2 sicon si185 第2张.png]

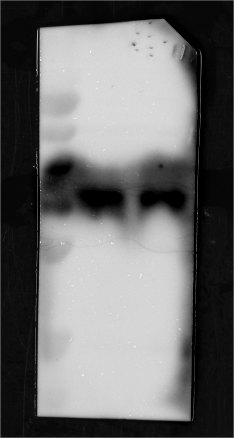

Supplement: Supplemental Information 4 — Western blot original strip, quantitative gray value and statistical map. [file peerj-12-18497-s004.zip › In all Figure , all the original western blot images, original gray value data and statistical graphs were obtained(In addition to overexpression and knock-down validation bands)/HUCCT1/hucct1 western blot bax bcl-2 n-cadherin e-cadherin/2024.4.17 hucct emt bax bcl-2/bcl-2 sicon si185 第3张 GAPDH.png]

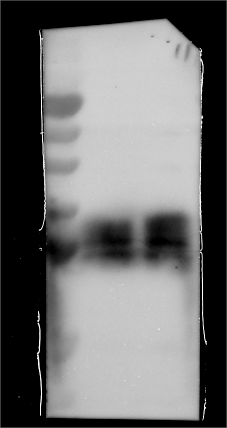

Supplement: Supplemental Information 4 — Western blot original strip, quantitative gray value and statistical map. [file peerj-12-18497-s004.zip › In all Figure , all the original western blot images, original gray value data and statistical graphs were obtained(In addition to overexpression and knock-down validation bands)/HUCCT1/hucct1 western blot bax bcl-2 n-cadherin e-cadherin/2024.4.17 hucct emt bax bcl-2/bcl-2 sicon si185 第3张.png]

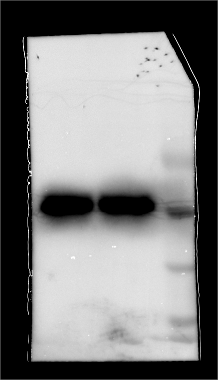

Supplement: Supplemental Information 4 — Western blot original strip, quantitative gray value and statistical map. [file peerj-12-18497-s004.zip › In all Figure , all the original western blot images, original gray value data and statistical graphs were obtained(In addition to overexpression and knock-down validation bands)/HUCCT1/hucct1 western blot bax bcl-2 n-cadherin e-cadherin/2024.4.17 hucct emt bax bcl-2/e-cad nc oe 第1张 TUBULIN.png]

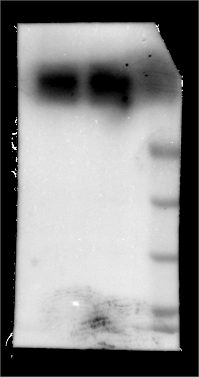

Supplement: Supplemental Information 4 — Western blot original strip, quantitative gray value and statistical map. [file peerj-12-18497-s004.zip › In all Figure , all the original western blot images, original gray value data and statistical graphs were obtained(In addition to overexpression and knock-down validation bands)/HUCCT1/hucct1 western blot bax bcl-2 n-cadherin e-cadherin/2024.4.17 hucct emt bax bcl-2/e-cad nc oe 第1张.png]

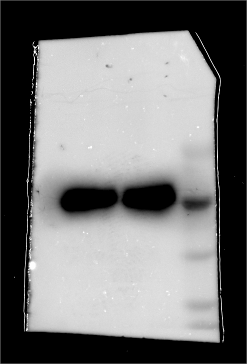

Supplement: Supplemental Information 4 — Western blot original strip, quantitative gray value and statistical map. [file peerj-12-18497-s004.zip › In all Figure , all the original western blot images, original gray value data and statistical graphs were obtained(In addition to overexpression and knock-down validation bands)/HUCCT1/hucct1 western blot bax bcl-2 n-cadherin e-cadherin/2024.4.17 hucct emt bax bcl-2/e-cad nc oe 第2张 TUBULIN.png]

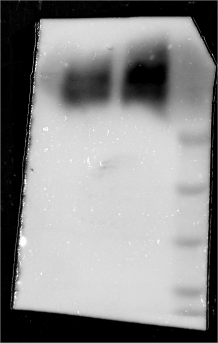

Supplement: Supplemental Information 4 — Western blot original strip, quantitative gray value and statistical map. [file peerj-12-18497-s004.zip › In all Figure , all the original western blot images, original gray value data and statistical graphs were obtained(In addition to overexpression and knock-down validation bands)/HUCCT1/hucct1 western blot bax bcl-2 n-cadherin e-cadherin/2024.4.17 hucct emt bax bcl-2/e-cad nc oe 第2张.png]

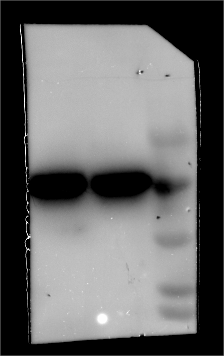

Supplement: Supplemental Information 4 — Western blot original strip, quantitative gray value and statistical map. [file peerj-12-18497-s004.zip › In all Figure , all the original western blot images, original gray value data and statistical graphs were obtained(In addition to overexpression and knock-down validation bands)/HUCCT1/hucct1 western blot bax bcl-2 n-cadherin e-cadherin/2024.4.17 hucct emt bax bcl-2/e-cad nc oe 第3张 TUBULIN.png]

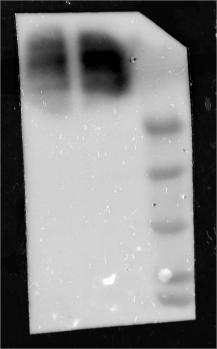

Supplement: Supplemental Information 4 — Western blot original strip, quantitative gray value and statistical map. [file peerj-12-18497-s004.zip › In all Figure , all the original western blot images, original gray value data and statistical graphs were obtained(In addition to overexpression and knock-down validation bands)/HUCCT1/hucct1 western blot bax bcl-2 n-cadherin e-cadherin/2024.4.17 hucct emt bax bcl-2/e-cad nc oe 第3张.png]

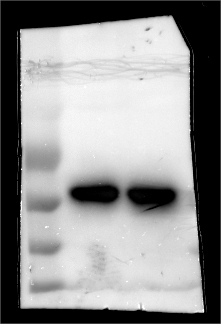

Supplement: Supplemental Information 4 — Western blot original strip, quantitative gray value and statistical map. [file peerj-12-18497-s004.zip › In all Figure , all the original western blot images, original gray value data and statistical graphs were obtained(In addition to overexpression and knock-down validation bands)/HUCCT1/hucct1 western blot bax bcl-2 n-cadherin e-cadherin/2024.4.17 hucct emt bax bcl-2/e-cad sicon si185 第1张 TUBULIN.pn]

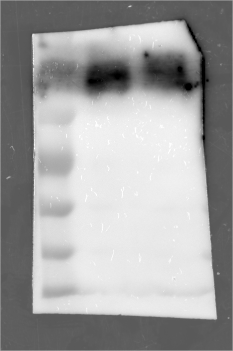

Supplement: Supplemental Information 4 — Western blot original strip, quantitative gray value and statistical map. [file peerj-12-18497-s004.zip › In all Figure , all the original western blot images, original gray value data and statistical graphs were obtained(In addition to overexpression and knock-down validation bands)/HUCCT1/hucct1 western blot bax bcl-2 n-cadherin e-cadherin/2024.4.17 hucct emt bax bcl-2/e-cad sicon si185 第1张.png]

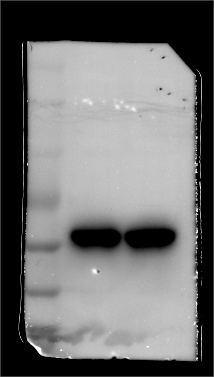

Supplement: Supplemental Information 4 — Western blot original strip, quantitative gray value and statistical map. [file peerj-12-18497-s004.zip › In all Figure , all the original western blot images, original gray value data and statistical graphs were obtained(In addition to overexpression and knock-down validation bands)/HUCCT1/hucct1 western blot bax bcl-2 n-cadherin e-cadherin/2024.4.17 hucct emt bax bcl-2/e-cad sicon si185 第2张 TUBULIN.pn]

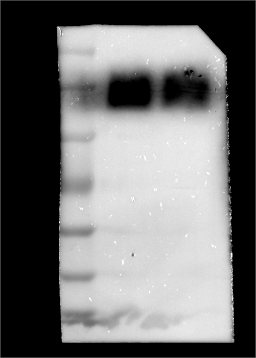

Supplement: Supplemental Information 4 — Western blot original strip, quantitative gray value and statistical map. [file peerj-12-18497-s004.zip › In all Figure , all the original western blot images, original gray value data and statistical graphs were obtained(In addition to overexpression and knock-down validation bands)/HUCCT1/hucct1 western blot bax bcl-2 n-cadherin e-cadherin/2024.4.17 hucct emt bax bcl-2/e-cad sicon si185 第2张.png]

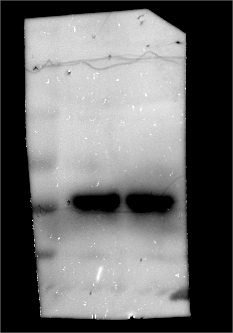

Supplement: Supplemental Information 4 — Western blot original strip, quantitative gray value and statistical map. [file peerj-12-18497-s004.zip › In all Figure , all the original western blot images, original gray value data and statistical graphs were obtained(In addition to overexpression and knock-down validation bands)/HUCCT1/hucct1 western blot bax bcl-2 n-cadherin e-cadherin/2024.4.17 hucct emt bax bcl-2/n-cad nc oe TUBULIN.png]

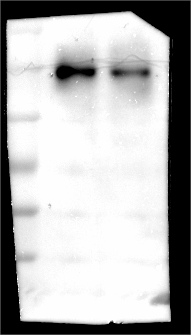

Supplement: Supplemental Information 4 — Western blot original strip, quantitative gray value and statistical map. [file peerj-12-18497-s004.zip › In all Figure , all the original western blot images, original gray value data and statistical graphs were obtained(In addition to overexpression and knock-down validation bands)/HUCCT1/hucct1 western blot bax bcl-2 n-cadherin e-cadherin/2024.4.17 hucct emt bax bcl-2/n-cad nc oe.png]

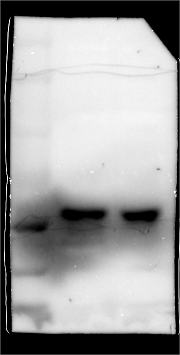

Supplement: Supplemental Information 4 — Western blot original strip, quantitative gray value and statistical map. [file peerj-12-18497-s004.zip › In all Figure , all the original western blot images, original gray value data and statistical graphs were obtained(In addition to overexpression and knock-down validation bands)/HUCCT1/hucct1 western blot bax bcl-2 n-cadherin e-cadherin/2024.4.17 hucct emt bax bcl-2/n-cad sicon si185 第1张 TUBULIN.pn]

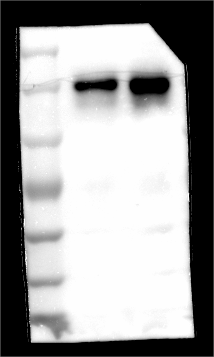

Supplement: Supplemental Information 4 — Western blot original strip, quantitative gray value and statistical map. [file peerj-12-18497-s004.zip › In all Figure , all the original western blot images, original gray value data and statistical graphs were obtained(In addition to overexpression and knock-down validation bands)/HUCCT1/hucct1 western blot bax bcl-2 n-cadherin e-cadherin/2024.4.17 hucct emt bax bcl-2/n-cad sicon si185 第1张.png]

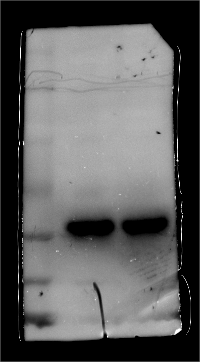

Supplement: Supplemental Information 4 — Western blot original strip, quantitative gray value and statistical map. [file peerj-12-18497-s004.zip › In all Figure , all the original western blot images, original gray value data and statistical graphs were obtained(In addition to overexpression and knock-down validation bands)/HUCCT1/hucct1 western blot bax bcl-2 n-cadherin e-cadherin/2024.4.17 hucct emt bax bcl-2/n-cad sicon si185 第2张 TUBULIN.pn]

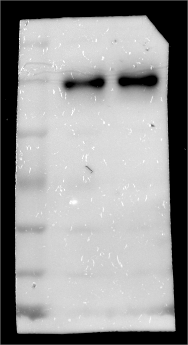

Supplement: Supplemental Information 4 — Western blot original strip, quantitative gray value and statistical map. [file peerj-12-18497-s004.zip › In all Figure , all the original western blot images, original gray value data and statistical graphs were obtained(In addition to overexpression and knock-down validation bands)/HUCCT1/hucct1 western blot bax bcl-2 n-cadherin e-cadherin/2024.4.17 hucct emt bax bcl-2/n-cad sicon si185 第2张.png]

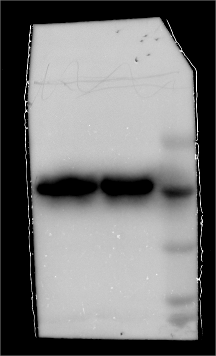

Supplement: Supplemental Information 4 — Western blot original strip, quantitative gray value and statistical map. [file peerj-12-18497-s004.zip › In all Figure , all the original western blot images, original gray value data and statistical graphs were obtained(In addition to overexpression and knock-down validation bands)/HUCCT1/hucct1 western blot bax bcl-2 n-cadherin e-cadherin/2024.4.17 hucct emt bax bcl-2/n-cad sicon si185 第3张 TUBULIN.pn]

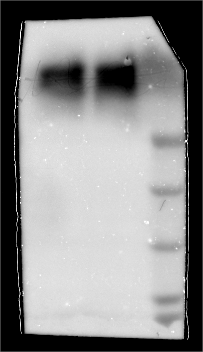

Supplement: Supplemental Information 4 — Western blot original strip, quantitative gray value and statistical map. [file peerj-12-18497-s004.zip › In all Figure , all the original western blot images, original gray value data and statistical graphs were obtained(In addition to overexpression and knock-down validation bands)/HUCCT1/hucct1 western blot bax bcl-2 n-cadherin e-cadherin/2024.4.17 hucct emt bax bcl-2/n-cad sicon si185 第3张.png]

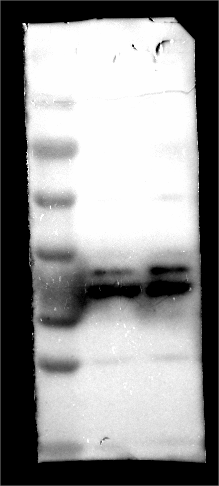

Supplement: Supplemental Information 4 — Western blot original strip, quantitative gray value and statistical map. [file peerj-12-18497-s004.zip › In all Figure , all the original western blot images, original gray value data and statistical graphs were obtained(In addition to overexpression and knock-down validation bands)/qbc939/qbc939 over expression clec3b(nc oe) and knock down clec3b (sicon si185)/2024.3.10 QBC 939 clec3b nc oe sicon si18]

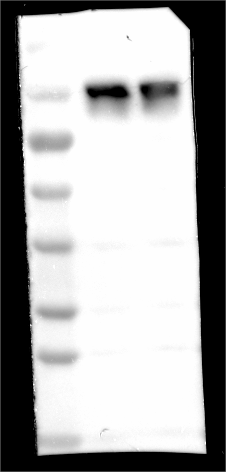

Supplement: Supplemental Information 4 — Western blot original strip, quantitative gray value and statistical map. [file peerj-12-18497-s004.zip › In all Figure , all the original western blot images, original gray value data and statistical graphs were obtained(In addition to overexpression and knock-down validation bands)/qbc939/qbc939 over expression clec3b(nc oe) and knock down clec3b (sicon si185)/2024.3.10 QBC 939 clec3b nc oe sicon si18]

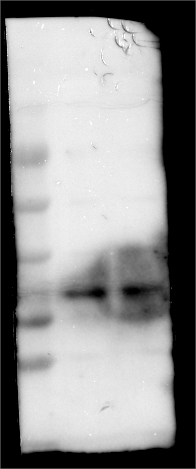

Supplement: Supplemental Information 4 — Western blot original strip, quantitative gray value and statistical map. [file peerj-12-18497-s004.zip › In all Figure , all the original western blot images, original gray value data and statistical graphs were obtained(In addition to overexpression and knock-down validation bands)/qbc939/qbc939 over expression clec3b(nc oe) and knock down clec3b (sicon si185)/2024.3.10 QBC 939 clec3b nc oe sicon si18]

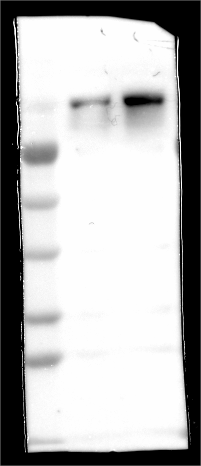

Supplement: Supplemental Information 4 — Western blot original strip, quantitative gray value and statistical map. [file peerj-12-18497-s004.zip › In all Figure , all the original western blot images, original gray value data and statistical graphs were obtained(In addition to overexpression and knock-down validation bands)/qbc939/qbc939 over expression clec3b(nc oe) and knock down clec3b (sicon si185)/2024.3.10 QBC 939 clec3b nc oe sicon si18]

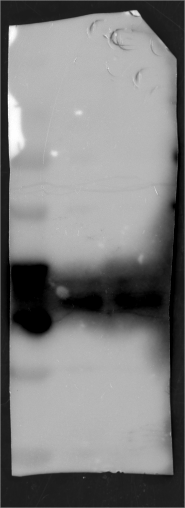

Supplement: Supplemental Information 4 — Western blot original strip, quantitative gray value and statistical map. [file peerj-12-18497-s004.zip › In all Figure , all the original western blot images, original gray value data and statistical graphs were obtained(In addition to overexpression and knock-down validation bands)/qbc939/qbc939 over expression clec3b(nc oe) and knock down clec3b (sicon si185)/2024.3.10 QBC 939 clec3b nc oe sicon si18]

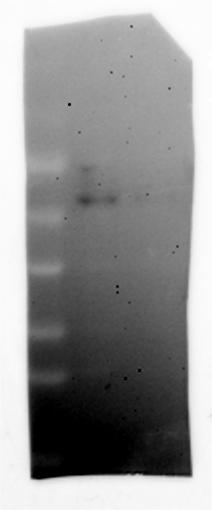

Supplement: Supplemental Information 4 — Western blot original strip, quantitative gray value and statistical map. [file peerj-12-18497-s004.zip › In all Figure , all the original western blot images, original gray value data and statistical graphs were obtained(In addition to overexpression and knock-down validation bands)/qbc939/qbc939 over expression clec3b(nc oe) and knock down clec3b (sicon si185)/2024.3.10 QBC 939 clec3b nc oe sicon si18]

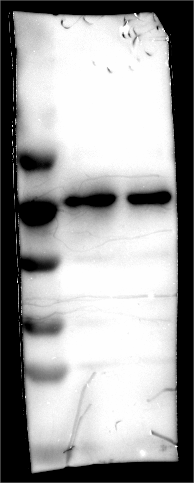

Supplement: Supplemental Information 4 — Western blot original strip, quantitative gray value and statistical map. [file peerj-12-18497-s004.zip › In all Figure , all the original western blot images, original gray value data and statistical graphs were obtained(In addition to overexpression and knock-down validation bands)/qbc939/qbc939 over expression clec3b(nc oe) and knock down clec3b (sicon si185)/2024.3.10 QBC 939 clec3b nc oe sicon si18]

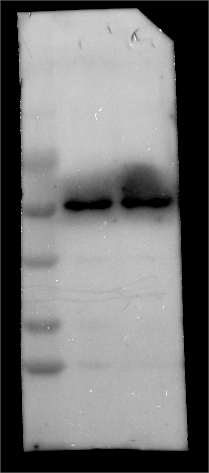

Supplement: Supplemental Information 4 — Western blot original strip, quantitative gray value and statistical map. [file peerj-12-18497-s004.zip › In all Figure , all the original western blot images, original gray value data and statistical graphs were obtained(In addition to overexpression and knock-down validation bands)/qbc939/qbc939 over expression clec3b(nc oe) and knock down clec3b (sicon si185)/2024.3.10 QBC 939 clec3b nc oe sicon si18]

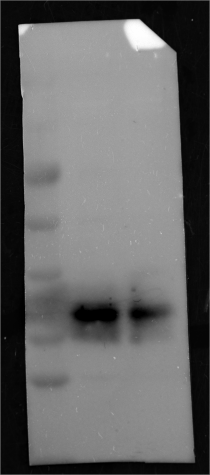

Supplement: Supplemental Information 4 — Western blot original strip, quantitative gray value and statistical map. [file peerj-12-18497-s004.zip › In all Figure , all the original western blot images, original gray value data and statistical graphs were obtained(In addition to overexpression and knock-down validation bands)/qbc939/qbc939 over expression clec3b(nc oe) and knock down clec3b (sicon si185)/2024.3.10 QBC 939 clec3b nc oe sicon si18]

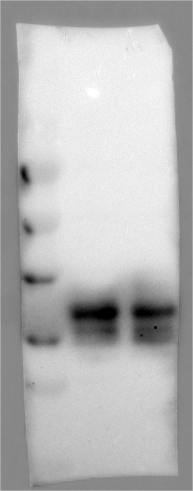

Supplement: Supplemental Information 4 — Western blot original strip, quantitative gray value and statistical map. [file peerj-12-18497-s004.zip › In all Figure , all the original western blot images, original gray value data and statistical graphs were obtained(In addition to overexpression and knock-down validation bands)/qbc939/qbc939 over expression clec3b(nc oe) and knock down clec3b (sicon si185)/2024.3.10 QBC 939 clec3b nc oe sicon si18]

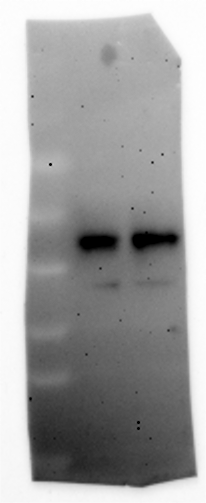

Supplement: Supplemental Information 4 — Western blot original strip, quantitative gray value and statistical map. [file peerj-12-18497-s004.zip › In all Figure , all the original western blot images, original gray value data and statistical graphs were obtained(In addition to overexpression and knock-down validation bands)/qbc939/qbc939 over expression clec3b(nc oe) and knock down clec3b (sicon si185)/2024.3.10 QBC 939 clec3b nc oe sicon si18]

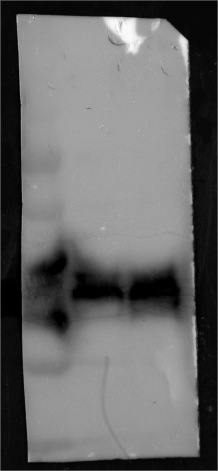

Supplement: Supplemental Information 4 — Western blot original strip, quantitative gray value and statistical map. [file peerj-12-18497-s004.zip › In all Figure , all the original western blot images, original gray value data and statistical graphs were obtained(In addition to overexpression and knock-down validation bands)/qbc939/qbc939 over expression clec3b(nc oe) and knock down clec3b (sicon si185)/2024.3.10 QBC 939 clec3b nc oe sicon si18]

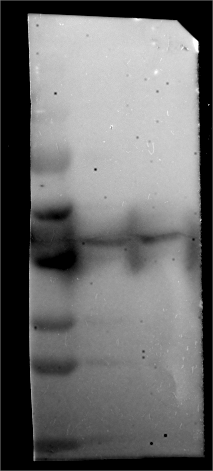

Supplement: Supplemental Information 4 — Western blot original strip, quantitative gray value and statistical map. [file peerj-12-18497-s004.zip › In all Figure , all the original western blot images, original gray value data and statistical graphs were obtained(In addition to overexpression and knock-down validation bands)/qbc939/qbc939 over expression clec3b(nc oe) and knock down clec3b (sicon si185)/2024.3.10 QBC 939 clec3b nc oe sicon si18]

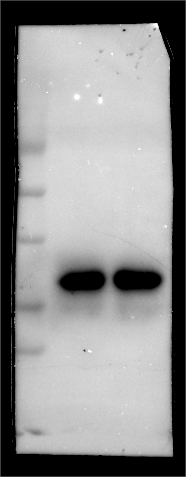

Supplement: Supplemental Information 4 — Western blot original strip, quantitative gray value and statistical map. [file peerj-12-18497-s004.zip › In all Figure , all the original western blot images, original gray value data and statistical graphs were obtained(In addition to overexpression and knock-down validation bands)/qbc939/qbc939 over expression clec3b(nc oe) and knock down clec3b (sicon si185)/2024.3.14 QBC 939 clec3b/CLEC3B NC OE 第3张]

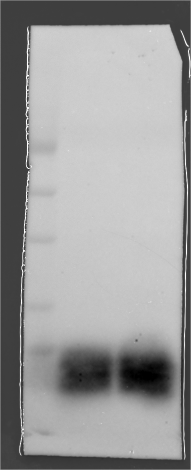

Supplement: Supplemental Information 4 — Western blot original strip, quantitative gray value and statistical map. [file peerj-12-18497-s004.zip › In all Figure , all the original western blot images, original gray value data and statistical graphs were obtained(In addition to overexpression and knock-down validation bands)/qbc939/qbc939 over expression clec3b(nc oe) and knock down clec3b (sicon si185)/2024.3.14 QBC 939 clec3b/CLEC3B NC OE 第3张]

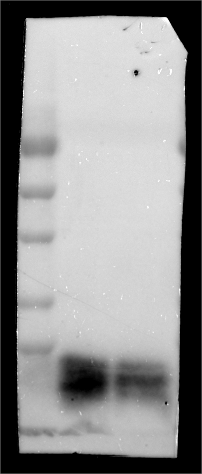

Supplement: Supplemental Information 4 — Western blot original strip, quantitative gray value and statistical map. [file peerj-12-18497-s004.zip › In all Figure , all the original western blot images, original gray value data and statistical graphs were obtained(In addition to overexpression and knock-down validation bands)/qbc939/qbc939 over expression clec3b(nc oe) and knock down clec3b (sicon si185)/2024.3.14 QBC 939 clec3b/CLEC3B sicon si1]

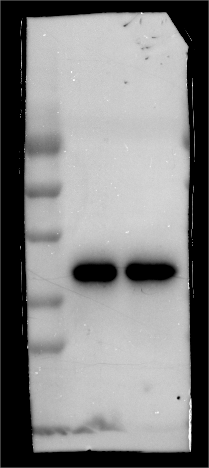

Supplement: Supplemental Information 4 — Western blot original strip, quantitative gray value and statistical map. [file peerj-12-18497-s004.zip › In all Figure , all the original western blot images, original gray value data and statistical graphs were obtained(In addition to overexpression and knock-down validation bands)/qbc939/qbc939 over expression clec3b(nc oe) and knock down clec3b (sicon si185)/2024.3.14 QBC 939 clec3b/CLEC3B sicon si1]

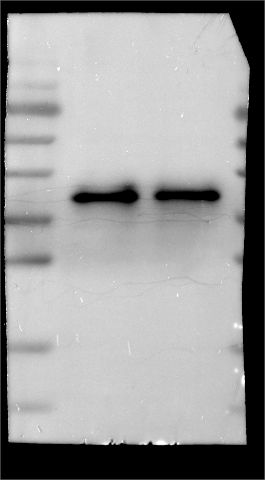

Supplement: Supplemental Information 4 — Western blot original strip, quantitative gray value and statistical map. [file peerj-12-18497-s004.zip › In all Figure , all the original western blot images, original gray value data and statistical graphs were obtained(In addition to overexpression and knock-down validation bands)/qbc939/qbc939 over expression clec3b(nc oe) and knock down clec3b (sicon si185)/2024.3.14 QBC 939 clec3b/clec3b nc oe 第1张]

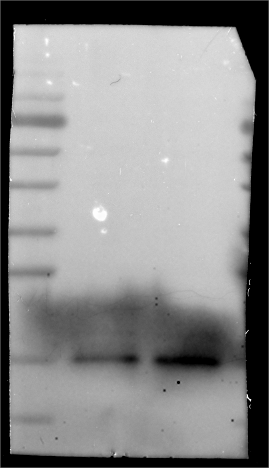

Supplement: Supplemental Information 4 — Western blot original strip, quantitative gray value and statistical map. [file peerj-12-18497-s004.zip › In all Figure , all the original western blot images, original gray value data and statistical graphs were obtained(In addition to overexpression and knock-down validation bands)/qbc939/qbc939 over expression clec3b(nc oe) and knock down clec3b (sicon si185)/2024.3.14 QBC 939 clec3b/clec3b nc oe 第1张]

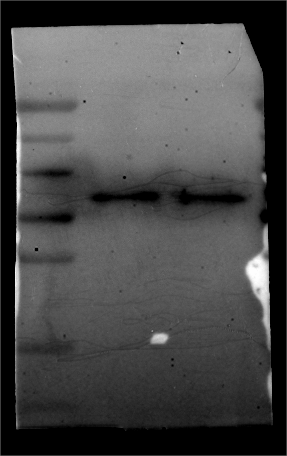

Supplement: Supplemental Information 4 — Western blot original strip, quantitative gray value and statistical map. [file peerj-12-18497-s004.zip › In all Figure , all the original western blot images, original gray value data and statistical graphs were obtained(In addition to overexpression and knock-down validation bands)/qbc939/qbc939 over expression clec3b(nc oe) and knock down clec3b (sicon si185)/2024.3.14 QBC 939 clec3b/clec3b nc oe 第2张]

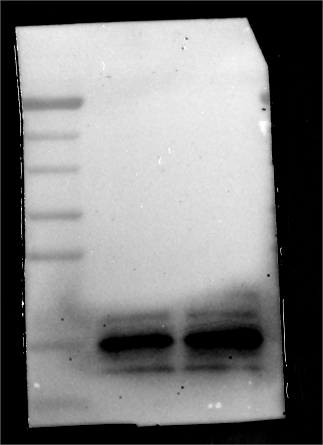

Supplement: Supplemental Information 4 — Western blot original strip, quantitative gray value and statistical map. [file peerj-12-18497-s004.zip › In all Figure , all the original western blot images, original gray value data and statistical graphs were obtained(In addition to overexpression and knock-down validation bands)/qbc939/qbc939 over expression clec3b(nc oe) and knock down clec3b (sicon si185)/2024.3.14 QBC 939 clec3b/clec3b nc oe 第2张]

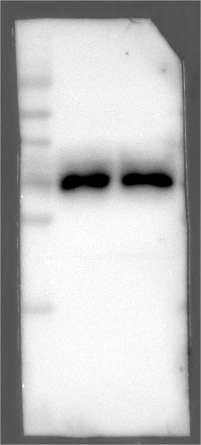

Supplement: Supplemental Information 4 — Western blot original strip, quantitative gray value and statistical map. [file peerj-12-18497-s004.zip › In all Figure , all the original western blot images, original gray value data and statistical graphs were obtained(In addition to overexpression and knock-down validation bands)/qbc939/qbc939 over expression clec3b(nc oe) and knock down clec3b (sicon si185)/2024.3.14 QBC 939 clec3b/clec3b nc oe 第5张]

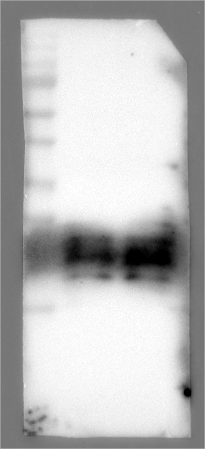

Supplement: Supplemental Information 4 — Western blot original strip, quantitative gray value and statistical map. [file peerj-12-18497-s004.zip › In all Figure , all the original western blot images, original gray value data and statistical graphs were obtained(In addition to overexpression and knock-down validation bands)/qbc939/qbc939 over expression clec3b(nc oe) and knock down clec3b (sicon si185)/2024.3.14 QBC 939 clec3b/clec3b nc oe 第5张]

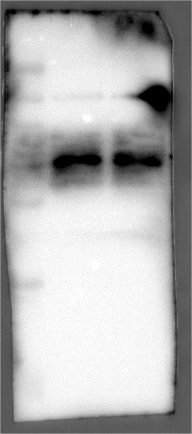

Supplement: Supplemental Information 4 — Western blot original strip, quantitative gray value and statistical map. [file peerj-12-18497-s004.zip › In all Figure , all the original western blot images, original gray value data and statistical graphs were obtained(In addition to overexpression and knock-down validation bands)/qbc939/qbc939 over expression clec3b(nc oe) and knock down clec3b (sicon si185)/2024.3.14 QBC 939 clec3b/clec3b nc oe 第6张]

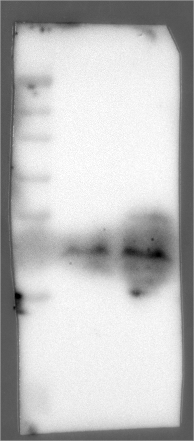

Supplement: Supplemental Information 4 — Western blot original strip, quantitative gray value and statistical map. [file peerj-12-18497-s004.zip › In all Figure , all the original western blot images, original gray value data and statistical graphs were obtained(In addition to overexpression and knock-down validation bands)/qbc939/qbc939 over expression clec3b(nc oe) and knock down clec3b (sicon si185)/2024.3.14 QBC 939 clec3b/clec3b nc oe 第6张]

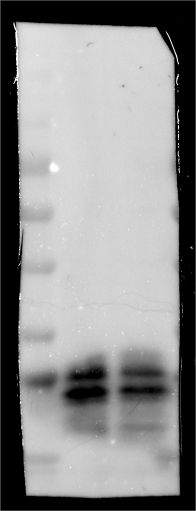

Supplement: Supplemental Information 4 — Western blot original strip, quantitative gray value and statistical map. [file peerj-12-18497-s004.zip › In all Figure , all the original western blot images, original gray value data and statistical graphs were obtained(In addition to overexpression and knock-down validation bands)/qbc939/qbc939 over expression clec3b(nc oe) and knock down clec3b (sicon si185)/2024.3.14 QBC 939 clec3b/clec3b sicon si1]

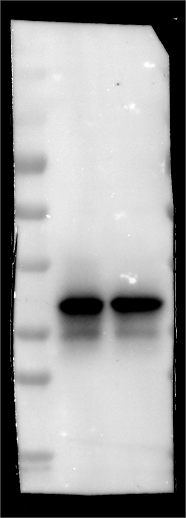

Supplement: Supplemental Information 4 — Western blot original strip, quantitative gray value and statistical map. [file peerj-12-18497-s004.zip › In all Figure , all the original western blot images, original gray value data and statistical graphs were obtained(In addition to overexpression and knock-down validation bands)/qbc939/qbc939 over expression clec3b(nc oe) and knock down clec3b (sicon si185)/2024.3.14 QBC 939 clec3b/clec3b sicon si1]

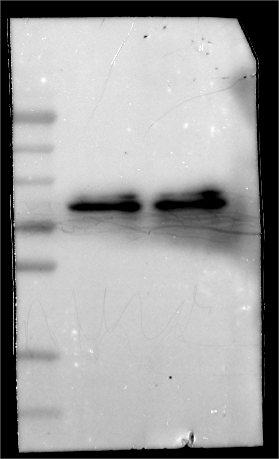

Supplement: Supplemental Information 4 — Western blot original strip, quantitative gray value and statistical map. [file peerj-12-18497-s004.zip › In all Figure , all the original western blot images, original gray value data and statistical graphs were obtained(In addition to overexpression and knock-down validation bands)/qbc939/qbc939 over expression clec3b(nc oe) and knock down clec3b (sicon si185)/2024.3.14 QBC 939 clec3b/clec3b sicon si1]

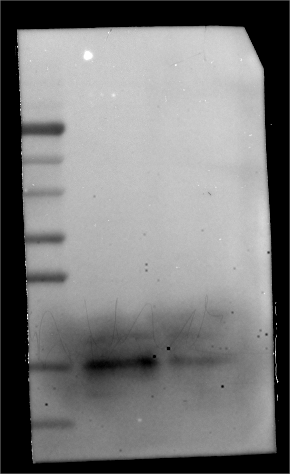

Supplement: Supplemental Information 4 — Western blot original strip, quantitative gray value and statistical map. [file peerj-12-18497-s004.zip › In all Figure , all the original western blot images, original gray value data and statistical graphs were obtained(In addition to overexpression and knock-down validation bands)/qbc939/qbc939 over expression clec3b(nc oe) and knock down clec3b (sicon si185)/2024.3.14 QBC 939 clec3b/clec3b sicon si1]

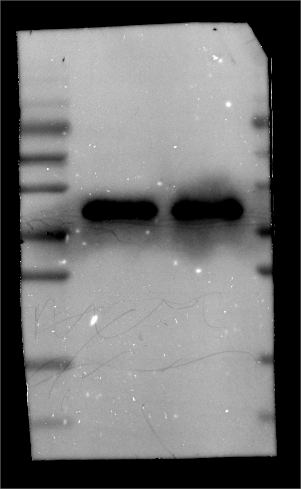

Supplement: Supplemental Information 4 — Western blot original strip, quantitative gray value and statistical map. [file peerj-12-18497-s004.zip › In all Figure , all the original western blot images, original gray value data and statistical graphs were obtained(In addition to overexpression and knock-down validation bands)/qbc939/qbc939 over expression clec3b(nc oe) and knock down clec3b (sicon si185)/2024.3.14 QBC 939 clec3b/clec3b sicon si1]

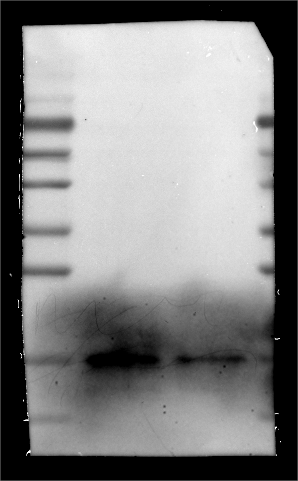

Supplement: Supplemental Information 4 — Western blot original strip, quantitative gray value and statistical map. [file peerj-12-18497-s004.zip › In all Figure , all the original western blot images, original gray value data and statistical graphs were obtained(In addition to overexpression and knock-down validation bands)/qbc939/qbc939 over expression clec3b(nc oe) and knock down clec3b (sicon si185)/2024.3.14 QBC 939 clec3b/clec3b sicon si1]

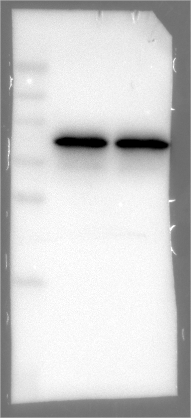

Supplement: Supplemental Information 4 — Western blot original strip, quantitative gray value and statistical map. [file peerj-12-18497-s004.zip › In all Figure , all the original western blot images, original gray value data and statistical graphs were obtained(In addition to overexpression and knock-down validation bands)/qbc939/qbc939 over expression clec3b(nc oe) and knock down clec3b (sicon si185)/2024.3.14 QBC 939 clec3b/clec3b sicon si1]

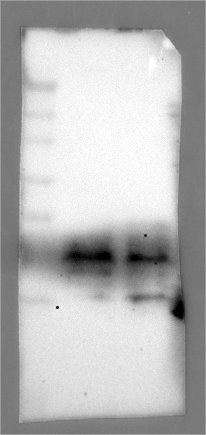

Supplement: Supplemental Information 4 — Western blot original strip, quantitative gray value and statistical map. [file peerj-12-18497-s004.zip › In all Figure , all the original western blot images, original gray value data and statistical graphs were obtained(In addition to overexpression and knock-down validation bands)/qbc939/qbc939 over expression clec3b(nc oe) and knock down clec3b (sicon si185)/2024.3.14 QBC 939 clec3b/clec3b sicon si1]

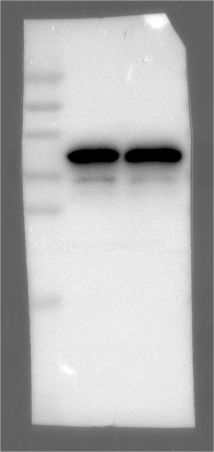

Supplement: Supplemental Information 4 — Western blot original strip, quantitative gray value and statistical map. [file peerj-12-18497-s004.zip › In all Figure , all the original western blot images, original gray value data and statistical graphs were obtained(In addition to overexpression and knock-down validation bands)/qbc939/qbc939 over expression clec3b(nc oe) and knock down clec3b (sicon si185)/2024.3.14 QBC 939 clec3b/clec3b sicon si1]

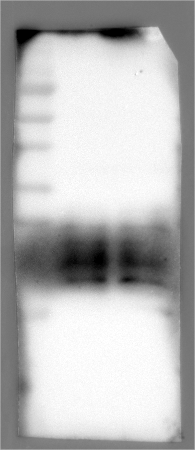

Supplement: Supplemental Information 4 — Western blot original strip, quantitative gray value and statistical map. [file peerj-12-18497-s004.zip › In all Figure , all the original western blot images, original gray value data and statistical graphs were obtained(In addition to overexpression and knock-down validation bands)/qbc939/qbc939 over expression clec3b(nc oe) and knock down clec3b (sicon si185)/2024.3.14 QBC 939 clec3b/clec3b sicon si1]

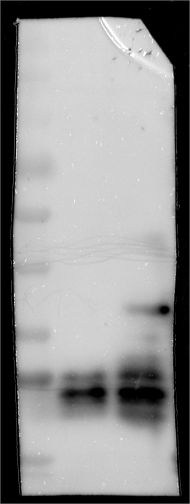

Supplement: Supplemental Information 4 — Western blot original strip, quantitative gray value and statistical map. [file peerj-12-18497-s004.zip › In all Figure , all the original western blot images, original gray value data and statistical graphs were obtained(In addition to overexpression and knock-down validation bands)/qbc939/qbc939 over expression clec3b(nc oe) and knock down clec3b (sicon si185)/2024.3.14 QBC 939 clec3b/clec3b nc oe 第4张]

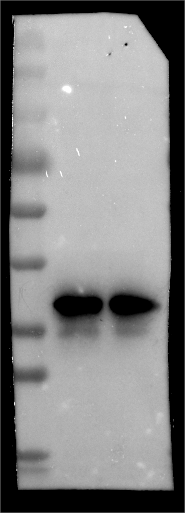

Supplement: Supplemental Information 4 — Western blot original strip, quantitative gray value and statistical map. [file peerj-12-18497-s004.zip › In all Figure , all the original western blot images, original gray value data and statistical graphs were obtained(In addition to overexpression and knock-down validation bands)/qbc939/qbc939 over expression clec3b(nc oe) and knock down clec3b (sicon si185)/2024.3.14 QBC 939 clec3b/clec3b nc oe 第4张]

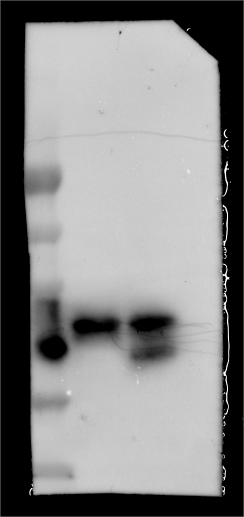

Supplement: Supplemental Information 4 — Western blot original strip, quantitative gray value and statistical map. [file peerj-12-18497-s004.zip › In all Figure , all the original western blot images, original gray value data and statistical graphs were obtained(In addition to overexpression and knock-down validation bands)/qbc939/qbc939 over expression clec3b(nc oe) and knock down clec3b (sicon si185)/2024.3.16 QBC939 clec3b nc oe sicon si185]

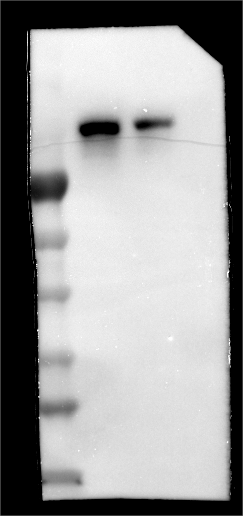

Supplement: Supplemental Information 4 — Western blot original strip, quantitative gray value and statistical map. [file peerj-12-18497-s004.zip › In all Figure , all the original western blot images, original gray value data and statistical graphs were obtained(In addition to overexpression and knock-down validation bands)/qbc939/qbc939 over expression clec3b(nc oe) and knock down clec3b (sicon si185)/2024.3.16 QBC939 clec3b nc oe sicon si185]

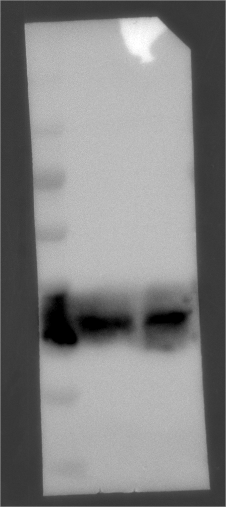

Supplement: Supplemental Information 4 — Western blot original strip, quantitative gray value and statistical map. [file peerj-12-18497-s004.zip › In all Figure , all the original western blot images, original gray value data and statistical graphs were obtained(In addition to overexpression and knock-down validation bands)/qbc939/qbc939 over expression clec3b(nc oe) and knock down clec3b (sicon si185)/2024.3.16 QBC939 clec3b nc oe sicon si185]

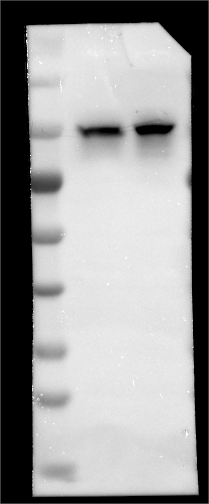

Supplement: Supplemental Information 4 — Western blot original strip, quantitative gray value and statistical map. [file peerj-12-18497-s004.zip › In all Figure , all the original western blot images, original gray value data and statistical graphs were obtained(In addition to overexpression and knock-down validation bands)/qbc939/qbc939 over expression clec3b(nc oe) and knock down clec3b (sicon si185)/2024.3.16 QBC939 clec3b nc oe sicon si185]

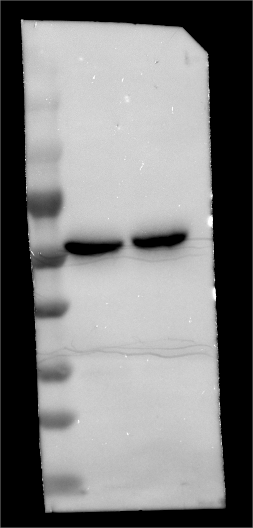

Supplement: Supplemental Information 4 — Western blot original strip, quantitative gray value and statistical map. [file peerj-12-18497-s004.zip › In all Figure , all the original western blot images, original gray value data and statistical graphs were obtained(In addition to overexpression and knock-down validation bands)/qbc939/qbc939 over expression clec3b(nc oe) and knock down clec3b (sicon si185)/2024.3.16 QBC939 clec3b nc oe sicon si185]

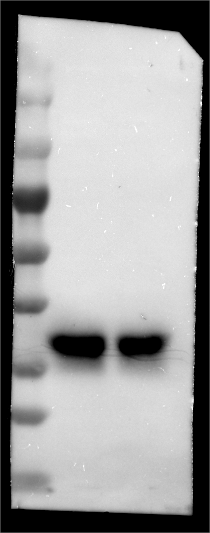

Supplement: Supplemental Information 4 — Western blot original strip, quantitative gray value and statistical map. [file peerj-12-18497-s004.zip › In all Figure , all the original western blot images, original gray value data and statistical graphs were obtained(In addition to overexpression and knock-down validation bands)/qbc939/qbc939 over expression clec3b(nc oe) and knock down clec3b (sicon si185)/2024.3.16 QBC939 clec3b nc oe sicon si185]

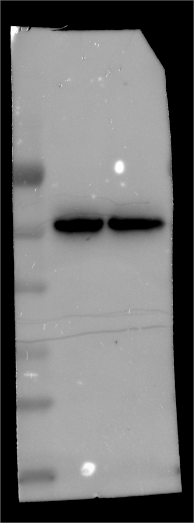

Supplement: Supplemental Information 4 — Western blot original strip, quantitative gray value and statistical map. [file peerj-12-18497-s004.zip › In all Figure , all the original western blot images, original gray value data and statistical graphs were obtained(In addition to overexpression and knock-down validation bands)/qbc939/qbc939 over expression clec3b(nc oe) and knock down clec3b (sicon si185)/2024.3.16 QBC939 clec3b nc oe sicon si185]

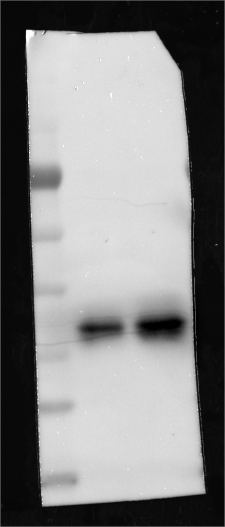

Supplement: Supplemental Information 4 — Western blot original strip, quantitative gray value and statistical map. [file peerj-12-18497-s004.zip › In all Figure , all the original western blot images, original gray value data and statistical graphs were obtained(In addition to overexpression and knock-down validation bands)/qbc939/qbc939 over expression clec3b(nc oe) and knock down clec3b (sicon si185)/2024.3.16 QBC939 clec3b nc oe sicon si185]

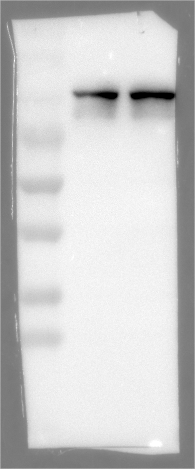

Supplement: Supplemental Information 4 — Western blot original strip, quantitative gray value and statistical map. [file peerj-12-18497-s004.zip › In all Figure , all the original western blot images, original gray value data and statistical graphs were obtained(In addition to overexpression and knock-down validation bands)/qbc939/qbc939 over expression clec3b(nc oe) and knock down clec3b (sicon si185)/2024.3.16 QBC939 clec3b nc oe sicon si185]

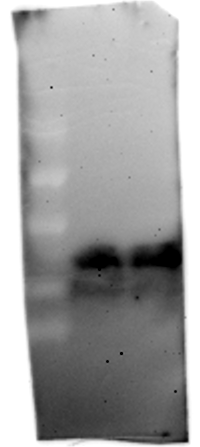

Supplement: Supplemental Information 4 — Western blot original strip, quantitative gray value and statistical map. [file peerj-12-18497-s004.zip › In all Figure , all the original western blot images, original gray value data and statistical graphs were obtained(In addition to overexpression and knock-down validation bands)/qbc939/qbc939 over expression clec3b(nc oe) and knock down clec3b (sicon si185)/2024.3.16 QBC939 clec3b nc oe sicon si185]

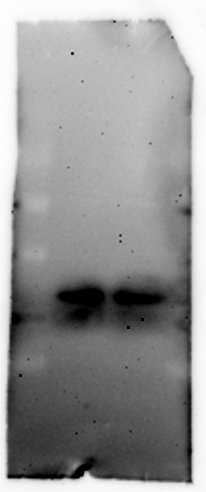

Supplement: Supplemental Information 4 — Western blot original strip, quantitative gray value and statistical map. [file peerj-12-18497-s004.zip › In all Figure , all the original western blot images, original gray value data and statistical graphs were obtained(In addition to overexpression and knock-down validation bands)/qbc939/qbc939 over expression clec3b(nc oe) and knock down clec3b (sicon si185)/2024.3.16 QBC939 clec3b nc oe sicon si185]

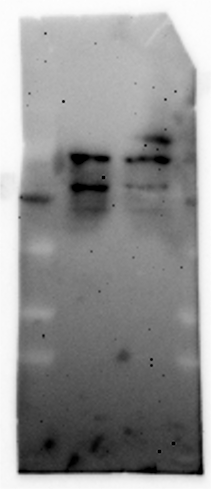

Supplement: Supplemental Information 4 — Western blot original strip, quantitative gray value and statistical map. [file peerj-12-18497-s004.zip › In all Figure , all the original western blot images, original gray value data and statistical graphs were obtained(In addition to overexpression and knock-down validation bands)/qbc939/qbc939 over expression clec3b(nc oe) and knock down clec3b (sicon si185)/2024.3.16 QBC939 clec3b nc oe sicon si185]

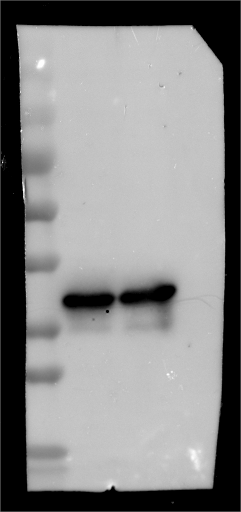

Supplement: Supplemental Information 4 — Western blot original strip, quantitative gray value and statistical map. [file peerj-12-18497-s004.zip › In all Figure , all the original western blot images, original gray value data and statistical graphs were obtained(In addition to overexpression and knock-down validation bands)/qbc939/qbc939 over expression clec3b(nc oe) and knock down clec3b (sicon si185)/2024.3.19 qbc939 clec3b nc oe sicon si185]

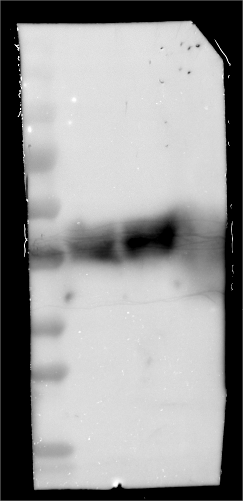

Supplement: Supplemental Information 4 — Western blot original strip, quantitative gray value and statistical map. [file peerj-12-18497-s004.zip › In all Figure , all the original western blot images, original gray value data and statistical graphs were obtained(In addition to overexpression and knock-down validation bands)/qbc939/qbc939 over expression clec3b(nc oe) and knock down clec3b (sicon si185)/2024.3.19 qbc939 clec3b nc oe sicon si185]

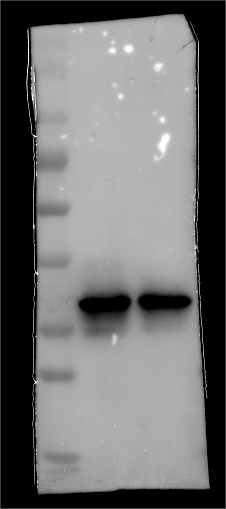

Supplement: Supplemental Information 4 — Western blot original strip, quantitative gray value and statistical map. [file peerj-12-18497-s004.zip › In all Figure , all the original western blot images, original gray value data and statistical graphs were obtained(In addition to overexpression and knock-down validation bands)/qbc939/qbc939 over expression clec3b(nc oe) and knock down clec3b (sicon si185)/2024.3.19 qbc939 clec3b nc oe sicon si185]

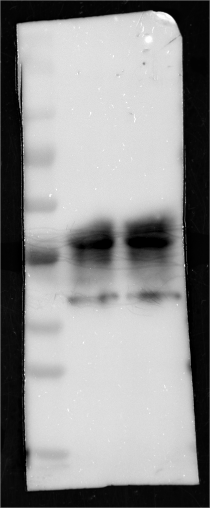

Supplement: Supplemental Information 4 — Western blot original strip, quantitative gray value and statistical map. [file peerj-12-18497-s004.zip › In all Figure , all the original western blot images, original gray value data and statistical graphs were obtained(In addition to overexpression and knock-down validation bands)/qbc939/qbc939 over expression clec3b(nc oe) and knock down clec3b (sicon si185)/2024.3.19 qbc939 clec3b nc oe sicon si185]

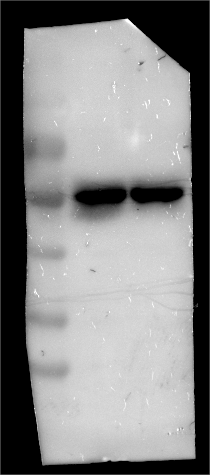

Supplement: Supplemental Information 4 — Western blot original strip, quantitative gray value and statistical map. [file peerj-12-18497-s004.zip › In all Figure , all the original western blot images, original gray value data and statistical graphs were obtained(In addition to overexpression and knock-down validation bands)/qbc939/qbc939 over expression clec3b(nc oe) and knock down clec3b (sicon si185)/2024.3.22 qbc939 CLEC3B NC OE SICON SI185]

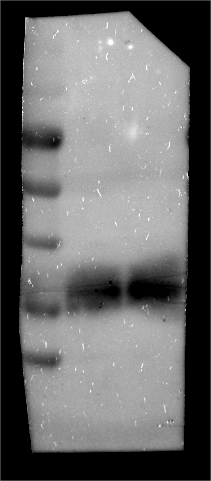

Supplement: Supplemental Information 4 — Western blot original strip, quantitative gray value and statistical map. [file peerj-12-18497-s004.zip › In all Figure , all the original western blot images, original gray value data and statistical graphs were obtained(In addition to overexpression and knock-down validation bands)/qbc939/qbc939 over expression clec3b(nc oe) and knock down clec3b (sicon si185)/2024.3.22 qbc939 CLEC3B NC OE SICON SI185]

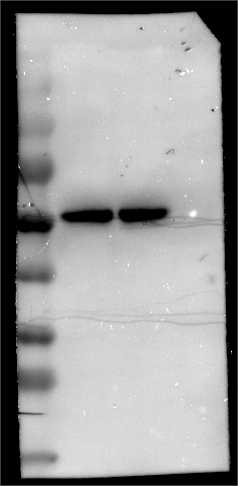

Supplement: Supplemental Information 4 — Western blot original strip, quantitative gray value and statistical map. [file peerj-12-18497-s004.zip › In all Figure , all the original western blot images, original gray value data and statistical graphs were obtained(In addition to overexpression and knock-down validation bands)/qbc939/qbc939 over expression clec3b(nc oe) and knock down clec3b (sicon si185)/2024.3.22 qbc939 CLEC3B NC OE SICON SI185]

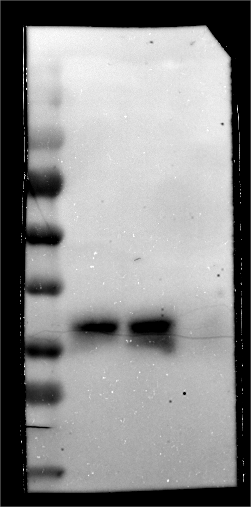

Supplement: Supplemental Information 4 — Western blot original strip, quantitative gray value and statistical map. [file peerj-12-18497-s004.zip › In all Figure , all the original western blot images, original gray value data and statistical graphs were obtained(In addition to overexpression and knock-down validation bands)/qbc939/qbc939 over expression clec3b(nc oe) and knock down clec3b (sicon si185)/2024.3.22 qbc939 CLEC3B NC OE SICON SI185]

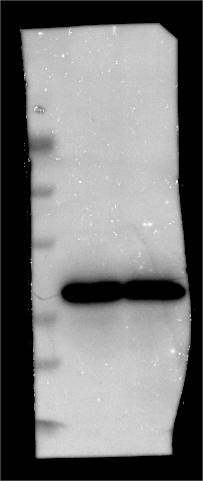

Supplement: Supplemental Information 4 — Western blot original strip, quantitative gray value and statistical map. [file peerj-12-18497-s004.zip › In all Figure , all the original western blot images, original gray value data and statistical graphs were obtained(In addition to overexpression and knock-down validation bands)/qbc939/qbc939 over expression clec3b(nc oe) and knock down clec3b (sicon si185)/2024.3.23 qbc939 clec3b nc oe sicon si185]

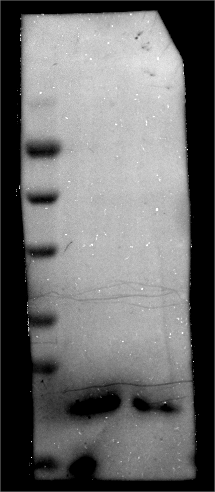

Supplement: Supplemental Information 4 — Western blot original strip, quantitative gray value and statistical map. [file peerj-12-18497-s004.zip › In all Figure , all the original western blot images, original gray value data and statistical graphs were obtained(In addition to overexpression and knock-down validation bands)/qbc939/qbc939 over expression clec3b(nc oe) and knock down clec3b (sicon si185)/2024.3.23 qbc939 clec3b nc oe sicon si185]

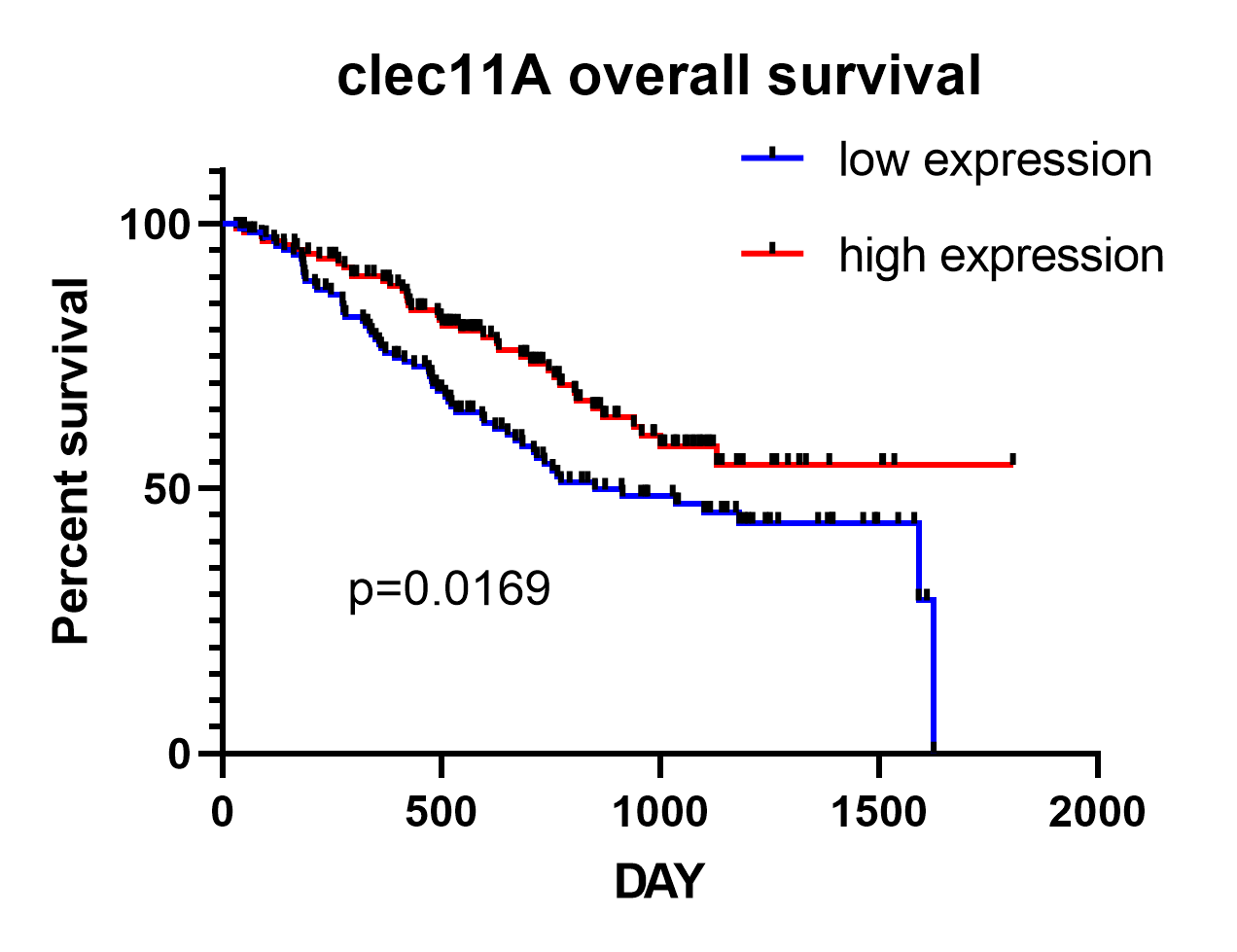

Supplement: Supplemental Information 6 [file peerj-12-18497-s006.zip › Raw data from survival analysis of eight genes of the C-type lectin family/生存分析tif/CLEC11A.tif]

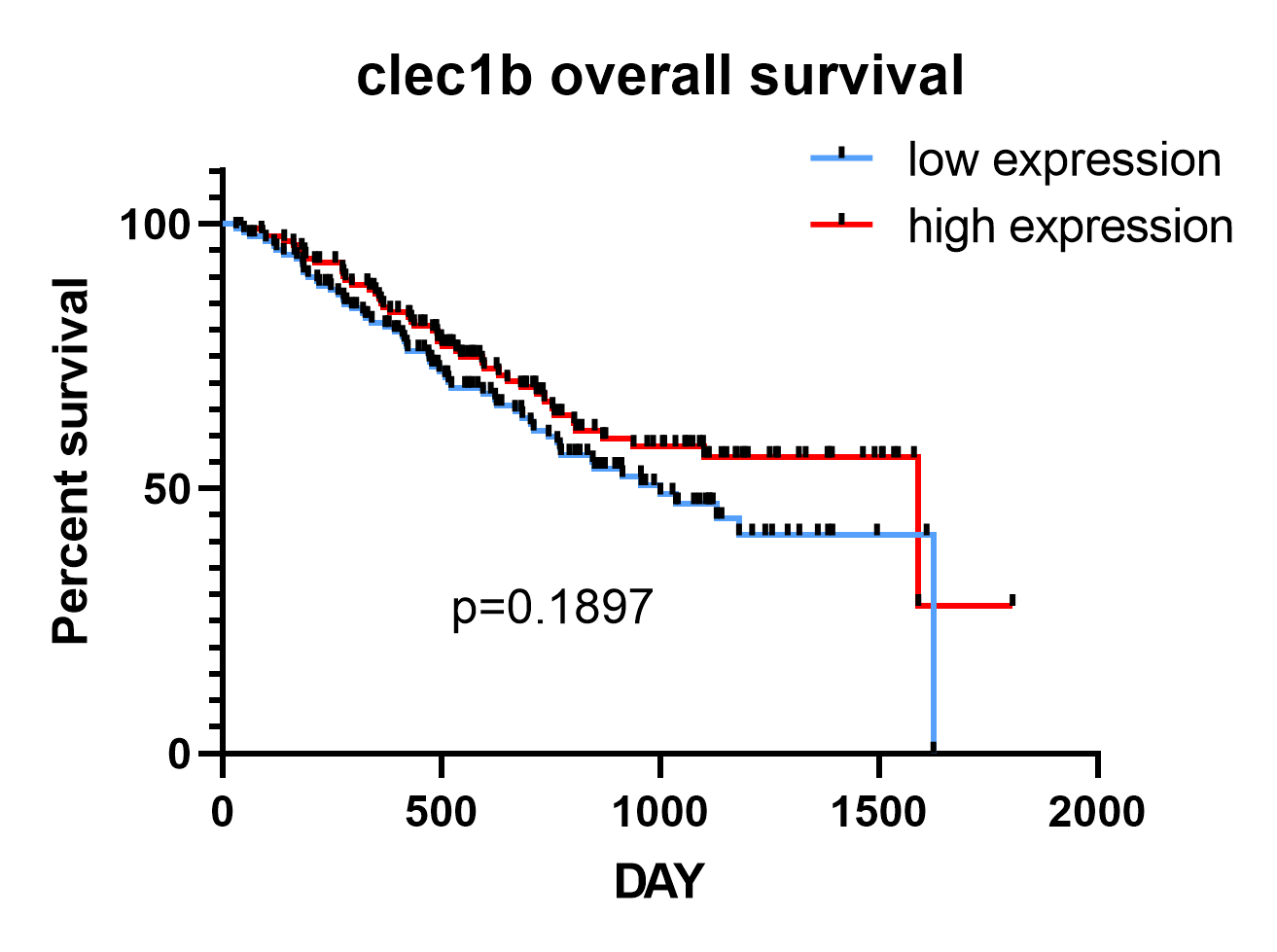

Supplement: Supplemental Information 6 [file peerj-12-18497-s006.zip › Raw data from survival analysis of eight genes of the C-type lectin family/生存分析tif/clec1b.tif]
