# Supplementary figures and images for: The CLEC3B inhibits cellular proliferation and metastasis of cholangiocarcinoma through Wnt/β-catenin pathway (part 3 of 5)
Source: PeerJ. 2024 Nov 13;12:e18497. doi: 10.7717/peerj.18497 (PMC11568818; doi:10.7717/peerj.18497)

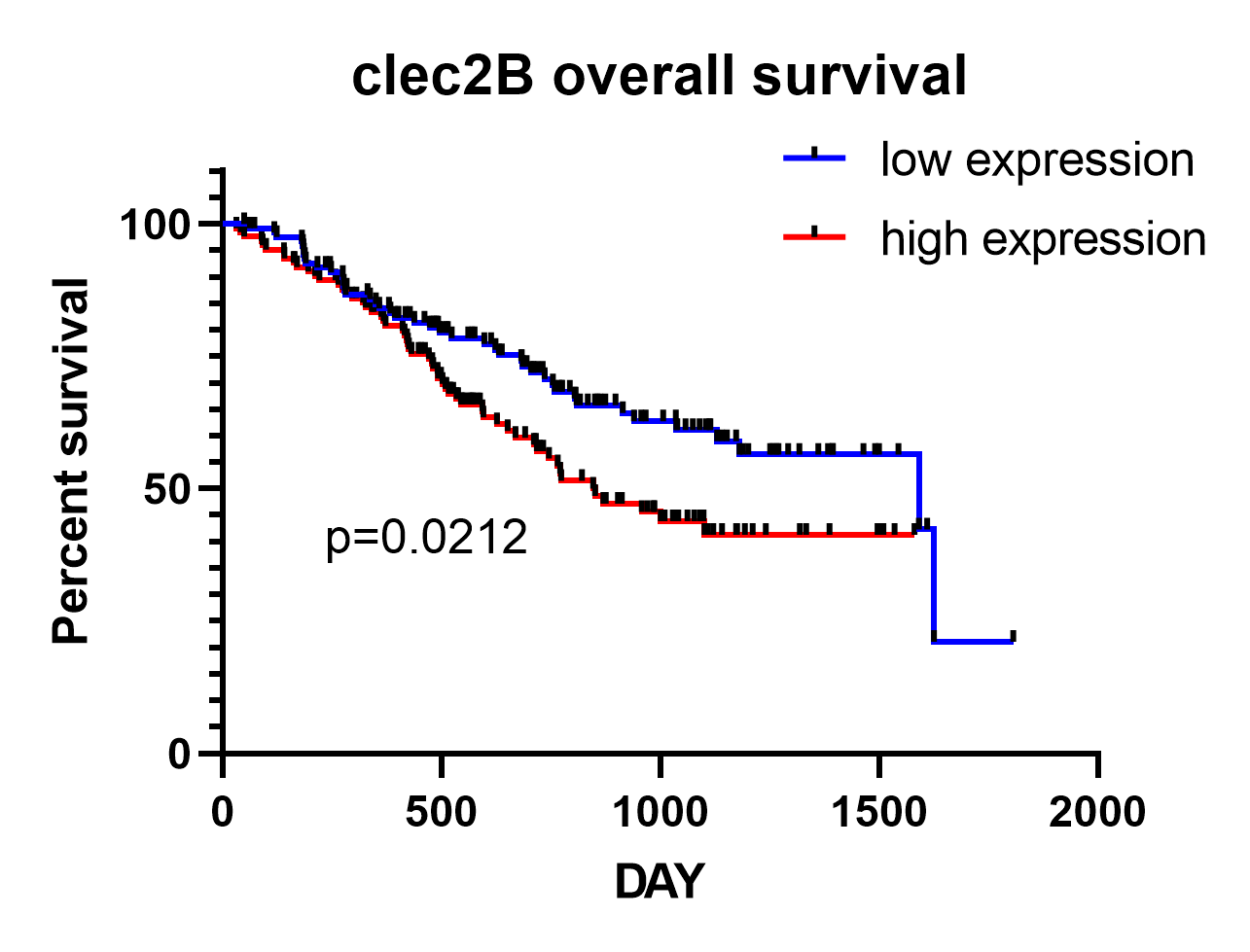

Supplement: Supplemental Information 6 [file peerj-12-18497-s006.zip › Raw data from survival analysis of eight genes of the C-type lectin family/生存分析tif/CLEC2B.tif]

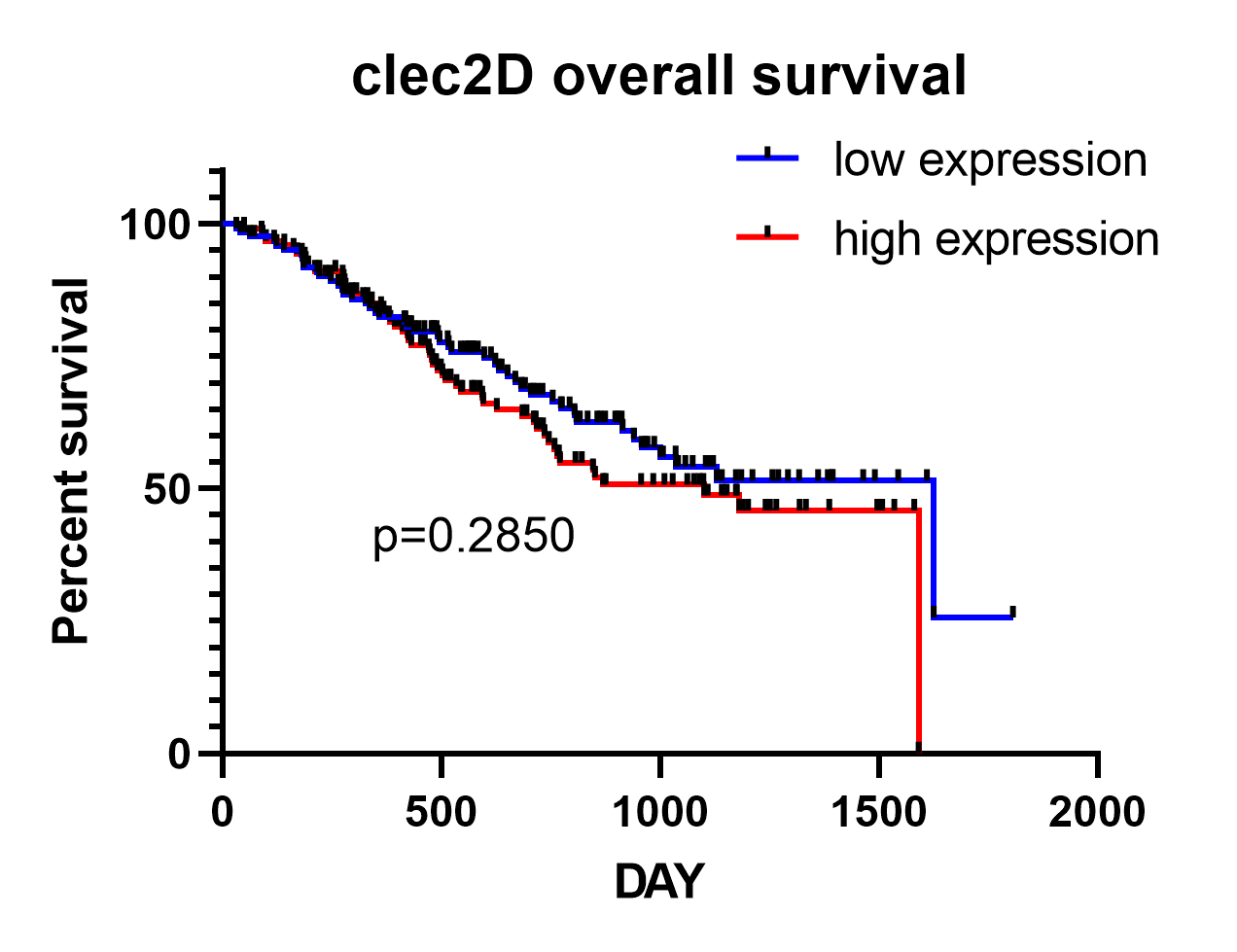

Supplement: Supplemental Information 6 [file peerj-12-18497-s006.zip › Raw data from survival analysis of eight genes of the C-type lectin family/生存分析tif/clec2d.tif]

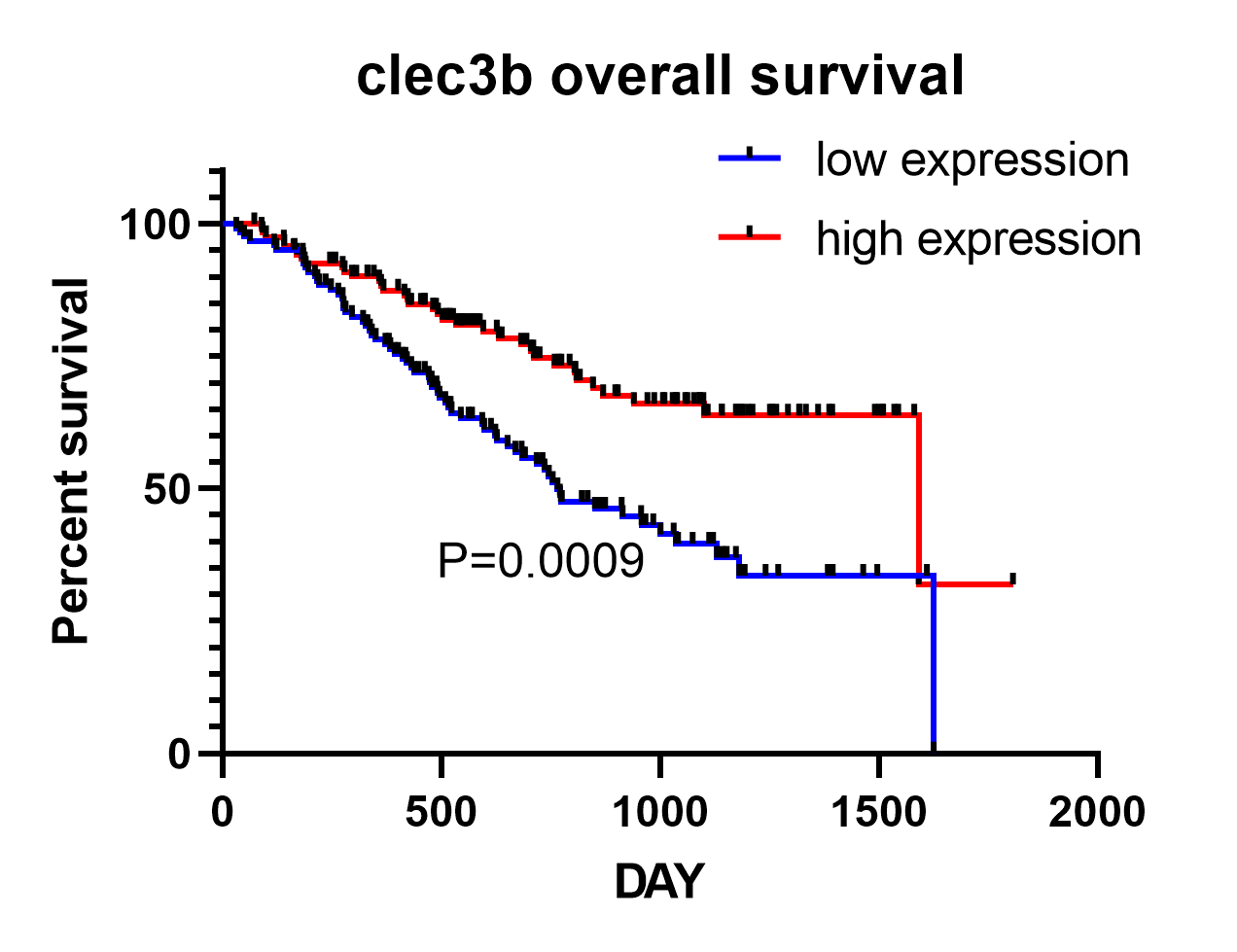

Supplement: Supplemental Information 6 [file peerj-12-18497-s006.zip › Raw data from survival analysis of eight genes of the C-type lectin family/生存分析tif/clec3b.tif]

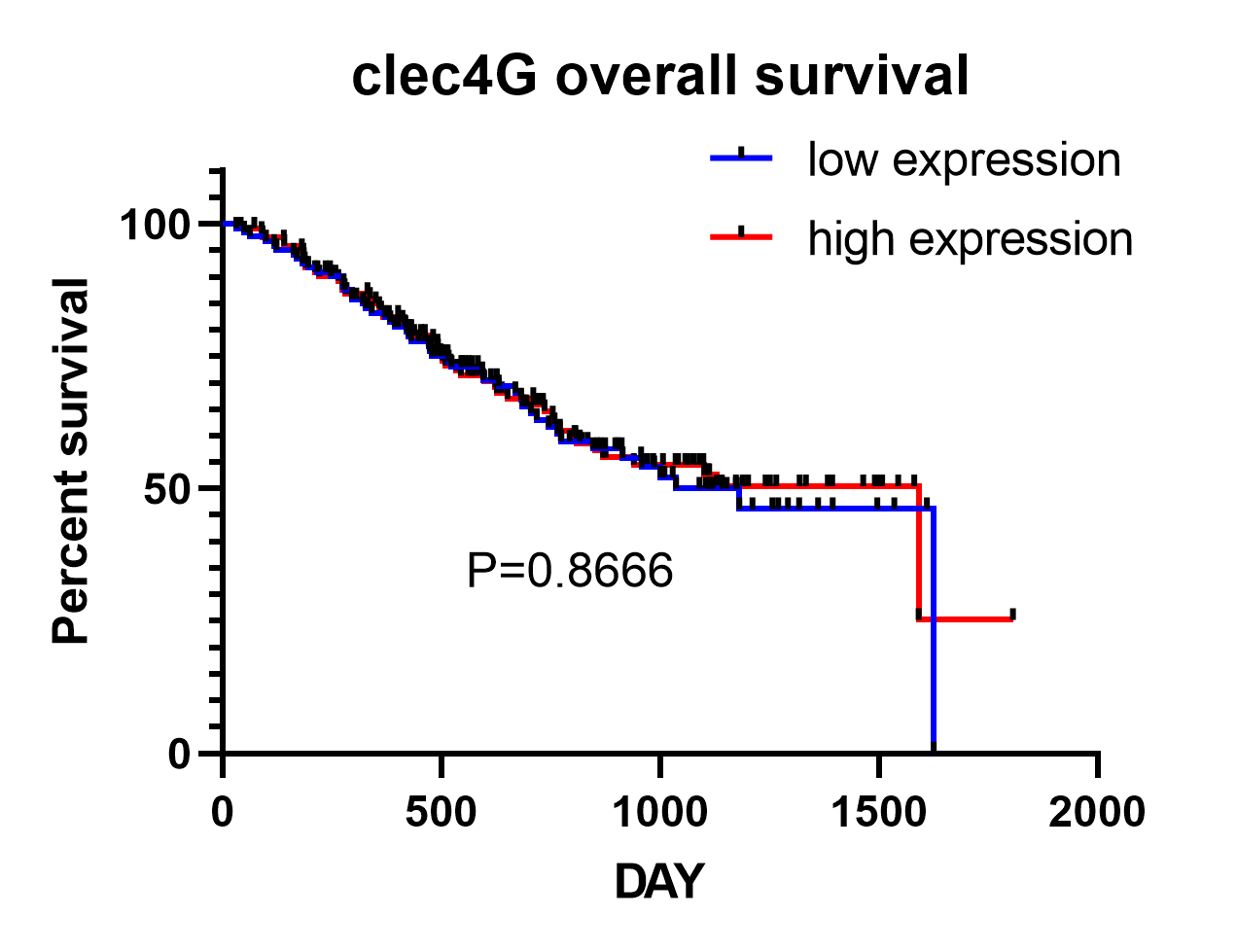

Supplement: Supplemental Information 6 [file peerj-12-18497-s006.zip › Raw data from survival analysis of eight genes of the C-type lectin family/生存分析tif/CLEC4G.tif]

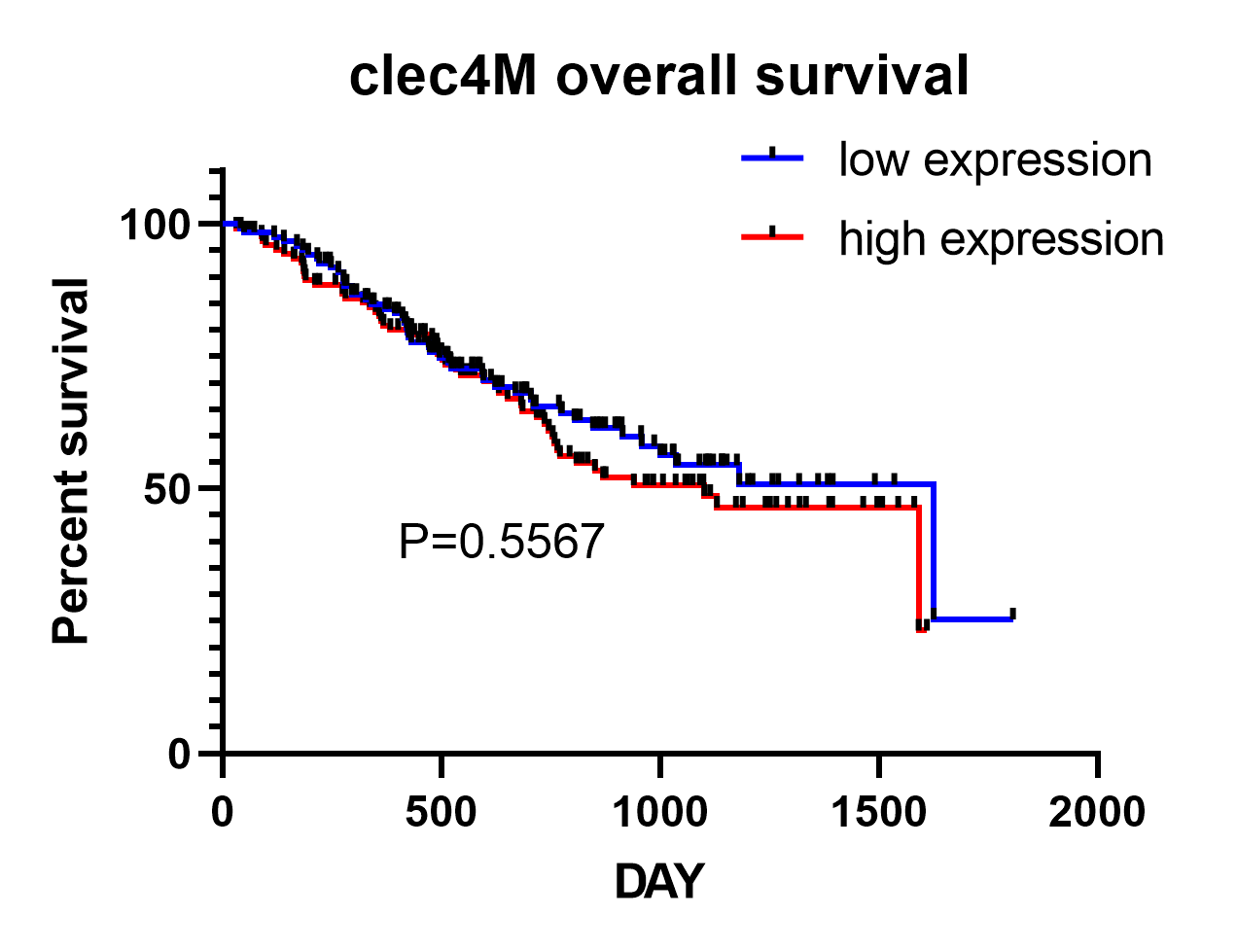

Supplement: Supplemental Information 6 [file peerj-12-18497-s006.zip › Raw data from survival analysis of eight genes of the C-type lectin family/生存分析tif/CLEC4M.tif]

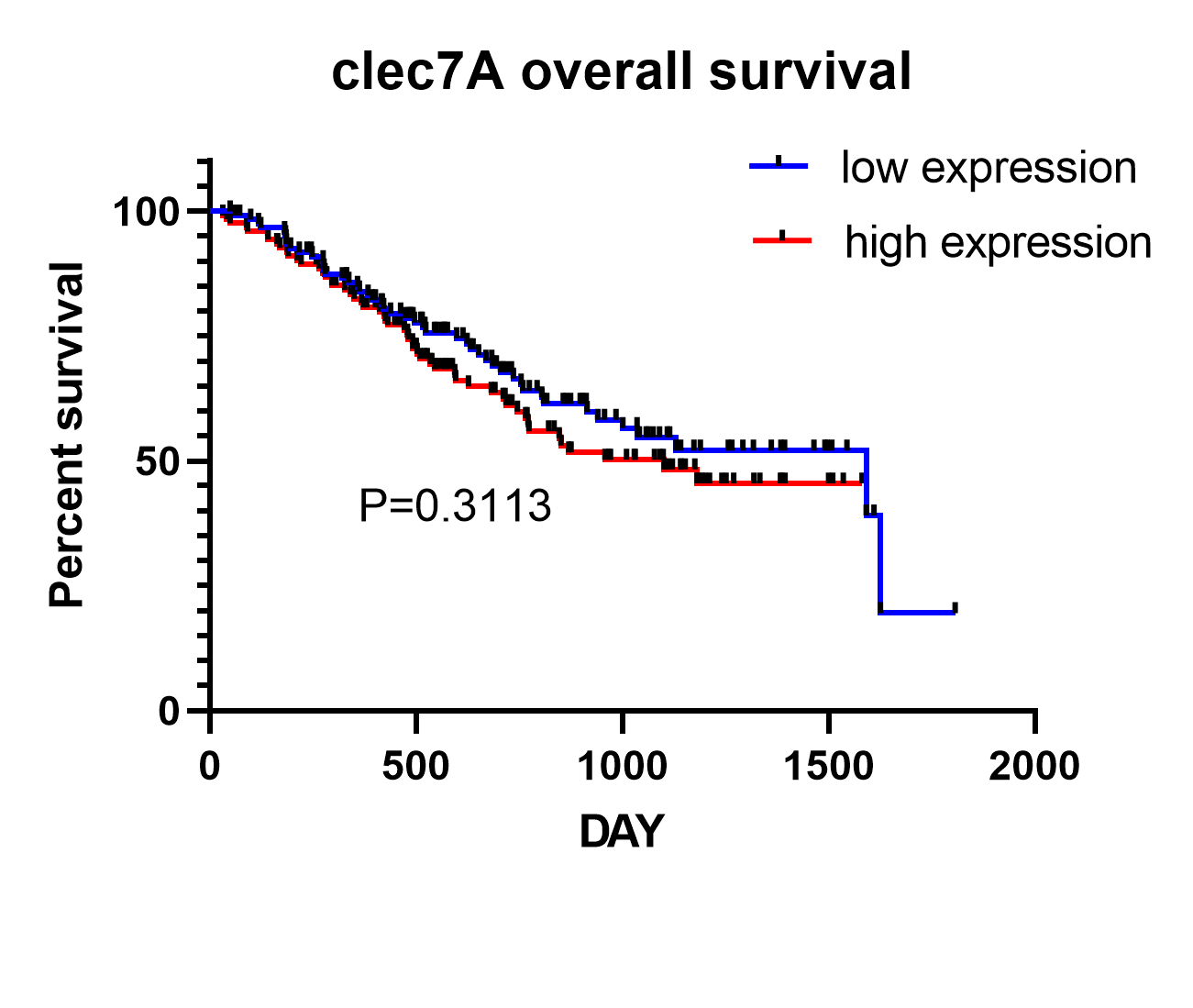

Supplement: Supplemental Information 6 [file peerj-12-18497-s006.zip › Raw data from survival analysis of eight genes of the C-type lectin family/生存分析tif/CLEC7A.tif]

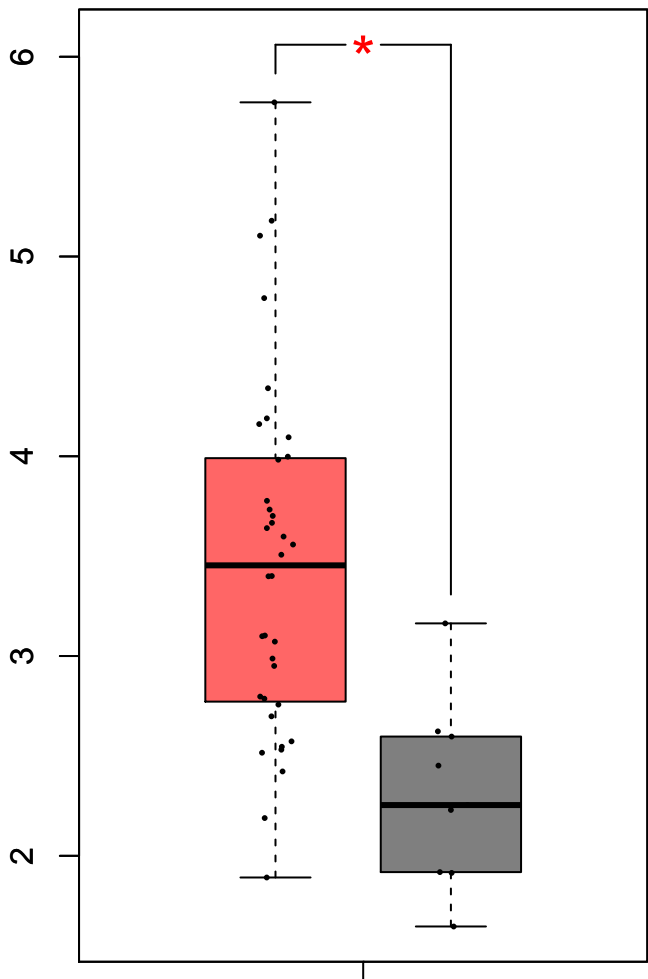

CHOL  
(num(T)=36; num(N)=9)

Supplement: Supplemental Information 7 [file peerj-12-18497-s007.zip › TCGA database analysis clec family/CLEC11A_boxplot_cG3sb.pdf]

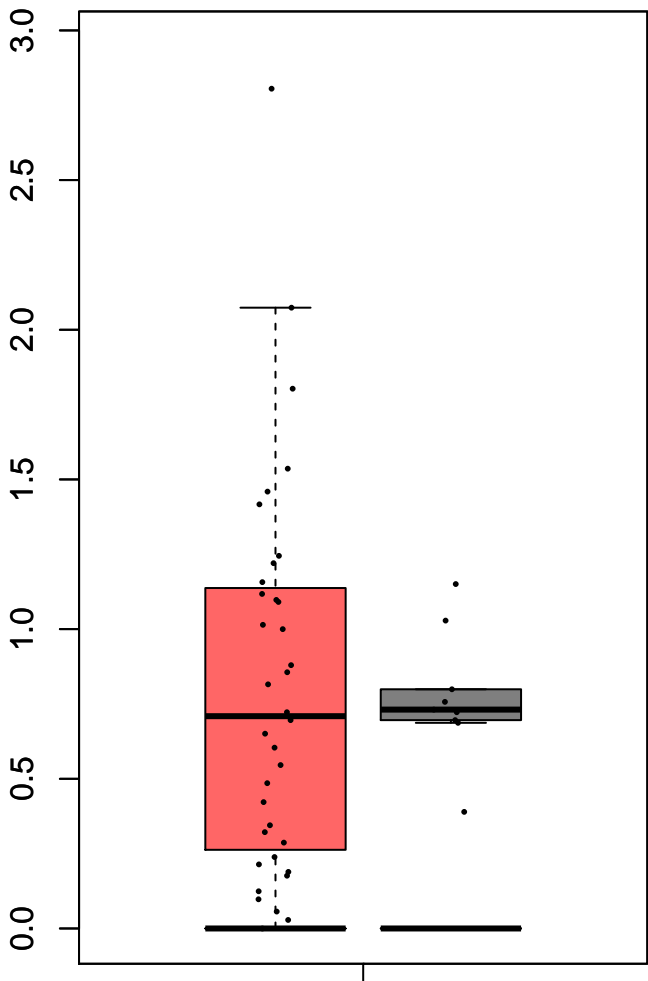

CHOL  
(num(T)=36; num(N)=9)

Supplement: Supplemental Information 7 [file peerj-12-18497-s007.zip › TCGA database analysis clec family/CLEC12A_boxplot_QTyC5.pdf]

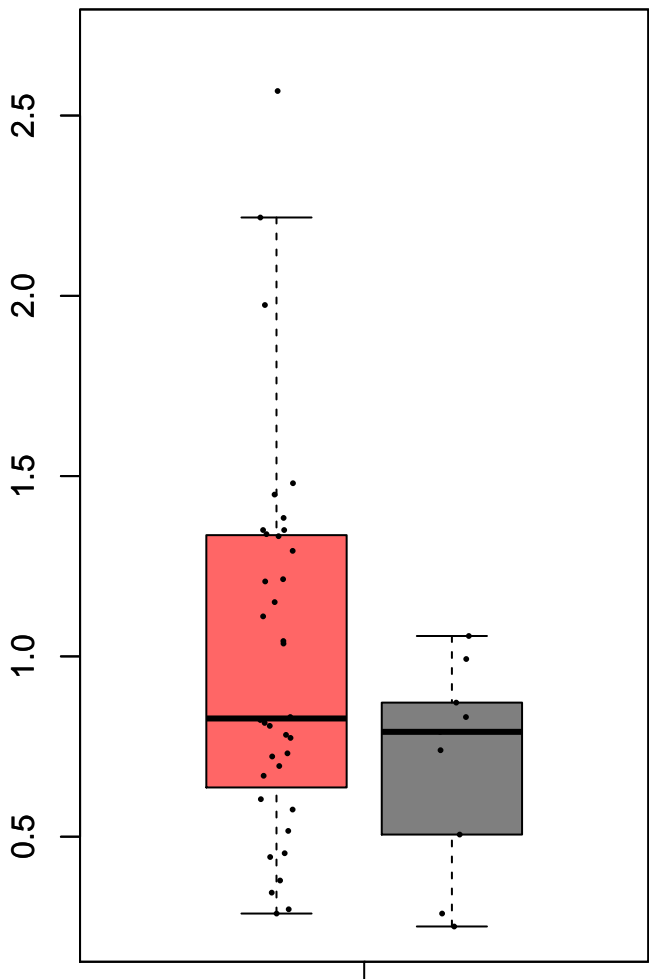

CHOL  
(num(T)=36; num(N)=9)

Supplement: Supplemental Information 7 [file peerj-12-18497-s007.zip › TCGA database analysis clec family/CLEC1A_boxplot_m8b4R.pdf]

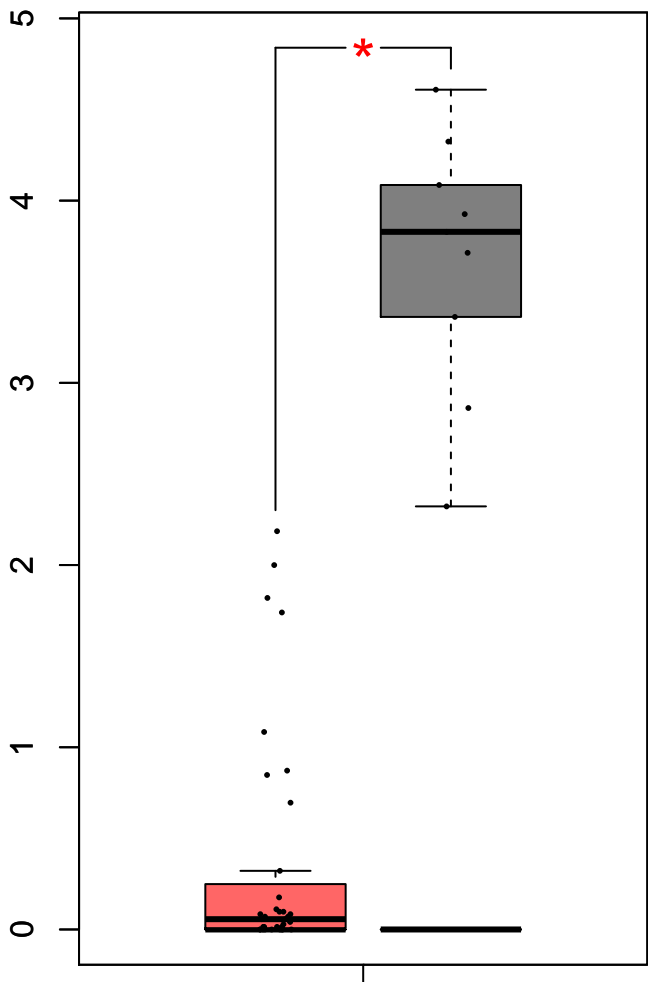

CHOL  
(num(T)=36; num(N)=9)

Supplement: Supplemental Information 7 [file peerj-12-18497-s007.zip › TCGA database analysis clec family/CLEC1B_boxplot_VUFhm.pdf]

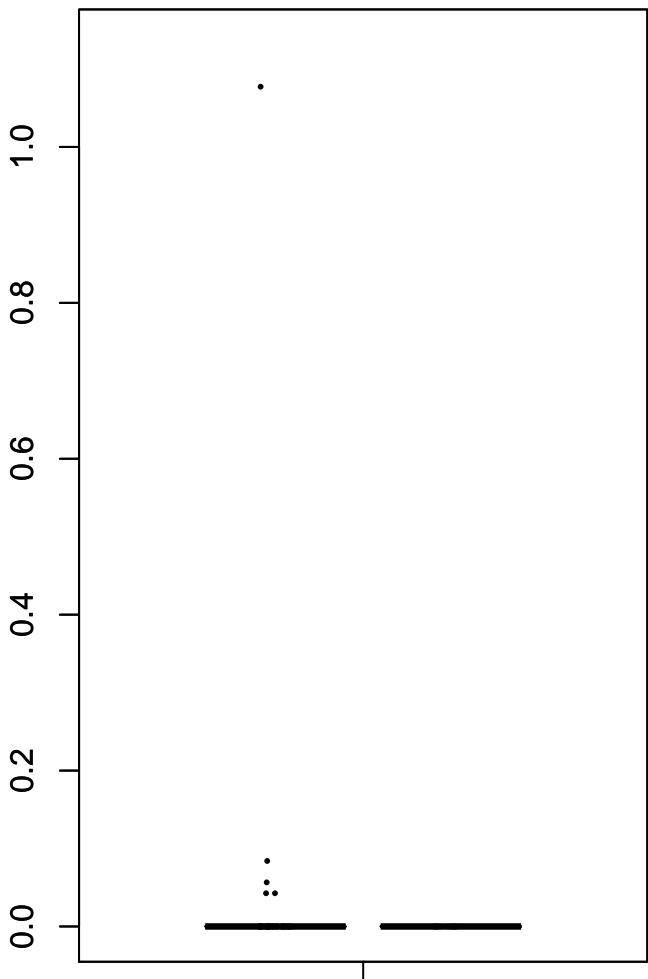

CHOL  
(num(T)=36; num(N)=9)

Supplement: Supplemental Information 7 [file peerj-12-18497-s007.zip › TCGA database analysis clec family/CLEC2A_boxplot_oq1Ss.pdf]

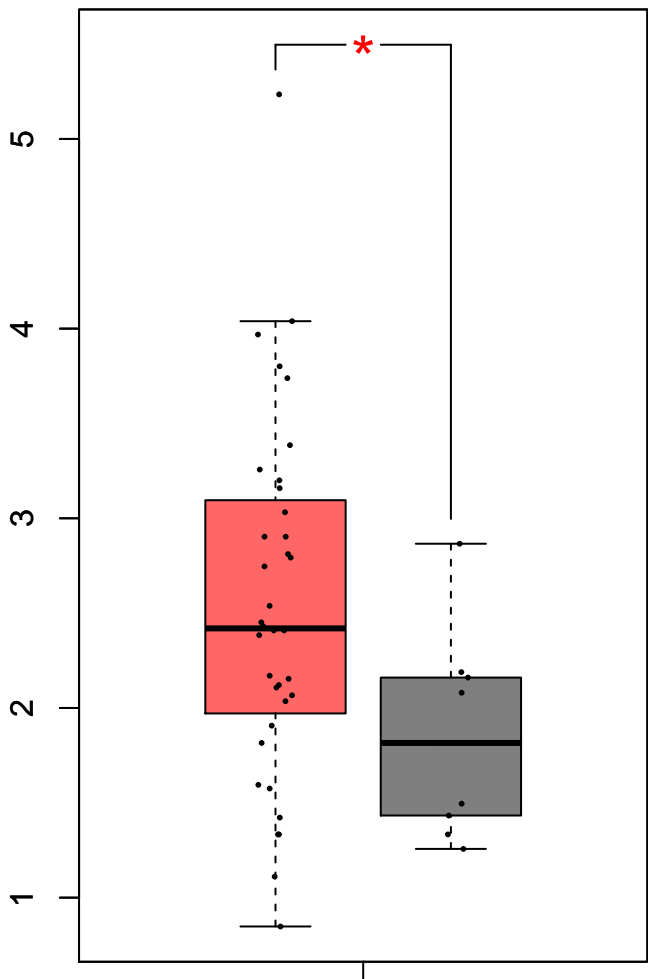

CHOL  
(num(T)=36; num(N)=9)

Supplement: Supplemental Information 7 [file peerj-12-18497-s007.zip › TCGA database analysis clec family/CLEC2B_boxplot_yHbjE.pdf]

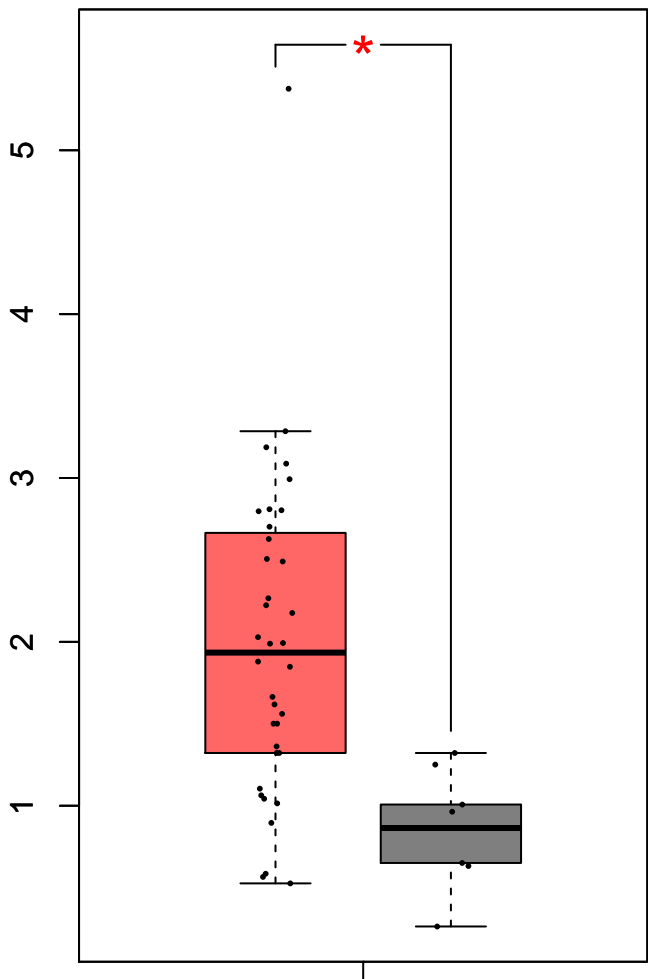

CHOL  
(num(T)=36; num(N)=9)

Supplement: Supplemental Information 7 [file peerj-12-18497-s007.zip › TCGA database analysis clec family/CLEC2D_boxplot_28NWS.pdf]

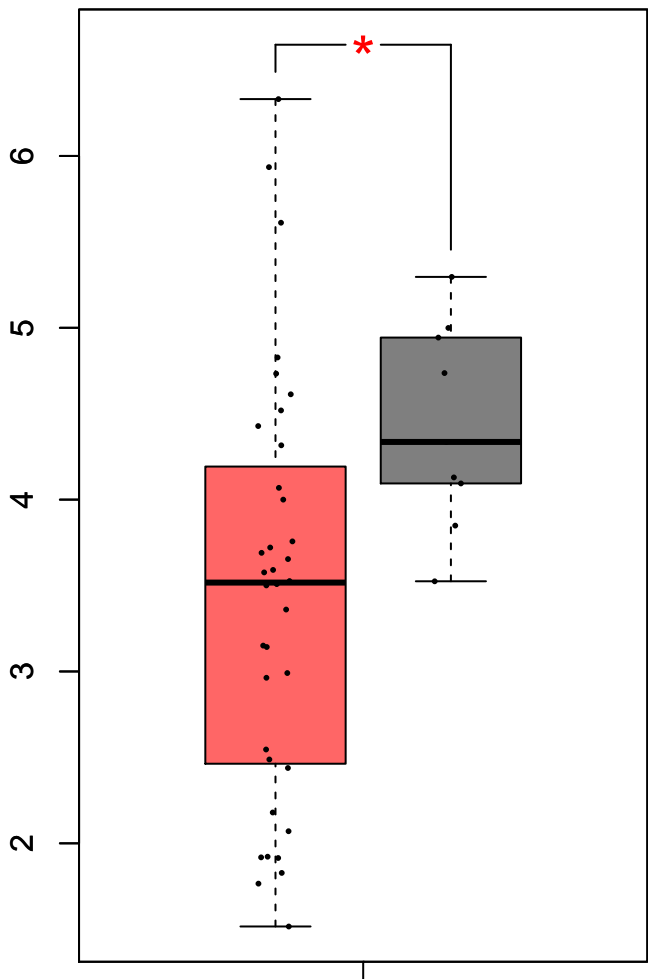

CHOL  
(num(T)=36; num(N)=9)

Supplement: Supplemental Information 7 [file peerj-12-18497-s007.zip › TCGA database analysis clec family/CLEC3B_boxplot_9QcpC.pdf]

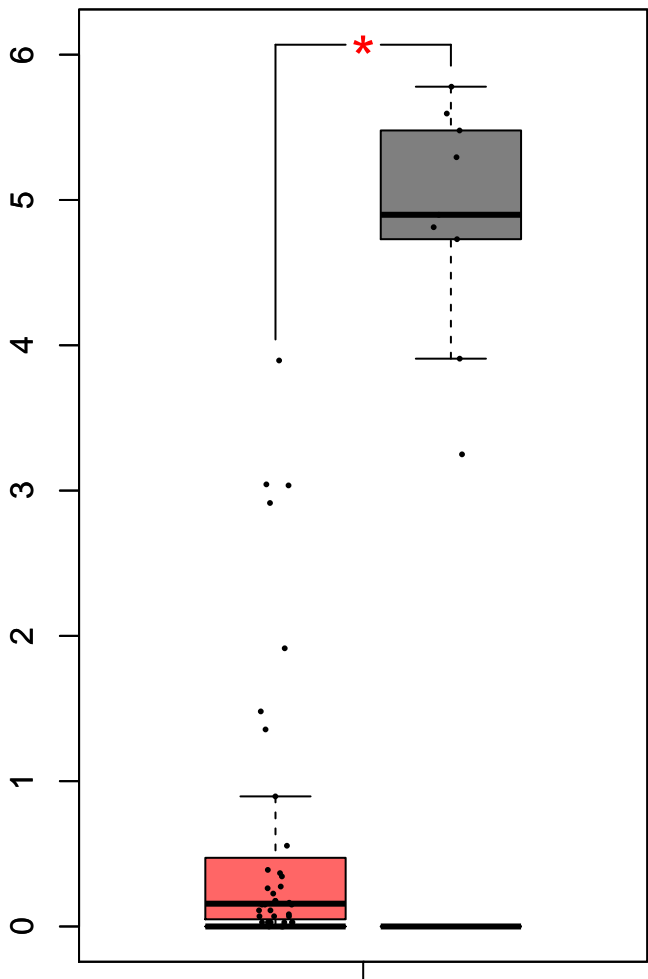

CHOL  
(num(T)=36; num(N)=9)

Supplement: Supplemental Information 7 [file peerj-12-18497-s007.zip › TCGA database analysis clec family/CLEC4G_boxplot_gt1Of.pdf]

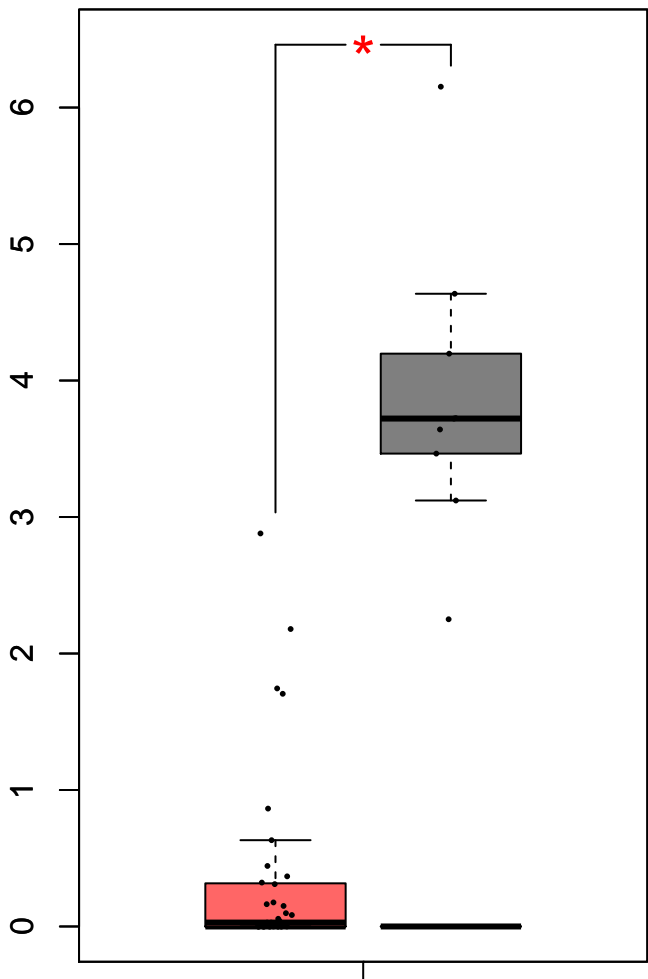

CHOL  
(num(T)=36; num(N)=9)

Supplement: Supplemental Information 7 [file peerj-12-18497-s007.zip › TCGA database analysis clec family/CLEC4M_boxplot_me7X8.pdf]

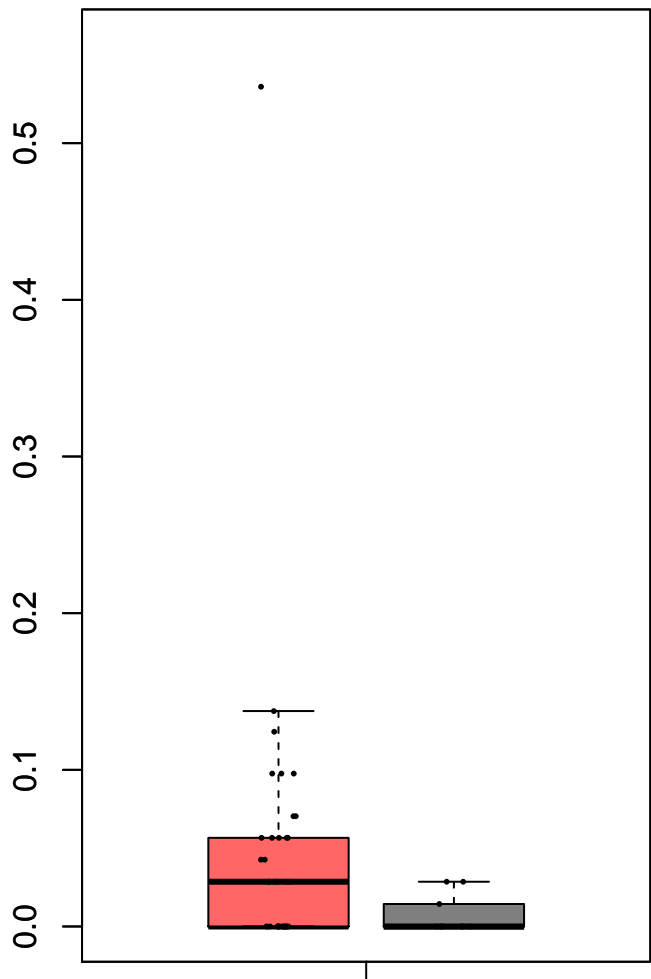

CHOL  
(num(T)=36; num(N)=9)

Supplement: Supplemental Information 7 [file peerj-12-18497-s007.zip › TCGA database analysis clec family/CLEC6A_boxplot_iffPz.pdf]

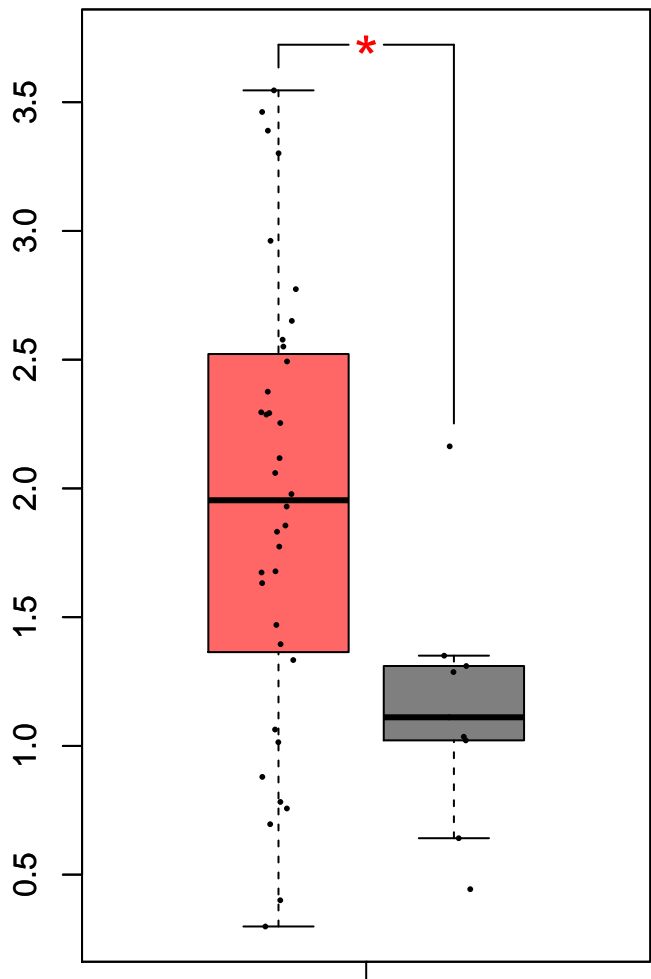

CHOL  
(num(T)=36; num(N)=9)

Supplement: Supplemental Information 7 [file peerj-12-18497-s007.zip › TCGA database analysis clec family/CLEC7A_boxplot_5zVxA.pdf]

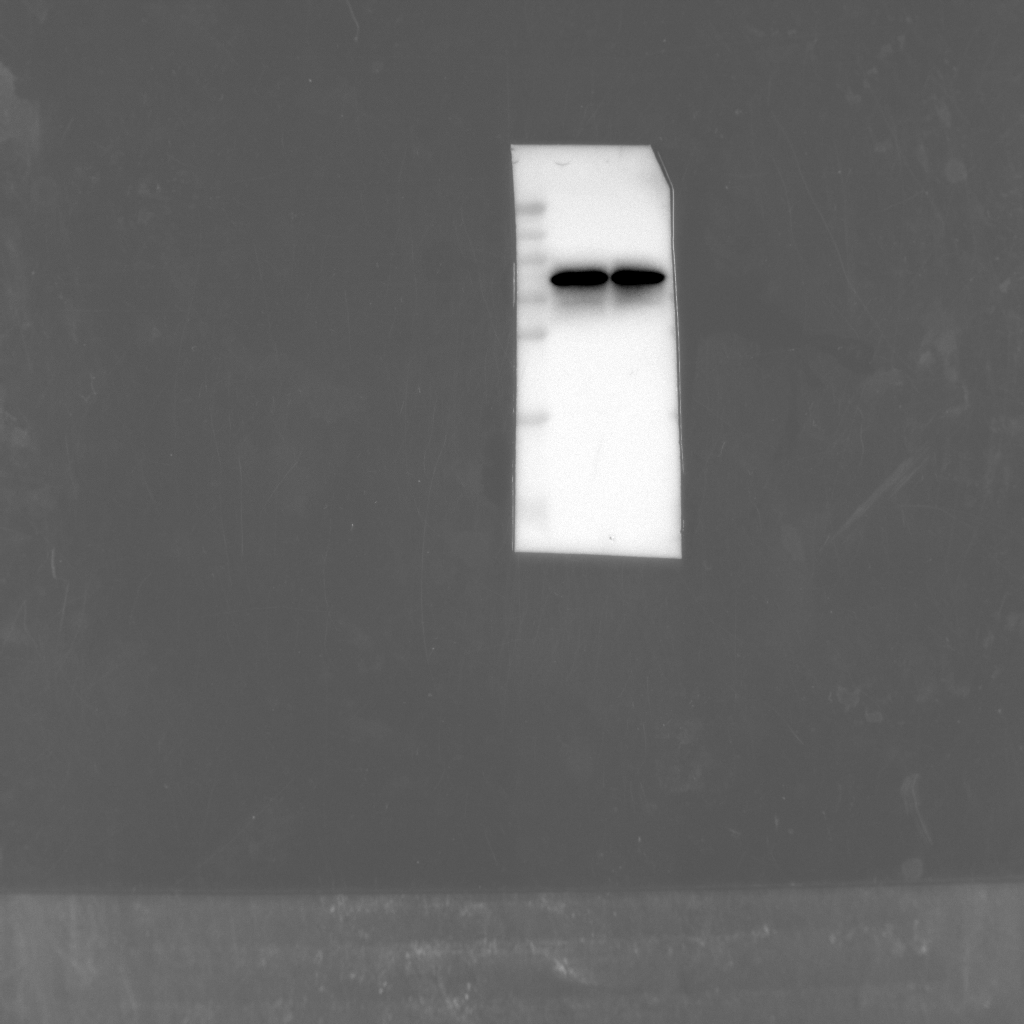

Supplement: Supplemental Information 8 — The original strips of western blot that validated the transfection efficiency of CLEC3B (Fig.2). [file peerj-12-18497-s008.zip › In Figure 2, overexpression of CLEC3B and knock-down of the original strip were verified by western blot/si/hucct/clec3b sicon si185 第一张 GAPDH-1.Tif]

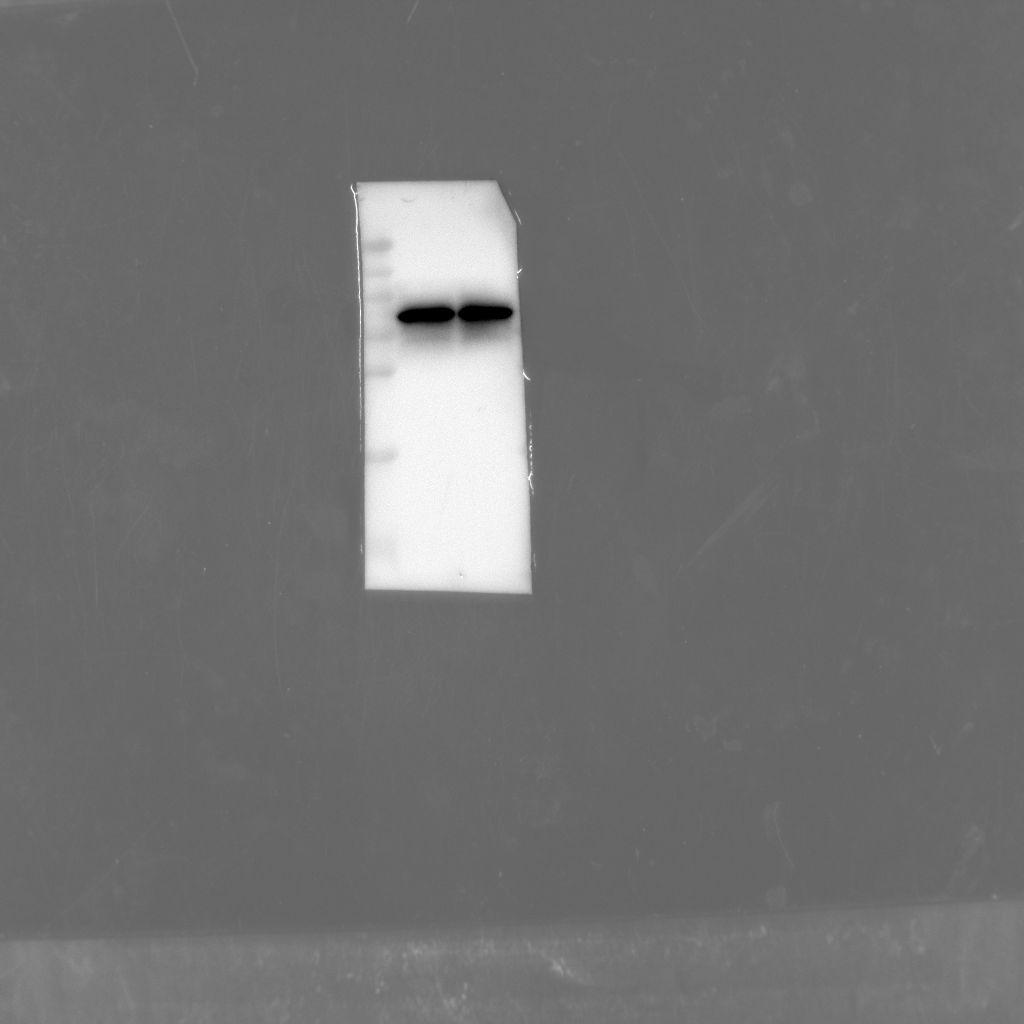

Supplement: Supplemental Information 8 — The original strips of western blot that validated the transfection efficiency of CLEC3B (Fig.2). [file peerj-12-18497-s008.zip › In Figure 2, overexpression of CLEC3B and knock-down of the original strip were verified by western blot/si/hucct/clec3b sicon si185 第一张 GAPDH-2.Tif]

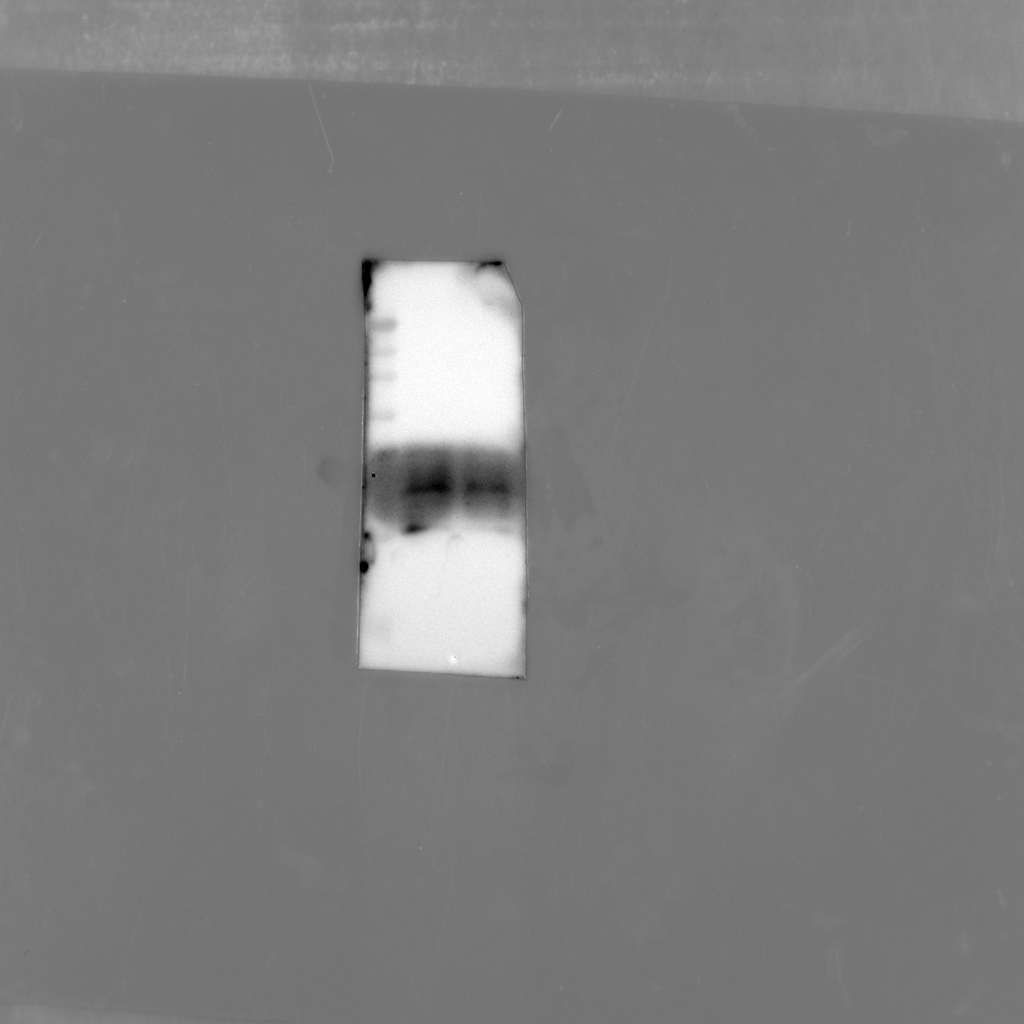

Supplement: Supplemental Information 8 — The original strips of western blot that validated the transfection efficiency of CLEC3B (Fig.2). [file peerj-12-18497-s008.zip › In Figure 2, overexpression of CLEC3B and knock-down of the original strip were verified by western blot/si/hucct/clec3b sicon si185 第一张-1.Tif]

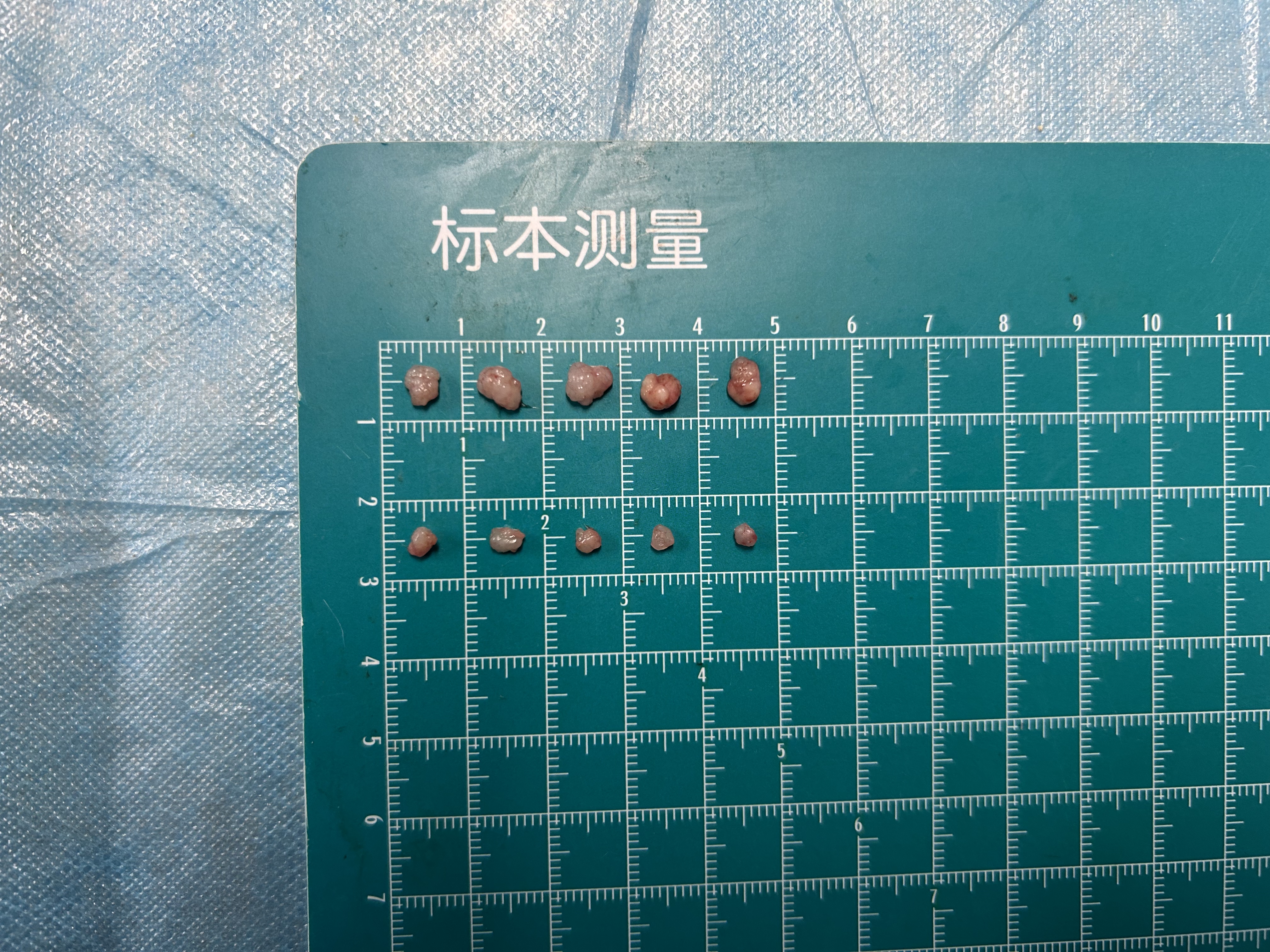

Supplement: Supplemental Information 9 — The raw images of tumor size in nude mice, western blot strips of tumor tissue, and corresponding statistical maps. [file peerj-12-18497-s009.zip › Raw tumor data of nude mice/tumor picture.jpg]

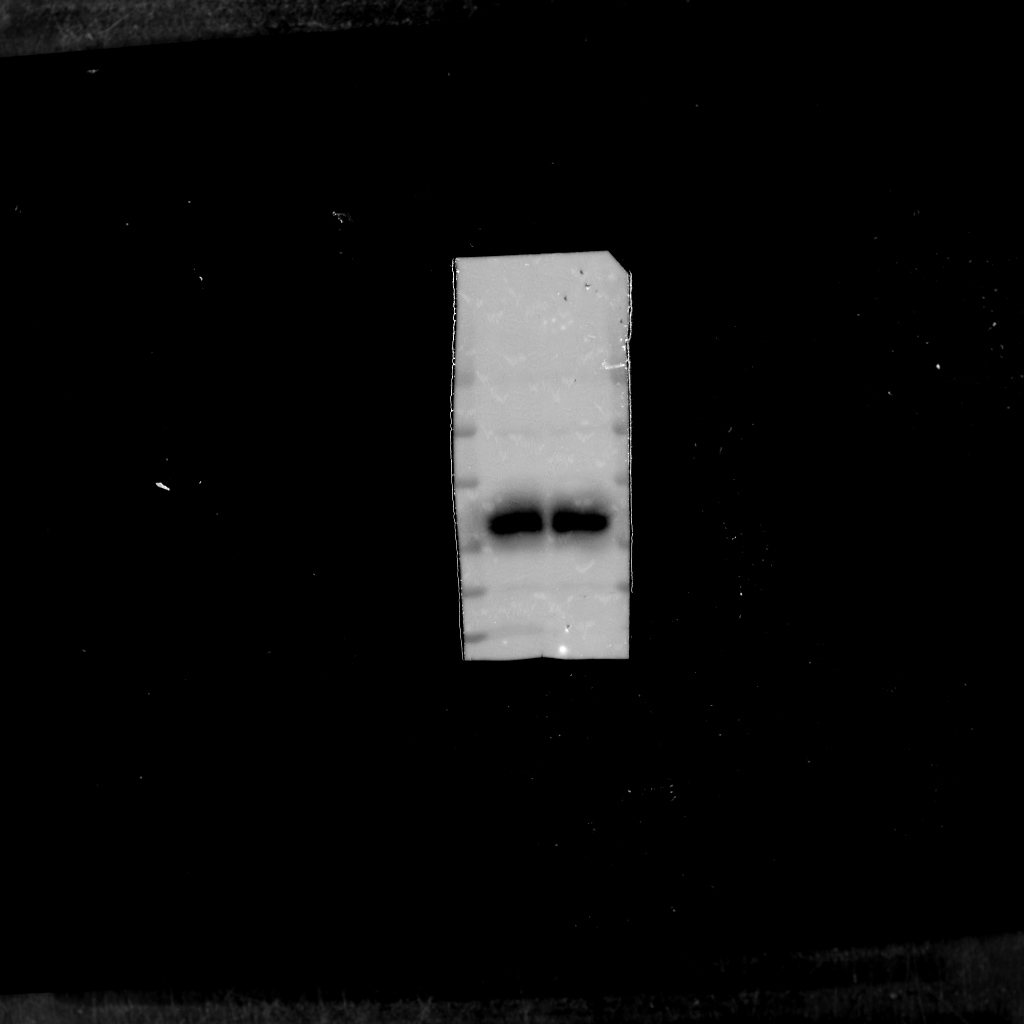

Supplement: Supplemental Information 9 — The raw images of tumor size in nude mice, western blot strips of tumor tissue, and corresponding statistical maps. [file peerj-12-18497-s009.zip › Raw tumor data of nude mice/western blot/未命名导出/bax 第1张 gapdh.jpg]

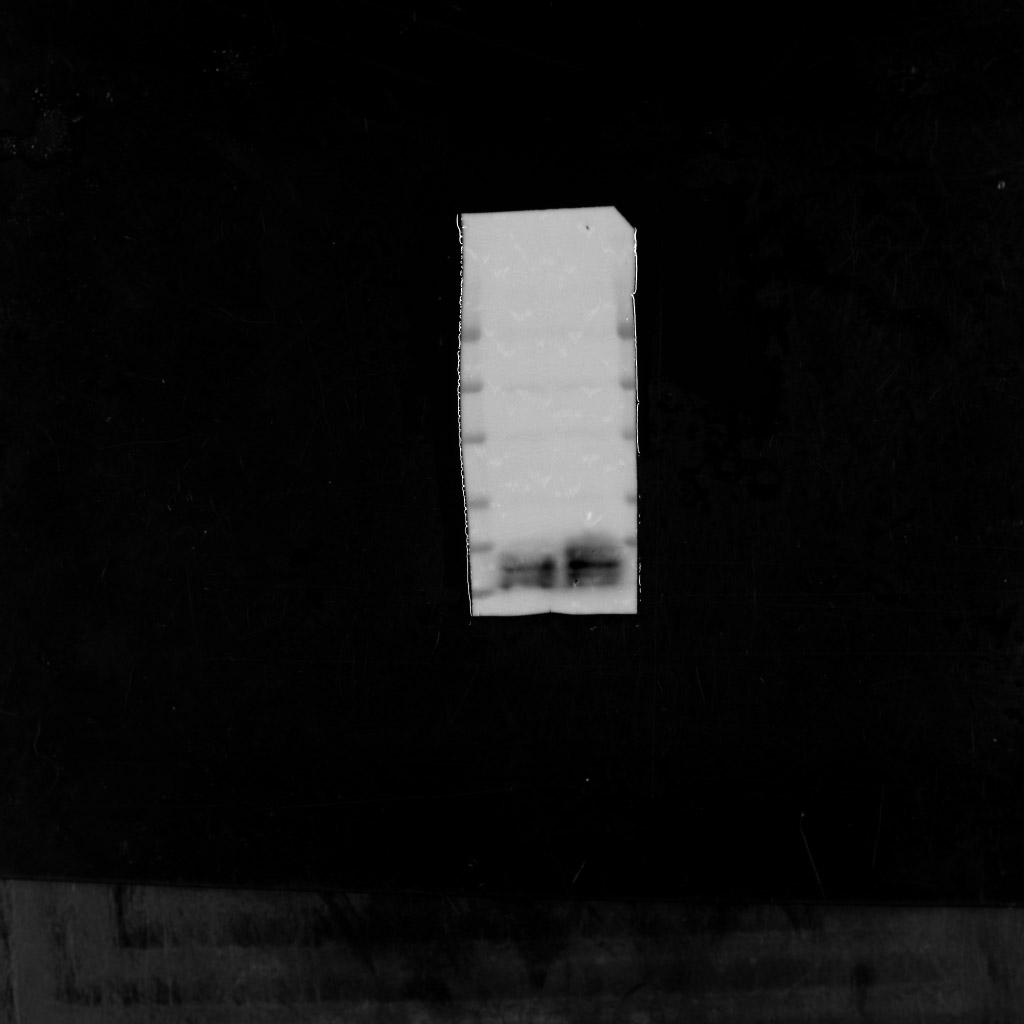

Supplement: Supplemental Information 9 — The raw images of tumor size in nude mice, western blot strips of tumor tissue, and corresponding statistical maps. [file peerj-12-18497-s009.zip › Raw tumor data of nude mice/western blot/未命名导出/bax 第1张.jpg]

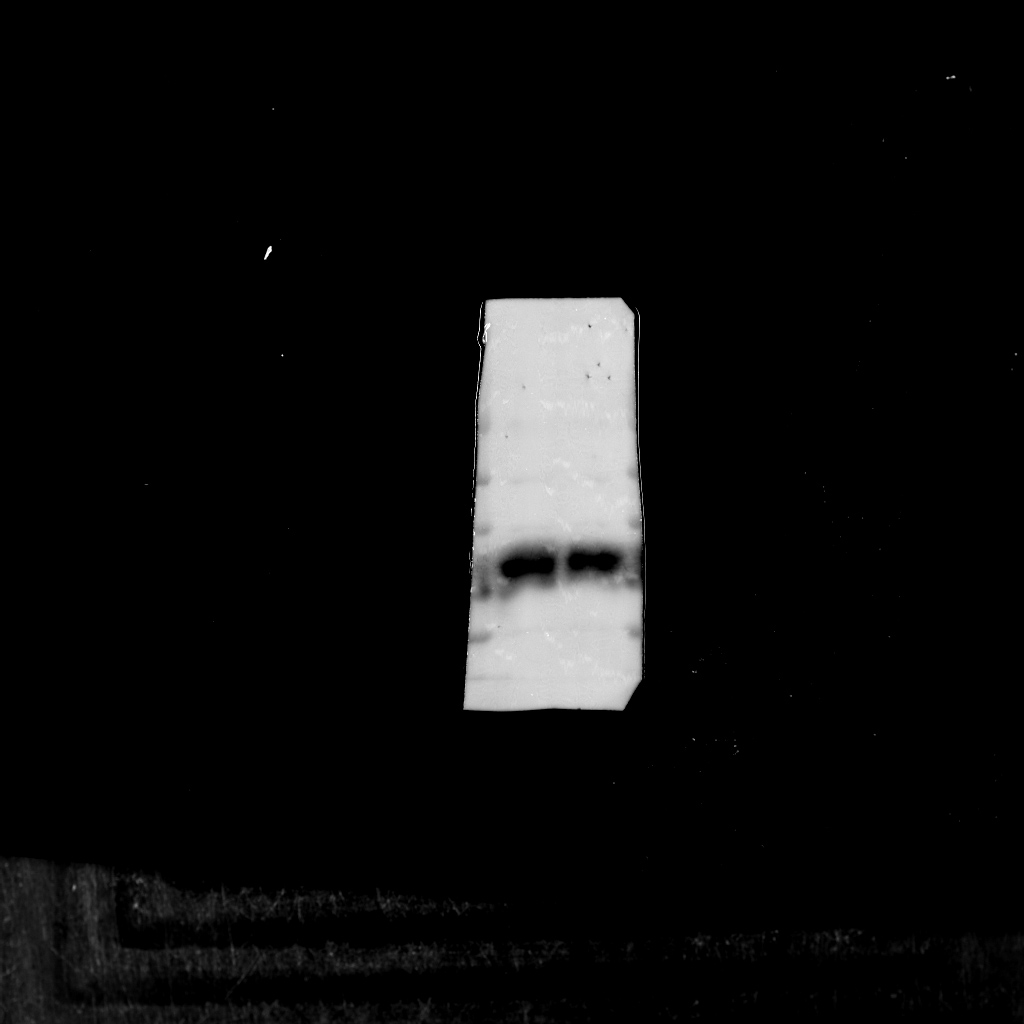

Supplement: Supplemental Information 9 — The raw images of tumor size in nude mice, western blot strips of tumor tissue, and corresponding statistical maps. [file peerj-12-18497-s009.zip › Raw tumor data of nude mice/western blot/未命名导出/bax 第2张 gapdh.jpg]

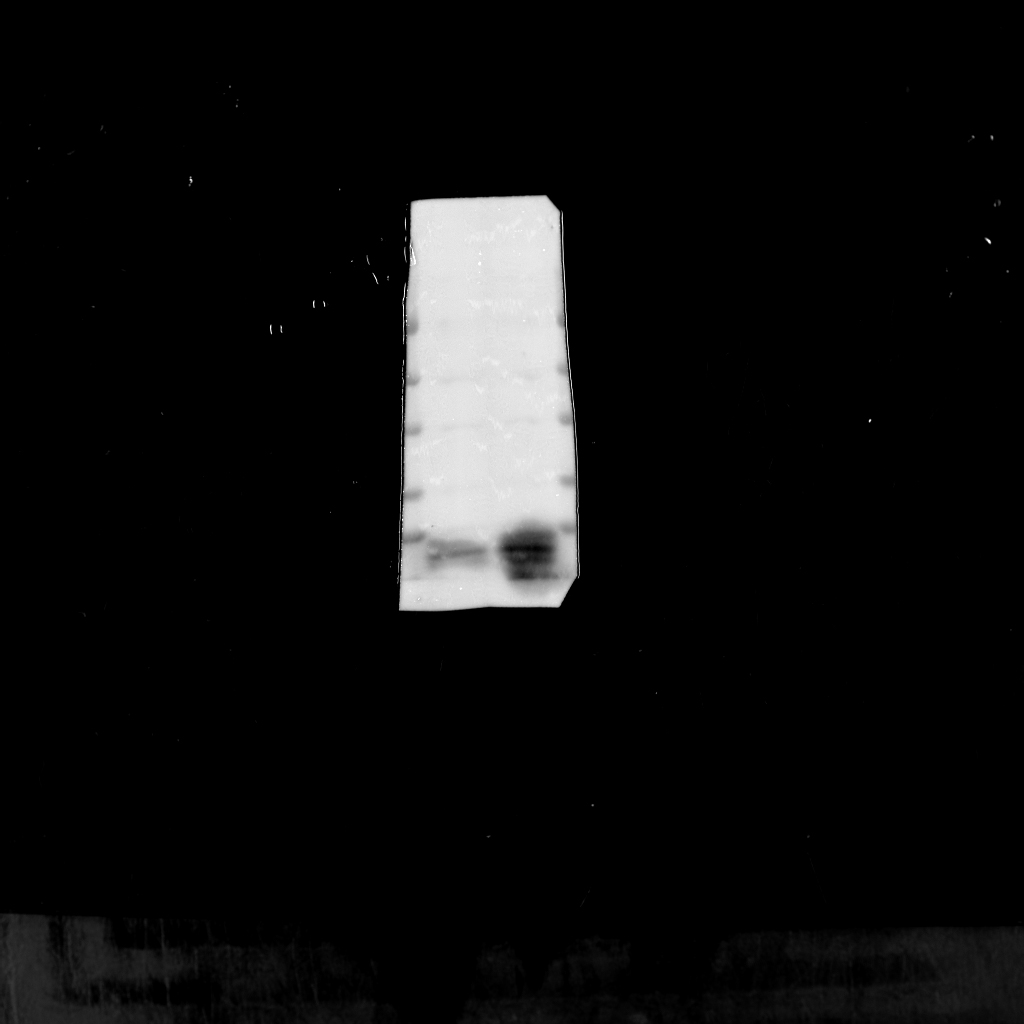

Supplement: Supplemental Information 9 — The raw images of tumor size in nude mice, western blot strips of tumor tissue, and corresponding statistical maps. [file peerj-12-18497-s009.zip › Raw tumor data of nude mice/western blot/未命名导出/bax 第2张.jpg]

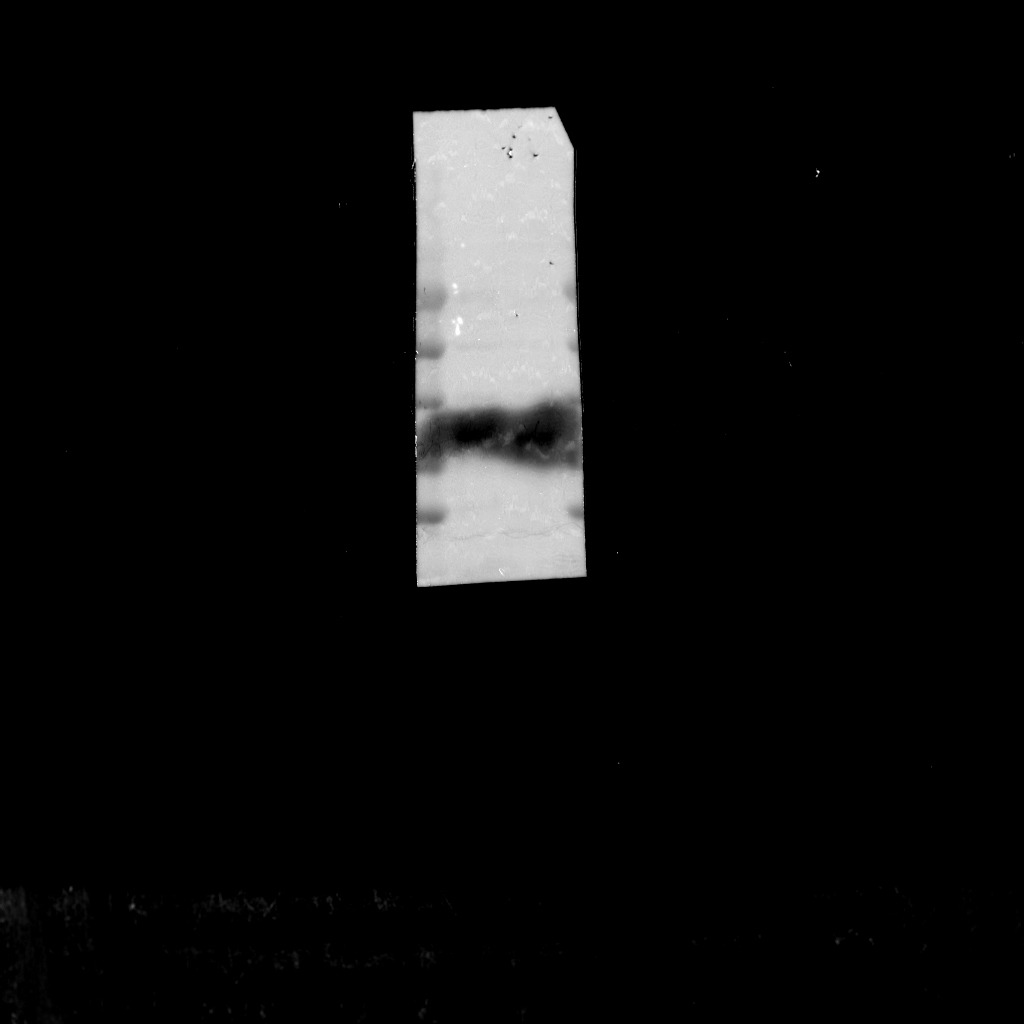

Supplement: Supplemental Information 9 — The raw images of tumor size in nude mice, western blot strips of tumor tissue, and corresponding statistical maps. [file peerj-12-18497-s009.zip › Raw tumor data of nude mice/western blot/未命名导出/bax 第3张 gapdh.jpg]

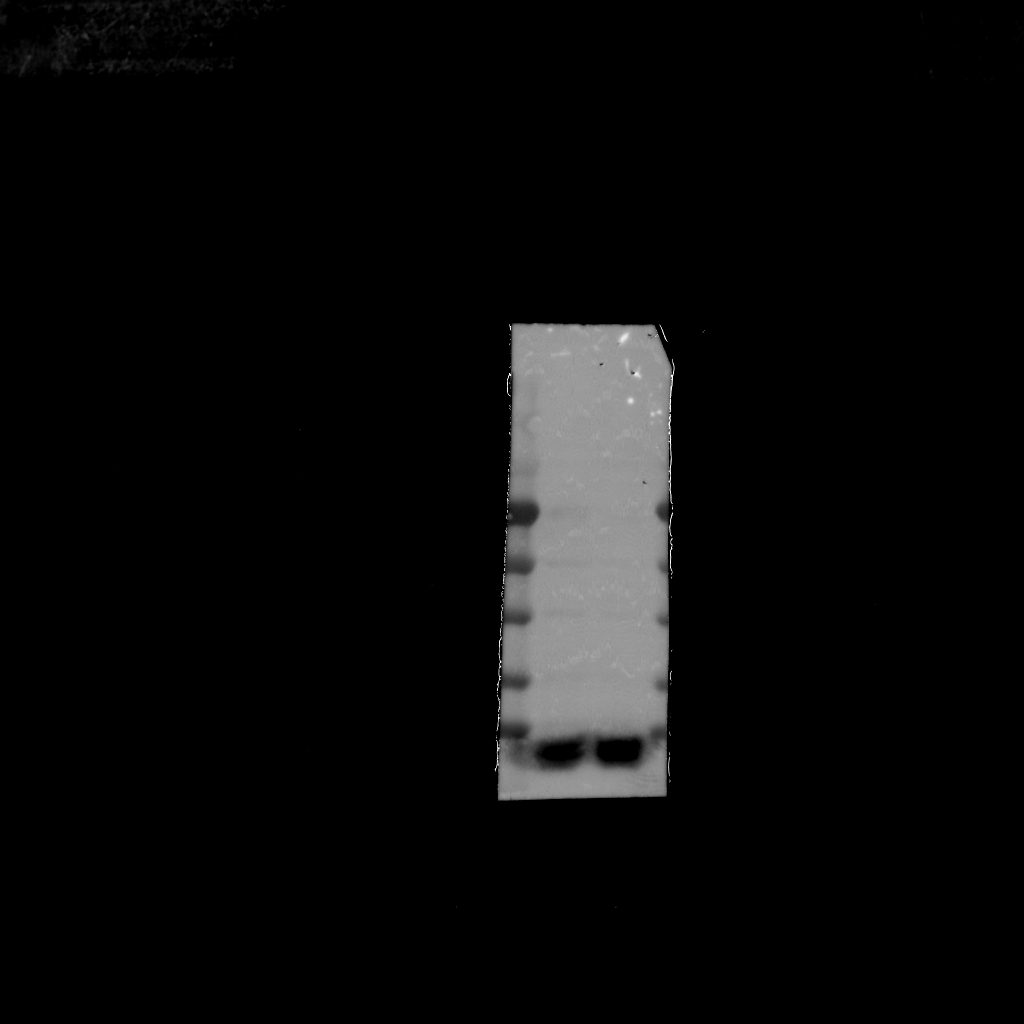

Supplement: Supplemental Information 9 — The raw images of tumor size in nude mice, western blot strips of tumor tissue, and corresponding statistical maps. [file peerj-12-18497-s009.zip › Raw tumor data of nude mice/western blot/未命名导出/bax 第3张.jpg]

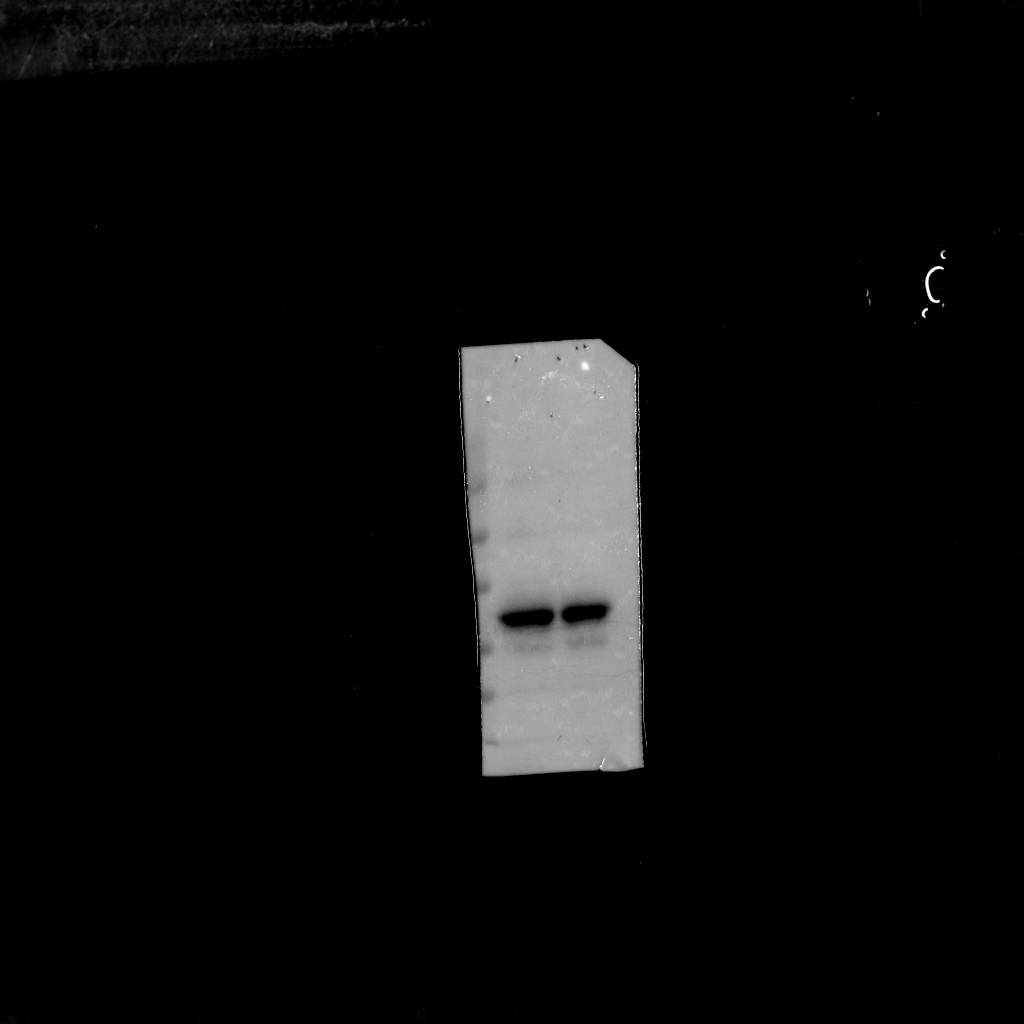

Supplement: Supplemental Information 9 — The raw images of tumor size in nude mice, western blot strips of tumor tissue, and corresponding statistical maps. [file peerj-12-18497-s009.zip › Raw tumor data of nude mice/western blot/未命名导出/bcl-2 第1张 gapdh.jpg]

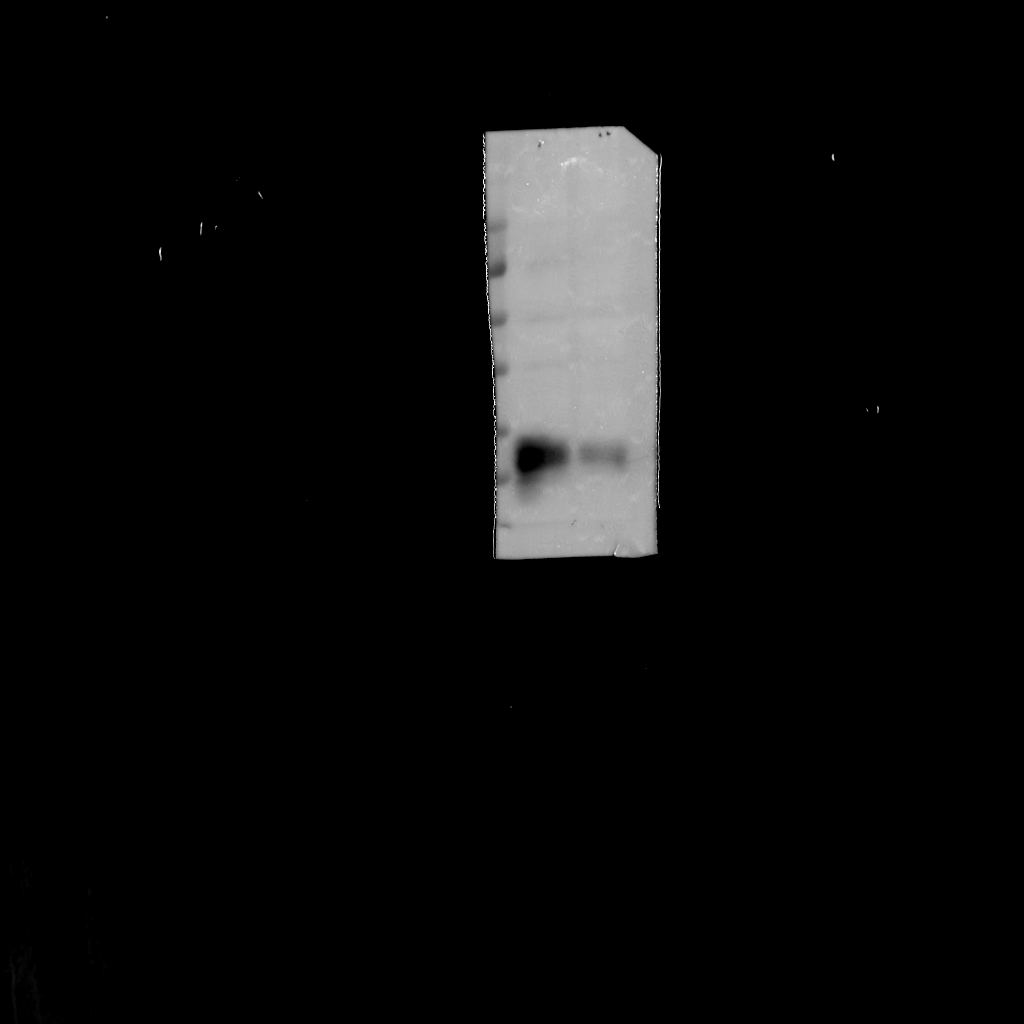

Supplement: Supplemental Information 9 — The raw images of tumor size in nude mice, western blot strips of tumor tissue, and corresponding statistical maps. [file peerj-12-18497-s009.zip › Raw tumor data of nude mice/western blot/未命名导出/bcl-2 第1张.jpg]

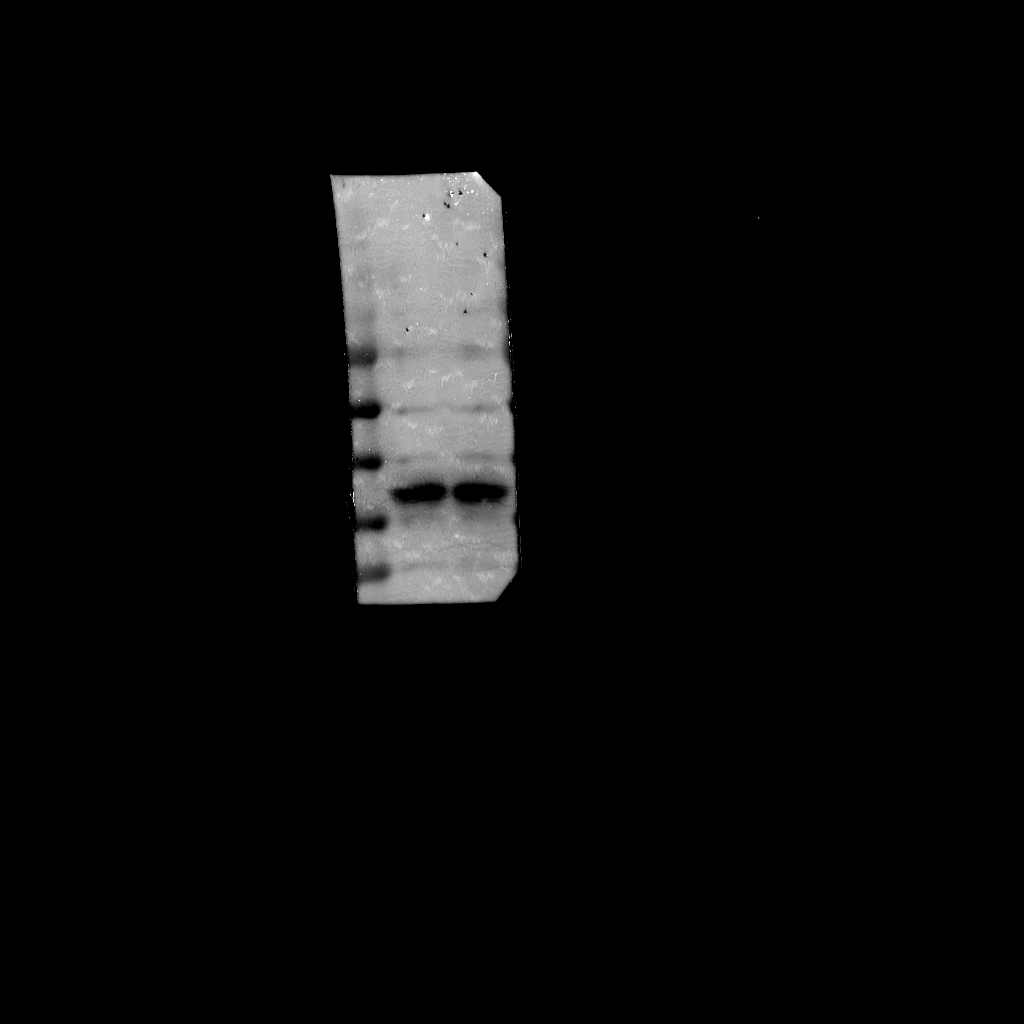

Supplement: Supplemental Information 9 — The raw images of tumor size in nude mice, western blot strips of tumor tissue, and corresponding statistical maps. [file peerj-12-18497-s009.zip › Raw tumor data of nude mice/western blot/未命名导出/bcl-2 第2张 gapdh.jpg]

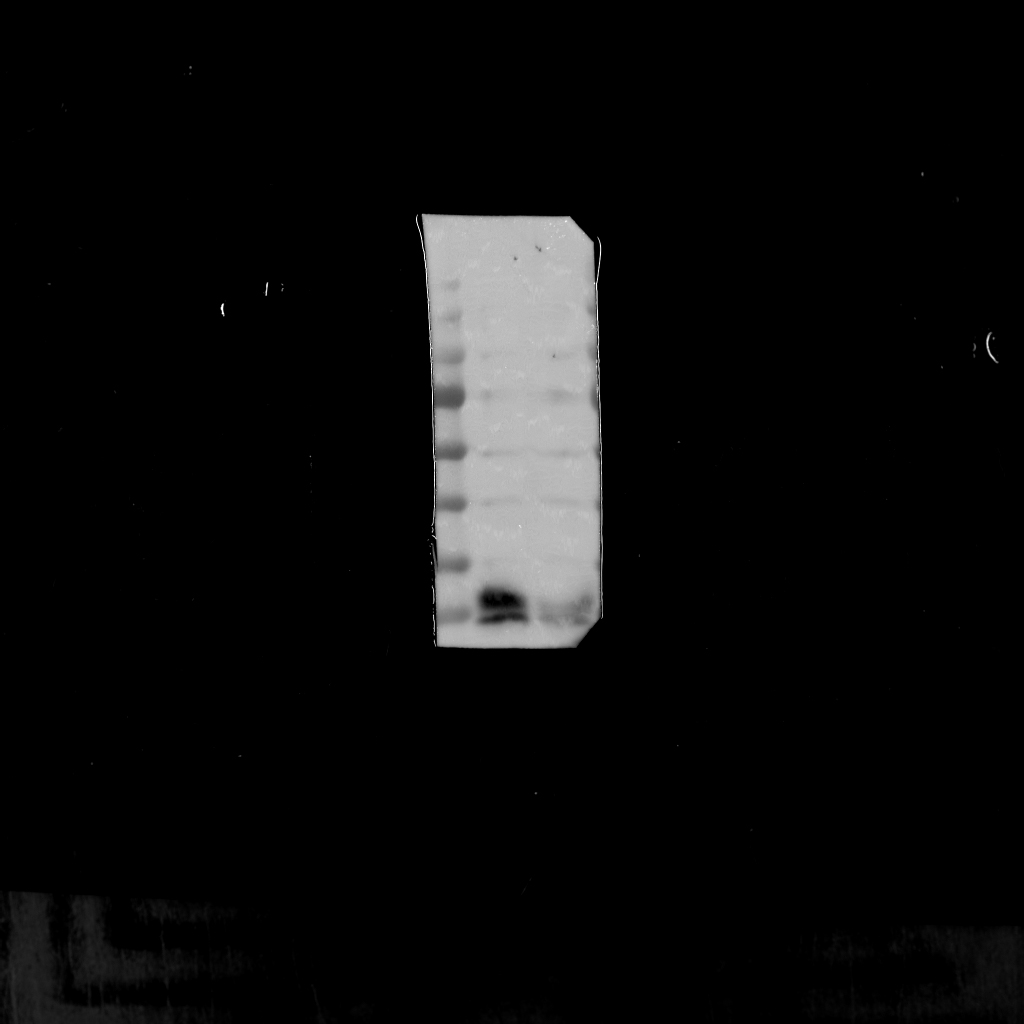

Supplement: Supplemental Information 9 — The raw images of tumor size in nude mice, western blot strips of tumor tissue, and corresponding statistical maps. [file peerj-12-18497-s009.zip › Raw tumor data of nude mice/western blot/未命名导出/bcl-2 第2张.jpg]

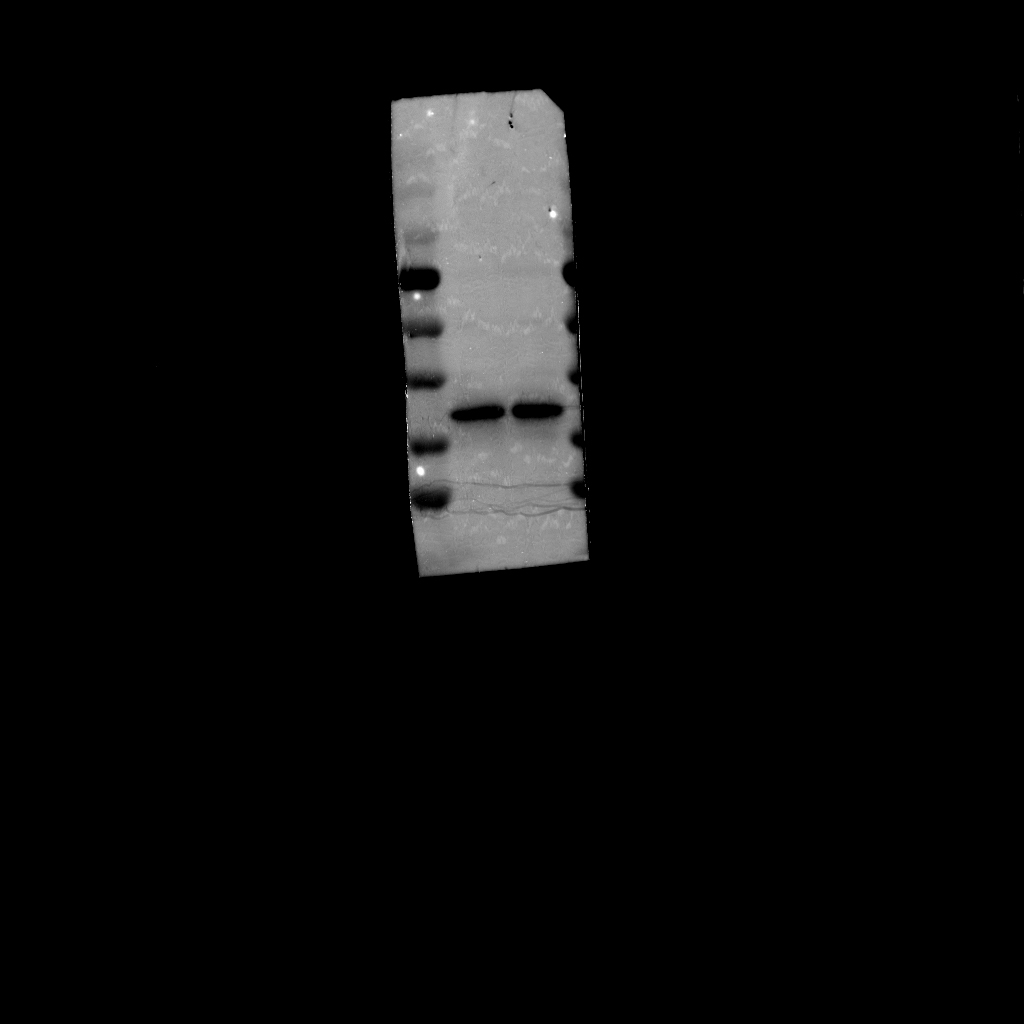

Supplement: Supplemental Information 9 — The raw images of tumor size in nude mice, western blot strips of tumor tissue, and corresponding statistical maps. [file peerj-12-18497-s009.zip › Raw tumor data of nude mice/western blot/未命名导出/bcl-2 第3张gapdh.jpg]

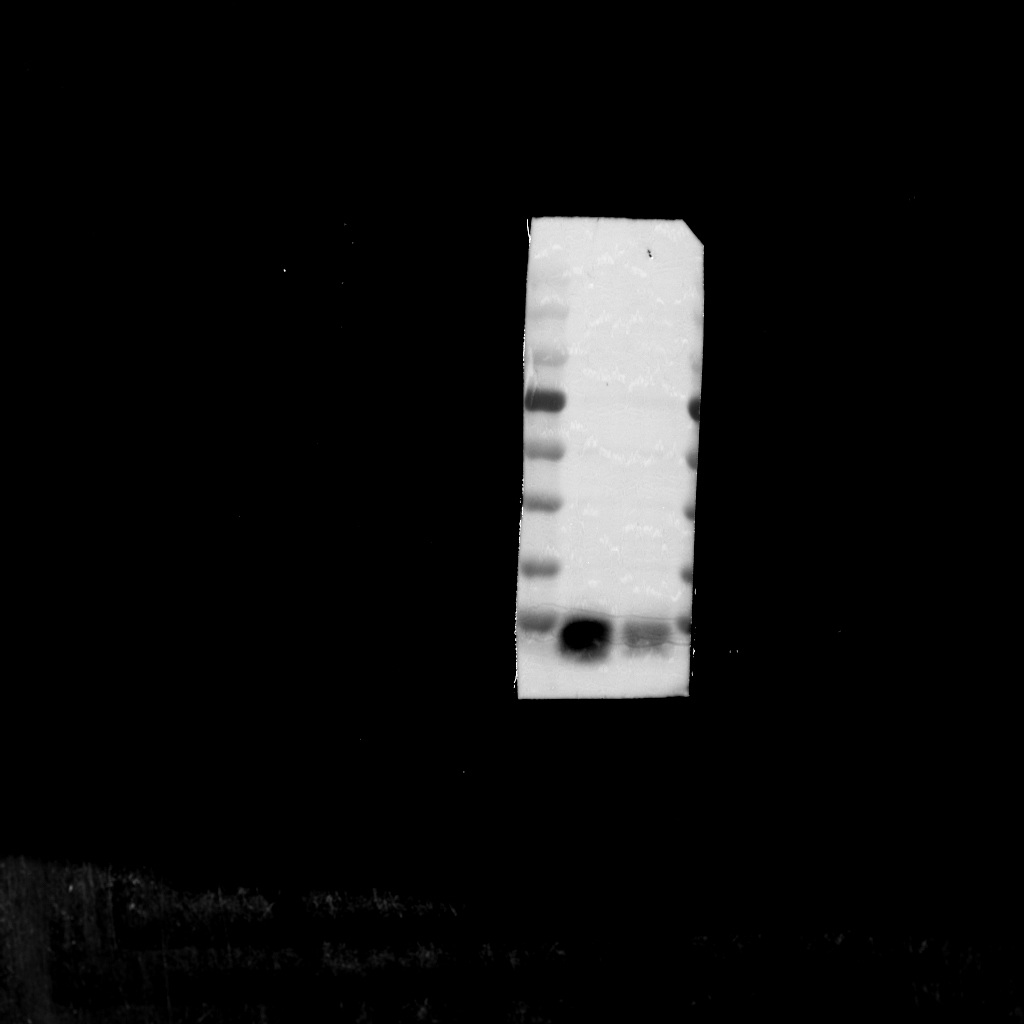

Supplement: Supplemental Information 9 — The raw images of tumor size in nude mice, western blot strips of tumor tissue, and corresponding statistical maps. [file peerj-12-18497-s009.zip › Raw tumor data of nude mice/western blot/未命名导出/bcl-2-第3张.jpg]

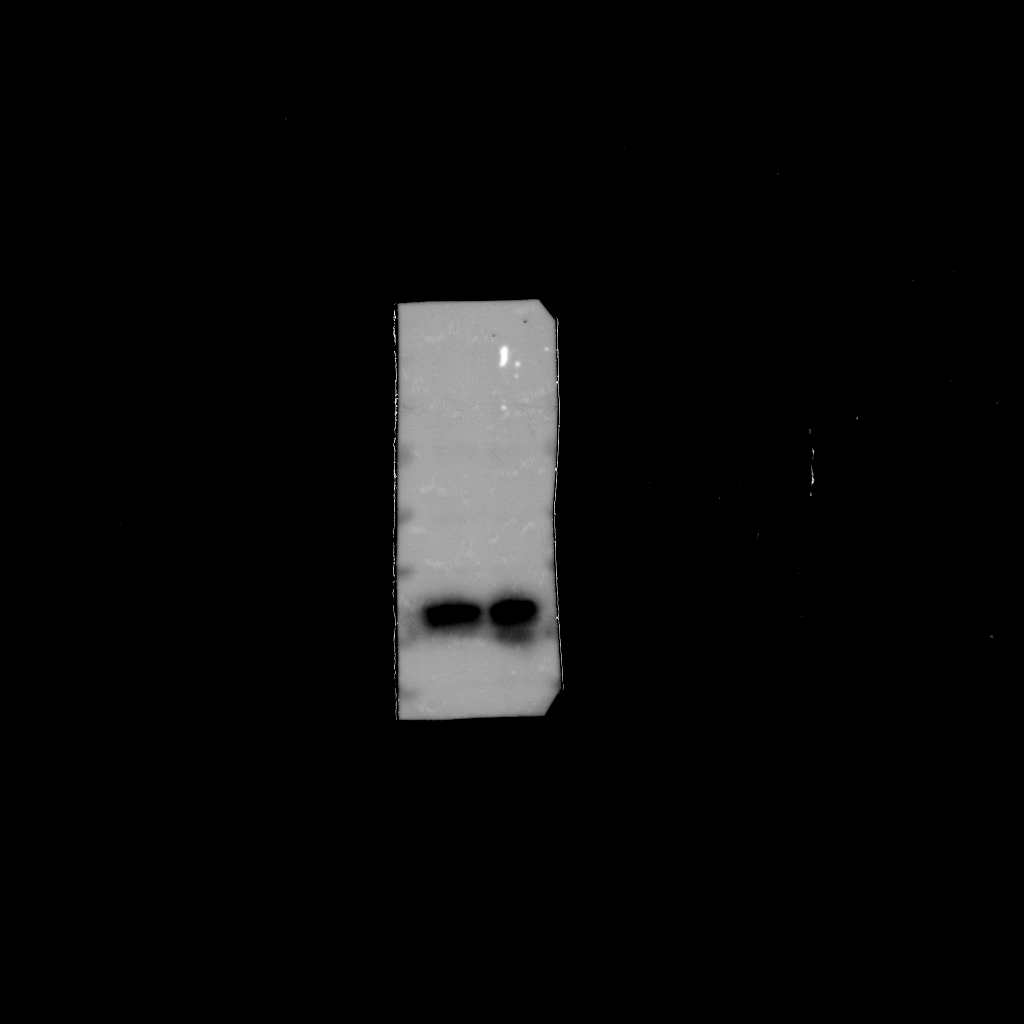

Supplement: Supplemental Information 9 — The raw images of tumor size in nude mice, western blot strips of tumor tissue, and corresponding statistical maps. [file peerj-12-18497-s009.zip › Raw tumor data of nude mice/western blot/未命名导出/beita-catenin 第1张 gapdh.jpg]

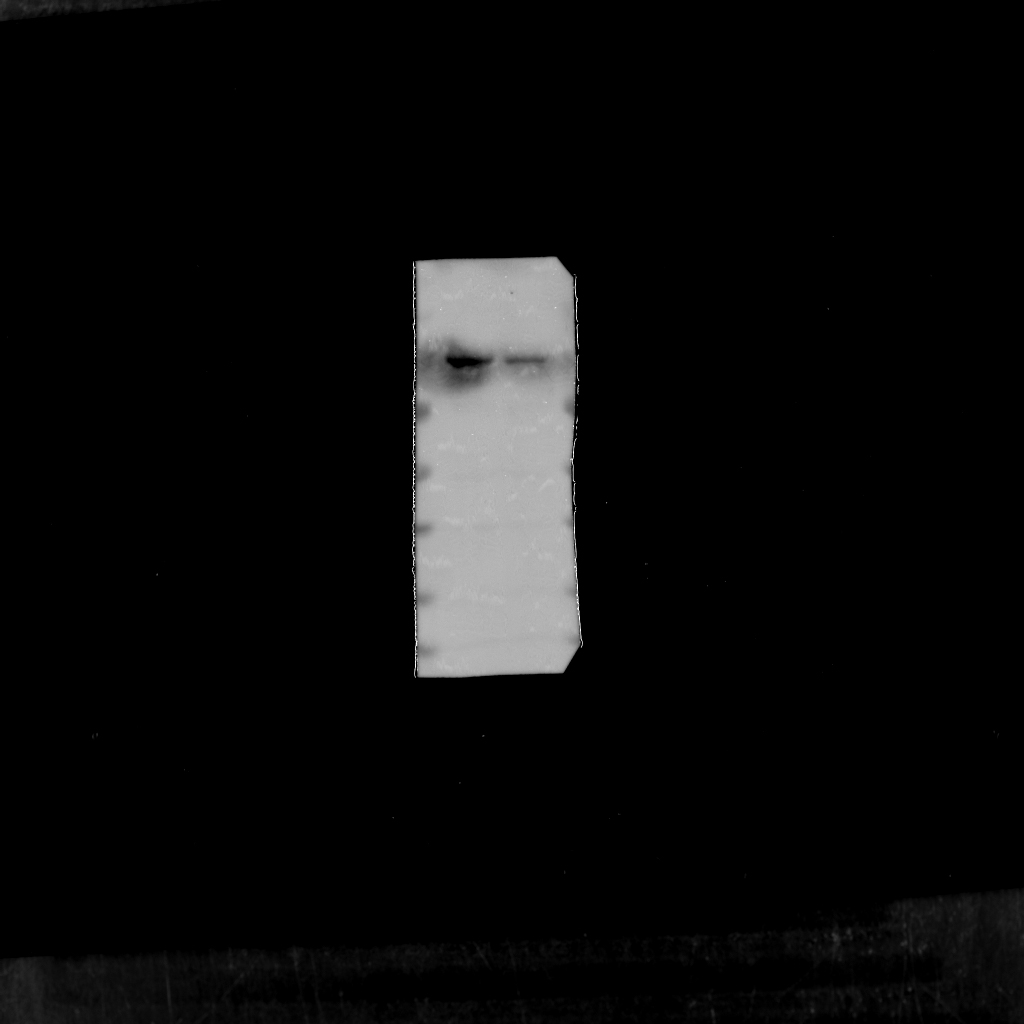

Supplement: Supplemental Information 9 — The raw images of tumor size in nude mice, western blot strips of tumor tissue, and corresponding statistical maps. [file peerj-12-18497-s009.zip › Raw tumor data of nude mice/western blot/未命名导出/beita-catenin 第1张.jpg]

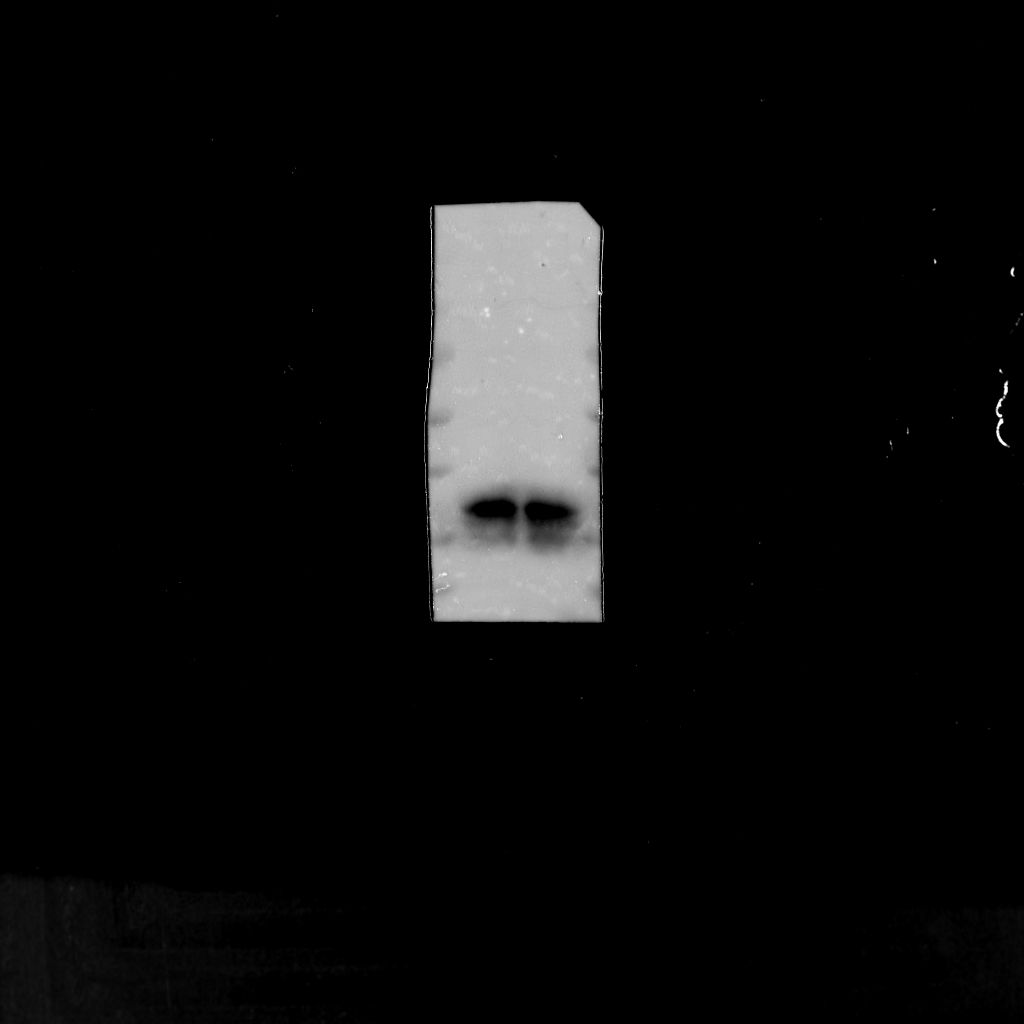

Supplement: Supplemental Information 9 — The raw images of tumor size in nude mice, western blot strips of tumor tissue, and corresponding statistical maps. [file peerj-12-18497-s009.zip › Raw tumor data of nude mice/western blot/未命名导出/beita-catenin 第2张 gapdh.jpg]

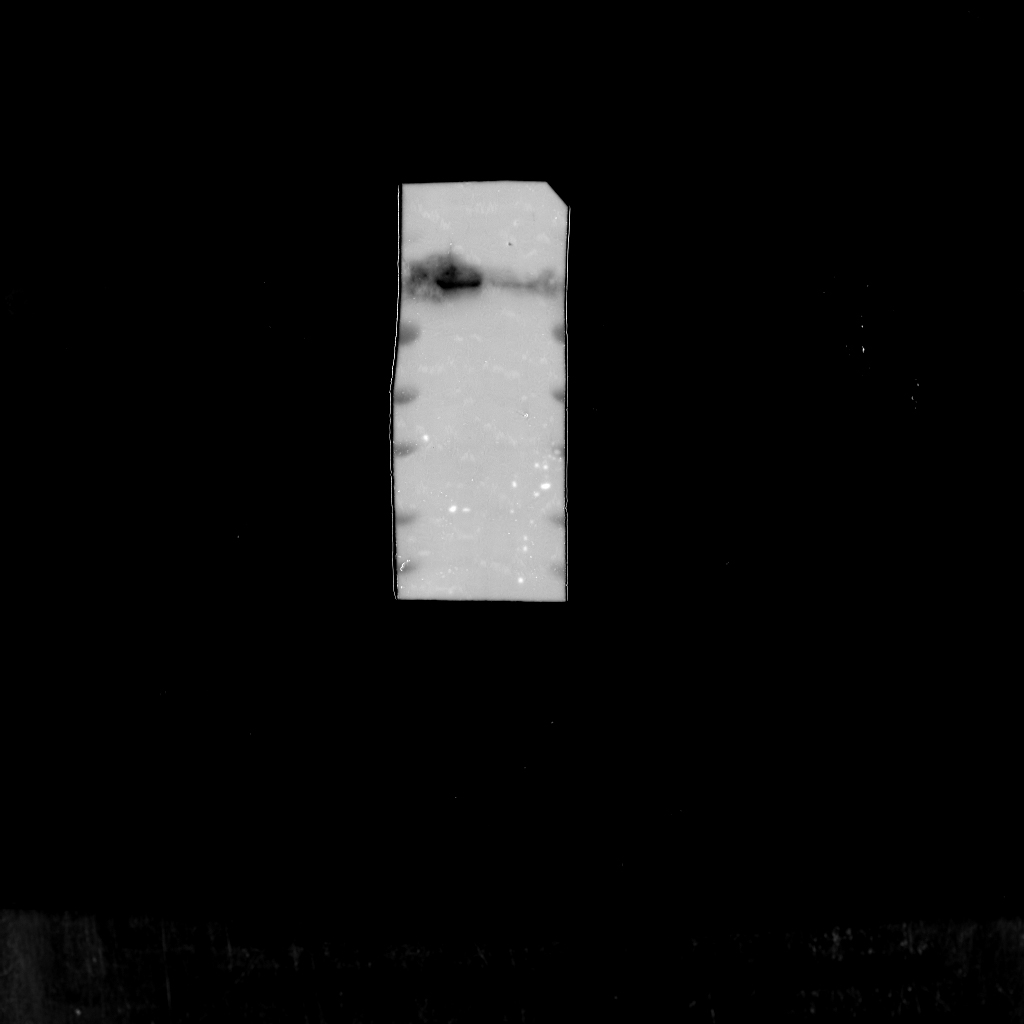

Supplement: Supplemental Information 9 — The raw images of tumor size in nude mice, western blot strips of tumor tissue, and corresponding statistical maps. [file peerj-12-18497-s009.zip › Raw tumor data of nude mice/western blot/未命名导出/beita-catenin 第2张.jpg]

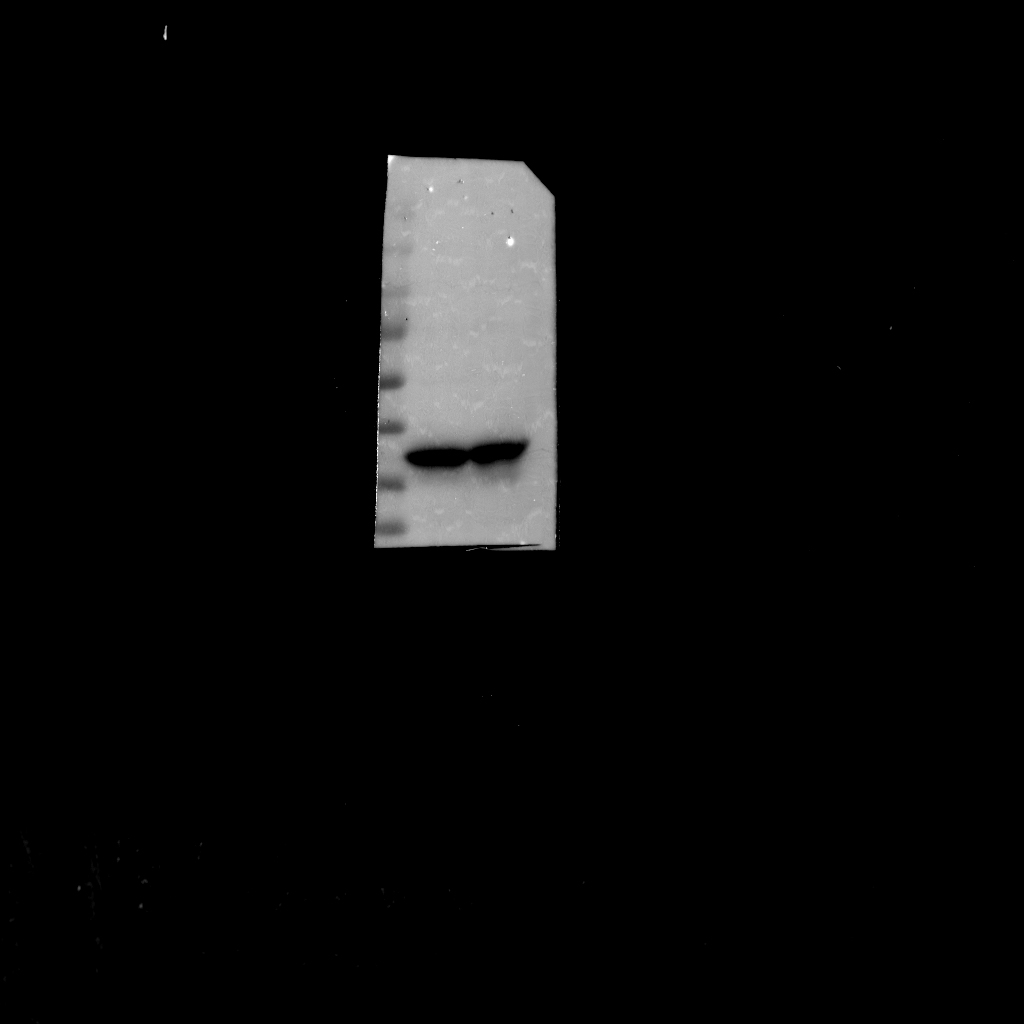

Supplement: Supplemental Information 9 — The raw images of tumor size in nude mice, western blot strips of tumor tissue, and corresponding statistical maps. [file peerj-12-18497-s009.zip › Raw tumor data of nude mice/western blot/未命名导出/beita-catenin 第3张 gapdh.jpg]

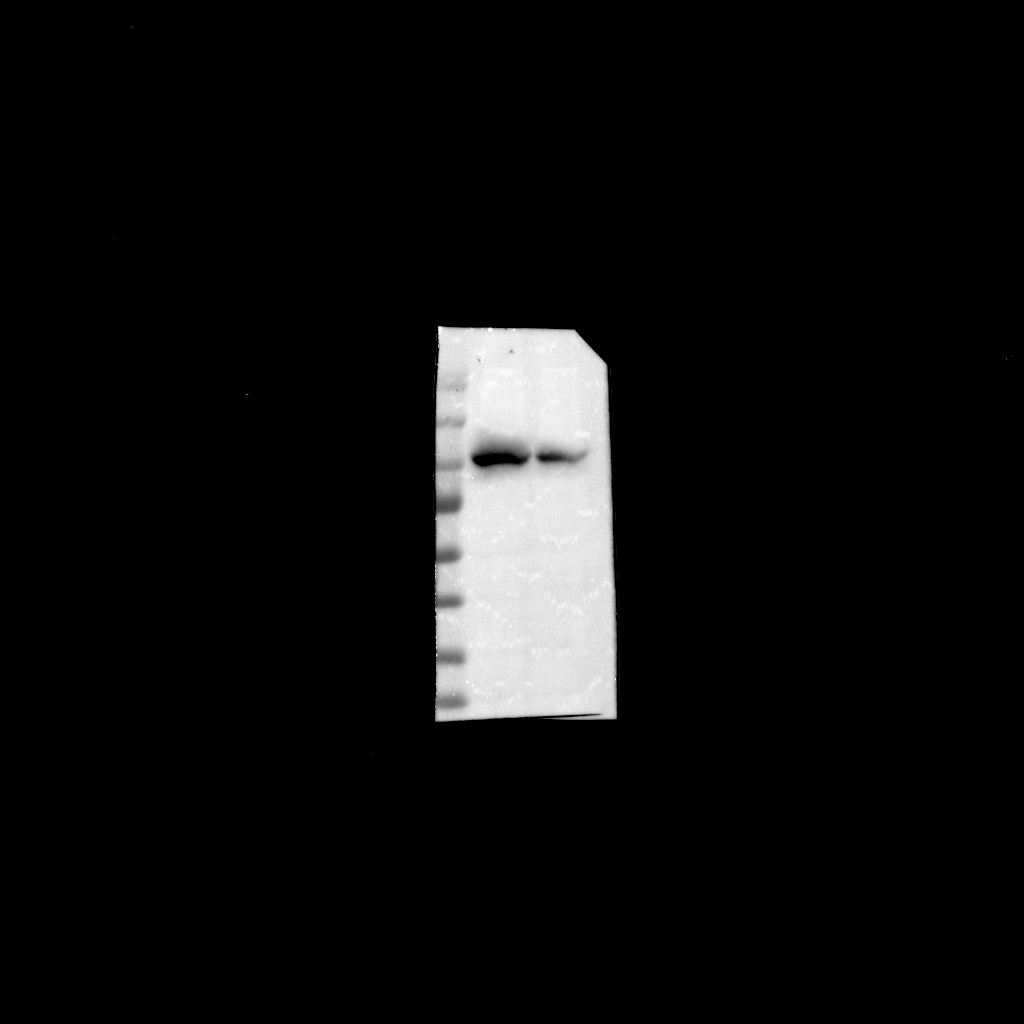

Supplement: Supplemental Information 9 — The raw images of tumor size in nude mice, western blot strips of tumor tissue, and corresponding statistical maps. [file peerj-12-18497-s009.zip › Raw tumor data of nude mice/western blot/未命名导出/beita-catenin 第3张.jpg]

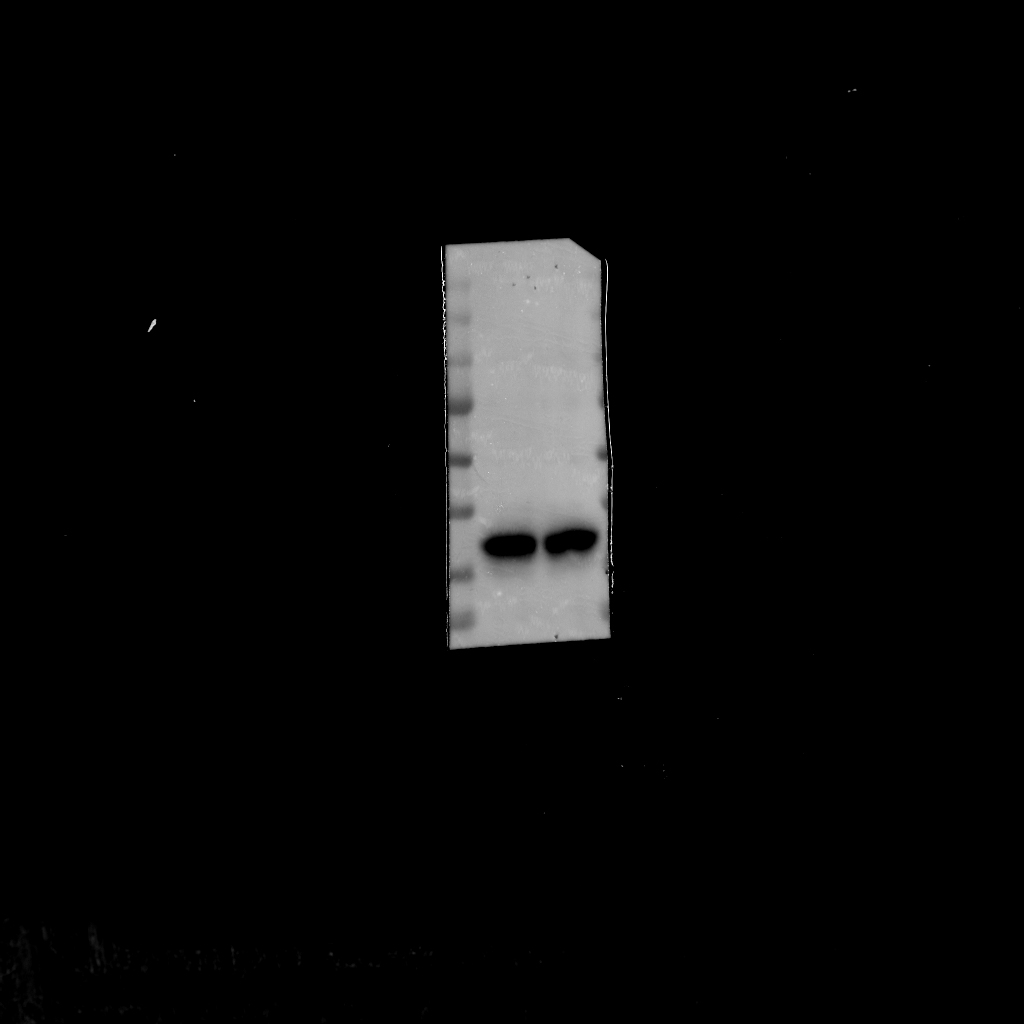

Supplement: Supplemental Information 9 — The raw images of tumor size in nude mice, western blot strips of tumor tissue, and corresponding statistical maps. [file peerj-12-18497-s009.zip › Raw tumor data of nude mice/western blot/未命名导出/c-myc 第1张 gapdh.jpg]

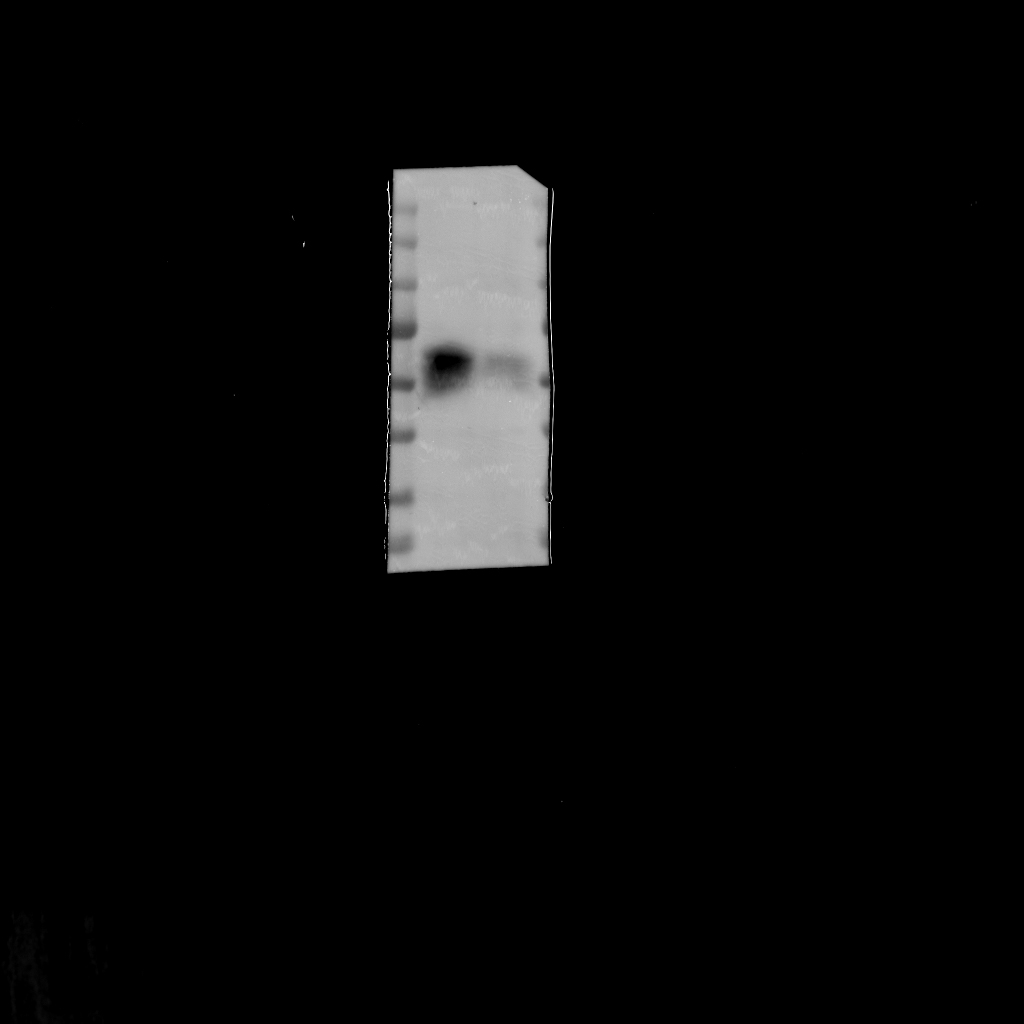

Supplement: Supplemental Information 9 — The raw images of tumor size in nude mice, western blot strips of tumor tissue, and corresponding statistical maps. [file peerj-12-18497-s009.zip › Raw tumor data of nude mice/western blot/未命名导出/c-myc 第1张.jpg]

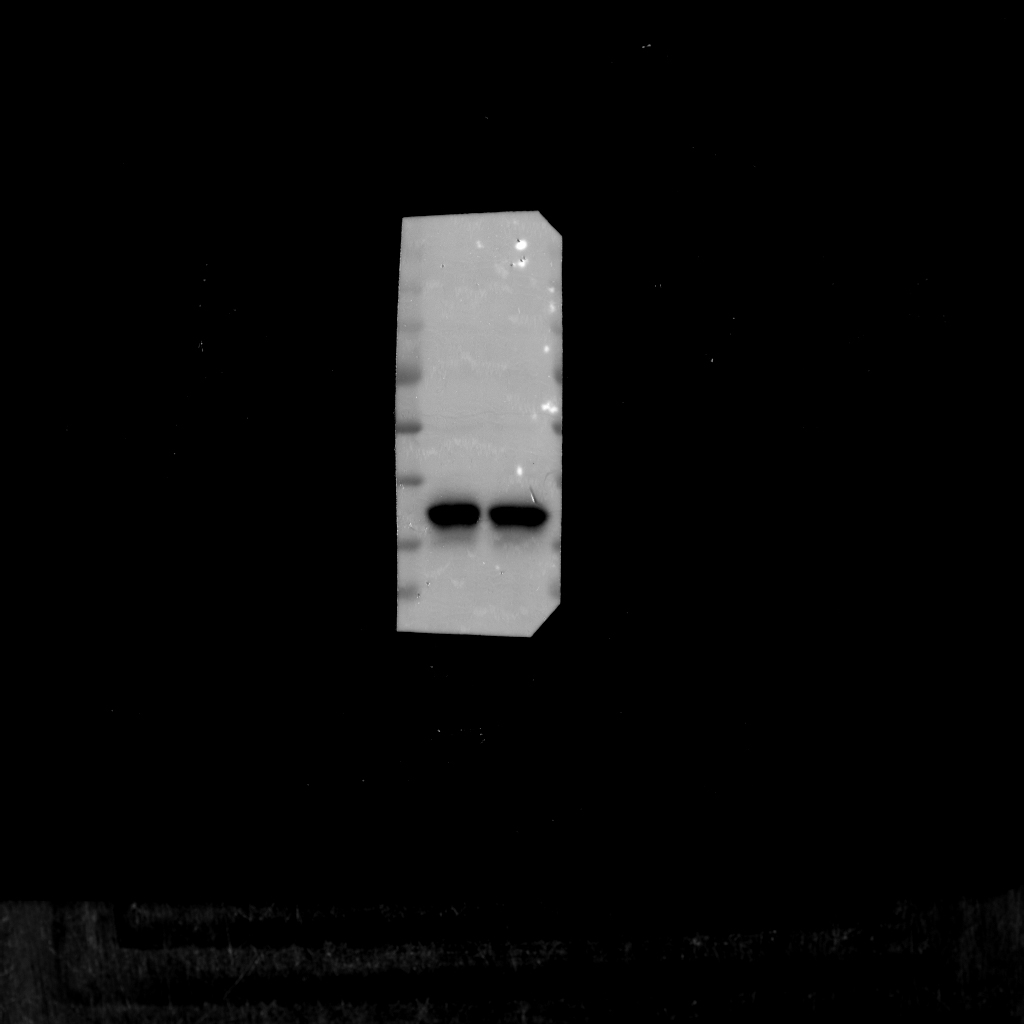

Supplement: Supplemental Information 9 — The raw images of tumor size in nude mice, western blot strips of tumor tissue, and corresponding statistical maps. [file peerj-12-18497-s009.zip › Raw tumor data of nude mice/western blot/未命名导出/c-myc 第2张 gapdh.jpg]

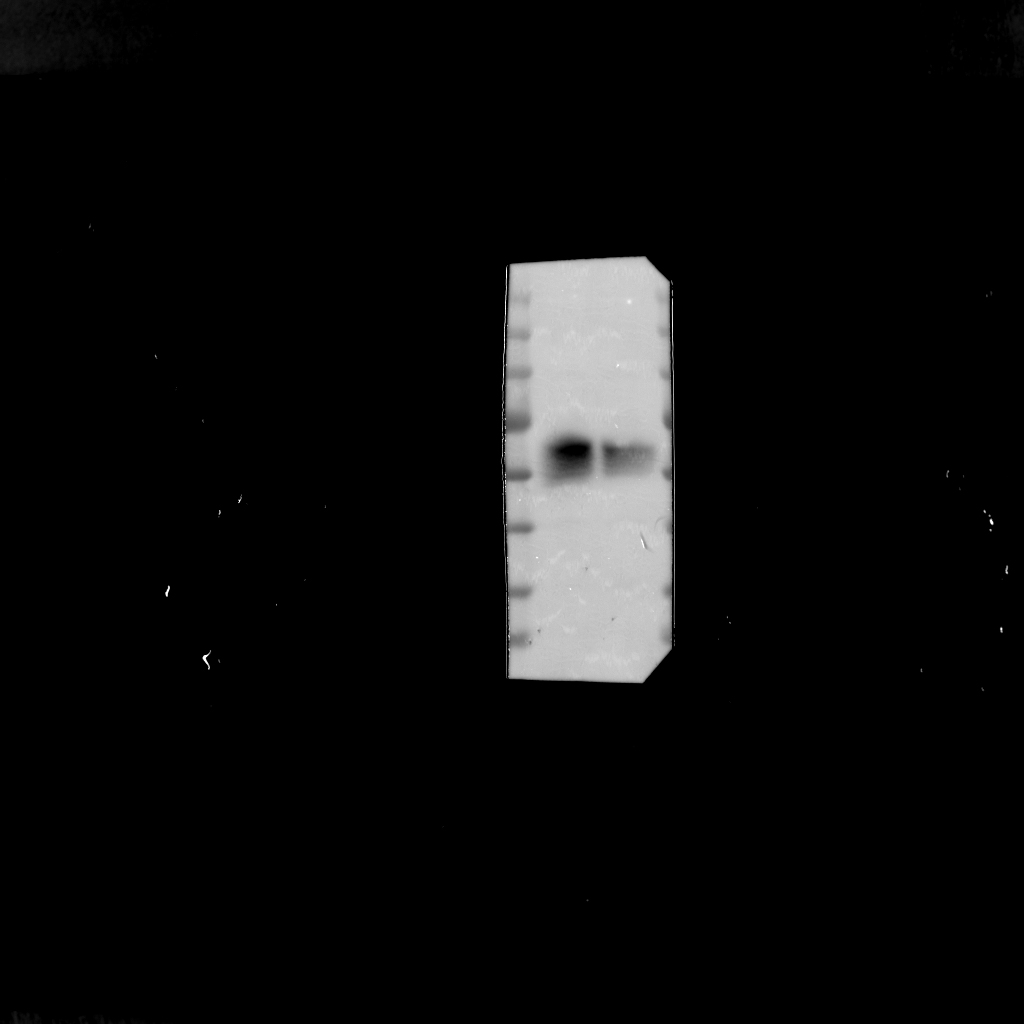

Supplement: Supplemental Information 9 — The raw images of tumor size in nude mice, western blot strips of tumor tissue, and corresponding statistical maps. [file peerj-12-18497-s009.zip › Raw tumor data of nude mice/western blot/未命名导出/c-myc 第2张.jpg]

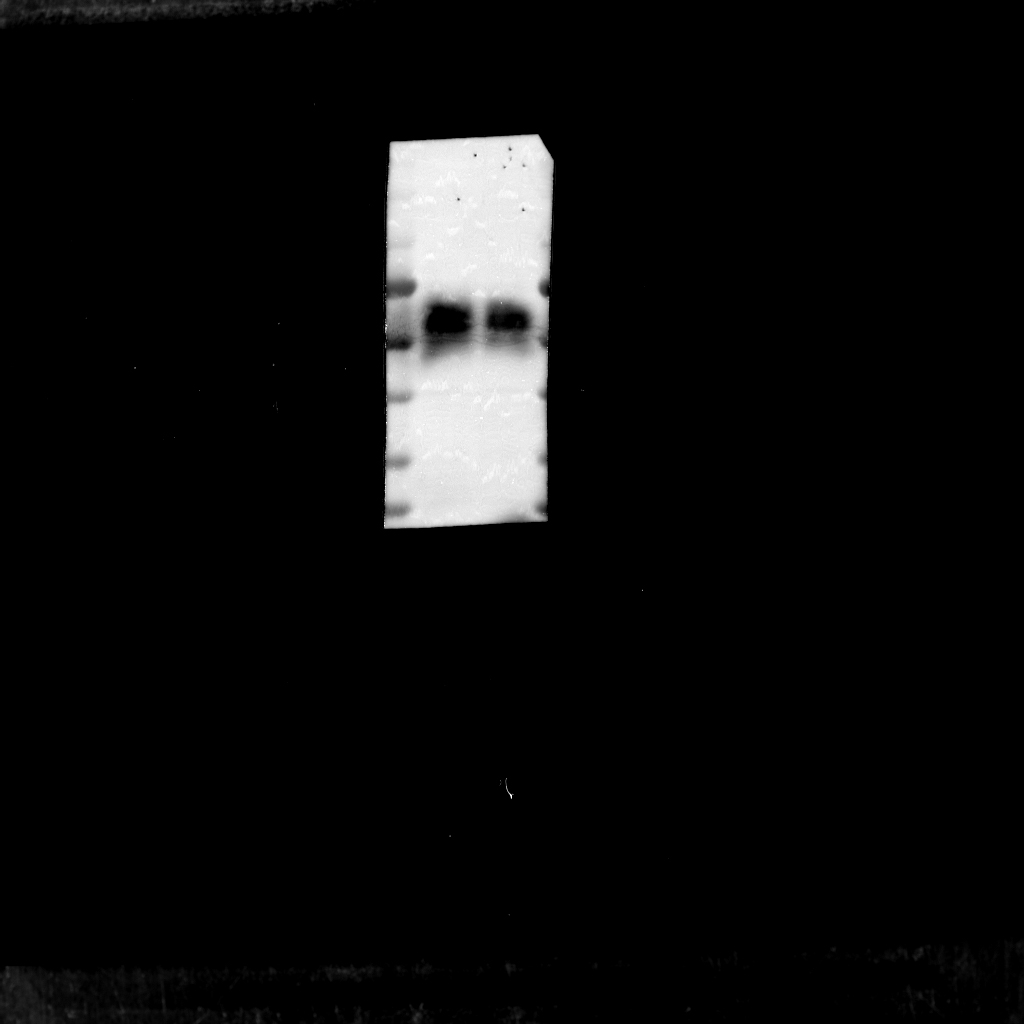

Supplement: Supplemental Information 9 — The raw images of tumor size in nude mice, western blot strips of tumor tissue, and corresponding statistical maps. [file peerj-12-18497-s009.zip › Raw tumor data of nude mice/western blot/未命名导出/c-myc 第3张.jpg]

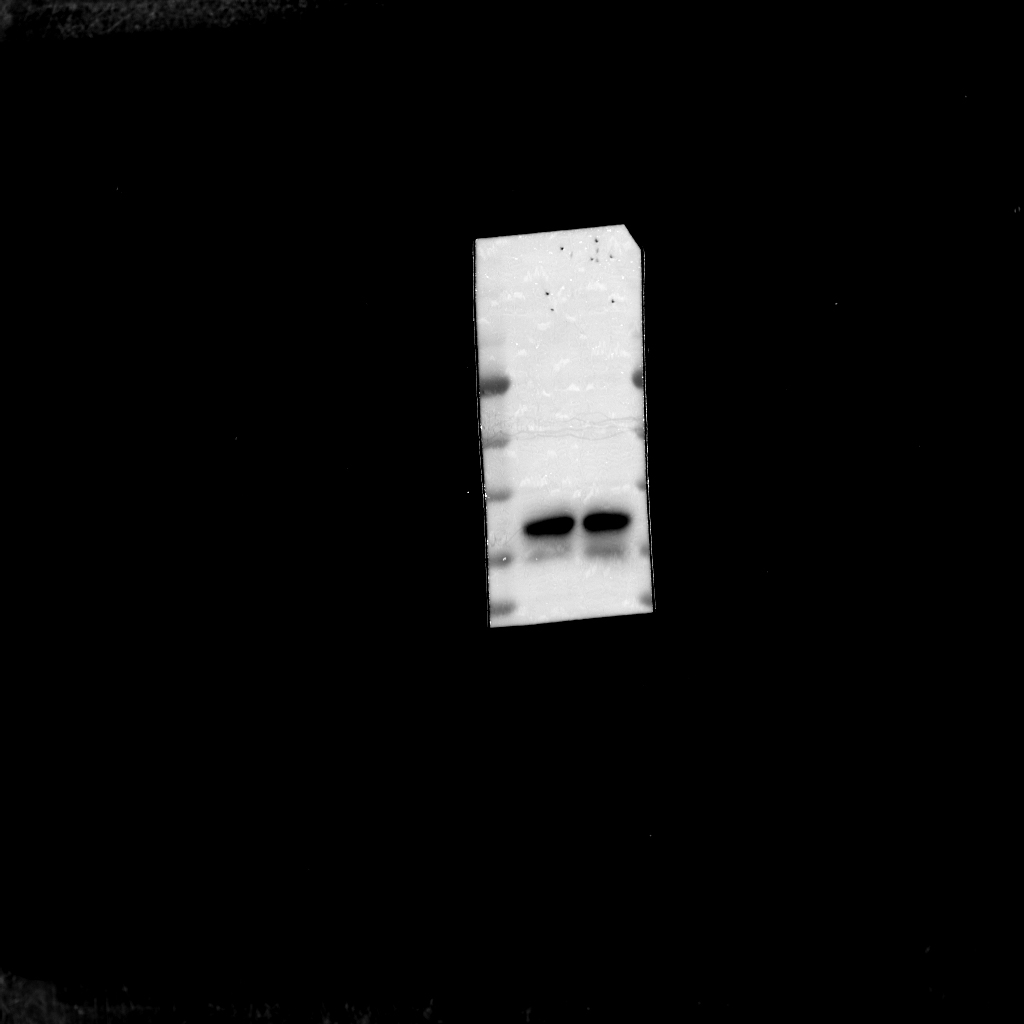

Supplement: Supplemental Information 9 — The raw images of tumor size in nude mice, western blot strips of tumor tissue, and corresponding statistical maps. [file peerj-12-18497-s009.zip › Raw tumor data of nude mice/western blot/未命名导出/c-myc 第3张gapdh.jpg]

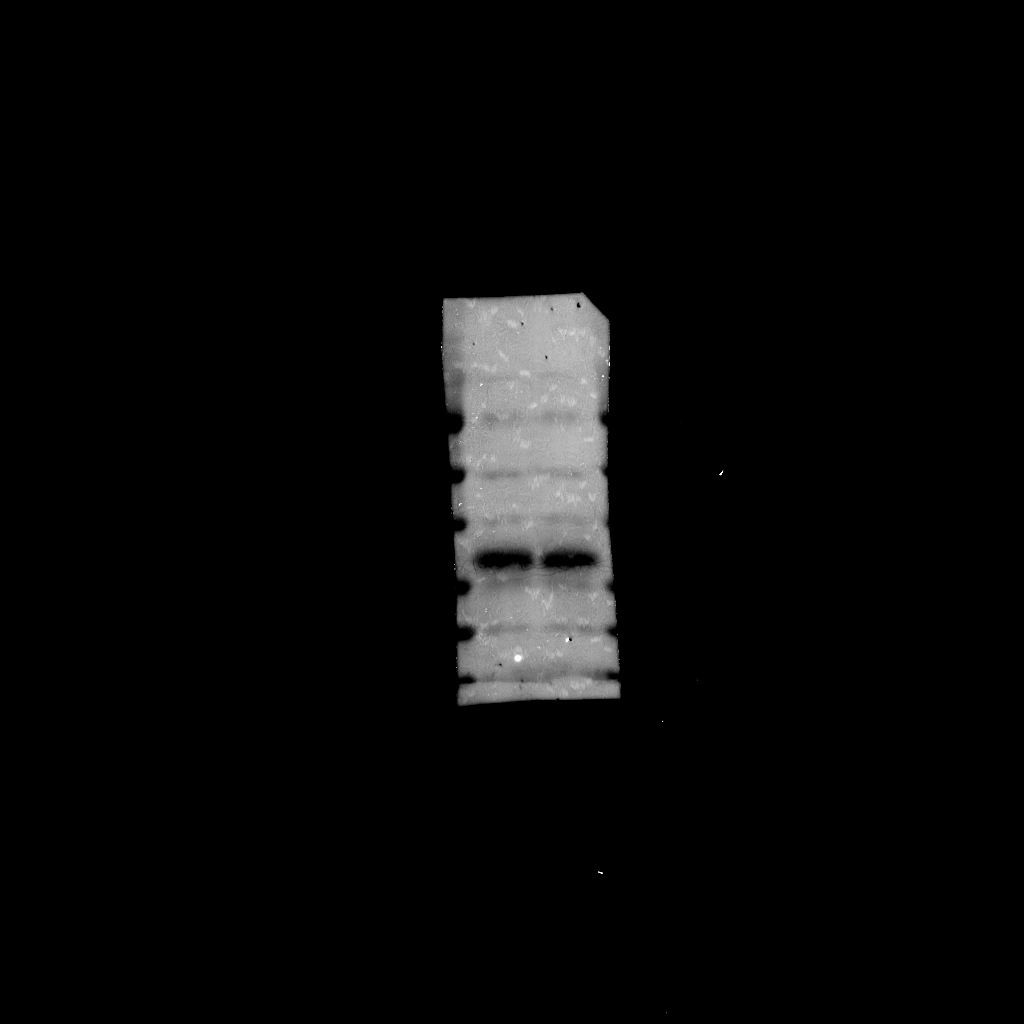

Supplement: Supplemental Information 9 — The raw images of tumor size in nude mice, western blot strips of tumor tissue, and corresponding statistical maps. [file peerj-12-18497-s009.zip › Raw tumor data of nude mice/western blot/未命名导出/clec3b 第1张 gapdh.jpg]

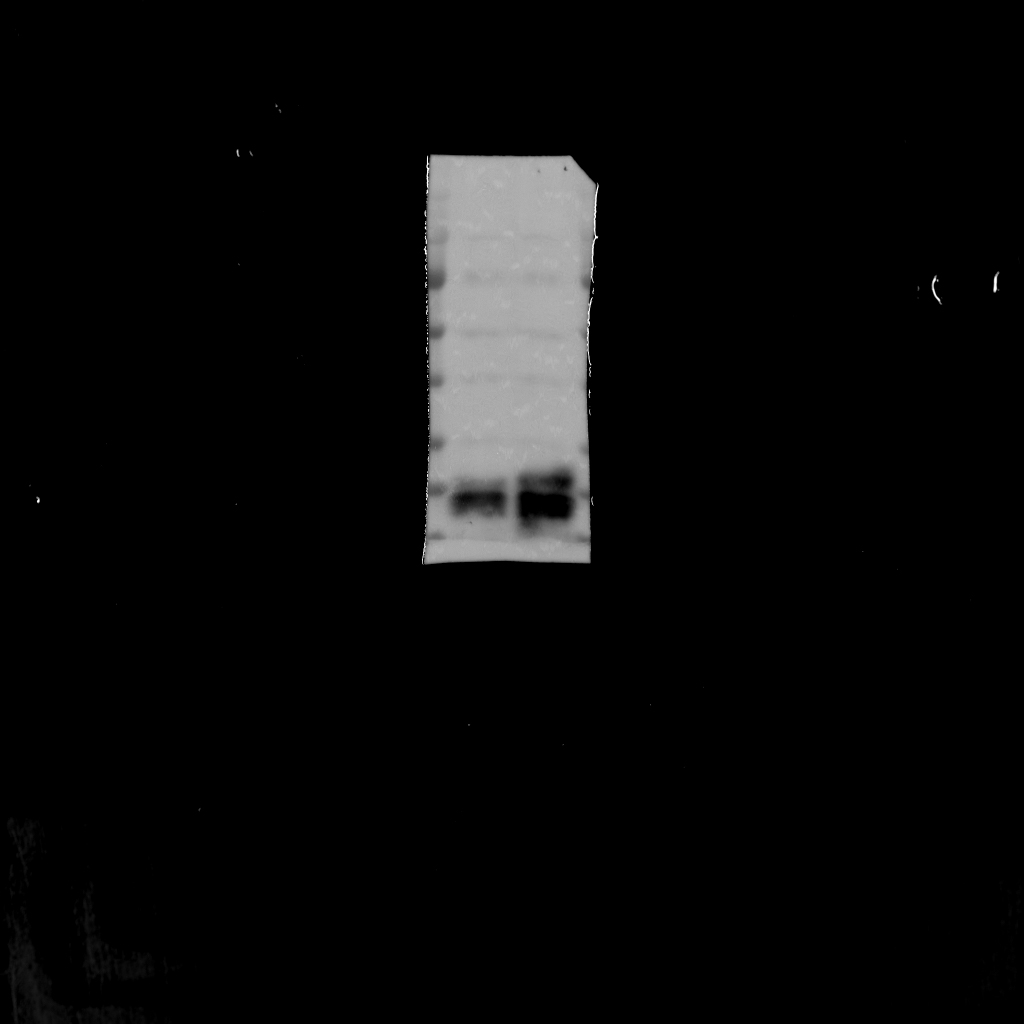

Supplement: Supplemental Information 9 — The raw images of tumor size in nude mice, western blot strips of tumor tissue, and corresponding statistical maps. [file peerj-12-18497-s009.zip › Raw tumor data of nude mice/western blot/未命名导出/clec3b 第1张.jpg]

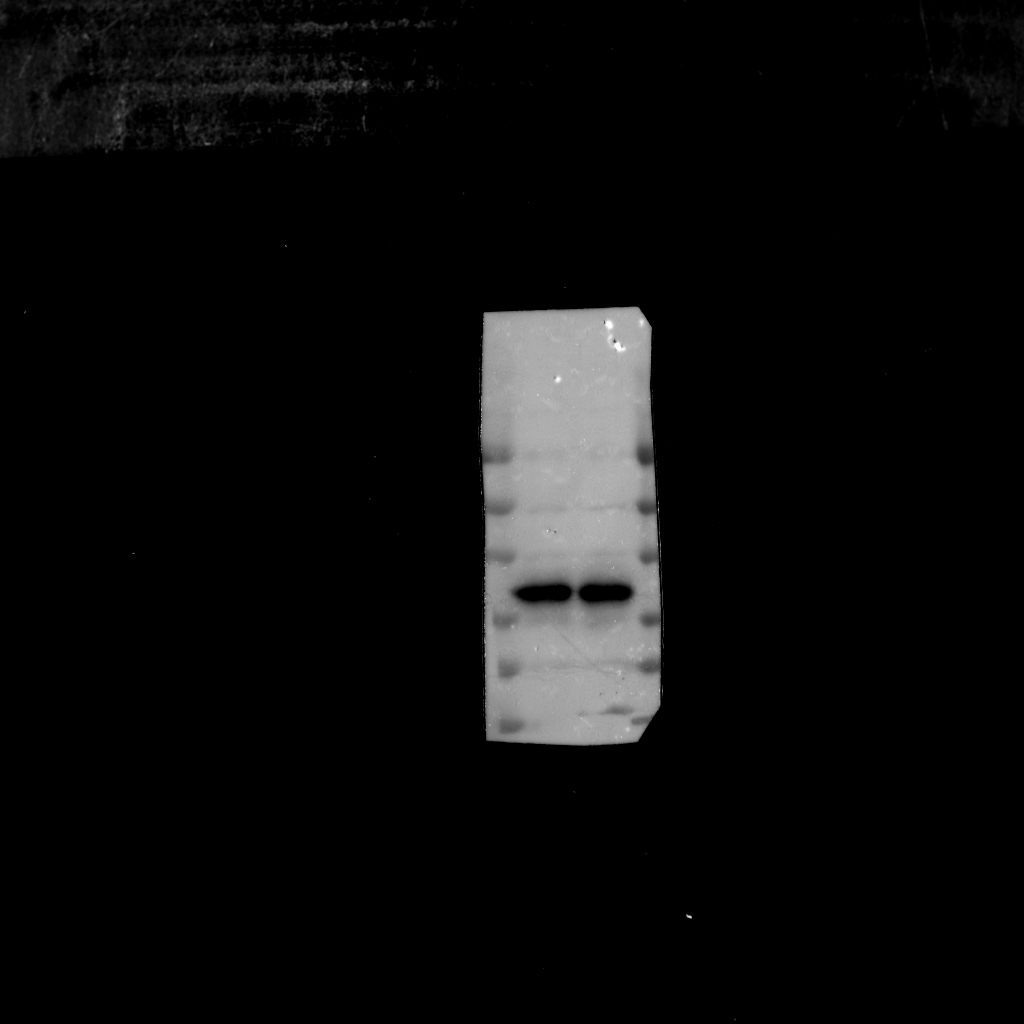

Supplement: Supplemental Information 9 — The raw images of tumor size in nude mice, western blot strips of tumor tissue, and corresponding statistical maps. [file peerj-12-18497-s009.zip › Raw tumor data of nude mice/western blot/未命名导出/clec3b 第2张 gapdh.jpg]

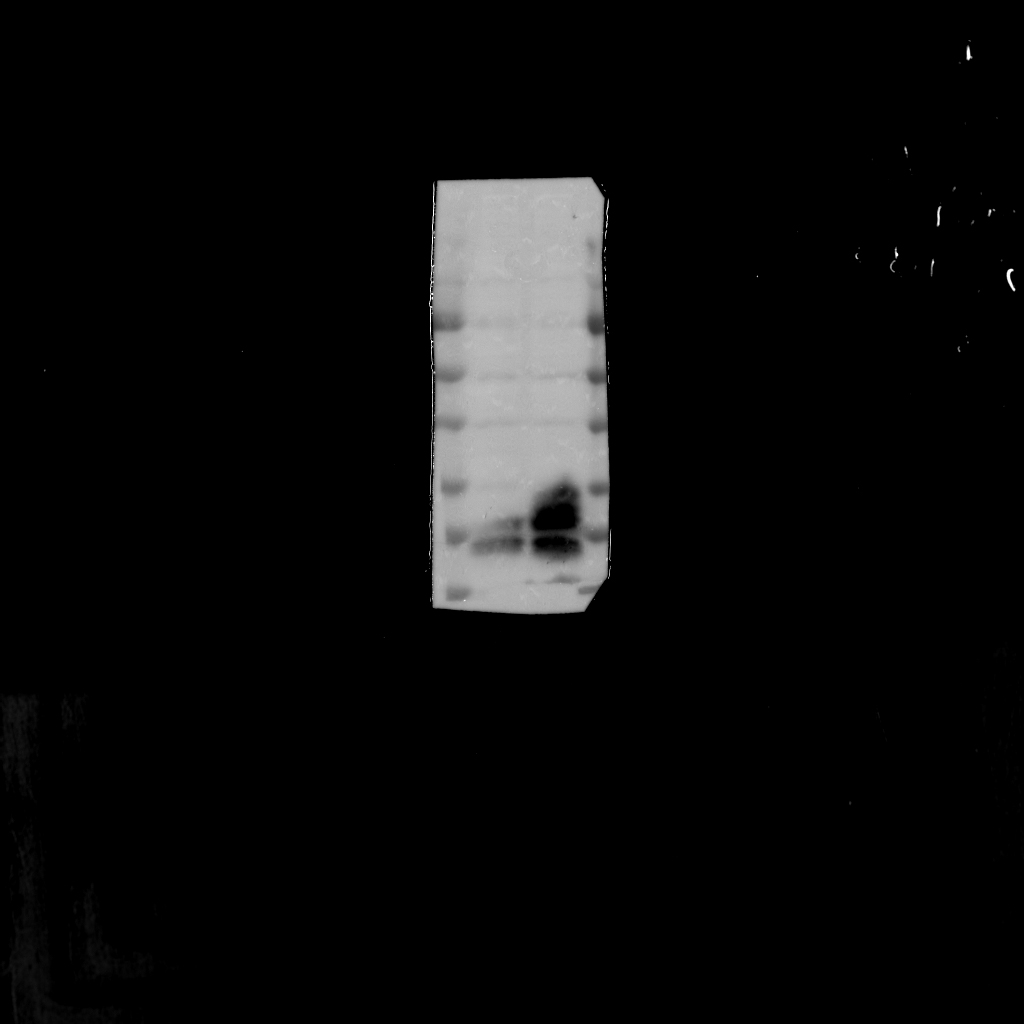

Supplement: Supplemental Information 9 — The raw images of tumor size in nude mice, western blot strips of tumor tissue, and corresponding statistical maps. [file peerj-12-18497-s009.zip › Raw tumor data of nude mice/western blot/未命名导出/clec3b 第2张.jpg]

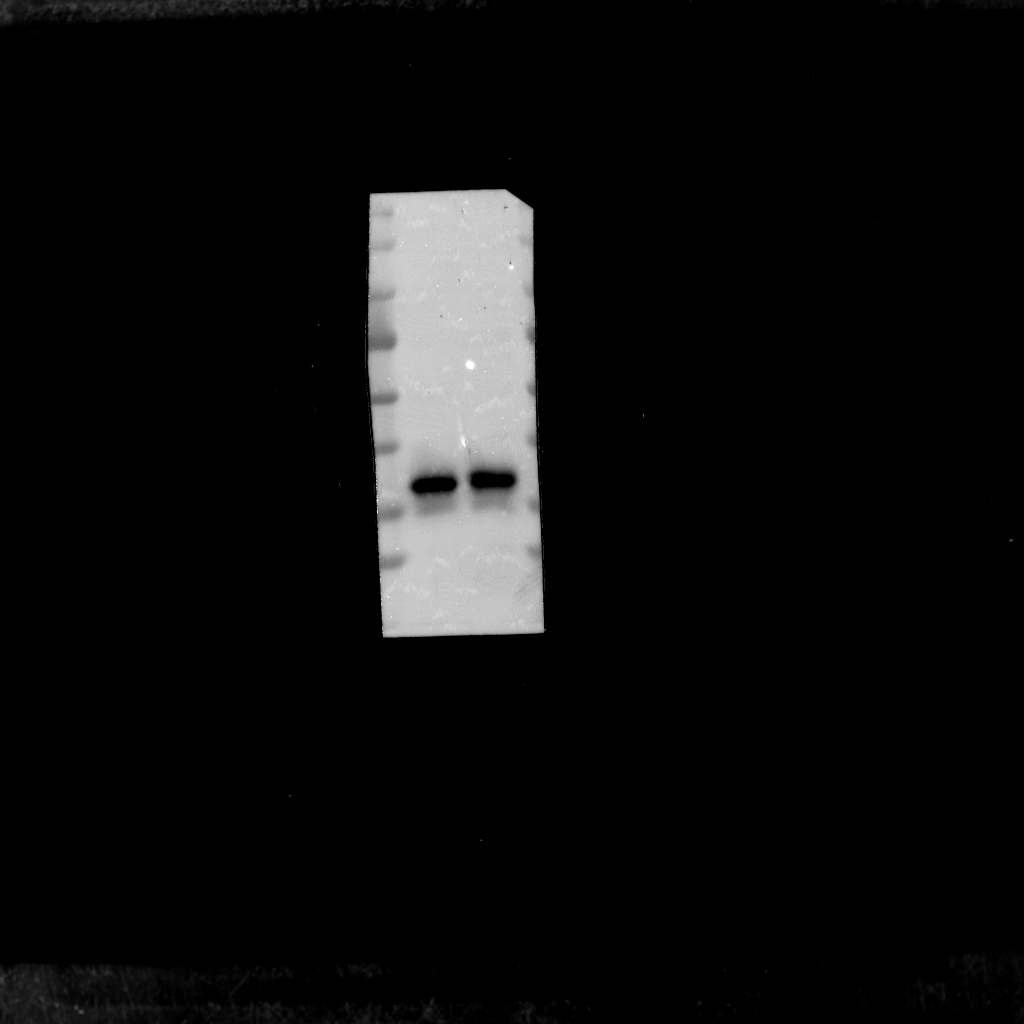

Supplement: Supplemental Information 9 — The raw images of tumor size in nude mice, western blot strips of tumor tissue, and corresponding statistical maps. [file peerj-12-18497-s009.zip › Raw tumor data of nude mice/western blot/未命名导出/clec3b 第3张 gapdh.jpg]

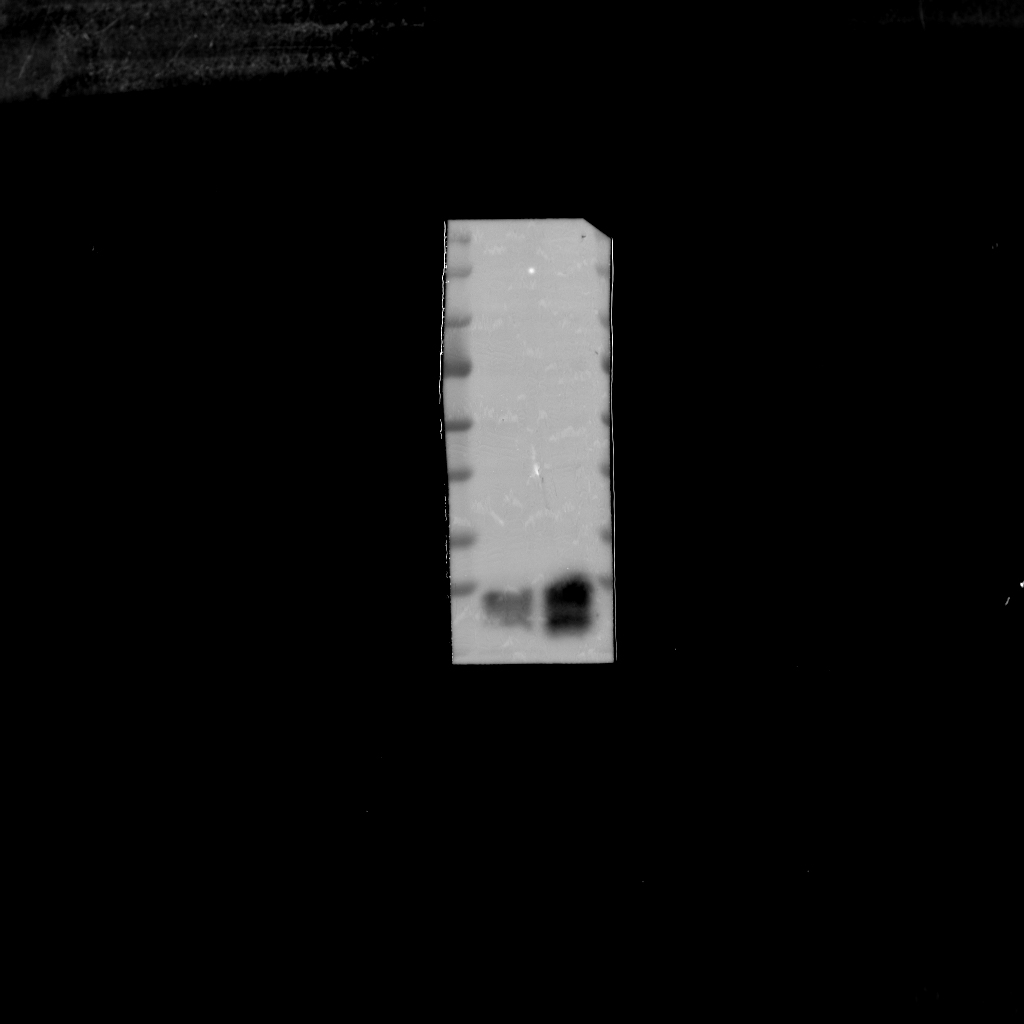

Supplement: Supplemental Information 9 — The raw images of tumor size in nude mice, western blot strips of tumor tissue, and corresponding statistical maps. [file peerj-12-18497-s009.zip › Raw tumor data of nude mice/western blot/未命名导出/clec3b 第3张.jpg]

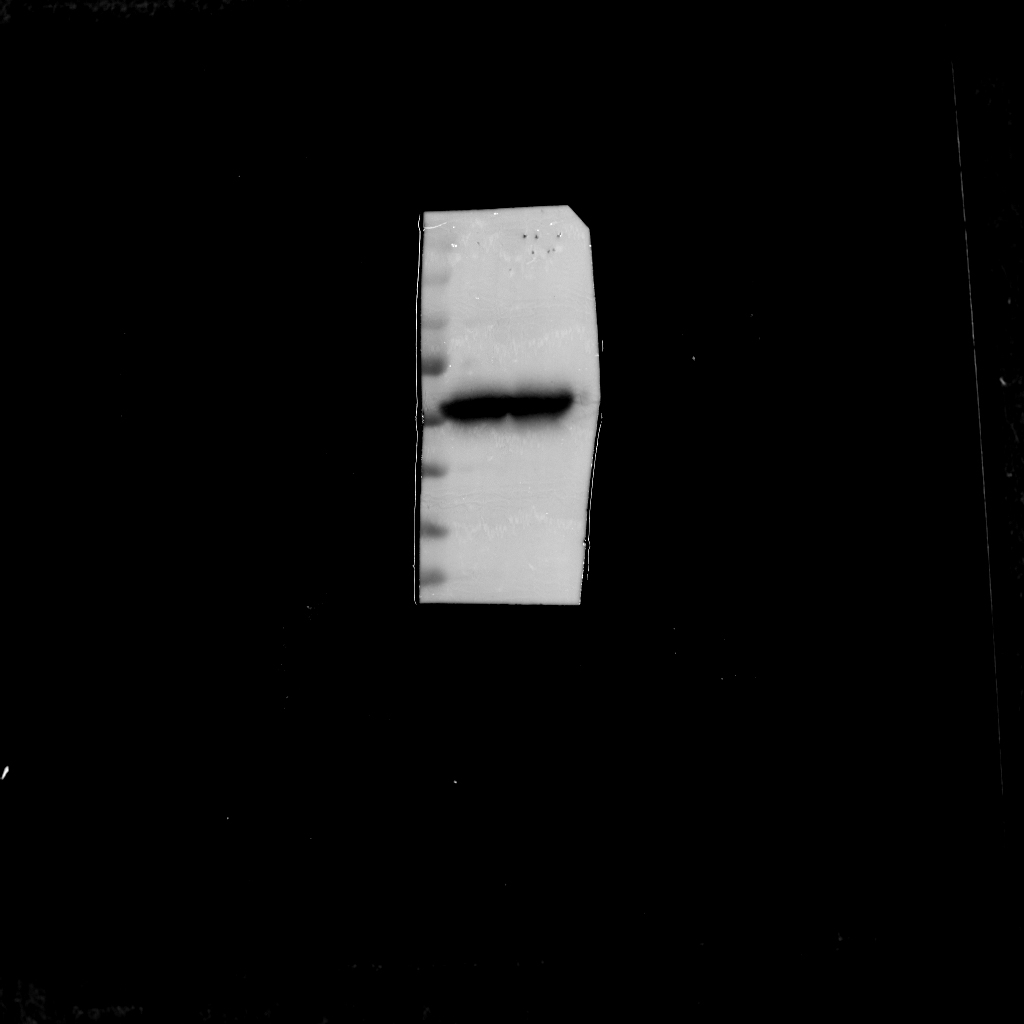

Supplement: Supplemental Information 9 — The raw images of tumor size in nude mice, western blot strips of tumor tissue, and corresponding statistical maps. [file peerj-12-18497-s009.zip › Raw tumor data of nude mice/western blot/未命名导出/cyclind1 第1张 tubulin.jpg]

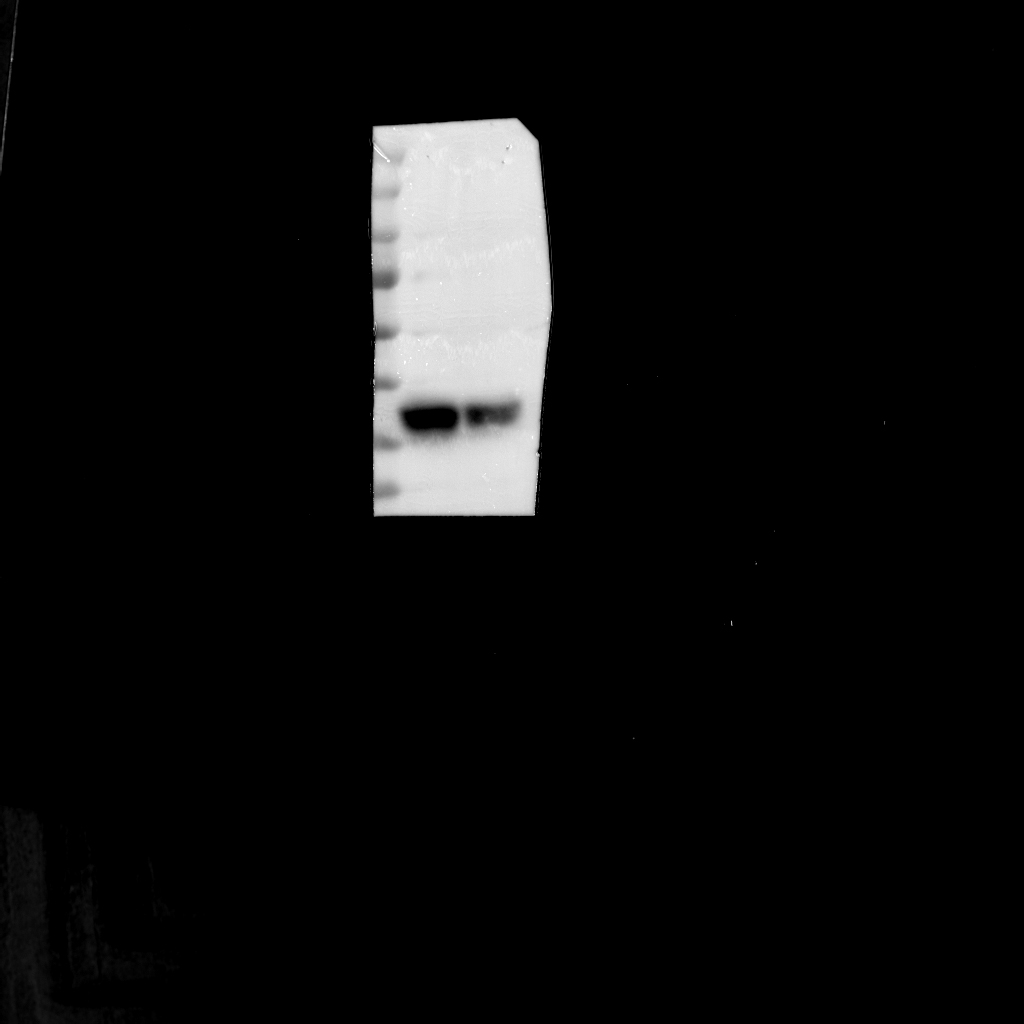

Supplement: Supplemental Information 9 — The raw images of tumor size in nude mice, western blot strips of tumor tissue, and corresponding statistical maps. [file peerj-12-18497-s009.zip › Raw tumor data of nude mice/western blot/未命名导出/cyclind1 第1张.jpg]

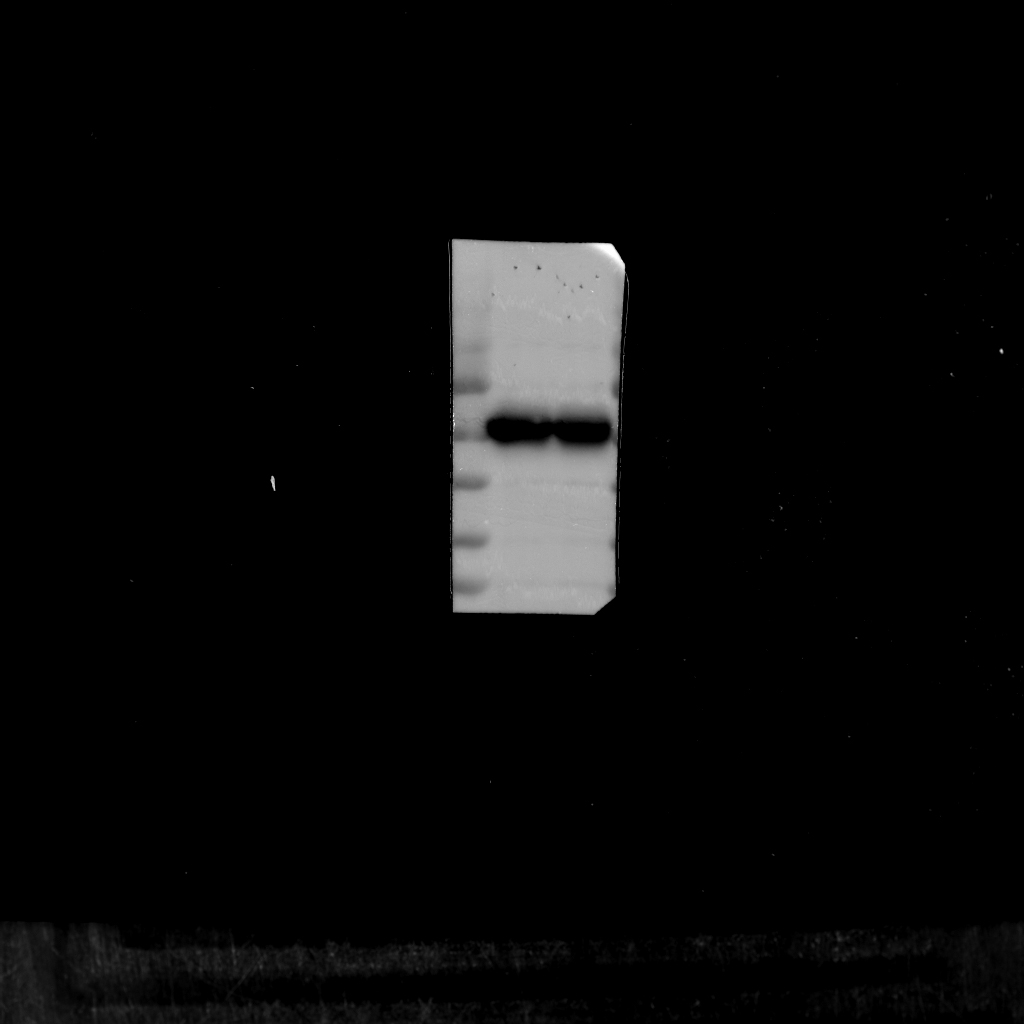

Supplement: Supplemental Information 9 — The raw images of tumor size in nude mice, western blot strips of tumor tissue, and corresponding statistical maps. [file peerj-12-18497-s009.zip › Raw tumor data of nude mice/western blot/未命名导出/cyclind1 第2张 tubulin.jpg]

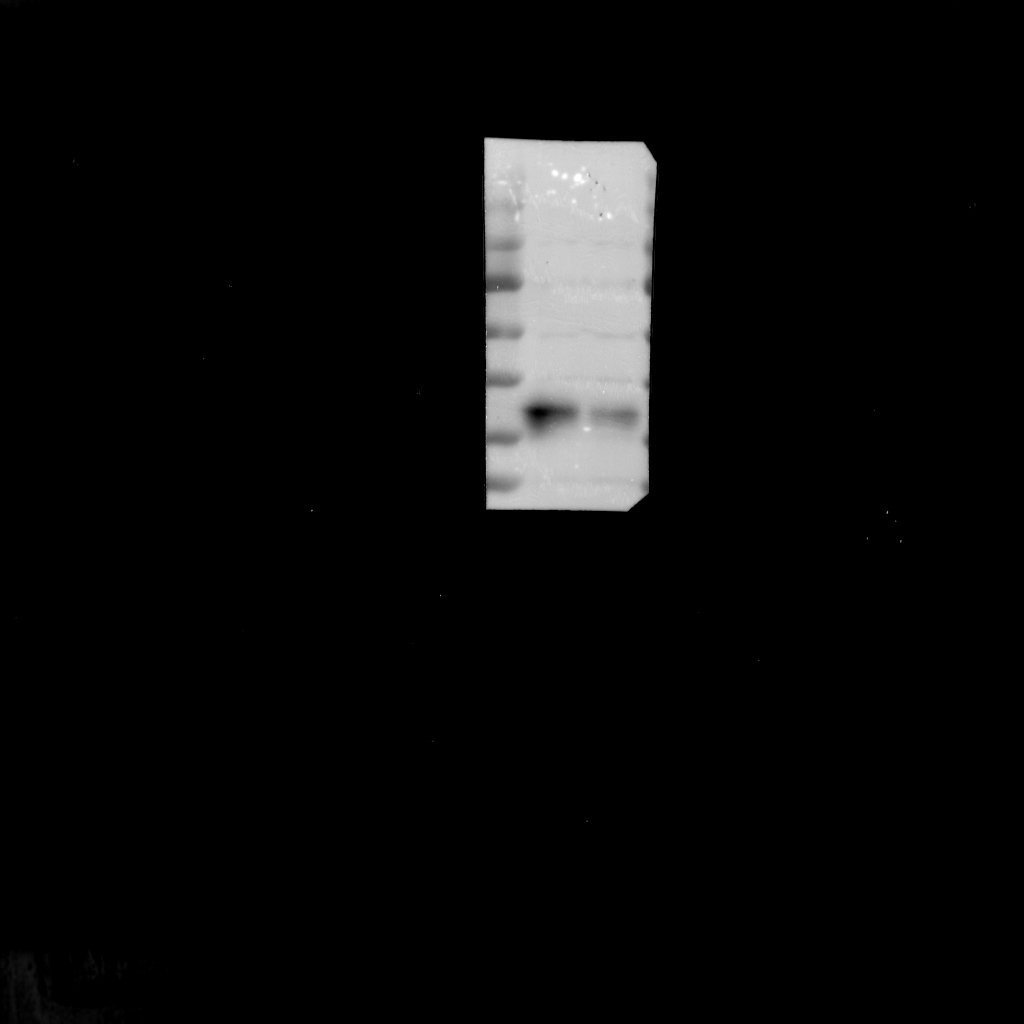

Supplement: Supplemental Information 9 — The raw images of tumor size in nude mice, western blot strips of tumor tissue, and corresponding statistical maps. [file peerj-12-18497-s009.zip › Raw tumor data of nude mice/western blot/未命名导出/cyclind1 第2张.jpg]

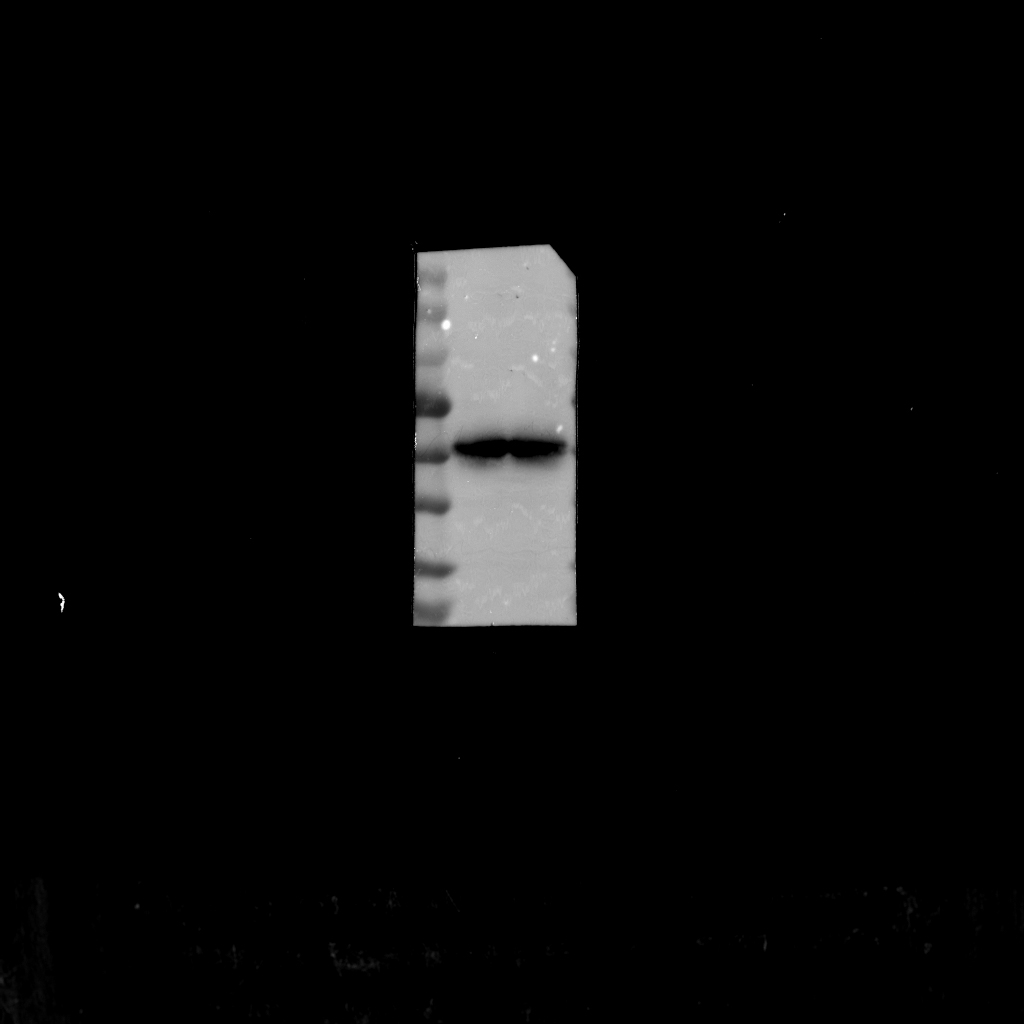

Supplement: Supplemental Information 9 — The raw images of tumor size in nude mice, western blot strips of tumor tissue, and corresponding statistical maps. [file peerj-12-18497-s009.zip › Raw tumor data of nude mice/western blot/未命名导出/cyclind1 第3张 tubulin.jpg]

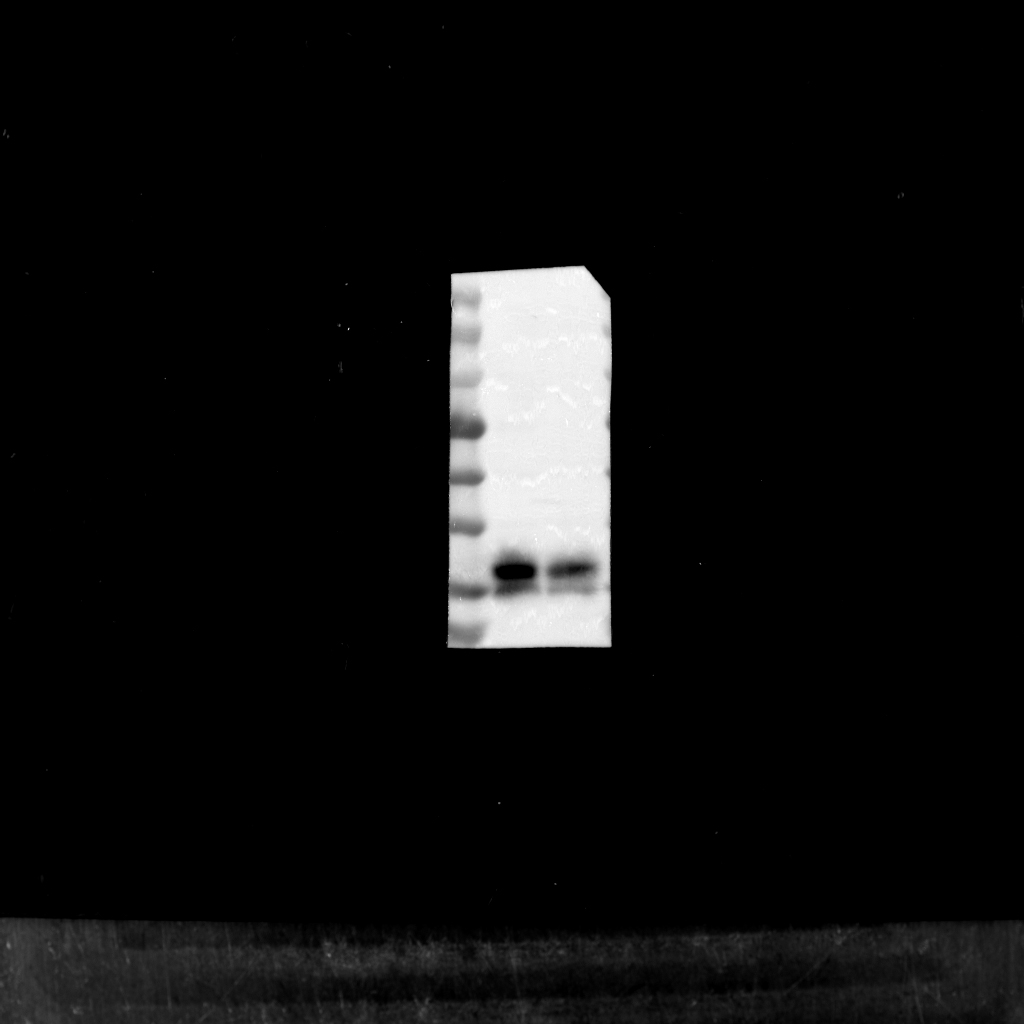

Supplement: Supplemental Information 9 — The raw images of tumor size in nude mice, western blot strips of tumor tissue, and corresponding statistical maps. [file peerj-12-18497-s009.zip › Raw tumor data of nude mice/western blot/未命名导出/cyclind1 第3张.jpg]

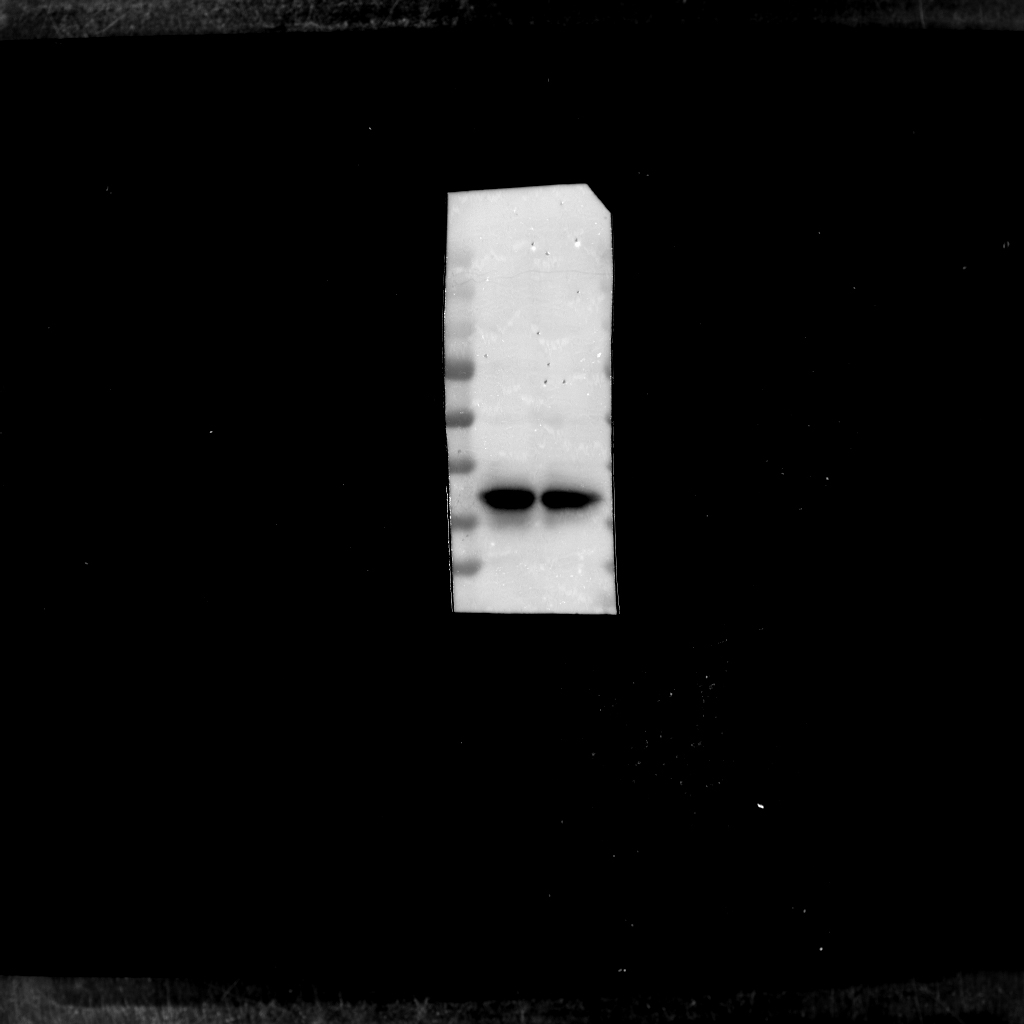

Supplement: Supplemental Information 9 — The raw images of tumor size in nude mice, western blot strips of tumor tissue, and corresponding statistical maps. [file peerj-12-18497-s009.zip › Raw tumor data of nude mice/western blot/未命名导出/e-cad 第1张 gapdh.jpg]

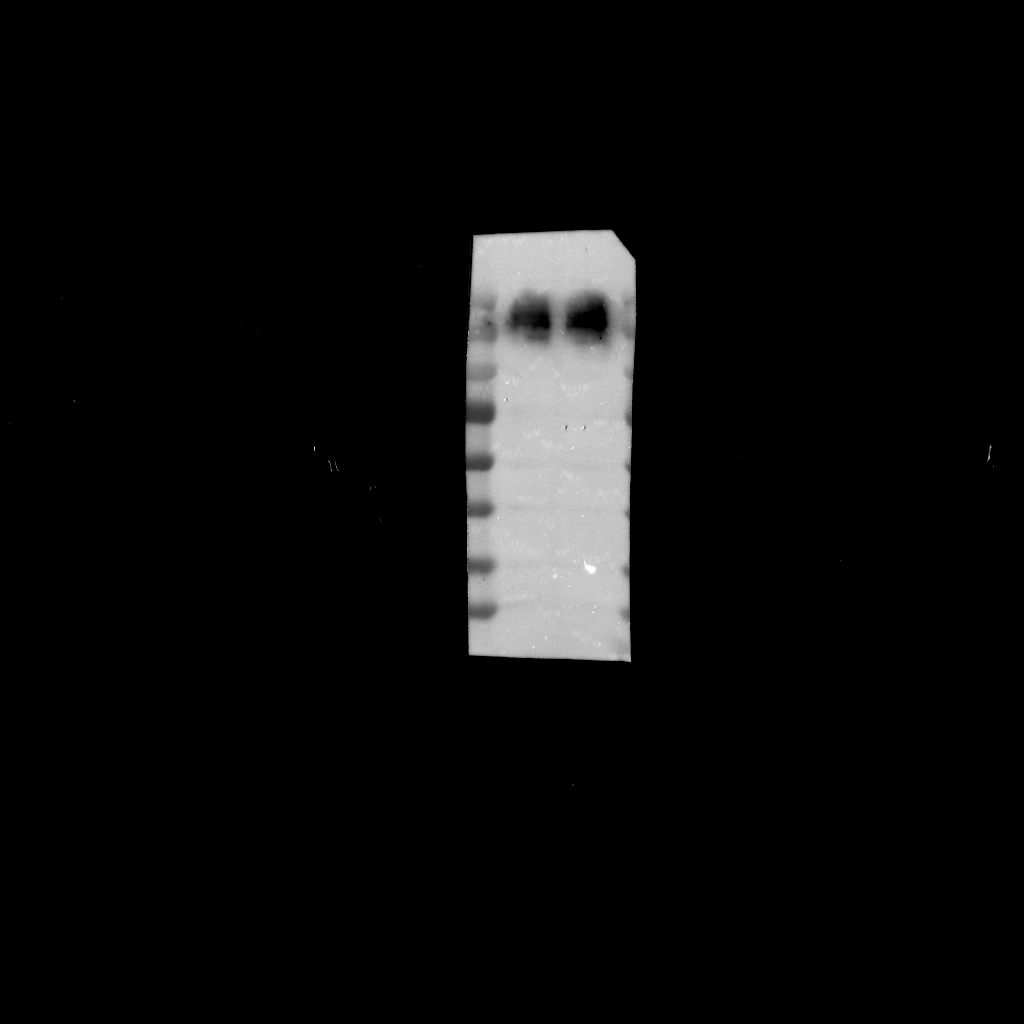

Supplement: Supplemental Information 9 — The raw images of tumor size in nude mice, western blot strips of tumor tissue, and corresponding statistical maps. [file peerj-12-18497-s009.zip › Raw tumor data of nude mice/western blot/未命名导出/e-cad 第1张.jpg]

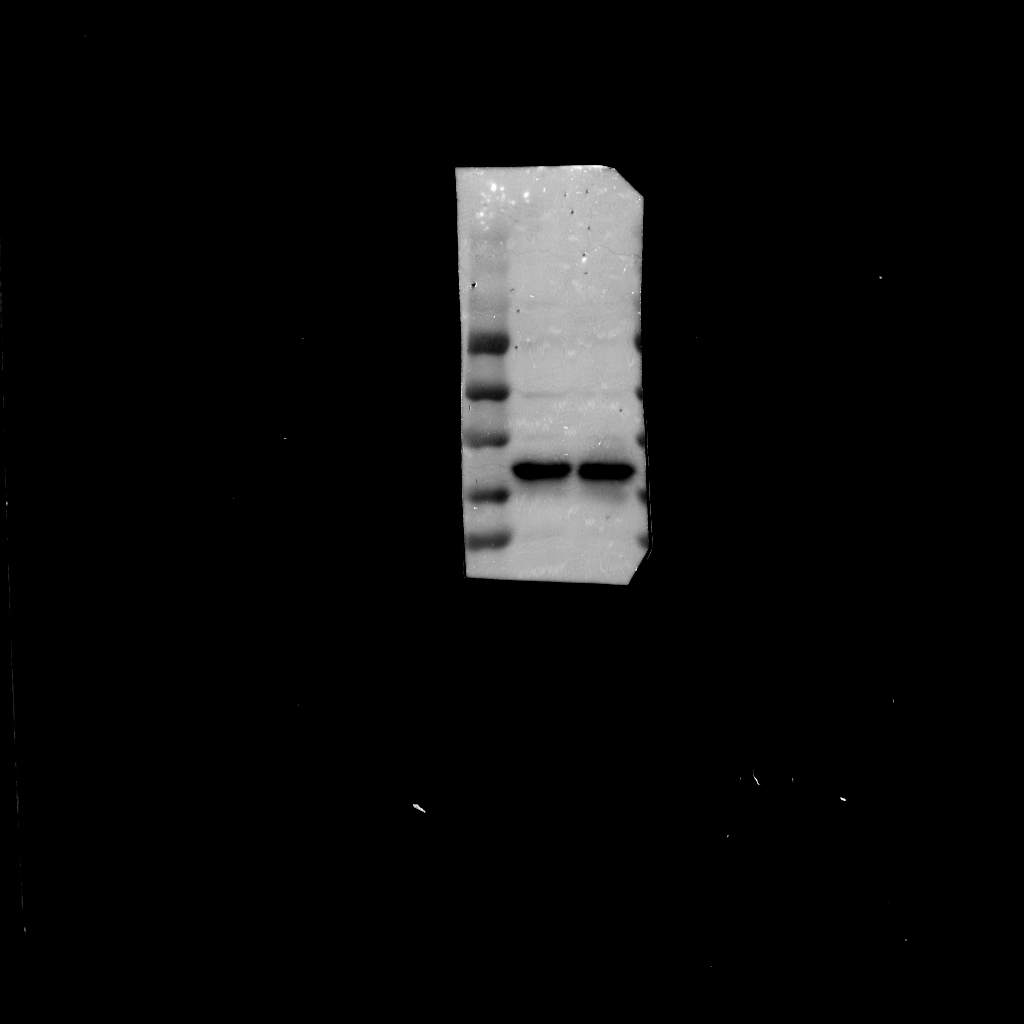

Supplement: Supplemental Information 9 — The raw images of tumor size in nude mice, western blot strips of tumor tissue, and corresponding statistical maps. [file peerj-12-18497-s009.zip › Raw tumor data of nude mice/western blot/未命名导出/e-cad 第2张 gapdh.jpg]

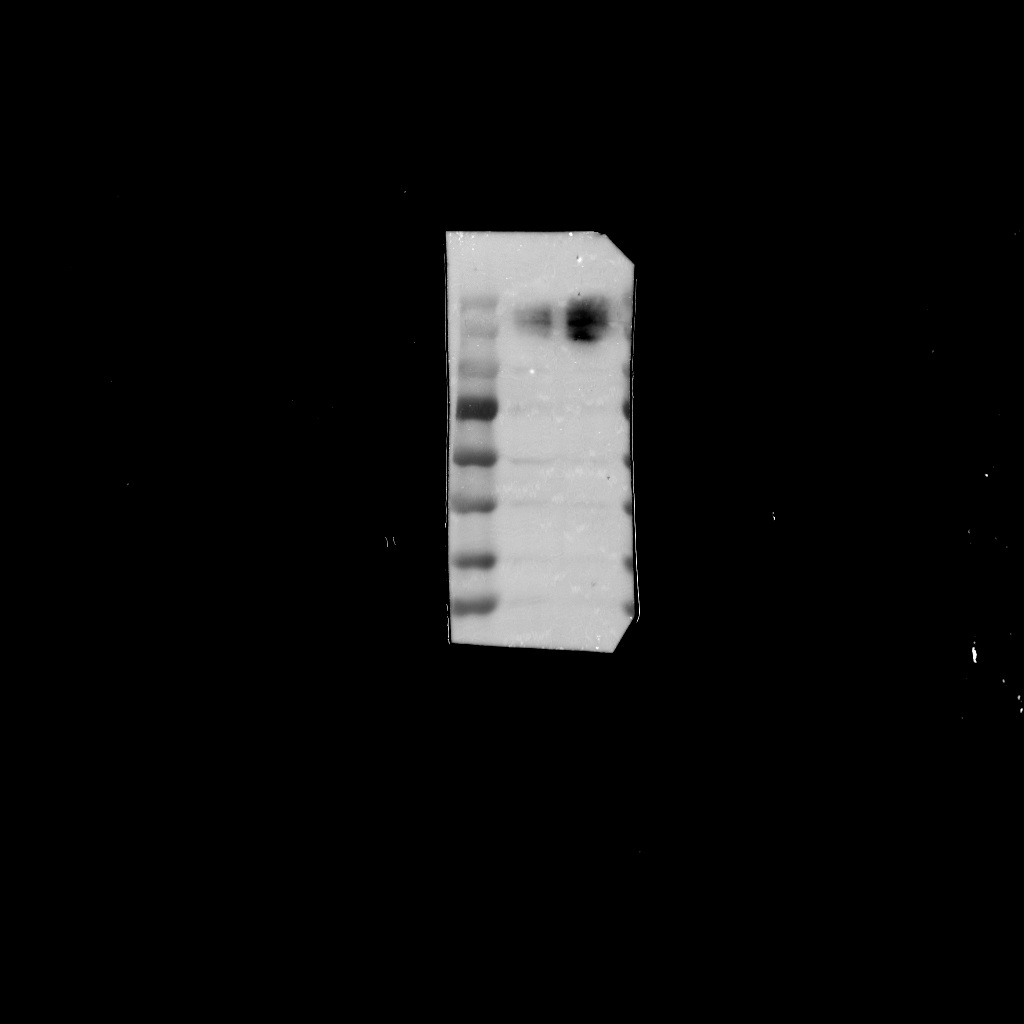

Supplement: Supplemental Information 9 — The raw images of tumor size in nude mice, western blot strips of tumor tissue, and corresponding statistical maps. [file peerj-12-18497-s009.zip › Raw tumor data of nude mice/western blot/未命名导出/e-cad 第2张.jpg]

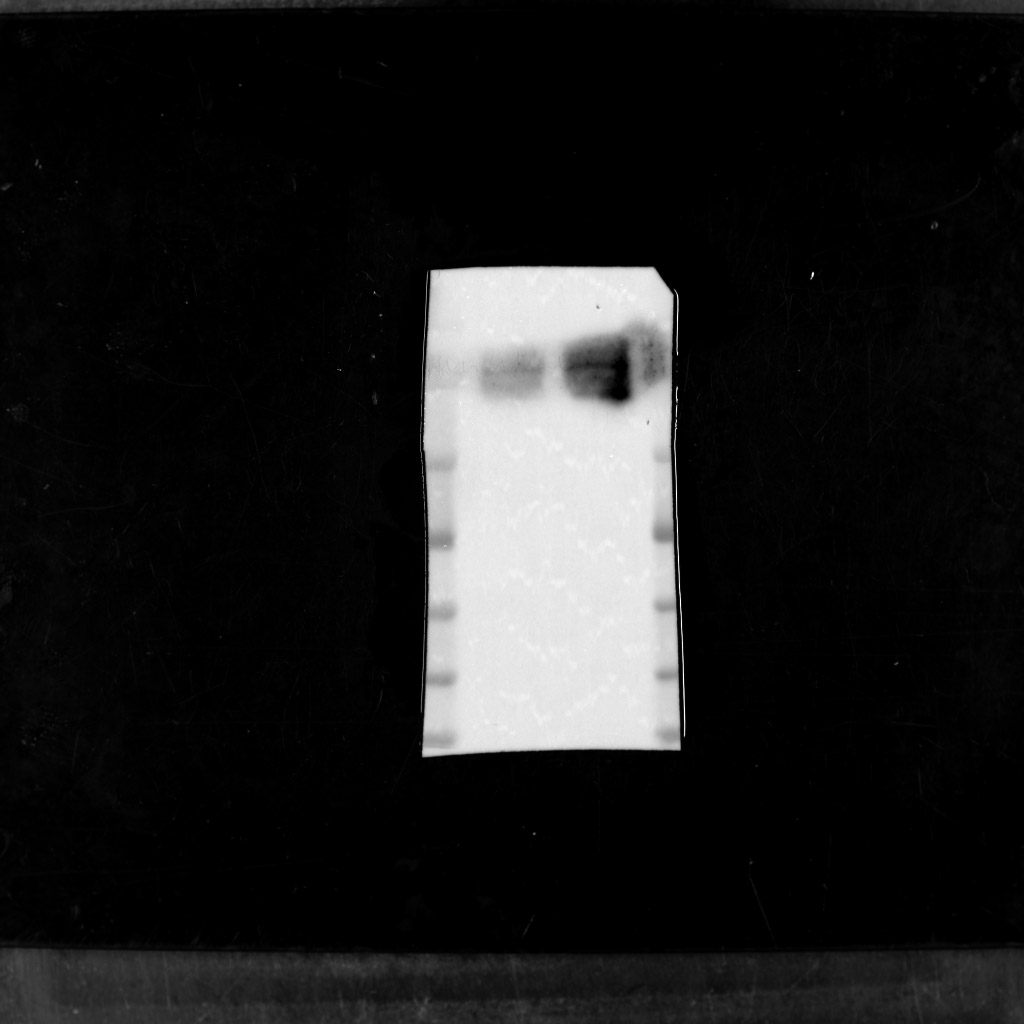

Supplement: Supplemental Information 9 — The raw images of tumor size in nude mice, western blot strips of tumor tissue, and corresponding statistical maps. [file peerj-12-18497-s009.zip › Raw tumor data of nude mice/western blot/未命名导出/e-cad 第3张.jpg]

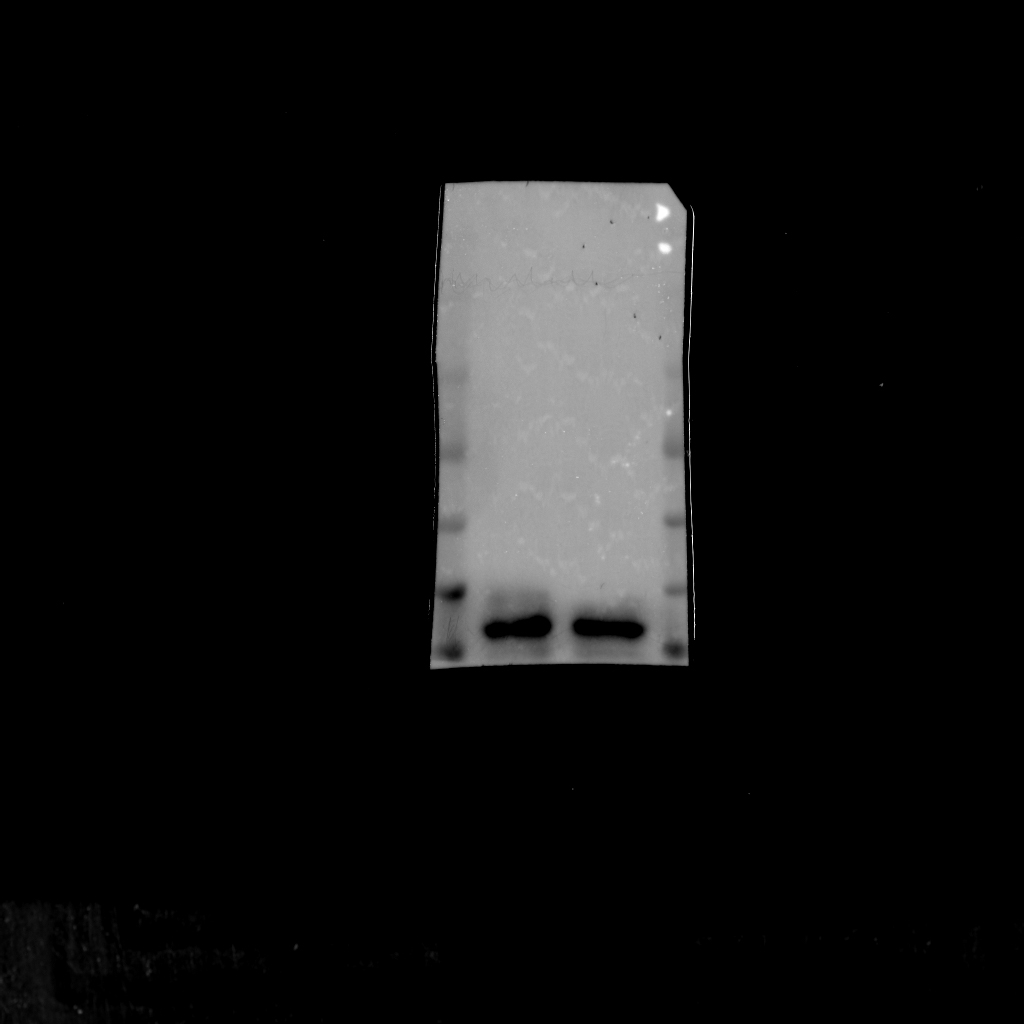

Supplement: Supplemental Information 9 — The raw images of tumor size in nude mice, western blot strips of tumor tissue, and corresponding statistical maps. [file peerj-12-18497-s009.zip › Raw tumor data of nude mice/western blot/未命名导出/e-cad 第3张gapdh.jpg]

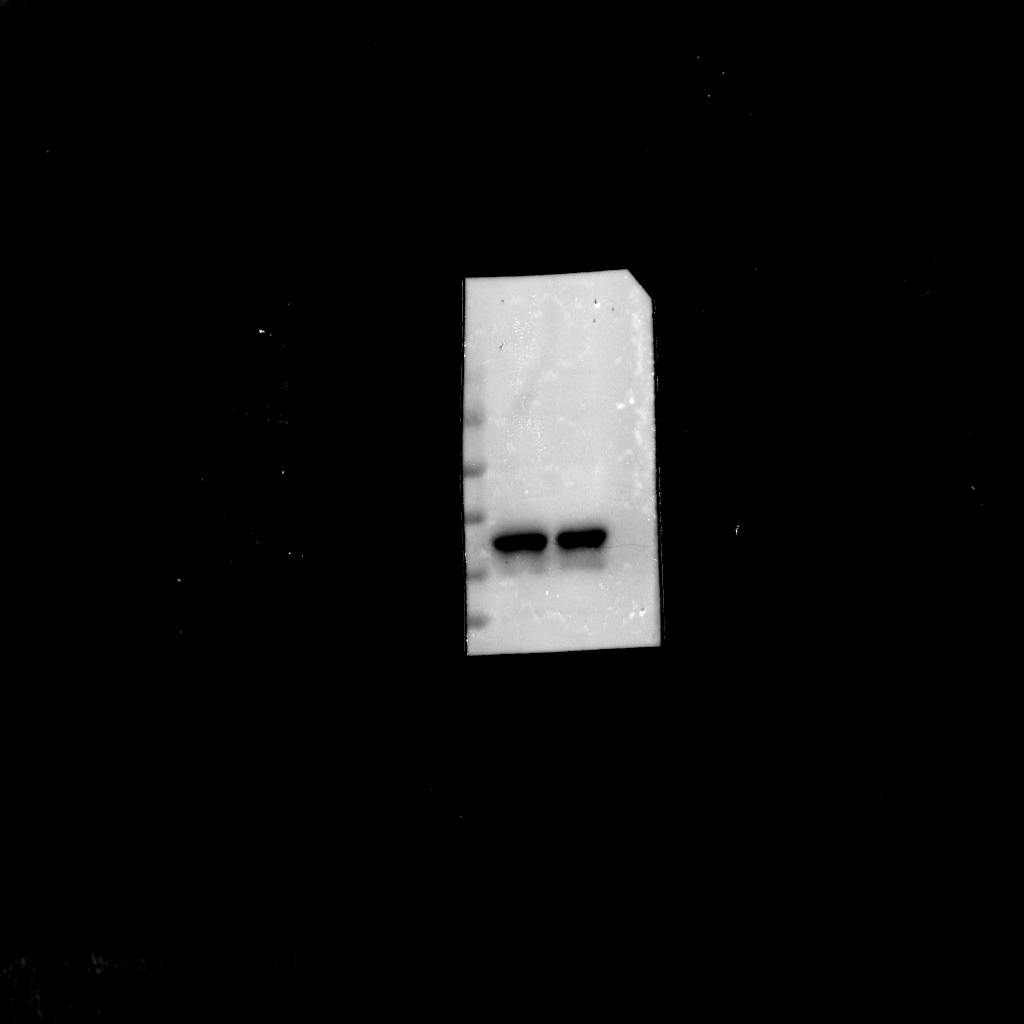

Supplement: Supplemental Information 9 — The raw images of tumor size in nude mice, western blot strips of tumor tissue, and corresponding statistical maps. [file peerj-12-18497-s009.zip › Raw tumor data of nude mice/western blot/未命名导出/gsk3beita 第1张 gapdh.jpg]

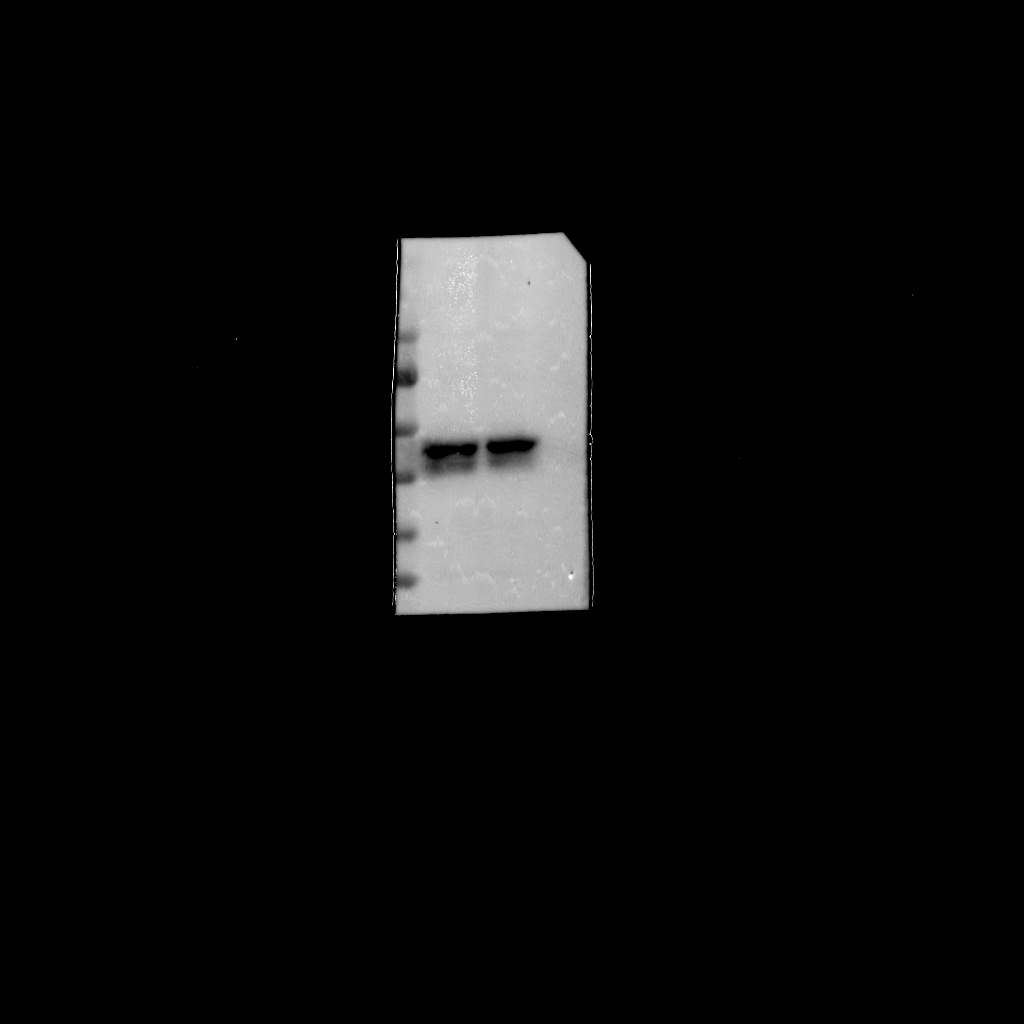

Supplement: Supplemental Information 9 — The raw images of tumor size in nude mice, western blot strips of tumor tissue, and corresponding statistical maps. [file peerj-12-18497-s009.zip › Raw tumor data of nude mice/western blot/未命名导出/gsk3beita 第1张.jpg]

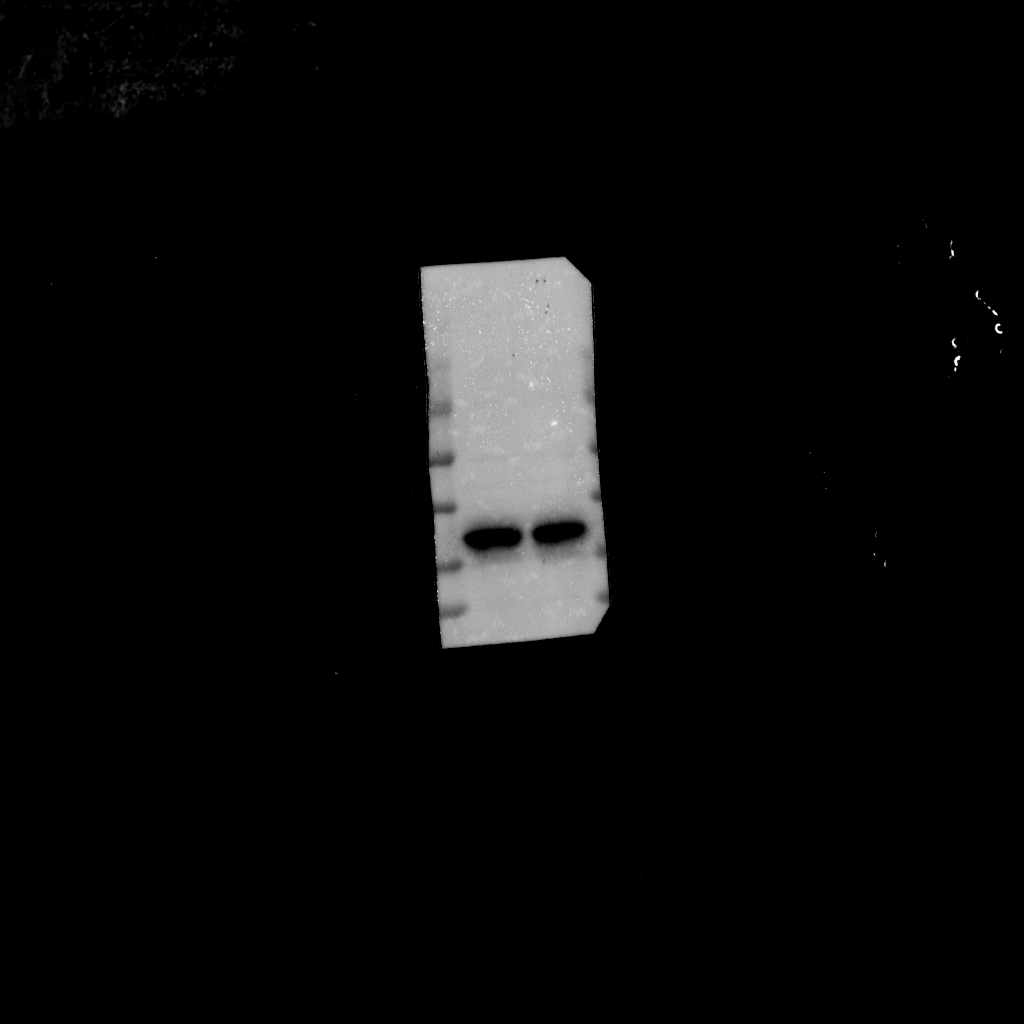

Supplement: Supplemental Information 9 — The raw images of tumor size in nude mice, western blot strips of tumor tissue, and corresponding statistical maps. [file peerj-12-18497-s009.zip › Raw tumor data of nude mice/western blot/未命名导出/gsk3beita 第2张 gapdh.jpg]

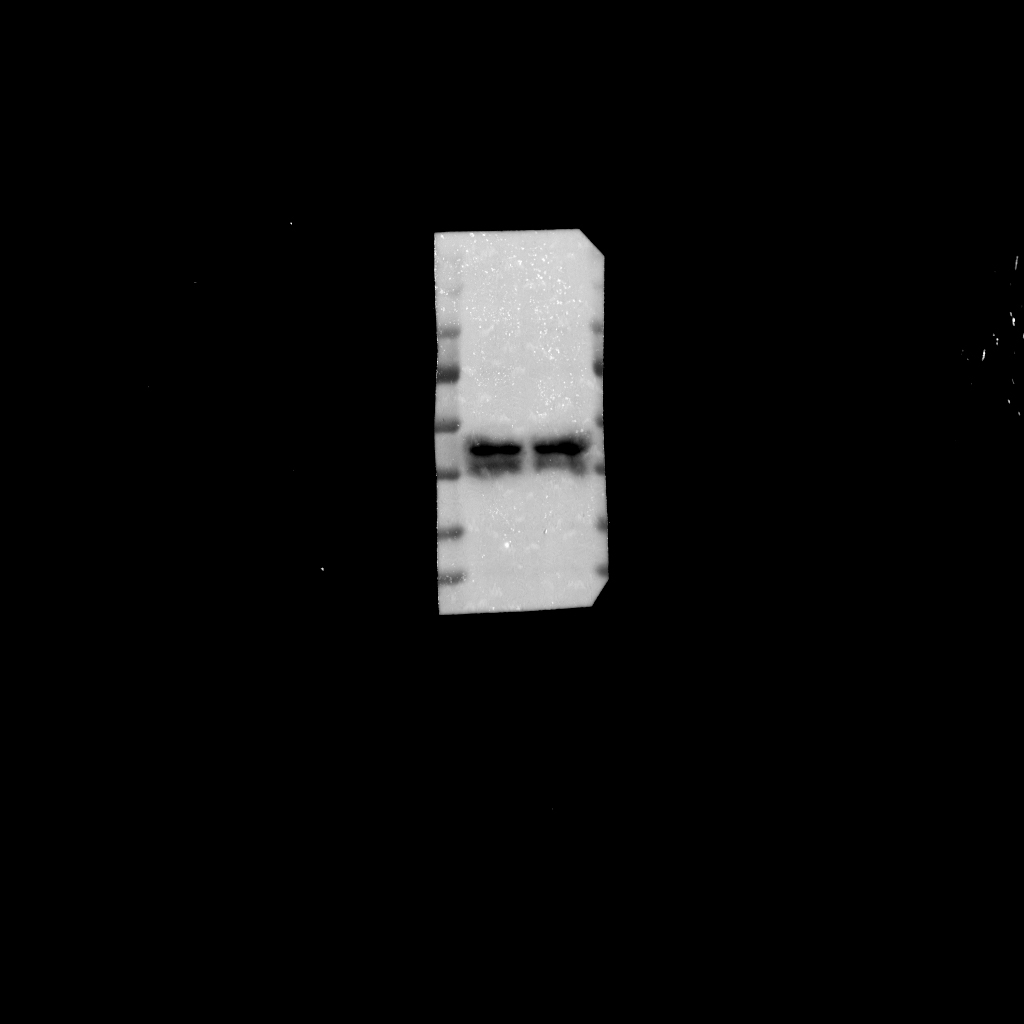

Supplement: Supplemental Information 9 — The raw images of tumor size in nude mice, western blot strips of tumor tissue, and corresponding statistical maps. [file peerj-12-18497-s009.zip › Raw tumor data of nude mice/western blot/未命名导出/gsk3beita 第2张.jpg]

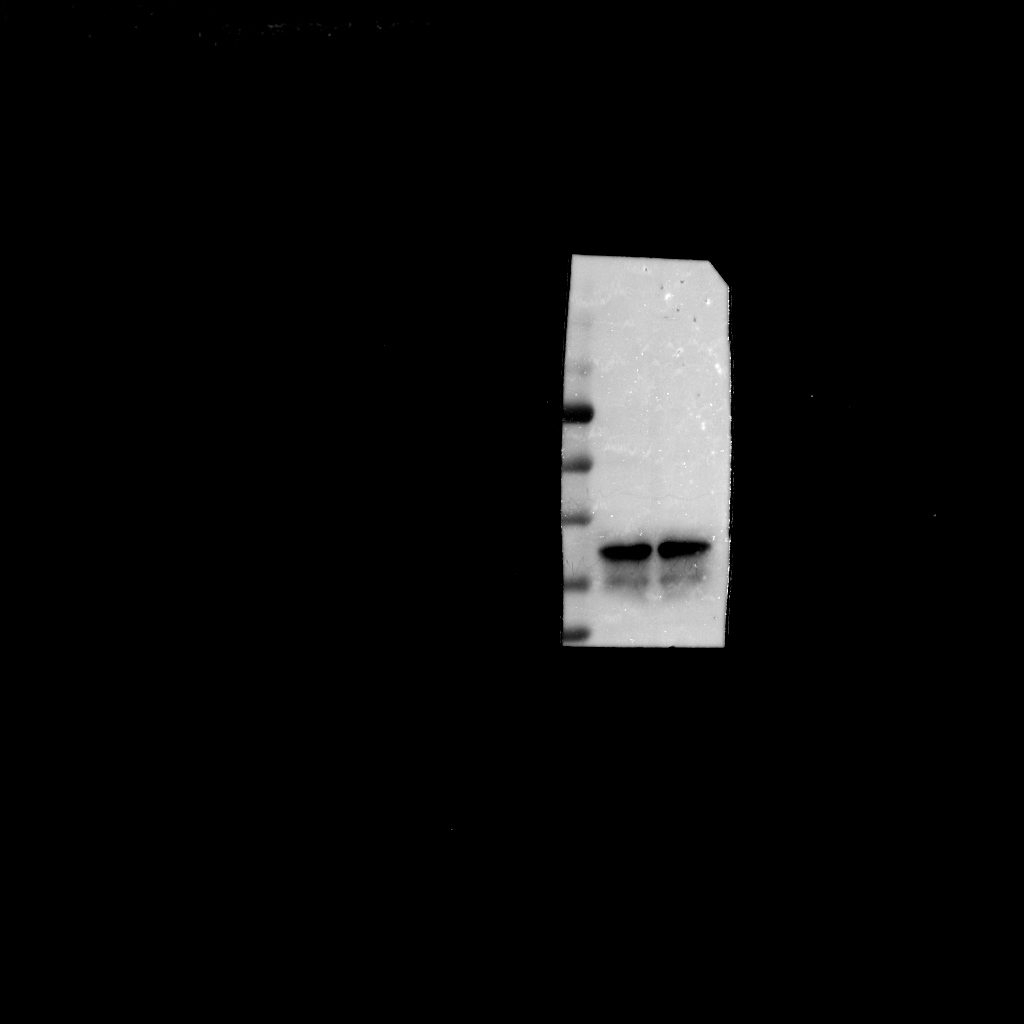

Supplement: Supplemental Information 9 — The raw images of tumor size in nude mice, western blot strips of tumor tissue, and corresponding statistical maps. [file peerj-12-18497-s009.zip › Raw tumor data of nude mice/western blot/未命名导出/gsk3beita 第3张 gapdh.jpg]

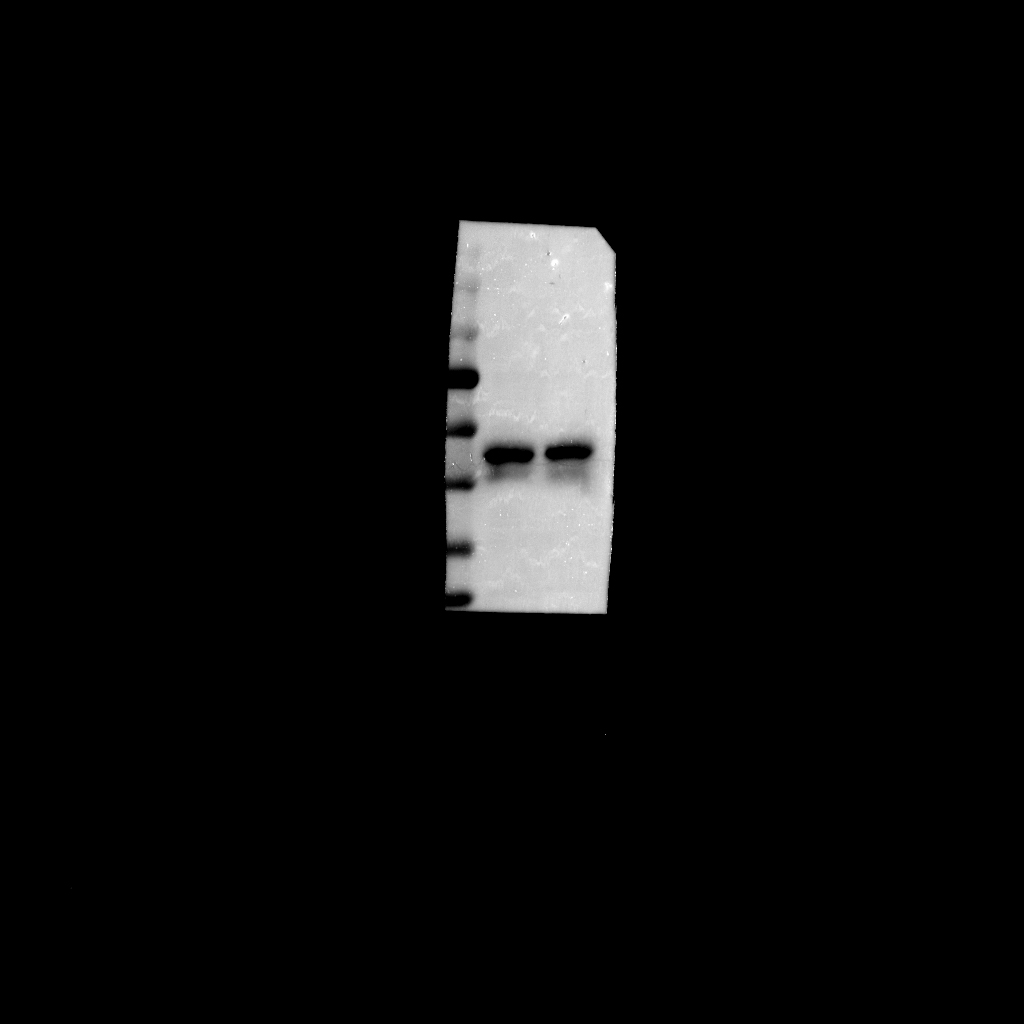

Supplement: Supplemental Information 9 — The raw images of tumor size in nude mice, western blot strips of tumor tissue, and corresponding statistical maps. [file peerj-12-18497-s009.zip › Raw tumor data of nude mice/western blot/未命名导出/gsk3beita 第3张.jpg]

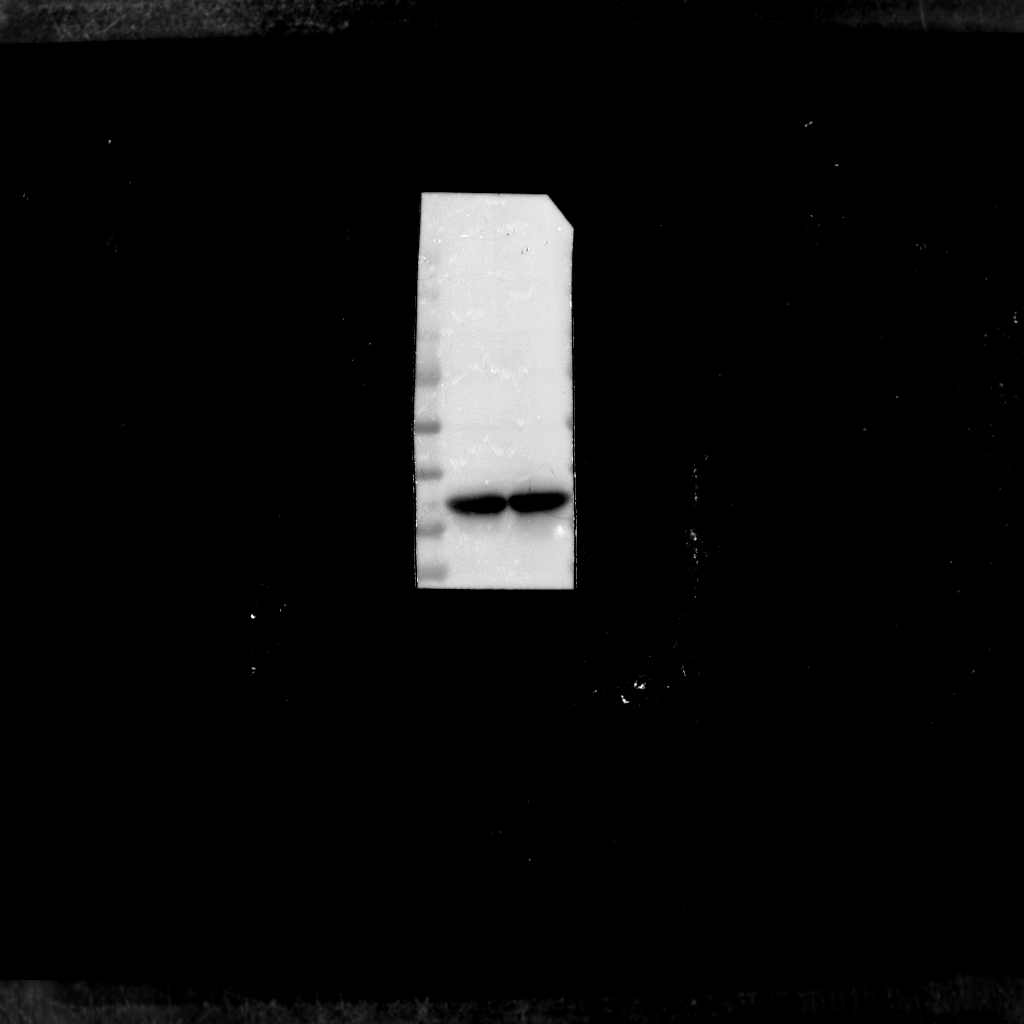

Supplement: Supplemental Information 9 — The raw images of tumor size in nude mice, western blot strips of tumor tissue, and corresponding statistical maps. [file peerj-12-18497-s009.zip › Raw tumor data of nude mice/western blot/未命名导出/n-cad 第1张 gapdh.jpg]

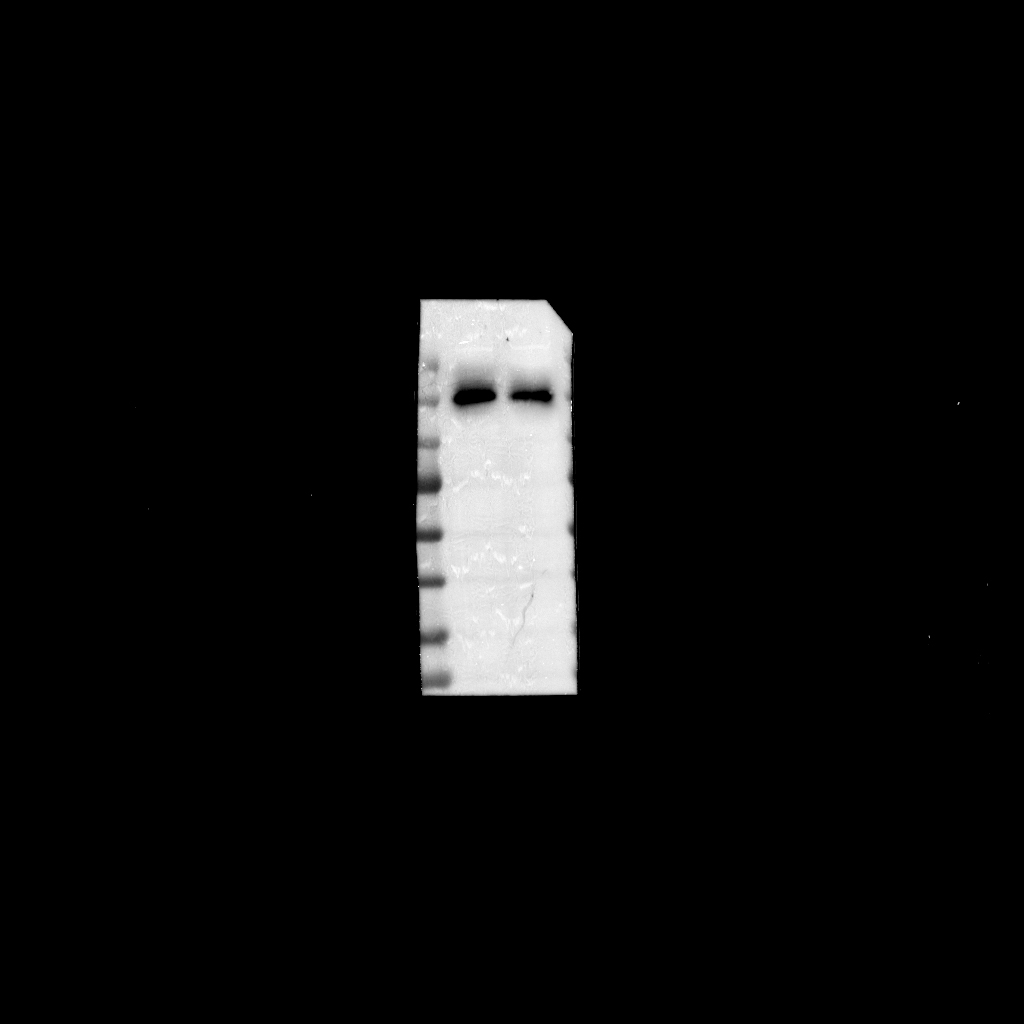

Supplement: Supplemental Information 9 — The raw images of tumor size in nude mice, western blot strips of tumor tissue, and corresponding statistical maps. [file peerj-12-18497-s009.zip › Raw tumor data of nude mice/western blot/未命名导出/n-cad 第1张.jpg]

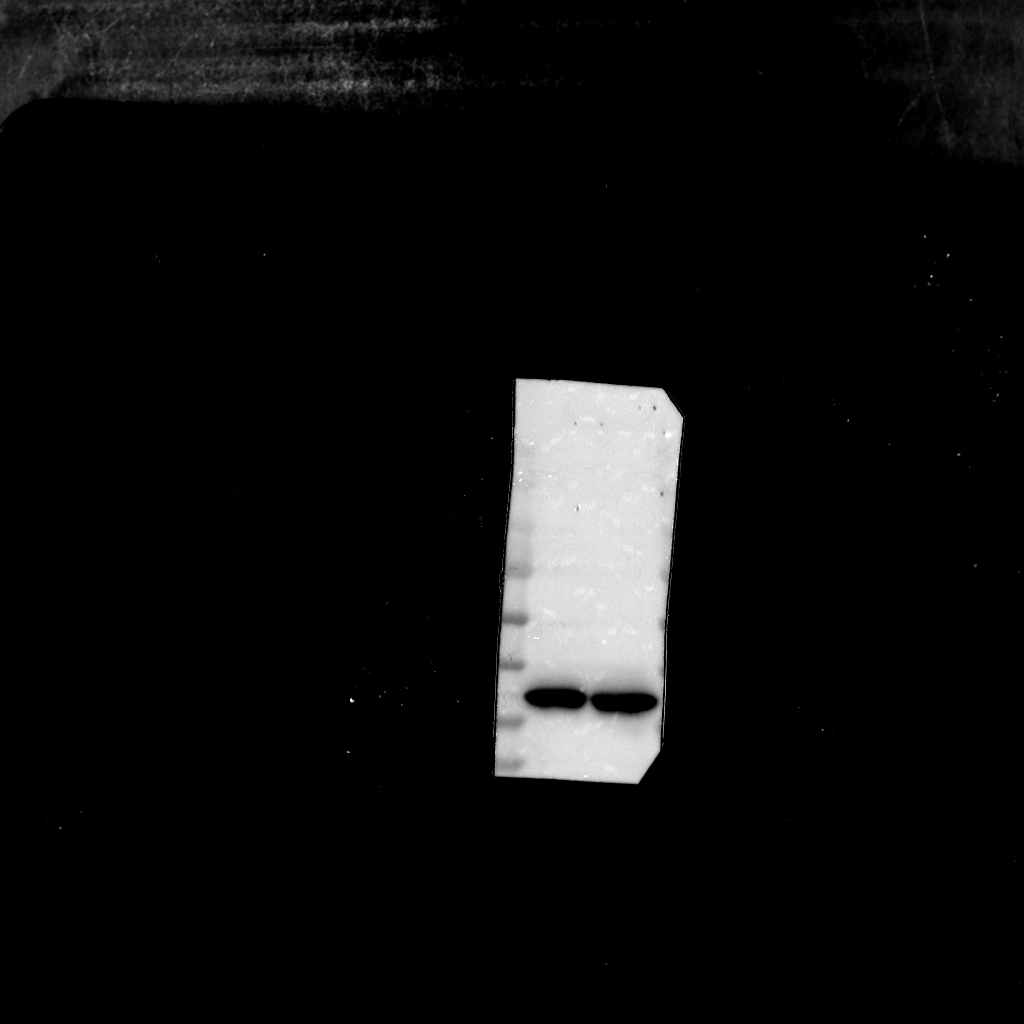

Supplement: Supplemental Information 9 — The raw images of tumor size in nude mice, western blot strips of tumor tissue, and corresponding statistical maps. [file peerj-12-18497-s009.zip › Raw tumor data of nude mice/western blot/未命名导出/n-cad 第2张 gapdh.jpg]

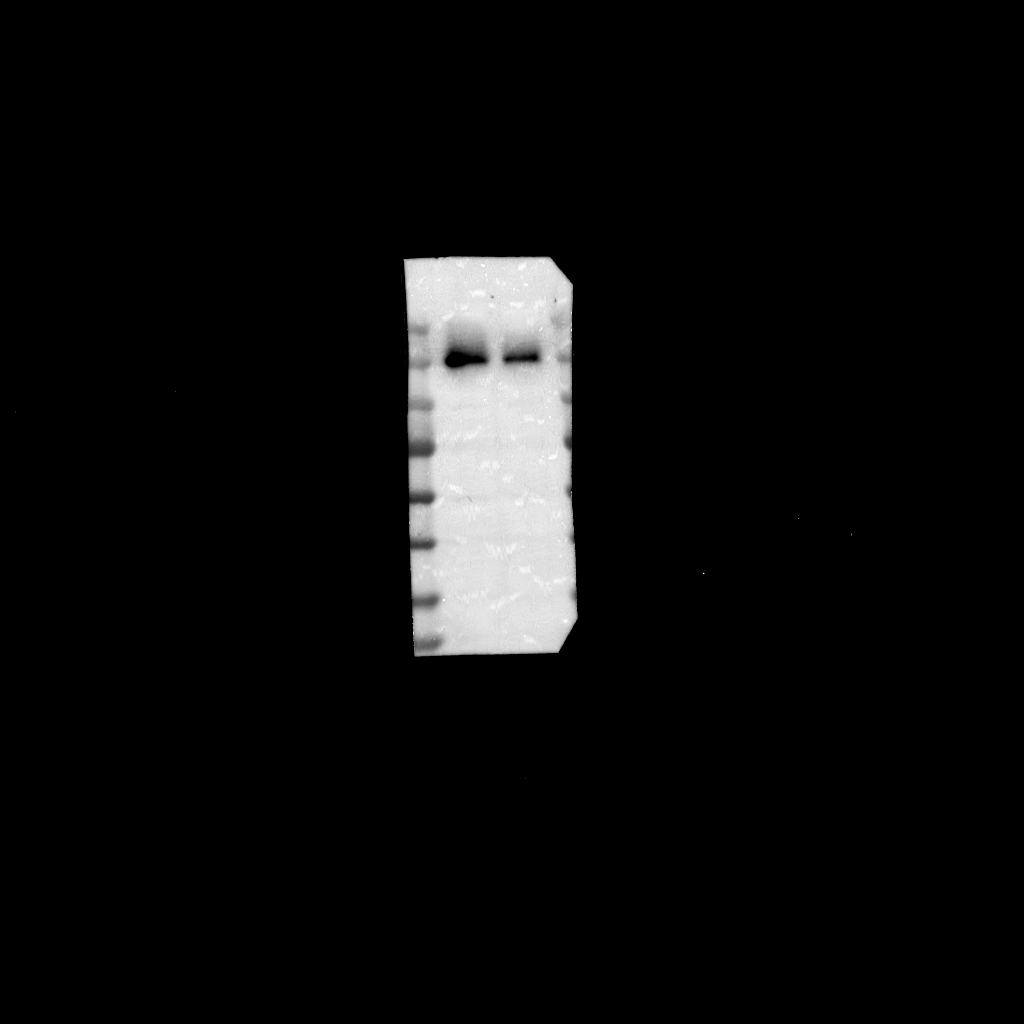

Supplement: Supplemental Information 9 — The raw images of tumor size in nude mice, western blot strips of tumor tissue, and corresponding statistical maps. [file peerj-12-18497-s009.zip › Raw tumor data of nude mice/western blot/未命名导出/n-cad 第2张.jpg]

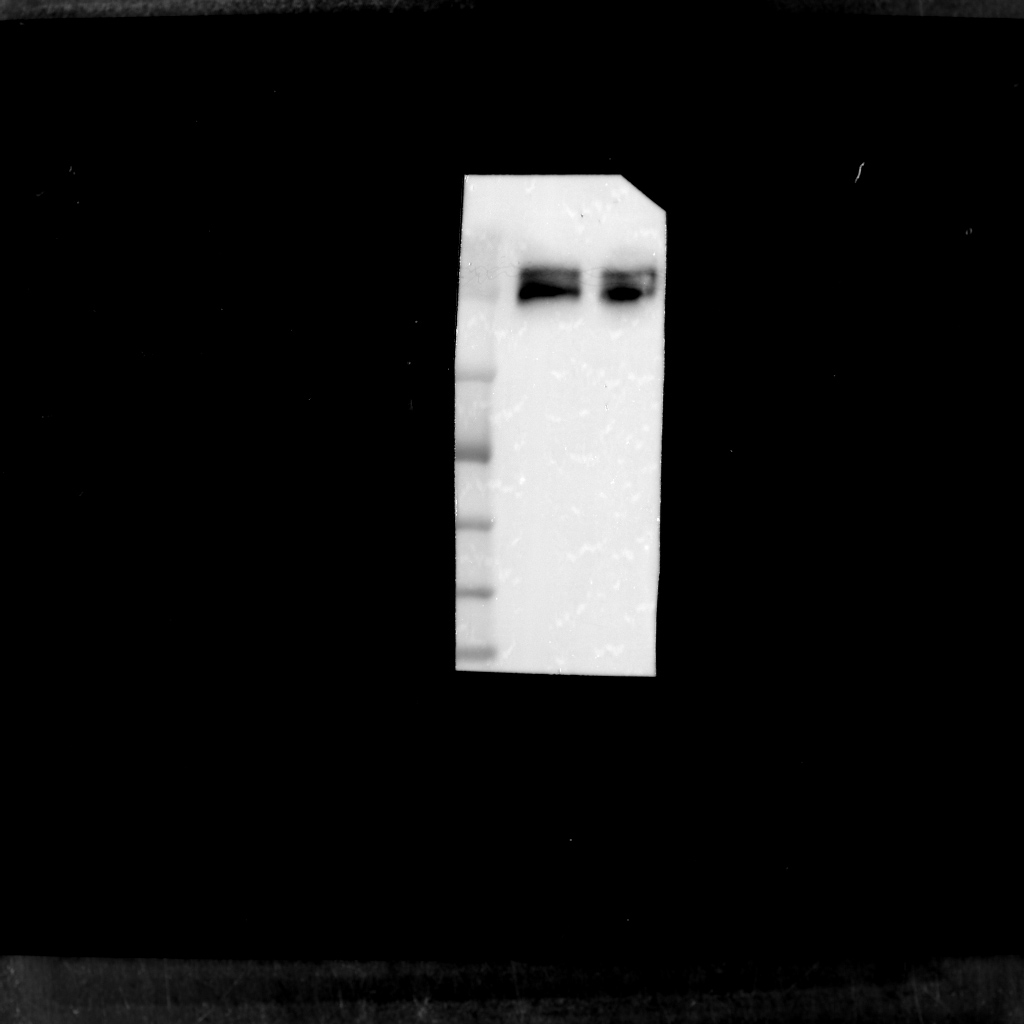

Supplement: Supplemental Information 9 — The raw images of tumor size in nude mice, western blot strips of tumor tissue, and corresponding statistical maps. [file peerj-12-18497-s009.zip › Raw tumor data of nude mice/western blot/未命名导出/n-cad 第3张.jpg]

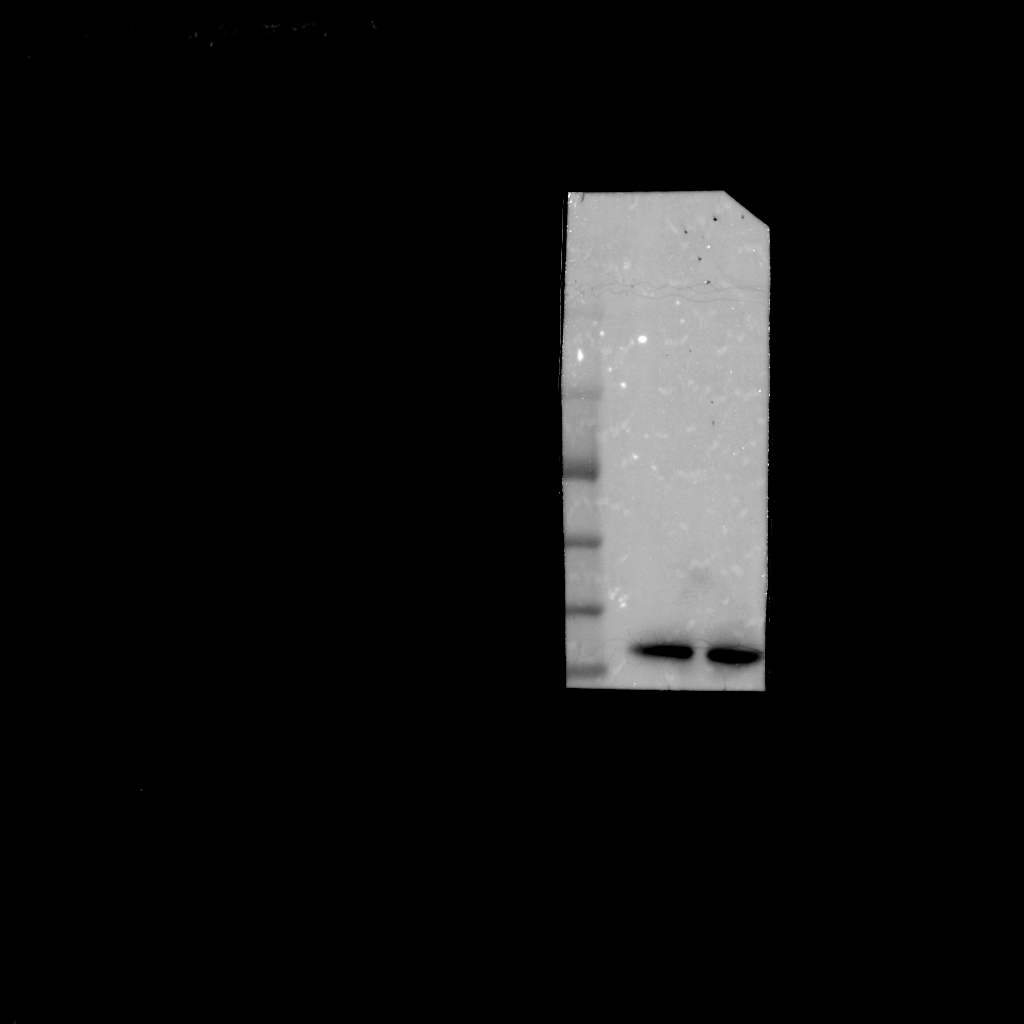

Supplement: Supplemental Information 9 — The raw images of tumor size in nude mice, western blot strips of tumor tissue, and corresponding statistical maps. [file peerj-12-18497-s009.zip › Raw tumor data of nude mice/western blot/未命名导出/n-cad 第3张gapdh.jpg]

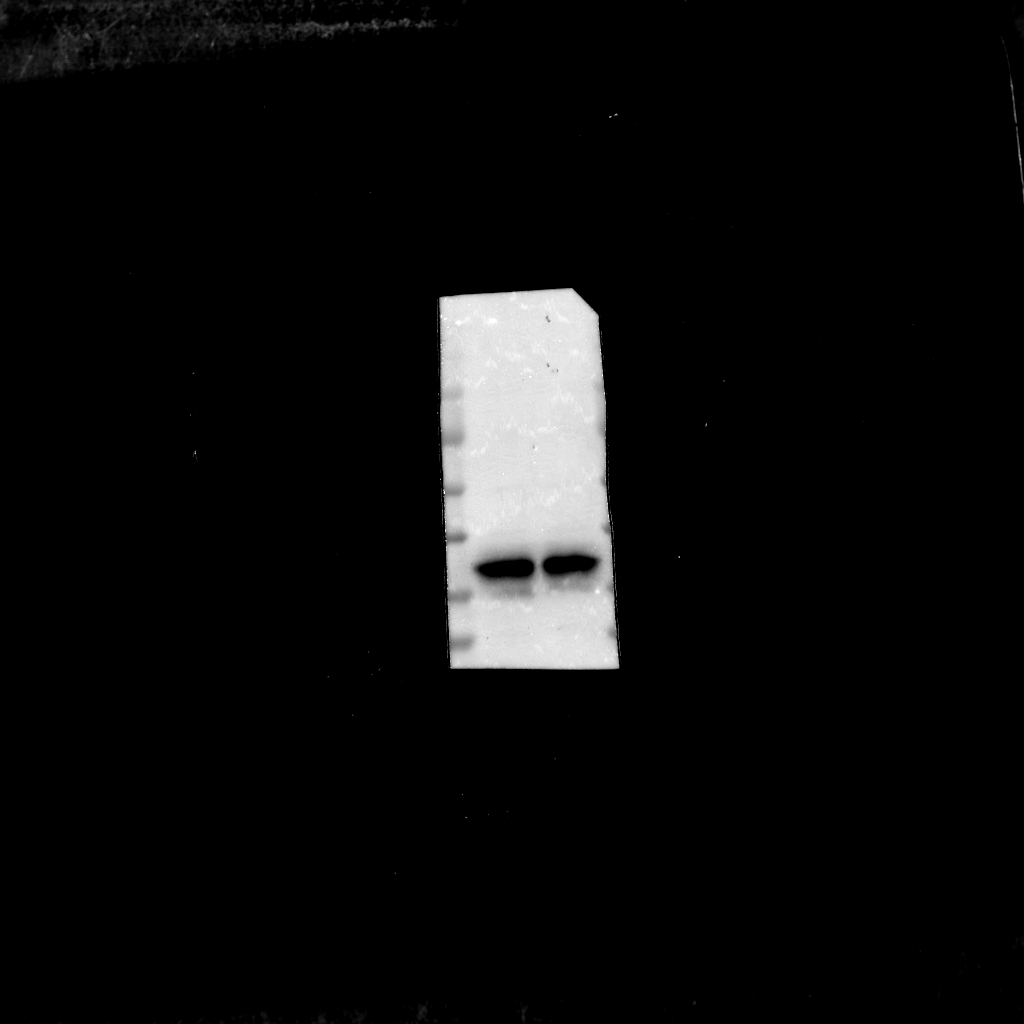

Supplement: Supplemental Information 9 — The raw images of tumor size in nude mice, western blot strips of tumor tissue, and corresponding statistical maps. [file peerj-12-18497-s009.zip › Raw tumor data of nude mice/western blot/未命名导出/p-gsk3beita 第1张 gapdh.jpg]

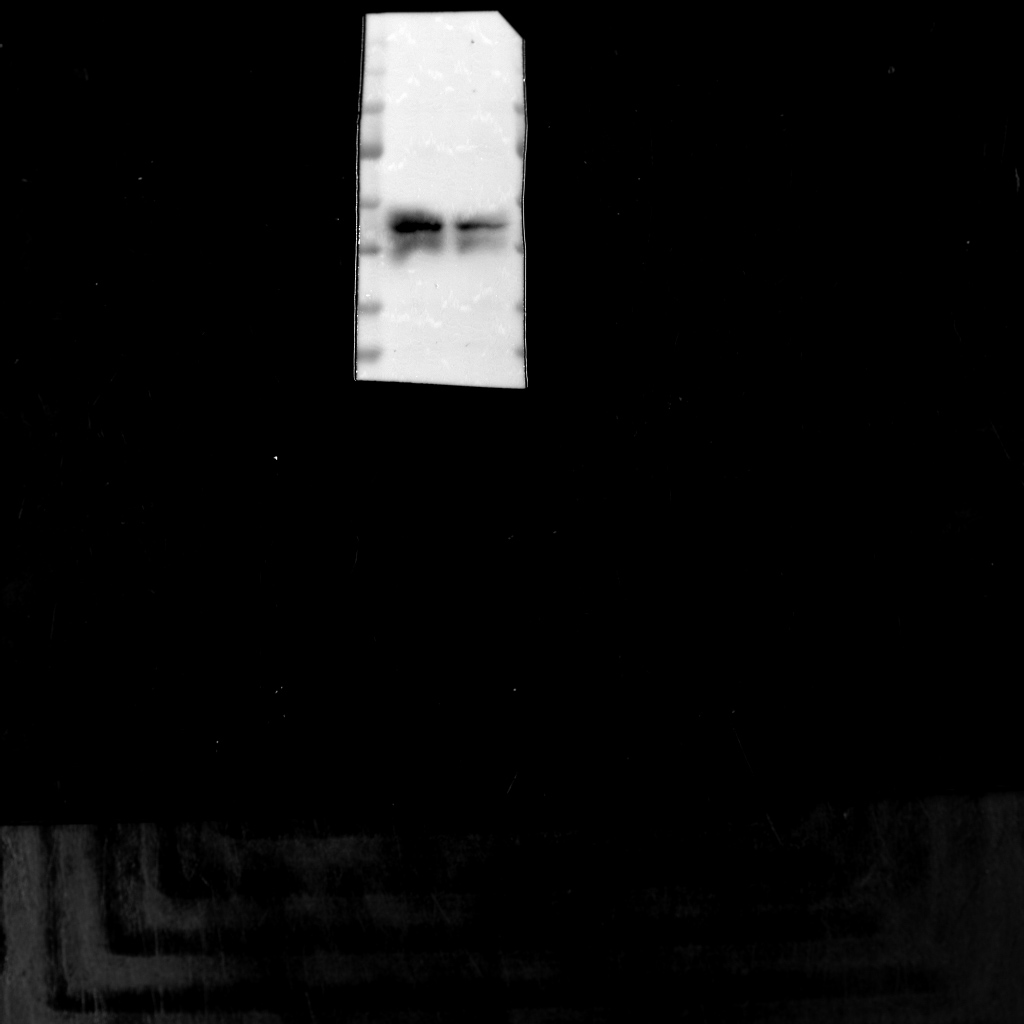

Supplement: Supplemental Information 9 — The raw images of tumor size in nude mice, western blot strips of tumor tissue, and corresponding statistical maps. [file peerj-12-18497-s009.zip › Raw tumor data of nude mice/western blot/未命名导出/p-gsk3beita 第1张.jpg]

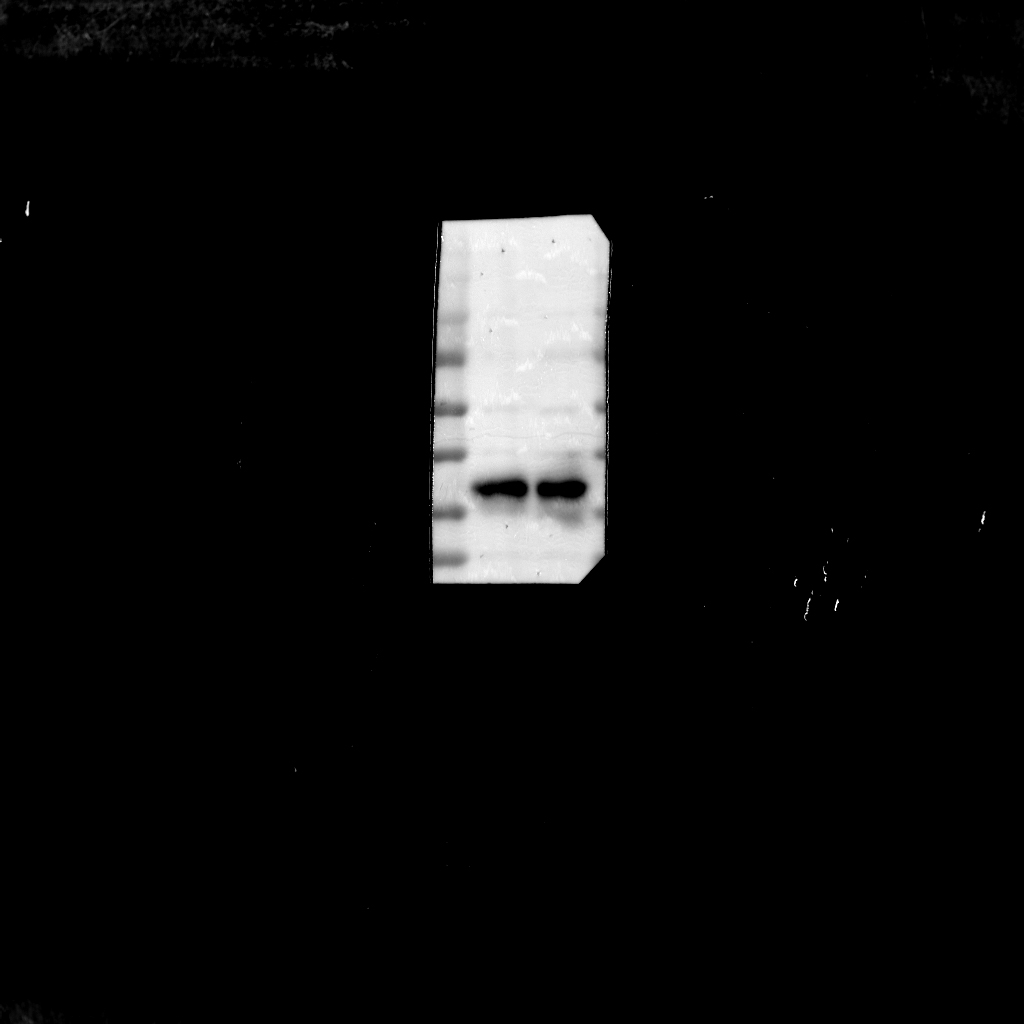

Supplement: Supplemental Information 9 — The raw images of tumor size in nude mice, western blot strips of tumor tissue, and corresponding statistical maps. [file peerj-12-18497-s009.zip › Raw tumor data of nude mice/western blot/未命名导出/p-gsk3beita 第2张 gapdh.jpg]

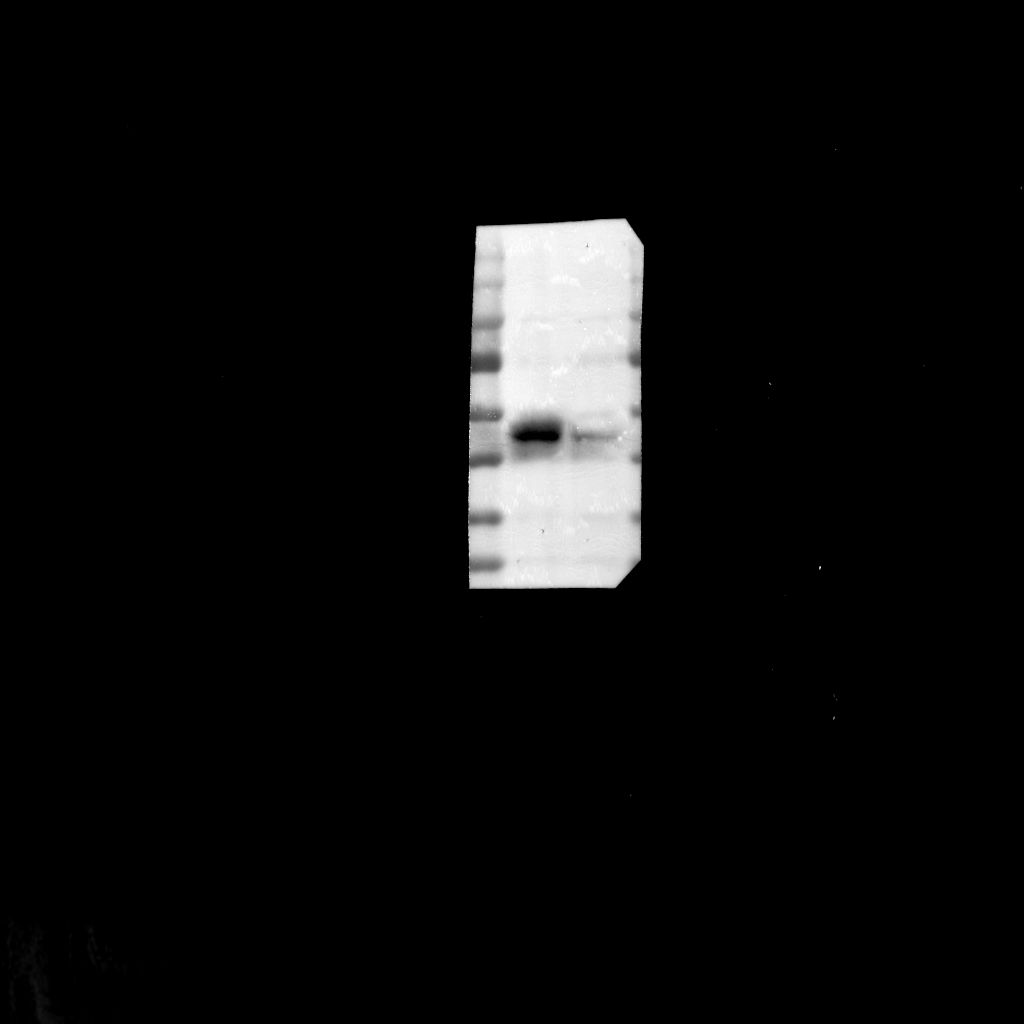

Supplement: Supplemental Information 9 — The raw images of tumor size in nude mice, western blot strips of tumor tissue, and corresponding statistical maps. [file peerj-12-18497-s009.zip › Raw tumor data of nude mice/western blot/未命名导出/p-gsk3beita 第2张.jpg]

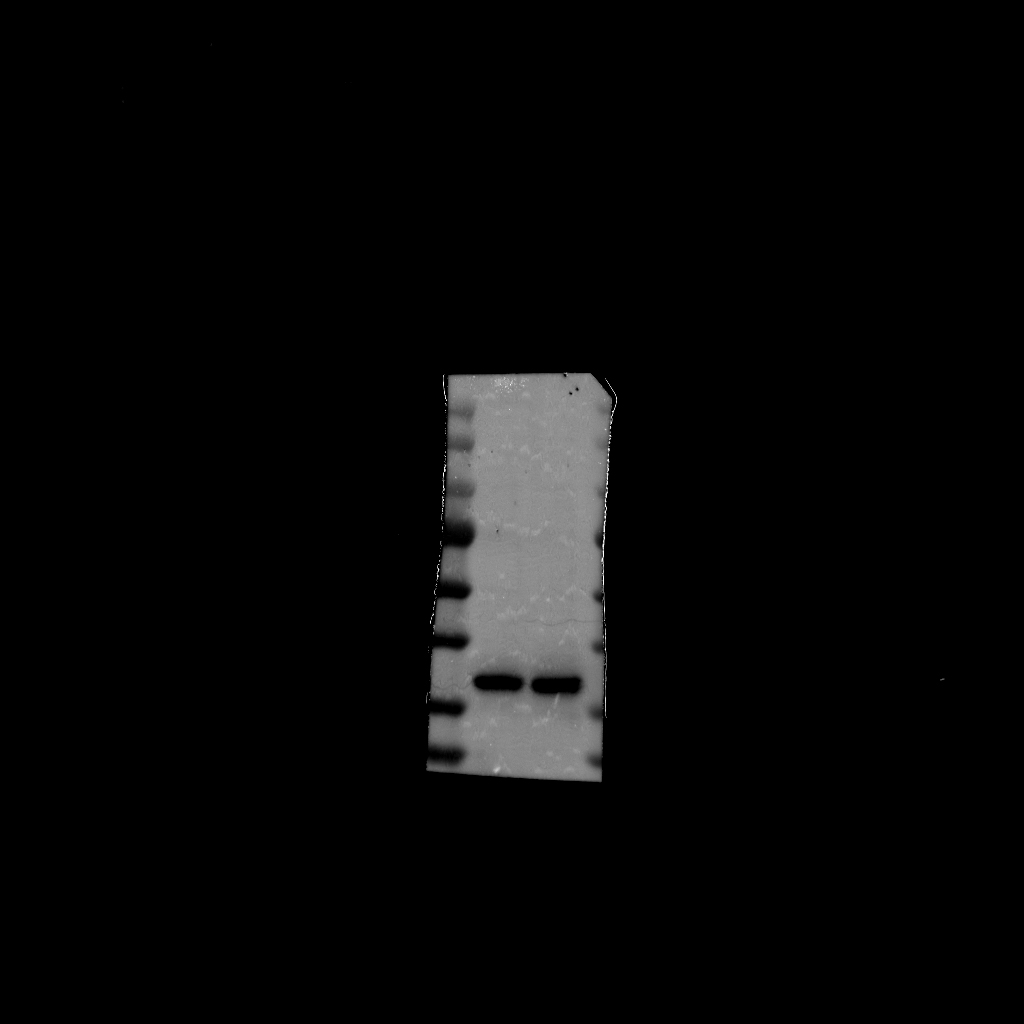

Supplement: Supplemental Information 9 — The raw images of tumor size in nude mice, western blot strips of tumor tissue, and corresponding statistical maps. [file peerj-12-18497-s009.zip › Raw tumor data of nude mice/western blot/未命名导出/p-gsk3beita 第3张 gapdh.jpg]

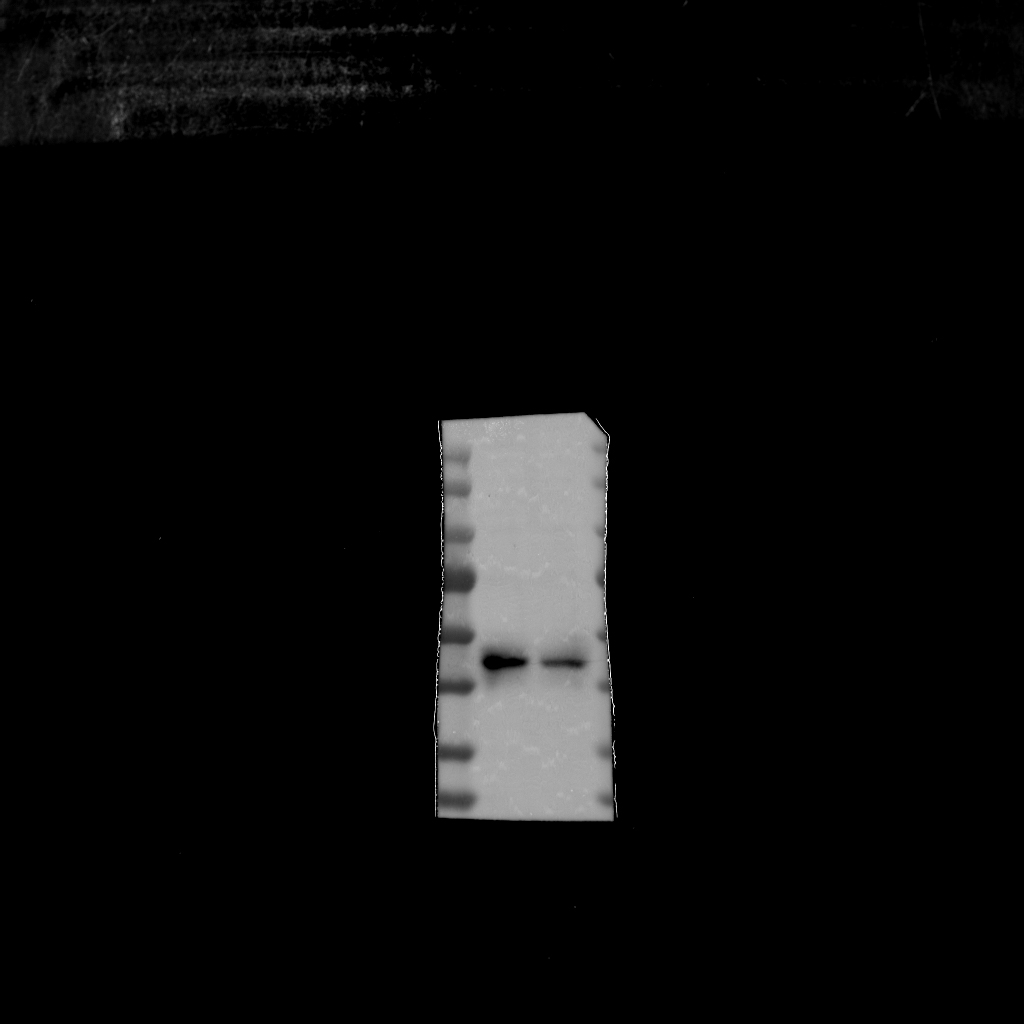

Supplement: Supplemental Information 9 — The raw images of tumor size in nude mice, western blot strips of tumor tissue, and corresponding statistical maps. [file peerj-12-18497-s009.zip › Raw tumor data of nude mice/western blot/未命名导出/p-gsk3beita 第3张.jpg]

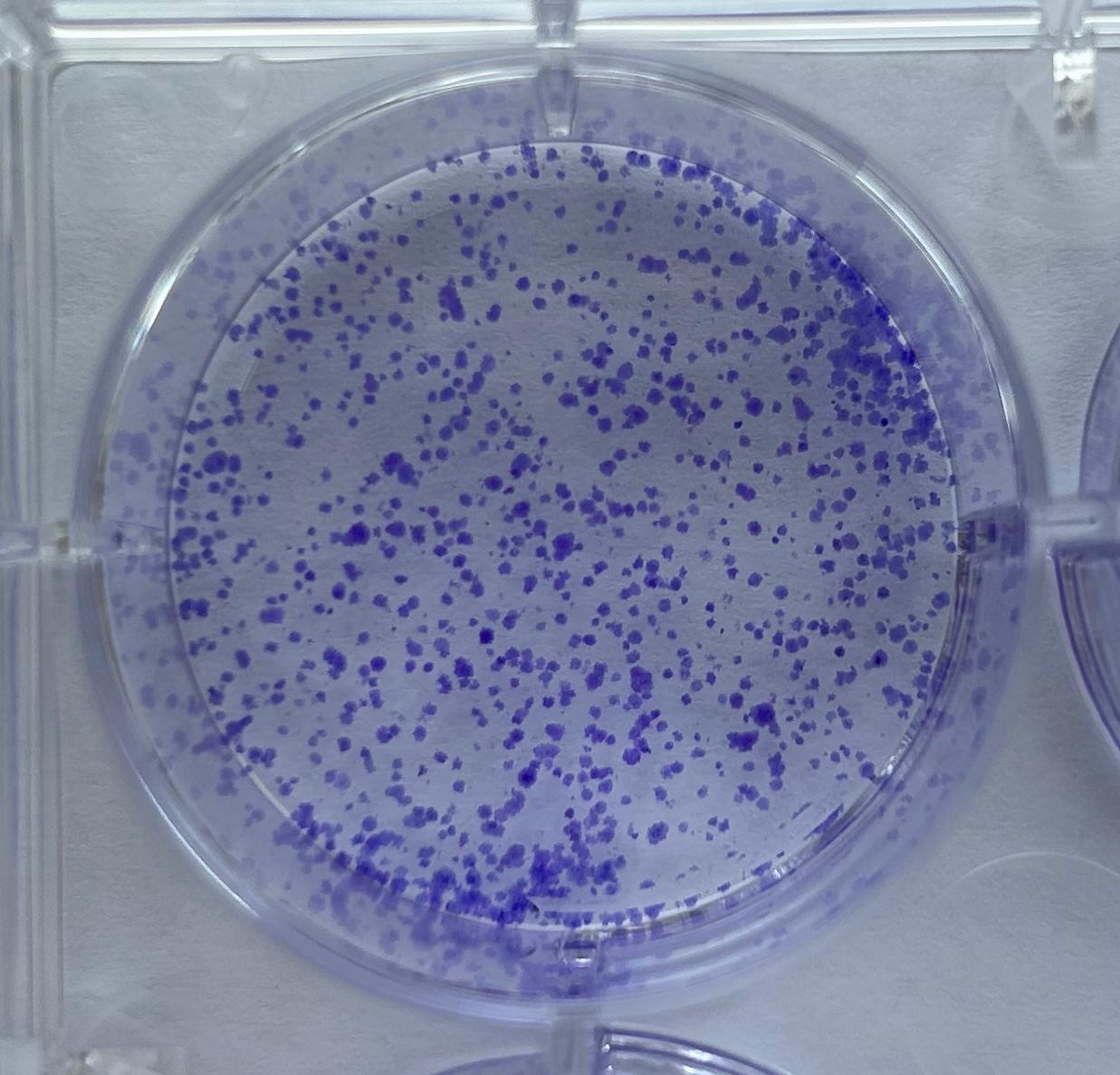

Supplement: Supplemental Information 11 [file peerj-12-18497-s011.zip › hucct1/hucct clone formation nc oe +Ca2+/nc1.jpg]

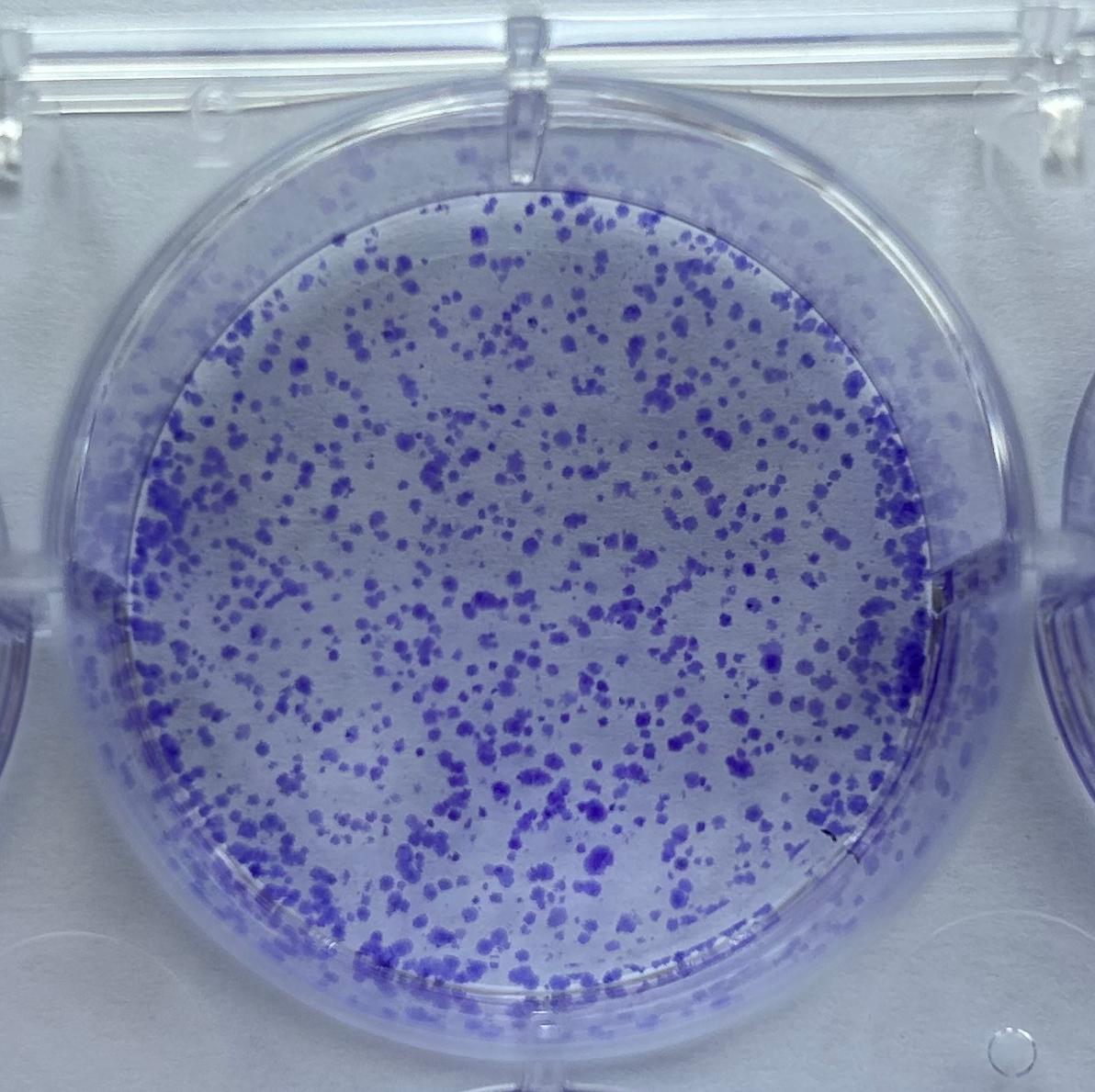

Supplement: Supplemental Information 11 [file peerj-12-18497-s011.zip › hucct1/hucct clone formation nc oe +Ca2+/nc2.jpg]

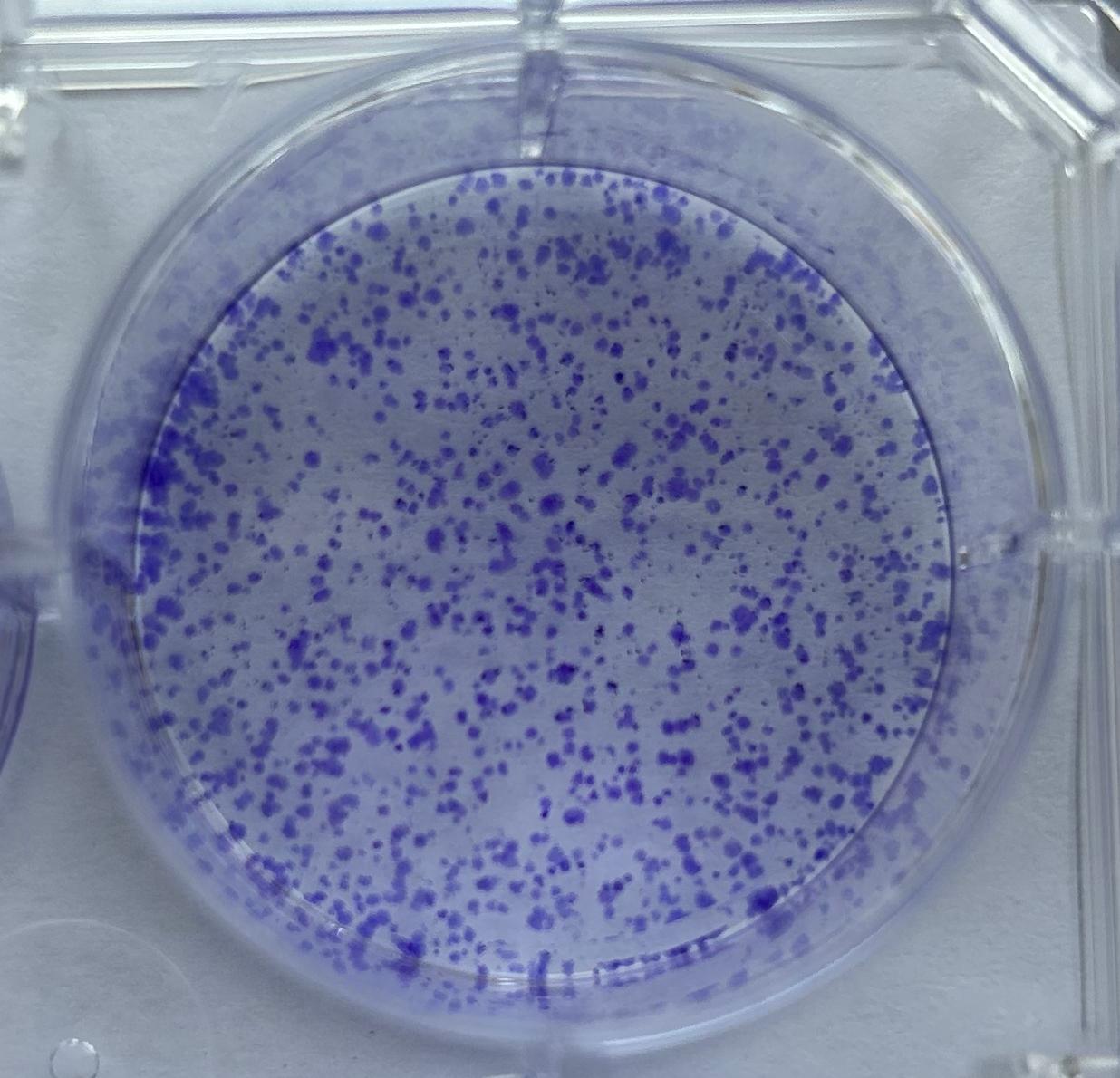

Supplement: Supplemental Information 11 [file peerj-12-18497-s011.zip › hucct1/hucct clone formation nc oe +Ca2+/nc3.jpg]

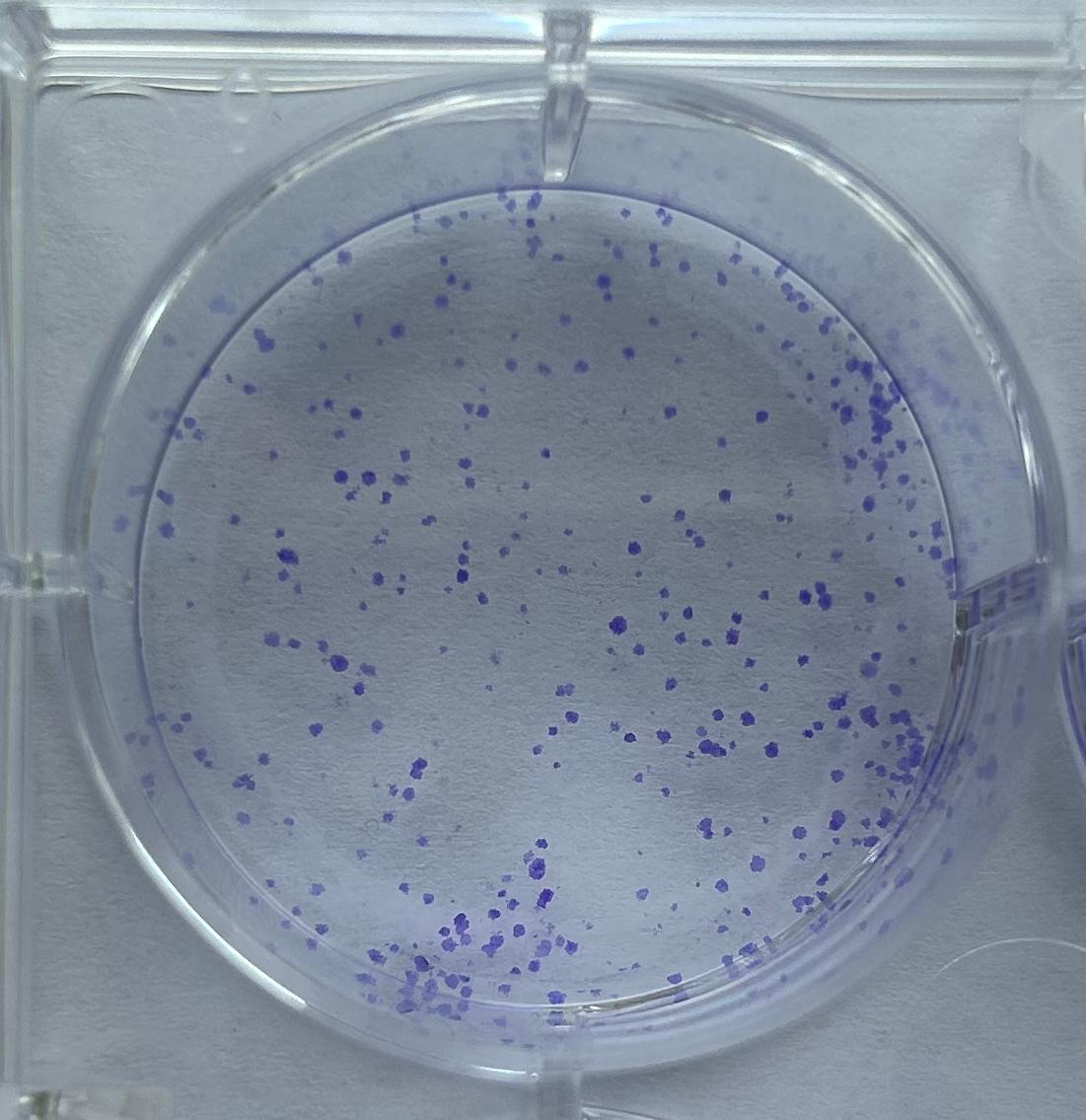

Supplement: Supplemental Information 11 [file peerj-12-18497-s011.zip › hucct1/hucct clone formation nc oe +Ca2+/oe+钙1.jpg]

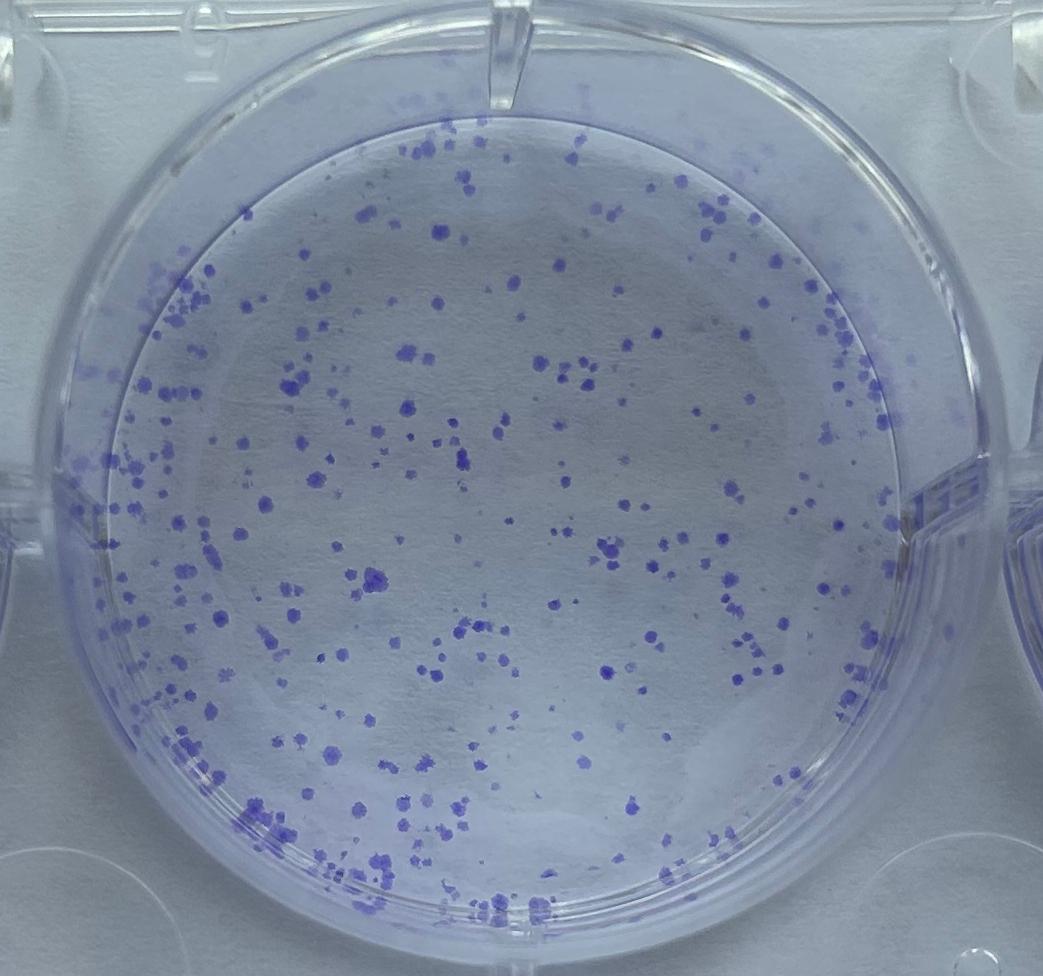

Supplement: Supplemental Information 11 [file peerj-12-18497-s011.zip › hucct1/hucct clone formation nc oe +Ca2+/oe+钙2.jpg]

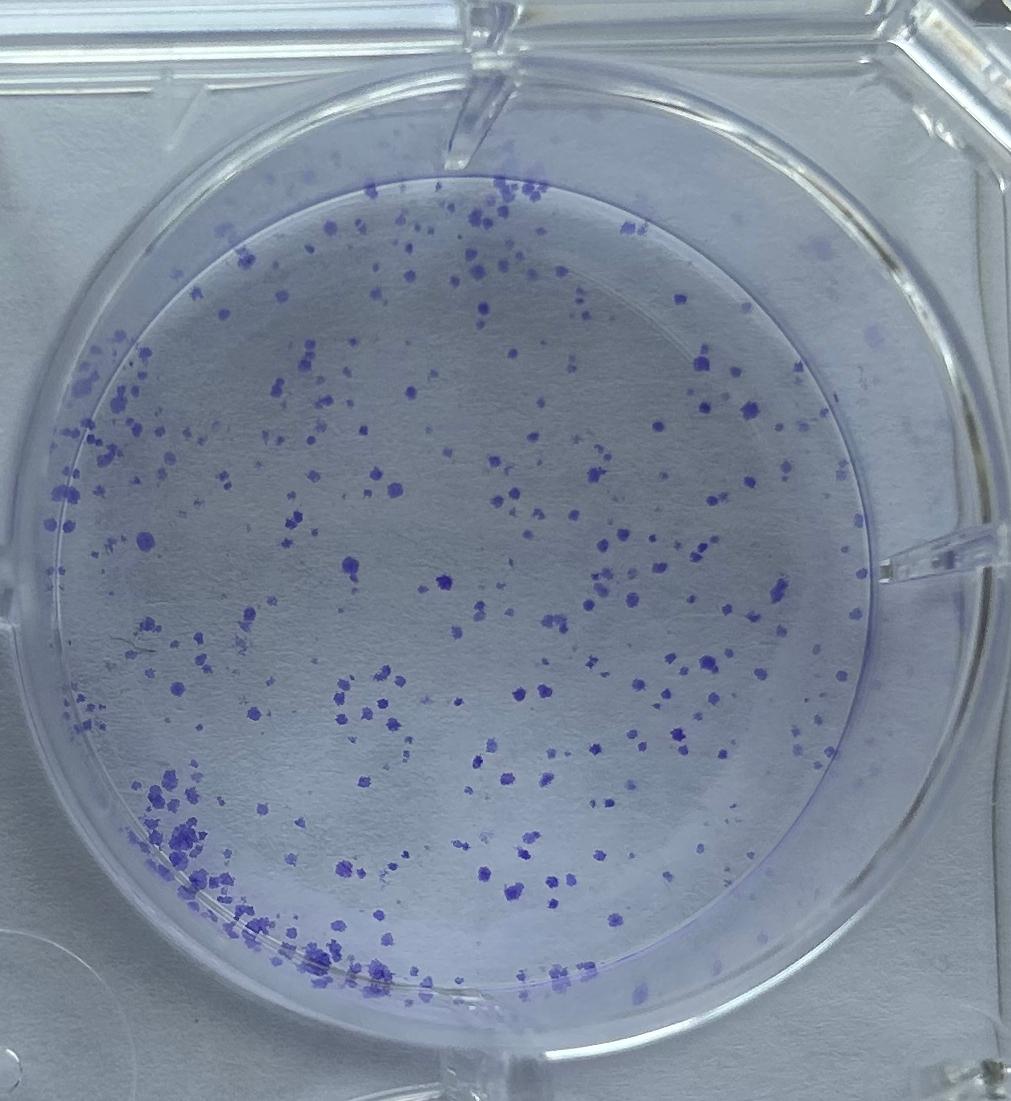

Supplement: Supplemental Information 11 [file peerj-12-18497-s011.zip › hucct1/hucct clone formation nc oe +Ca2+/oe+钙3.jpg]

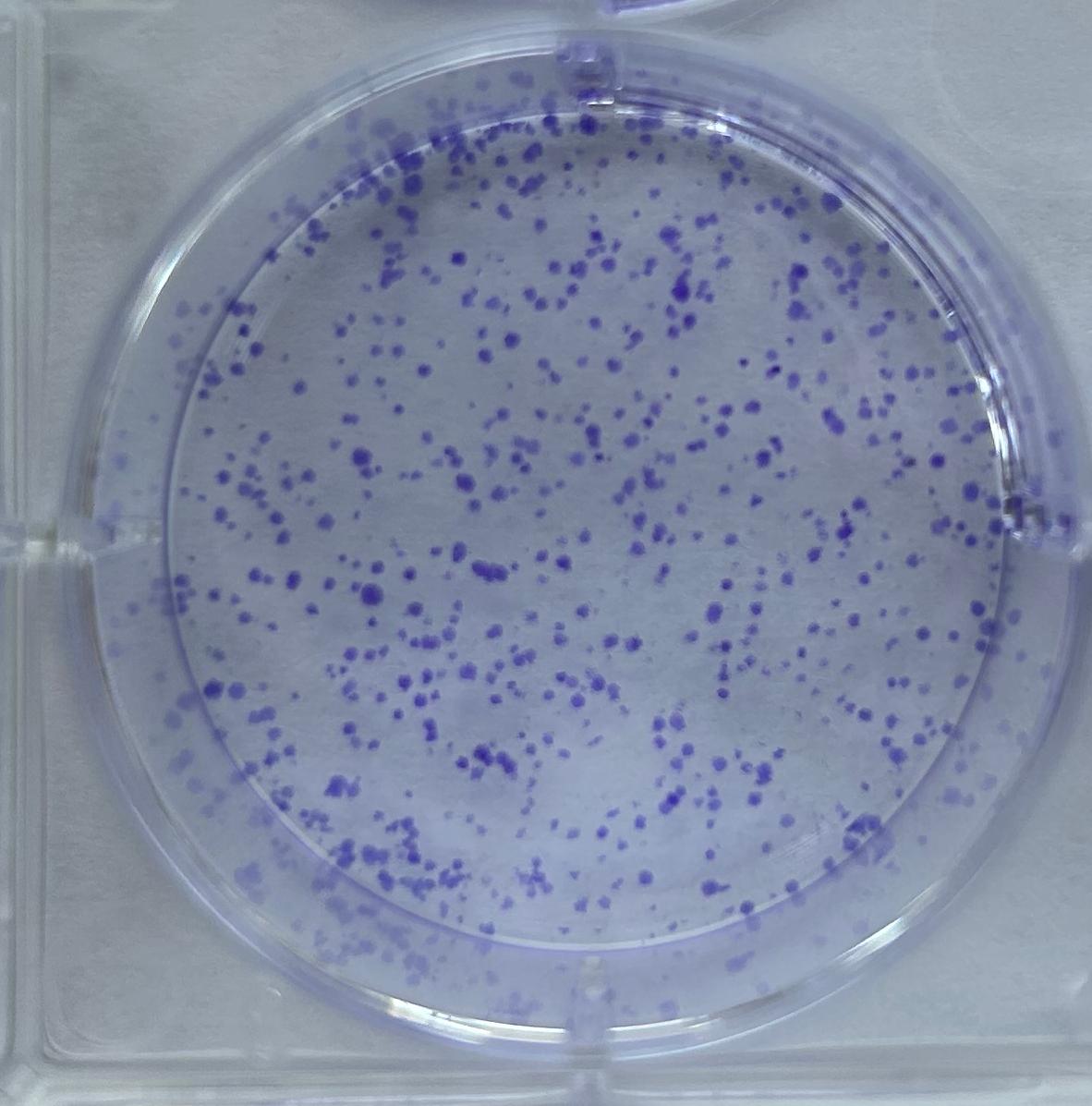

Supplement: Supplemental Information 11 [file peerj-12-18497-s011.zip › hucct1/hucct clone formation nc oe +Ca2+/oe1.jpg]

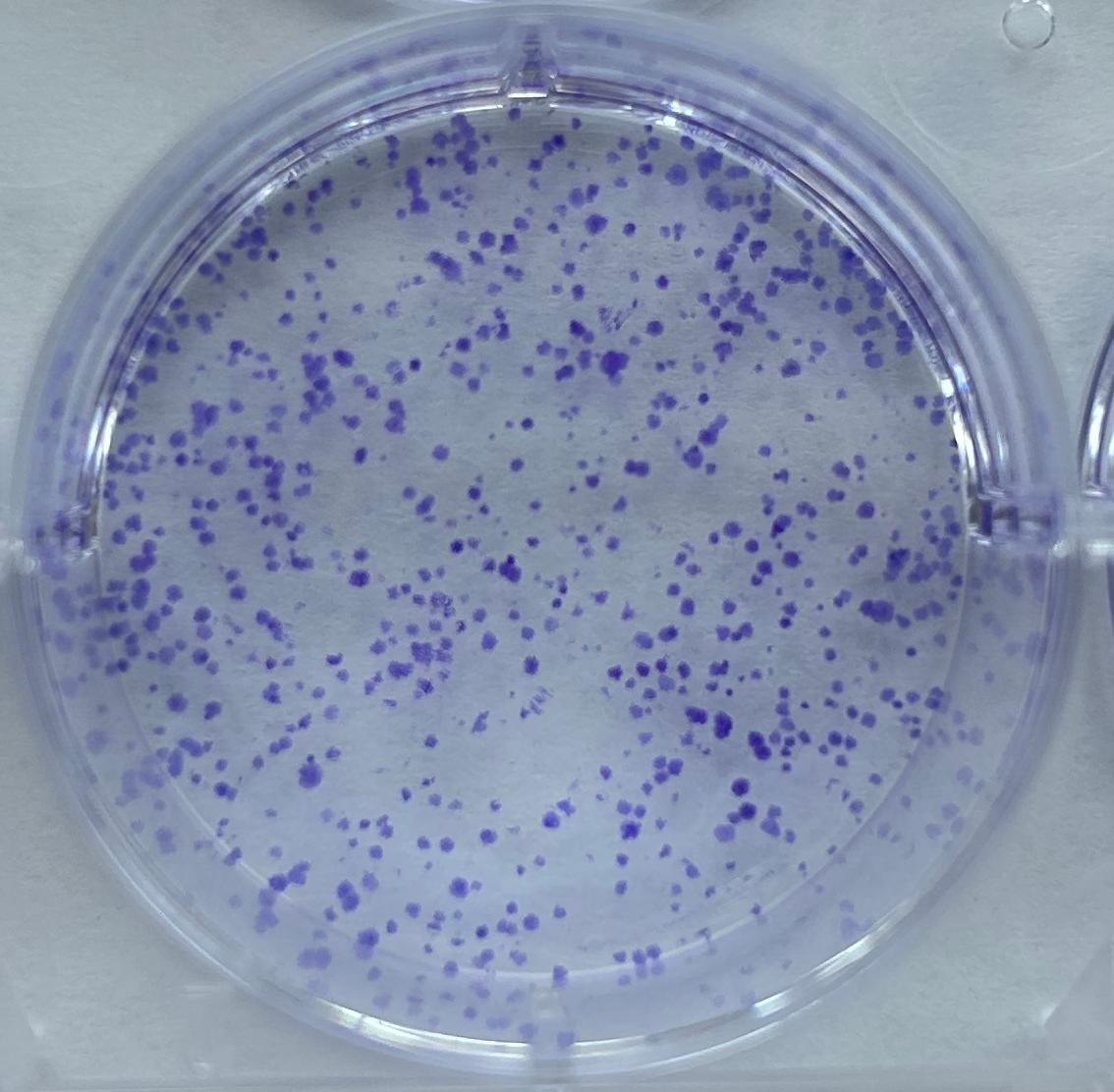

Supplement: Supplemental Information 11 [file peerj-12-18497-s011.zip › hucct1/hucct clone formation nc oe +Ca2+/oe2.jpg]

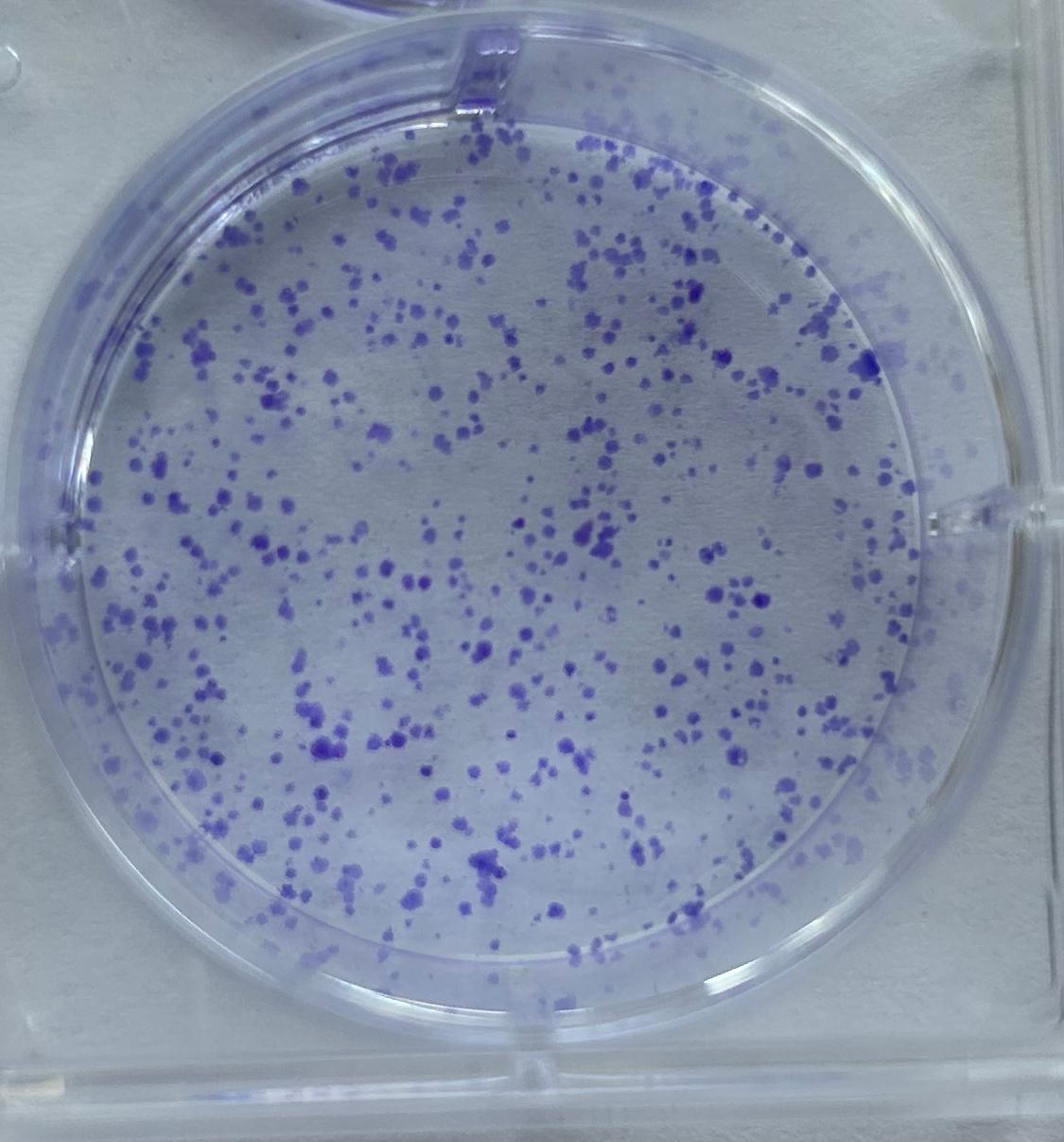

Supplement: Supplemental Information 11 [file peerj-12-18497-s011.zip › hucct1/hucct clone formation nc oe +Ca2+/oe3.jpg]

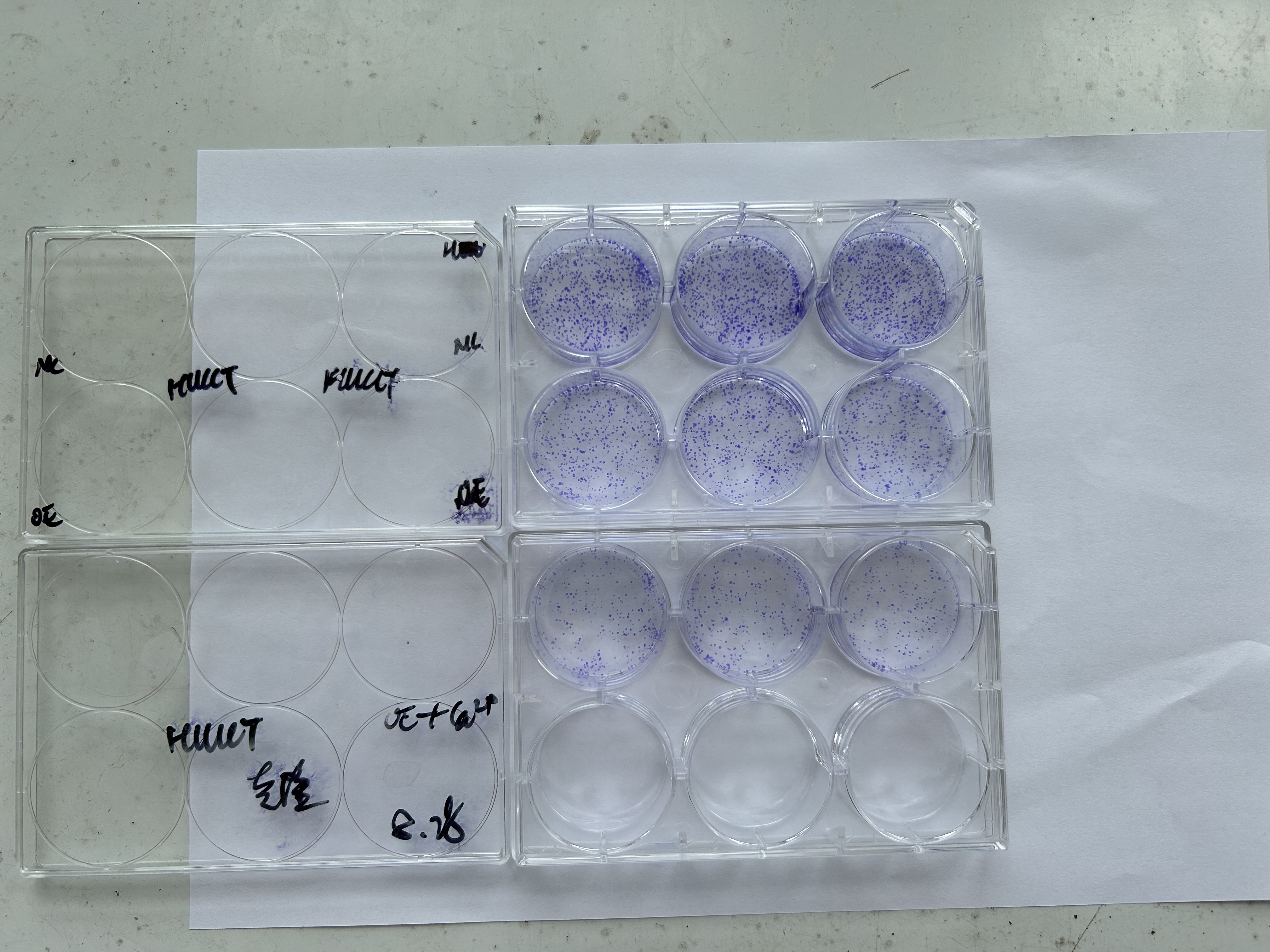

Supplement: Supplemental Information 11 [file peerj-12-18497-s011.zip › hucct1/hucct clone formation nc oe +Ca2+/全2.jpg]

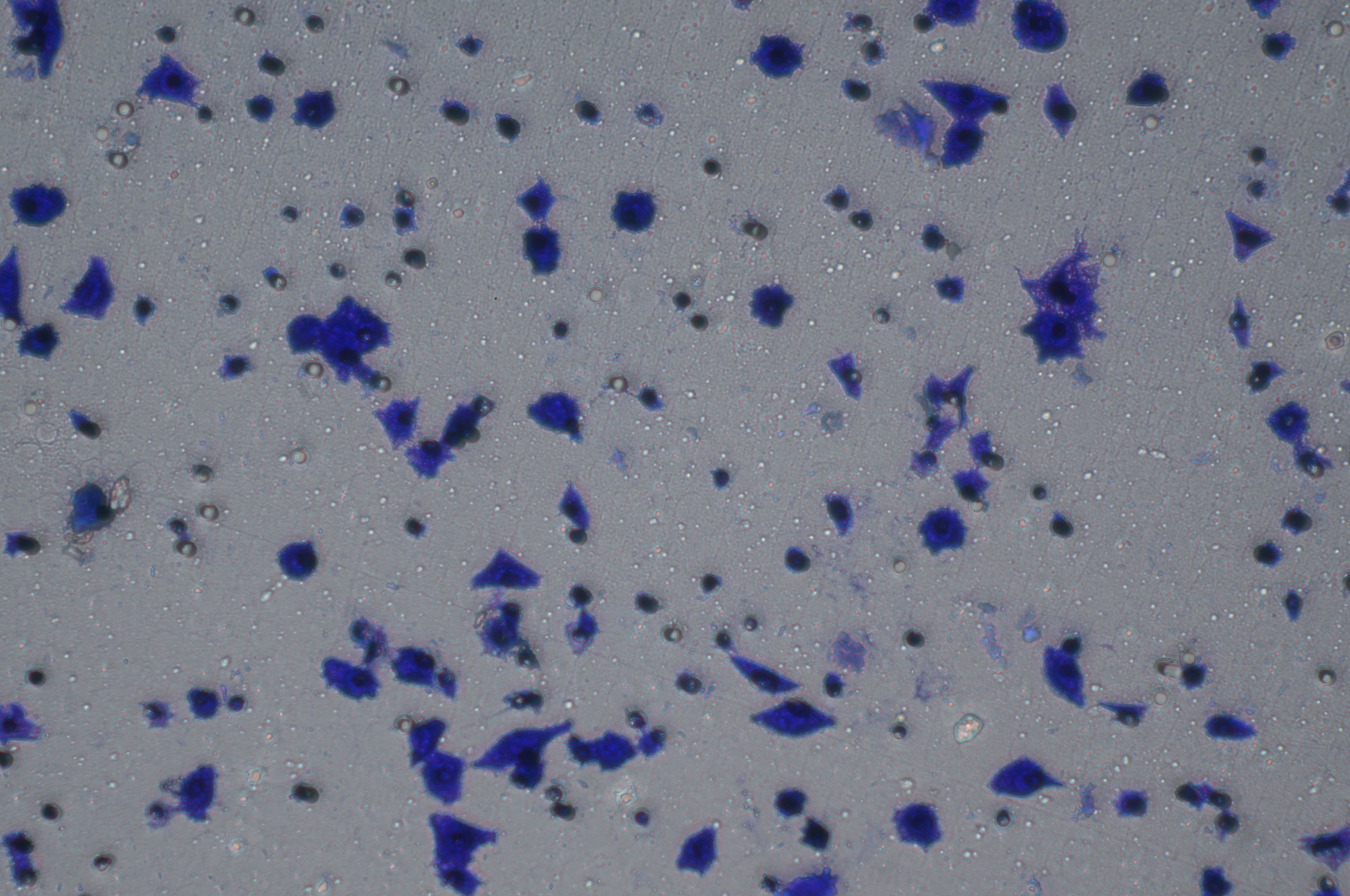

Supplement: Supplemental Information 11 [file peerj-12-18497-s011.zip › hucct1/hucct migration nc oe +Ca2+/picture/hucct clec3b 孔1 29.jpg]

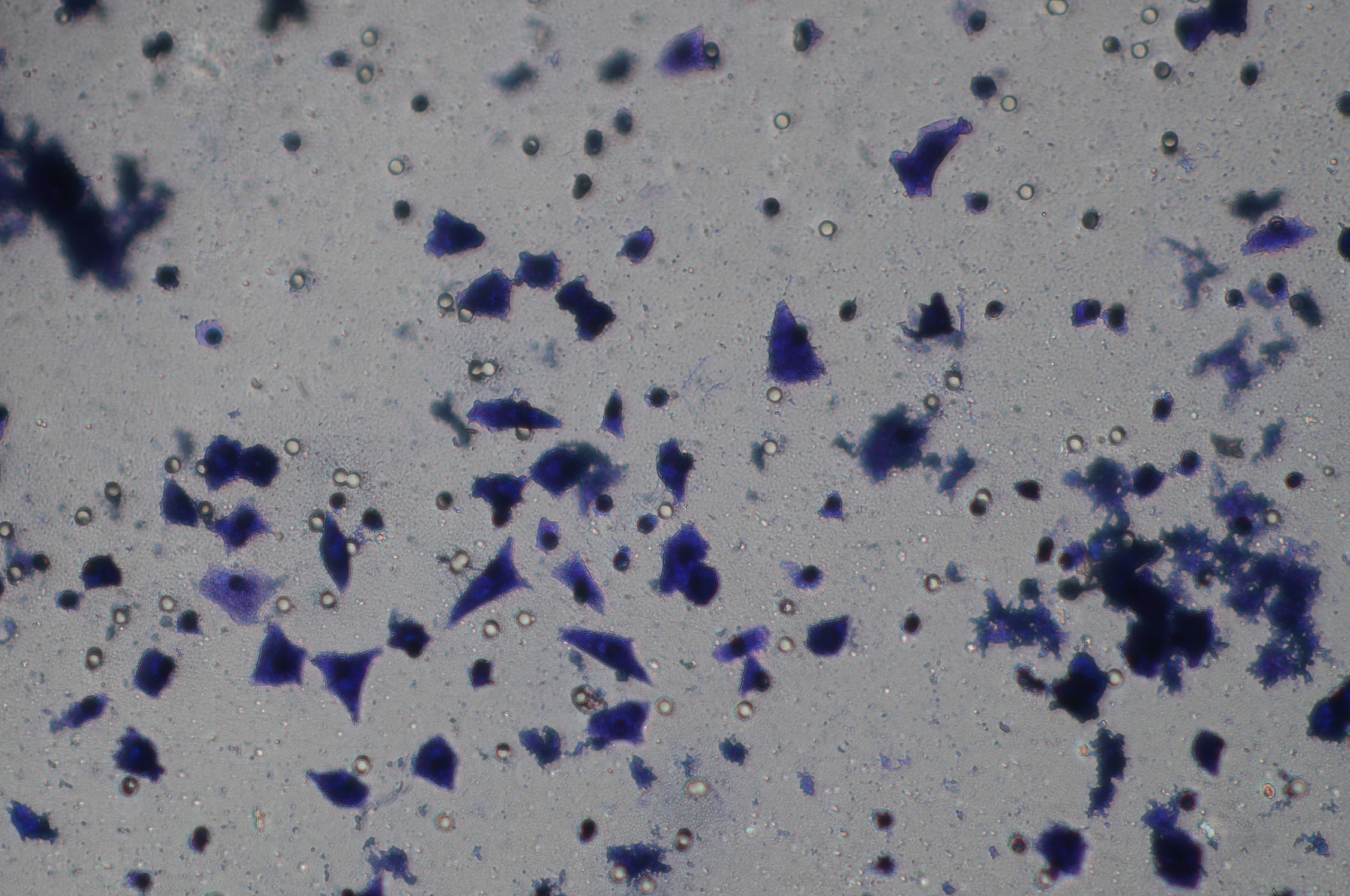

Supplement: Supplemental Information 11 [file peerj-12-18497-s011.zip › hucct1/hucct migration nc oe +Ca2+/picture/hucct clec3b 孔2 37.jpg]

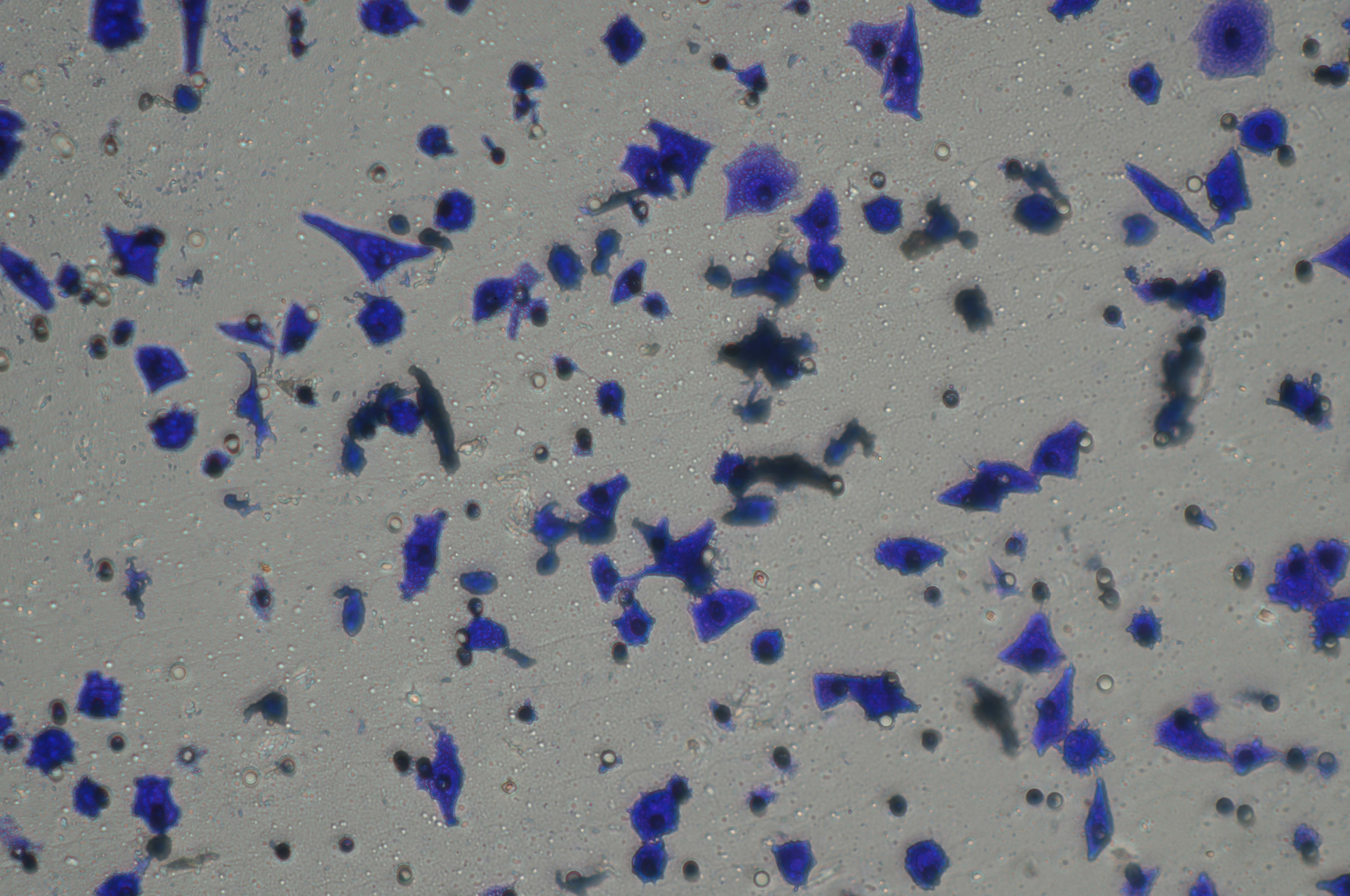

Supplement: Supplemental Information 11 [file peerj-12-18497-s011.zip › hucct1/hucct migration nc oe +Ca2+/picture/hucct clec3b 孔3 53.jpg]

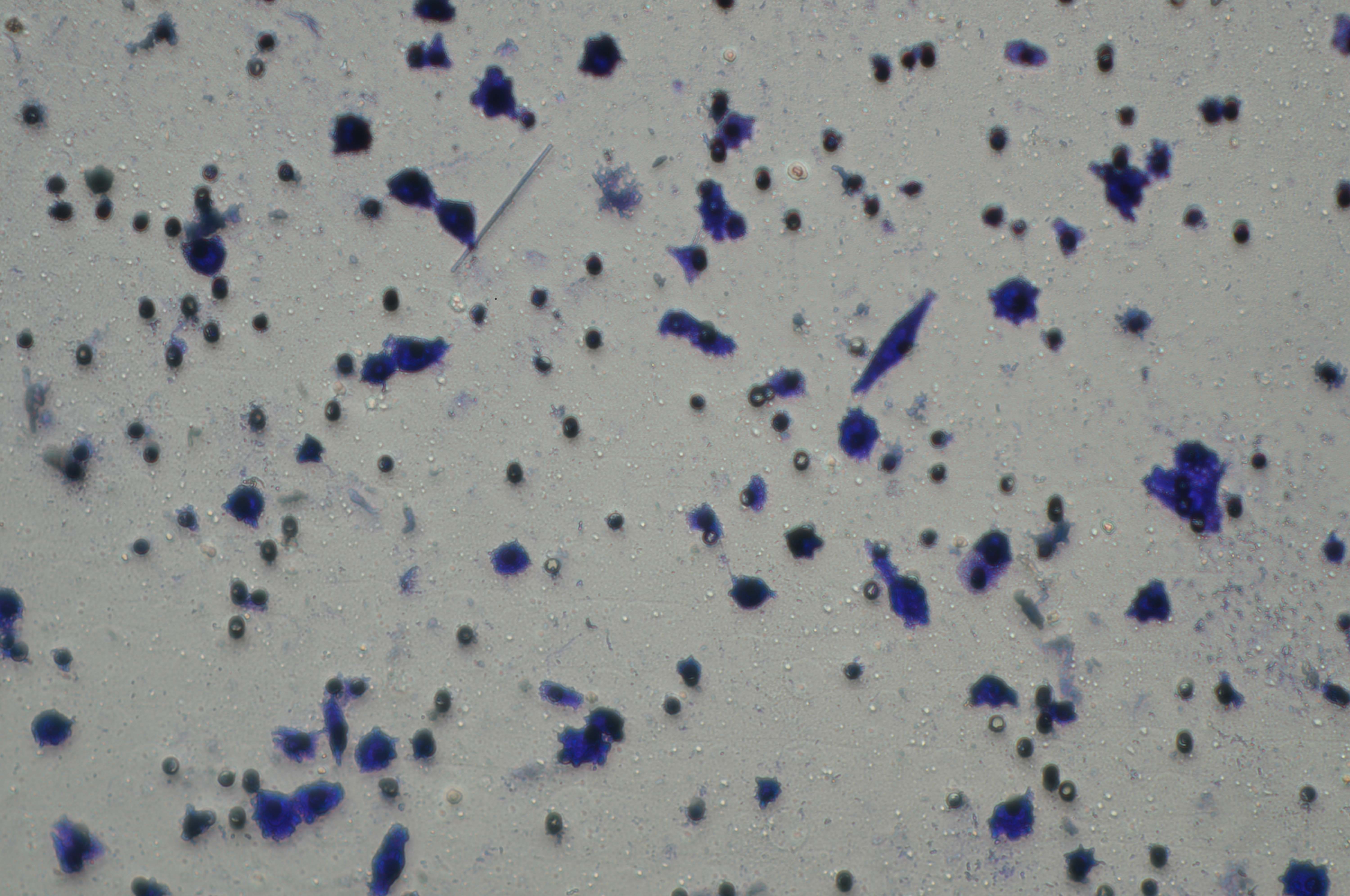

Supplement: Supplemental Information 11 [file peerj-12-18497-s011.zip › hucct1/hucct migration nc oe +Ca2+/picture/hucct clec3b加Ca2+ 孔1 58.jpg]

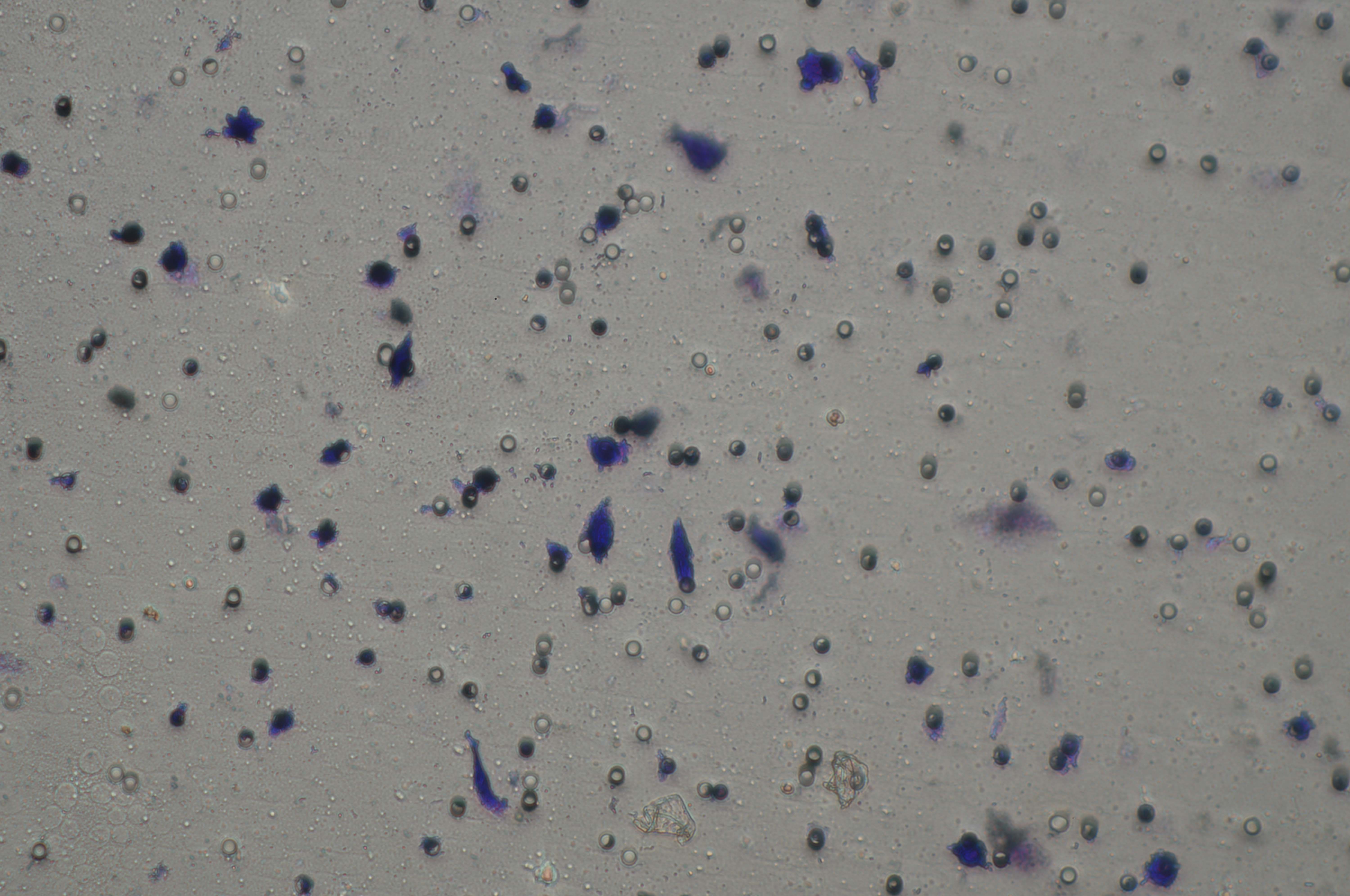

Supplement: Supplemental Information 11 [file peerj-12-18497-s011.zip › hucct1/hucct migration nc oe +Ca2+/picture/hucct clec3b加Ca2+ 孔267.jpg]

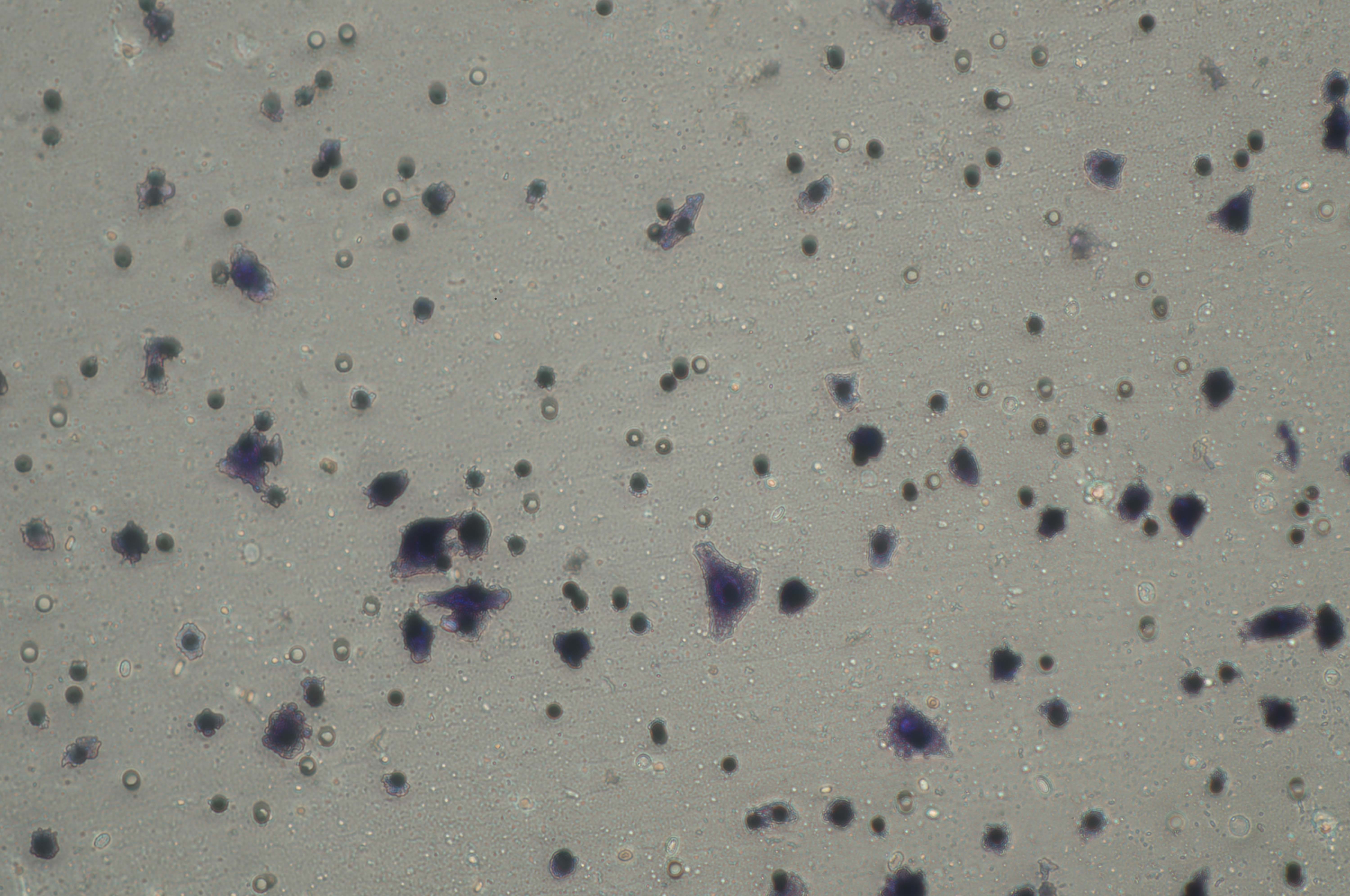

Supplement: Supplemental Information 11 [file peerj-12-18497-s011.zip › hucct1/hucct migration nc oe +Ca2+/picture/hucct clec3b加Ca2+ 孔373.jpg]

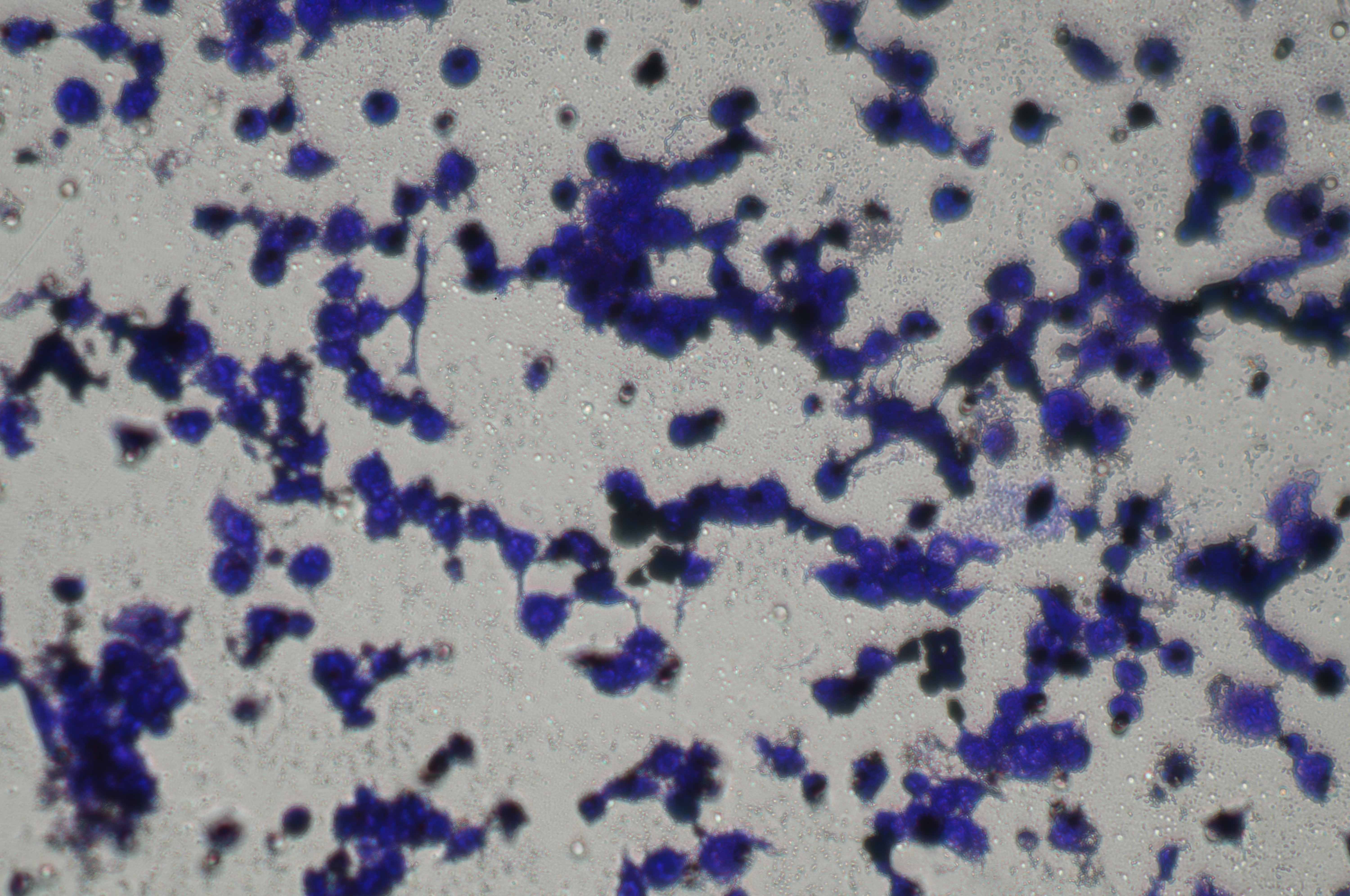

Supplement: Supplemental Information 11 [file peerj-12-18497-s011.zip › hucct1/hucct migration nc oe +Ca2+/picture/hucct nc 孔1.jpg]

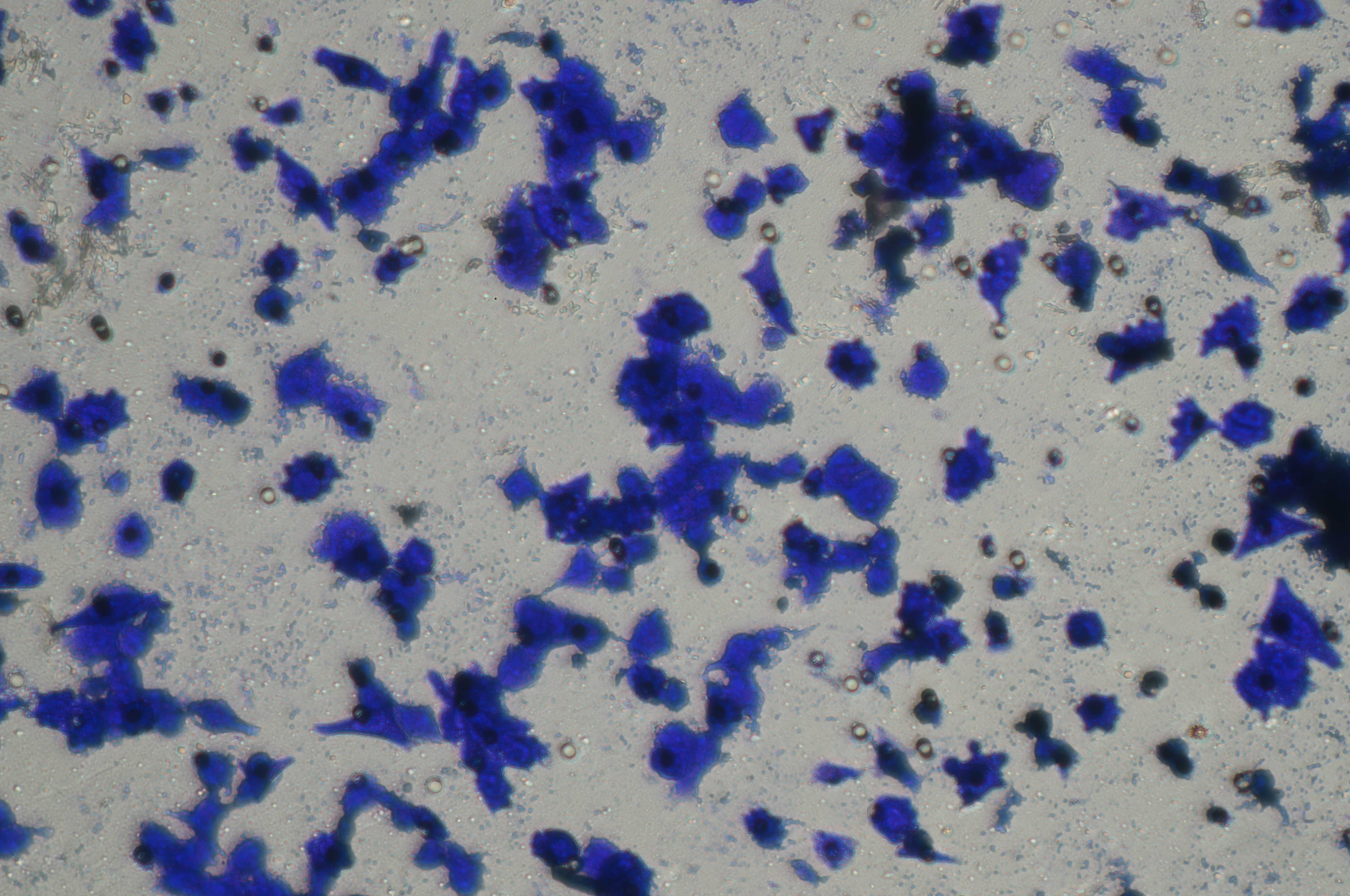

Supplement: Supplemental Information 11 [file peerj-12-18497-s011.zip › hucct1/hucct migration nc oe +Ca2+/picture/hucct nc 孔2.jpg]
