# Supplementary figures and images for: The CLEC3B inhibits cellular proliferation and metastasis of cholangiocarcinoma through Wnt/β-catenin pathway (part 4 of 5)
Source: PeerJ. 2024 Nov 13;12:e18497. doi: 10.7717/peerj.18497 (PMC11568818; doi:10.7717/peerj.18497)

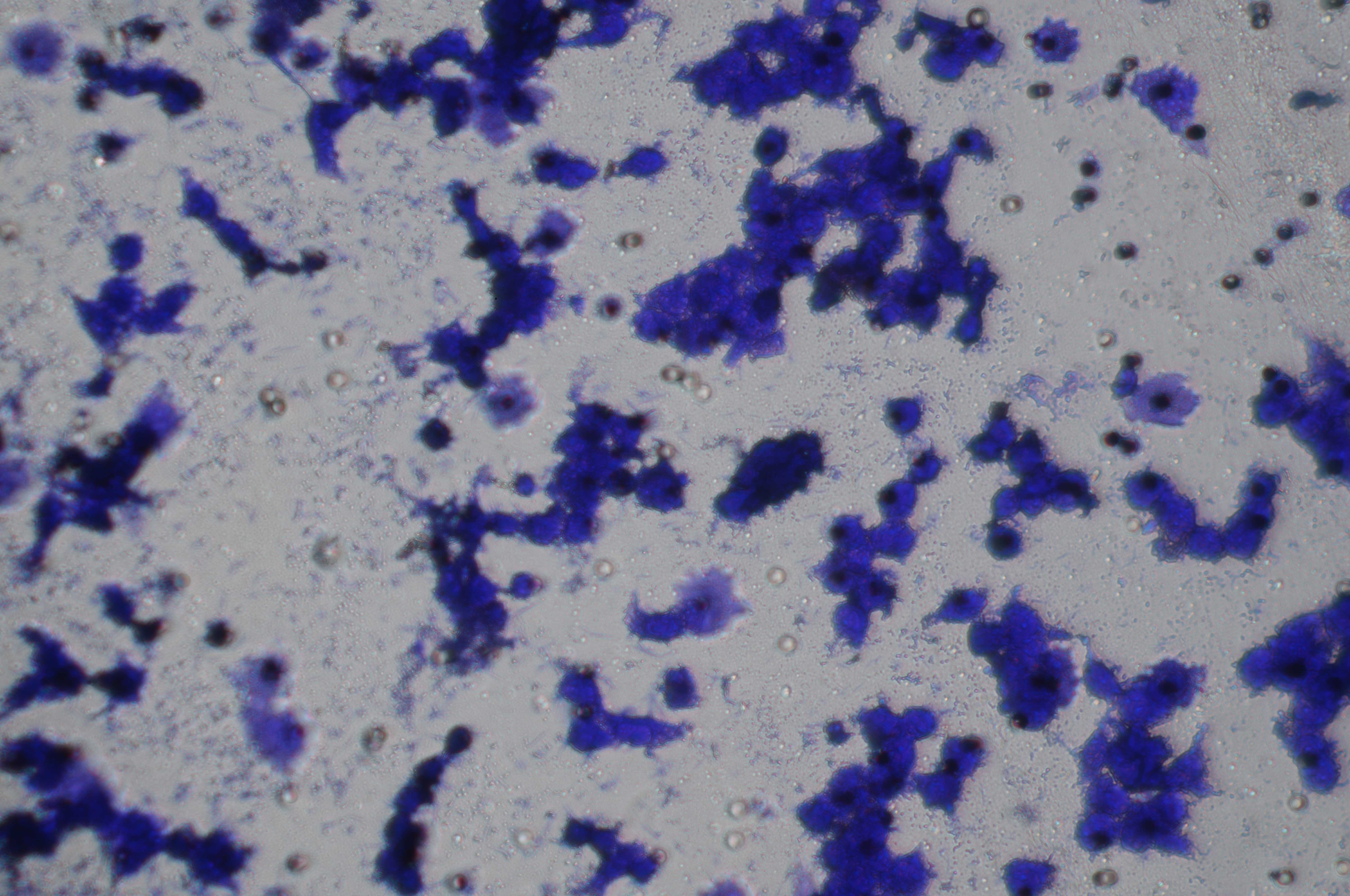

Supplement: Supplemental Information 11 [file peerj-12-18497-s011.zip › hucct1/hucct migration nc oe +Ca2+/picture/hucct nc 孔3.jpg]

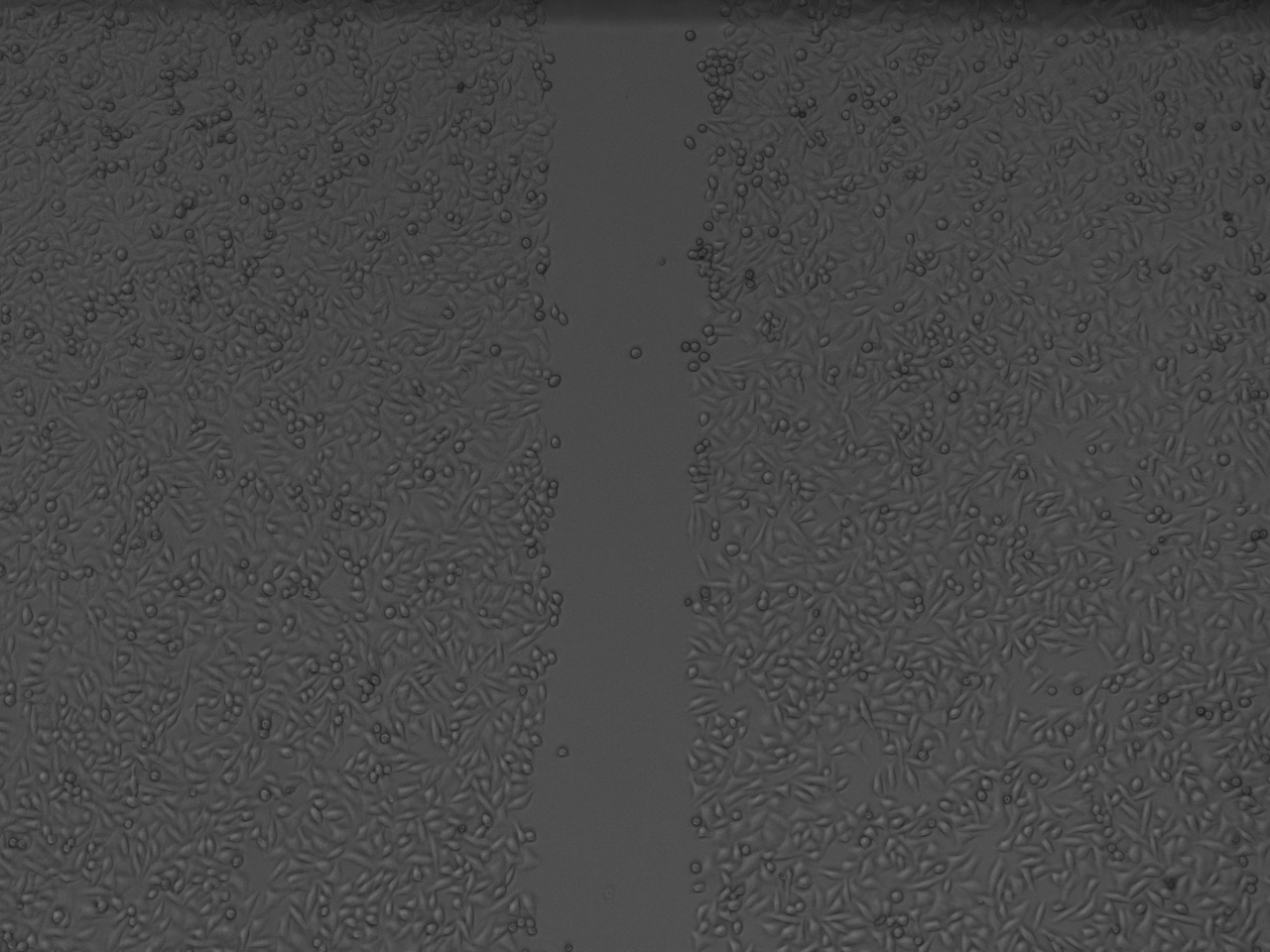

Supplement: Supplemental Information 11 [file peerj-12-18497-s011.zip › hucct1/hucct1 Wound Healing nc oe oe+Ca2+/nc/k2 b 0h.tif]

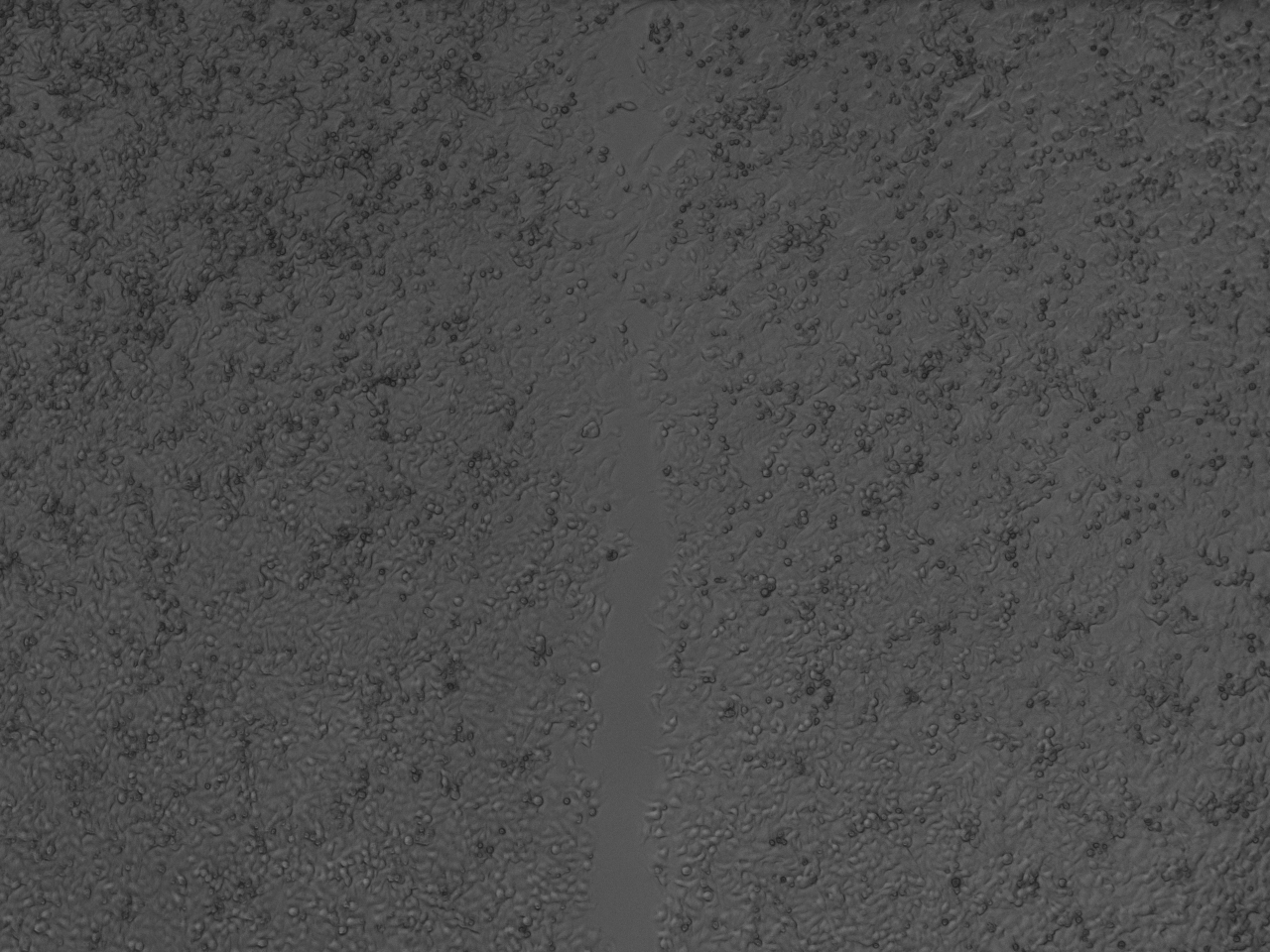

Supplement: Supplemental Information 11 [file peerj-12-18497-s011.zip › hucct1/hucct1 Wound Healing nc oe oe+Ca2+/nc/k2 b 24h.tif]

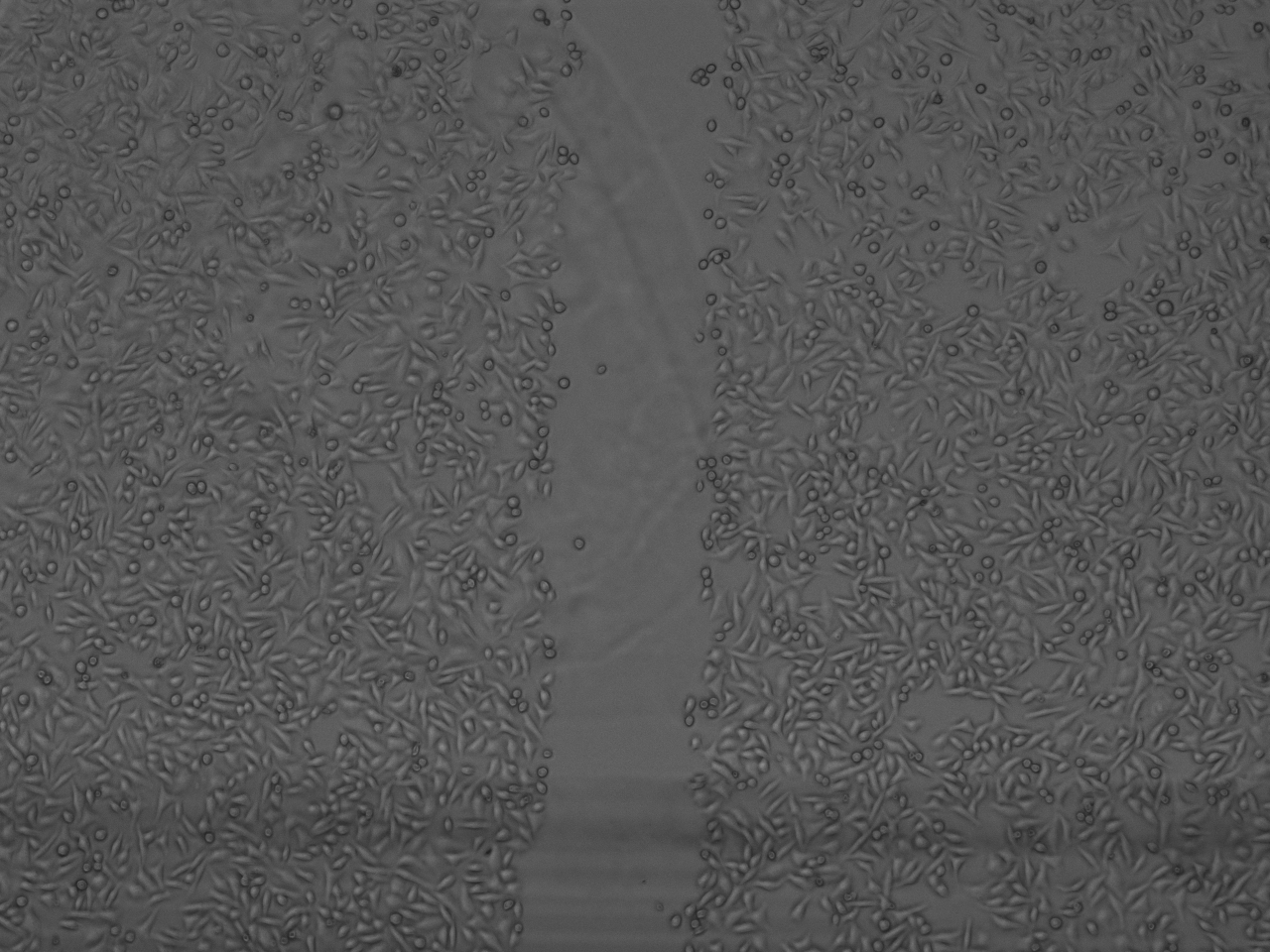

Supplement: Supplemental Information 11 [file peerj-12-18497-s011.zip › hucct1/hucct1 Wound Healing nc oe oe+Ca2+/nc/k2 c 0h.tif]

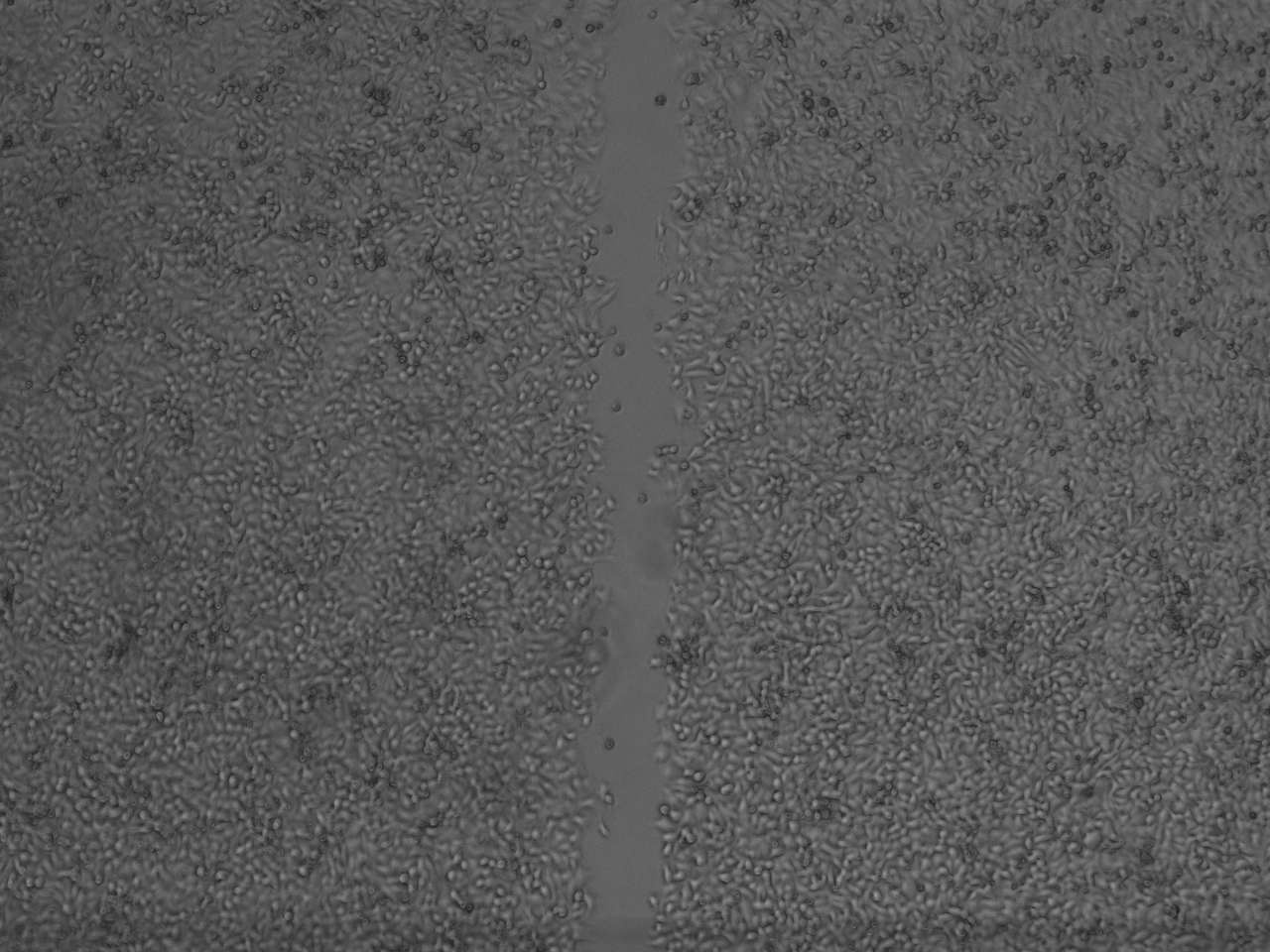

Supplement: Supplemental Information 11 [file peerj-12-18497-s011.zip › hucct1/hucct1 Wound Healing nc oe oe+Ca2+/nc/k2 c 24h.tif]

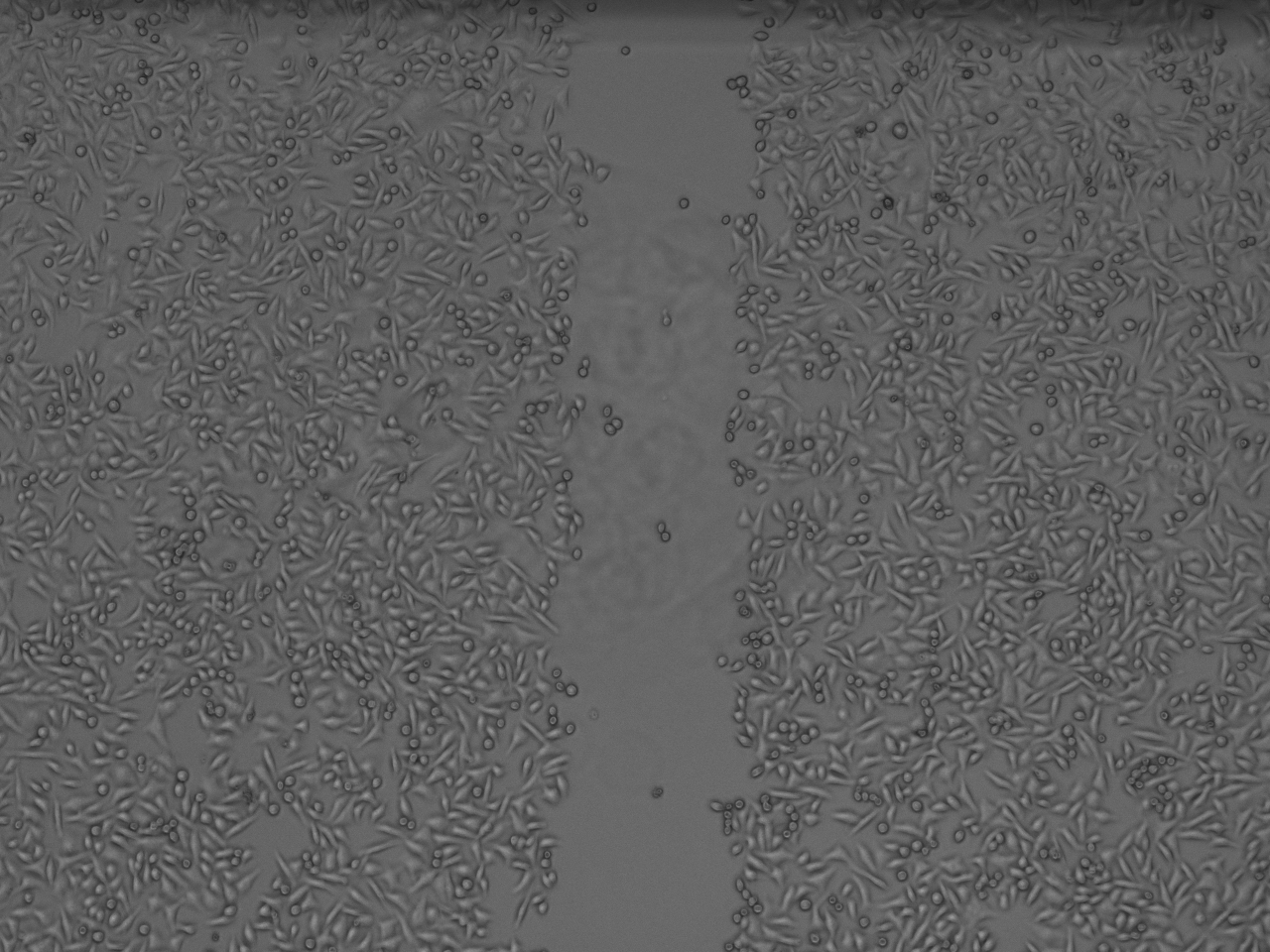

Supplement: Supplemental Information 11 [file peerj-12-18497-s011.zip › hucct1/hucct1 Wound Healing nc oe oe+Ca2+/nc/k2 d 0h.tif]

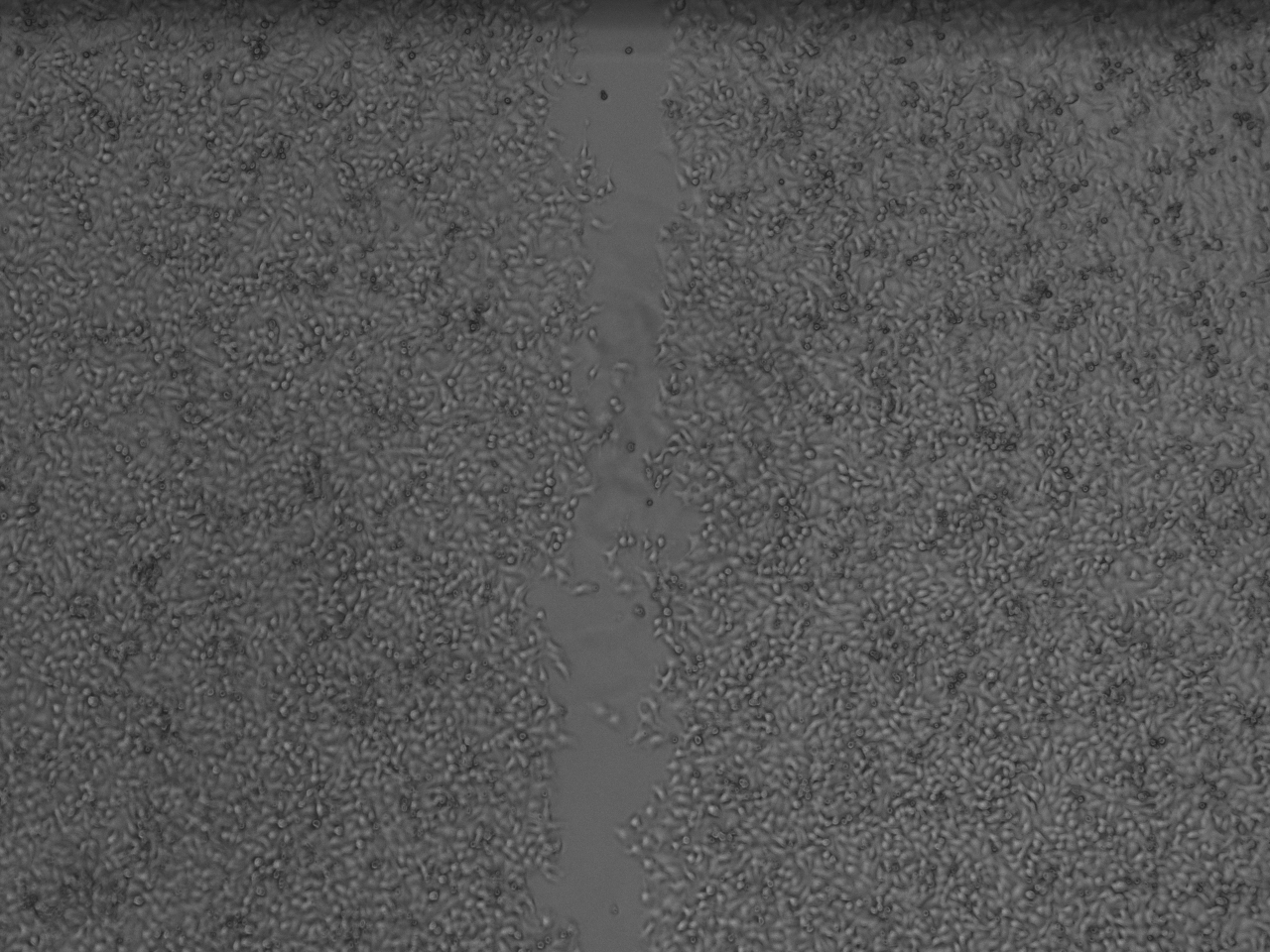

Supplement: Supplemental Information 11 [file peerj-12-18497-s011.zip › hucct1/hucct1 Wound Healing nc oe oe+Ca2+/nc/k2 d 24h.tif]

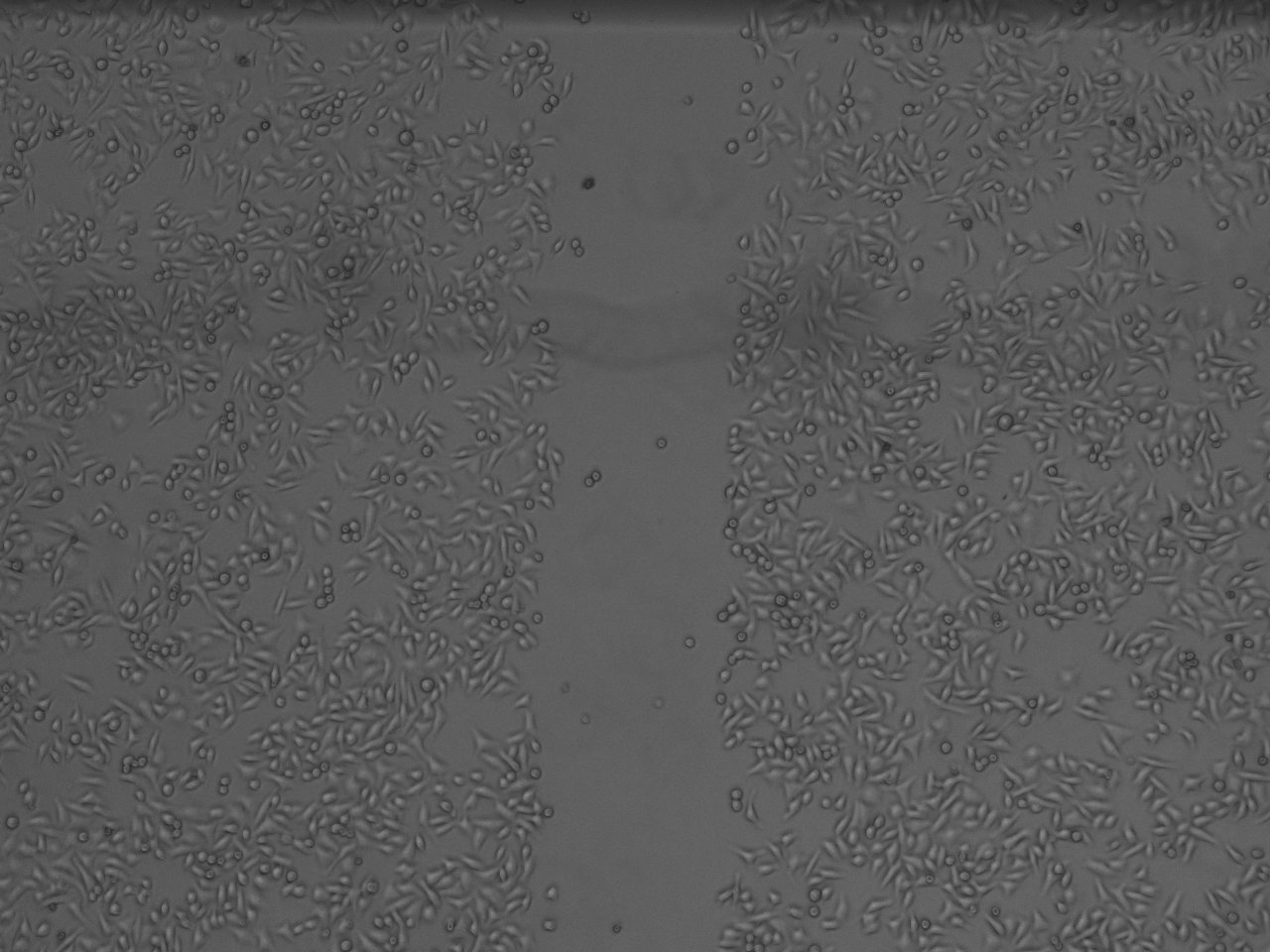

Supplement: Supplemental Information 11 [file peerj-12-18497-s011.zip › hucct1/hucct1 Wound Healing nc oe oe+Ca2+/oe/k3 b 0h.tif]

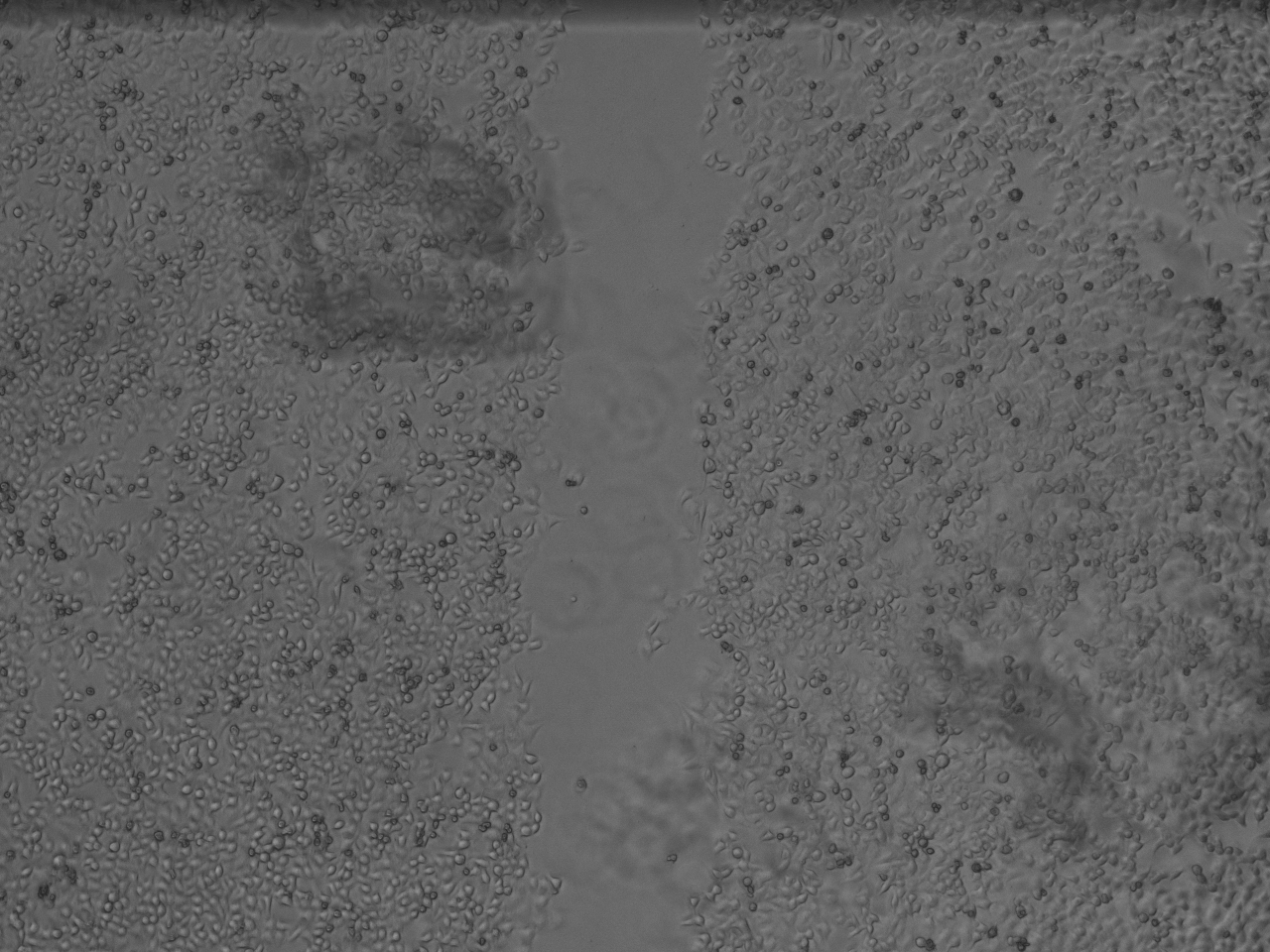

Supplement: Supplemental Information 11 [file peerj-12-18497-s011.zip › hucct1/hucct1 Wound Healing nc oe oe+Ca2+/oe/k3 b 24h.tif]

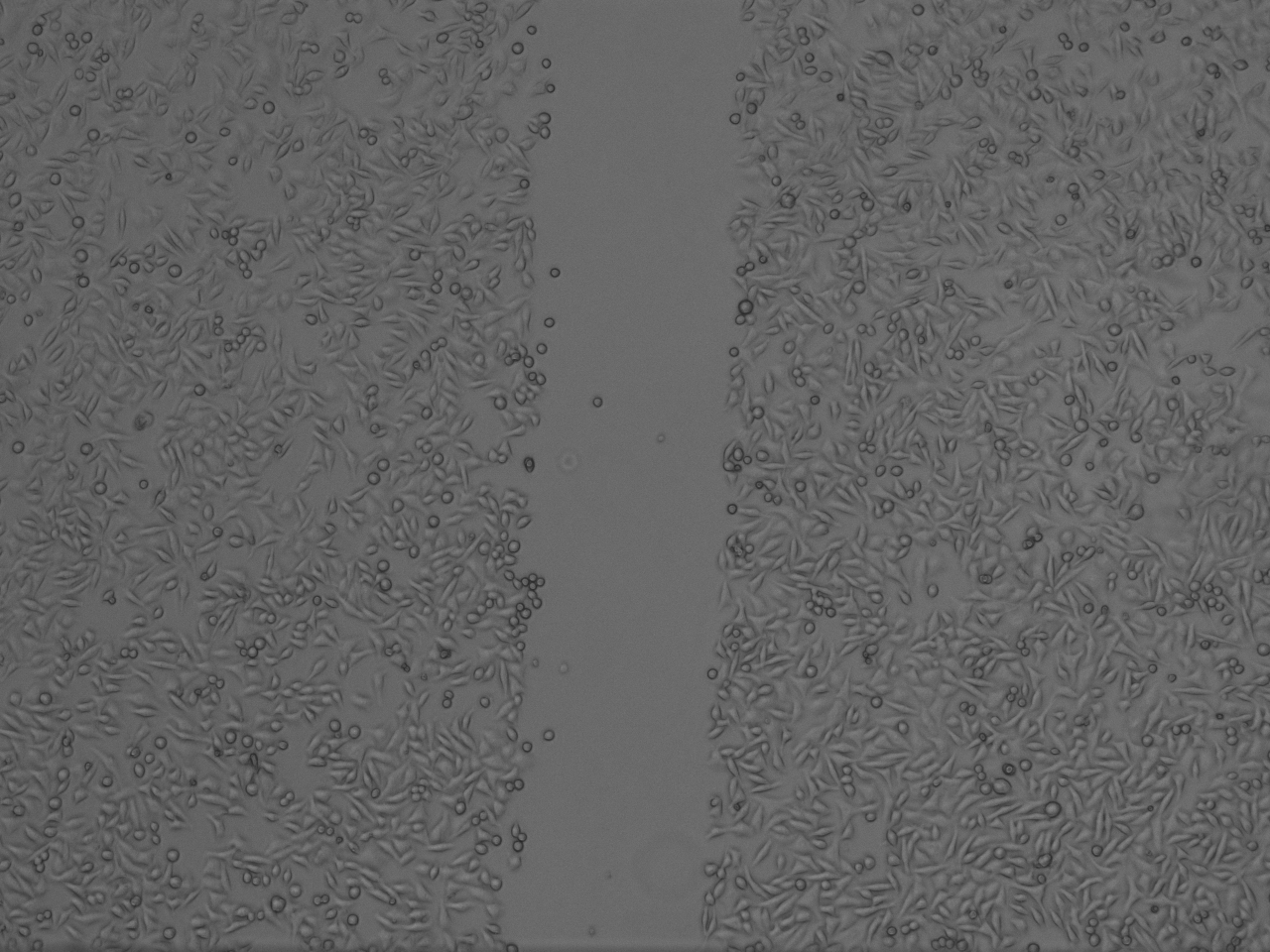

Supplement: Supplemental Information 11 [file peerj-12-18497-s011.zip › hucct1/hucct1 Wound Healing nc oe oe+Ca2+/oe/k3 c 0h.tif]

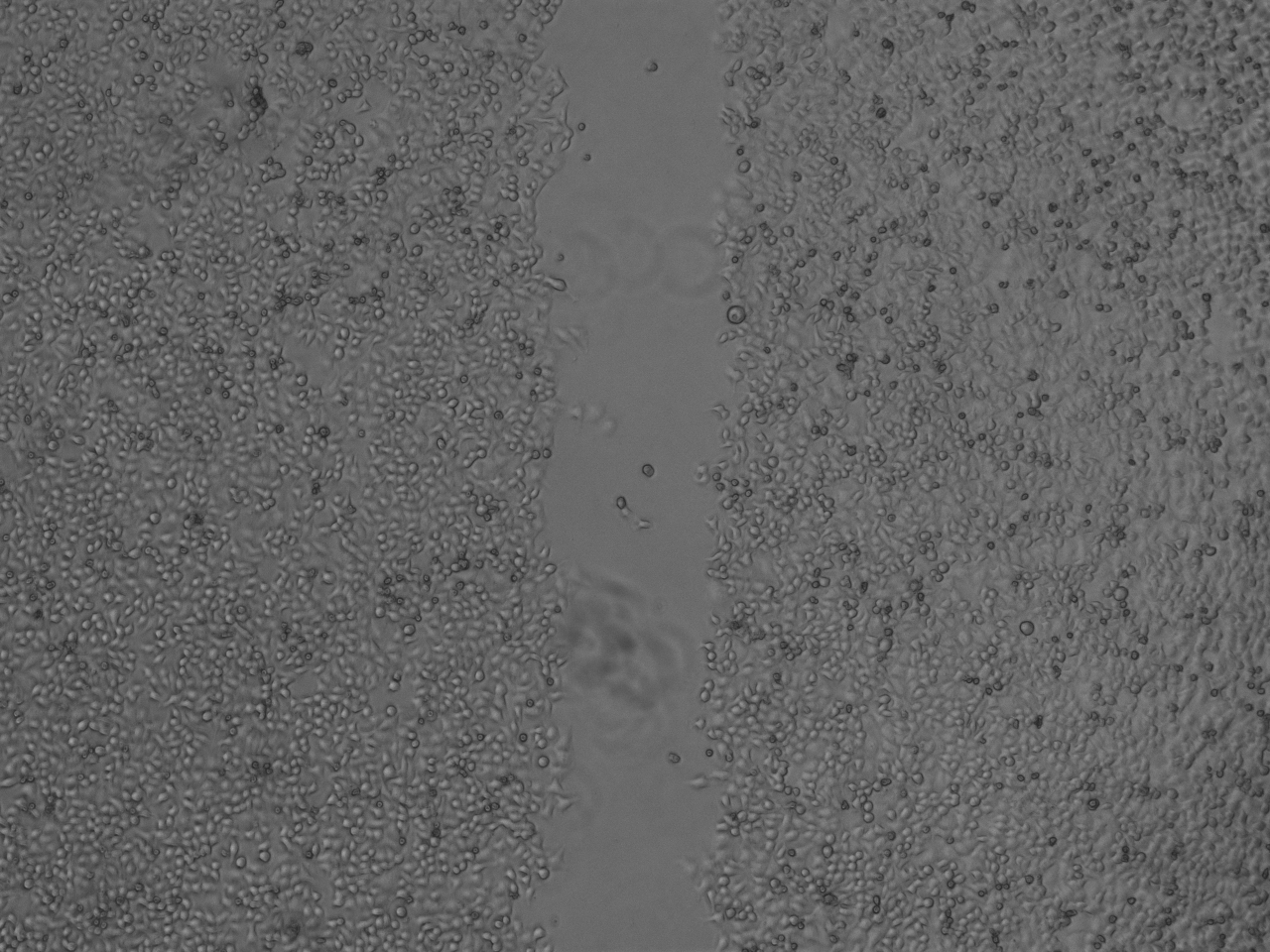

Supplement: Supplemental Information 11 [file peerj-12-18497-s011.zip › hucct1/hucct1 Wound Healing nc oe oe+Ca2+/oe/k3 c 24h.tif]

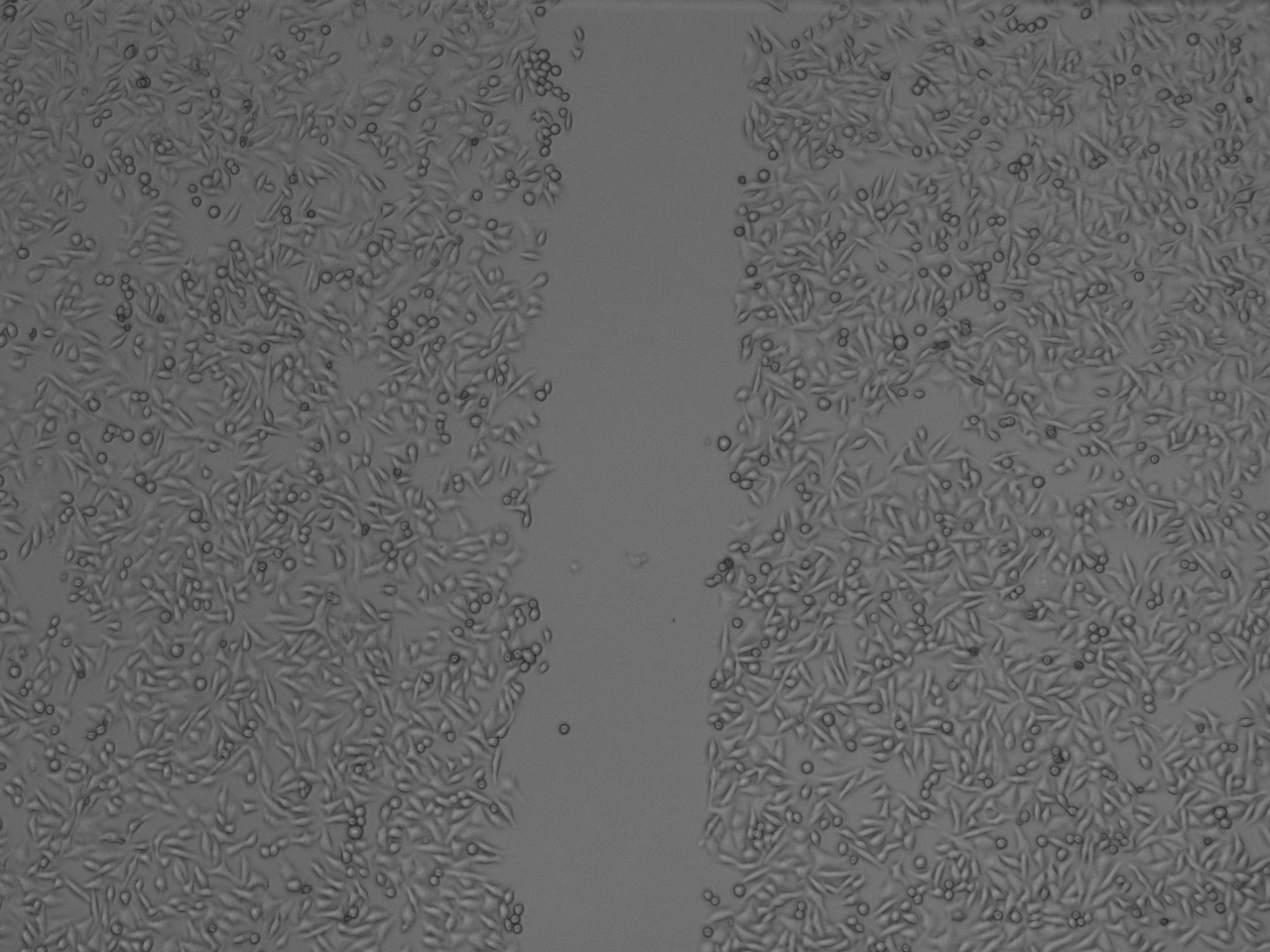

Supplement: Supplemental Information 11 [file peerj-12-18497-s011.zip › hucct1/hucct1 Wound Healing nc oe oe+Ca2+/oe/k3 d 0h.tif]

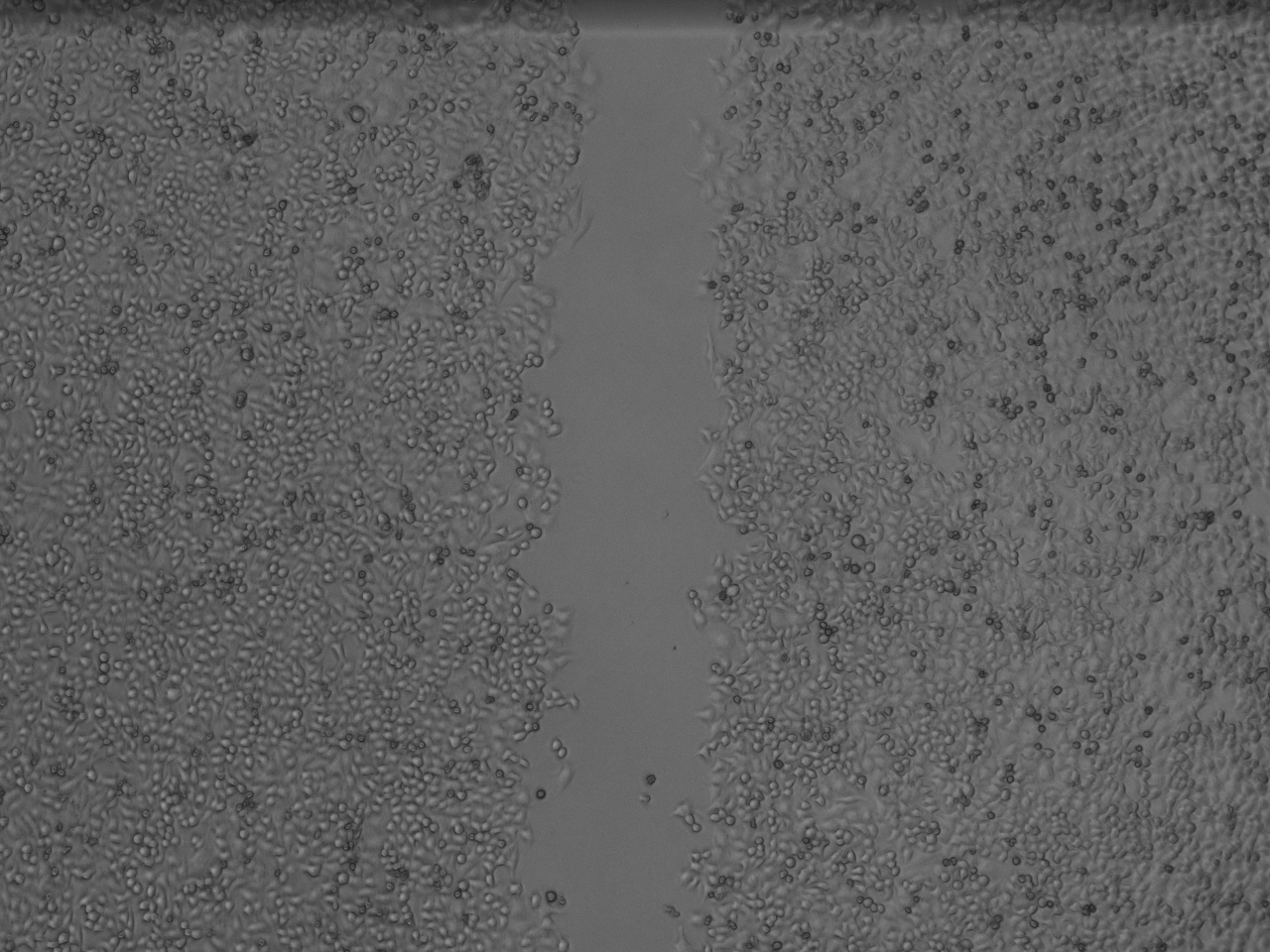

Supplement: Supplemental Information 11 [file peerj-12-18497-s011.zip › hucct1/hucct1 Wound Healing nc oe oe+Ca2+/oe/k3 d 24h.tif]

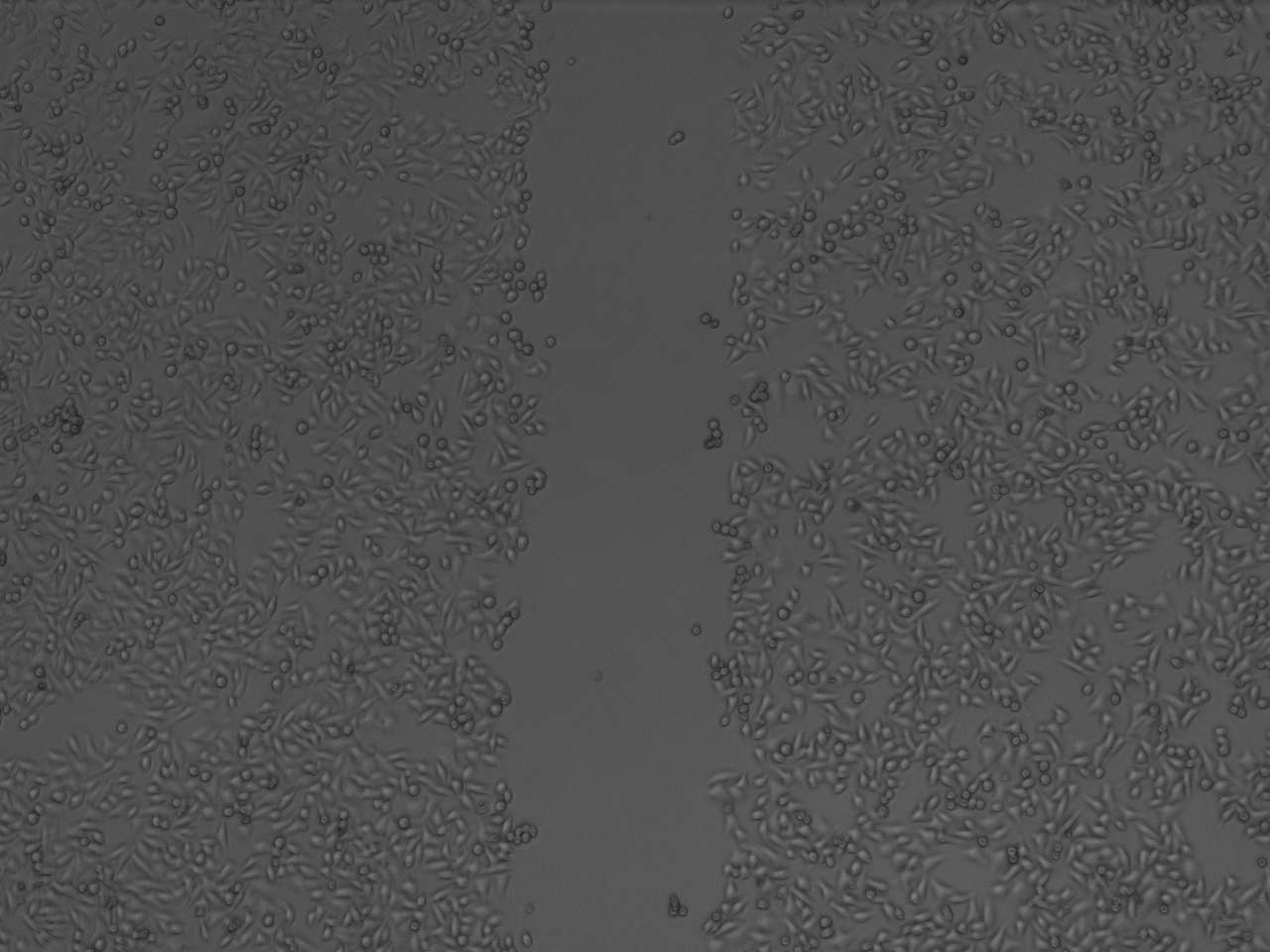

Supplement: Supplemental Information 11 [file peerj-12-18497-s011.zip › hucct1/hucct1 Wound Healing nc oe oe+Ca2+/oe+Ca2+/k5 b 0h.tif]

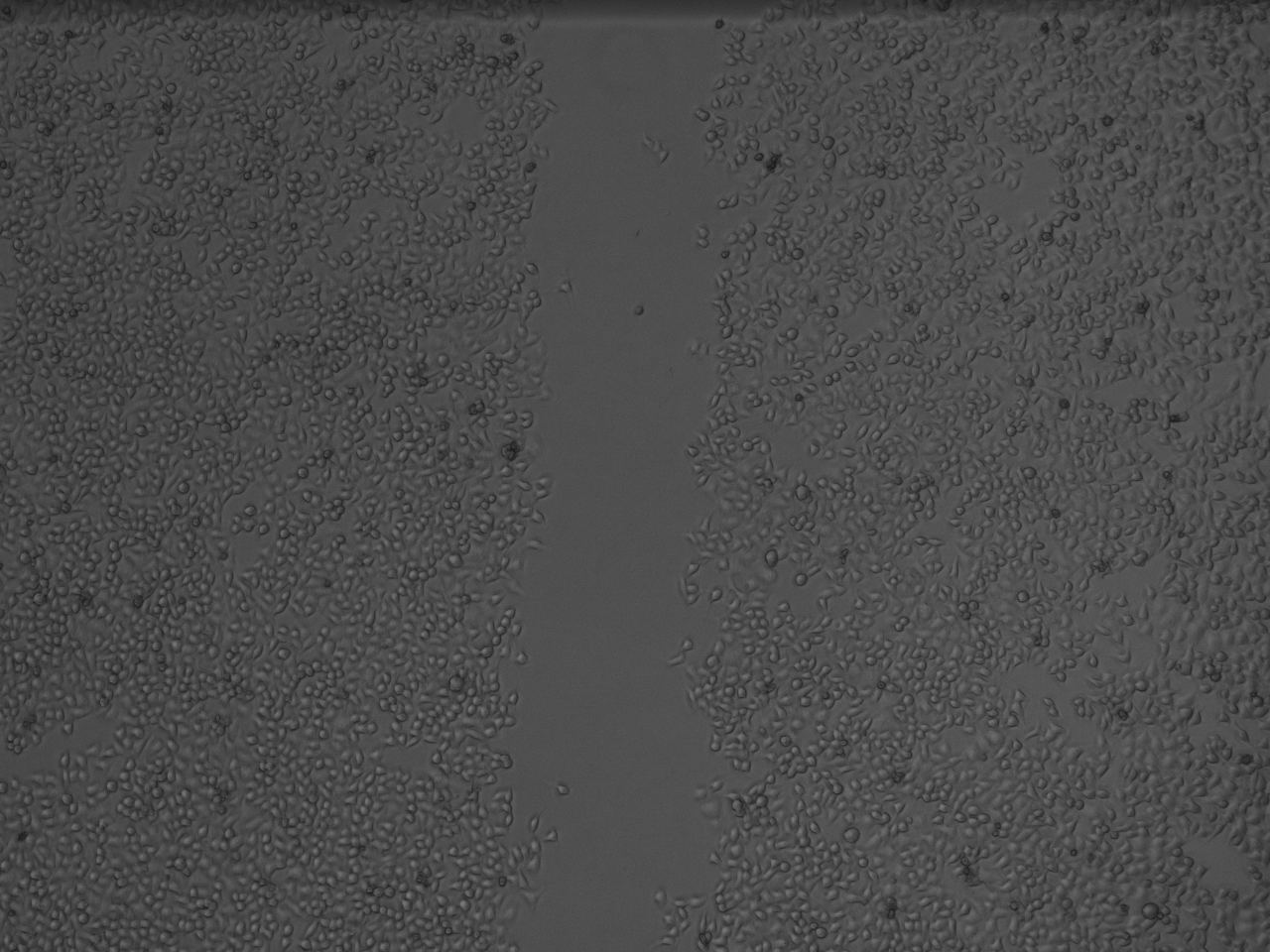

Supplement: Supplemental Information 11 [file peerj-12-18497-s011.zip › hucct1/hucct1 Wound Healing nc oe oe+Ca2+/oe+Ca2+/k5 b 24h.tif]

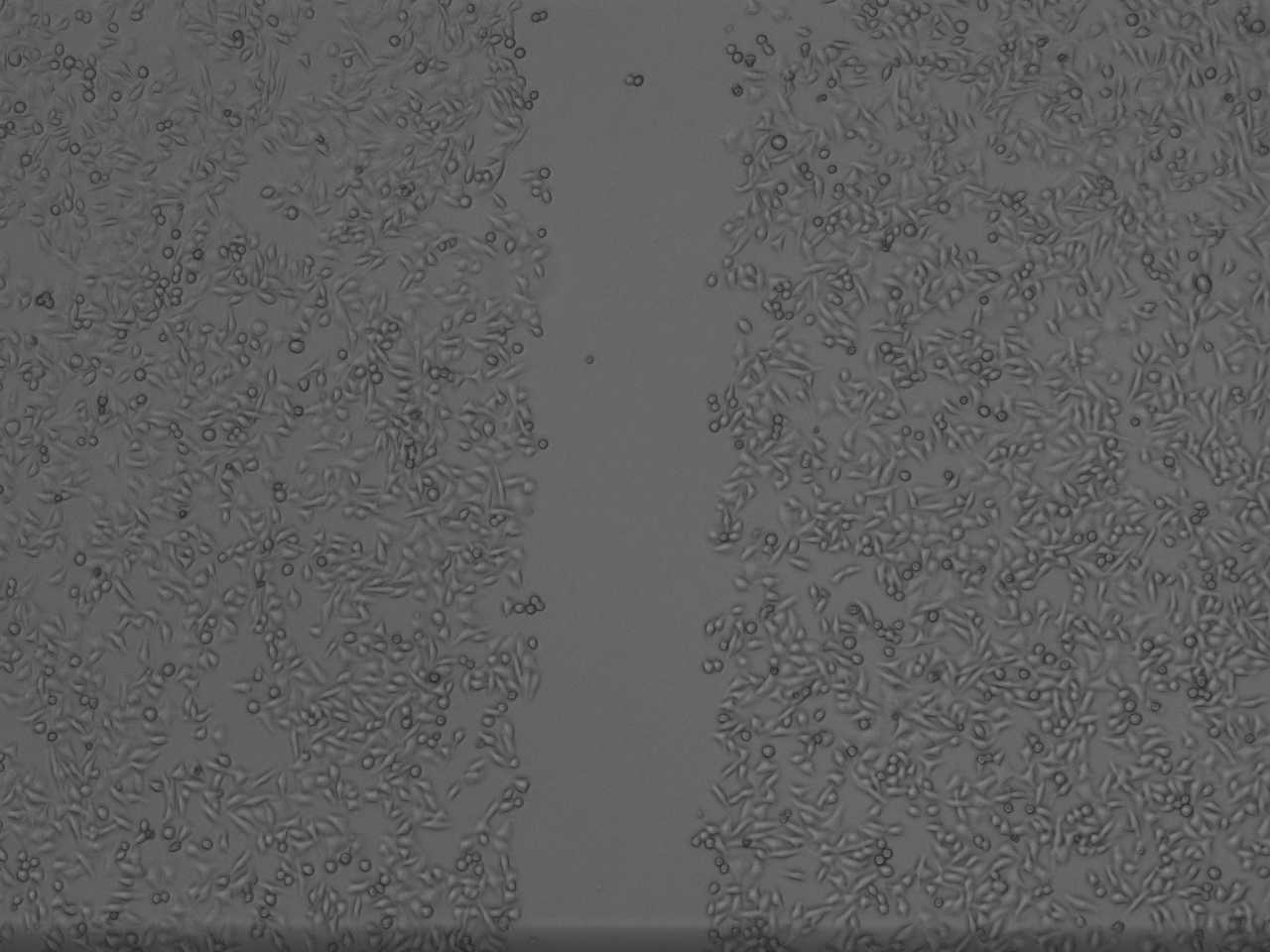

Supplement: Supplemental Information 11 [file peerj-12-18497-s011.zip › hucct1/hucct1 Wound Healing nc oe oe+Ca2+/oe+Ca2+/k5 c 0h.tif]

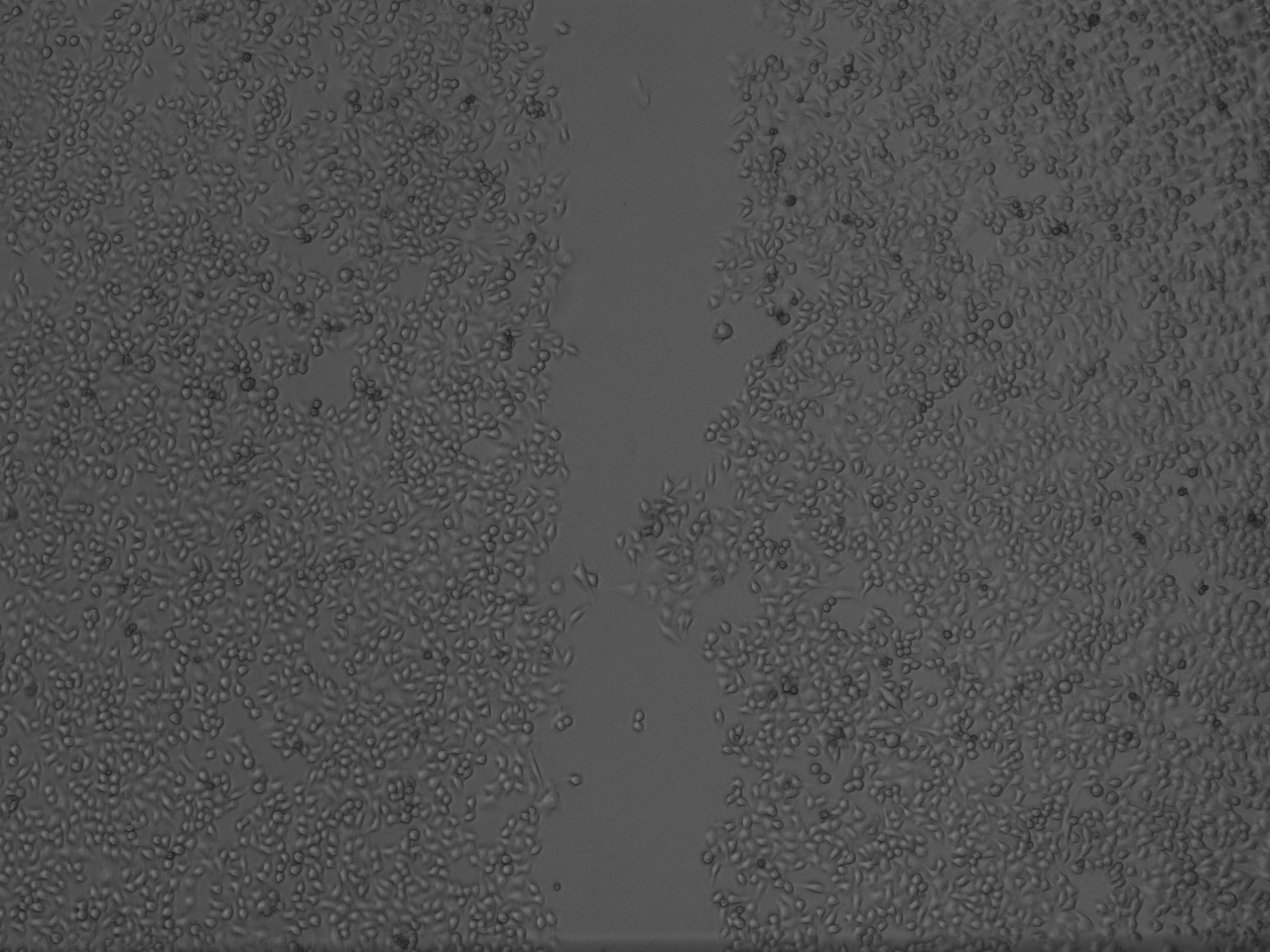

Supplement: Supplemental Information 11 [file peerj-12-18497-s011.zip › hucct1/hucct1 Wound Healing nc oe oe+Ca2+/oe+Ca2+/k5 c 24h.tif]

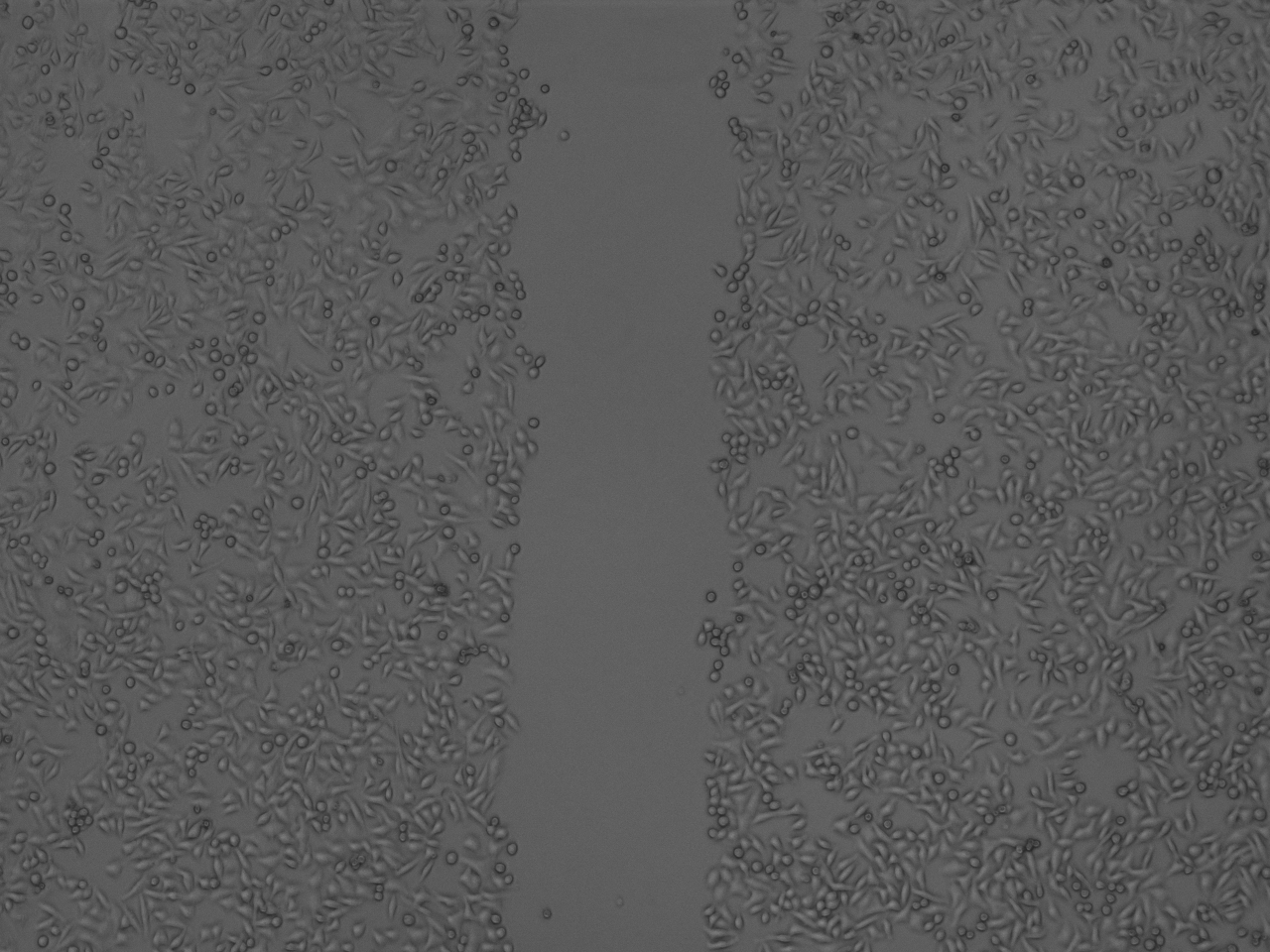

Supplement: Supplemental Information 11 [file peerj-12-18497-s011.zip › hucct1/hucct1 Wound Healing nc oe oe+Ca2+/oe+Ca2+/k5 d 0h.tif]

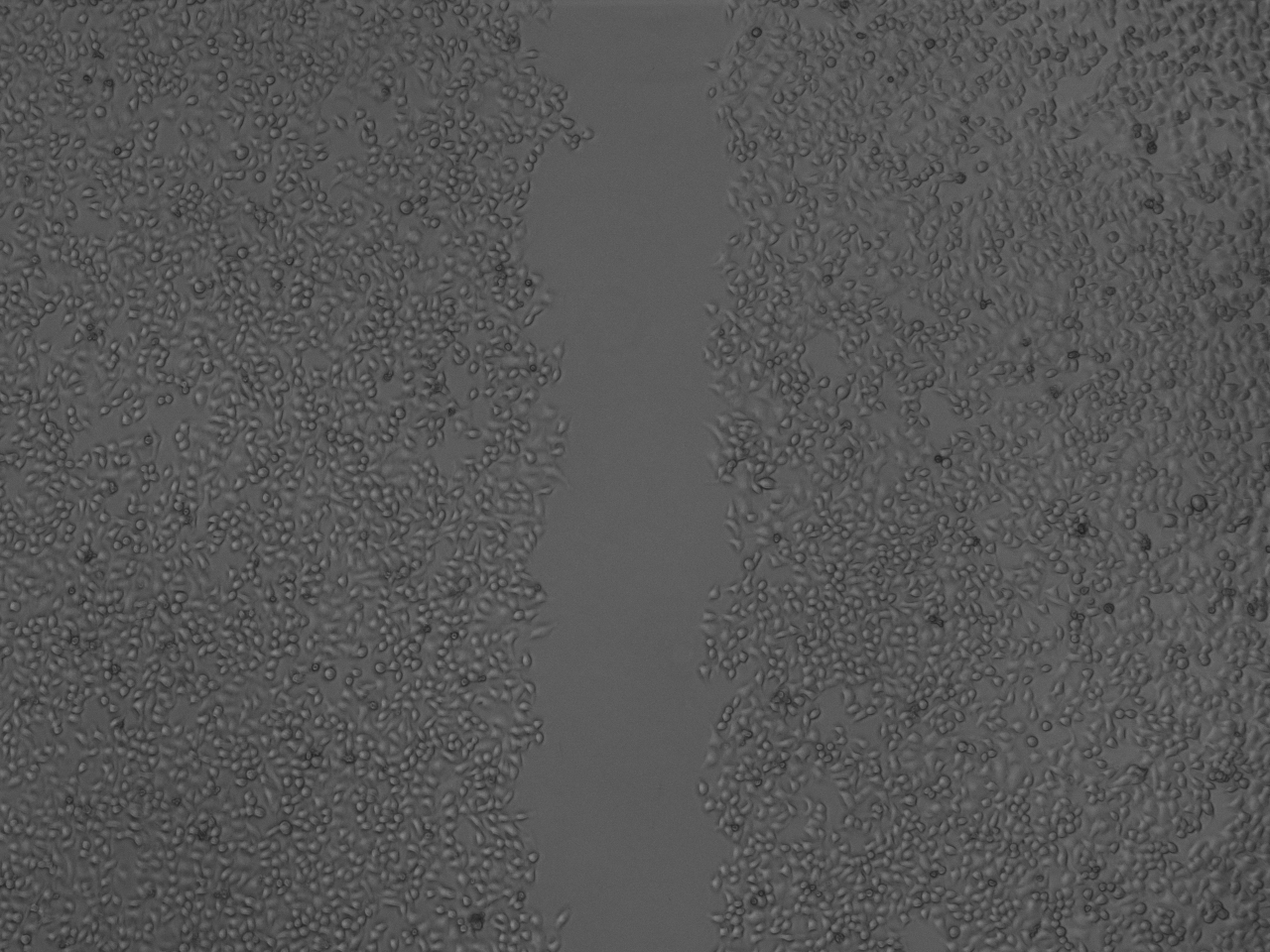

Supplement: Supplemental Information 11 [file peerj-12-18497-s011.zip › hucct1/hucct1 Wound Healing nc oe oe+Ca2+/oe+Ca2+/k5 d 24h.tif]

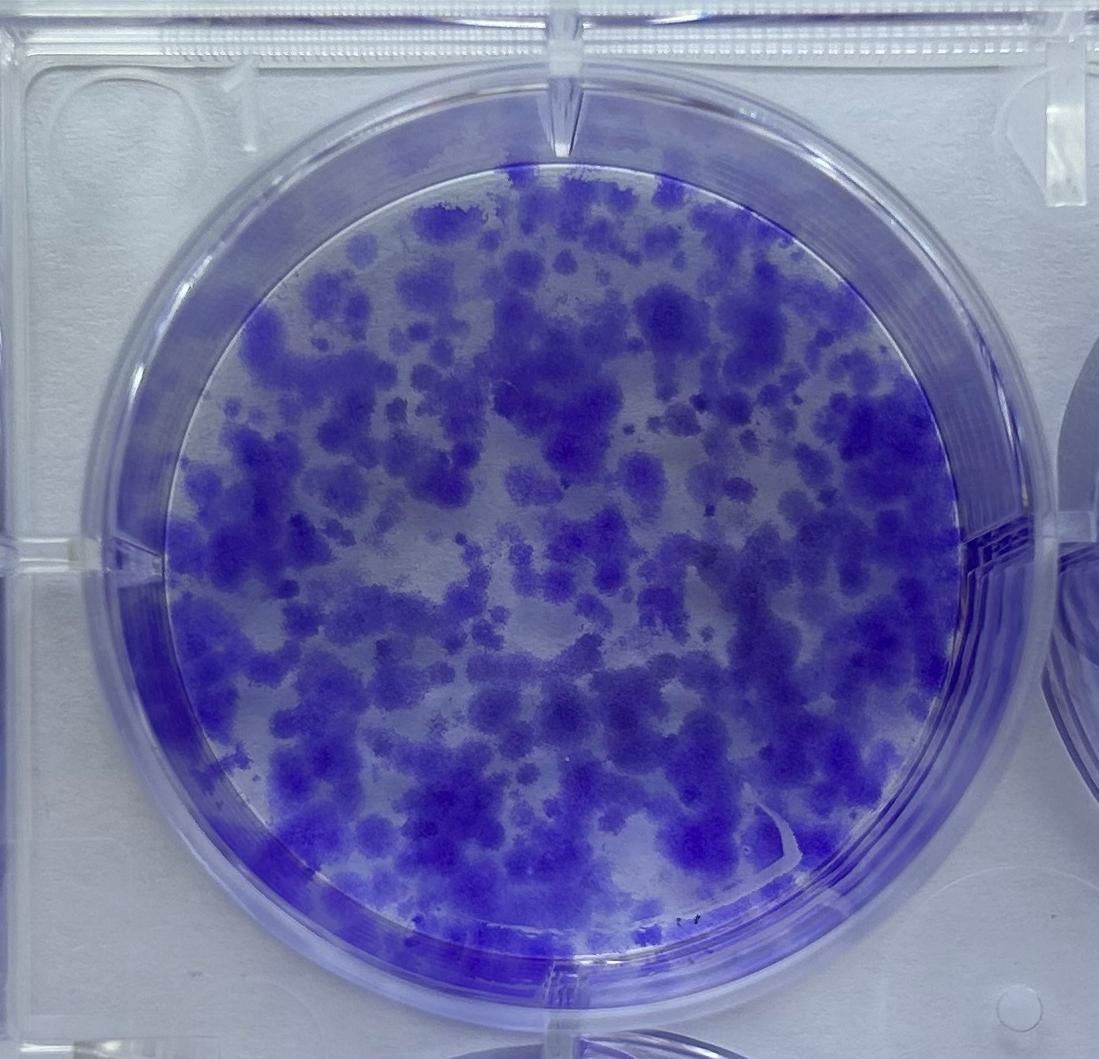

Supplement: Supplemental Information 12 [file peerj-12-18497-s012.zip › qbc939/qbc clone formation nc oe +Ca2+/nc1.jpg]

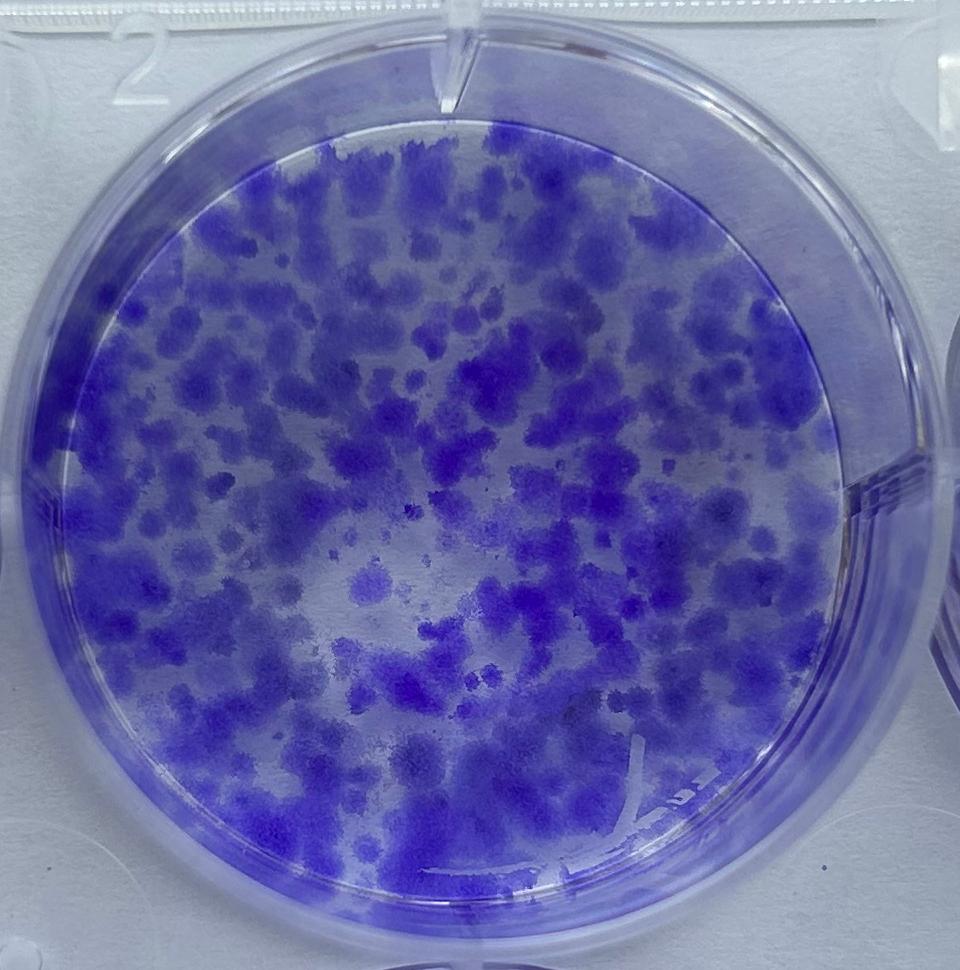

Supplement: Supplemental Information 12 [file peerj-12-18497-s012.zip › qbc939/qbc clone formation nc oe +Ca2+/nc2.jpg]

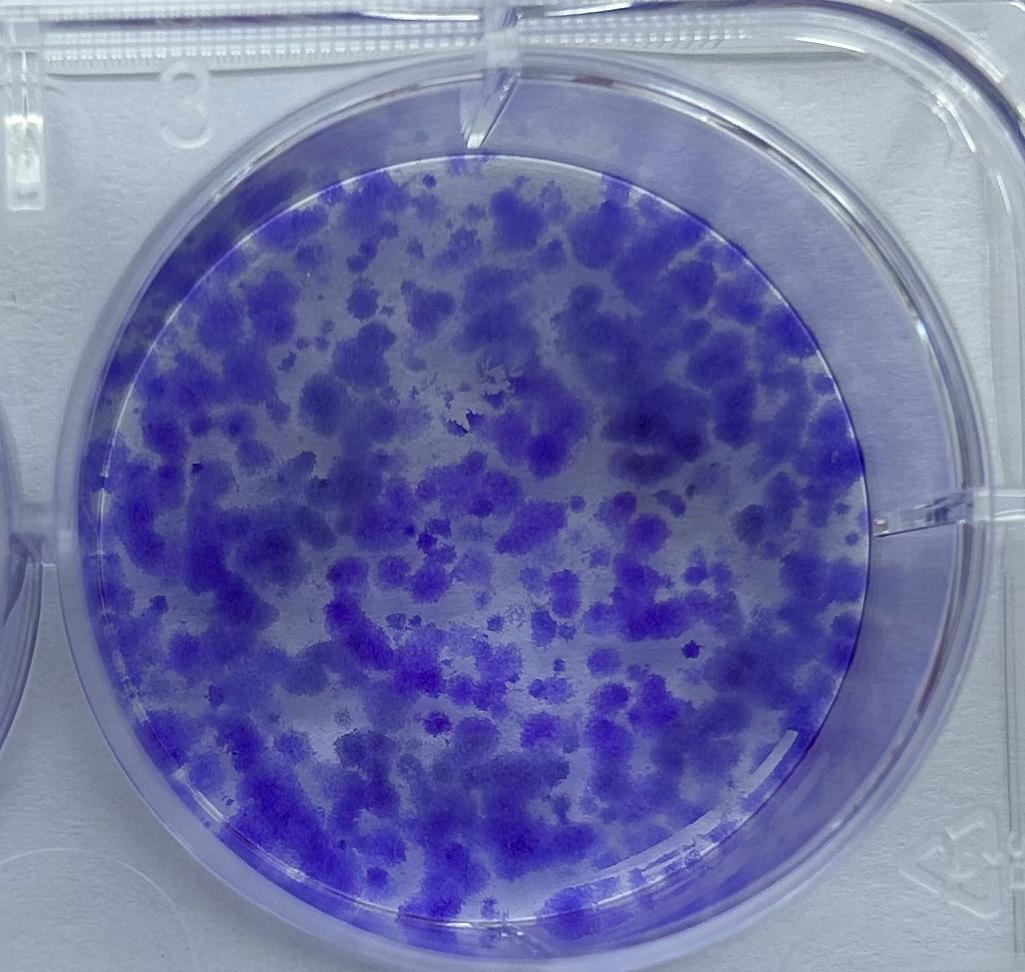

Supplement: Supplemental Information 12 [file peerj-12-18497-s012.zip › qbc939/qbc clone formation nc oe +Ca2+/nc3.jpg]

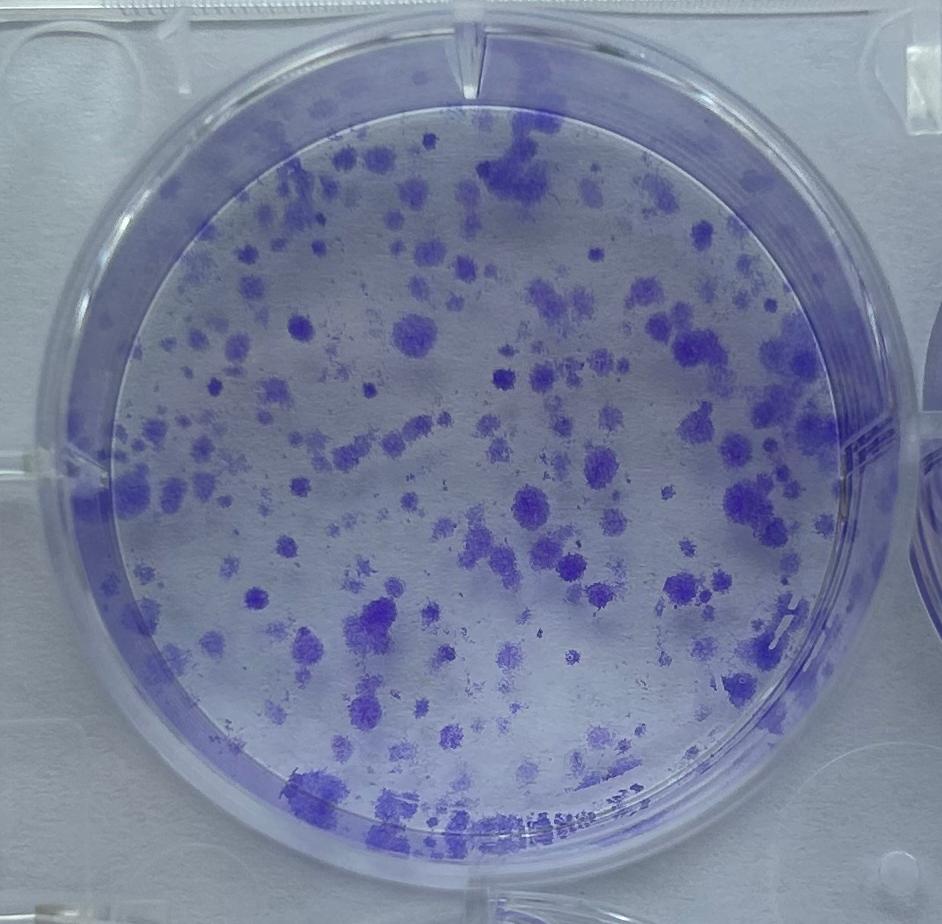

Supplement: Supplemental Information 12 [file peerj-12-18497-s012.zip › qbc939/qbc clone formation nc oe +Ca2+/oe+钙1.jpg]

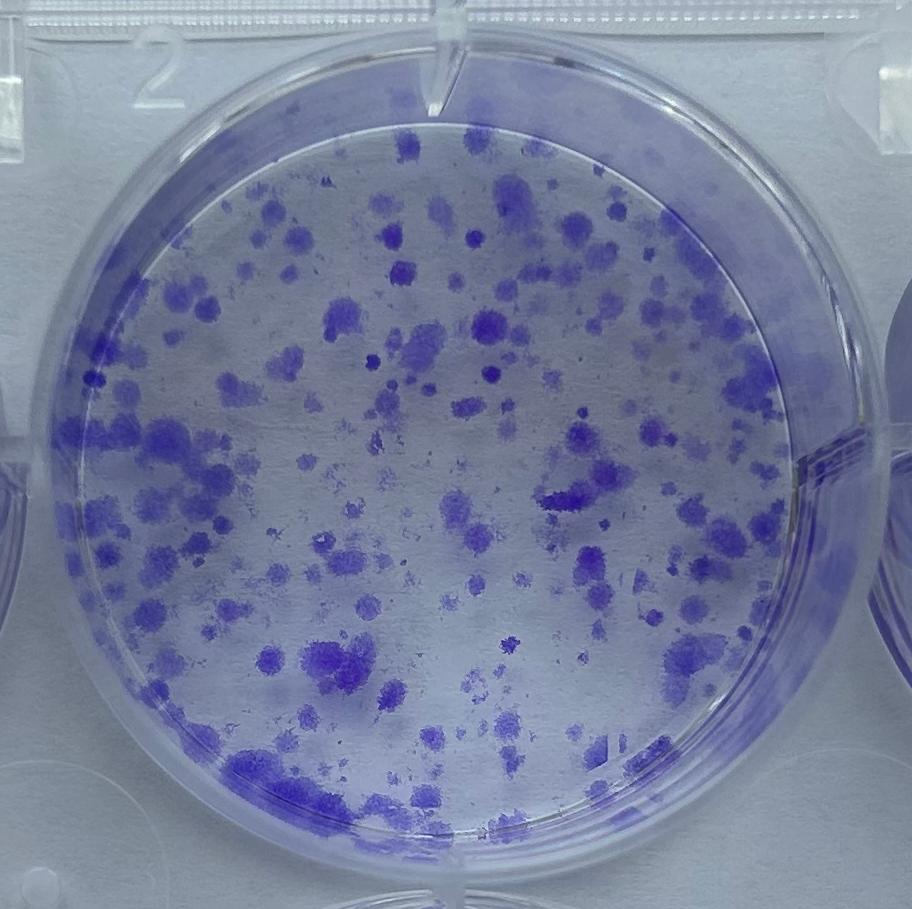

Supplement: Supplemental Information 12 [file peerj-12-18497-s012.zip › qbc939/qbc clone formation nc oe +Ca2+/oe+钙2.jpg]

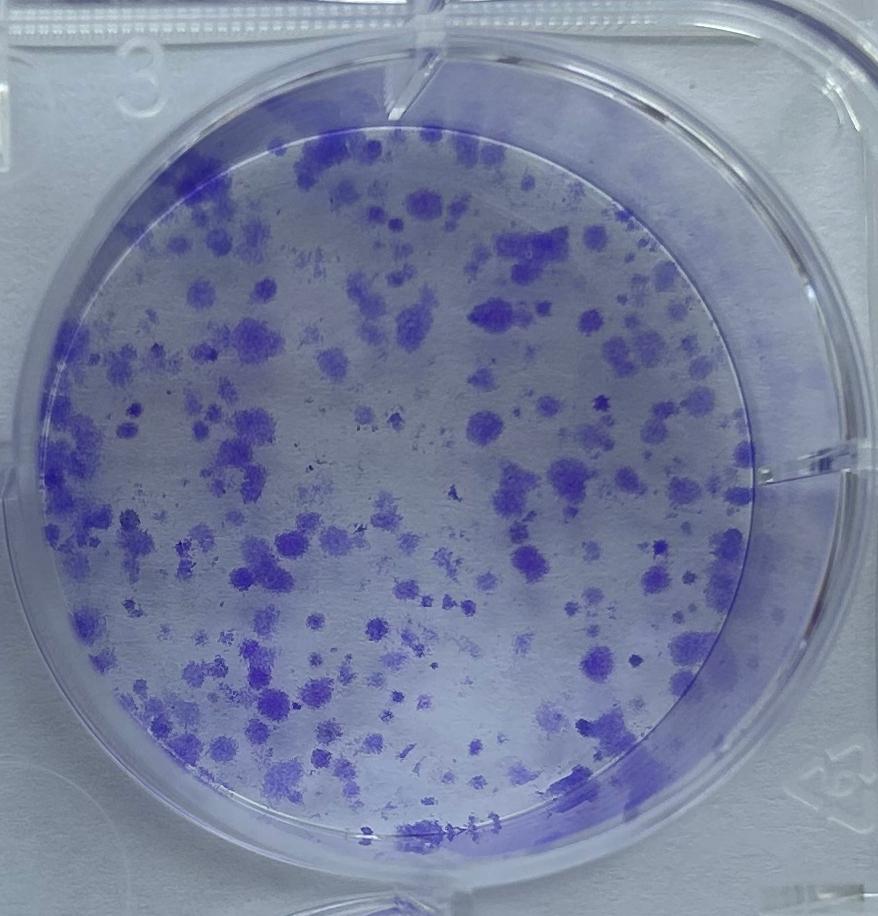

Supplement: Supplemental Information 12 [file peerj-12-18497-s012.zip › qbc939/qbc clone formation nc oe +Ca2+/oe+钙3.jpg]

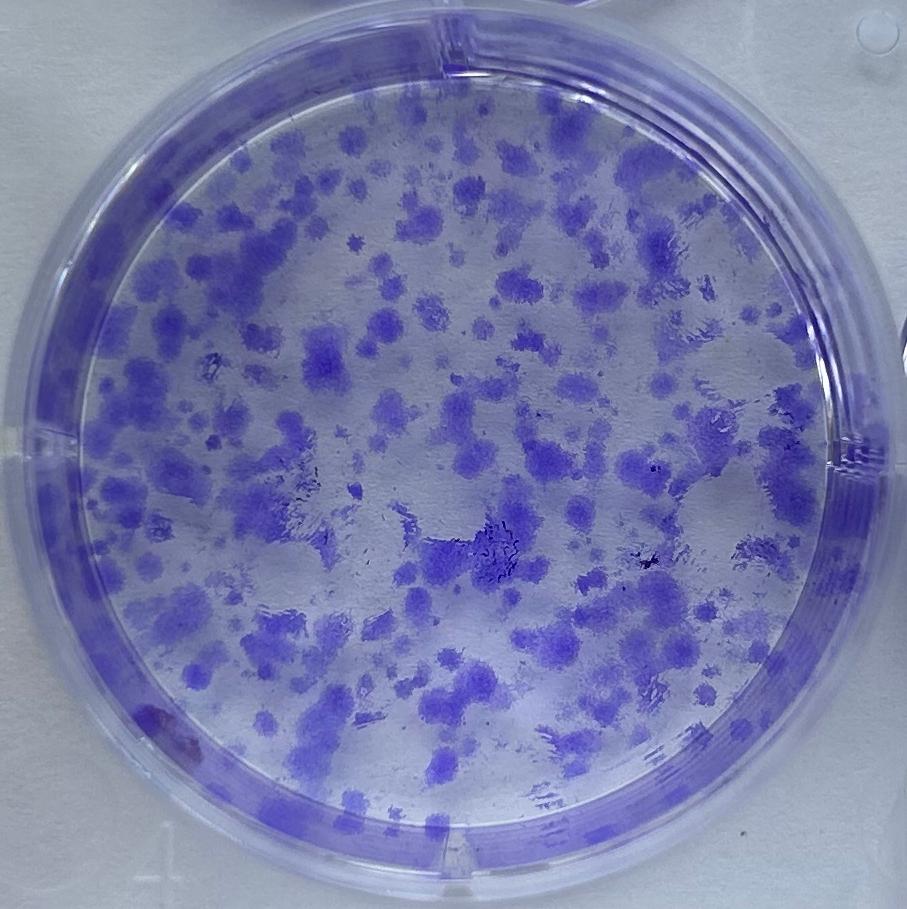

Supplement: Supplemental Information 12 [file peerj-12-18497-s012.zip › qbc939/qbc clone formation nc oe +Ca2+/oe1.jpg]

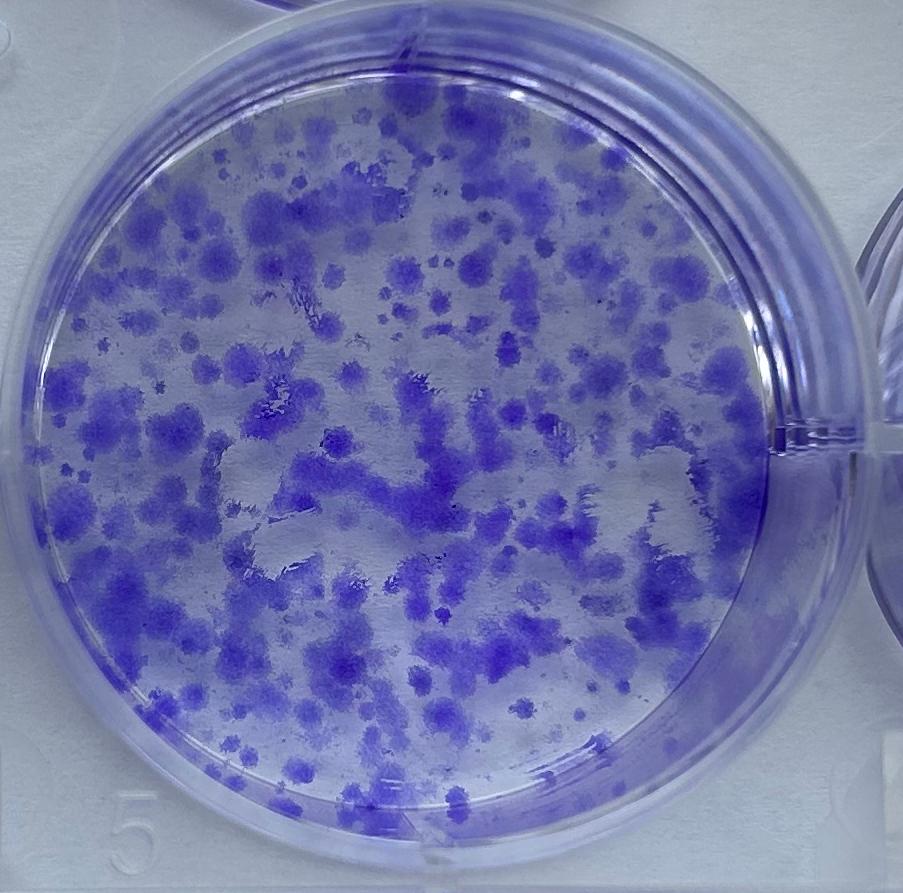

Supplement: Supplemental Information 12 [file peerj-12-18497-s012.zip › qbc939/qbc clone formation nc oe +Ca2+/oe2.jpg]

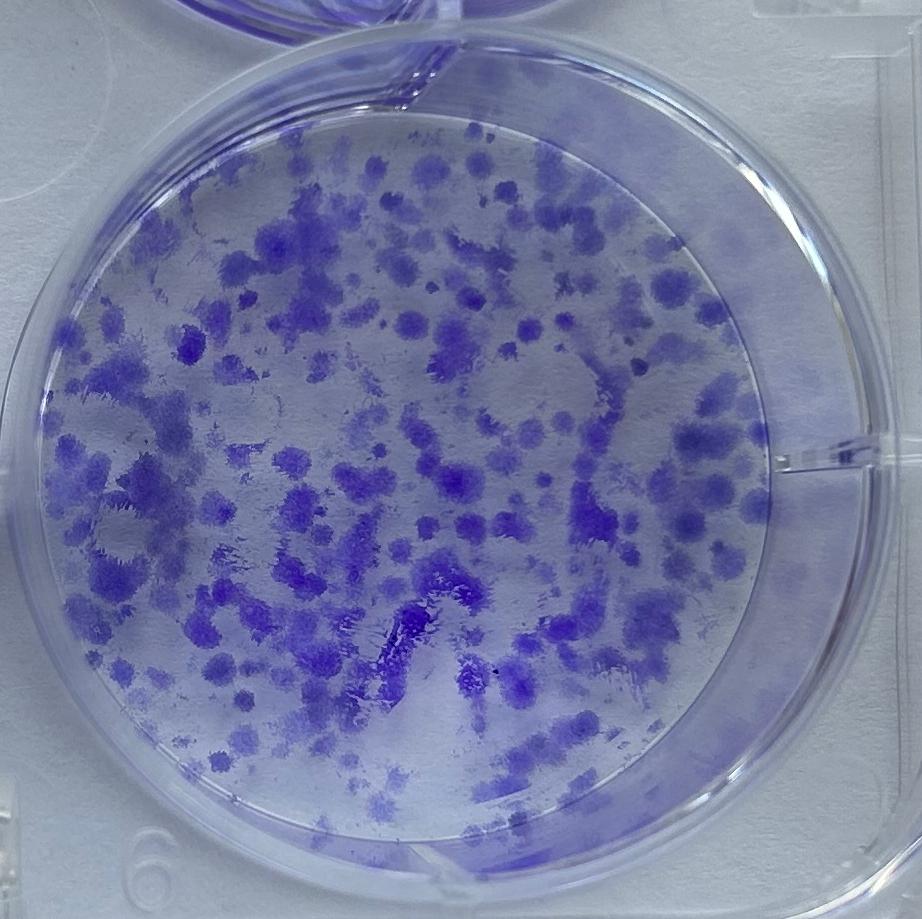

Supplement: Supplemental Information 12 [file peerj-12-18497-s012.zip › qbc939/qbc clone formation nc oe +Ca2+/oe3.jpg]

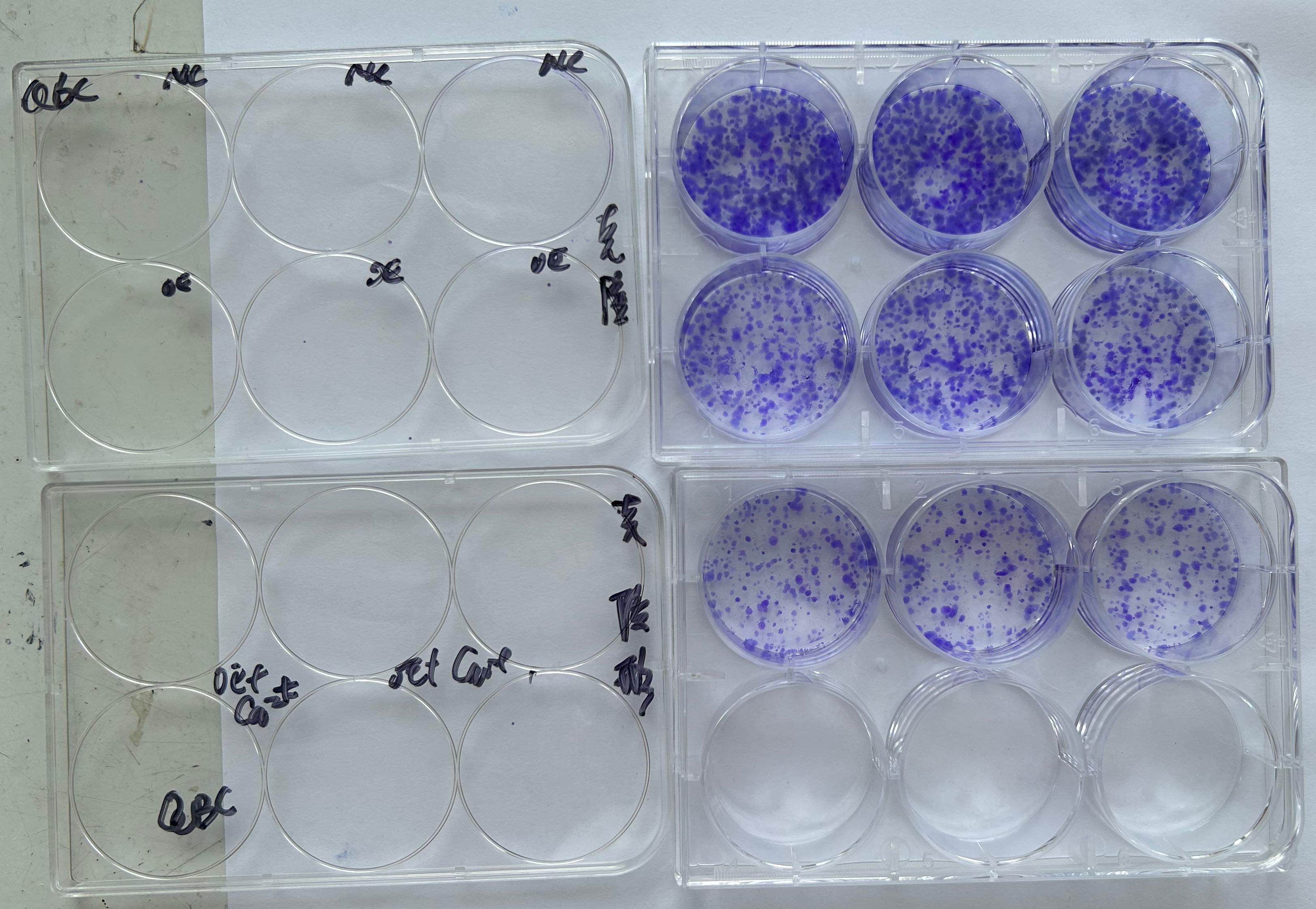

Supplement: Supplemental Information 12 [file peerj-12-18497-s012.zip › qbc939/qbc clone formation nc oe +Ca2+/全1.jpg]

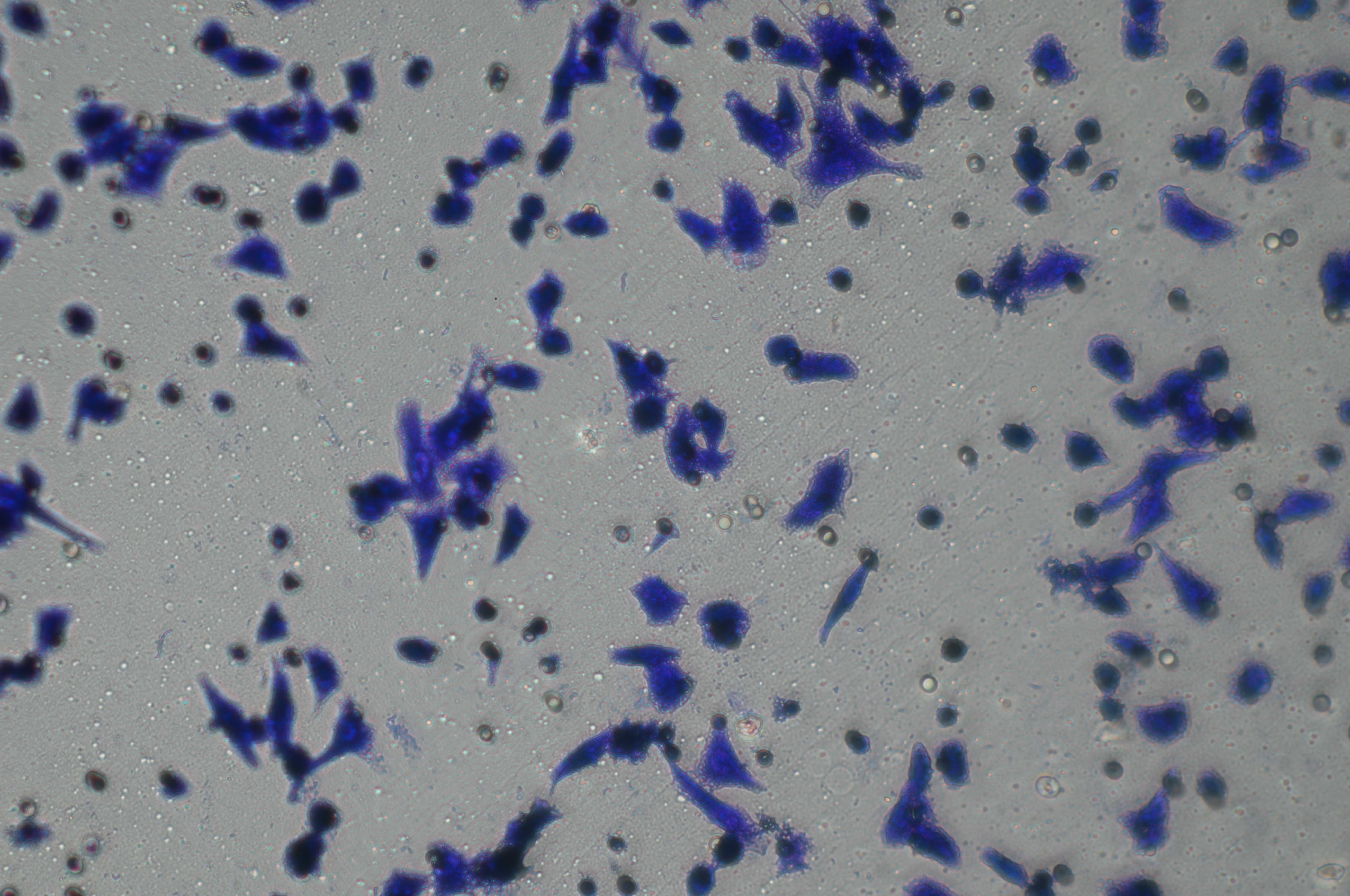

Supplement: Supplemental Information 12 [file peerj-12-18497-s012.zip › qbc939/qbc939 migration nc oe +Ca2+/picture/qbc939 clec3b 孔101.jpg]

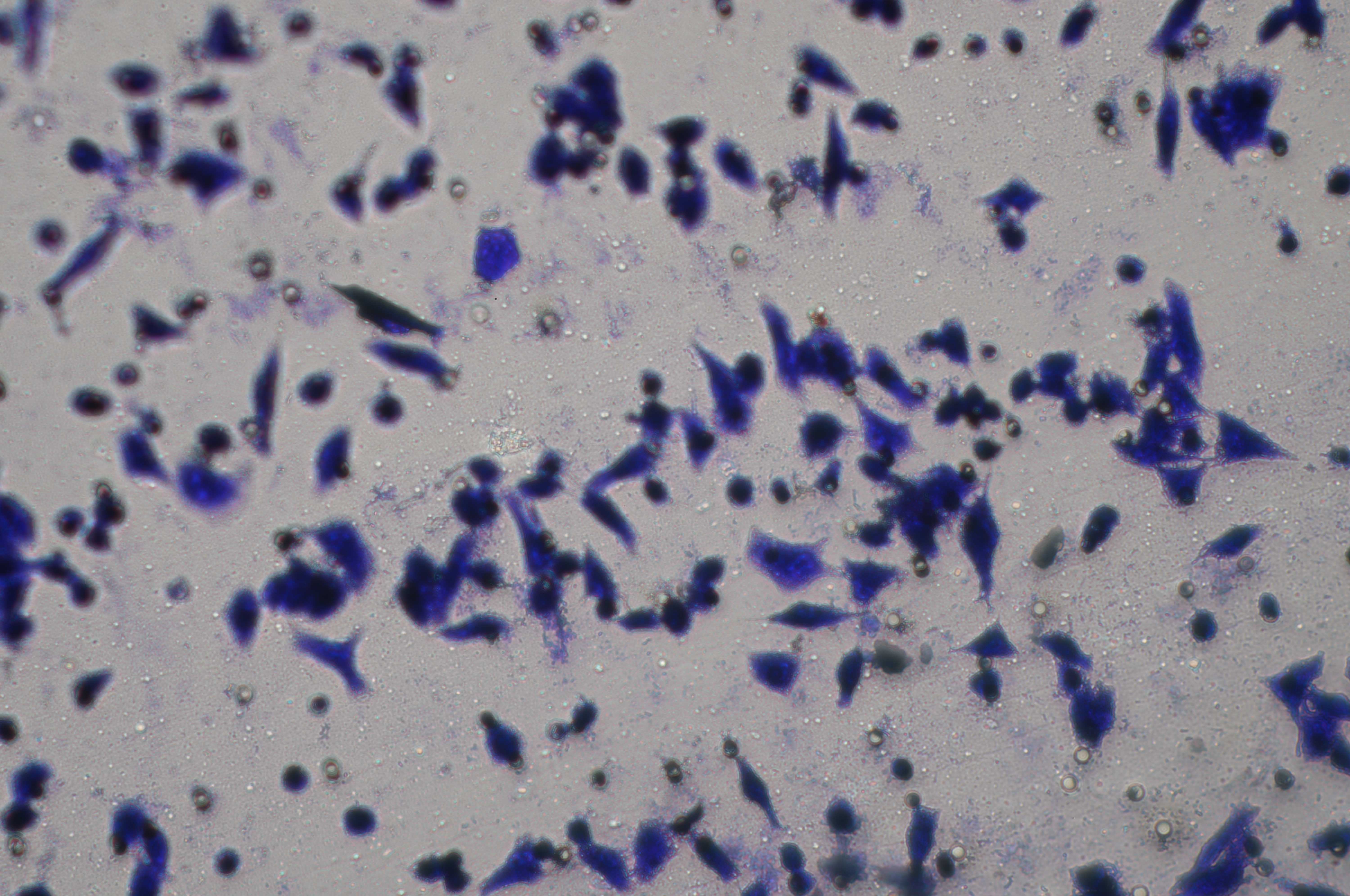

Supplement: Supplemental Information 12 [file peerj-12-18497-s012.zip › qbc939/qbc939 migration nc oe +Ca2+/picture/qbc939 clec3b 孔201.jpg]

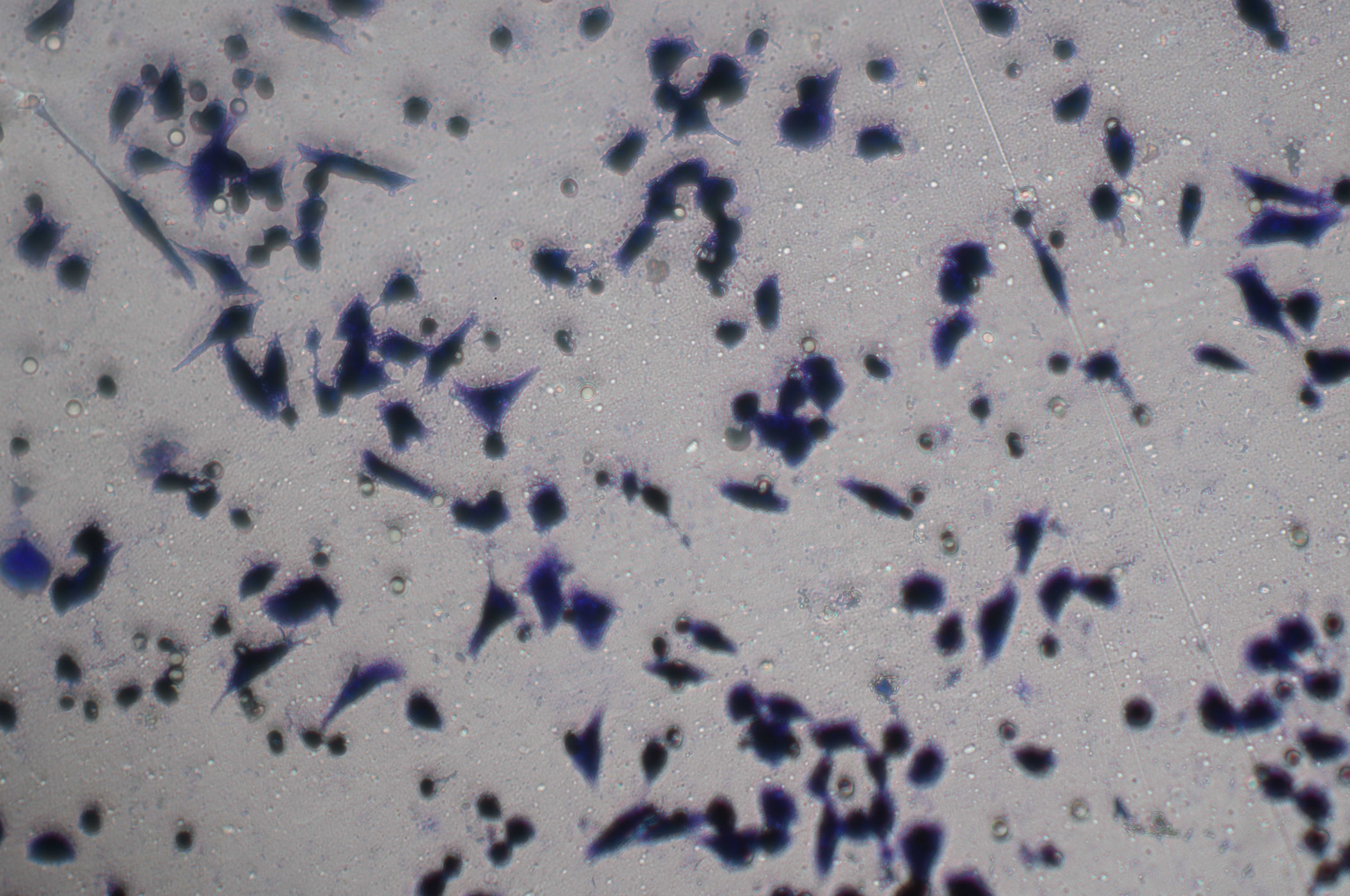

Supplement: Supplemental Information 12 [file peerj-12-18497-s012.zip › qbc939/qbc939 migration nc oe +Ca2+/picture/qbc939 clec3b 孔302.jpg]

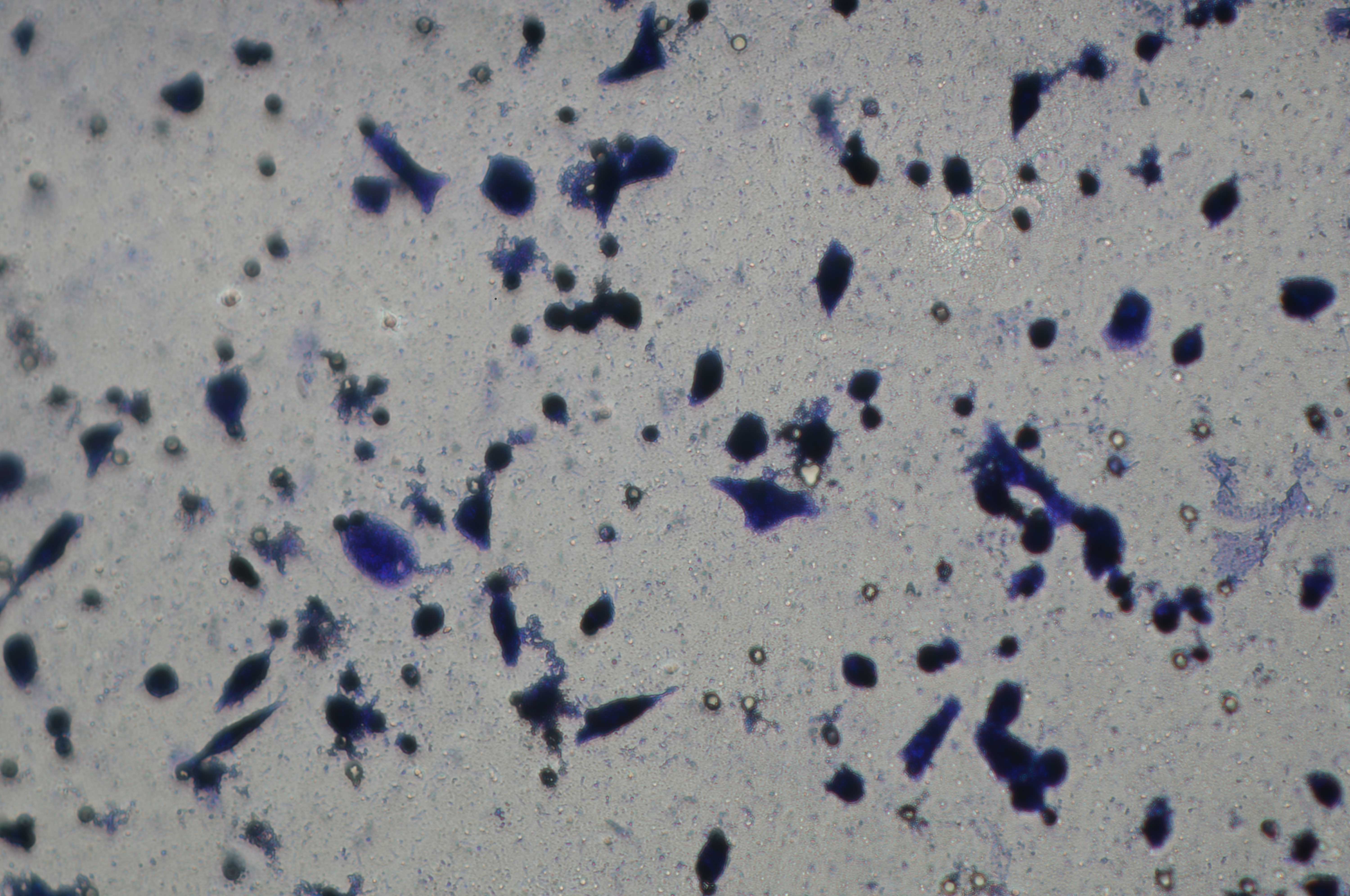

Supplement: Supplemental Information 12 [file peerj-12-18497-s012.zip › qbc939/qbc939 migration nc oe +Ca2+/picture/qbc939 clec3b加Ca2+ 孔101.jpg]

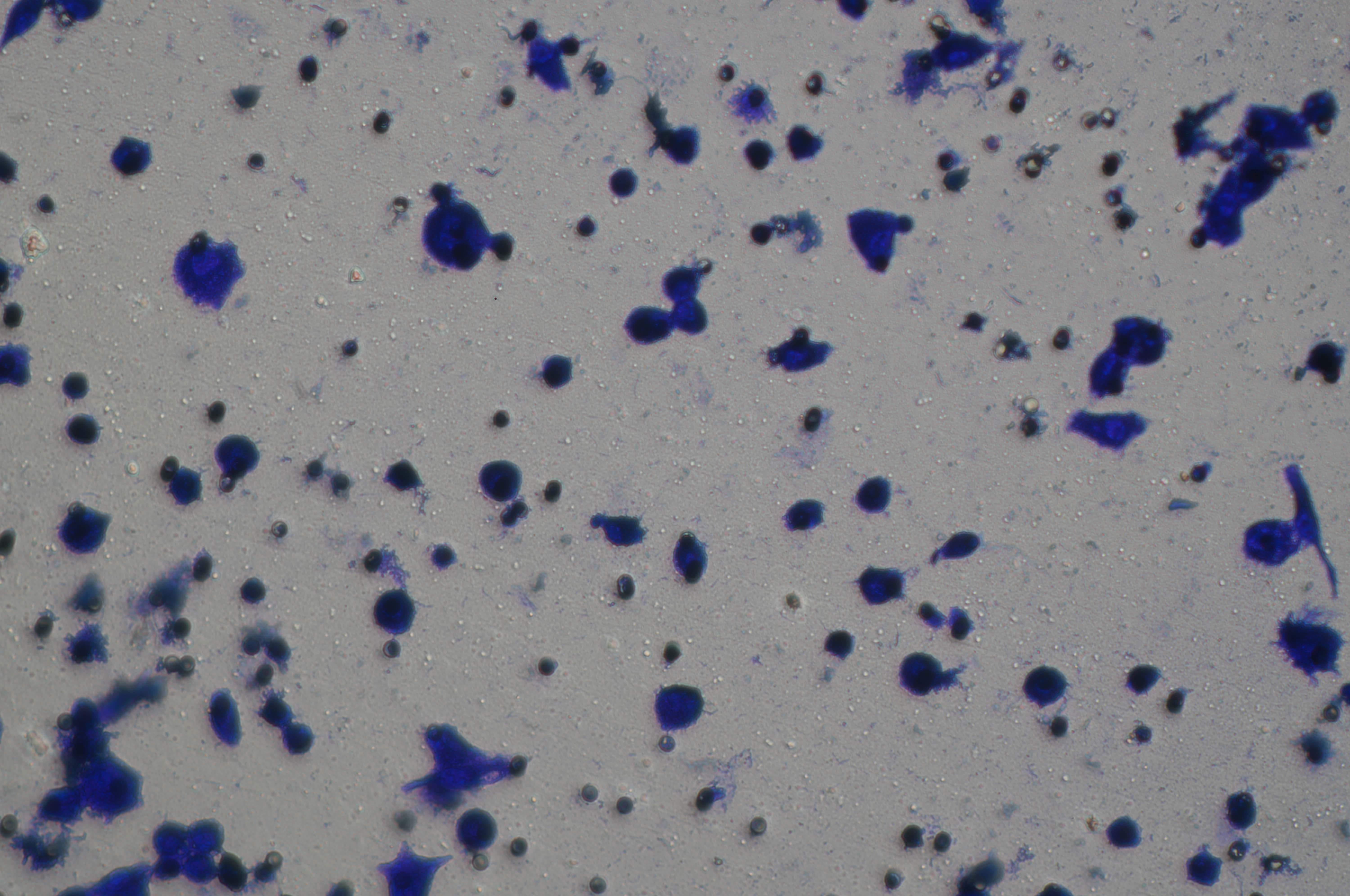

Supplement: Supplemental Information 12 [file peerj-12-18497-s012.zip › qbc939/qbc939 migration nc oe +Ca2+/picture/qbc939 clec3b加Ca2+ 孔201.jpg]

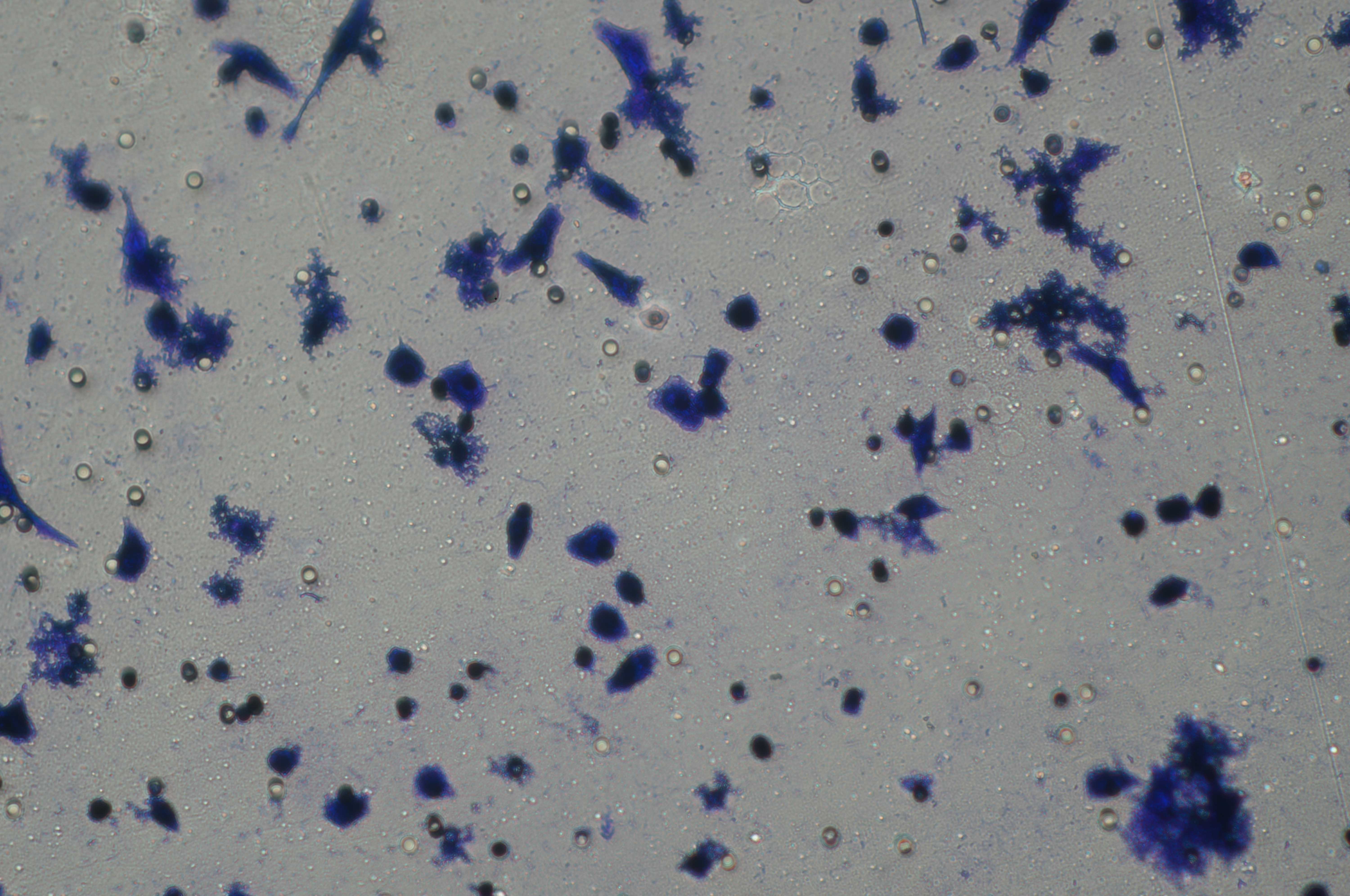

Supplement: Supplemental Information 12 [file peerj-12-18497-s012.zip › qbc939/qbc939 migration nc oe +Ca2+/picture/qbc939 clec3b加Ca2+ 孔302.jpg]

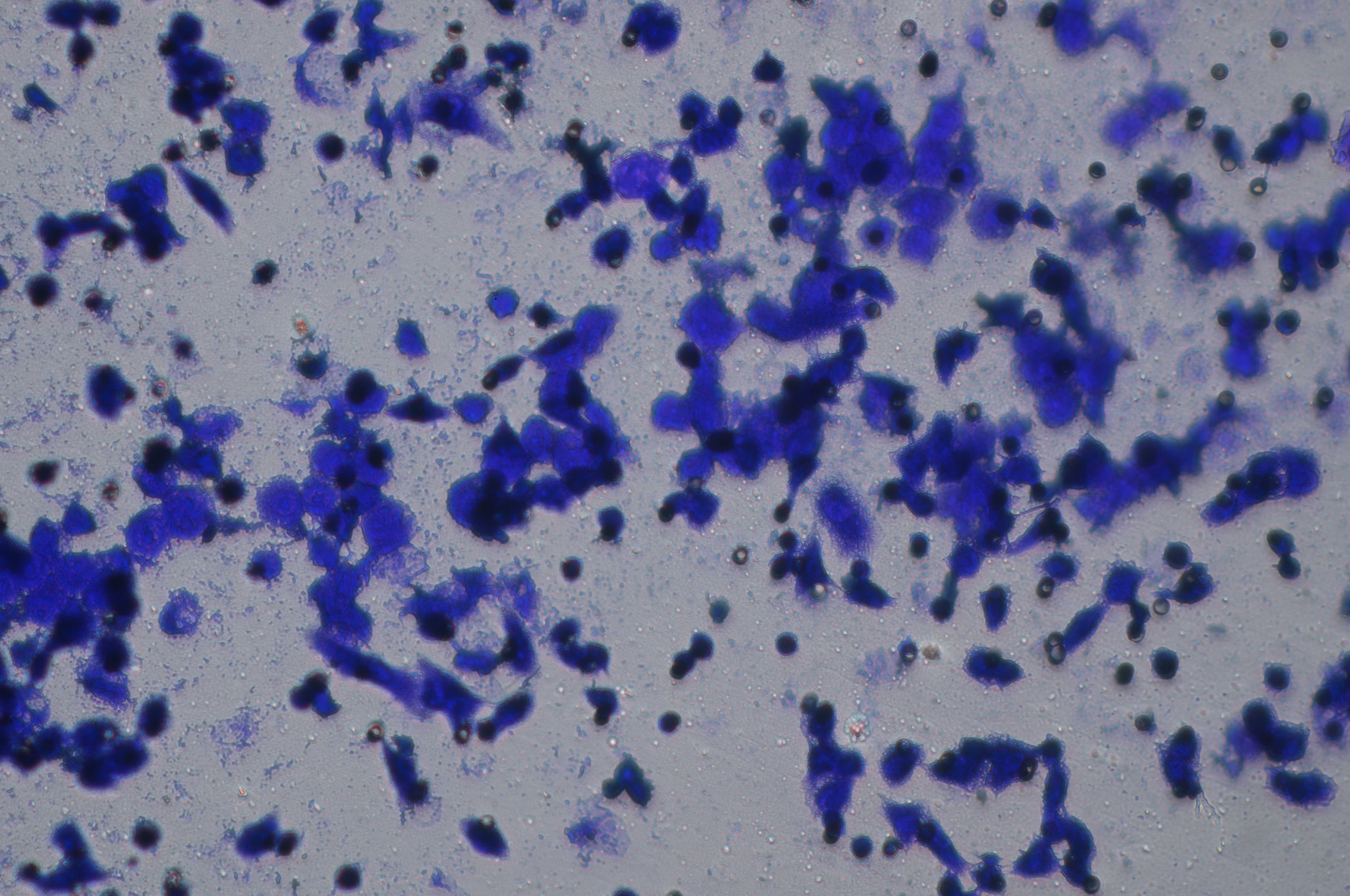

Supplement: Supplemental Information 12 [file peerj-12-18497-s012.zip › qbc939/qbc939 migration nc oe +Ca2+/picture/qbc939 nc 孔101.jpg]

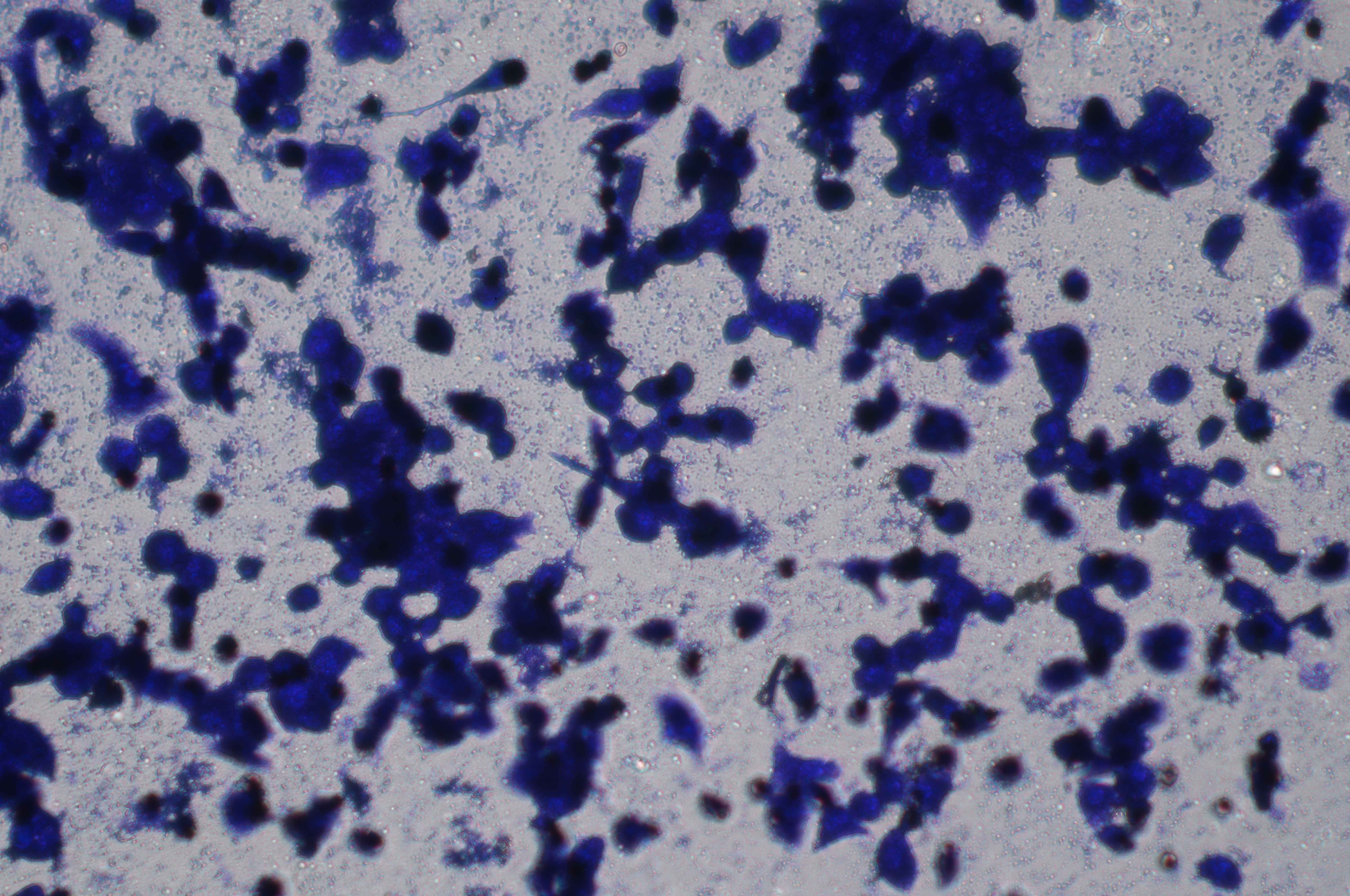

Supplement: Supplemental Information 12 [file peerj-12-18497-s012.zip › qbc939/qbc939 migration nc oe +Ca2+/picture/qbc939 nc 孔204.jpg]

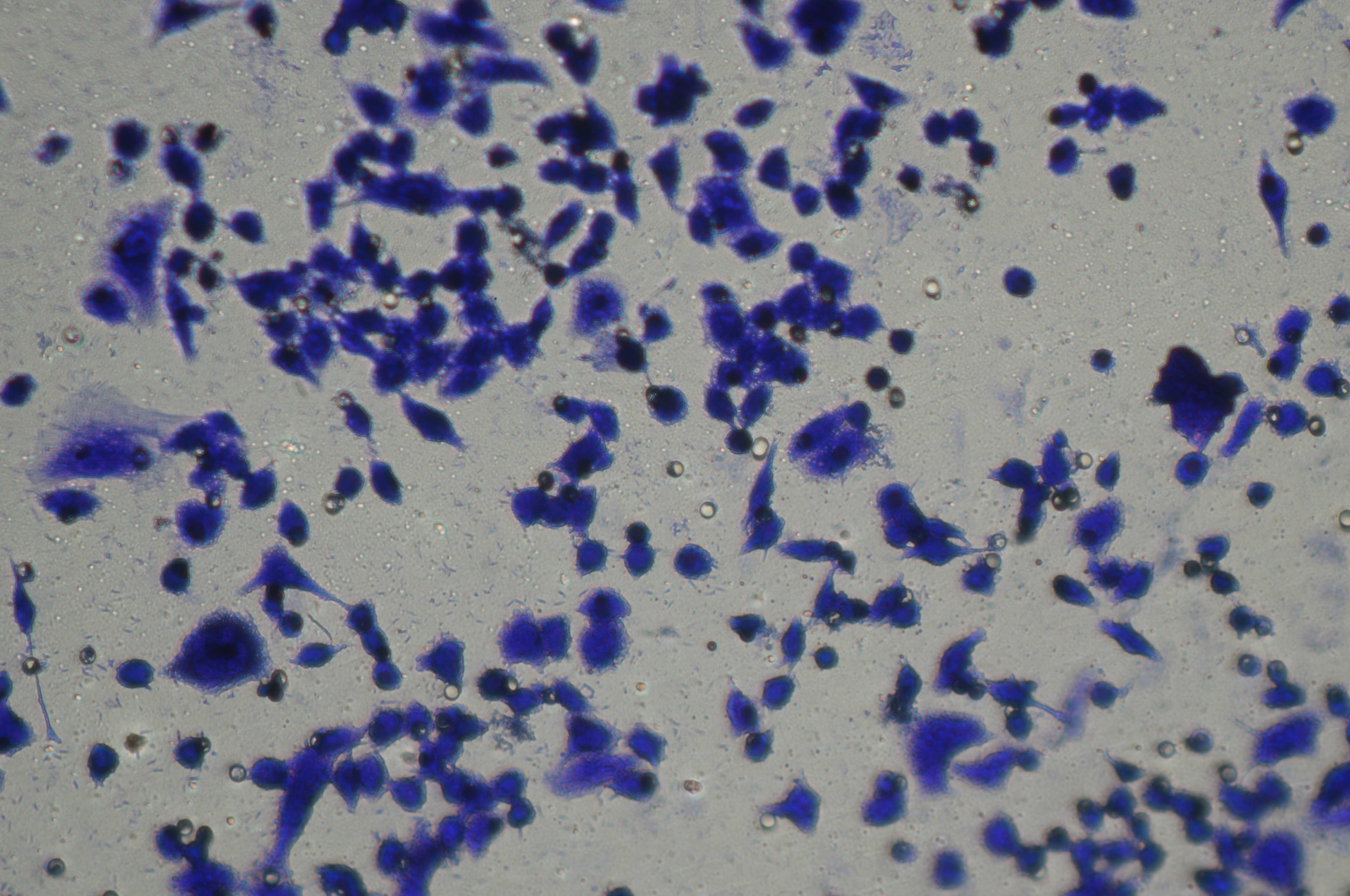

Supplement: Supplemental Information 12 [file peerj-12-18497-s012.zip › qbc939/qbc939 migration nc oe +Ca2+/picture/qbc939 nc 孔306.jpg]

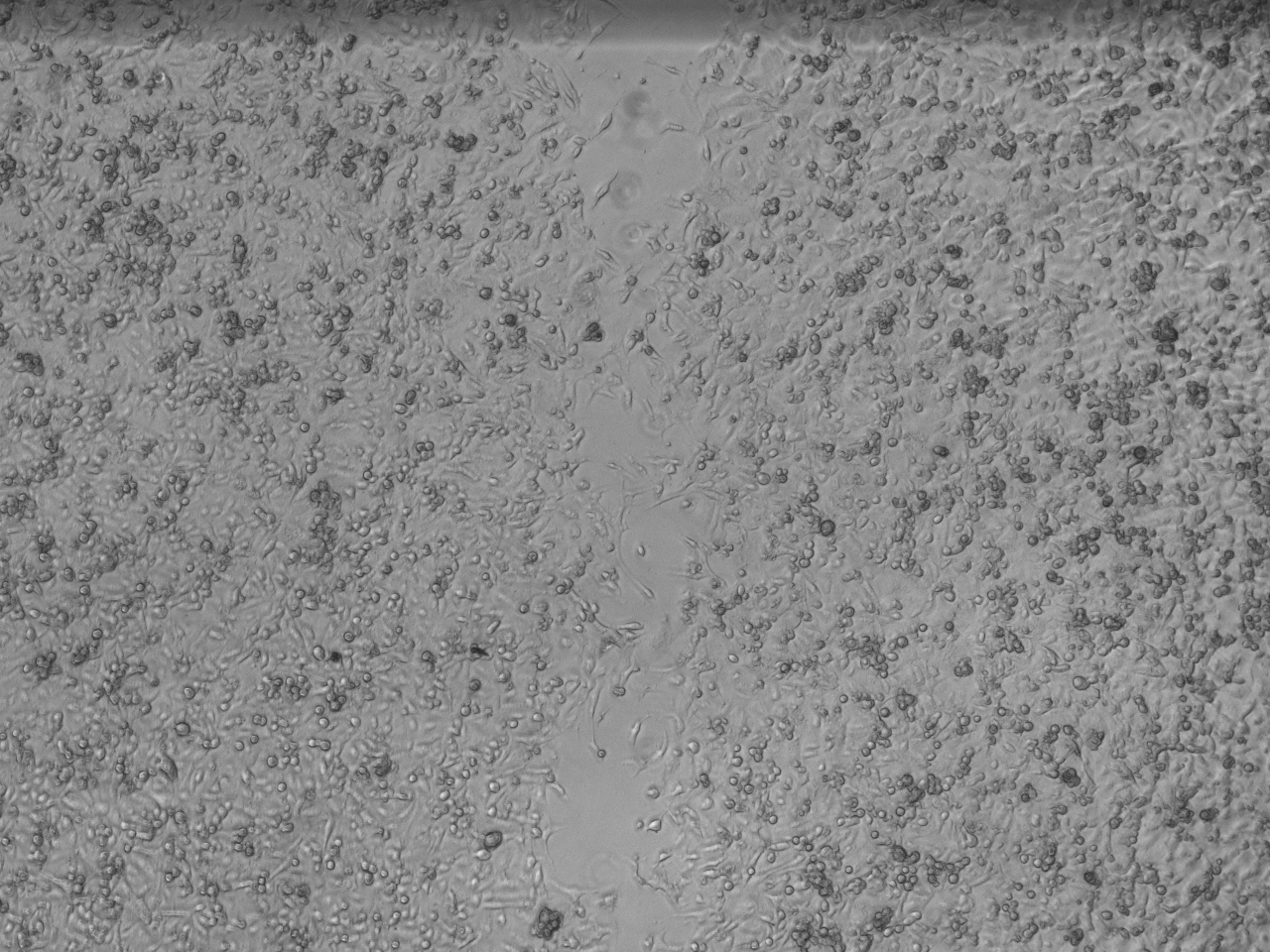

Supplement: Supplemental Information 12 [file peerj-12-18497-s012.zip › qbc939/QBC939 Wound Healing nc oe oe+Ca2+/nc/k1 NC1 24h.tif]

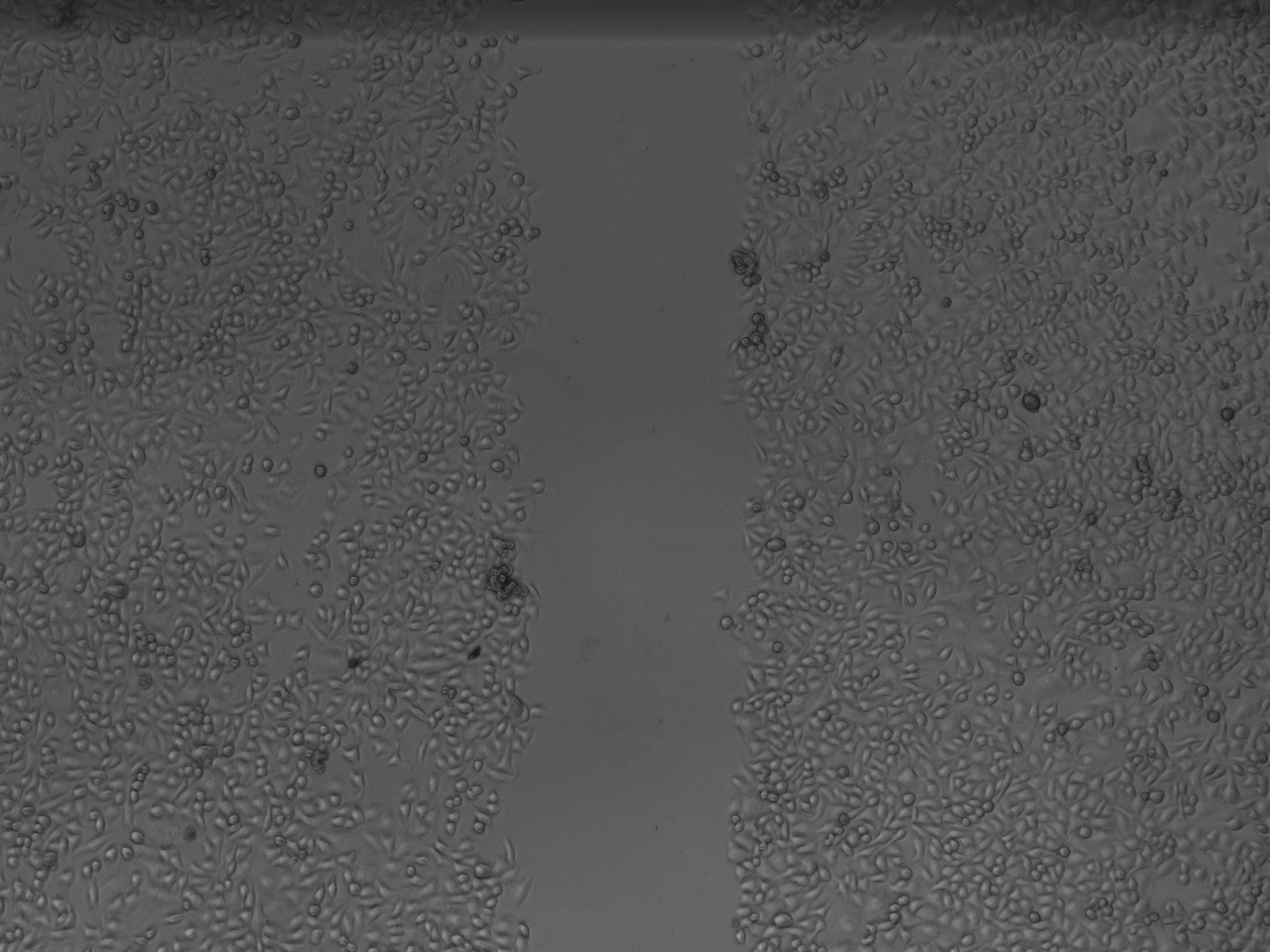

Supplement: Supplemental Information 12 [file peerj-12-18497-s012.zip › qbc939/QBC939 Wound Healing nc oe oe+Ca2+/nc/k1 NC1 oh.tif]

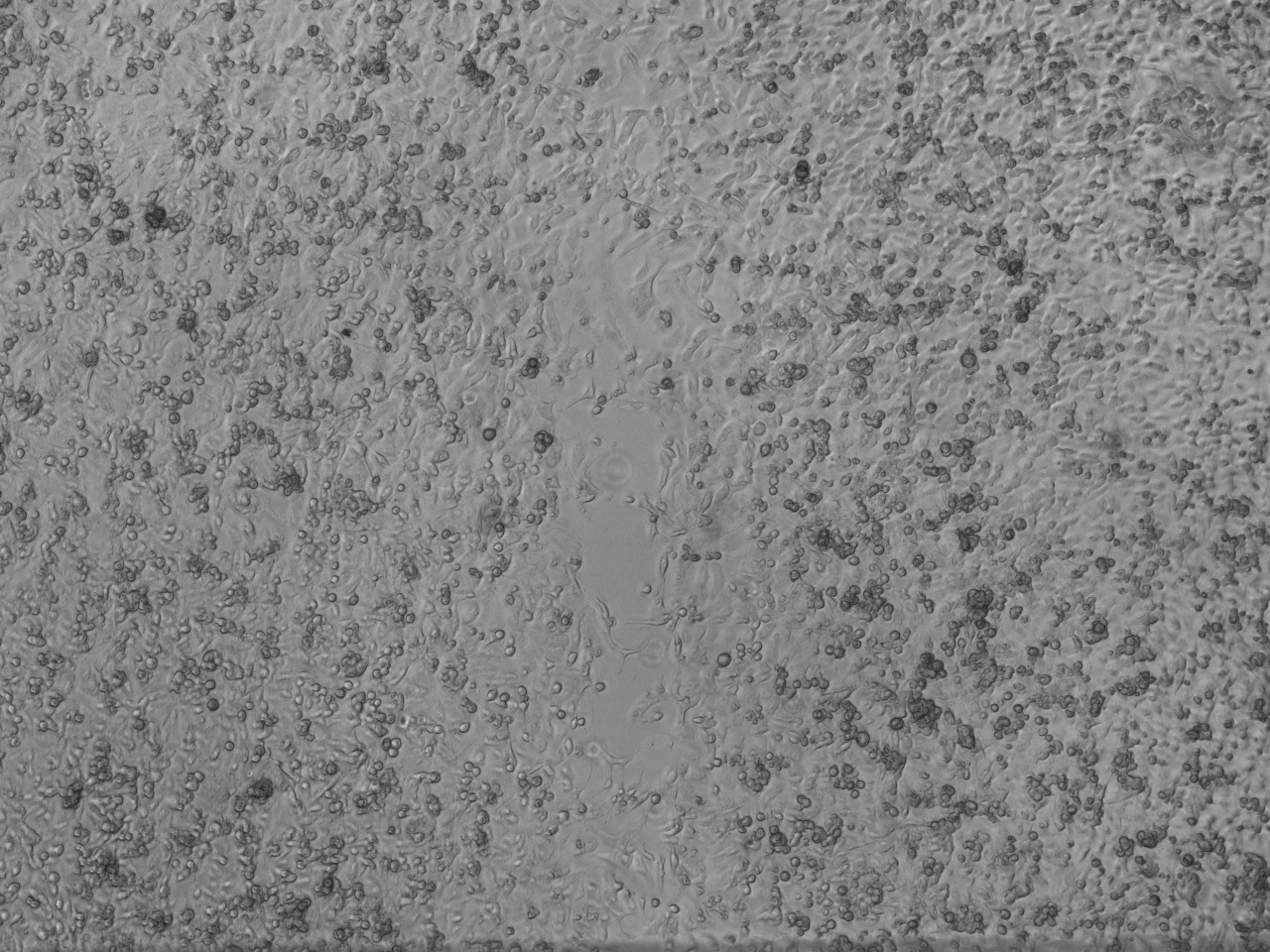

Supplement: Supplemental Information 12 [file peerj-12-18497-s012.zip › qbc939/QBC939 Wound Healing nc oe oe+Ca2+/nc/k1 NC2 24h.tif]

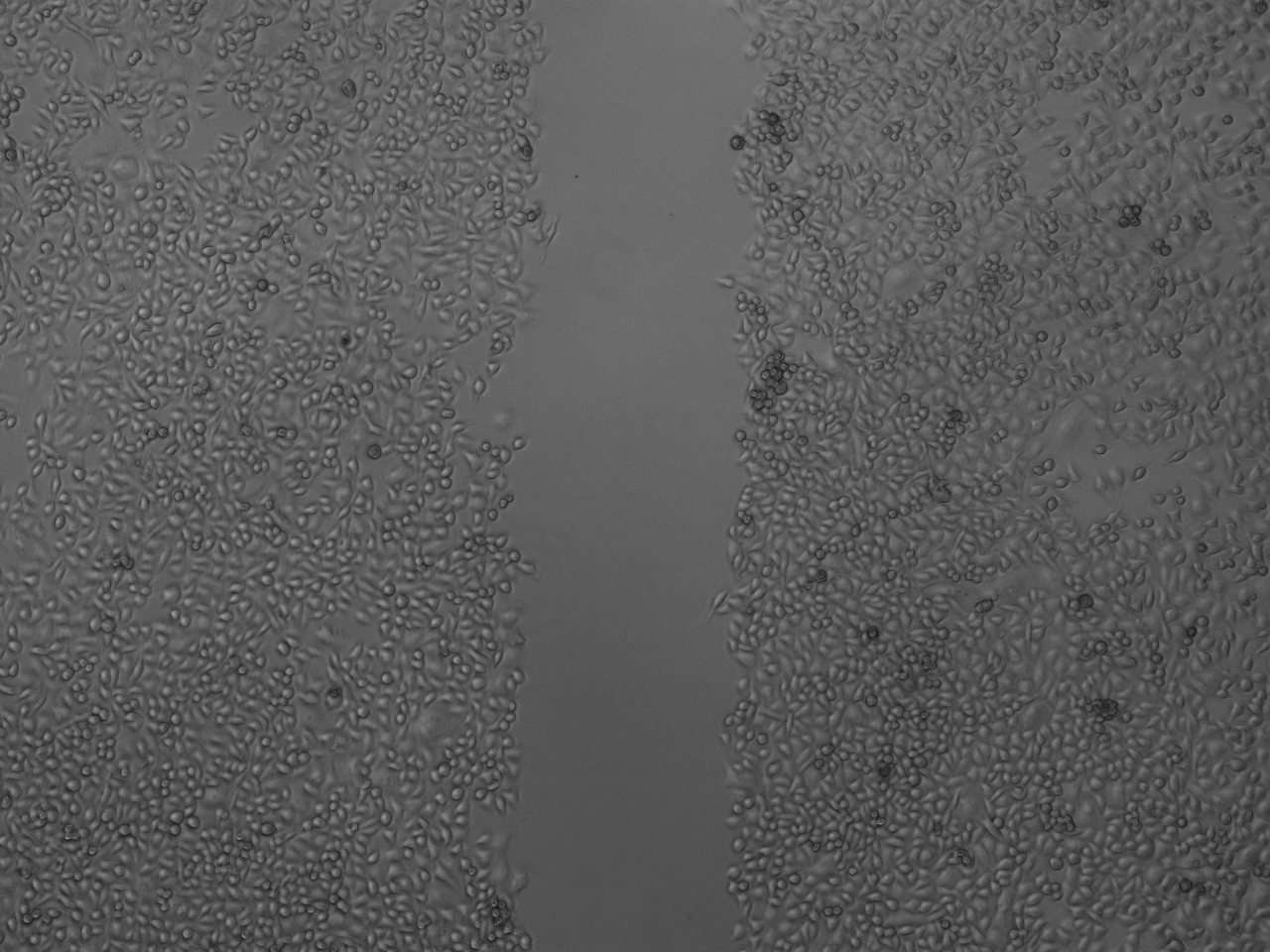

Supplement: Supplemental Information 12 [file peerj-12-18497-s012.zip › qbc939/QBC939 Wound Healing nc oe oe+Ca2+/nc/k1 NC2 oh.tif]

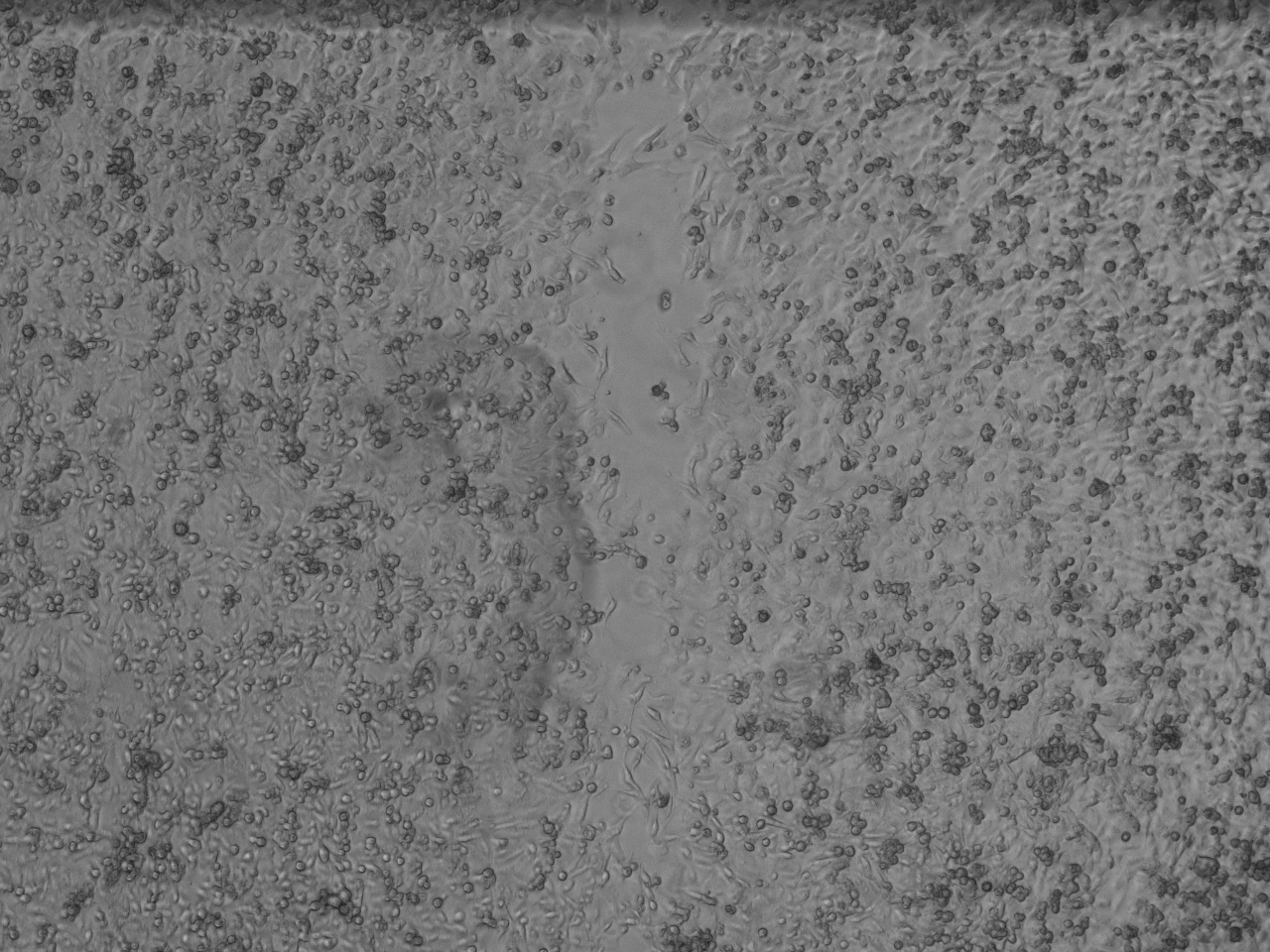

Supplement: Supplemental Information 12 [file peerj-12-18497-s012.zip › qbc939/QBC939 Wound Healing nc oe oe+Ca2+/nc/k1 NC3 24h.tif]

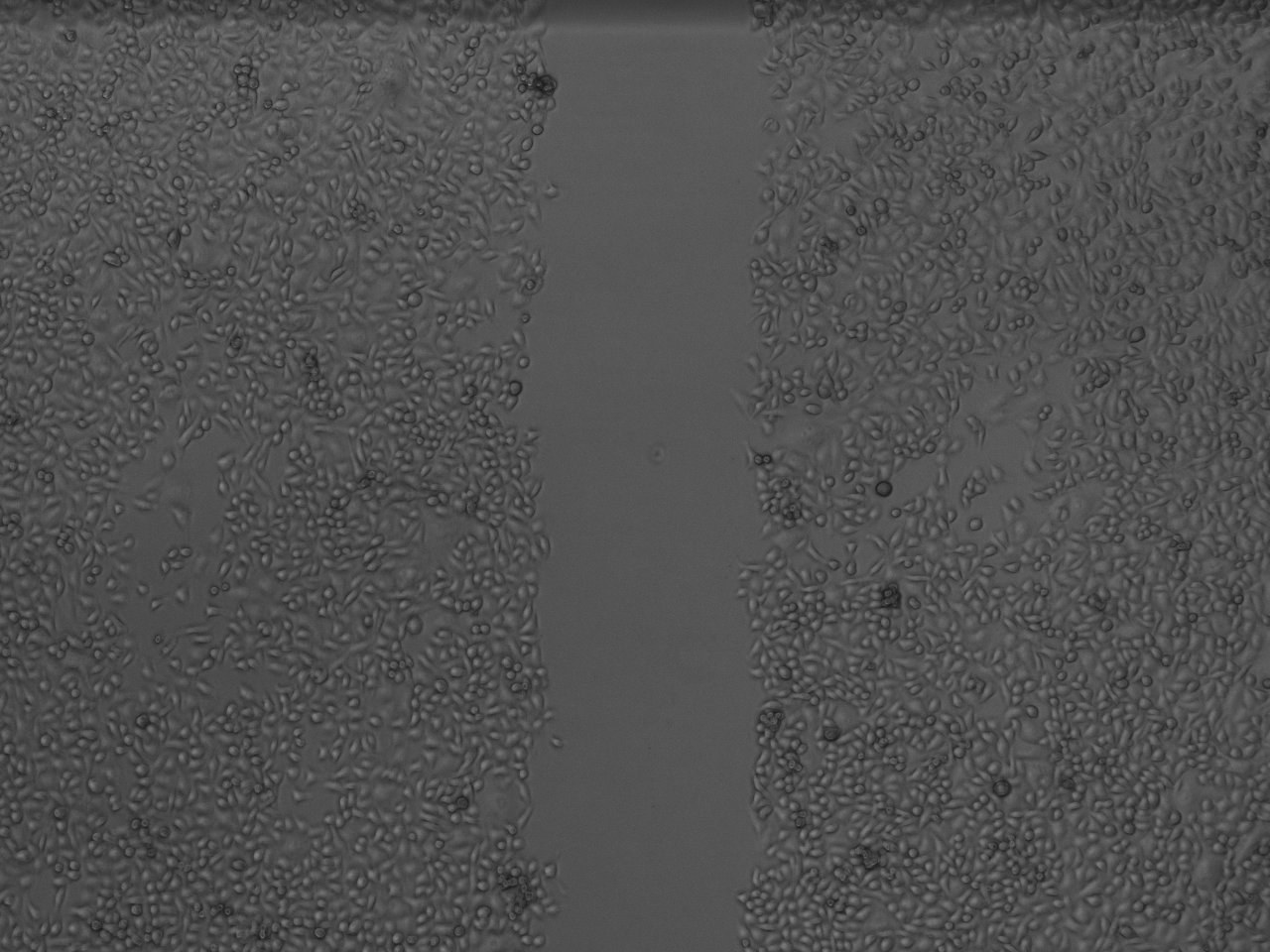

Supplement: Supplemental Information 12 [file peerj-12-18497-s012.zip › qbc939/QBC939 Wound Healing nc oe oe+Ca2+/nc/k1 NC3 oh.tif]

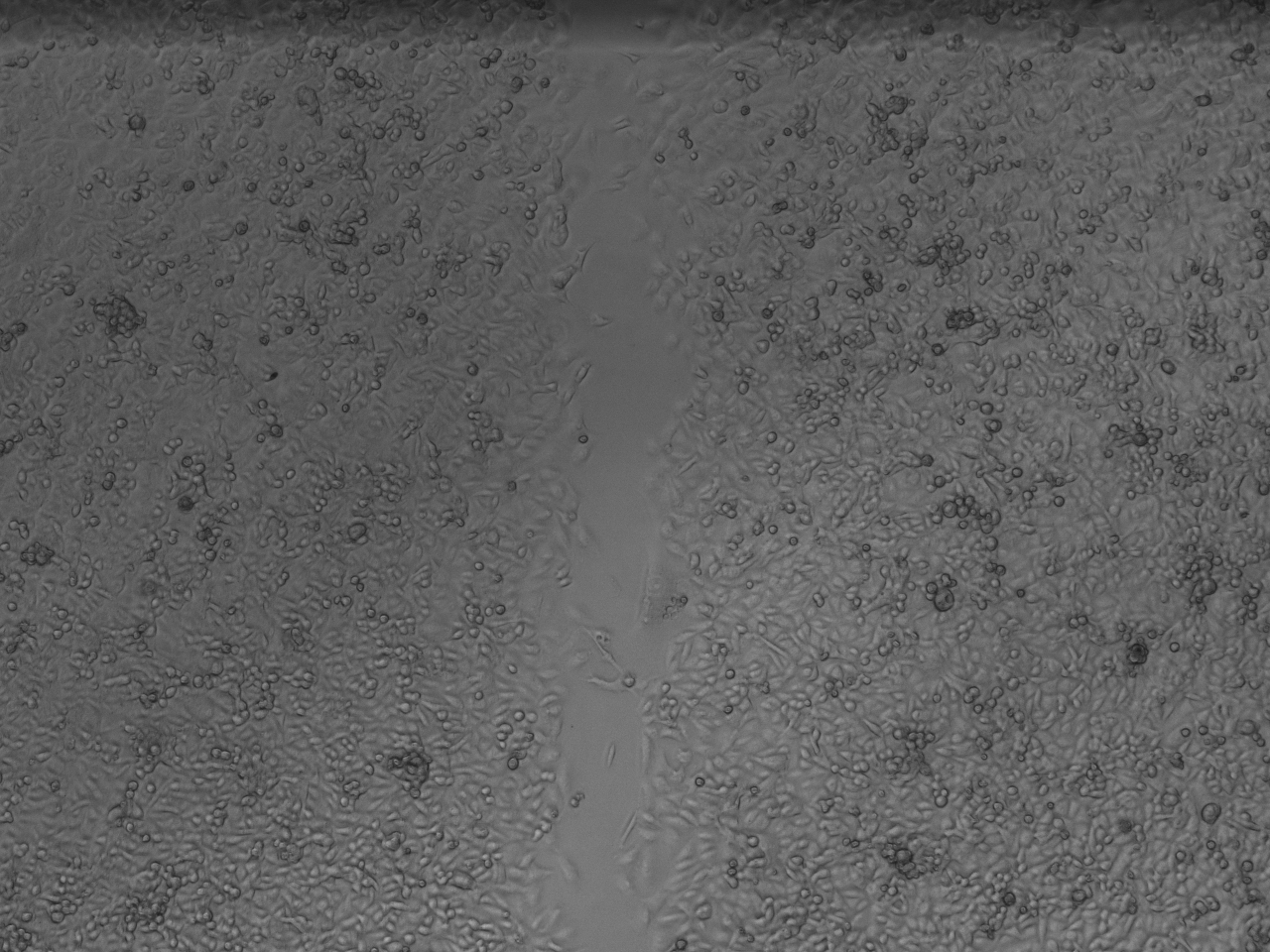

Supplement: Supplemental Information 12 [file peerj-12-18497-s012.zip › qbc939/QBC939 Wound Healing nc oe oe+Ca2+/oe/k3 OE1 24h.tif]

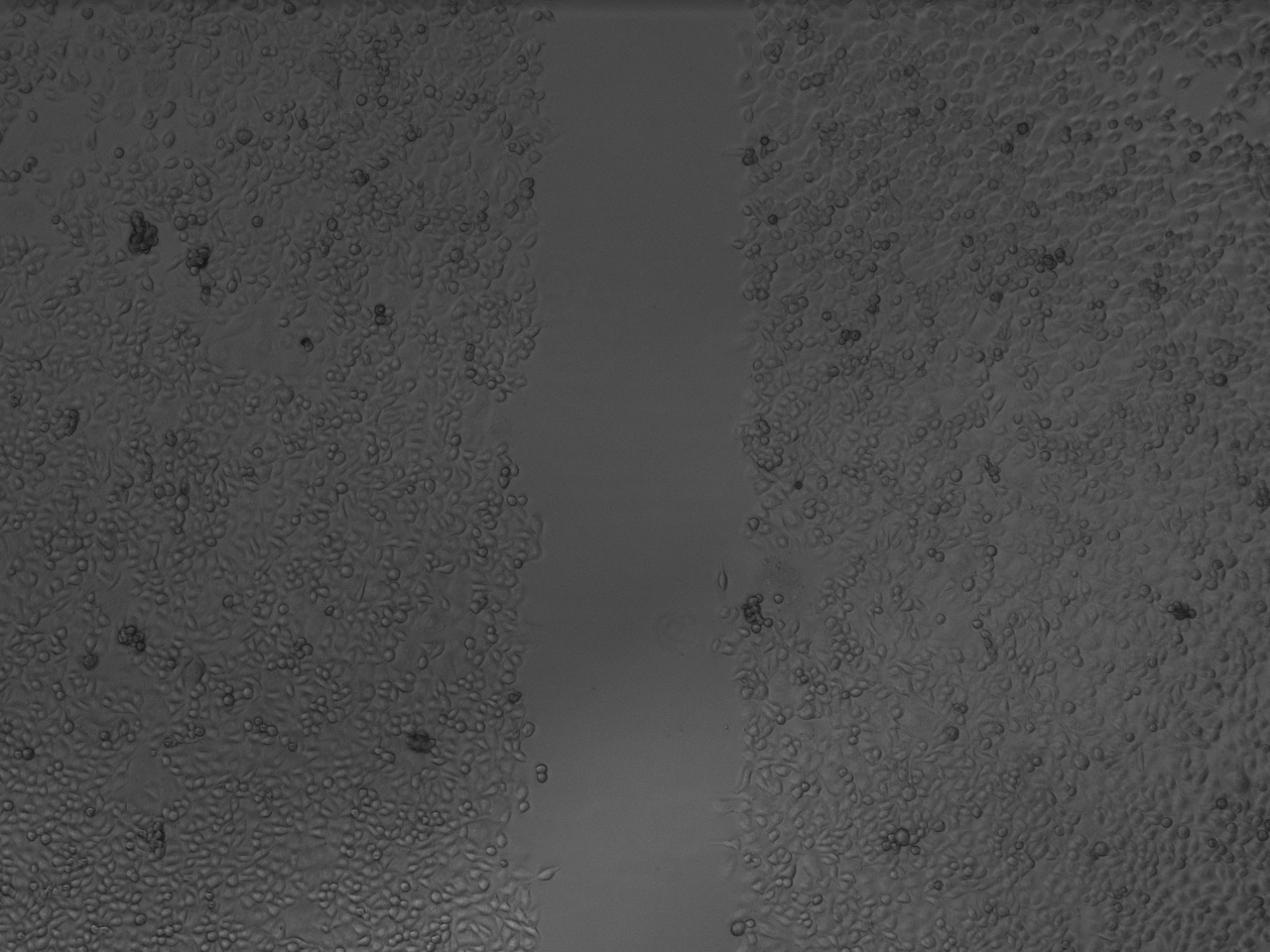

Supplement: Supplemental Information 12 [file peerj-12-18497-s012.zip › qbc939/QBC939 Wound Healing nc oe oe+Ca2+/oe/k3 OE1 oh.tif]

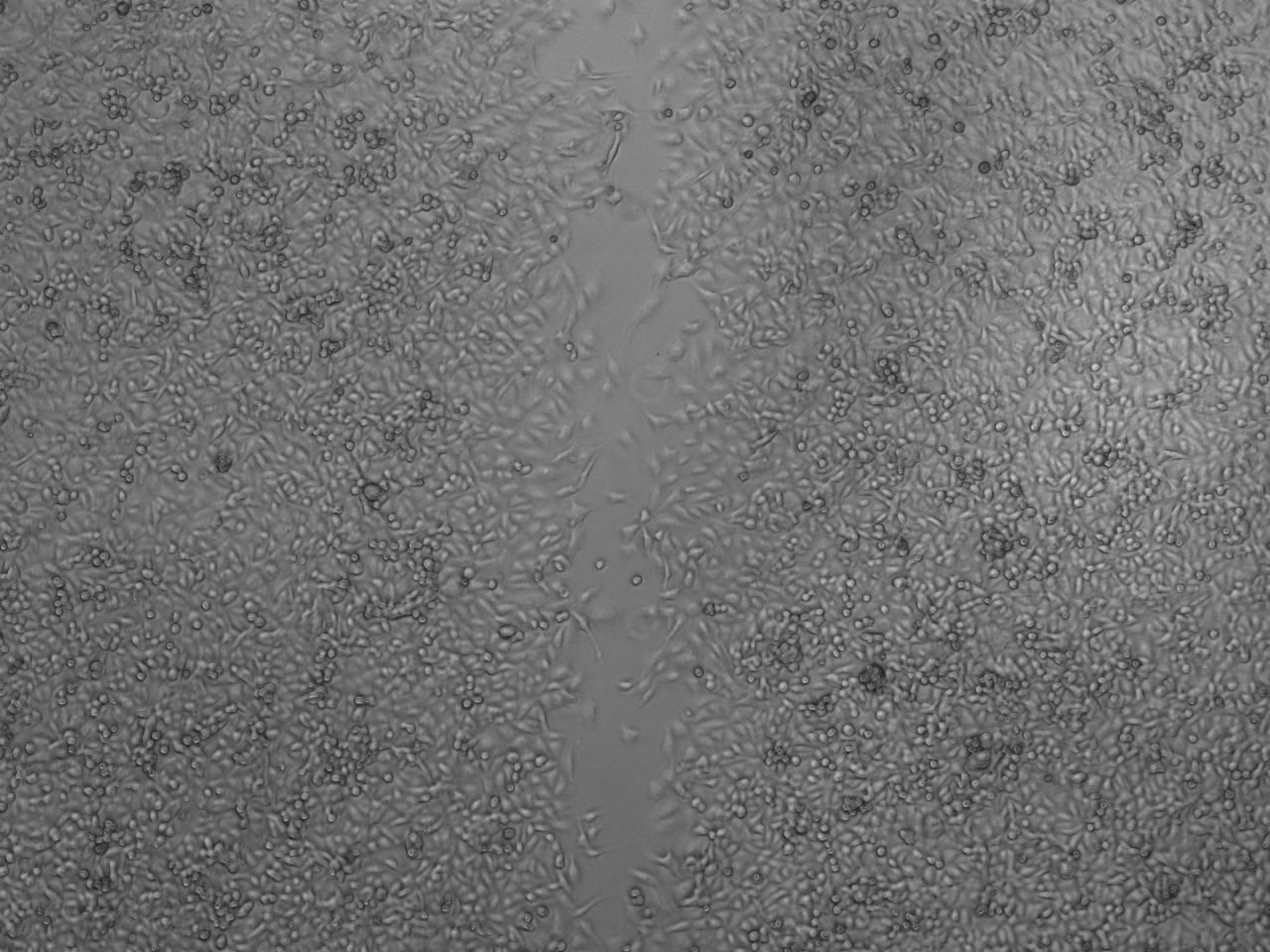

Supplement: Supplemental Information 12 [file peerj-12-18497-s012.zip › qbc939/QBC939 Wound Healing nc oe oe+Ca2+/oe/k3 OE2 24h.tif]

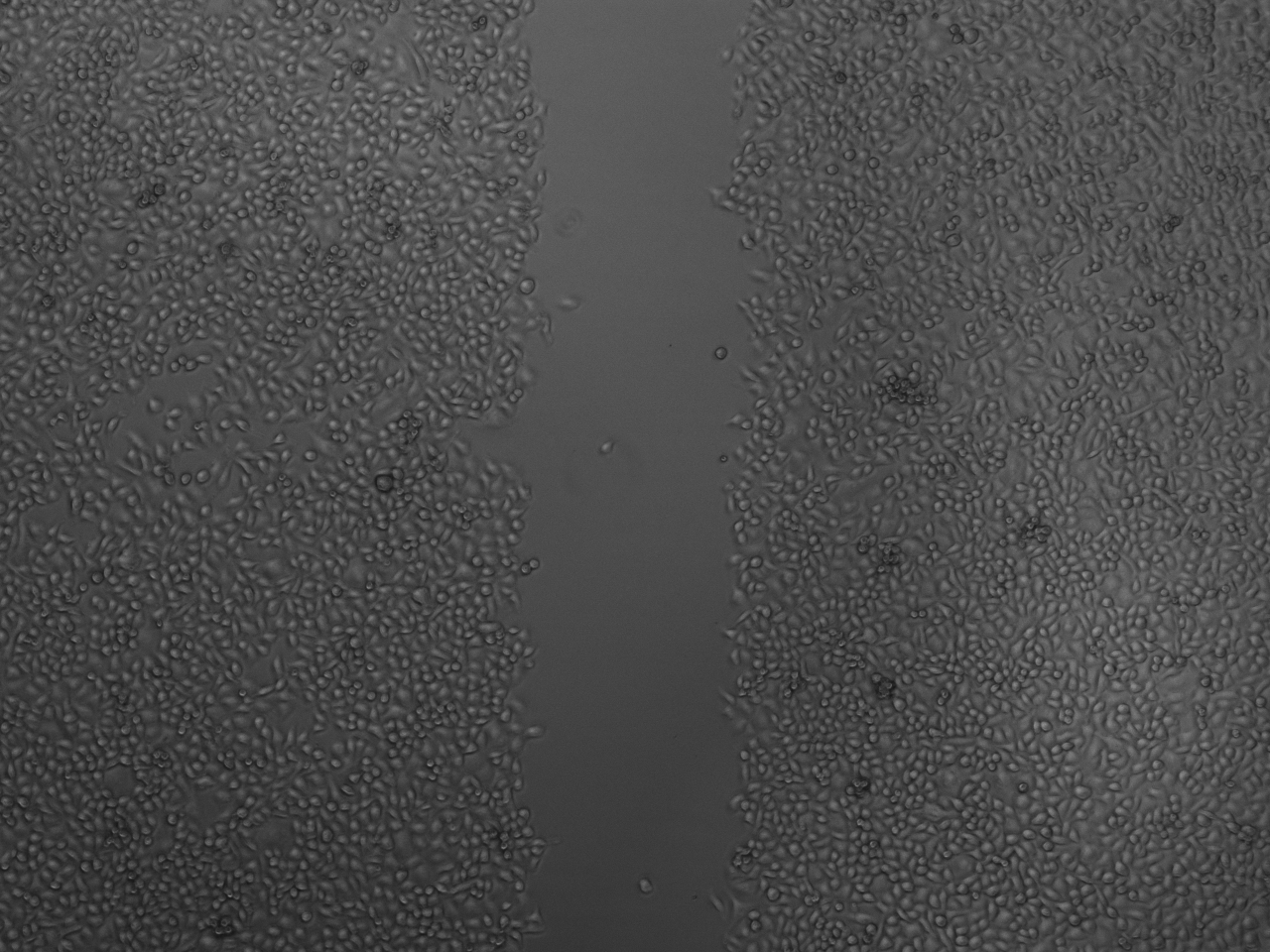

Supplement: Supplemental Information 12 [file peerj-12-18497-s012.zip › qbc939/QBC939 Wound Healing nc oe oe+Ca2+/oe/k3 OE2 oh.tif]

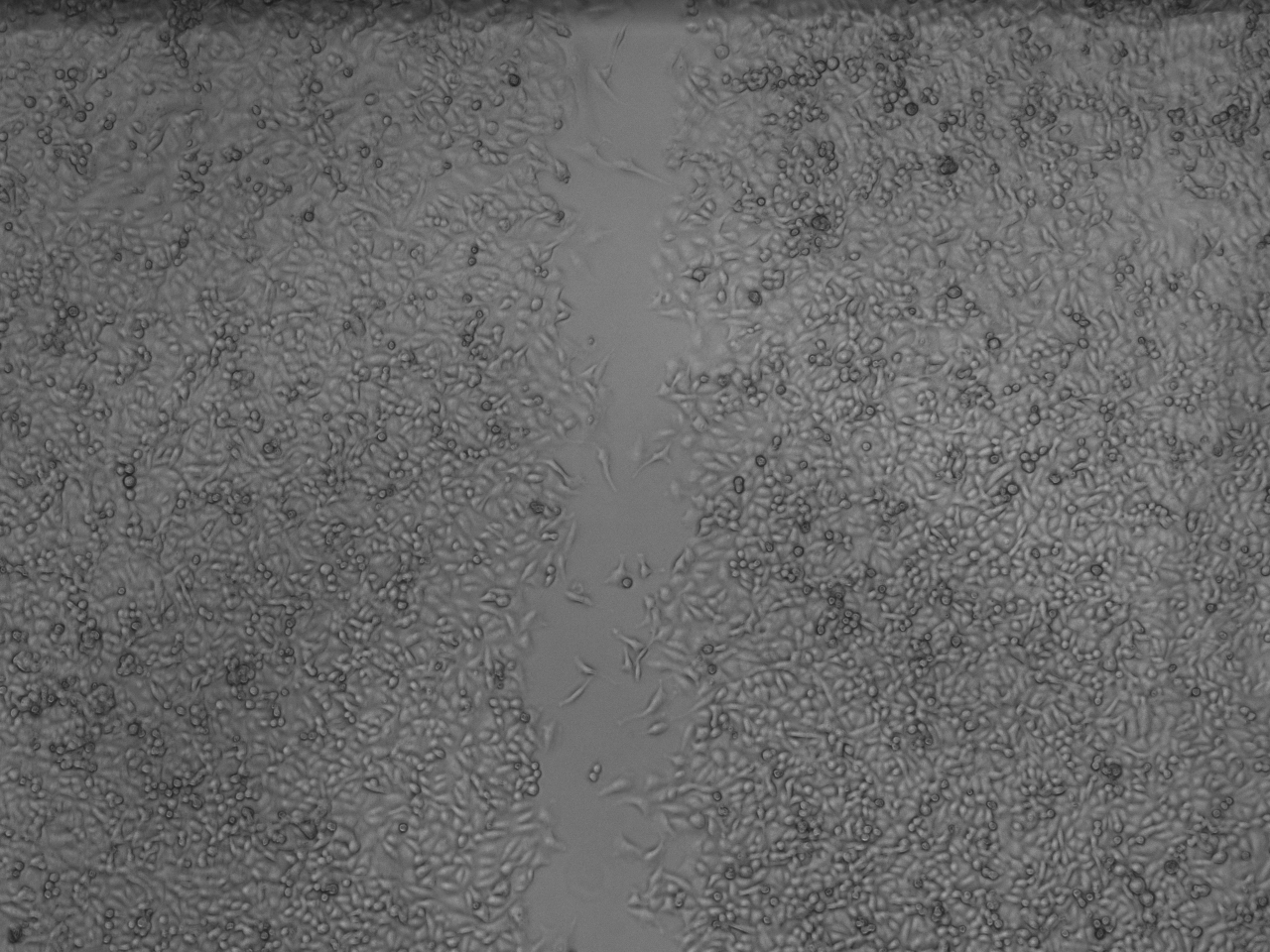

Supplement: Supplemental Information 12 [file peerj-12-18497-s012.zip › qbc939/QBC939 Wound Healing nc oe oe+Ca2+/oe/k3 OE3 24h.tif]

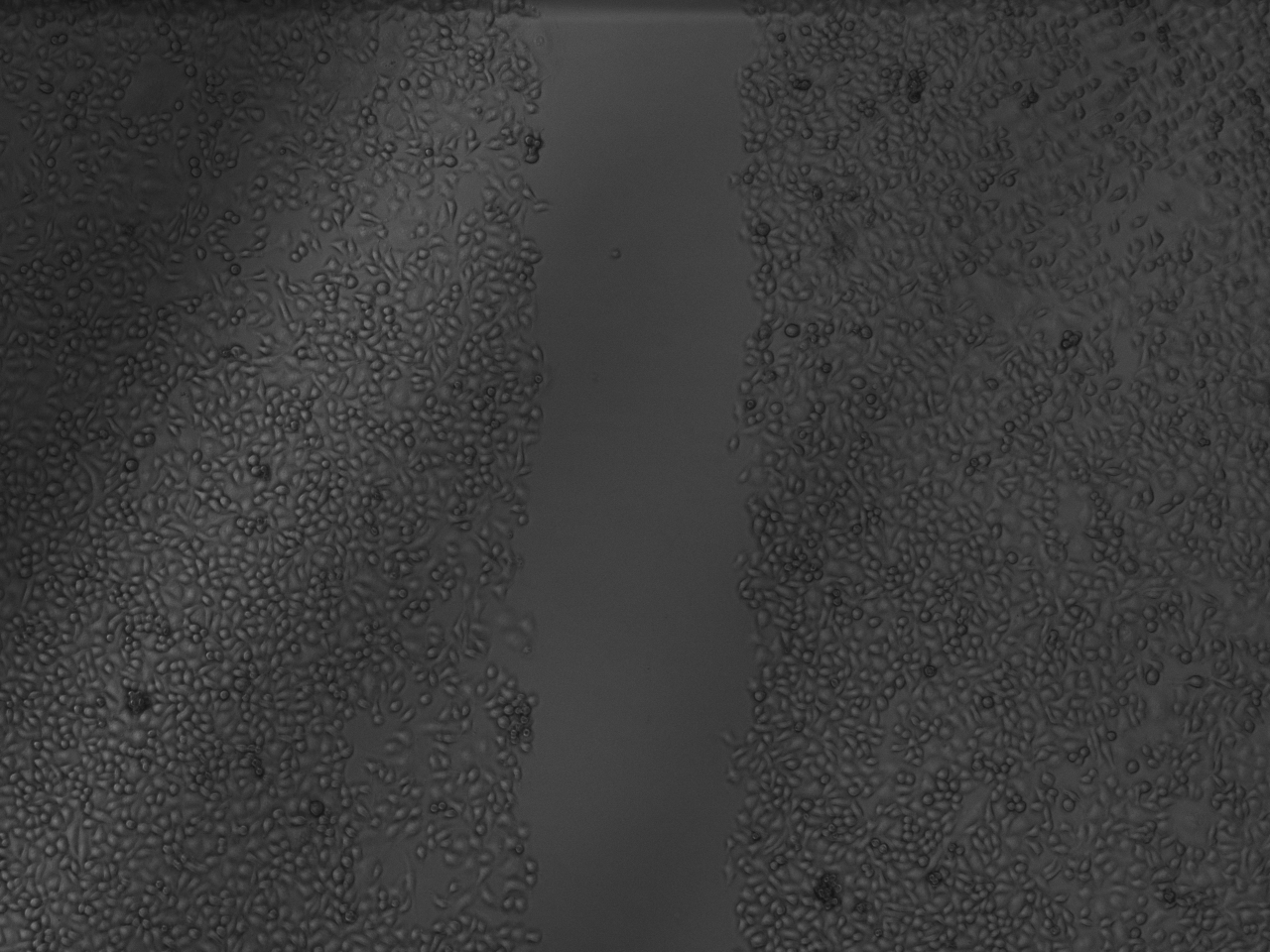

Supplement: Supplemental Information 12 [file peerj-12-18497-s012.zip › qbc939/QBC939 Wound Healing nc oe oe+Ca2+/oe/k3 OE3 oh.tif]

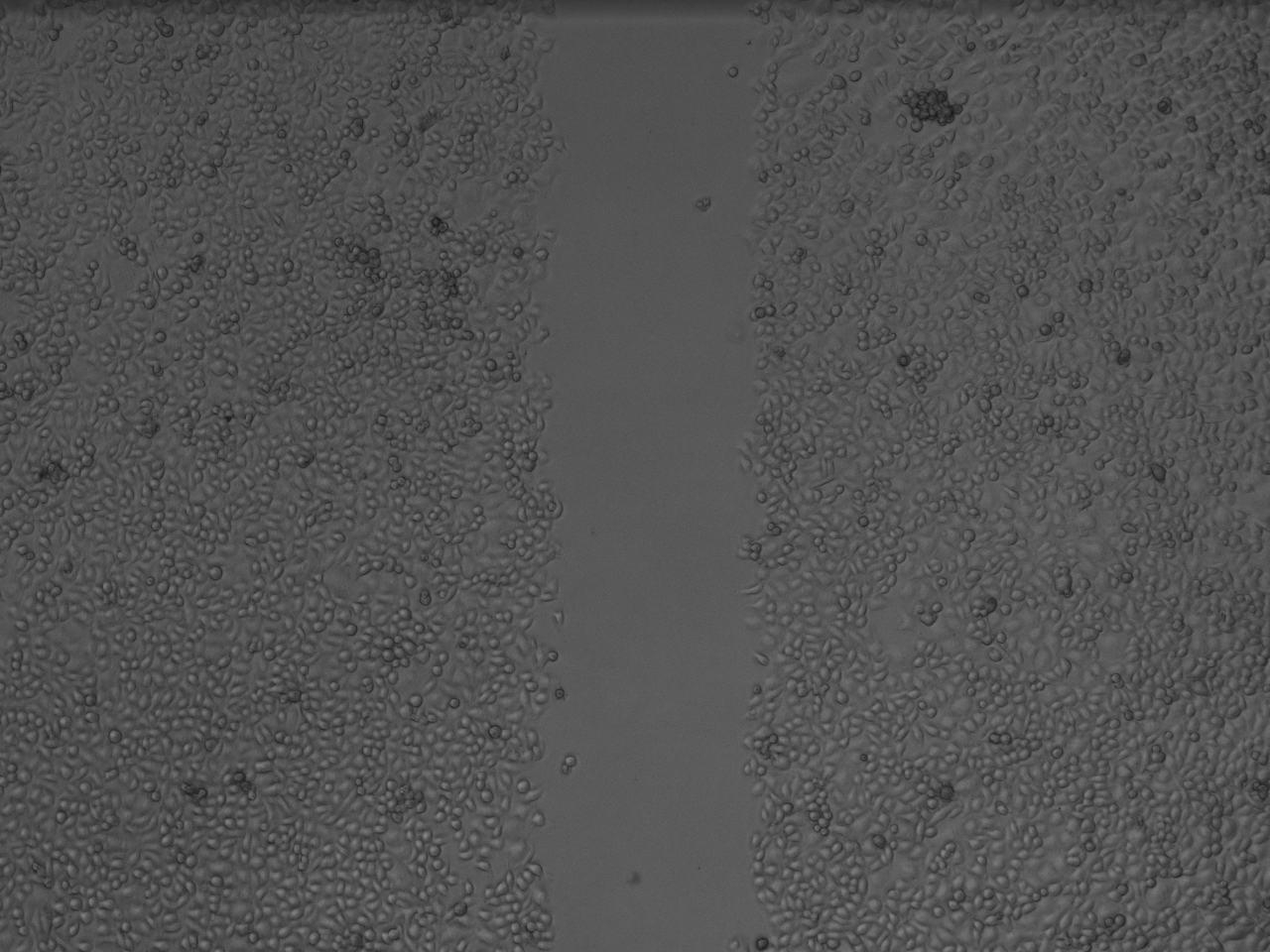

Supplement: Supplemental Information 12 [file peerj-12-18497-s012.zip › qbc939/QBC939 Wound Healing nc oe oe+Ca2+/oe +Ca2+/k5 OE+Ca2+ 1 oh.tif]

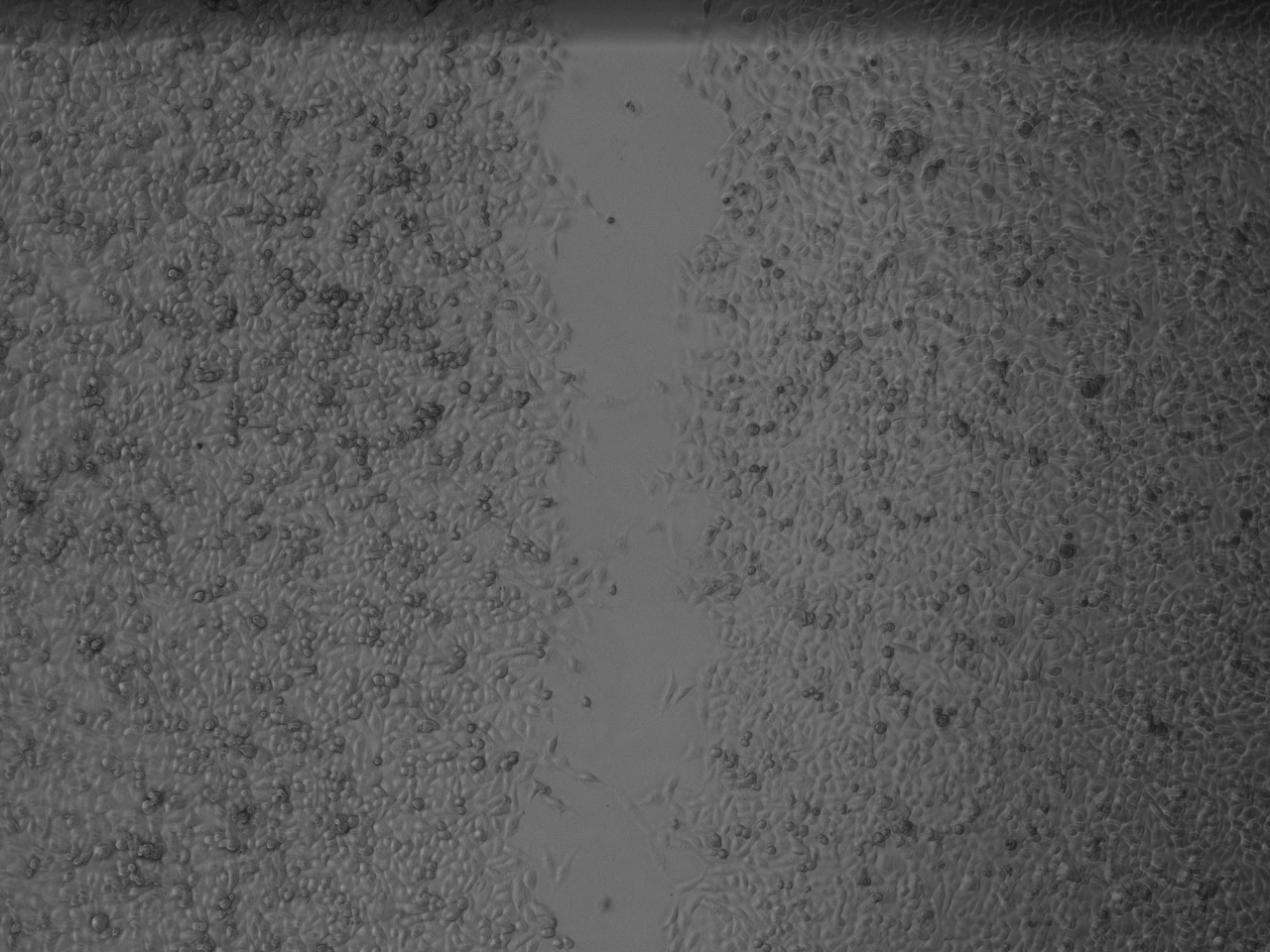

Supplement: Supplemental Information 12 [file peerj-12-18497-s012.zip › qbc939/QBC939 Wound Healing nc oe oe+Ca2+/oe +Ca2+/k5 OE+Ca2+ 1 24h.tif]

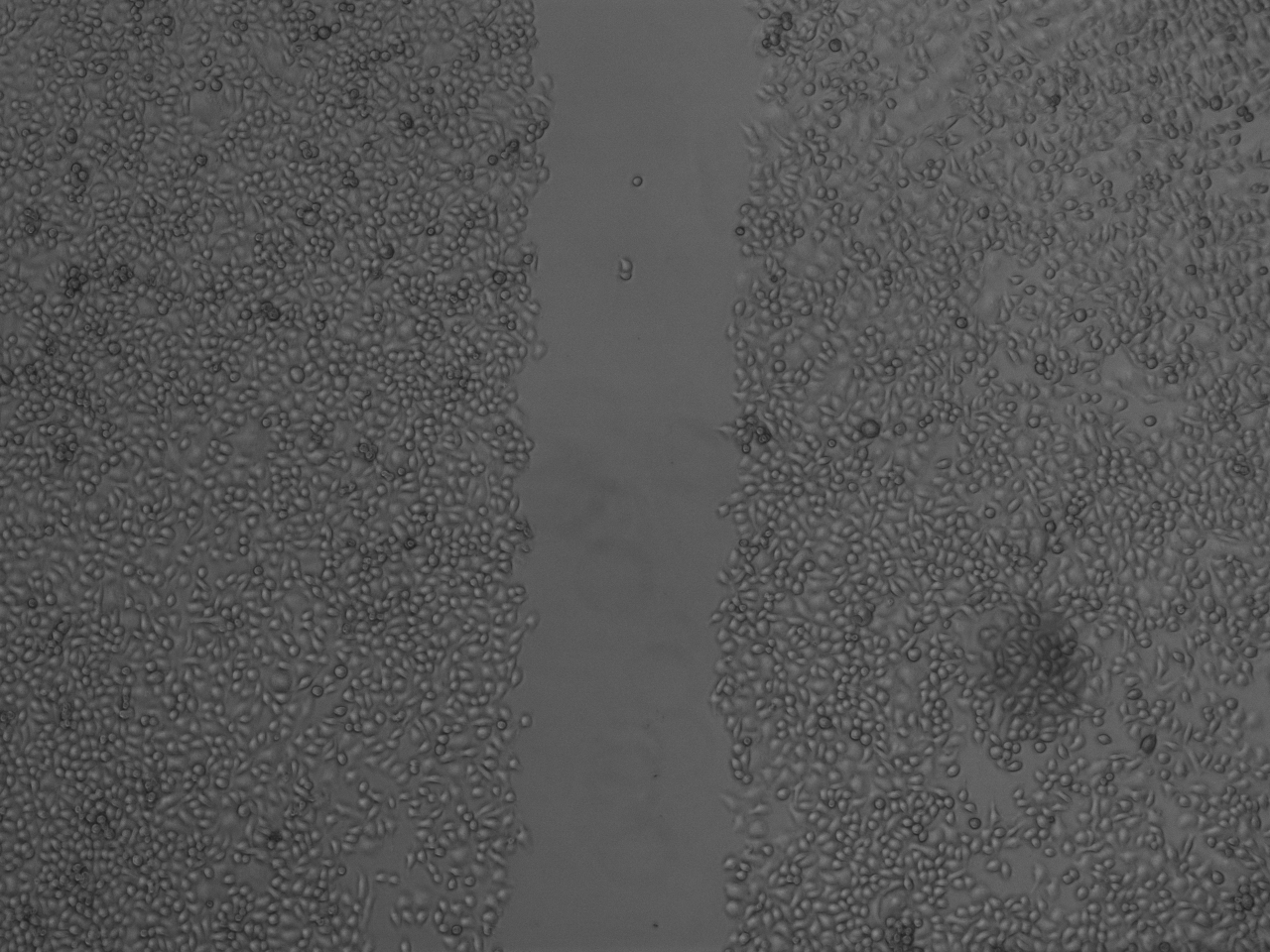

Supplement: Supplemental Information 12 [file peerj-12-18497-s012.zip › qbc939/QBC939 Wound Healing nc oe oe+Ca2+/oe +Ca2+/k5 OE+Ca2+ 2 oh.tif]

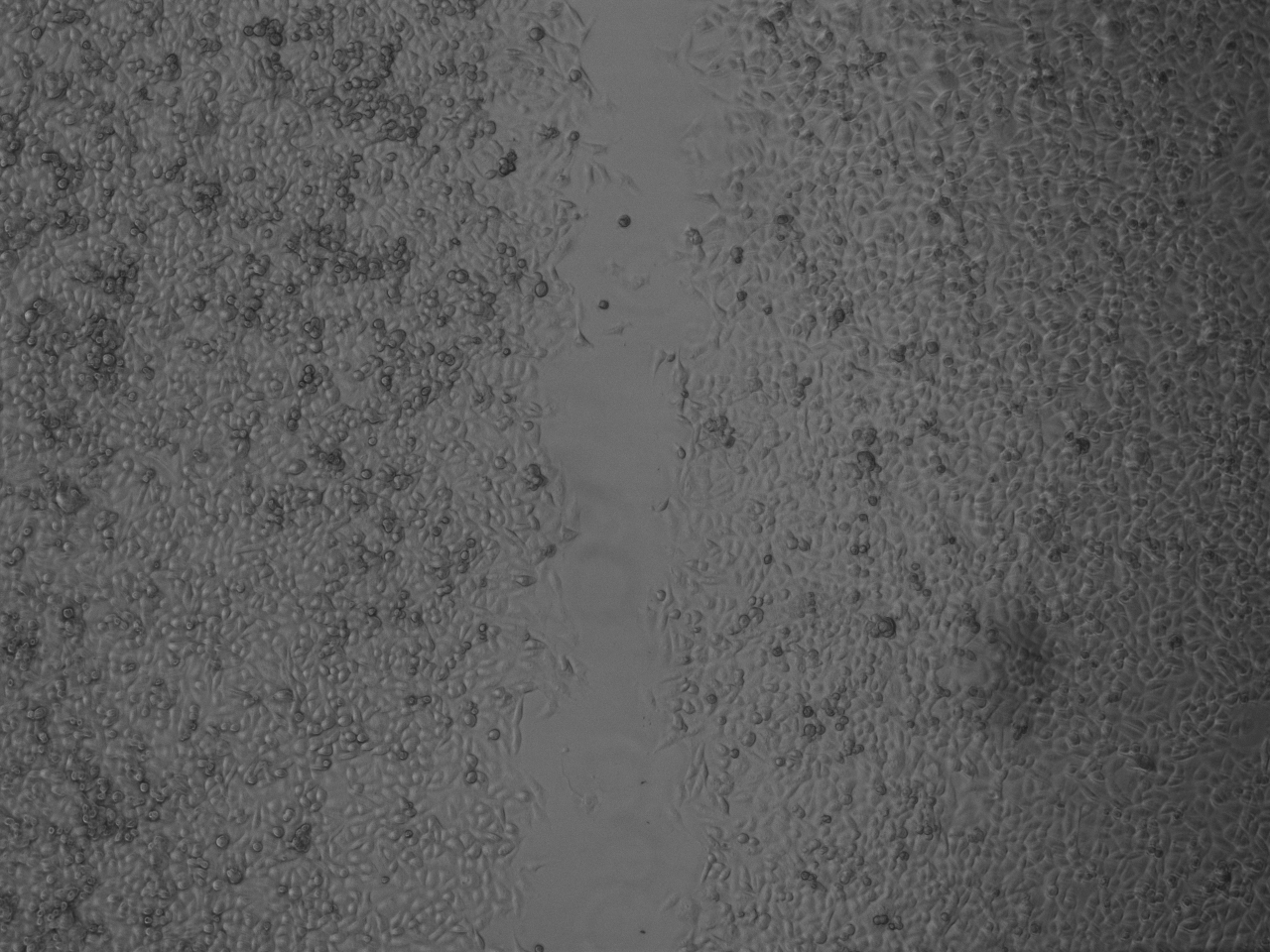

Supplement: Supplemental Information 12 [file peerj-12-18497-s012.zip › qbc939/QBC939 Wound Healing nc oe oe+Ca2+/oe +Ca2+/k5 OE+Ca2+ 2 24h.tif]

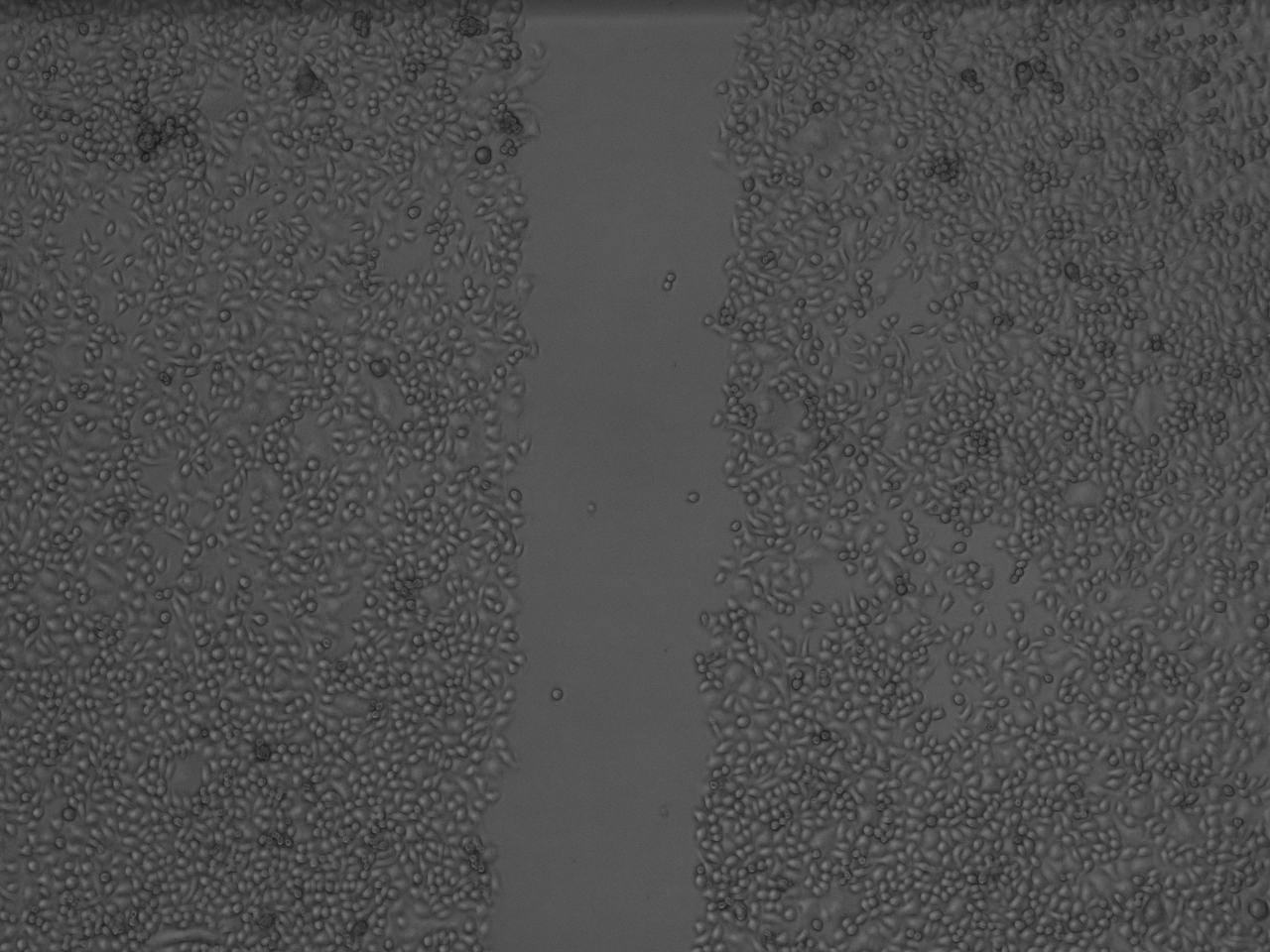

Supplement: Supplemental Information 12 [file peerj-12-18497-s012.zip › qbc939/QBC939 Wound Healing nc oe oe+Ca2+/oe +Ca2+/k5 OE+Ca2+ 3 oh.tif]

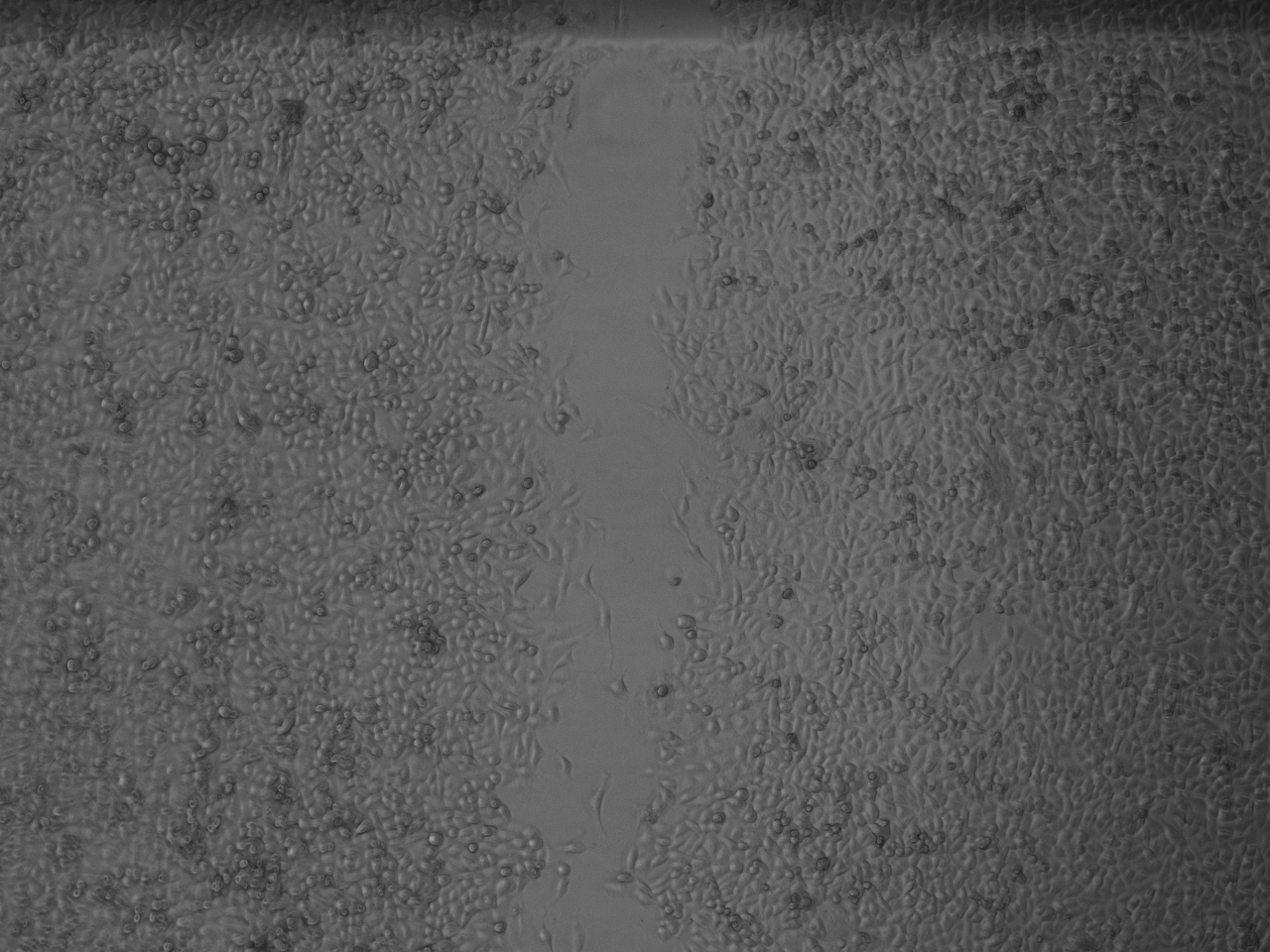

Supplement: Supplemental Information 12 [file peerj-12-18497-s012.zip › qbc939/QBC939 Wound Healing nc oe oe+Ca2+/oe +Ca2+/k5 OE+Ca2+ 3 24h.tif]

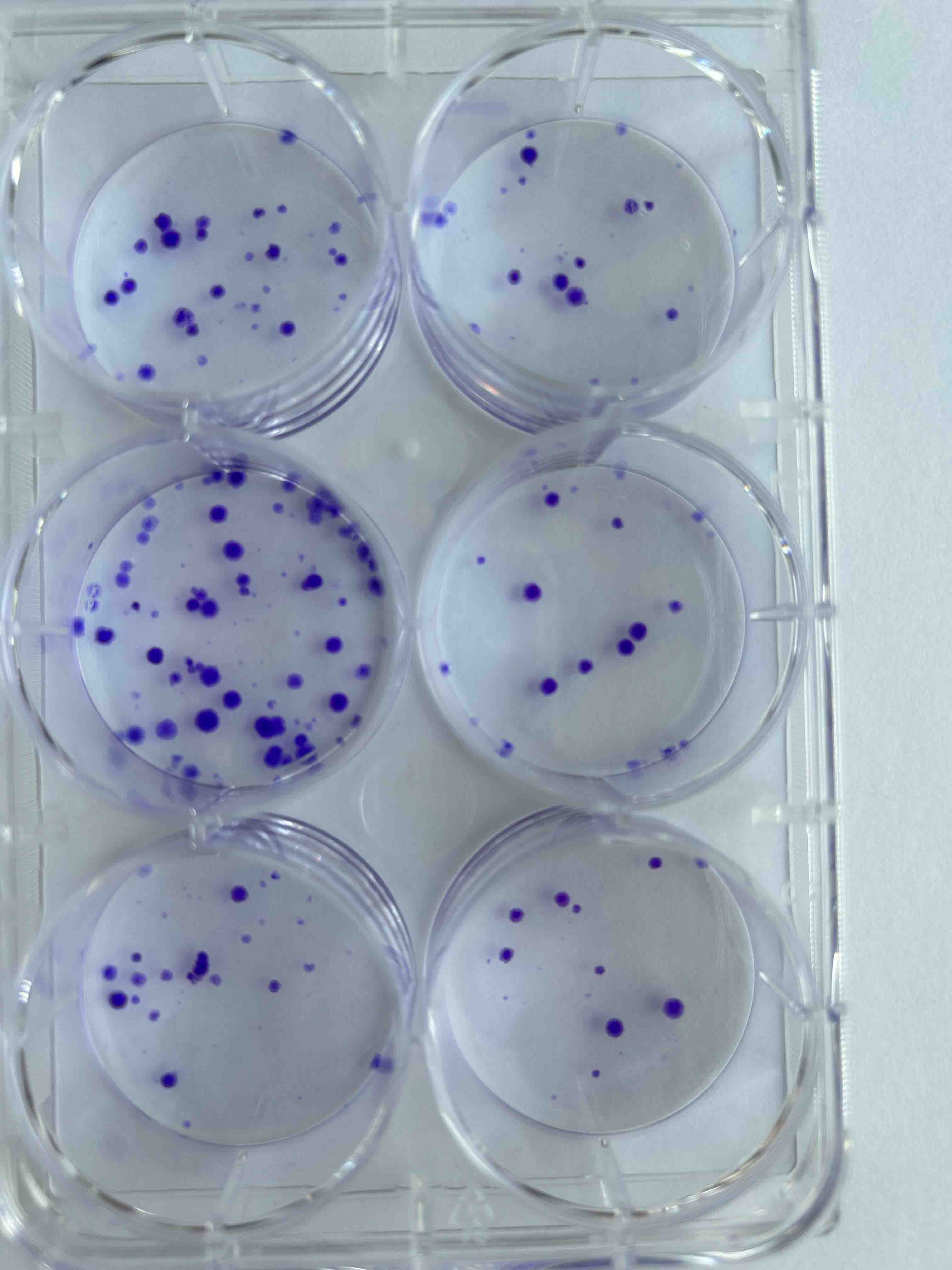

Supplement: Supplemental Information 13 [file peerj-12-18497-s013.zip › hucct1 functional experiment/control overexpression(NC OE)/hucct nc oe clone formation/picture/hucct clec3b过表达基因 nc oe.jpg]

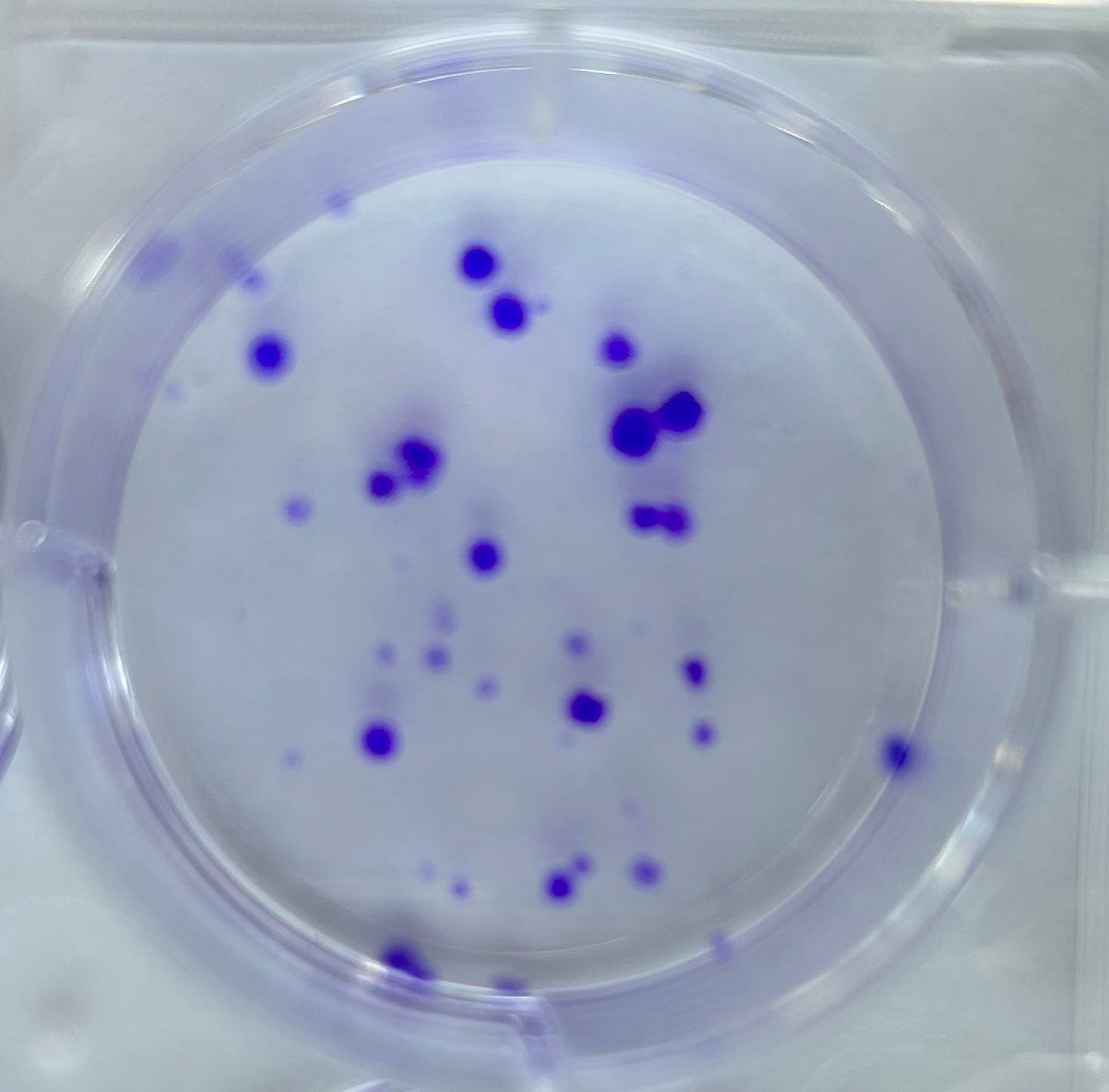

Supplement: Supplemental Information 13 [file peerj-12-18497-s013.zip › hucct1 functional experiment/control overexpression(NC OE)/hucct nc oe clone formation/picture/nc 孔1.jpg]

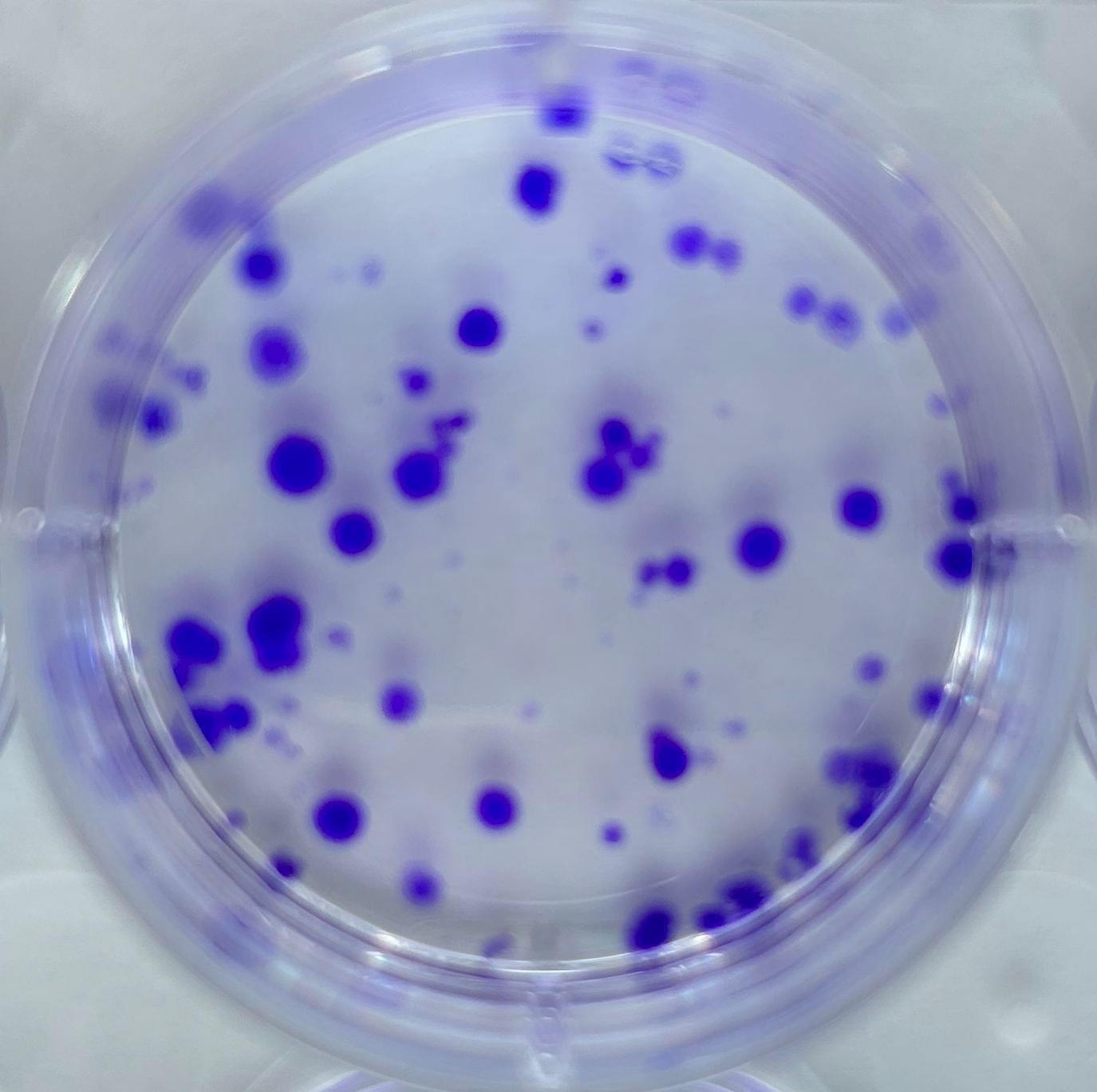

Supplement: Supplemental Information 13 [file peerj-12-18497-s013.zip › hucct1 functional experiment/control overexpression(NC OE)/hucct nc oe clone formation/picture/nc 孔2.jpg]

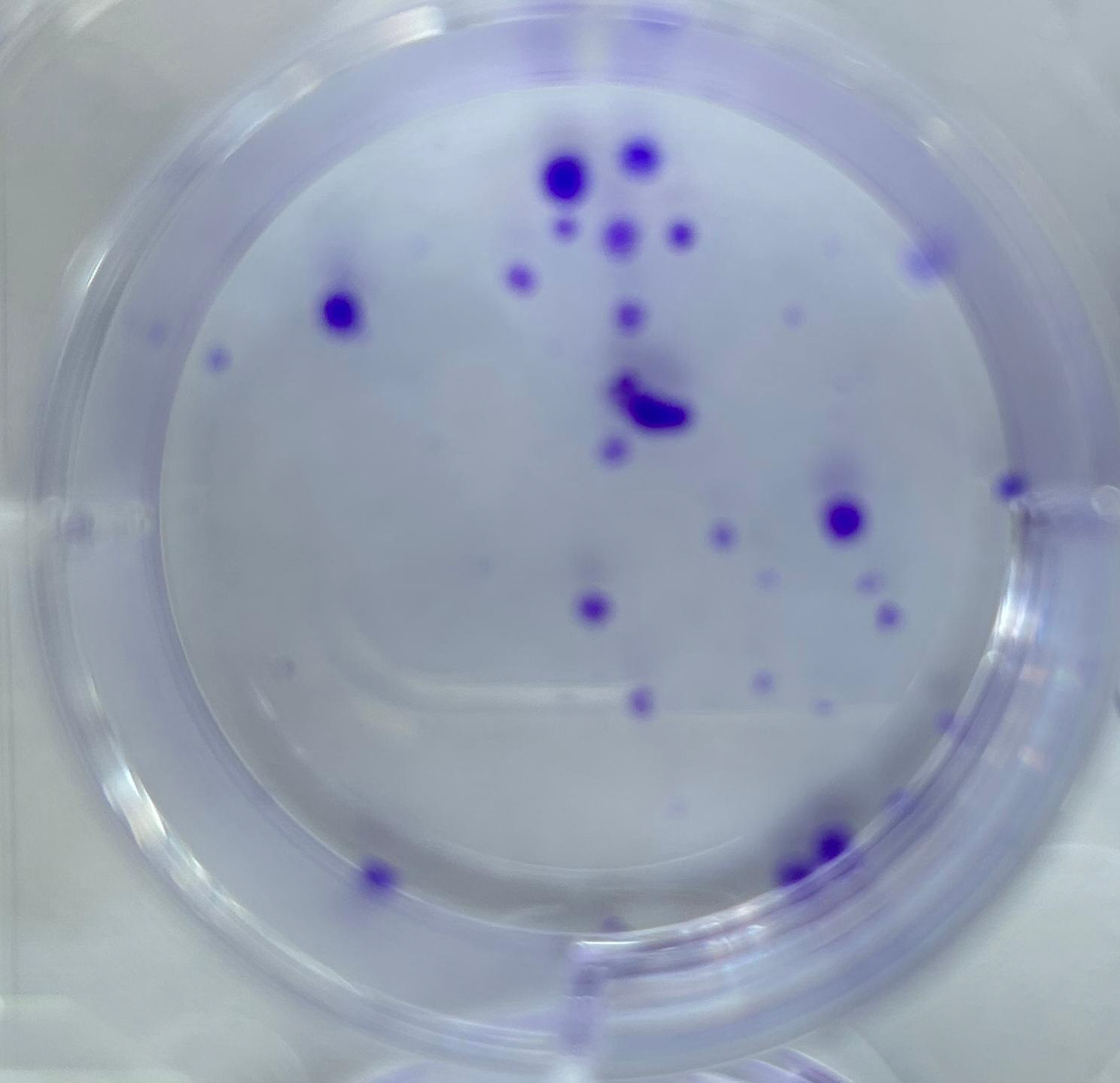

Supplement: Supplemental Information 13 [file peerj-12-18497-s013.zip › hucct1 functional experiment/control overexpression(NC OE)/hucct nc oe clone formation/picture/nc 孔3.jpg]

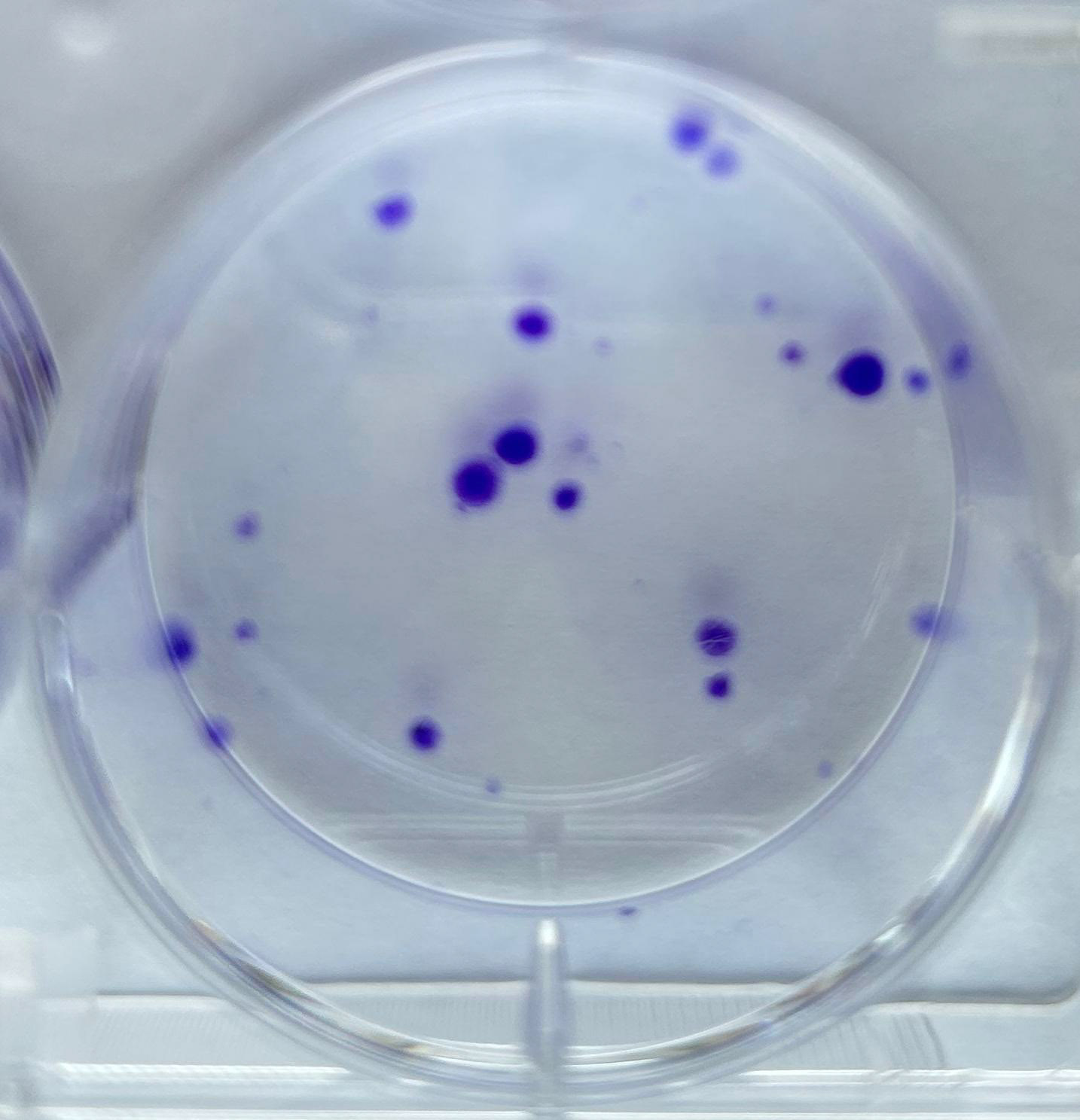

Supplement: Supplemental Information 13 [file peerj-12-18497-s013.zip › hucct1 functional experiment/control overexpression(NC OE)/hucct nc oe clone formation/picture/oe 孔1.jpg]

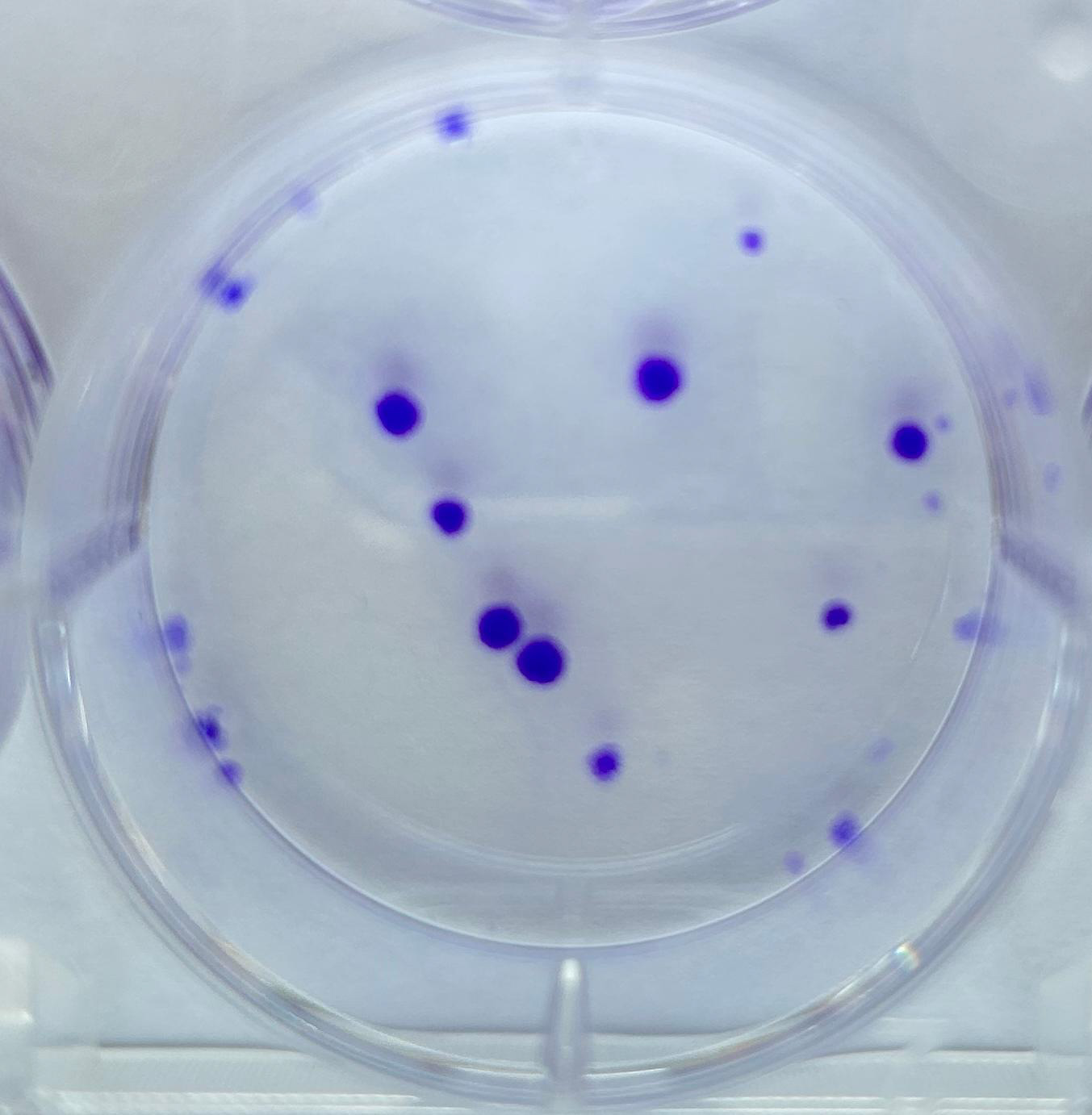

Supplement: Supplemental Information 13 [file peerj-12-18497-s013.zip › hucct1 functional experiment/control overexpression(NC OE)/hucct nc oe clone formation/picture/oe 孔2.jpg]

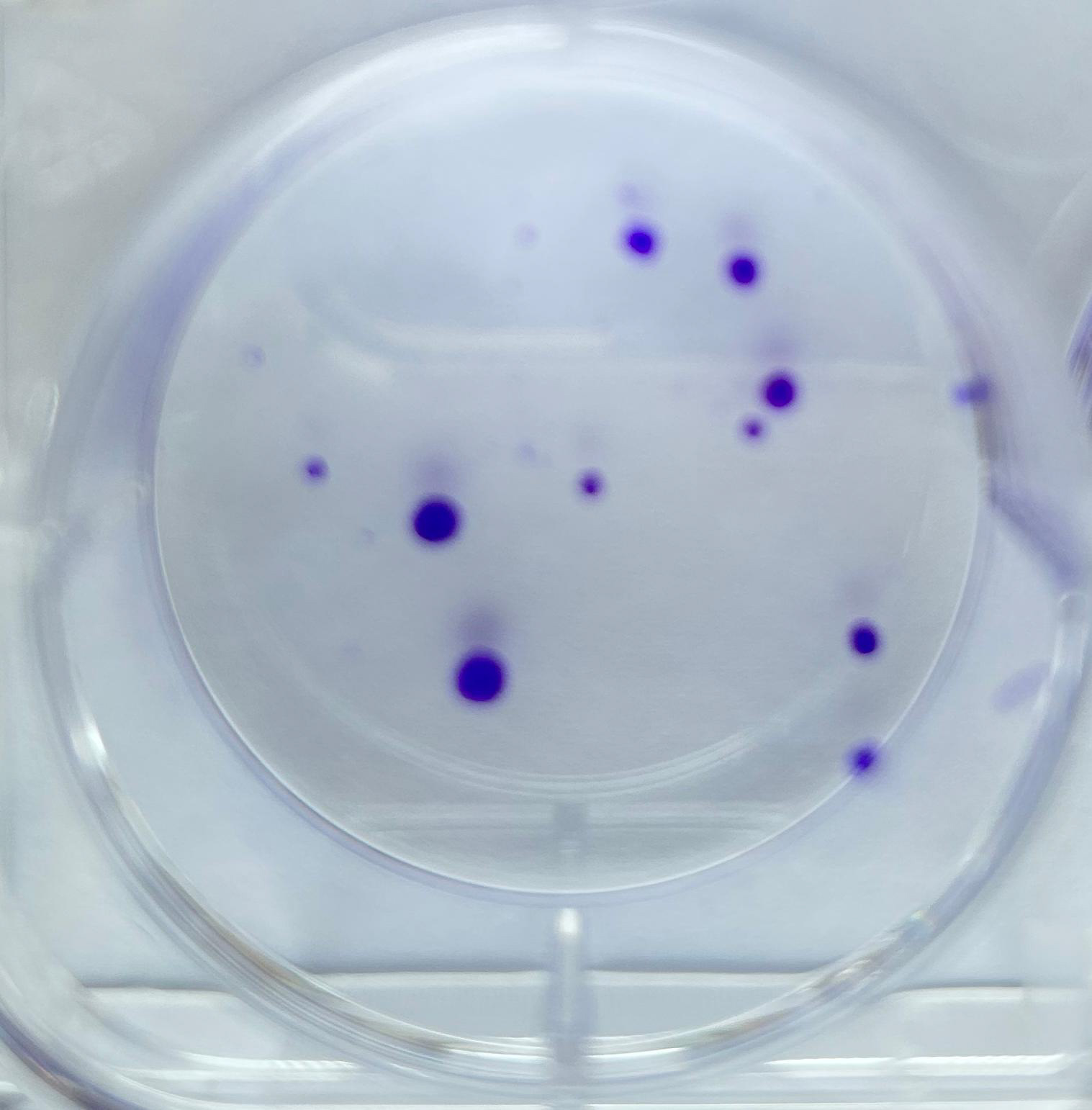

Supplement: Supplemental Information 13 [file peerj-12-18497-s013.zip › hucct1 functional experiment/control overexpression(NC OE)/hucct nc oe clone formation/picture/oe 孔3.jpg]

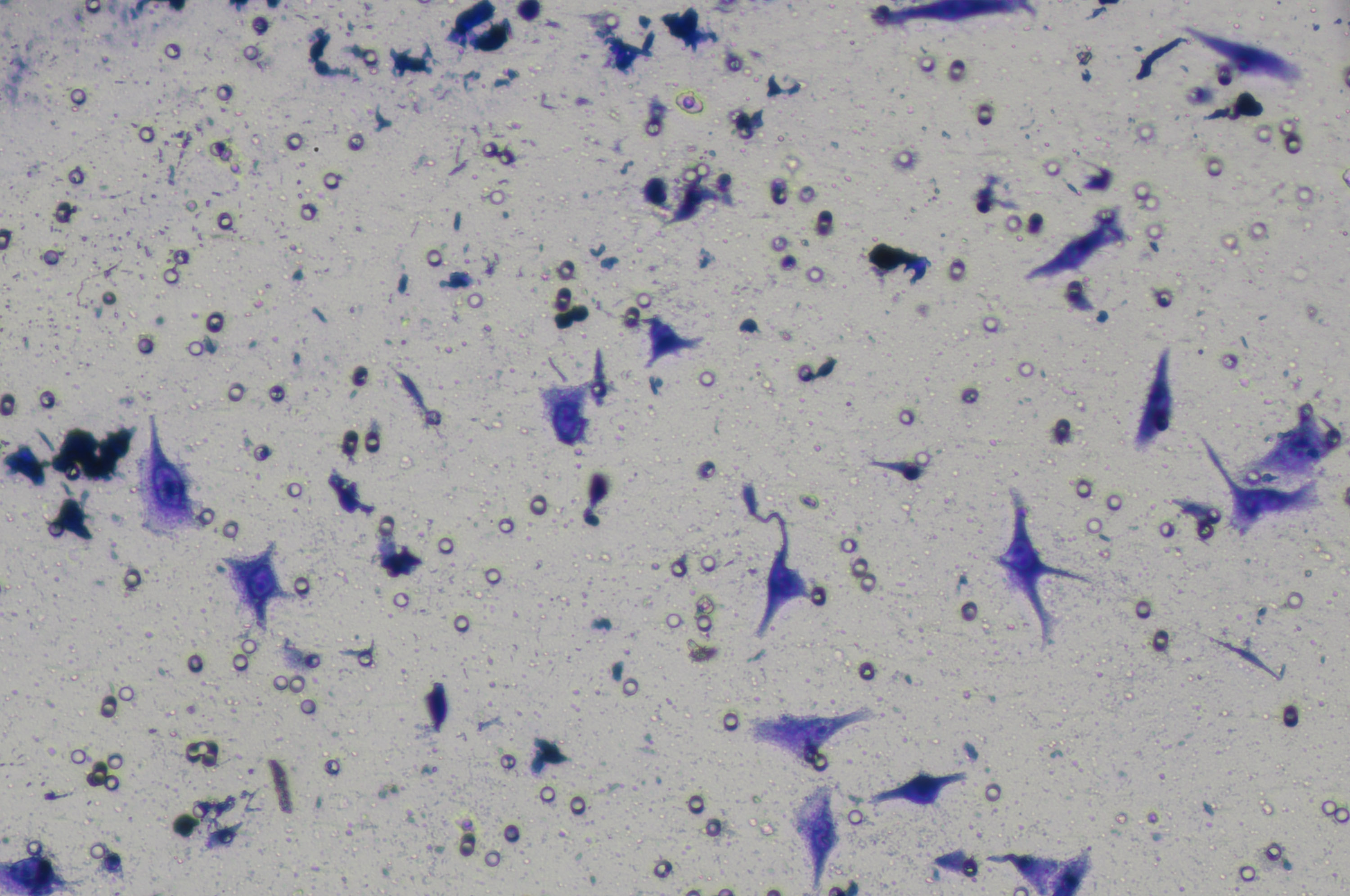

Supplement: Supplemental Information 13 [file peerj-12-18497-s013.zip › hucct1 functional experiment/control overexpression(NC OE)/hucct1 nc oe Invasion/picture/hucct 过表达 oe 第1孔 第1张.jpg]

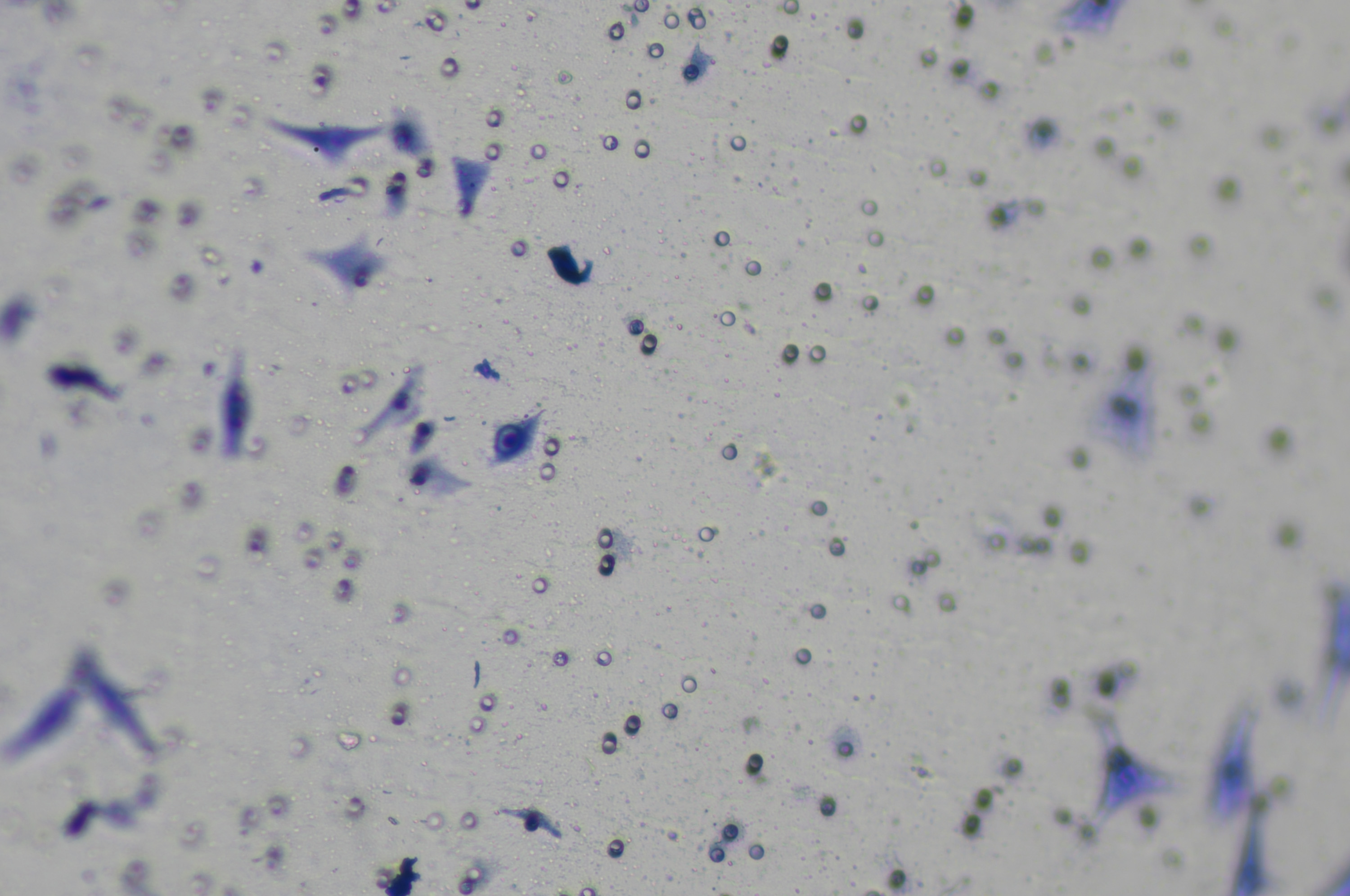

Supplement: Supplemental Information 13 [file peerj-12-18497-s013.zip › hucct1 functional experiment/control overexpression(NC OE)/hucct1 nc oe Invasion/picture/hucct 过表达 oe 第2孔 第1张.jpg]

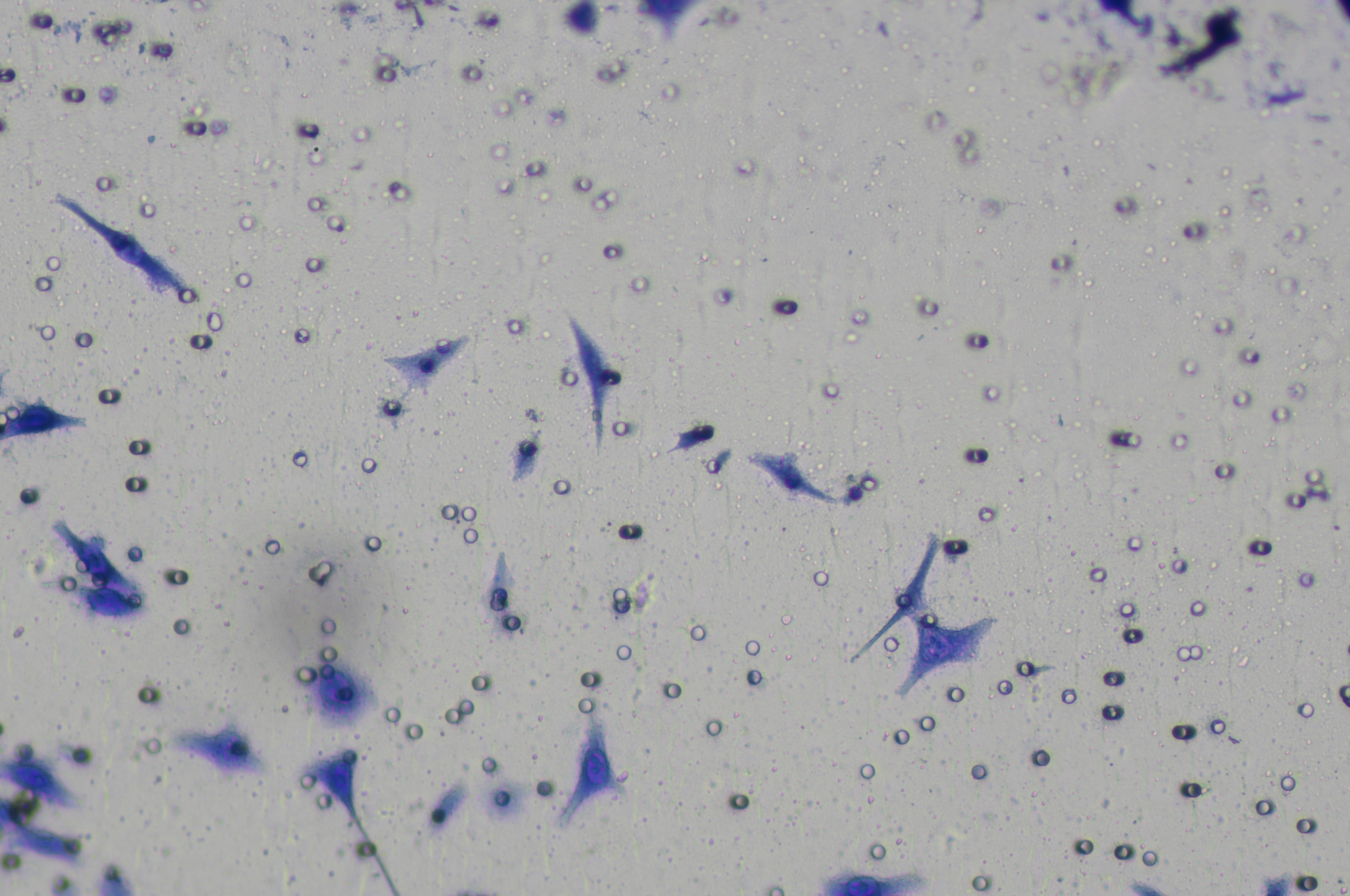

Supplement: Supplemental Information 13 [file peerj-12-18497-s013.zip › hucct1 functional experiment/control overexpression(NC OE)/hucct1 nc oe Invasion/picture/hucct 过表达 oe 第3孔 第1张.jpg]

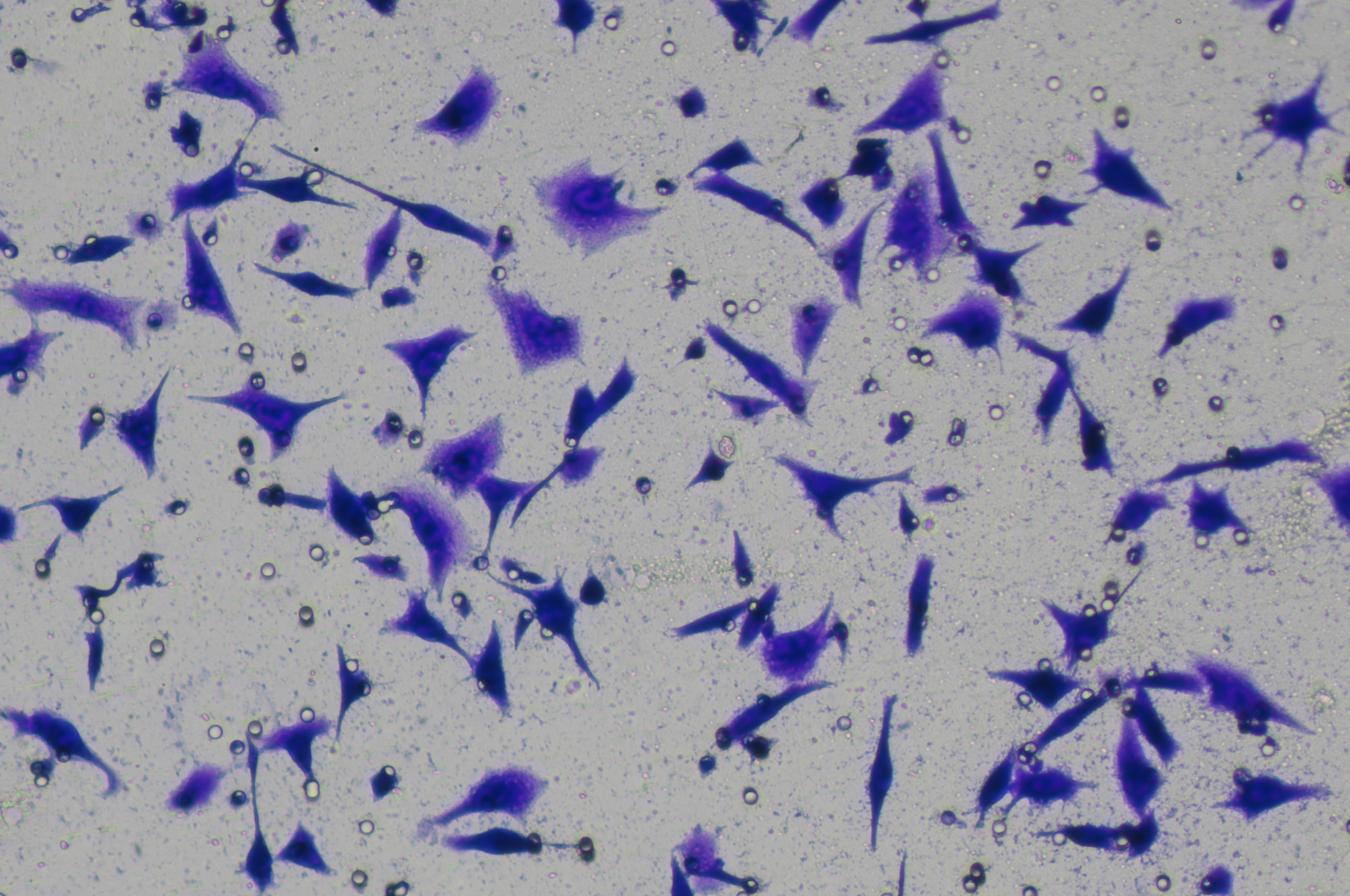

Supplement: Supplemental Information 13 [file peerj-12-18497-s013.zip › hucct1 functional experiment/control overexpression(NC OE)/hucct1 nc oe Invasion/picture/hucct 过表达的nc组 第1孔 第1张.jpg]

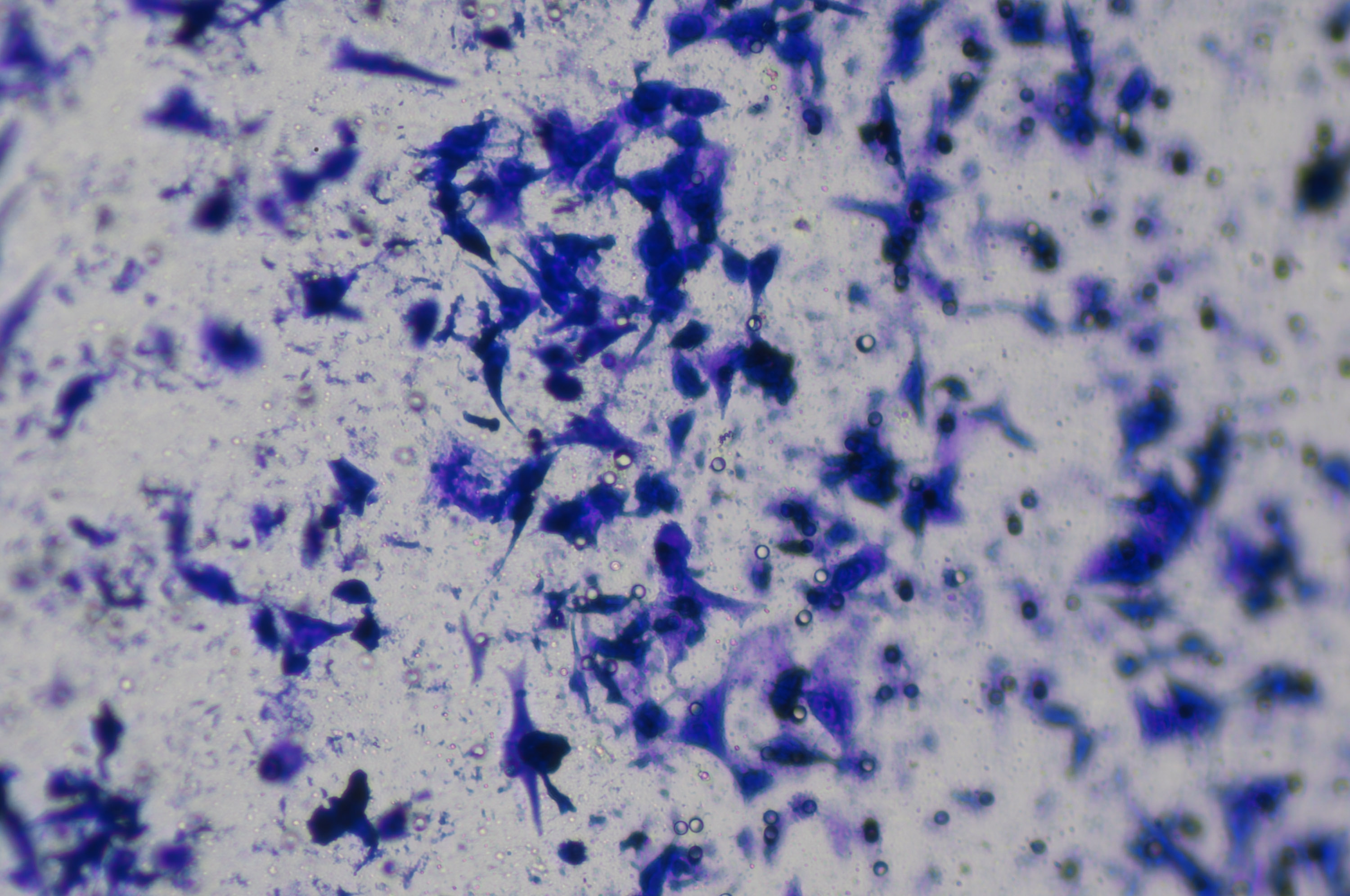

Supplement: Supplemental Information 13 [file peerj-12-18497-s013.zip › hucct1 functional experiment/control overexpression(NC OE)/hucct1 nc oe Invasion/picture/hucct 过表达的nc组 第2孔 第1张.jpg]

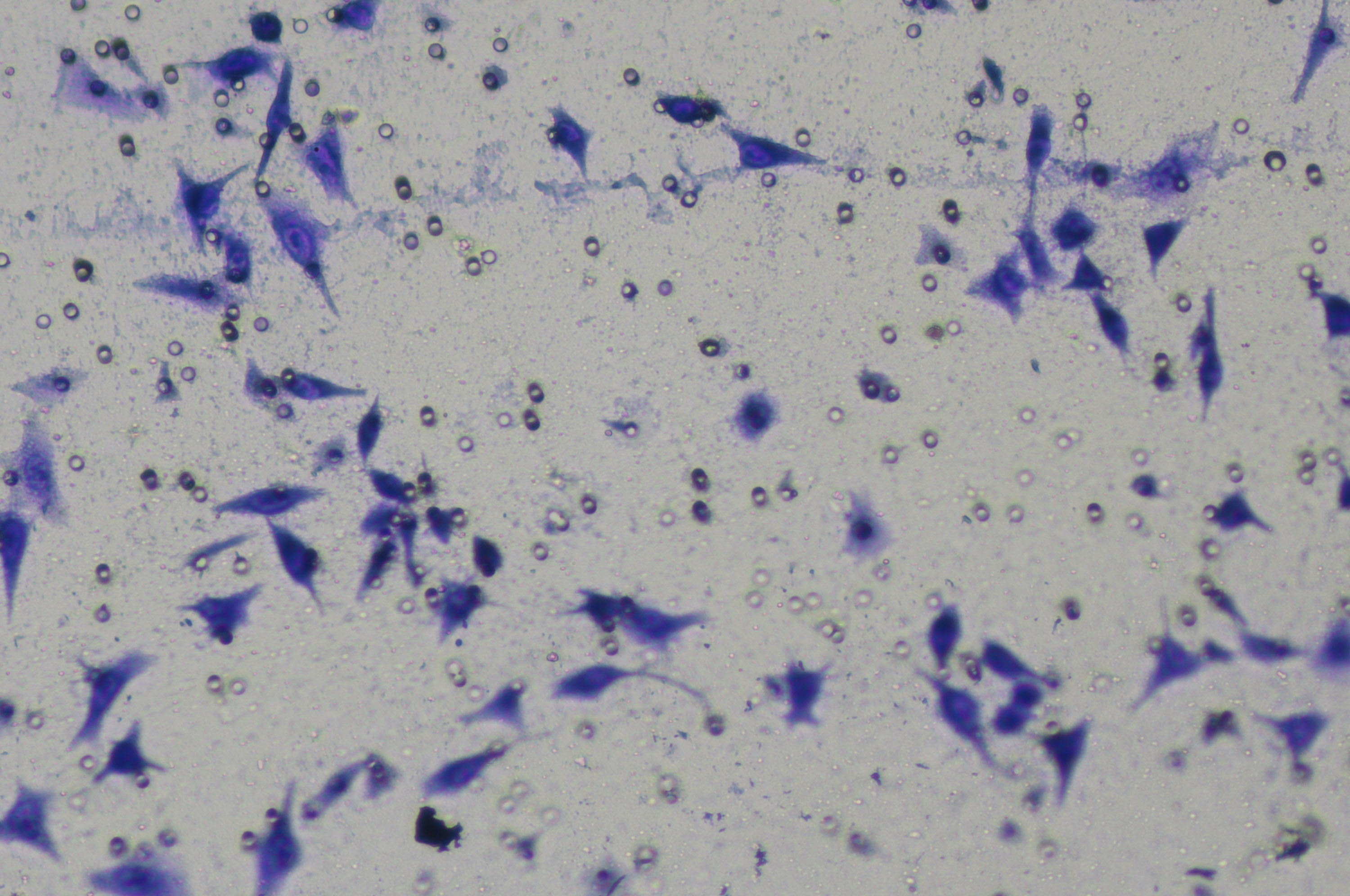

Supplement: Supplemental Information 13 [file peerj-12-18497-s013.zip › hucct1 functional experiment/control overexpression(NC OE)/hucct1 nc oe Invasion/picture/hucct 过表达的nc组 第3孔 第1张.jpg]

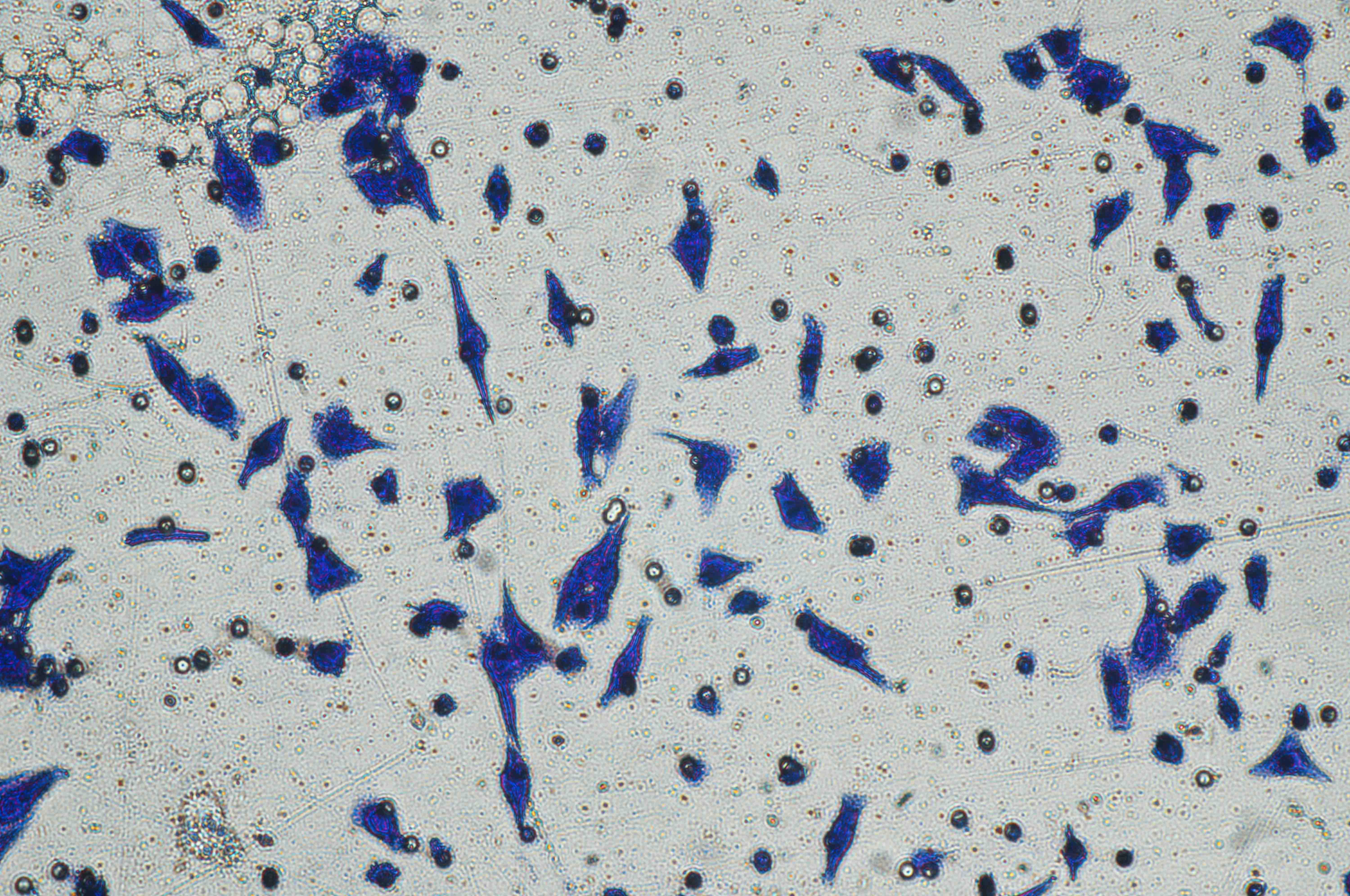

Supplement: Supplemental Information 13 [file peerj-12-18497-s013.zip › hucct1 functional experiment/control overexpression(NC OE)/hucct1 nc oe migration/picture/hucct clec3b nc孔1 20X033.jpg]

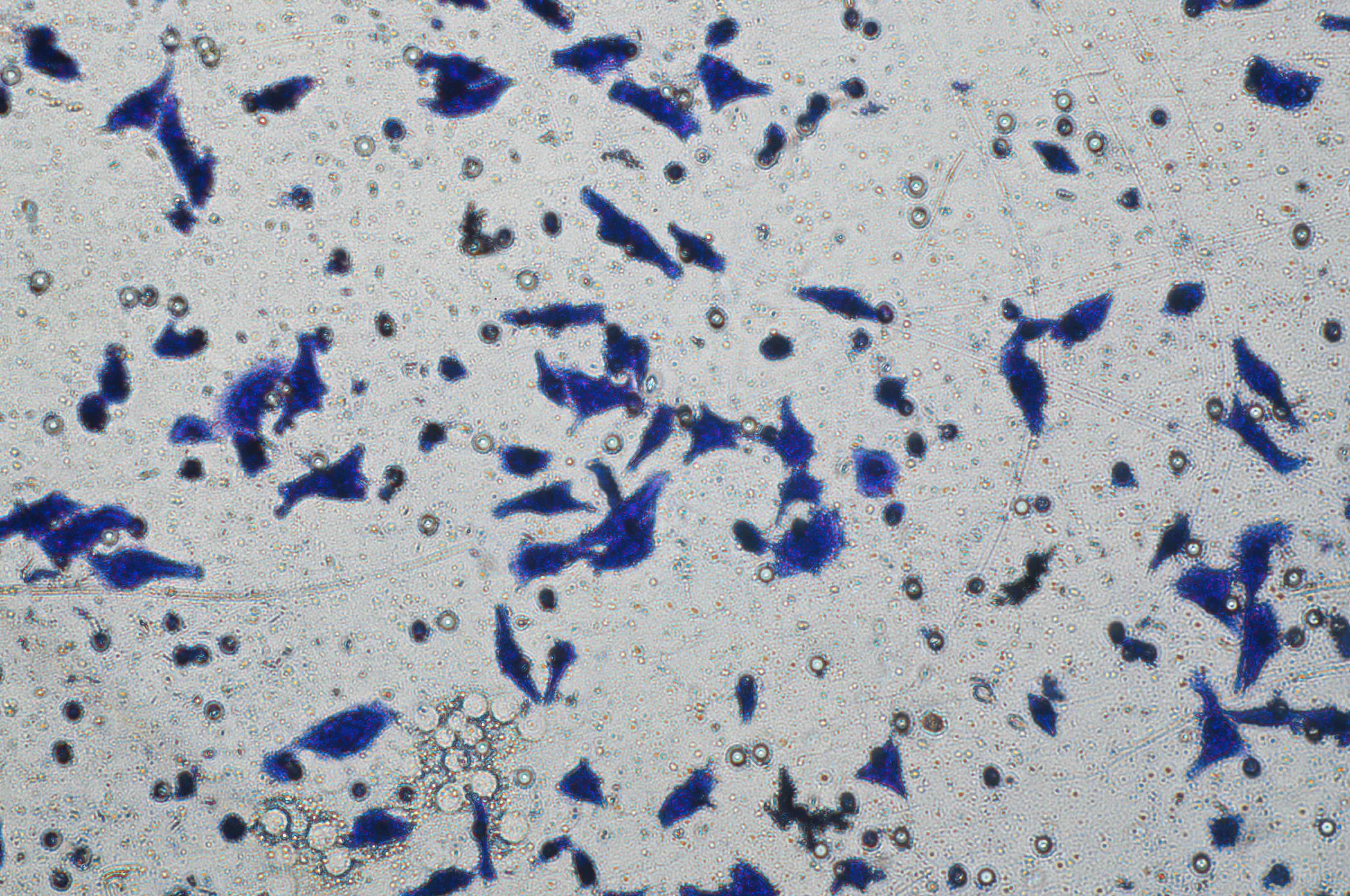

Supplement: Supplemental Information 13 [file peerj-12-18497-s013.zip › hucct1 functional experiment/control overexpression(NC OE)/hucct1 nc oe migration/picture/hucct clec3b nc孔2 20X041.jpg]

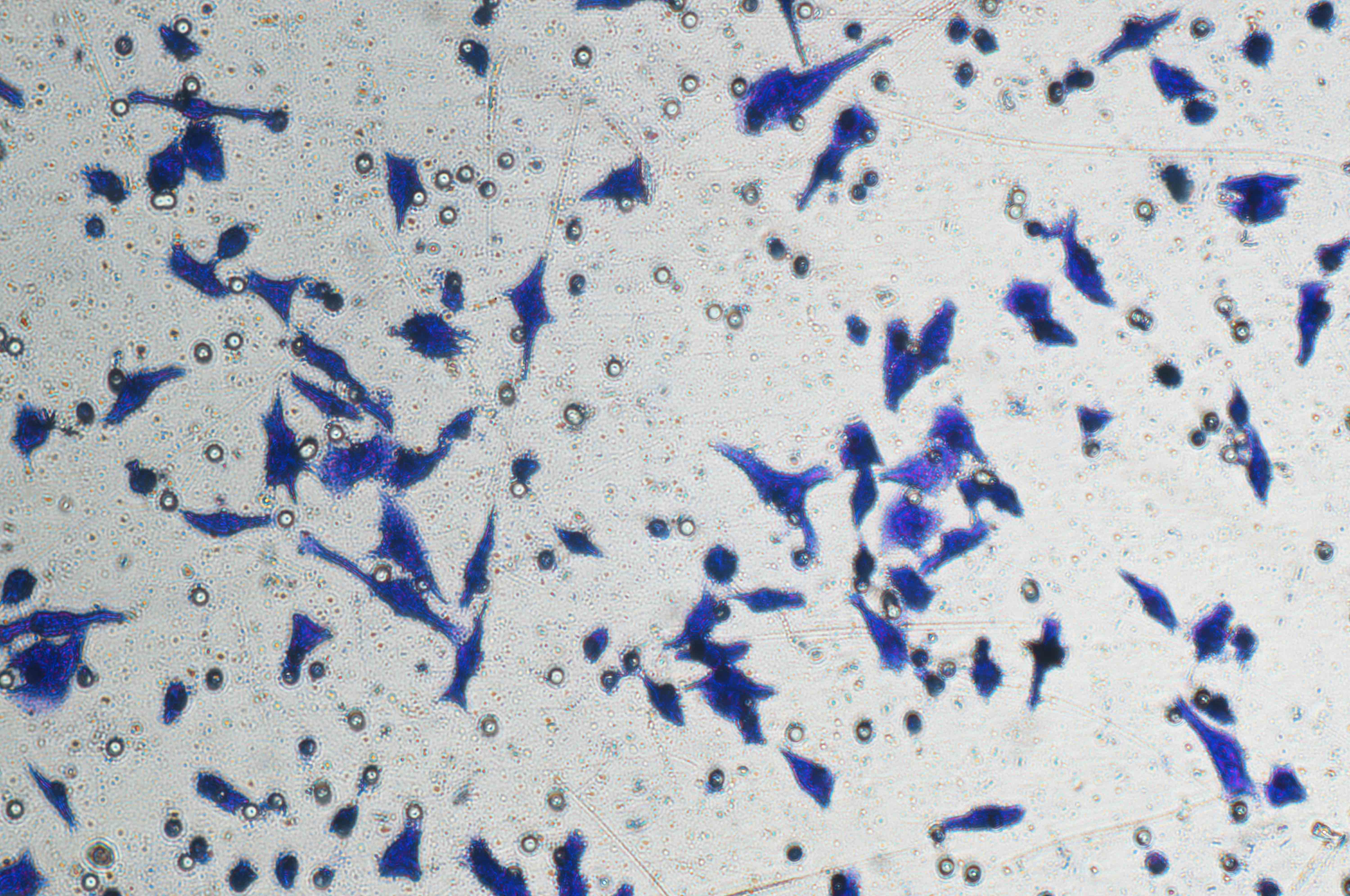

Supplement: Supplemental Information 13 [file peerj-12-18497-s013.zip › hucct1 functional experiment/control overexpression(NC OE)/hucct1 nc oe migration/picture/hucct clec3b nc孔3 20X050.jpg]

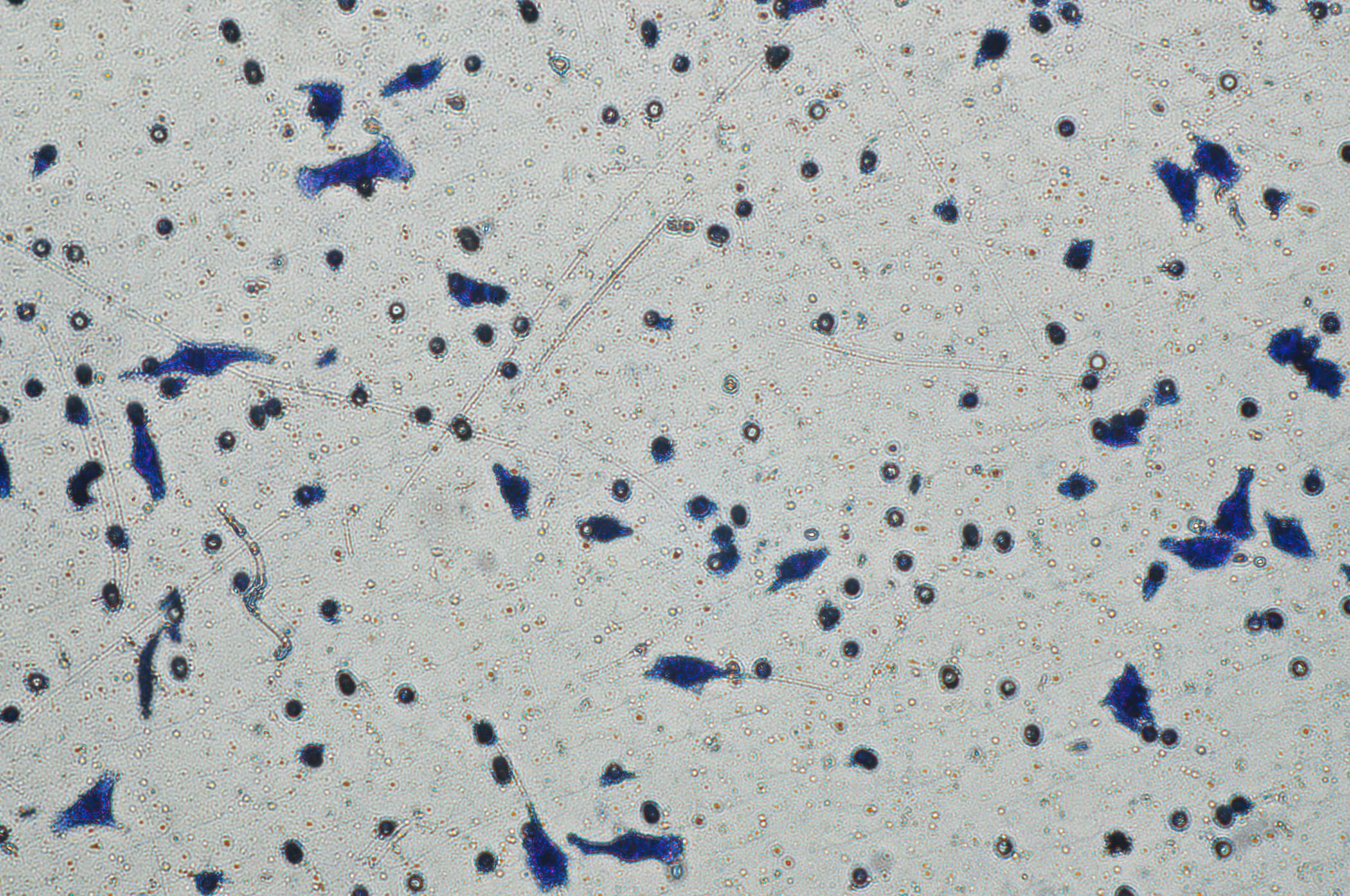

Supplement: Supplemental Information 13 [file peerj-12-18497-s013.zip › hucct1 functional experiment/control overexpression(NC OE)/hucct1 nc oe migration/picture/hucct clec3b oe孔1 20X057.jpg]

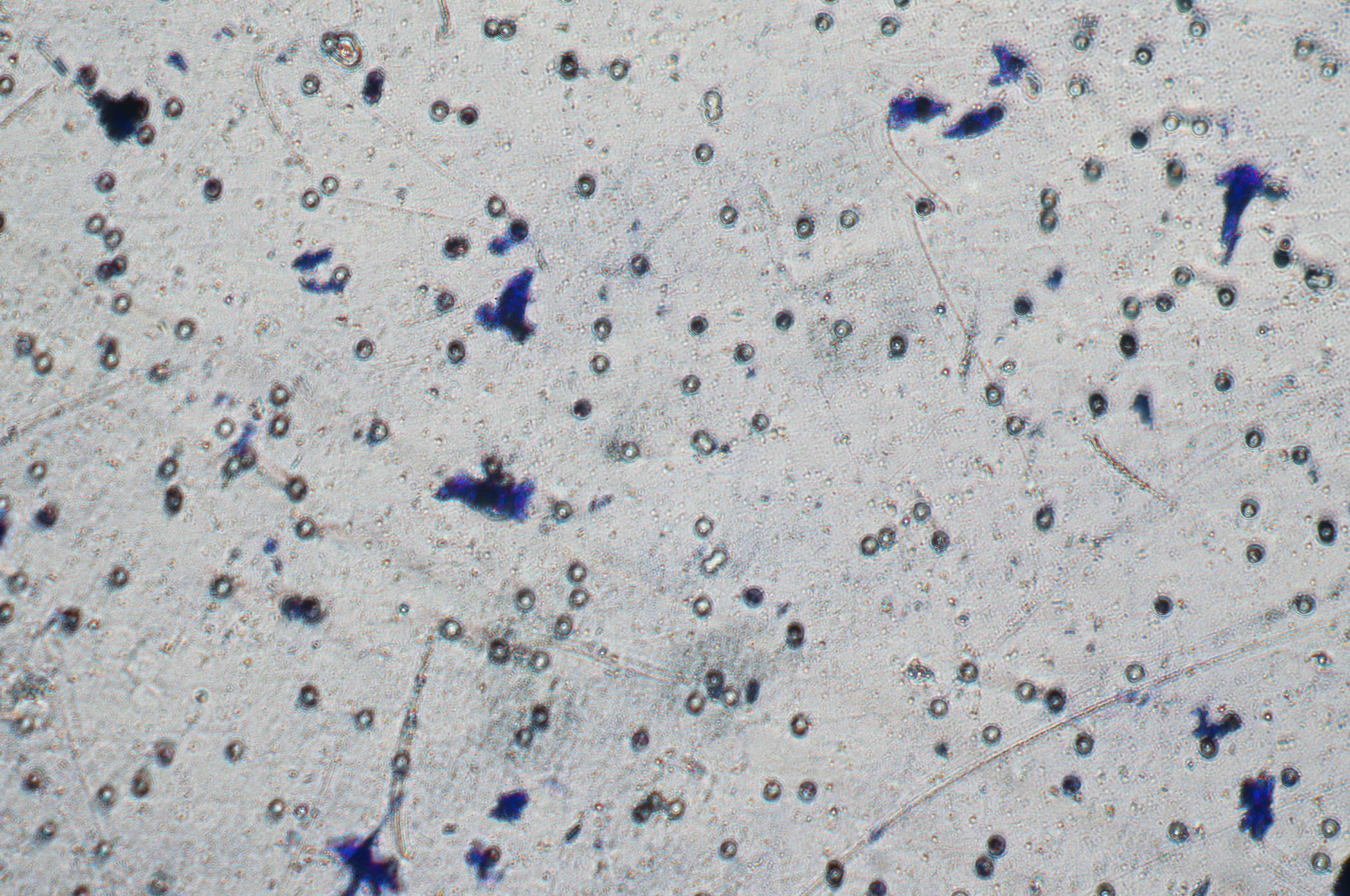

Supplement: Supplemental Information 13 [file peerj-12-18497-s013.zip › hucct1 functional experiment/control overexpression(NC OE)/hucct1 nc oe migration/picture/hucct clec3b oe孔2 20X070.jpg]

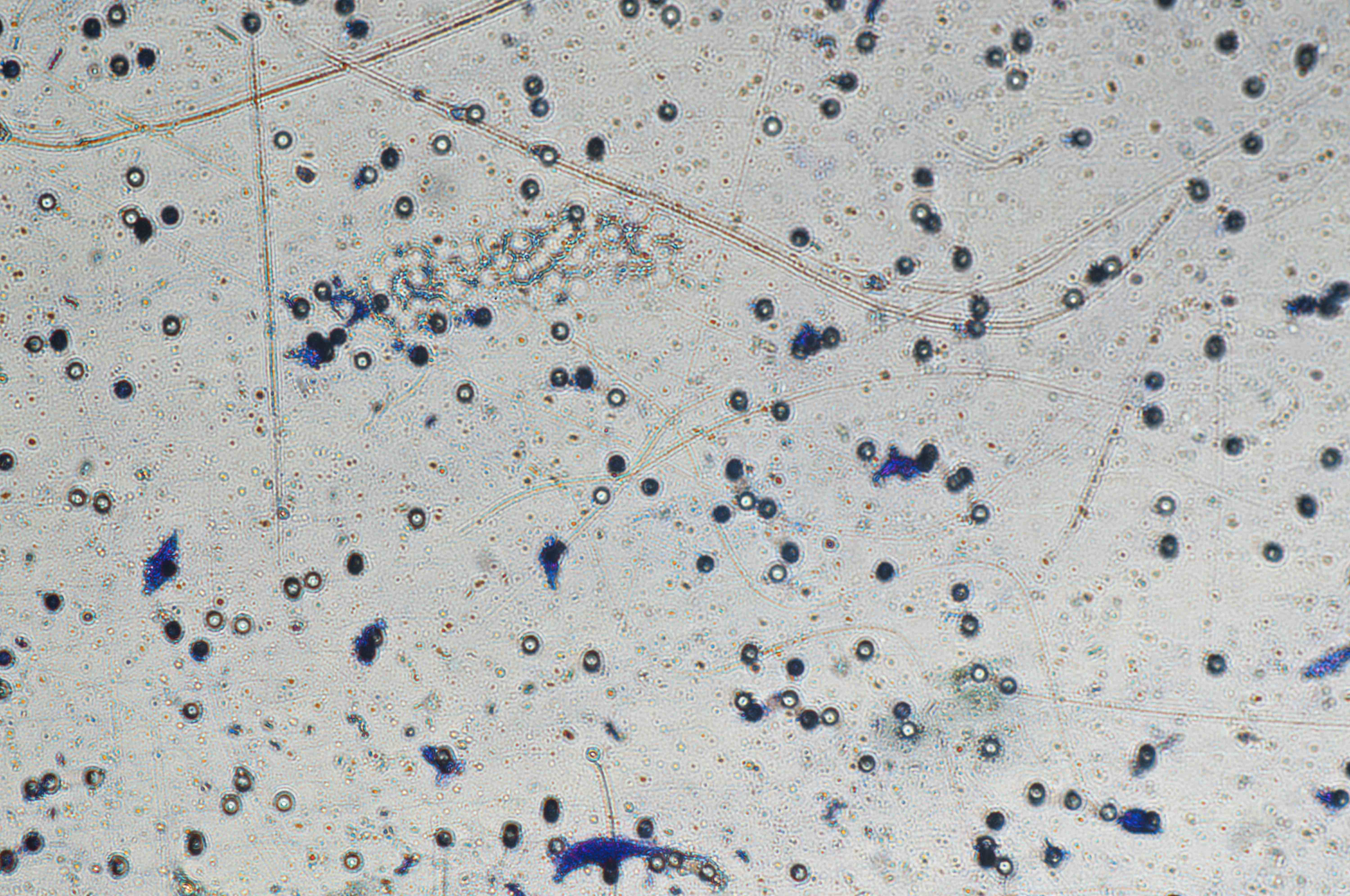

Supplement: Supplemental Information 13 [file peerj-12-18497-s013.zip › hucct1 functional experiment/control overexpression(NC OE)/hucct1 nc oe migration/picture/hucct clec3b oe孔3 20X076.jpg]

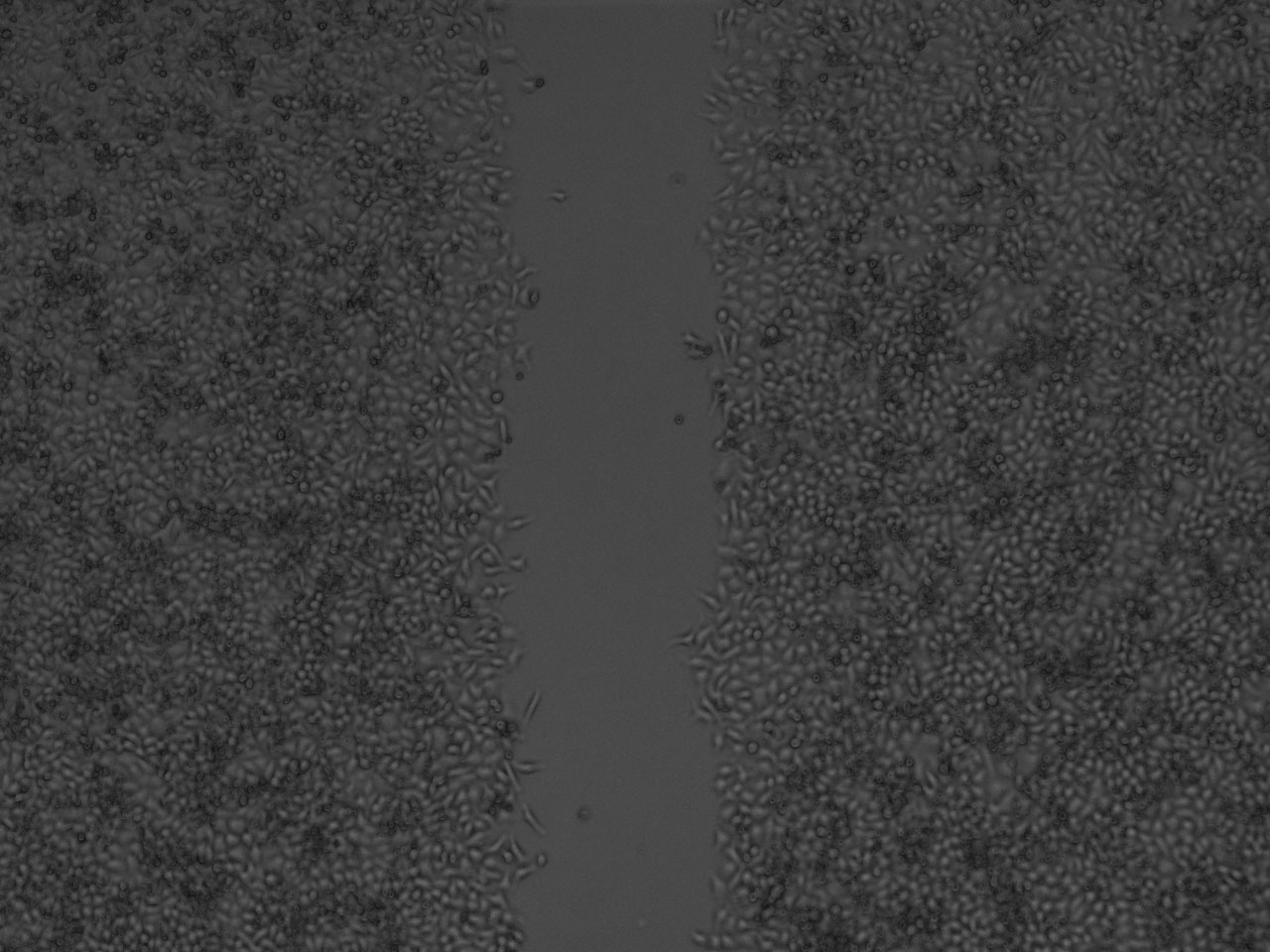

Supplement: Supplemental Information 13 [file peerj-12-18497-s013.zip › hucct1 functional experiment/control overexpression(NC OE)/hucct1 nc oe Wound Healing/picture/nc k1 j 0h.jpg]

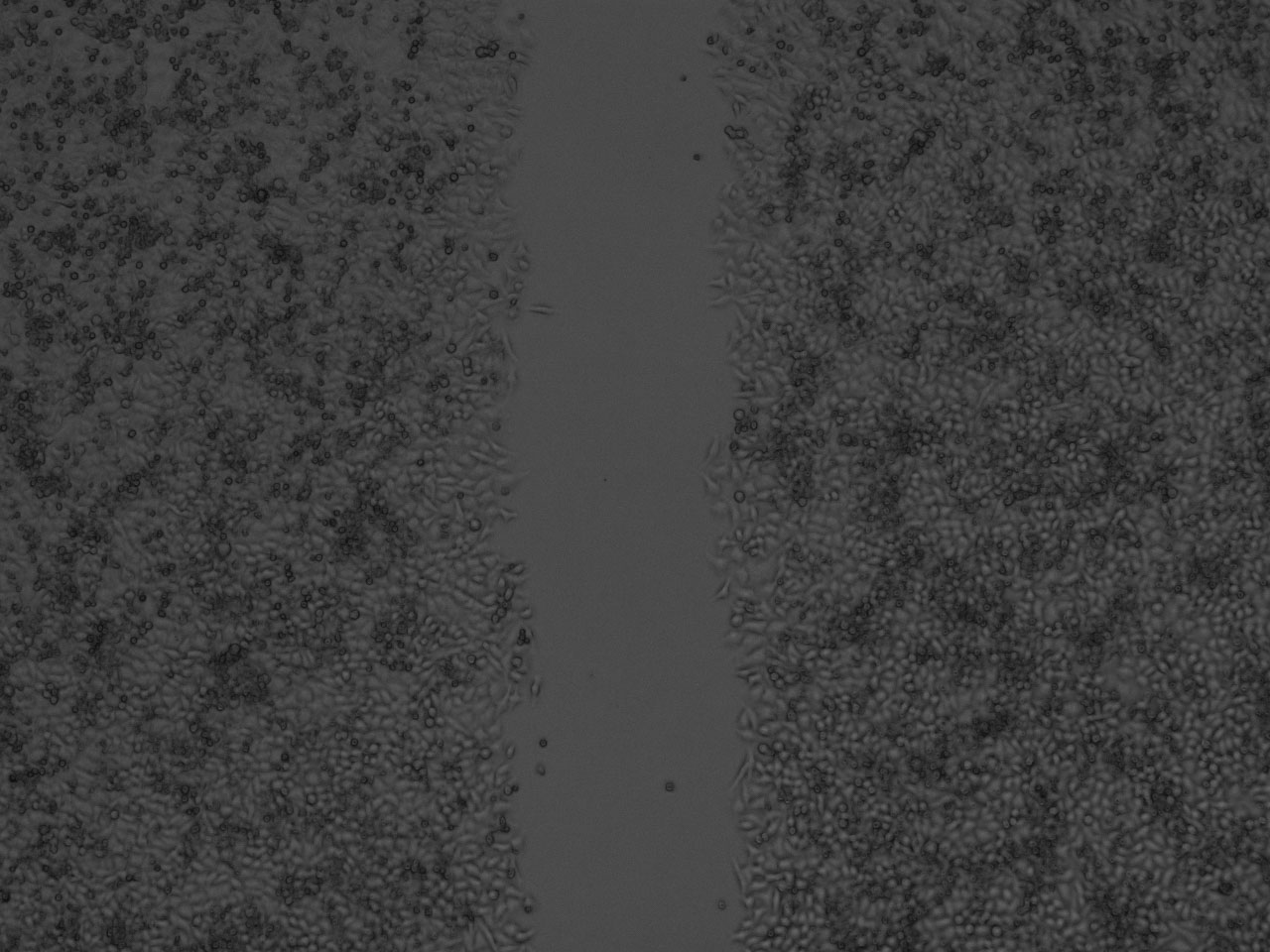

Supplement: Supplemental Information 13 [file peerj-12-18497-s013.zip › hucct1 functional experiment/control overexpression(NC OE)/hucct1 nc oe Wound Healing/picture/nc k1 k 0h.jpg]

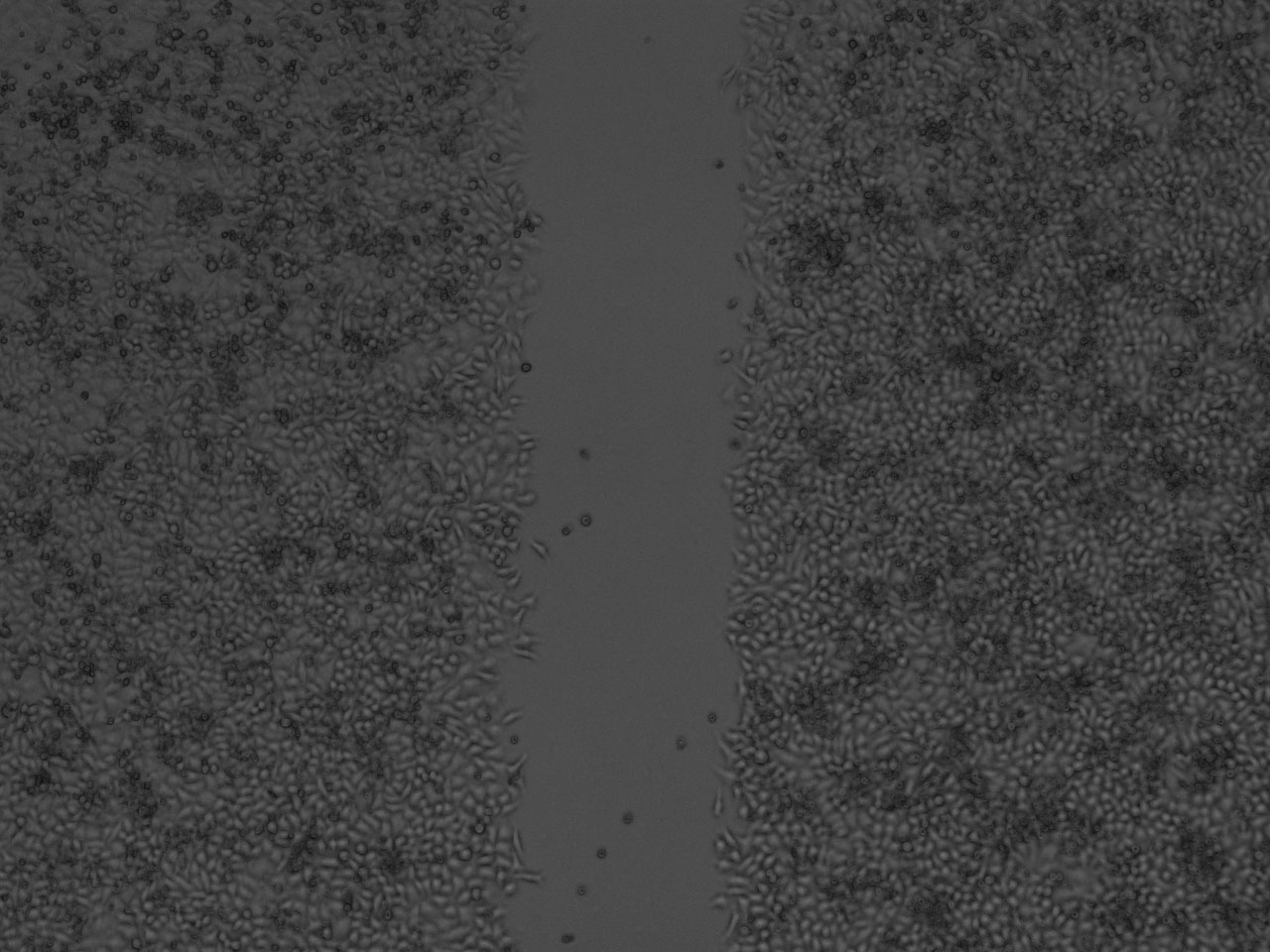

Supplement: Supplemental Information 13 [file peerj-12-18497-s013.zip › hucct1 functional experiment/control overexpression(NC OE)/hucct1 nc oe Wound Healing/picture/nc k1 l 0h.jpg]

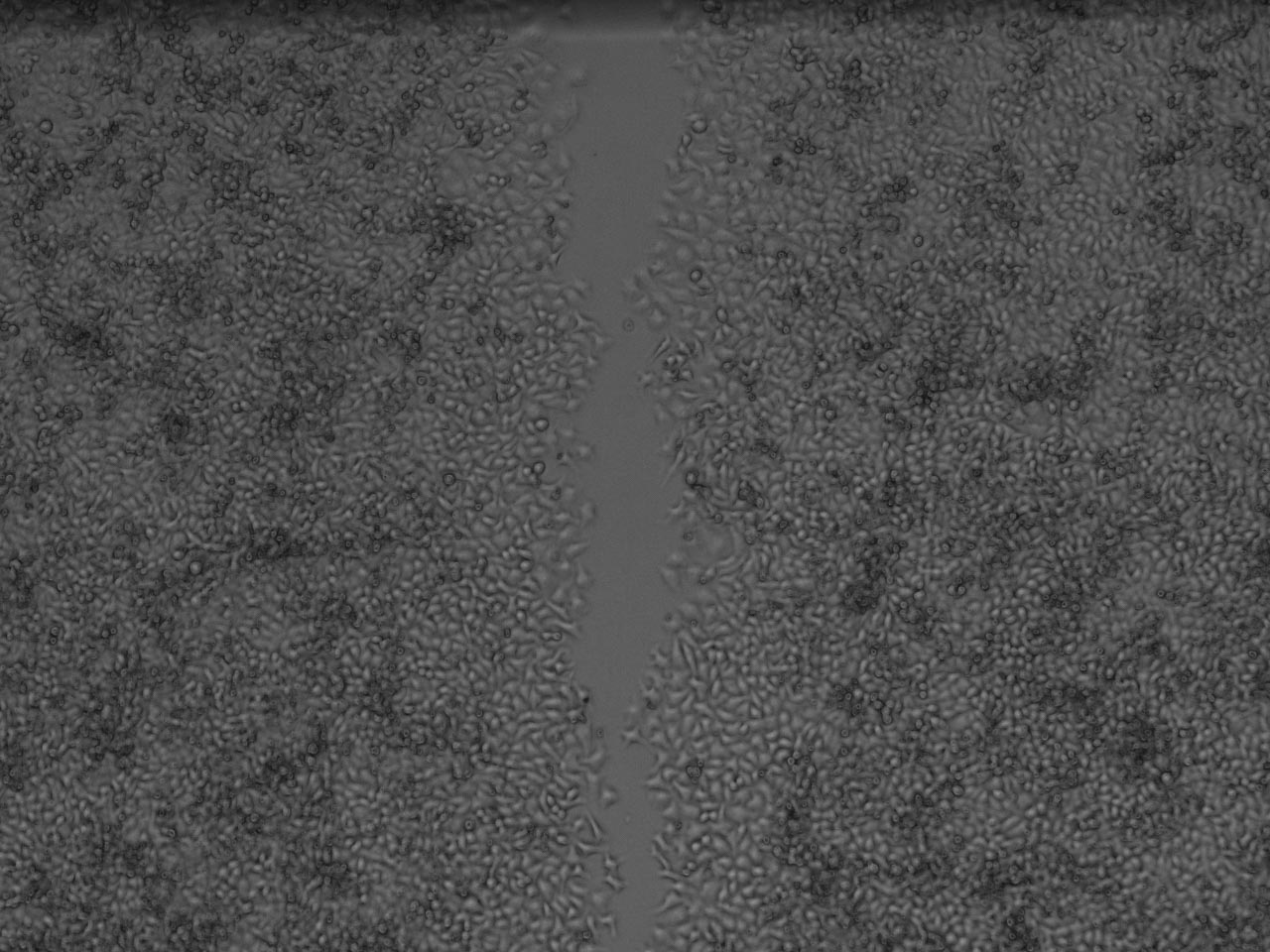

Supplement: Supplemental Information 13 [file peerj-12-18497-s013.zip › hucct1 functional experiment/control overexpression(NC OE)/hucct1 nc oe Wound Healing/picture/nc k1 j 24h.jpg]

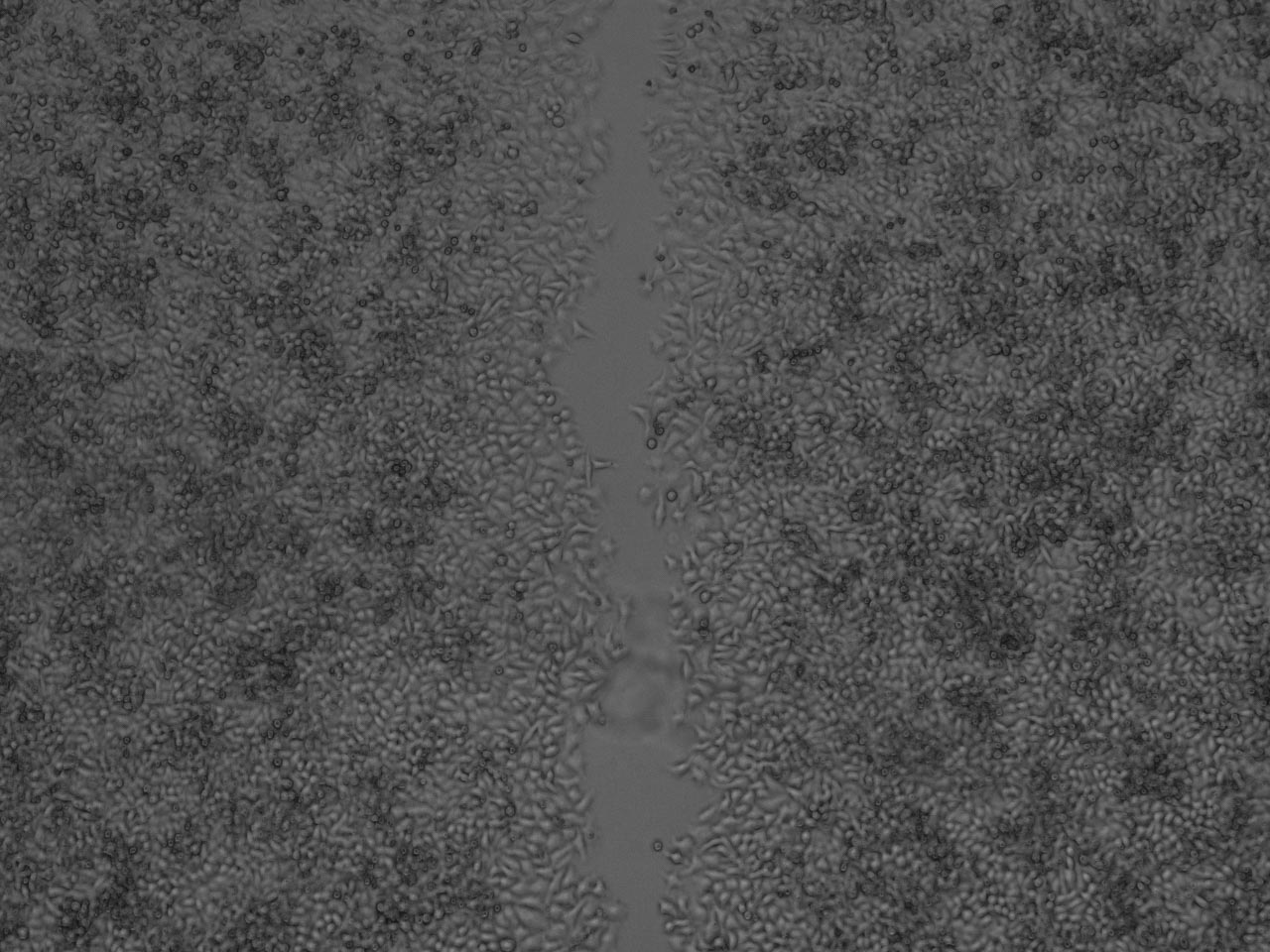

Supplement: Supplemental Information 13 [file peerj-12-18497-s013.zip › hucct1 functional experiment/control overexpression(NC OE)/hucct1 nc oe Wound Healing/picture/nc k1 k 24h.jpg]

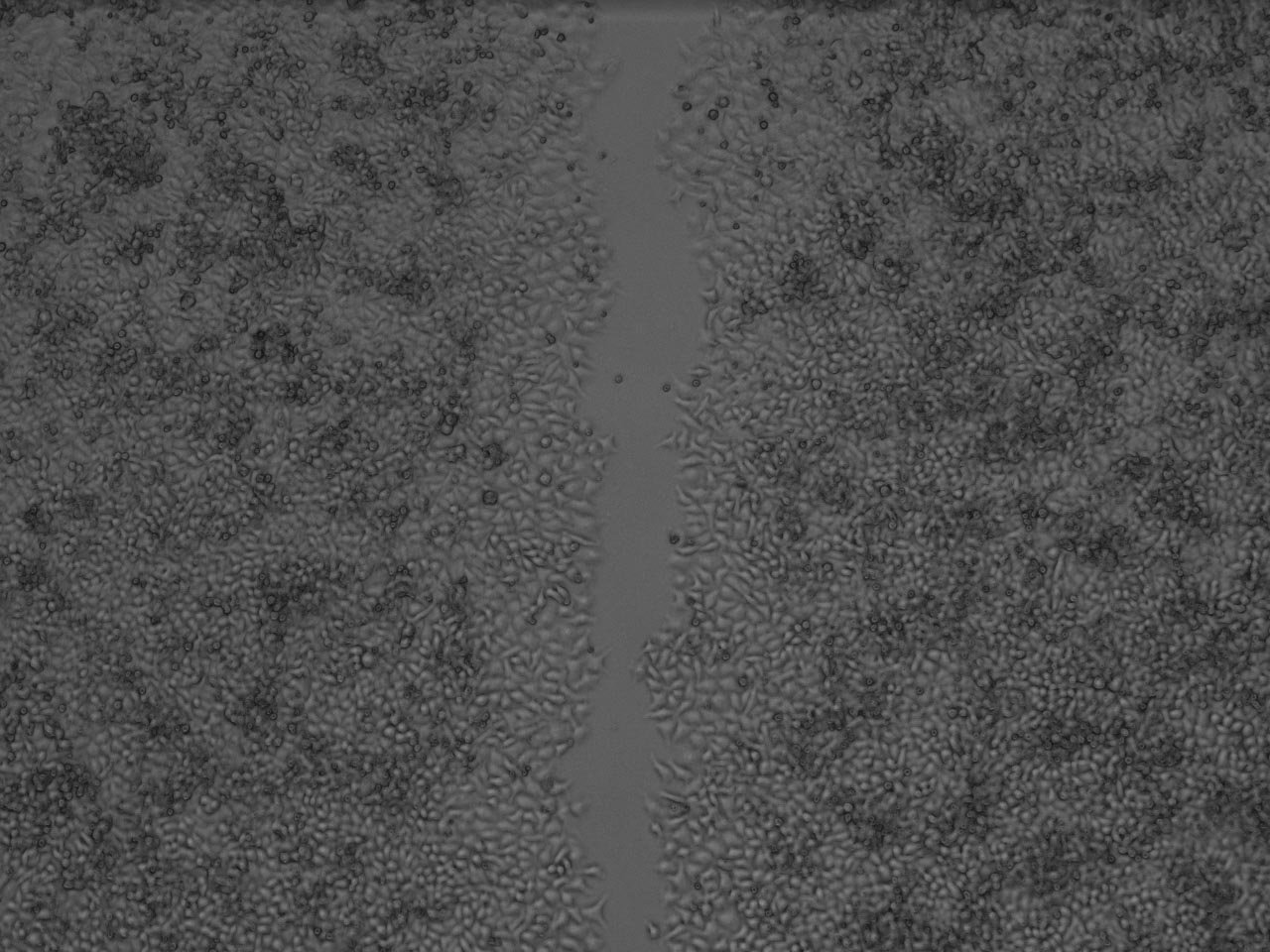

Supplement: Supplemental Information 13 [file peerj-12-18497-s013.zip › hucct1 functional experiment/control overexpression(NC OE)/hucct1 nc oe Wound Healing/picture/nc k1 l 24h.jpg]

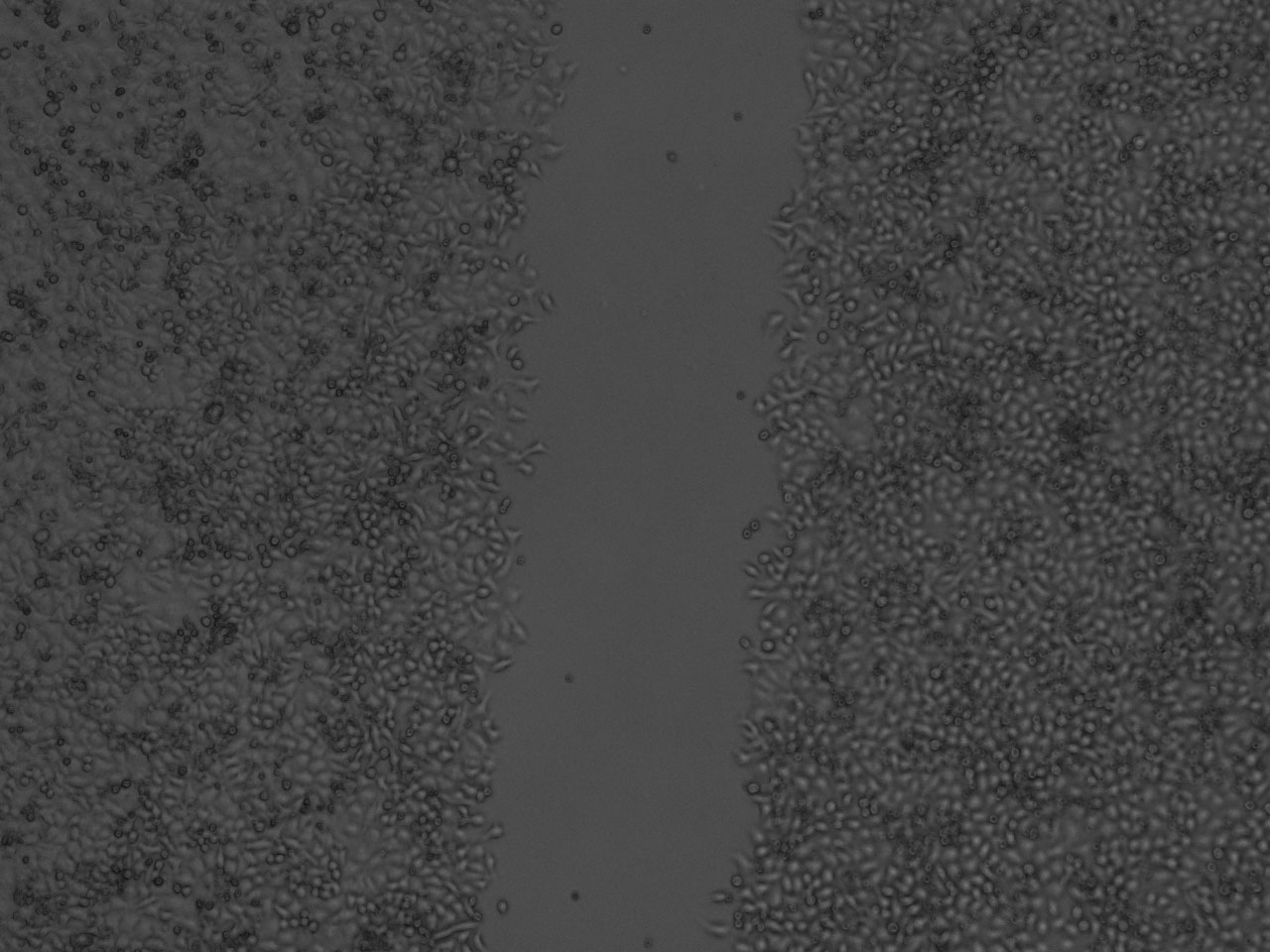

Supplement: Supplemental Information 13 [file peerj-12-18497-s013.zip › hucct1 functional experiment/control overexpression(NC OE)/hucct1 nc oe Wound Healing/picture/oe k1 k 0h.jpg]

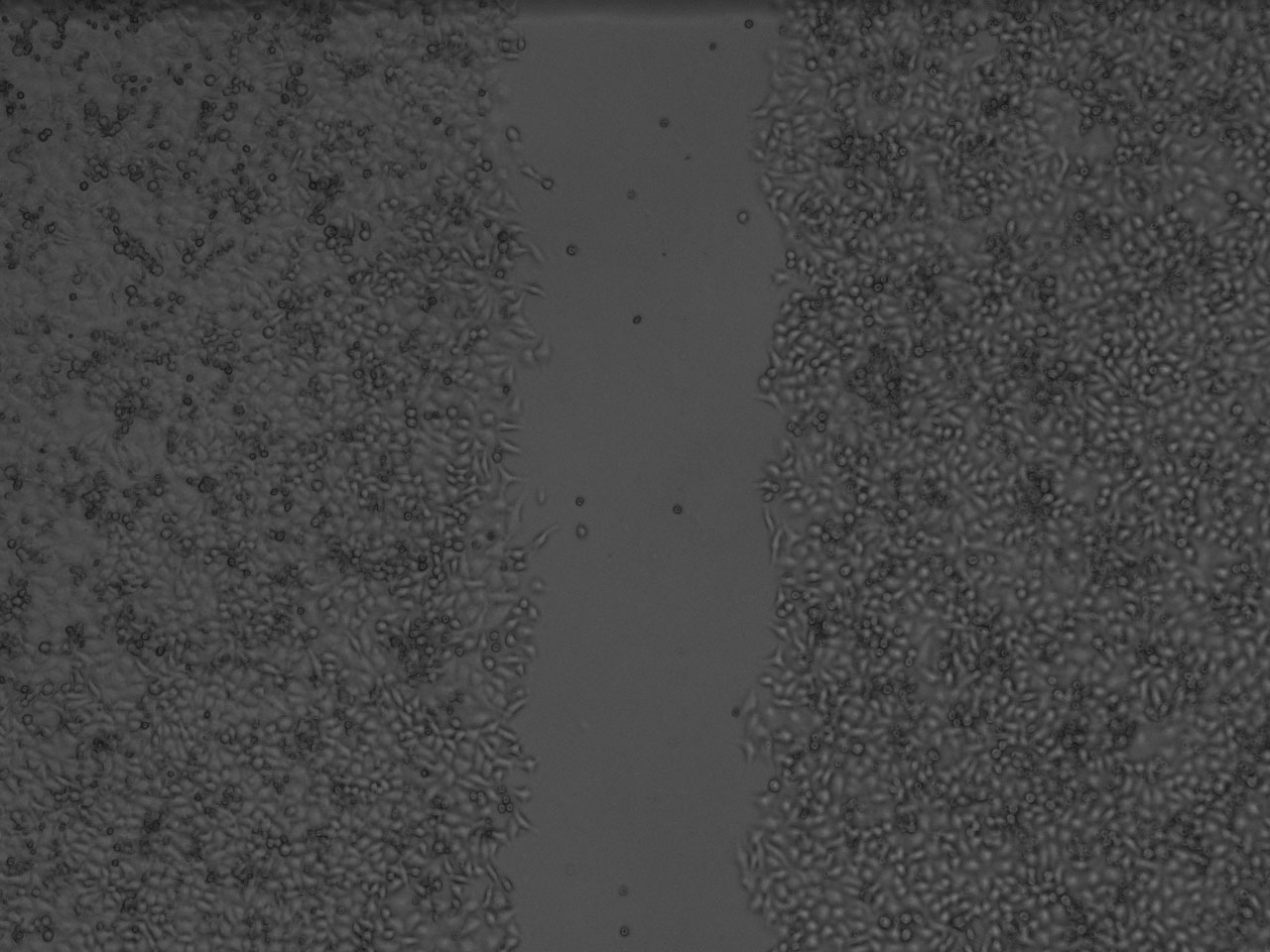

Supplement: Supplemental Information 13 [file peerj-12-18497-s013.zip › hucct1 functional experiment/control overexpression(NC OE)/hucct1 nc oe Wound Healing/picture/oe k1 l 0h.jpg]

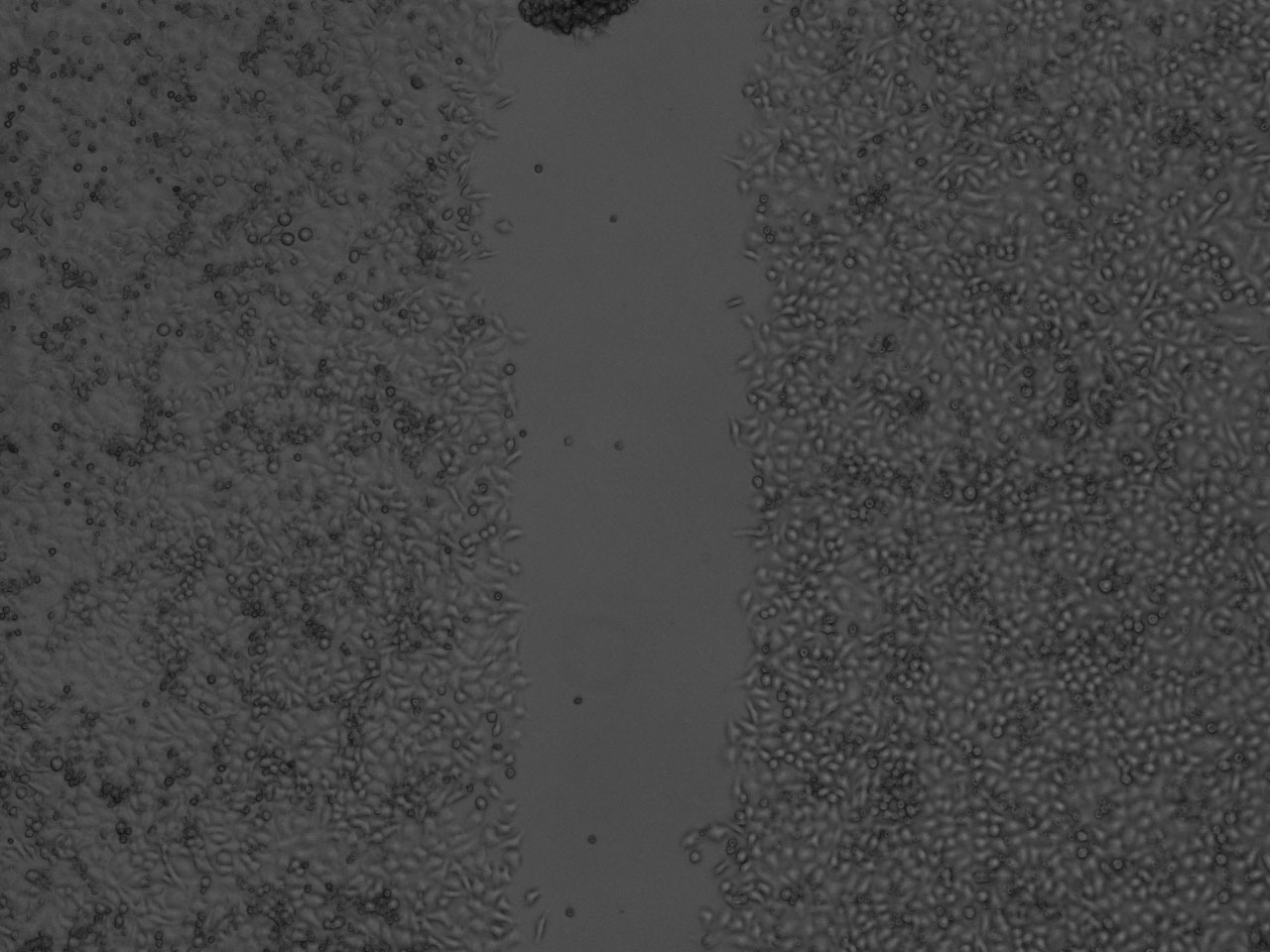

Supplement: Supplemental Information 13 [file peerj-12-18497-s013.zip › hucct1 functional experiment/control overexpression(NC OE)/hucct1 nc oe Wound Healing/picture/oe k1 m 0h.jpg]

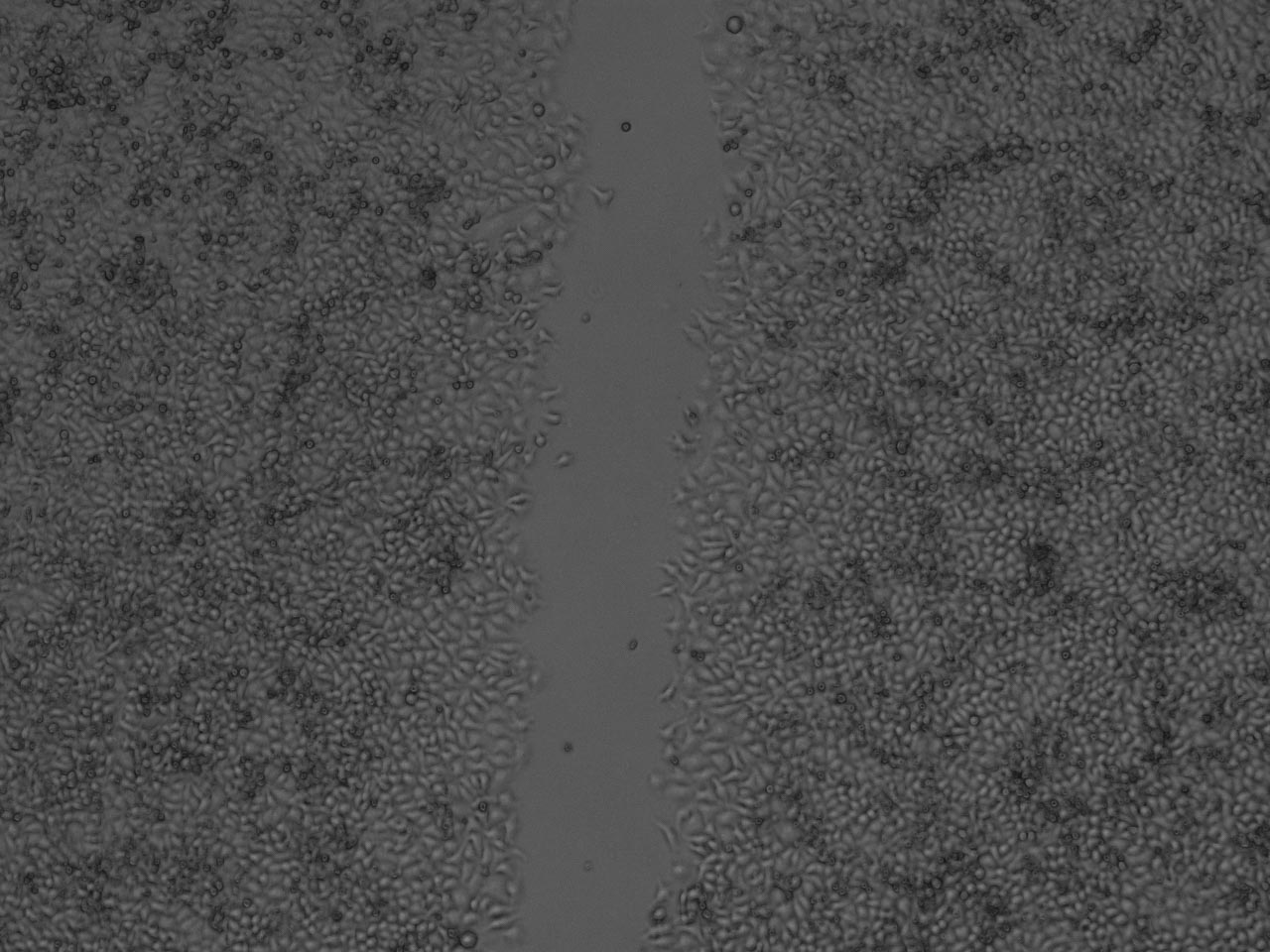

Supplement: Supplemental Information 13 [file peerj-12-18497-s013.zip › hucct1 functional experiment/control overexpression(NC OE)/hucct1 nc oe Wound Healing/picture/oe k1 k 24h.jpg]

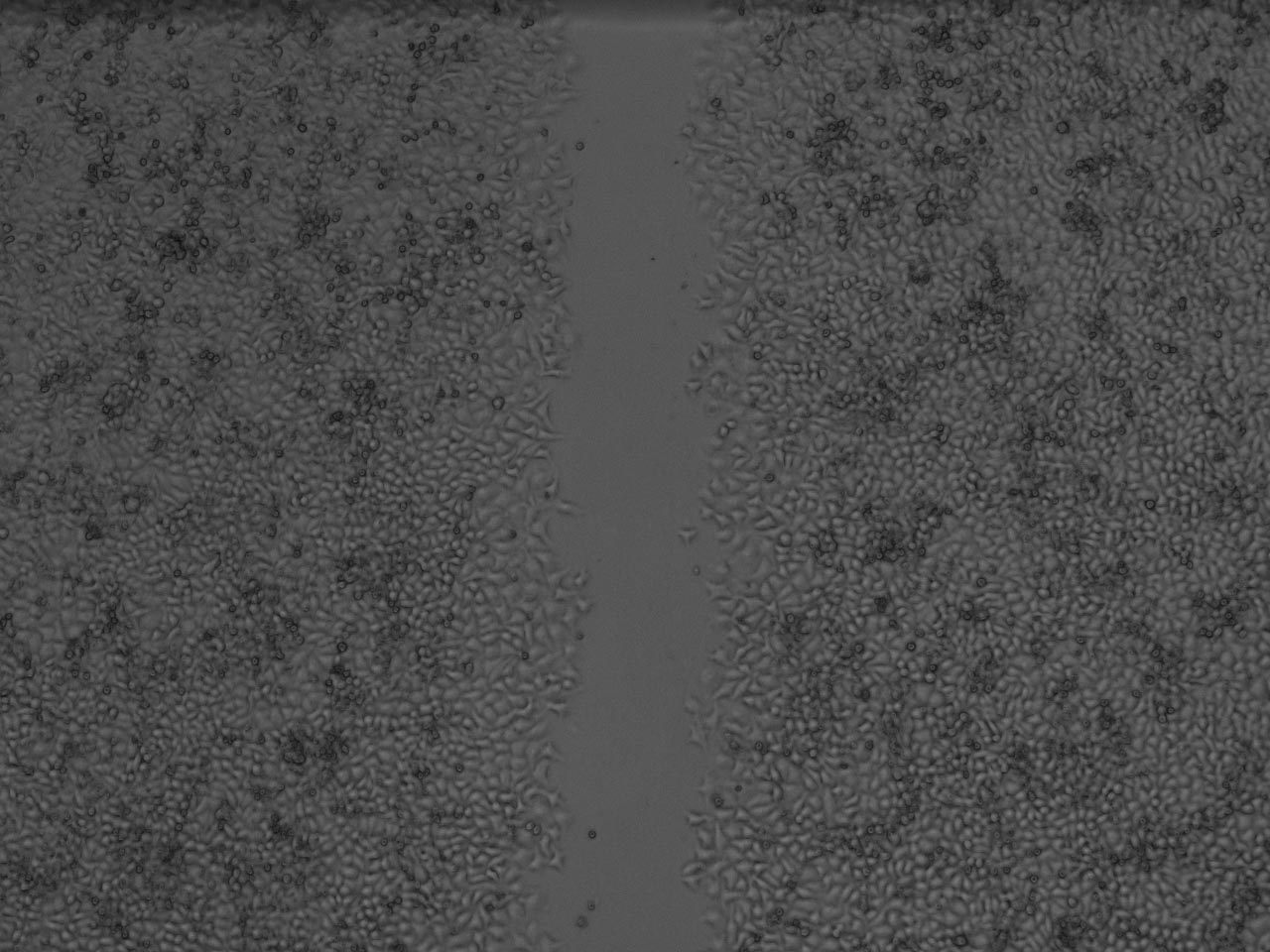

Supplement: Supplemental Information 13 [file peerj-12-18497-s013.zip › hucct1 functional experiment/control overexpression(NC OE)/hucct1 nc oe Wound Healing/picture/oe k1 l 24h.jpg]

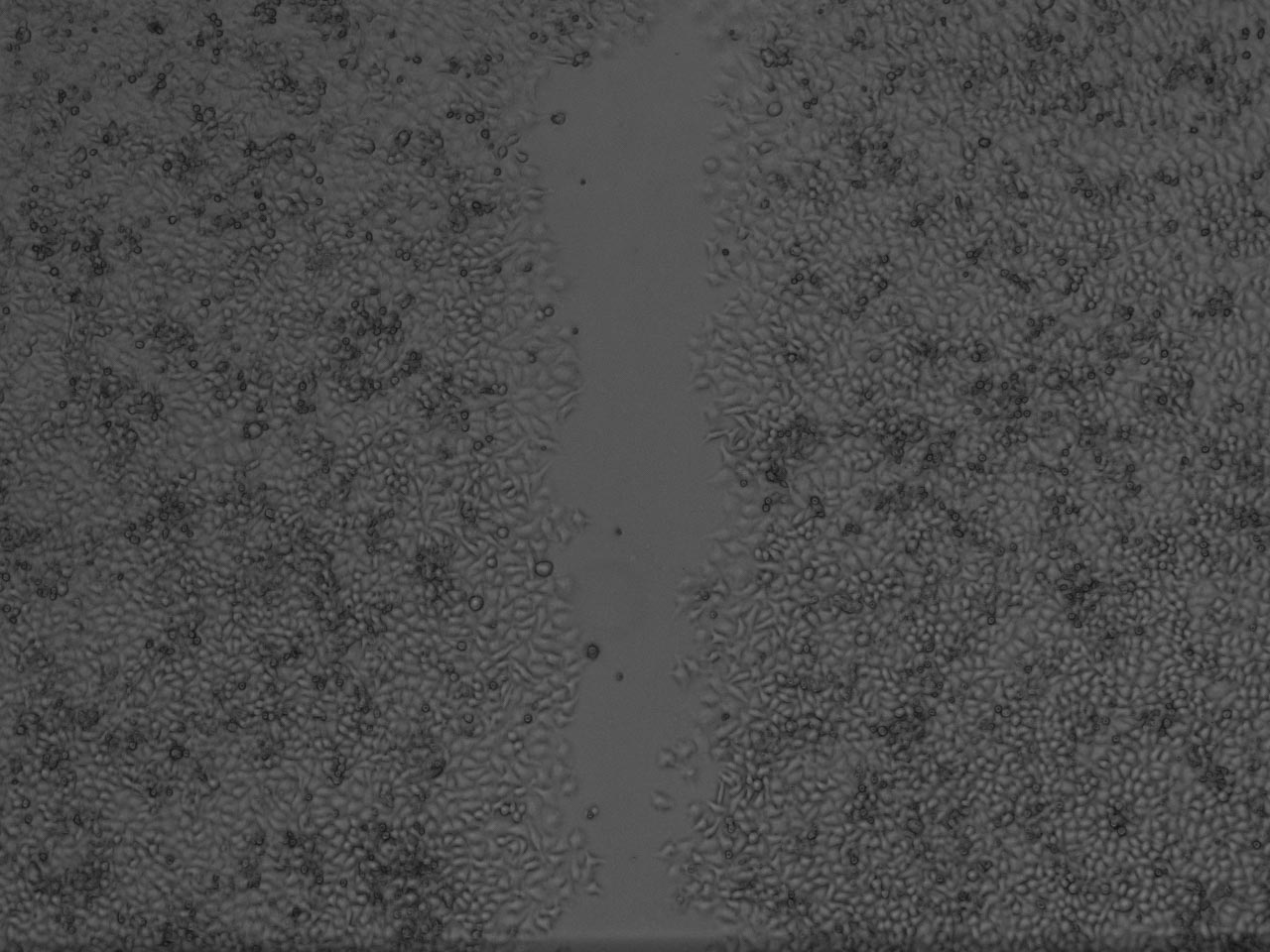

Supplement: Supplemental Information 13 [file peerj-12-18497-s013.zip › hucct1 functional experiment/control overexpression(NC OE)/hucct1 nc oe Wound Healing/picture/oe k1 m 24h.jpg]

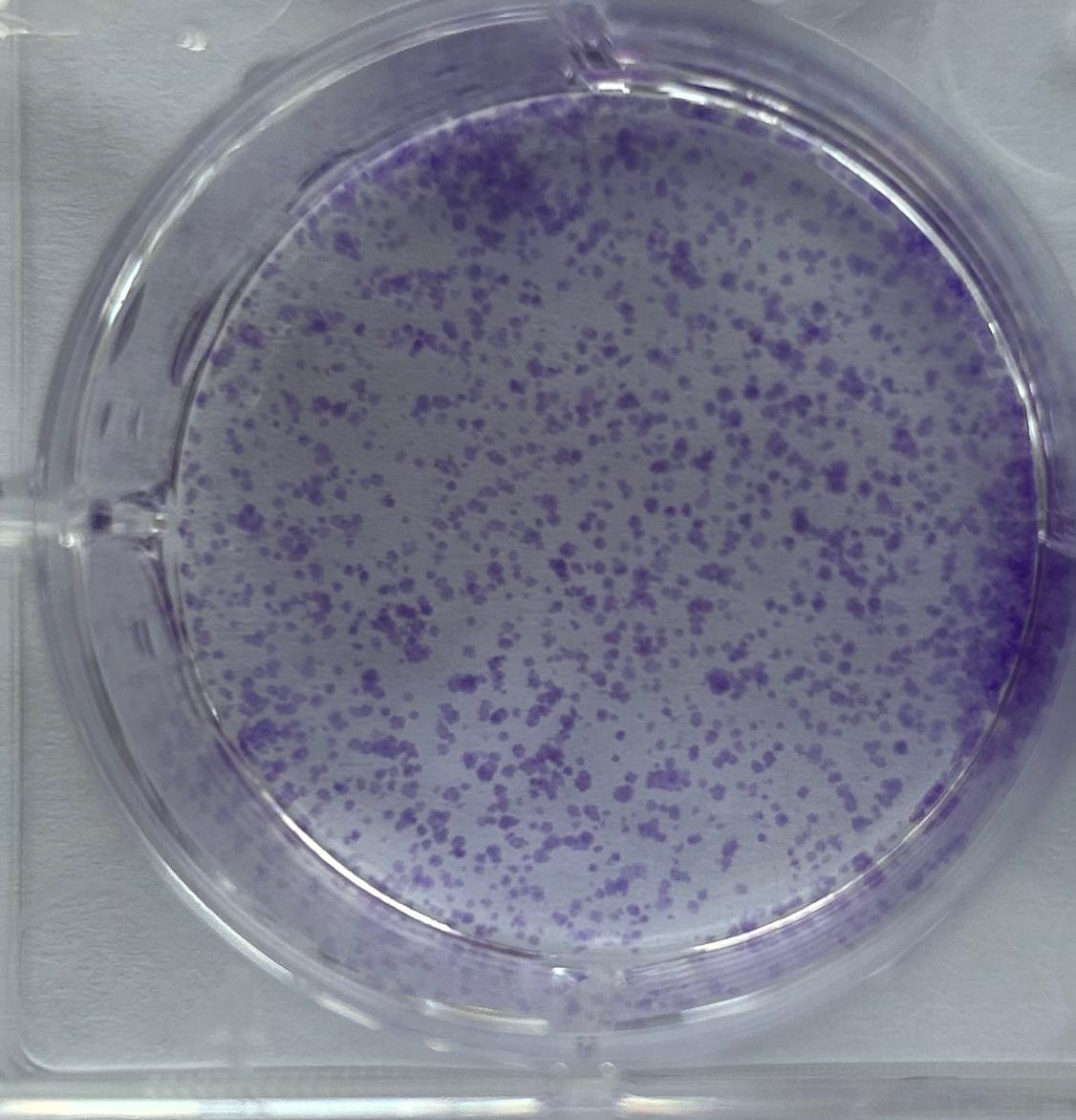

Supplement: Supplemental Information 13 [file peerj-12-18497-s013.zip › hucct1 functional experiment/NC knockdown (NC SI)/hucct nc si clone formation/picture/si185-1.jpg]

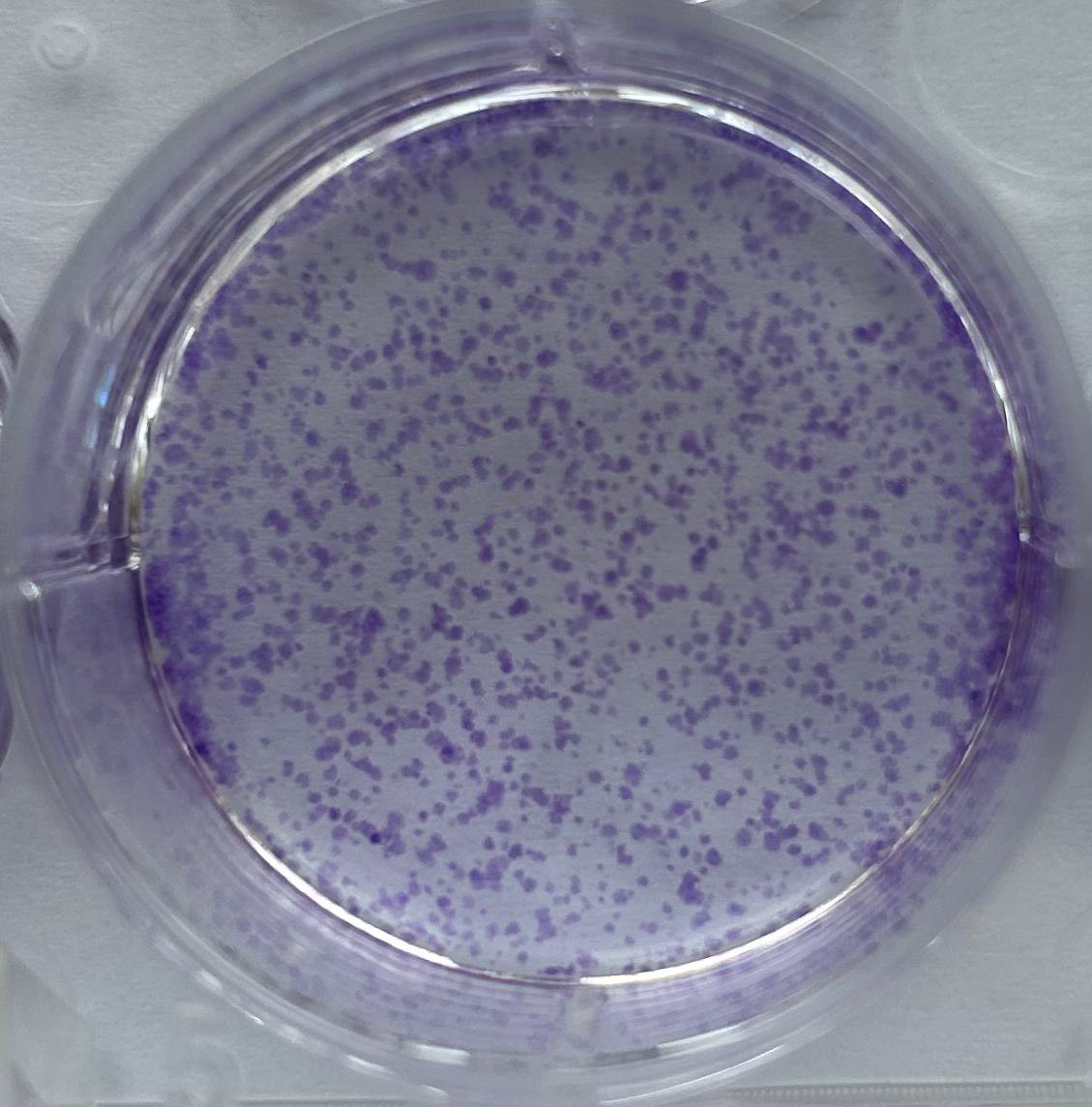

Supplement: Supplemental Information 13 [file peerj-12-18497-s013.zip › hucct1 functional experiment/NC knockdown (NC SI)/hucct nc si clone formation/picture/si185-2.jpg]

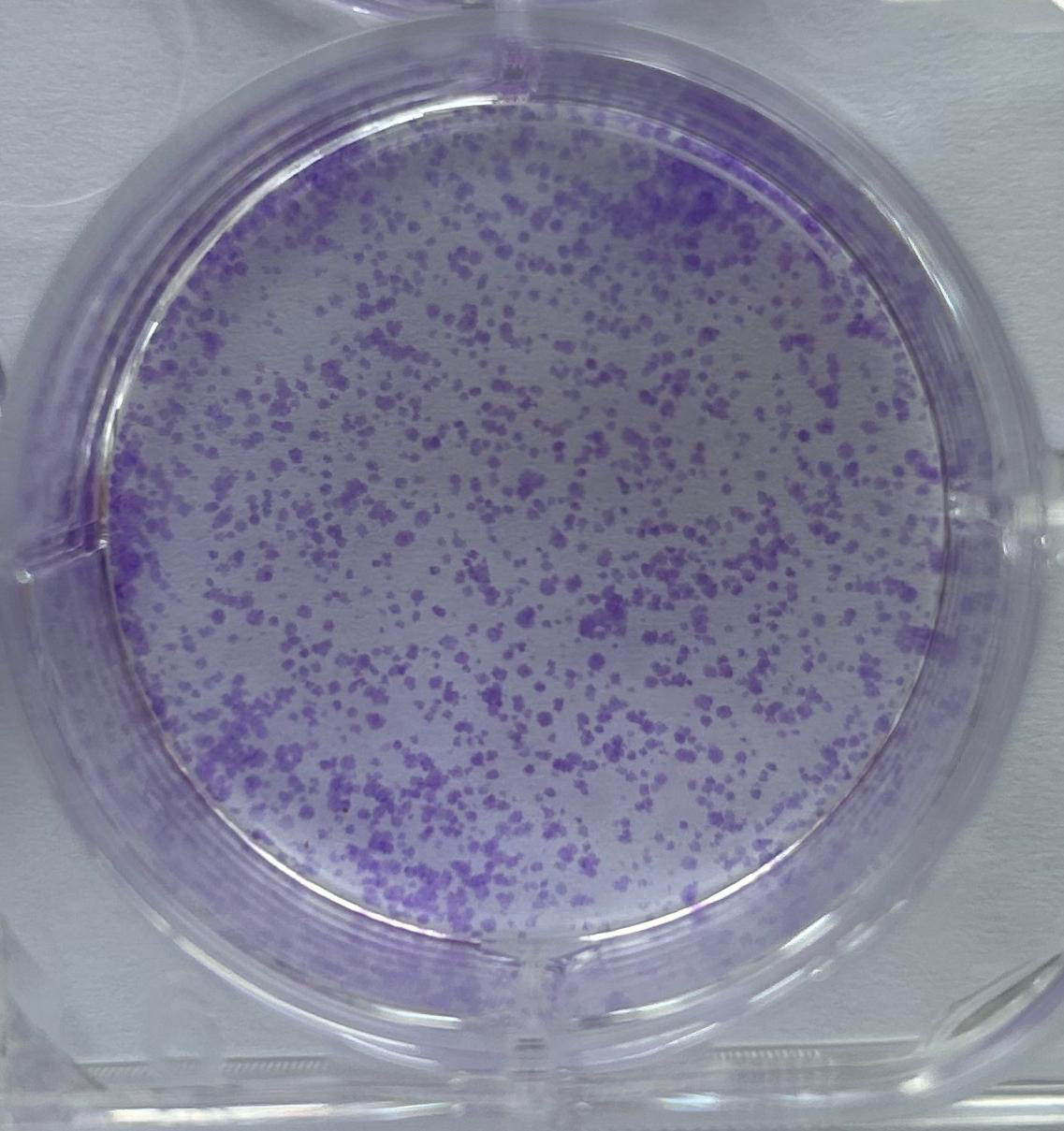

Supplement: Supplemental Information 13 [file peerj-12-18497-s013.zip › hucct1 functional experiment/NC knockdown (NC SI)/hucct nc si clone formation/picture/si185-3.jpg]

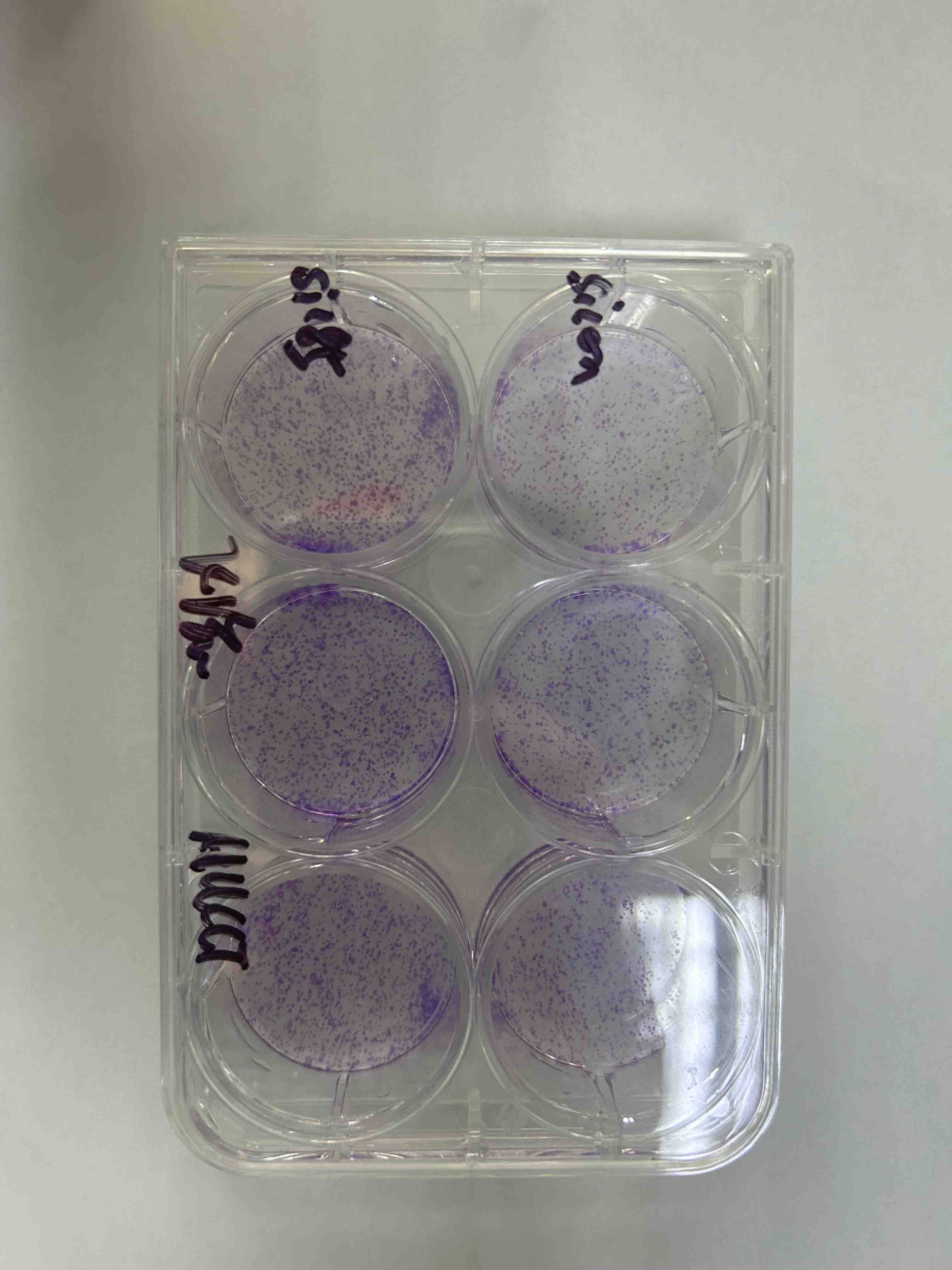

Supplement: Supplemental Information 13 [file peerj-12-18497-s013.zip › hucct1 functional experiment/NC knockdown (NC SI)/hucct nc si clone formation/picture/sicon si185.jpg]

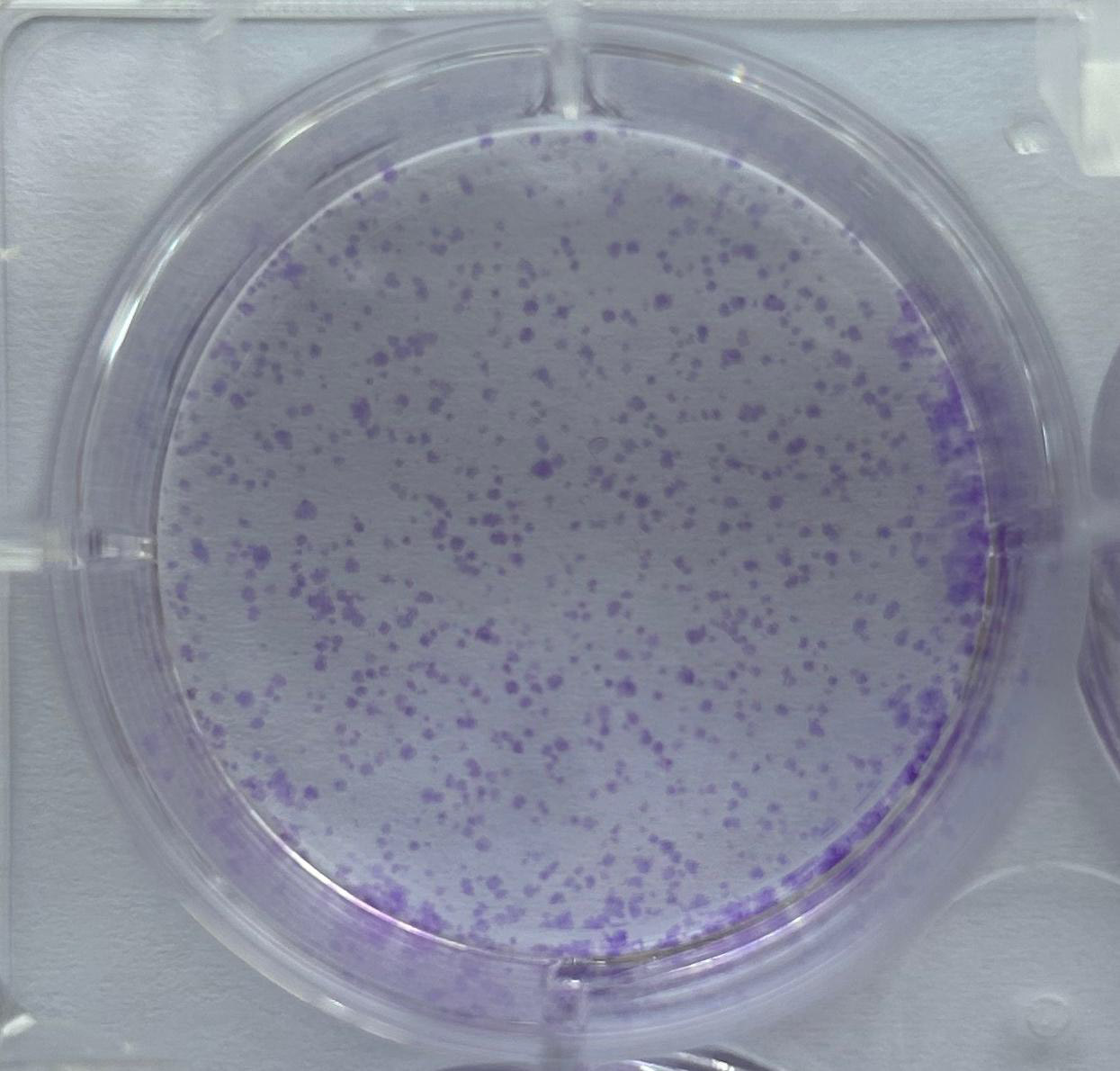

Supplement: Supplemental Information 13 [file peerj-12-18497-s013.zip › hucct1 functional experiment/NC knockdown (NC SI)/hucct nc si clone formation/picture/sicon-1.jpg]

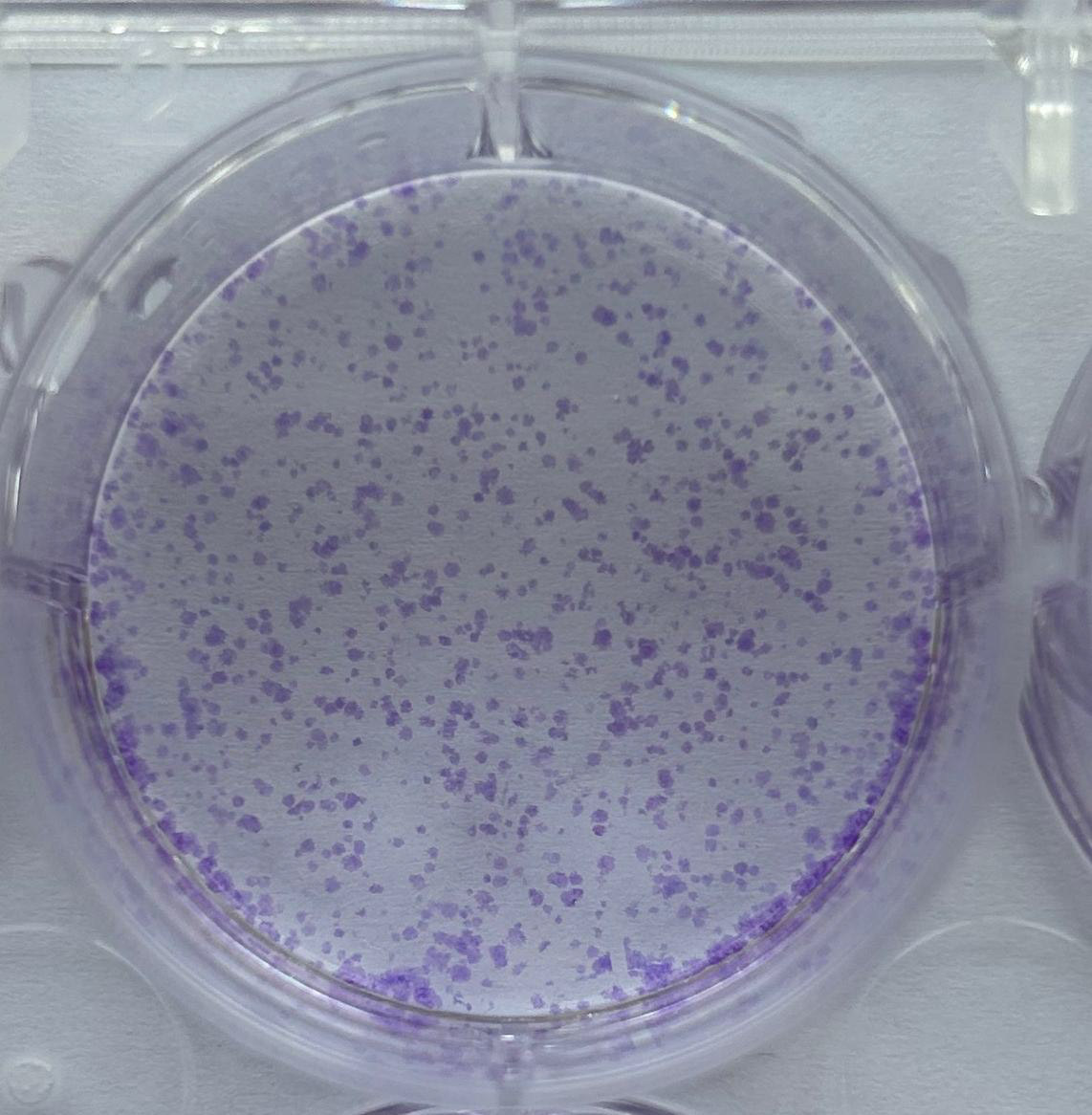

Supplement: Supplemental Information 13 [file peerj-12-18497-s013.zip › hucct1 functional experiment/NC knockdown (NC SI)/hucct nc si clone formation/picture/sicon-2.jpg]

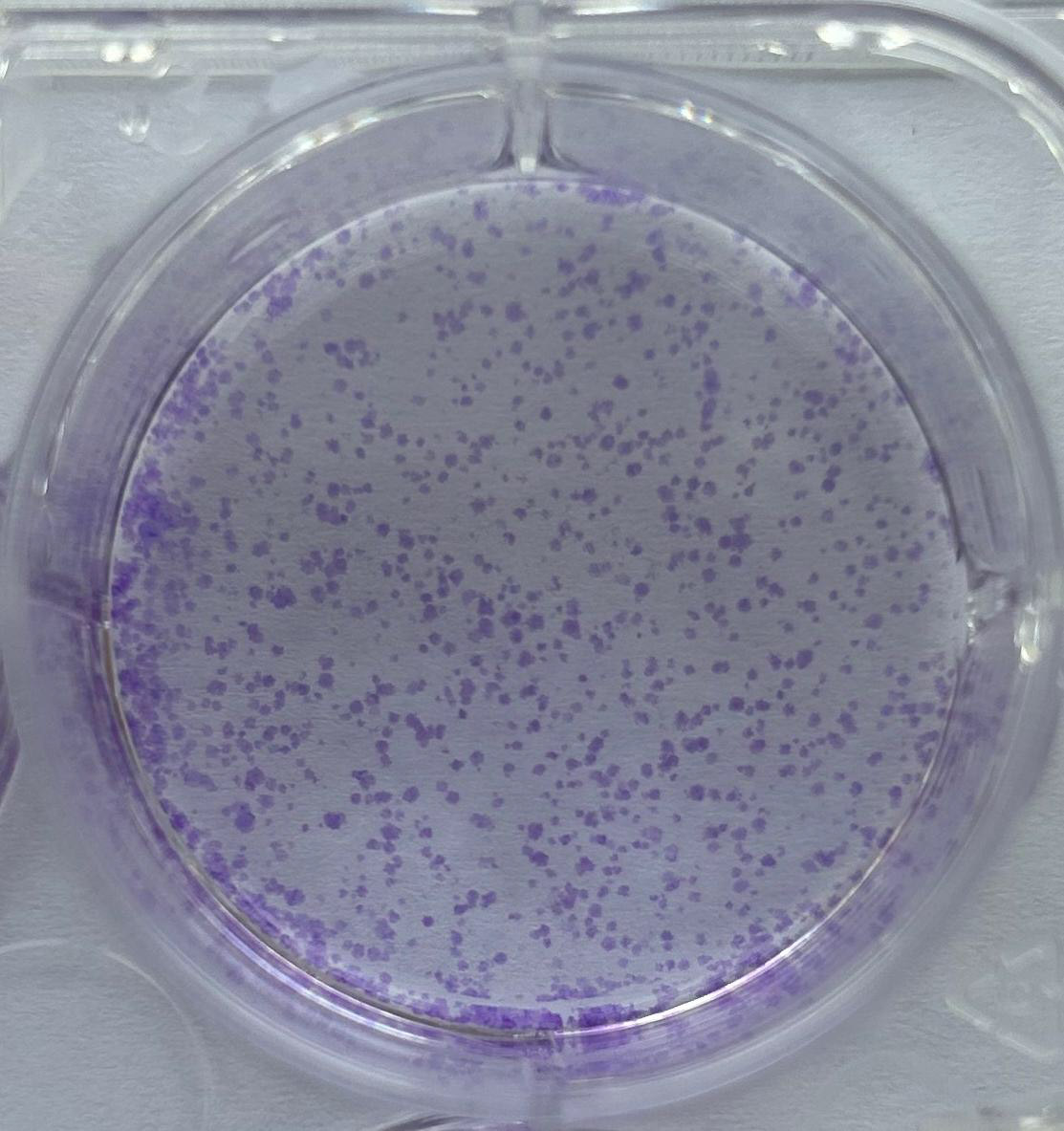

Supplement: Supplemental Information 13 [file peerj-12-18497-s013.zip › hucct1 functional experiment/NC knockdown (NC SI)/hucct nc si clone formation/picture/sicon-3.jpg]

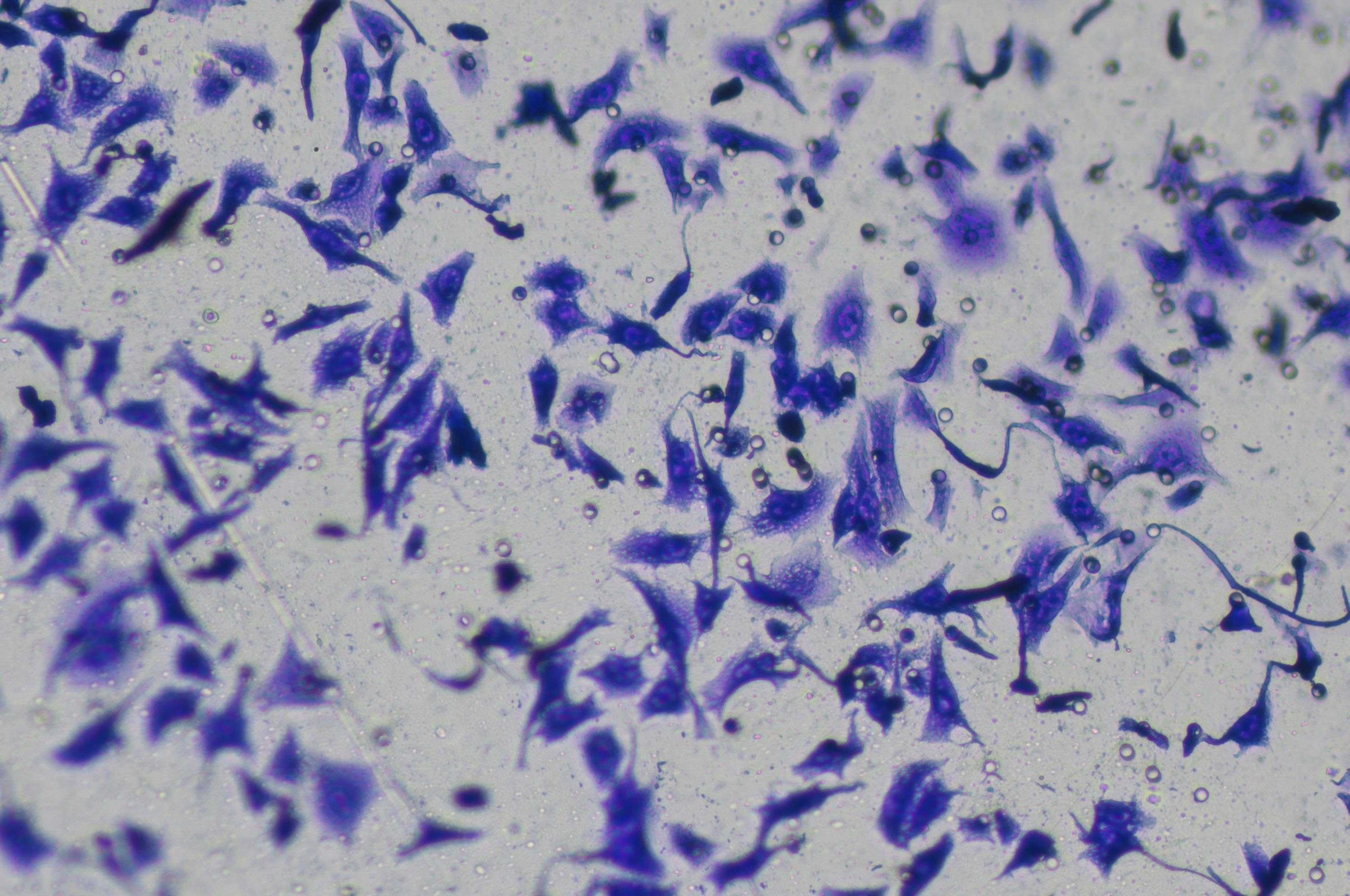

Supplement: Supplemental Information 13 [file peerj-12-18497-s013.zip › hucct1 functional experiment/NC knockdown (NC SI)/hucct1 nc si Invasion/picture/picture/hucct si185 第1孔 第1张.jpg]

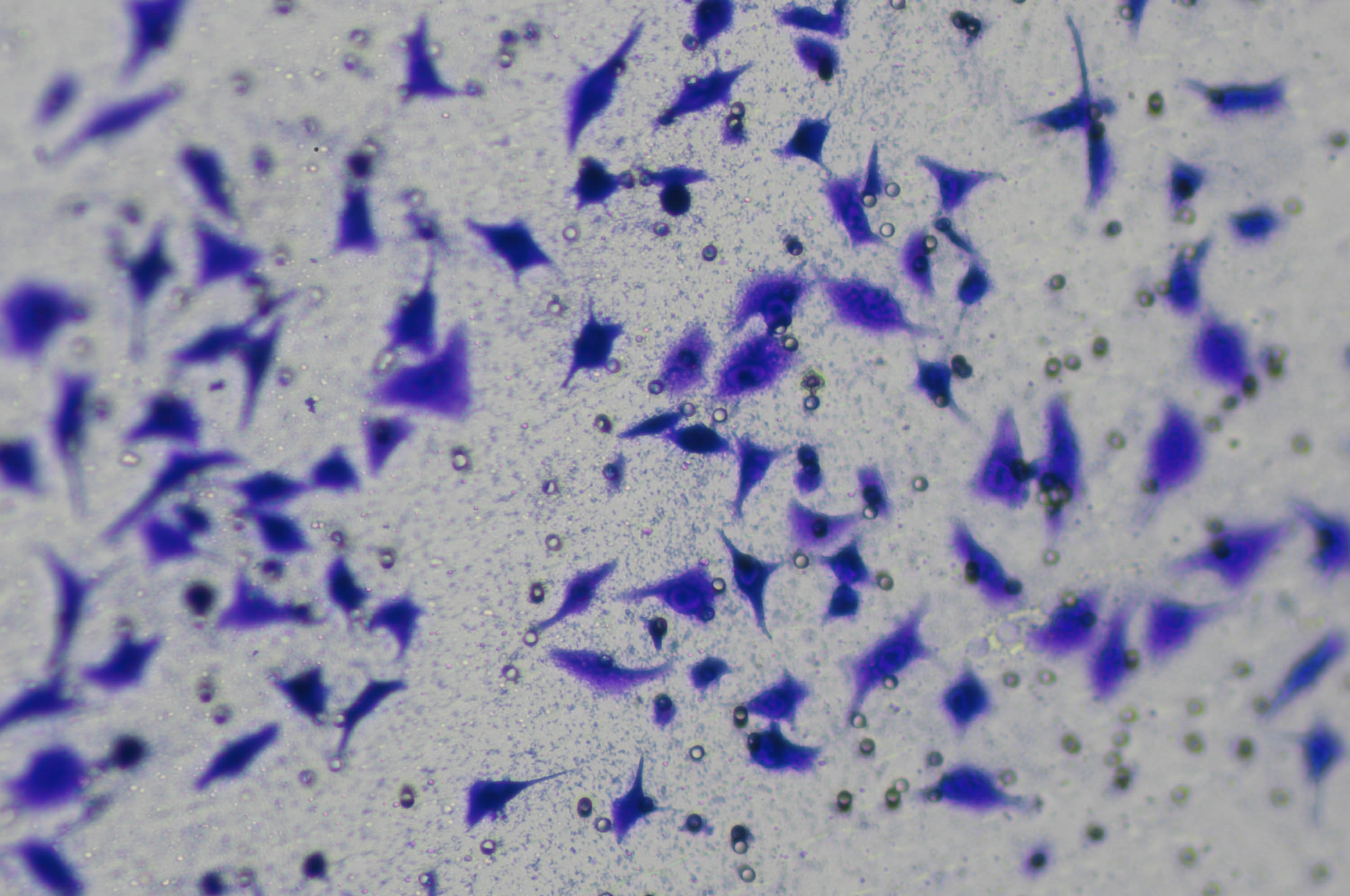

Supplement: Supplemental Information 13 [file peerj-12-18497-s013.zip › hucct1 functional experiment/NC knockdown (NC SI)/hucct1 nc si Invasion/picture/picture/hucct si185 第2孔 第1张.jpg]

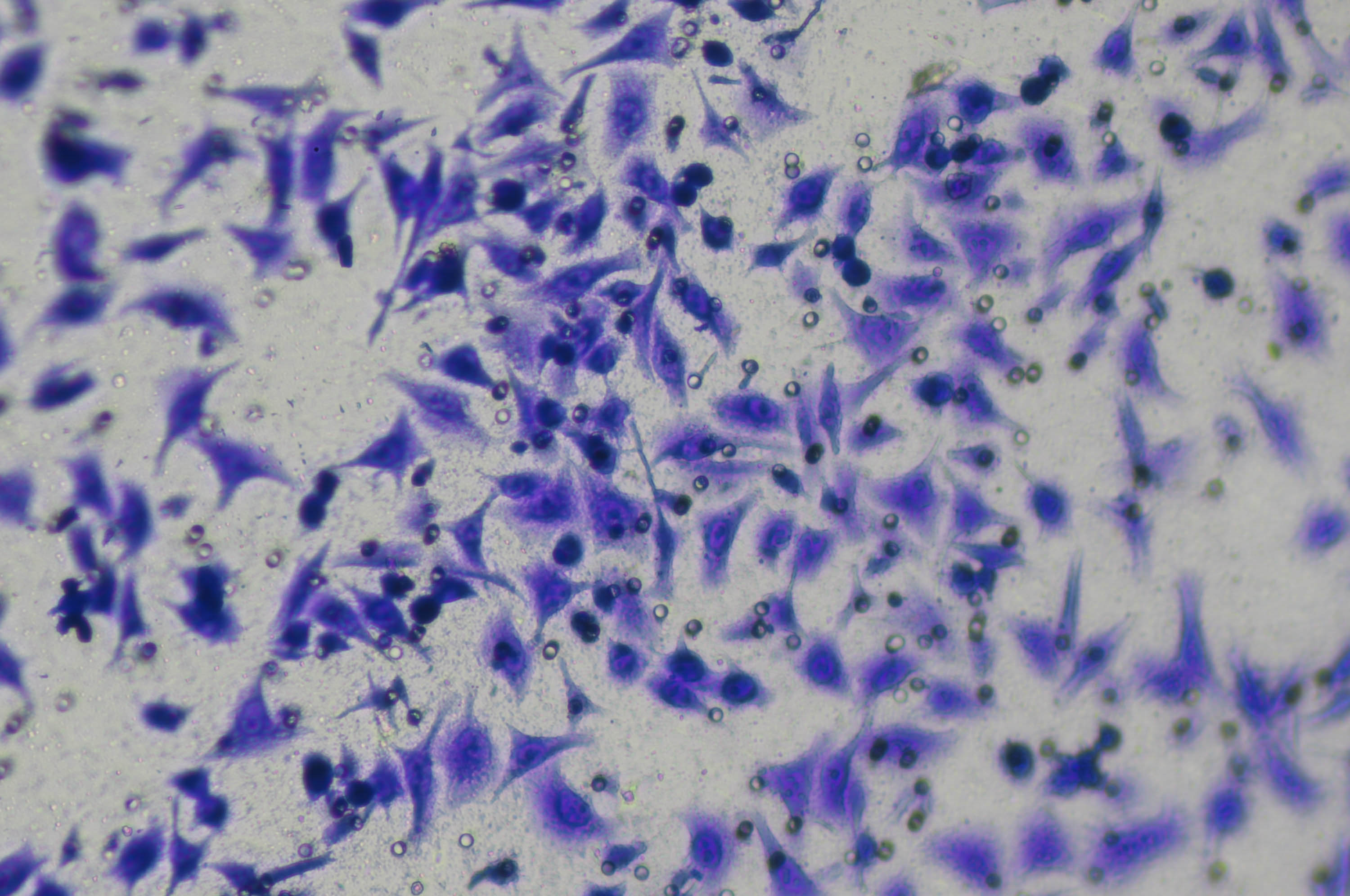

Supplement: Supplemental Information 13 [file peerj-12-18497-s013.zip › hucct1 functional experiment/NC knockdown (NC SI)/hucct1 nc si Invasion/picture/picture/hucct si185 第3孔 第2张.jpg]

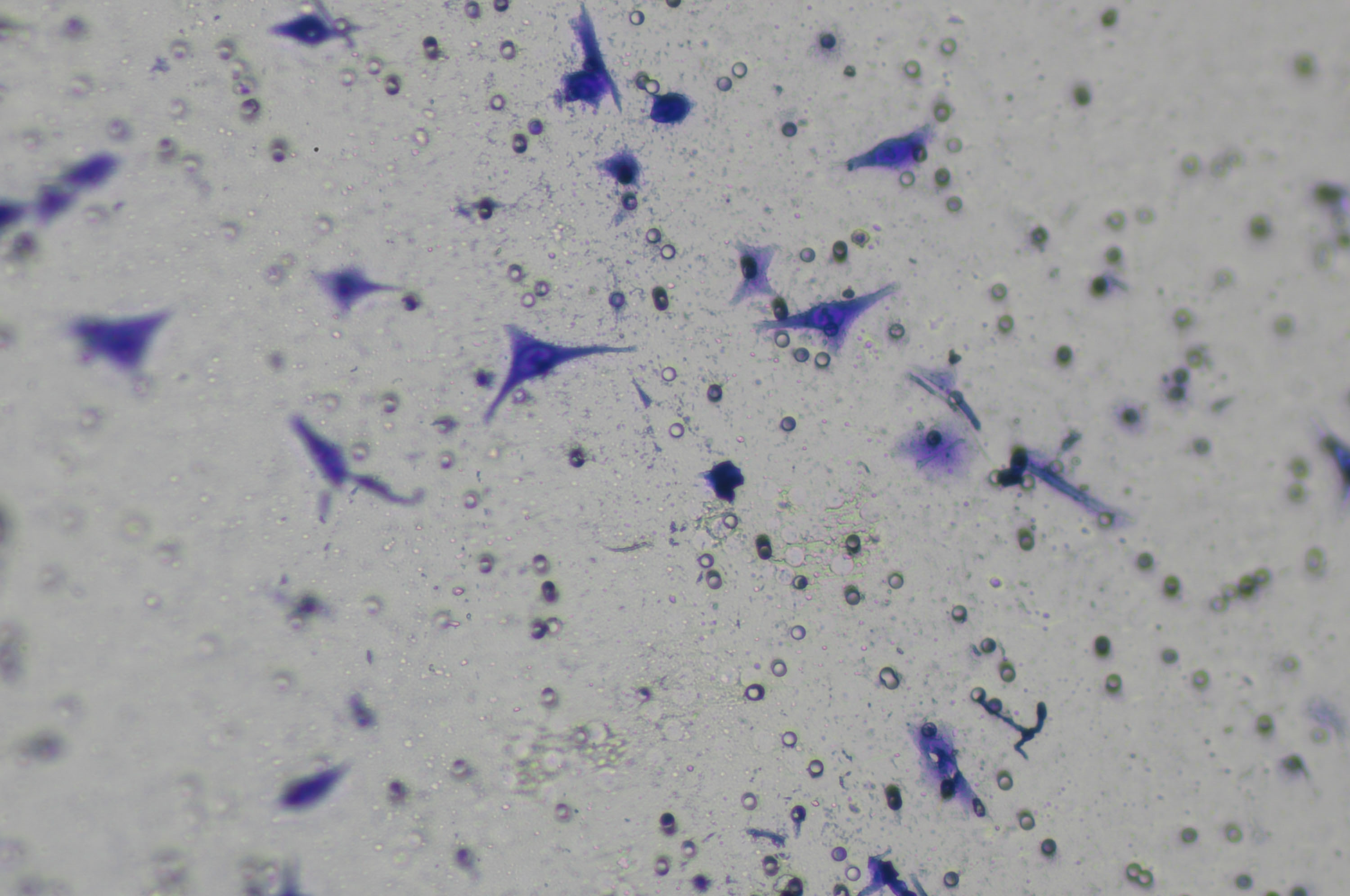

Supplement: Supplemental Information 13 [file peerj-12-18497-s013.zip › hucct1 functional experiment/NC knockdown (NC SI)/hucct1 nc si Invasion/picture/picture/hucct sicon 第1孔 第1张.jpg]

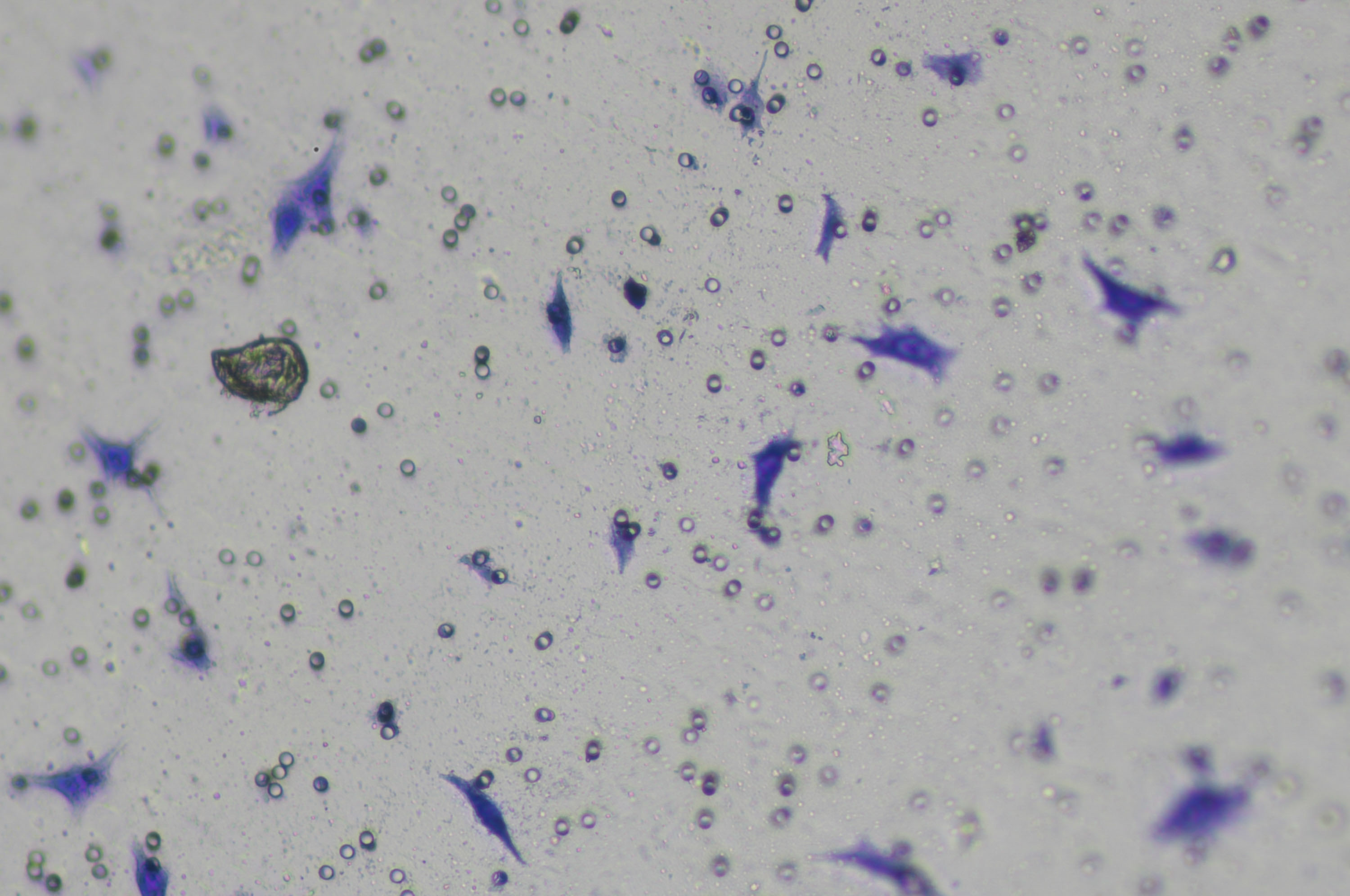

Supplement: Supplemental Information 13 [file peerj-12-18497-s013.zip › hucct1 functional experiment/NC knockdown (NC SI)/hucct1 nc si Invasion/picture/picture/hucct sicon 第2孔 第1张.jpg]

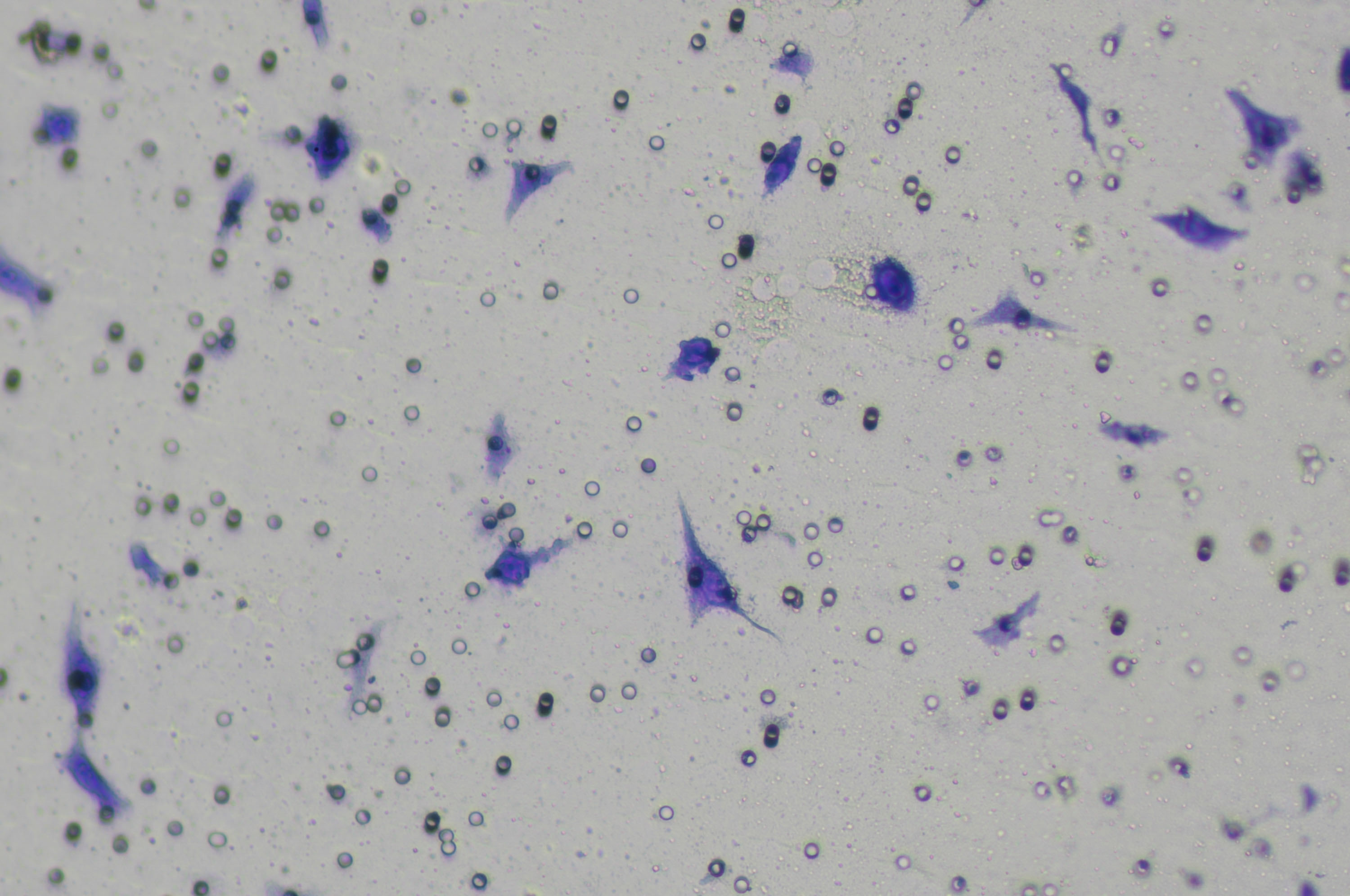

Supplement: Supplemental Information 13 [file peerj-12-18497-s013.zip › hucct1 functional experiment/NC knockdown (NC SI)/hucct1 nc si Invasion/picture/picture/hucct sicon 第3孔 第1张.jpg]
