# Supplementary figures and images for: The CLEC3B inhibits cellular proliferation and metastasis of cholangiocarcinoma through Wnt/β-catenin pathway (part 5 of 5)
Source: PeerJ. 2024 Nov 13;12:e18497. doi: 10.7717/peerj.18497 (PMC11568818; doi:10.7717/peerj.18497)

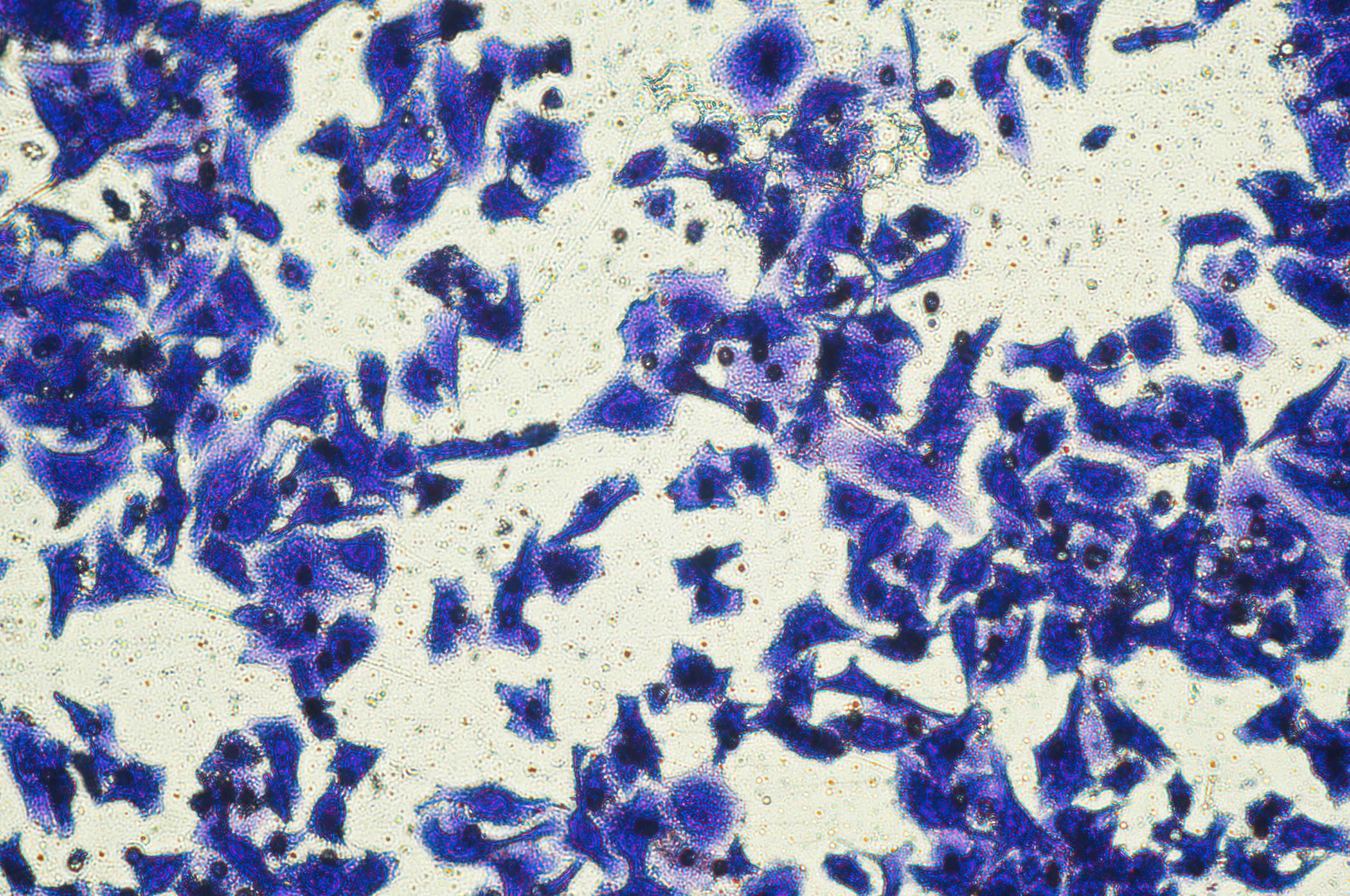

Supplement: Supplemental Information 13 [file peerj-12-18497-s013.zip › hucct1 functional experiment/NC knockdown (NC SI)/hucct1 nc si migration/picture/hucct clec3b si185孔1 20X015.jpg]

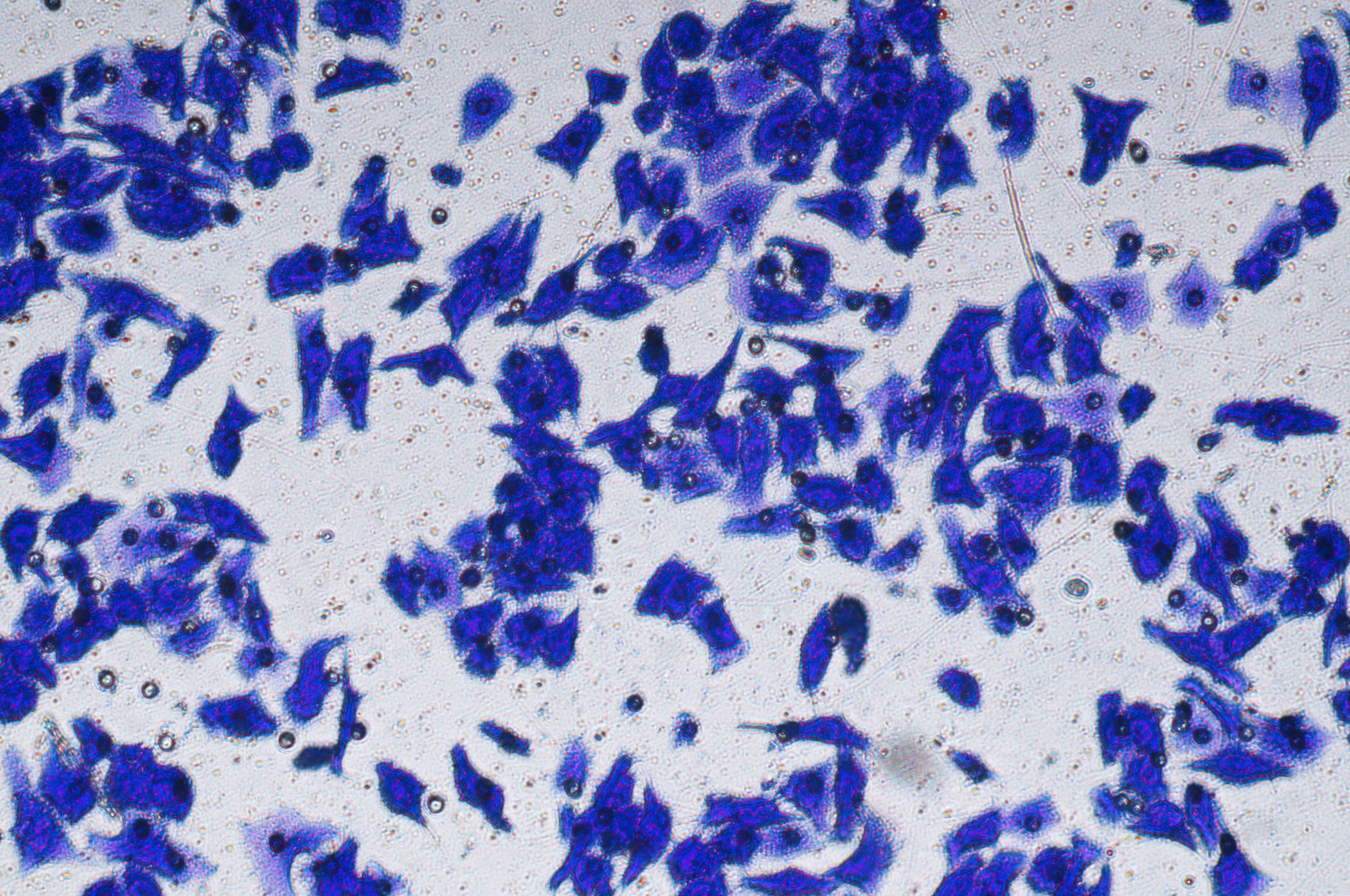

Supplement: Supplemental Information 13 [file peerj-12-18497-s013.zip › hucct1 functional experiment/NC knockdown (NC SI)/hucct1 nc si migration/picture/hucct clec3b si185孔2 20X024.jpg]

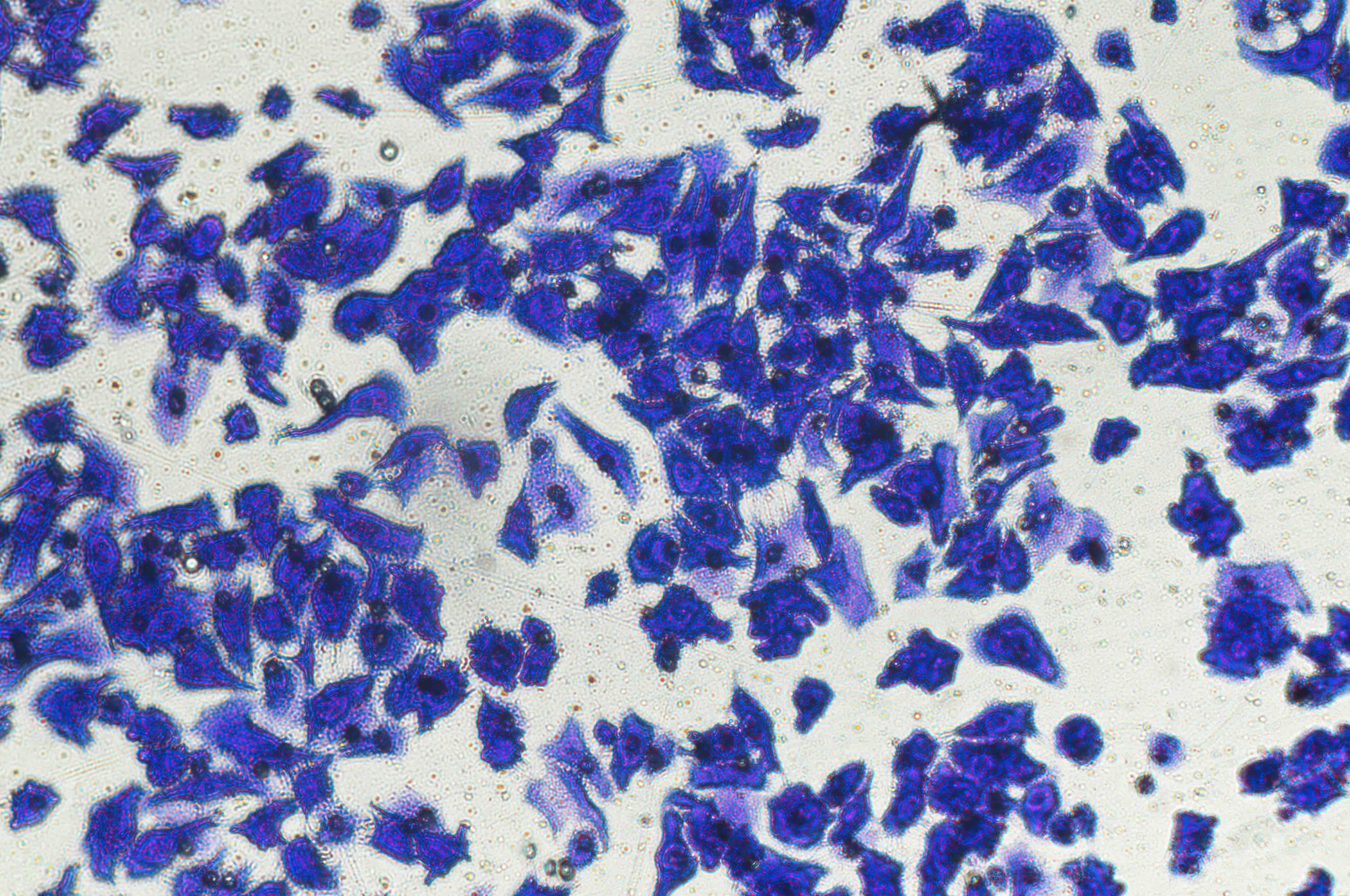

Supplement: Supplemental Information 13 [file peerj-12-18497-s013.zip › hucct1 functional experiment/NC knockdown (NC SI)/hucct1 nc si migration/picture/hucct clec3b si185孔3 20X032.jpg]

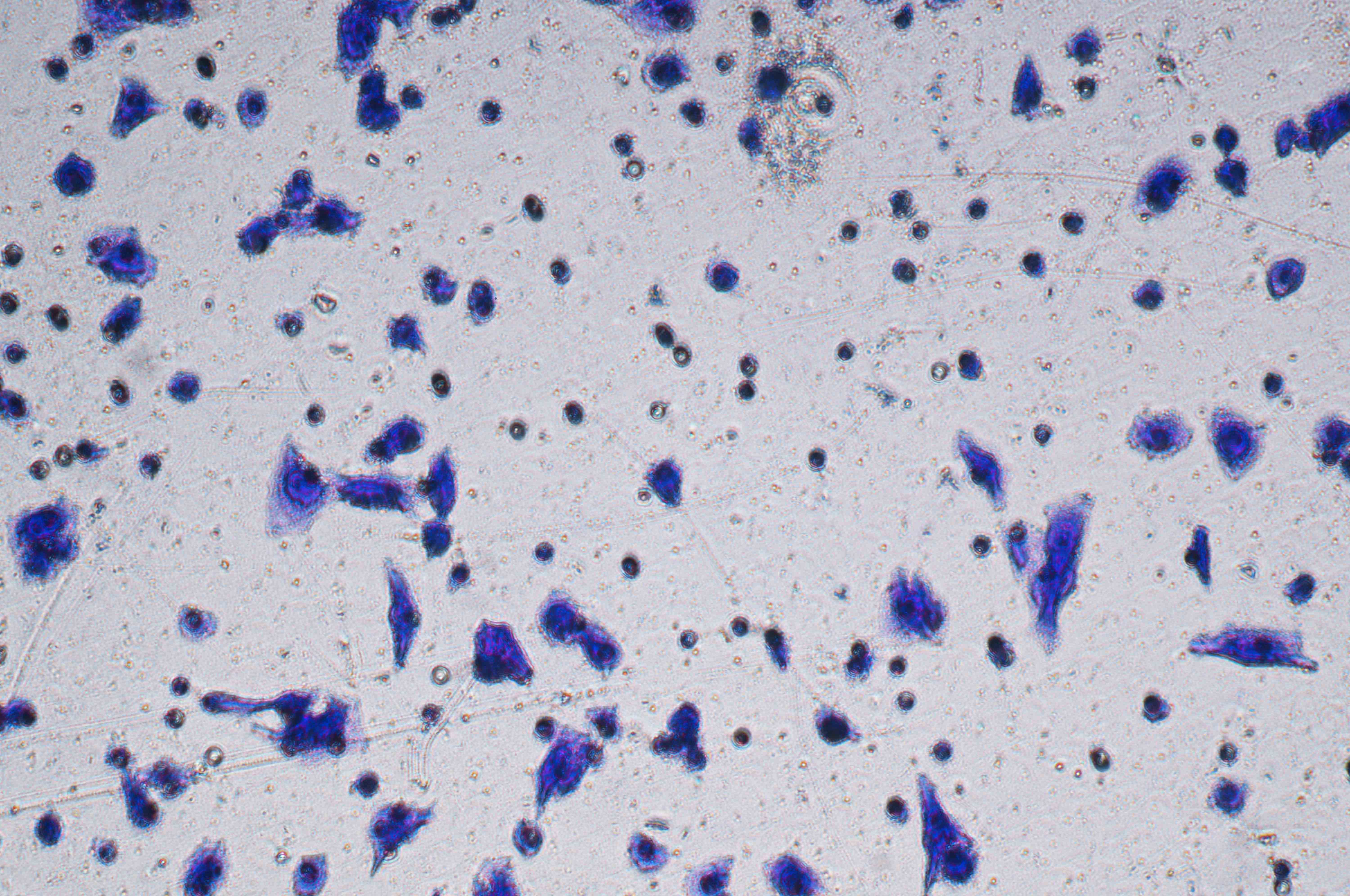

Supplement: Supplemental Information 13 [file peerj-12-18497-s013.zip › hucct1 functional experiment/NC knockdown (NC SI)/hucct1 nc si migration/picture/hucct clec3b sicon孔1 20X003.jpg]

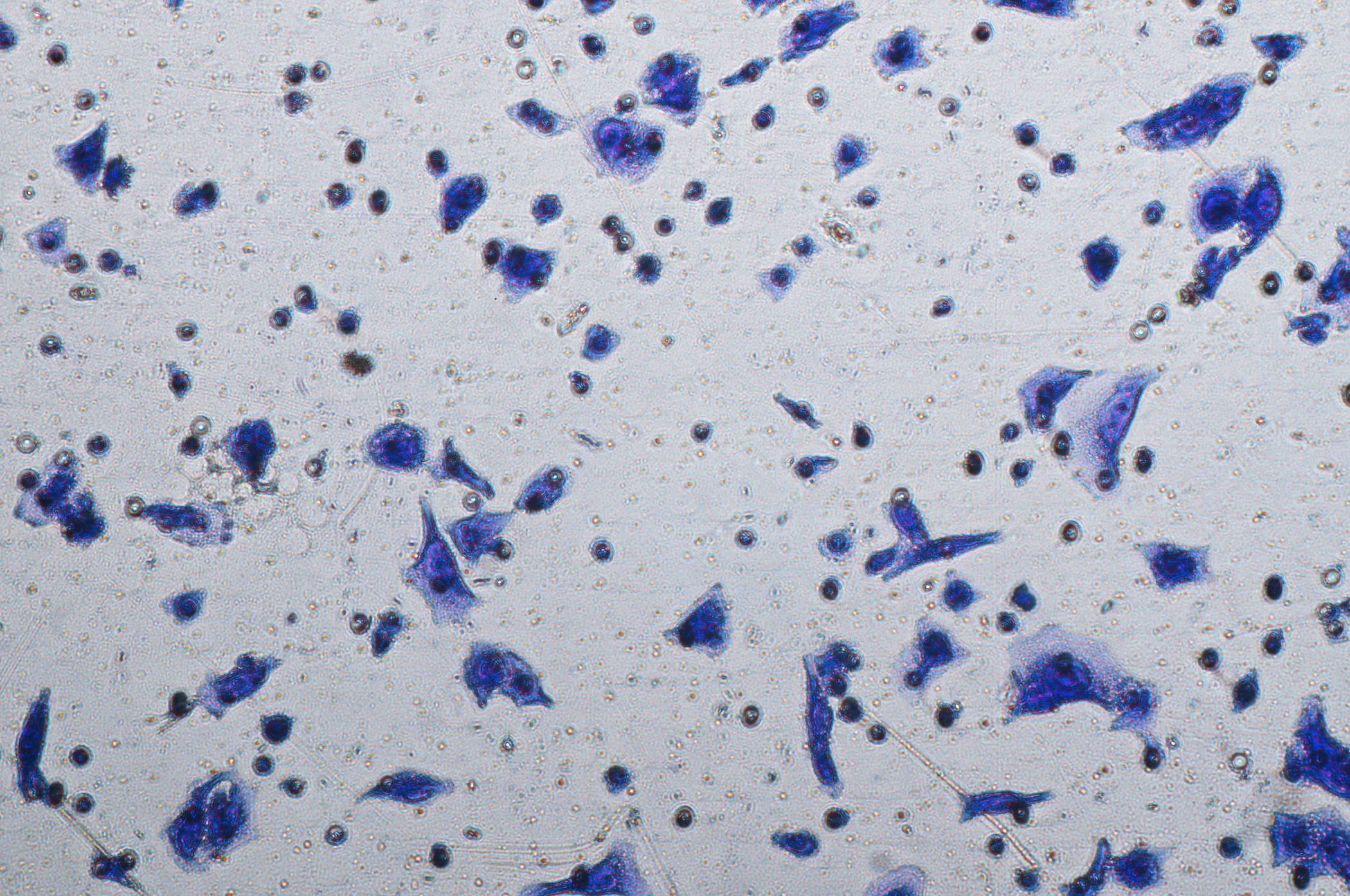

Supplement: Supplemental Information 13 [file peerj-12-18497-s013.zip › hucct1 functional experiment/NC knockdown (NC SI)/hucct1 nc si migration/picture/hucct clec3b sicon孔2 20X007.jpg]

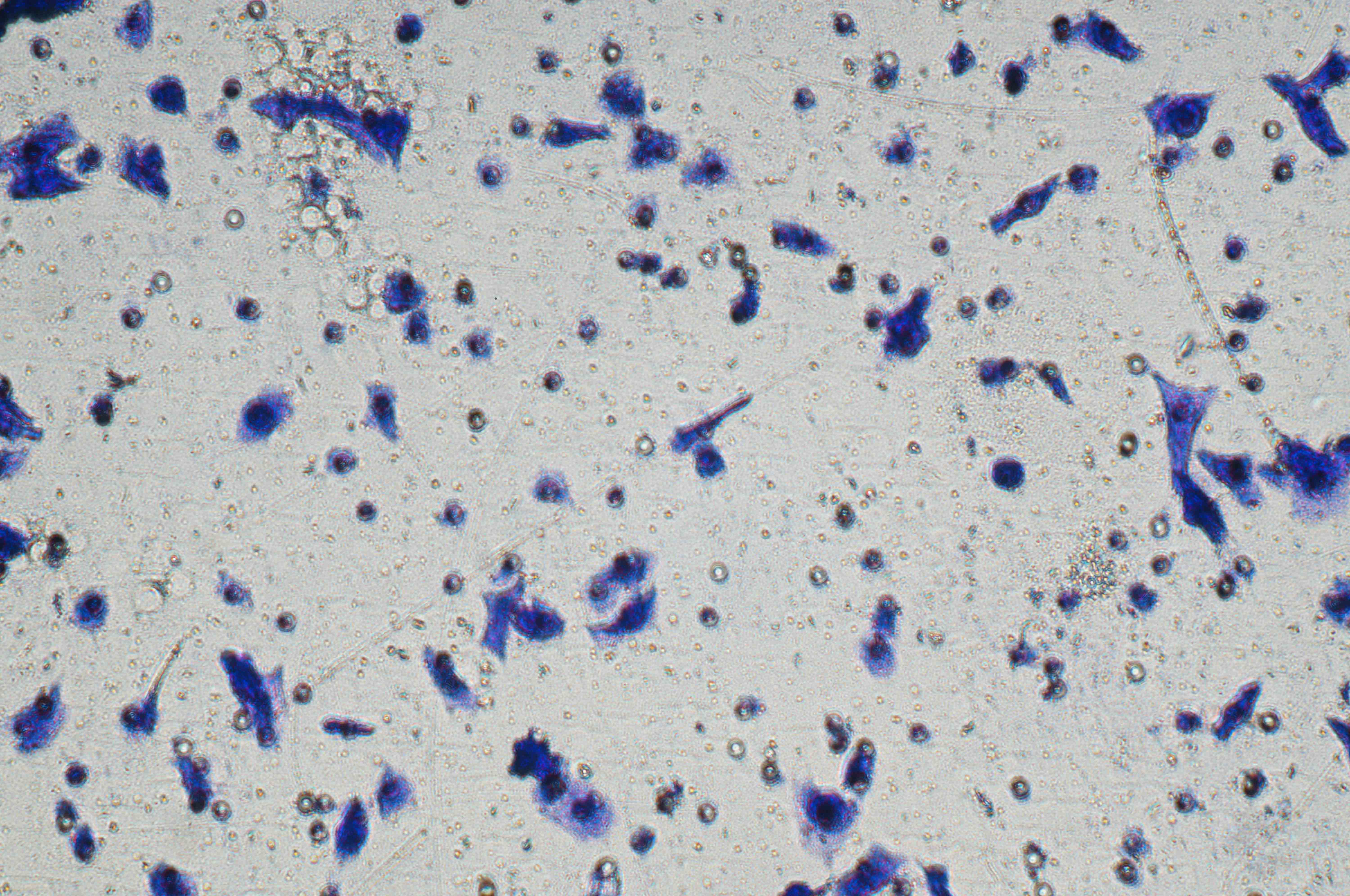

Supplement: Supplemental Information 13 [file peerj-12-18497-s013.zip › hucct1 functional experiment/NC knockdown (NC SI)/hucct1 nc si migration/picture/hucct clec3b sicon孔3 20X013.jpg]

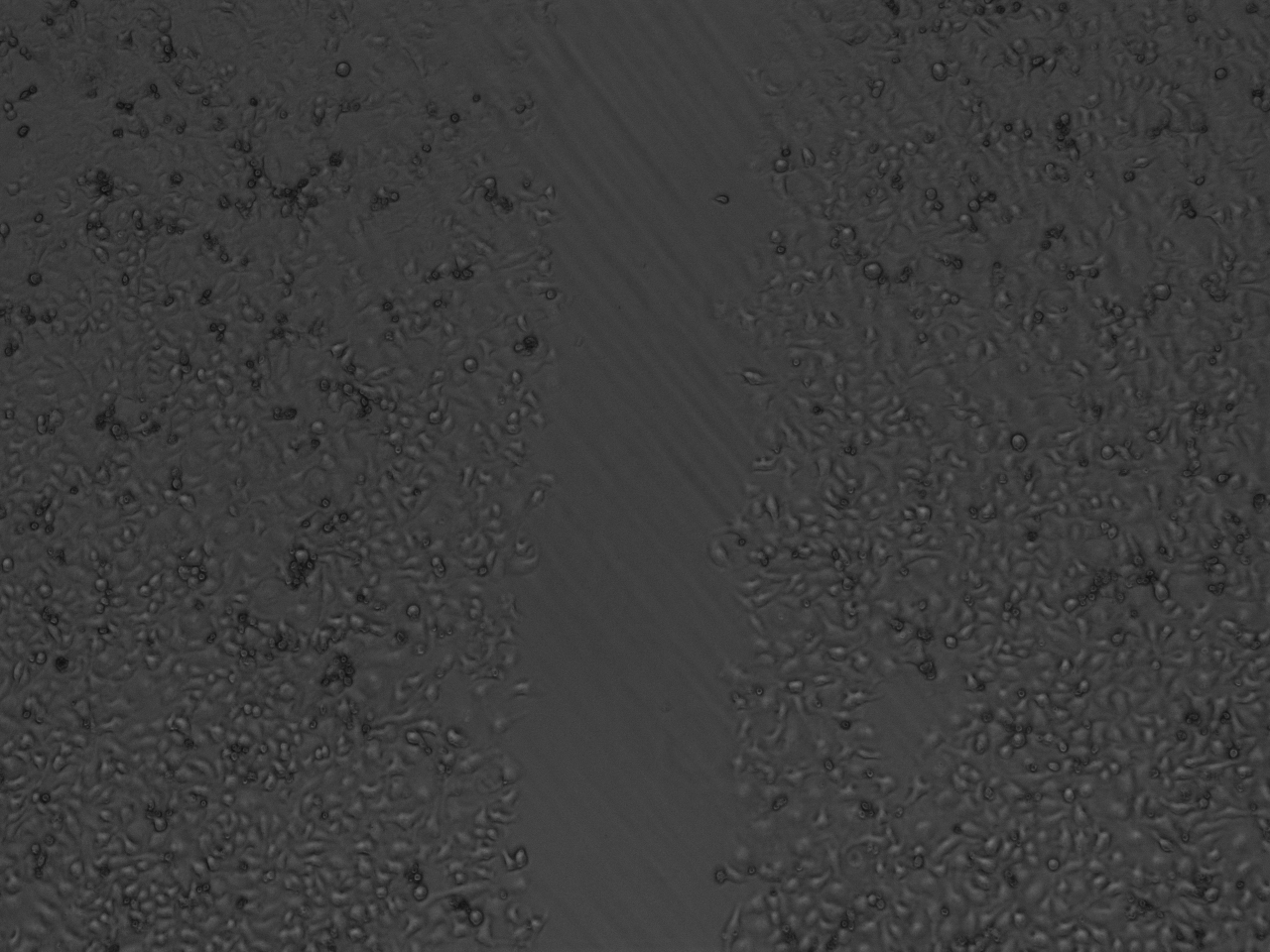

Supplement: Supplemental Information 13 [file peerj-12-18497-s013.zip › hucct1 functional experiment/NC knockdown (NC SI)/hucct1 nc si Wound Healing/picture/si185 k1 i 24h.jpg]

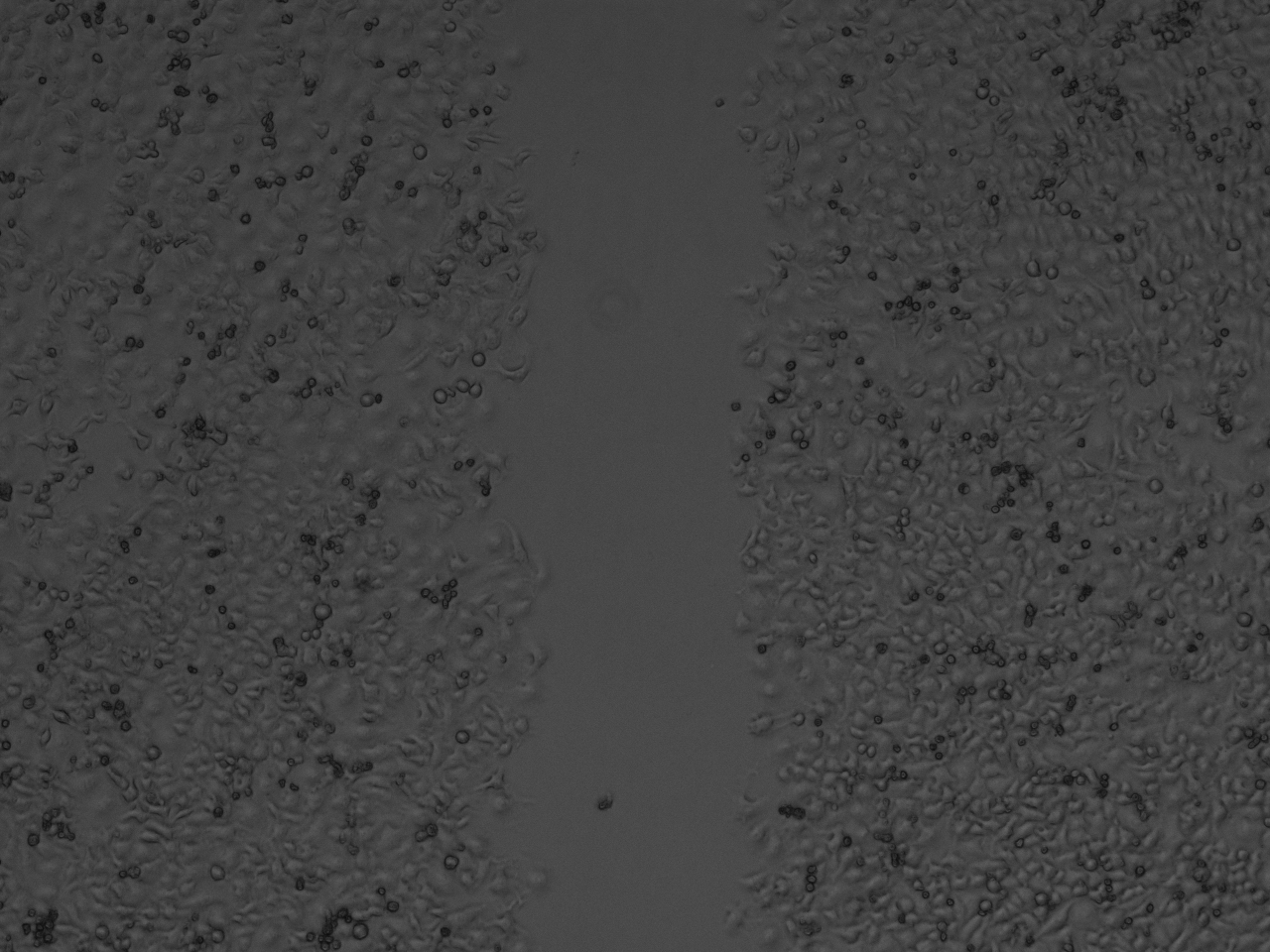

Supplement: Supplemental Information 13 [file peerj-12-18497-s013.zip › hucct1 functional experiment/NC knockdown (NC SI)/hucct1 nc si Wound Healing/picture/si185 k1 j 24h.jpg]

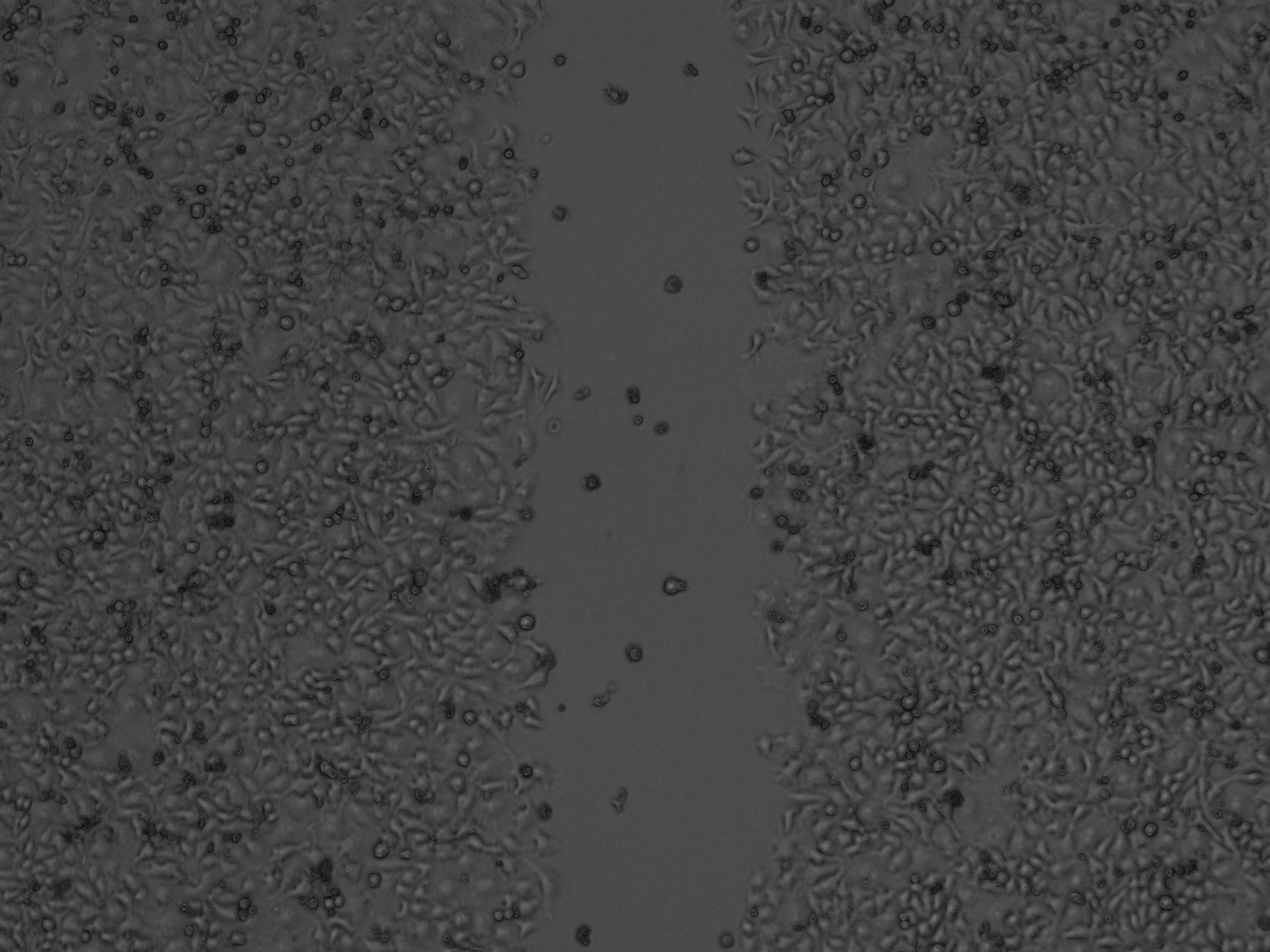

Supplement: Supplemental Information 13 [file peerj-12-18497-s013.zip › hucct1 functional experiment/NC knockdown (NC SI)/hucct1 nc si Wound Healing/picture/si185 k1 k 24h.jpg]

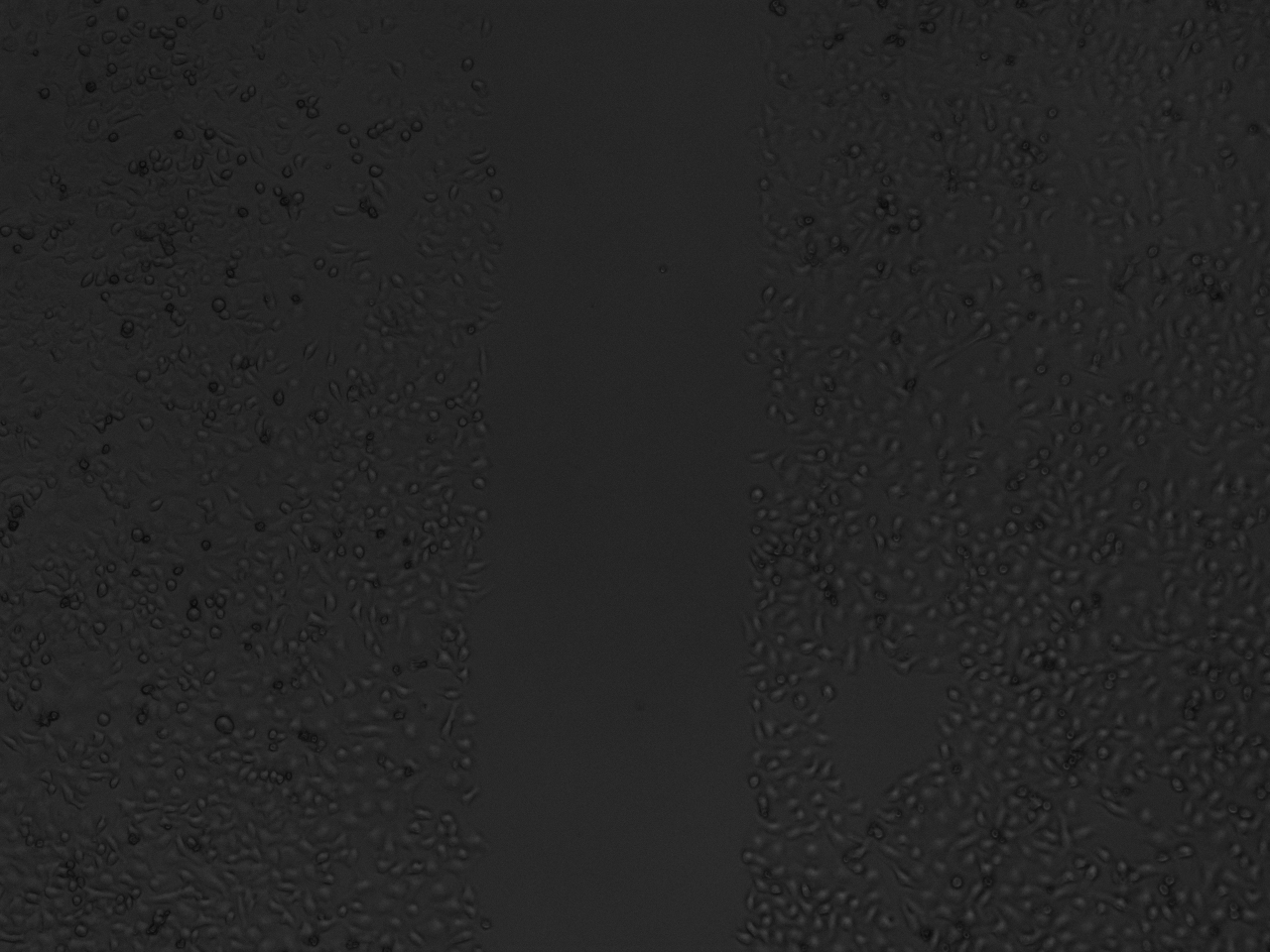

Supplement: Supplemental Information 13 [file peerj-12-18497-s013.zip › hucct1 functional experiment/NC knockdown (NC SI)/hucct1 nc si Wound Healing/picture/si185 k1 i 0h.jpg]

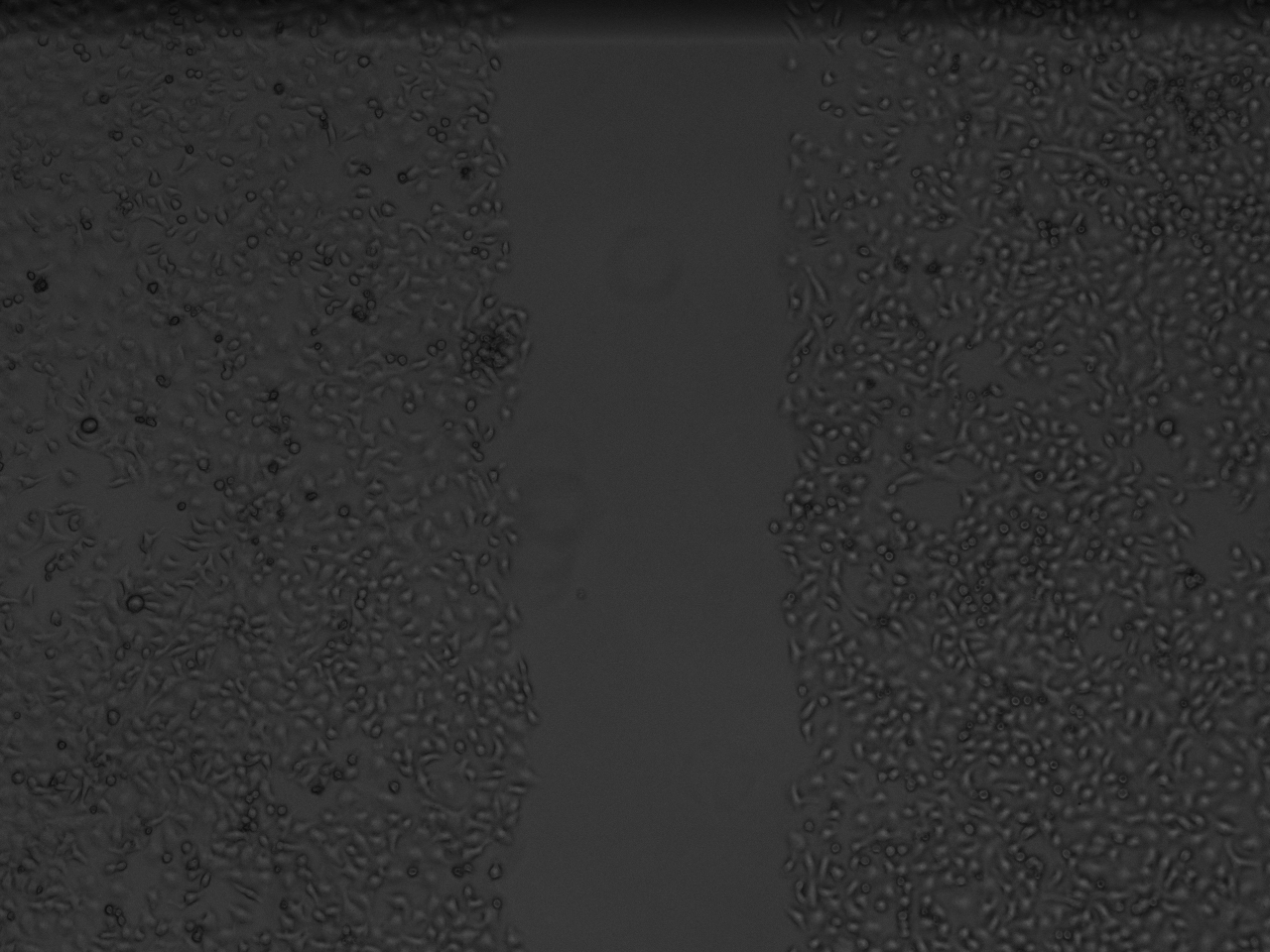

Supplement: Supplemental Information 13 [file peerj-12-18497-s013.zip › hucct1 functional experiment/NC knockdown (NC SI)/hucct1 nc si Wound Healing/picture/si185 k1 j 0h.jpg]

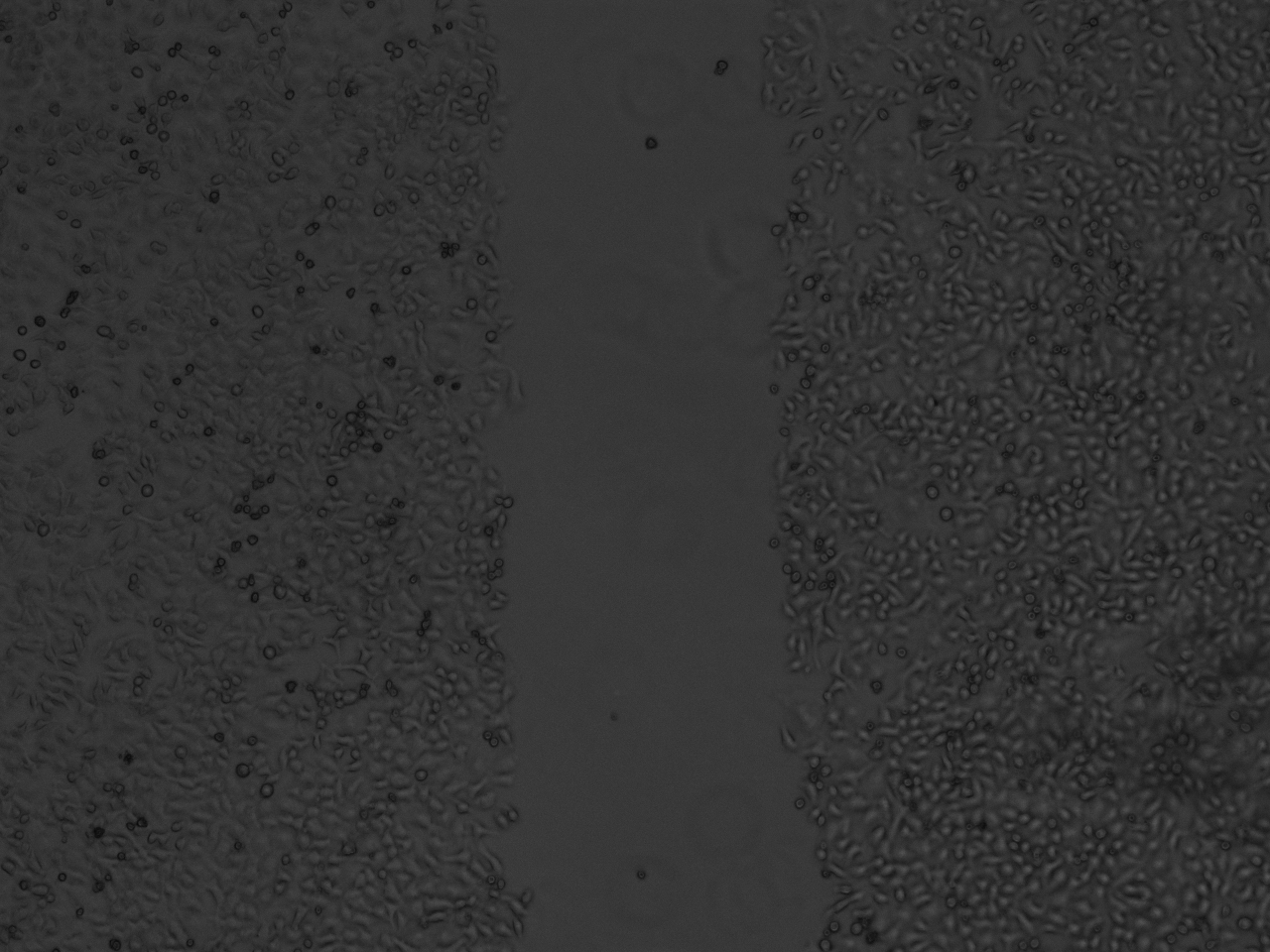

Supplement: Supplemental Information 13 [file peerj-12-18497-s013.zip › hucct1 functional experiment/NC knockdown (NC SI)/hucct1 nc si Wound Healing/picture/si185 k1 k 0h.jpg]

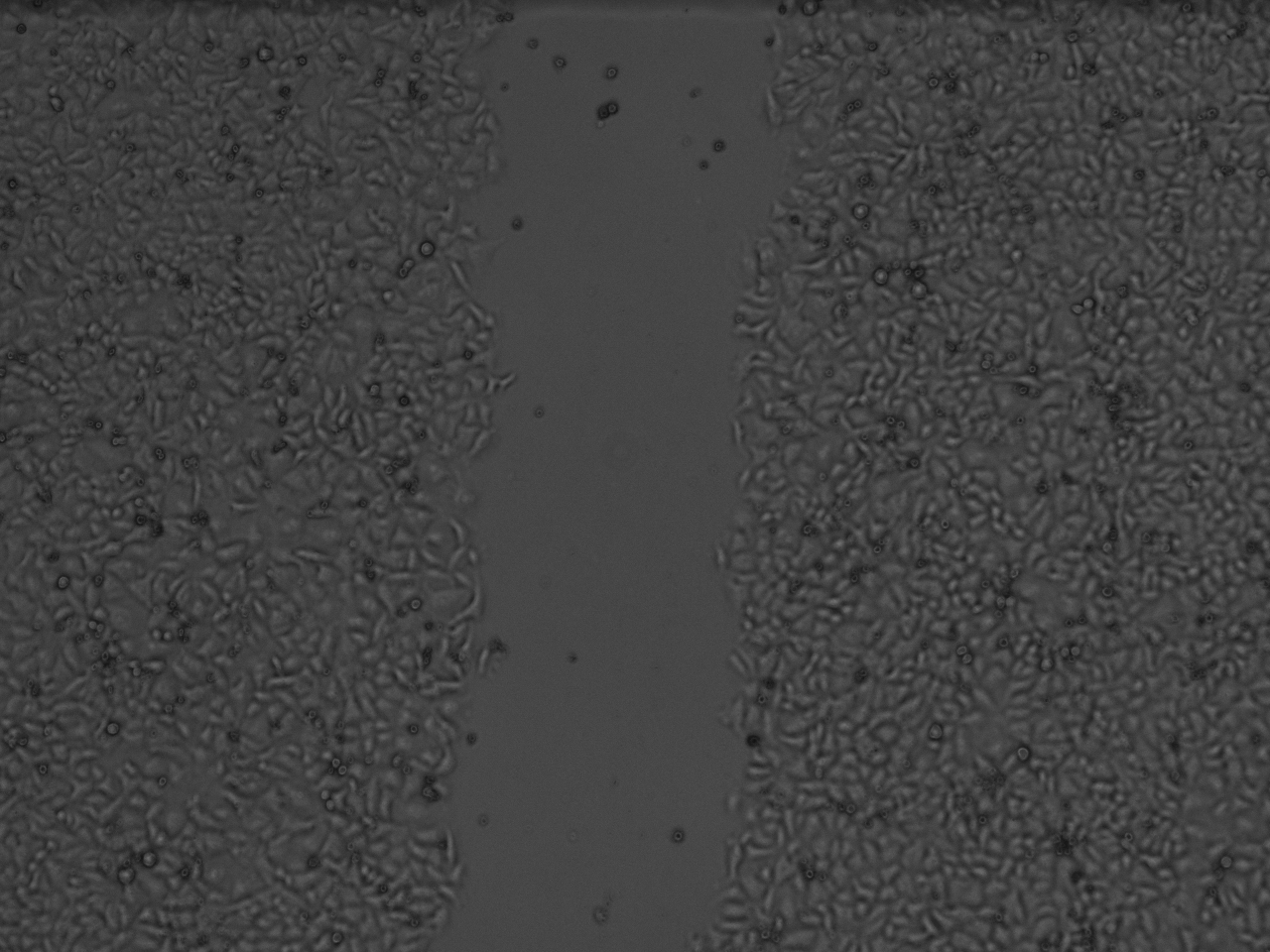

Supplement: Supplemental Information 13 [file peerj-12-18497-s013.zip › hucct1 functional experiment/NC knockdown (NC SI)/hucct1 nc si Wound Healing/picture/sicon k1 f 24h.jpg]

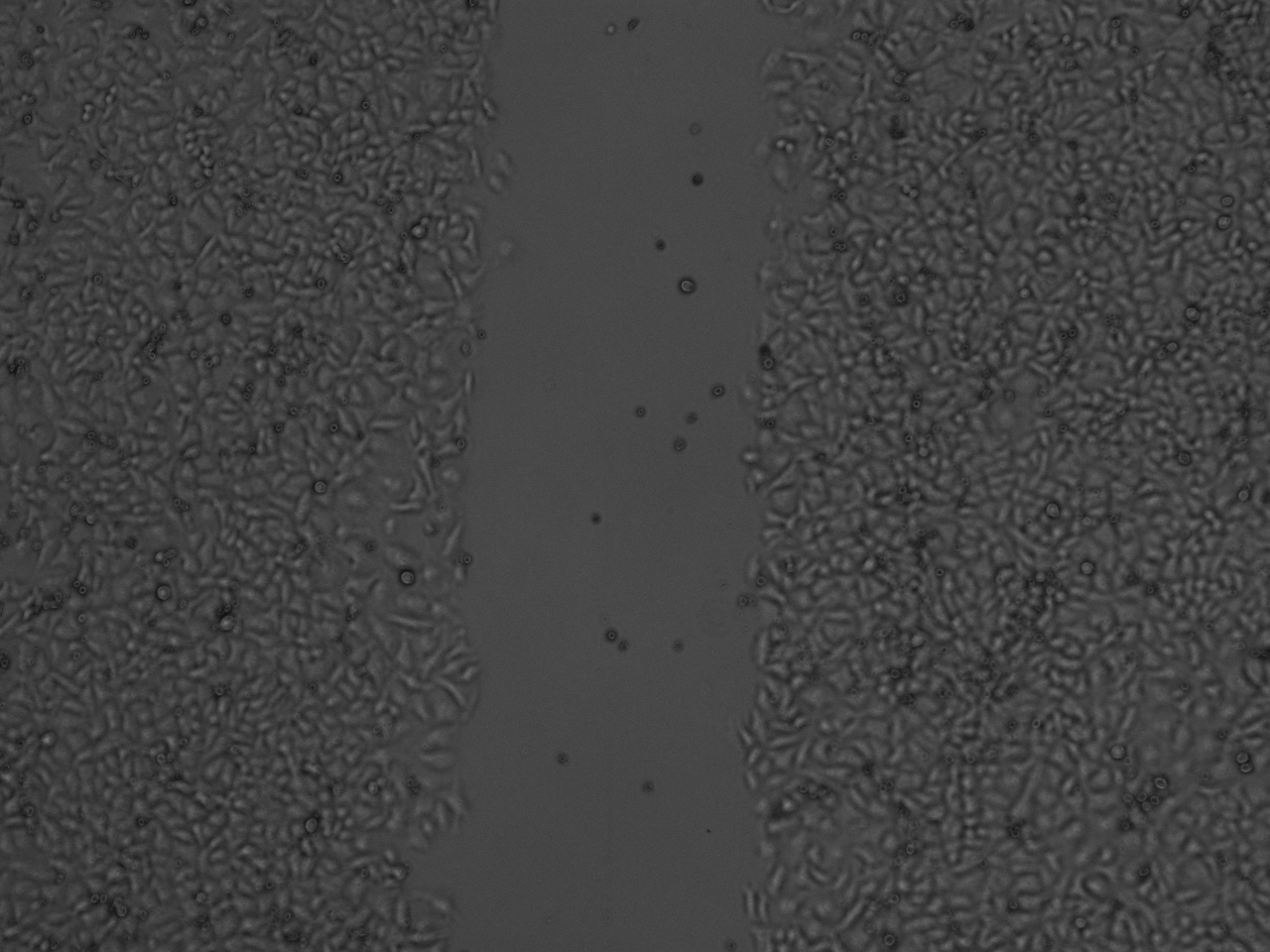

Supplement: Supplemental Information 13 [file peerj-12-18497-s013.zip › hucct1 functional experiment/NC knockdown (NC SI)/hucct1 nc si Wound Healing/picture/sicon k1 g 24h.jpg]

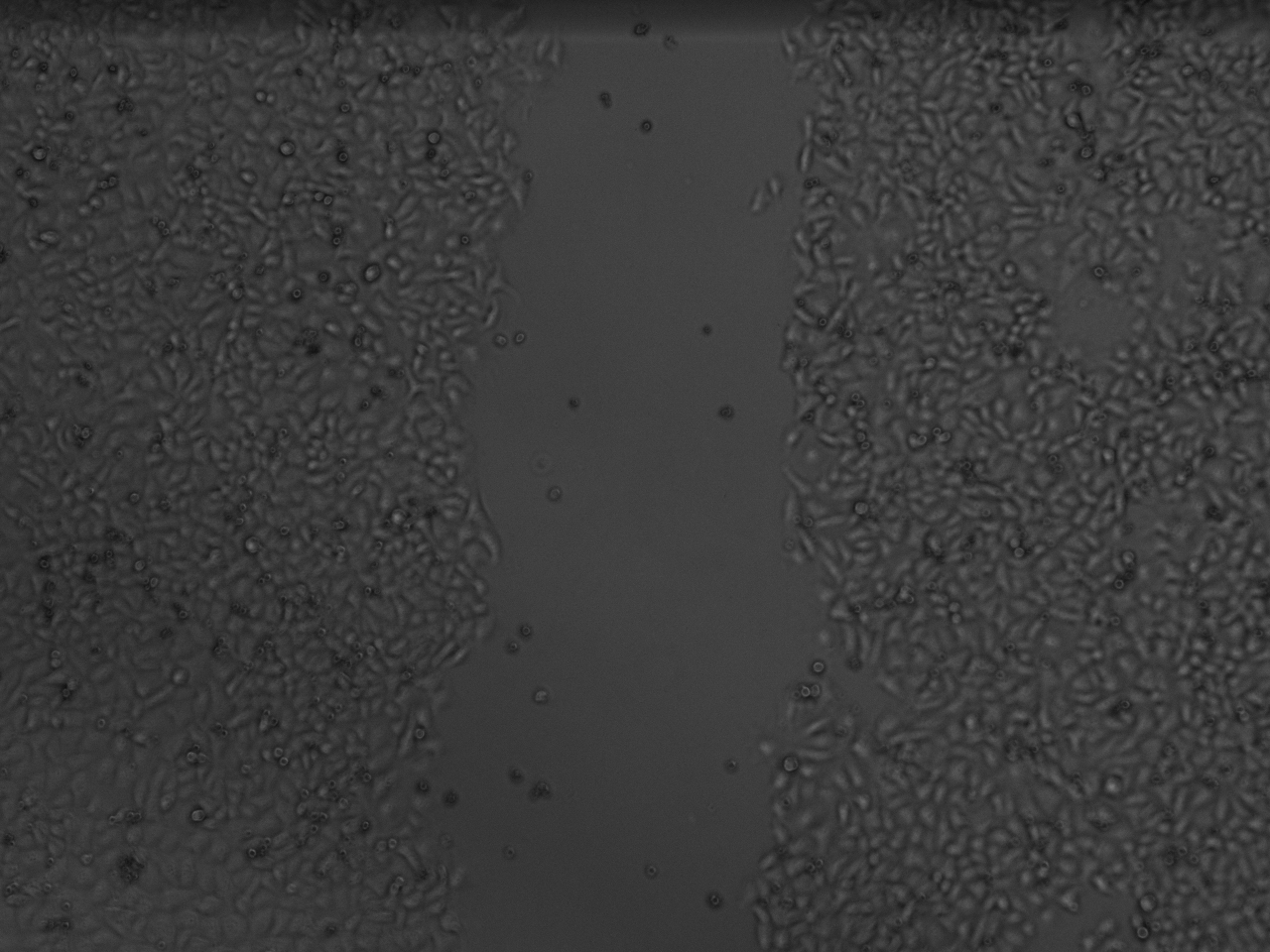

Supplement: Supplemental Information 13 [file peerj-12-18497-s013.zip › hucct1 functional experiment/NC knockdown (NC SI)/hucct1 nc si Wound Healing/picture/sicon k1 h 24h.jpg]

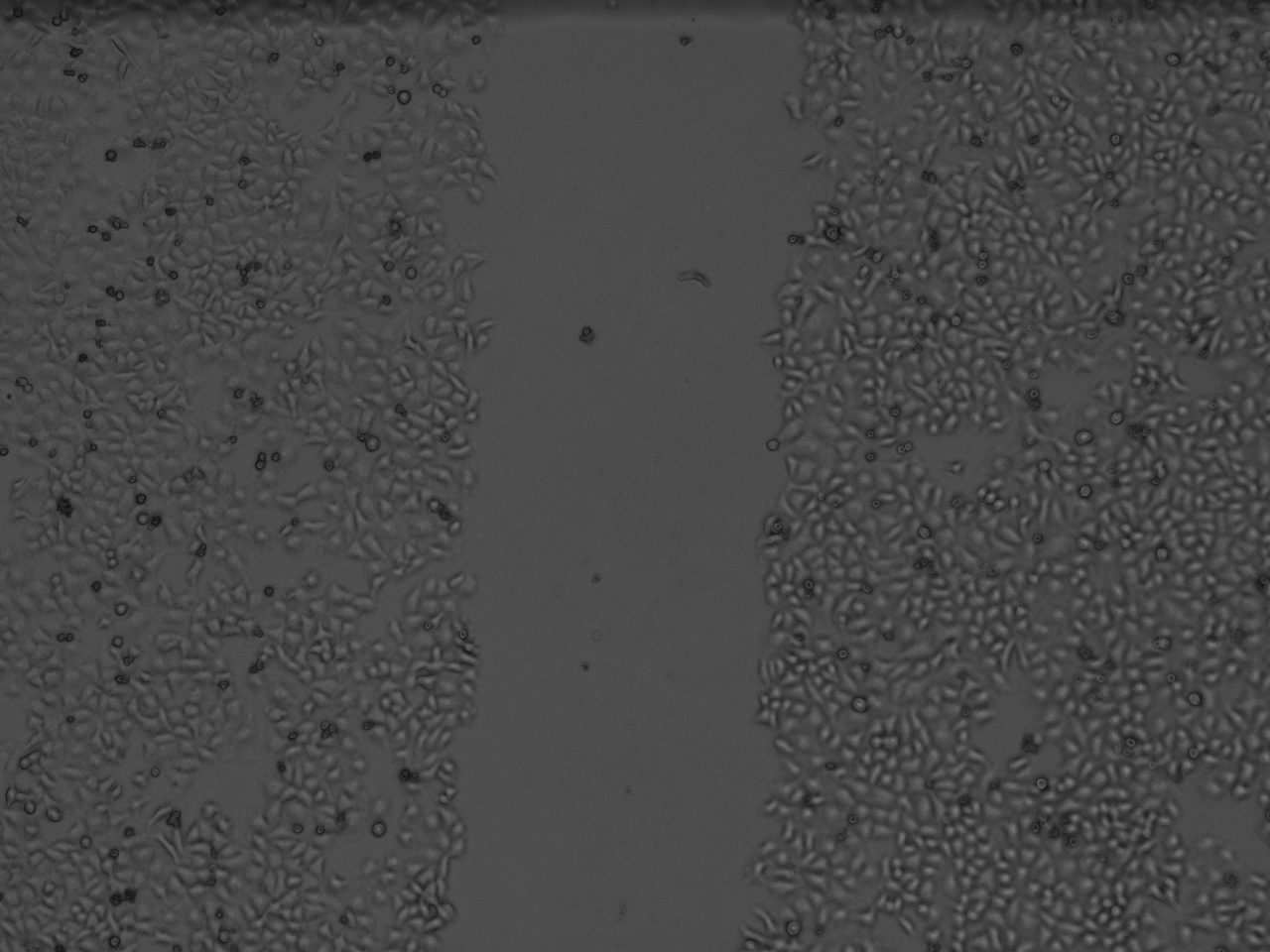

Supplement: Supplemental Information 13 [file peerj-12-18497-s013.zip › hucct1 functional experiment/NC knockdown (NC SI)/hucct1 nc si Wound Healing/picture/sicon k1 f 0h.jpg]

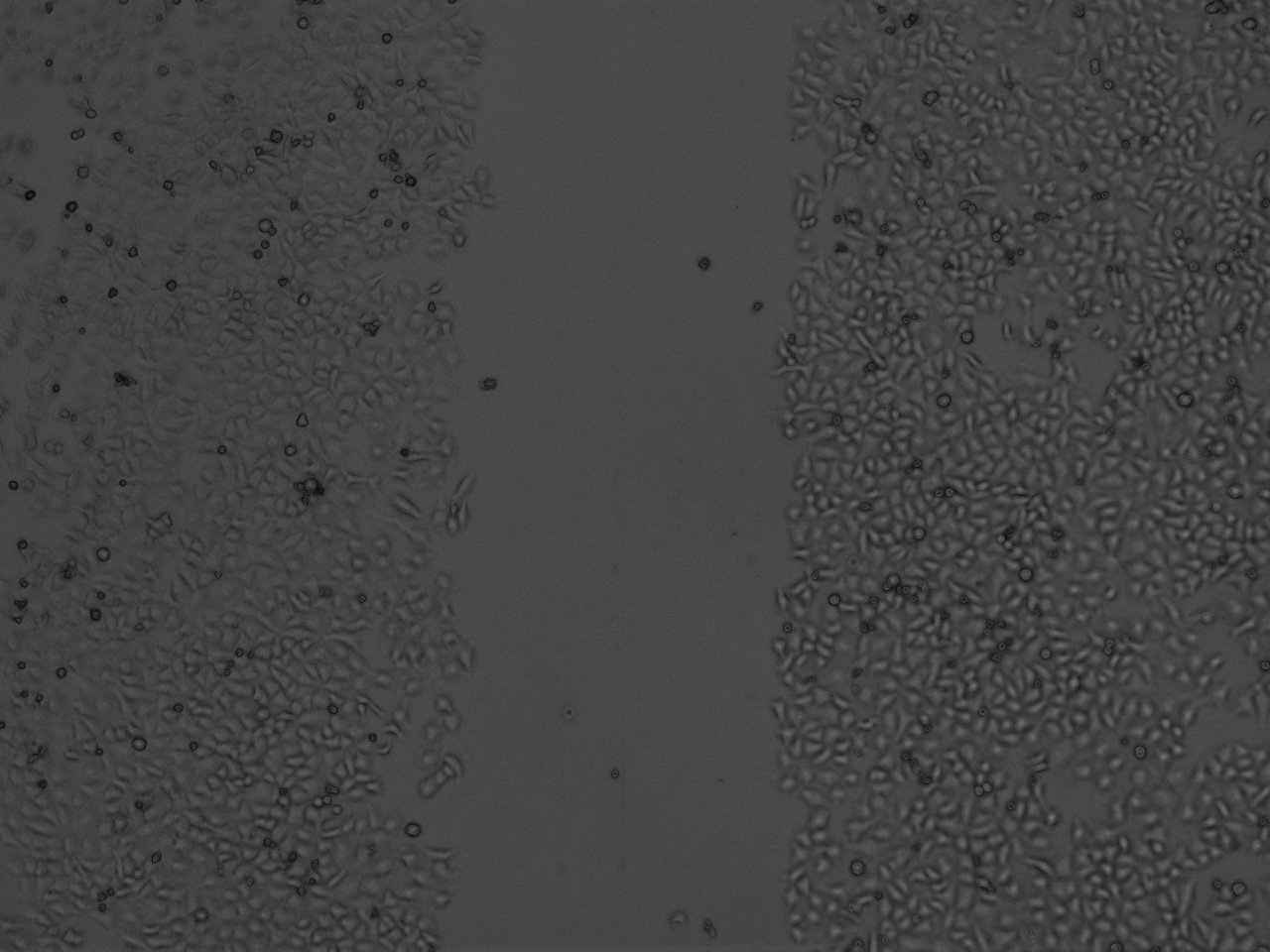

Supplement: Supplemental Information 13 [file peerj-12-18497-s013.zip › hucct1 functional experiment/NC knockdown (NC SI)/hucct1 nc si Wound Healing/picture/sicon k1 g 0h.jpg]

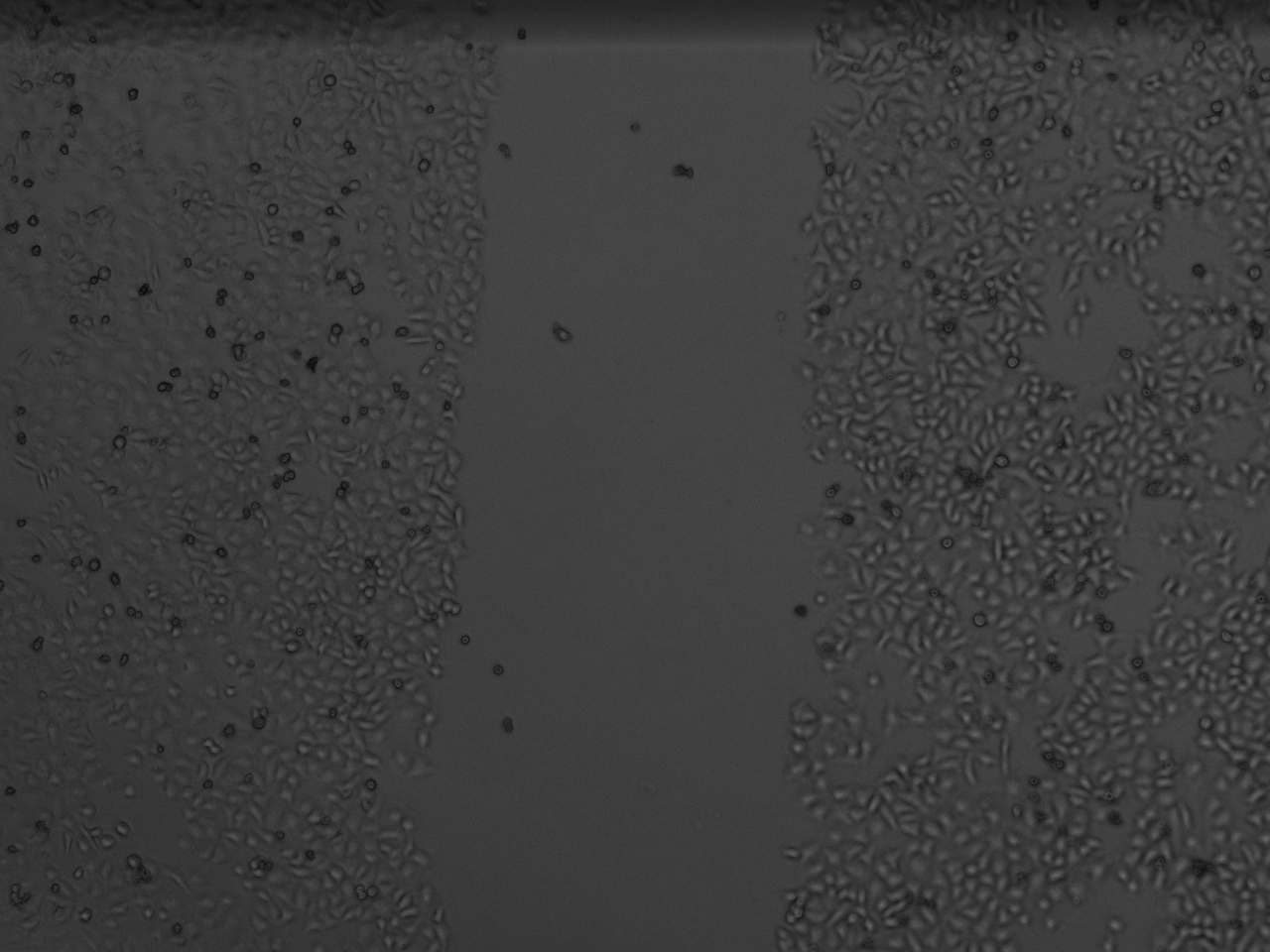

Supplement: Supplemental Information 13 [file peerj-12-18497-s013.zip › hucct1 functional experiment/NC knockdown (NC SI)/hucct1 nc si Wound Healing/picture/sicon k1 h 0h.jpg]

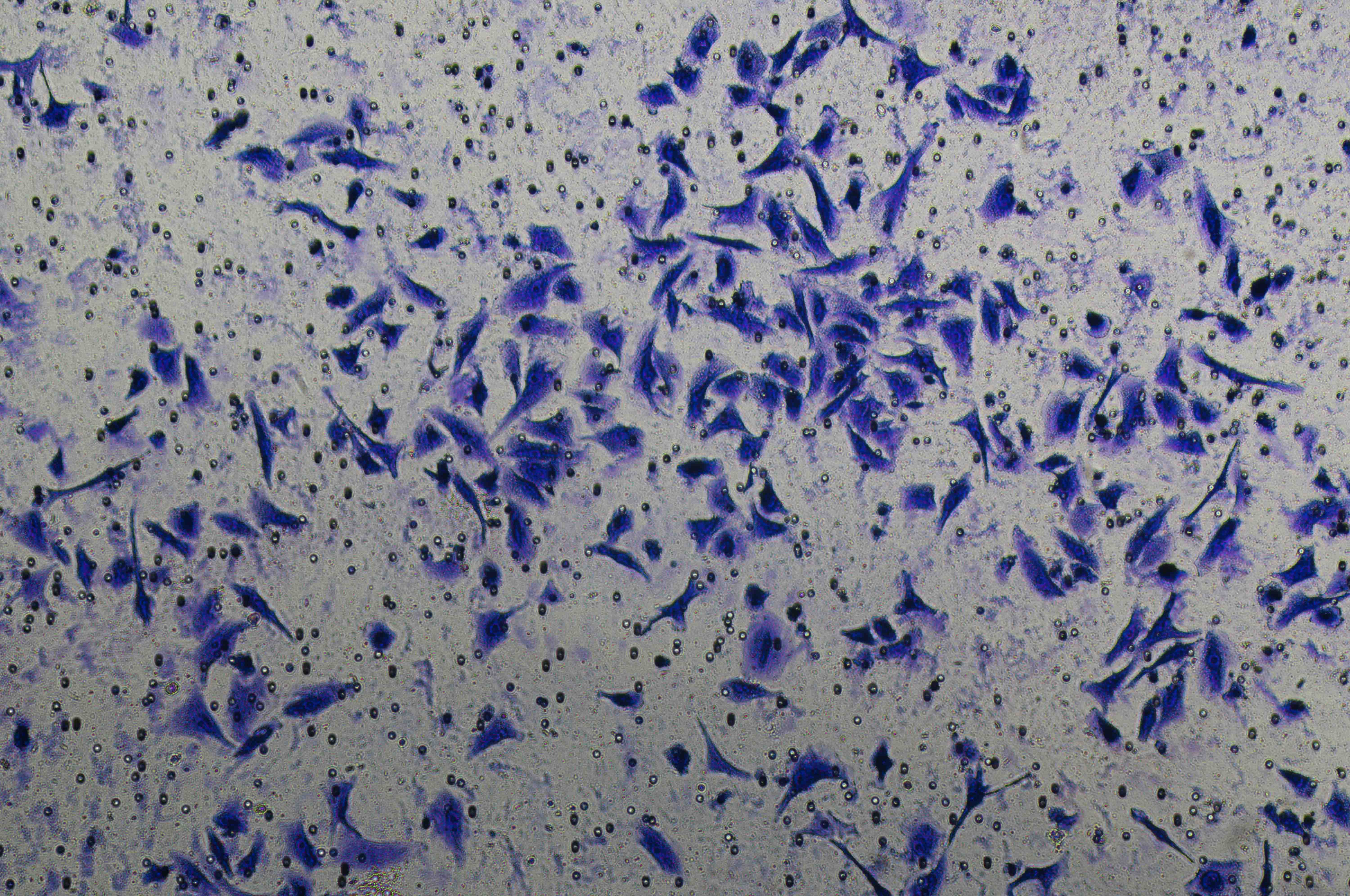

Supplement: Supplemental Information 14 [file peerj-12-18497-s014.zip › qbc939 functional experiment/control overexpression (nc oe)/qbc Invasion nc oe/picture/qbc939 nc 孔1 10倍01.jpg]

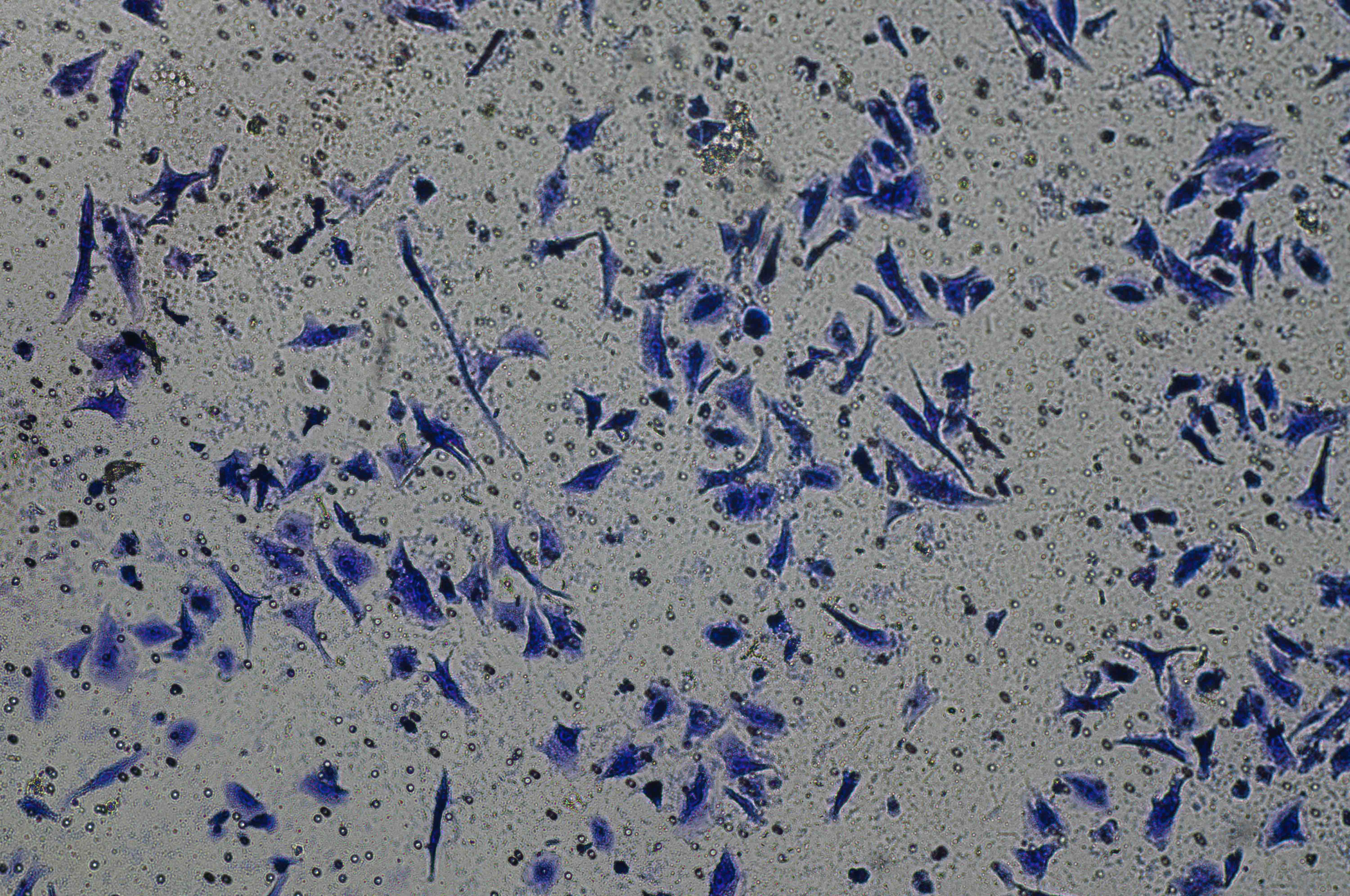

Supplement: Supplemental Information 14 [file peerj-12-18497-s014.zip › qbc939 functional experiment/control overexpression (nc oe)/qbc Invasion nc oe/picture/qbc939 nc 孔2 10倍01.jpg]

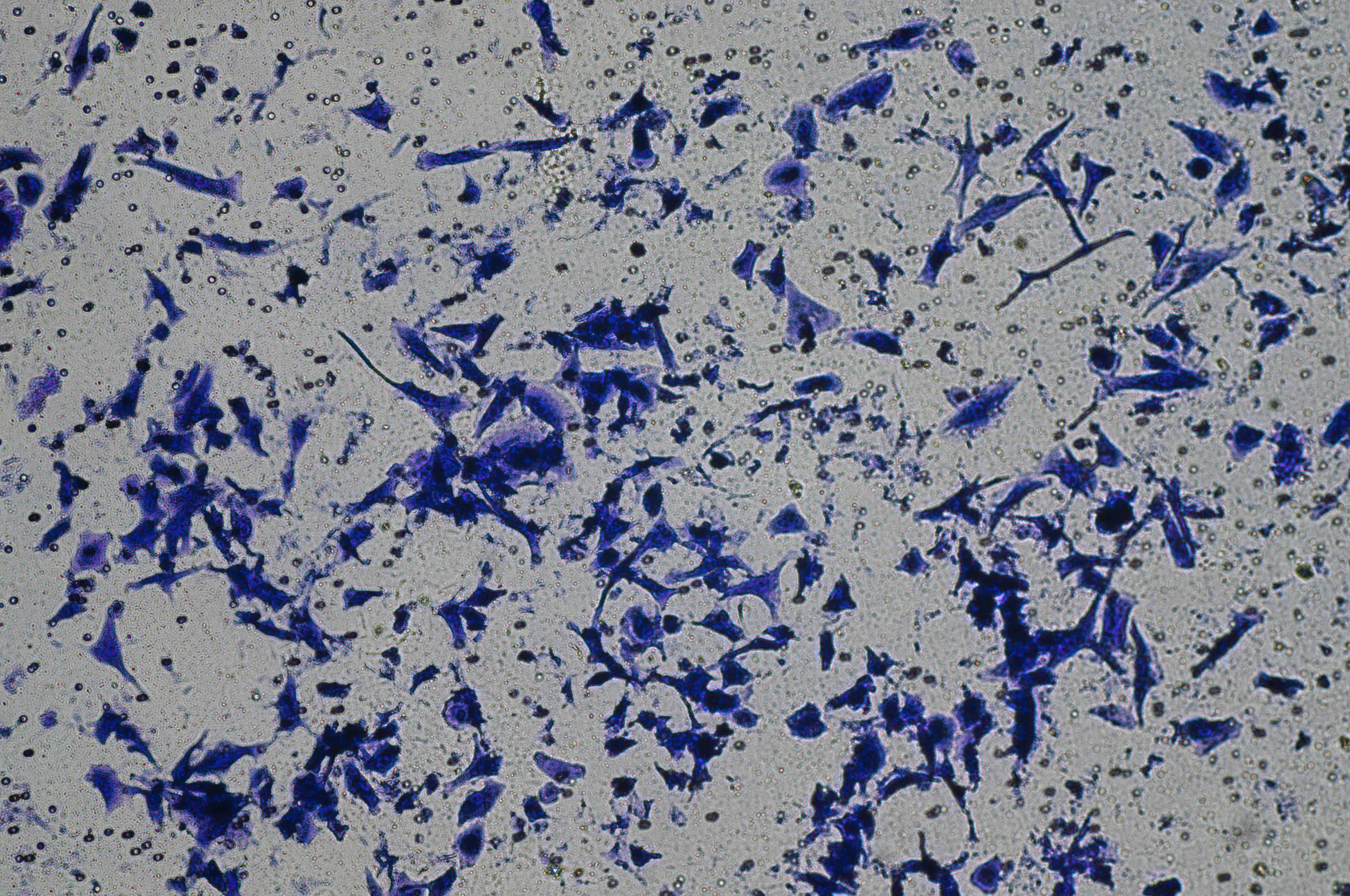

Supplement: Supplemental Information 14 [file peerj-12-18497-s014.zip › qbc939 functional experiment/control overexpression (nc oe)/qbc Invasion nc oe/picture/qbc939 nc 孔3 10倍01.jpg]

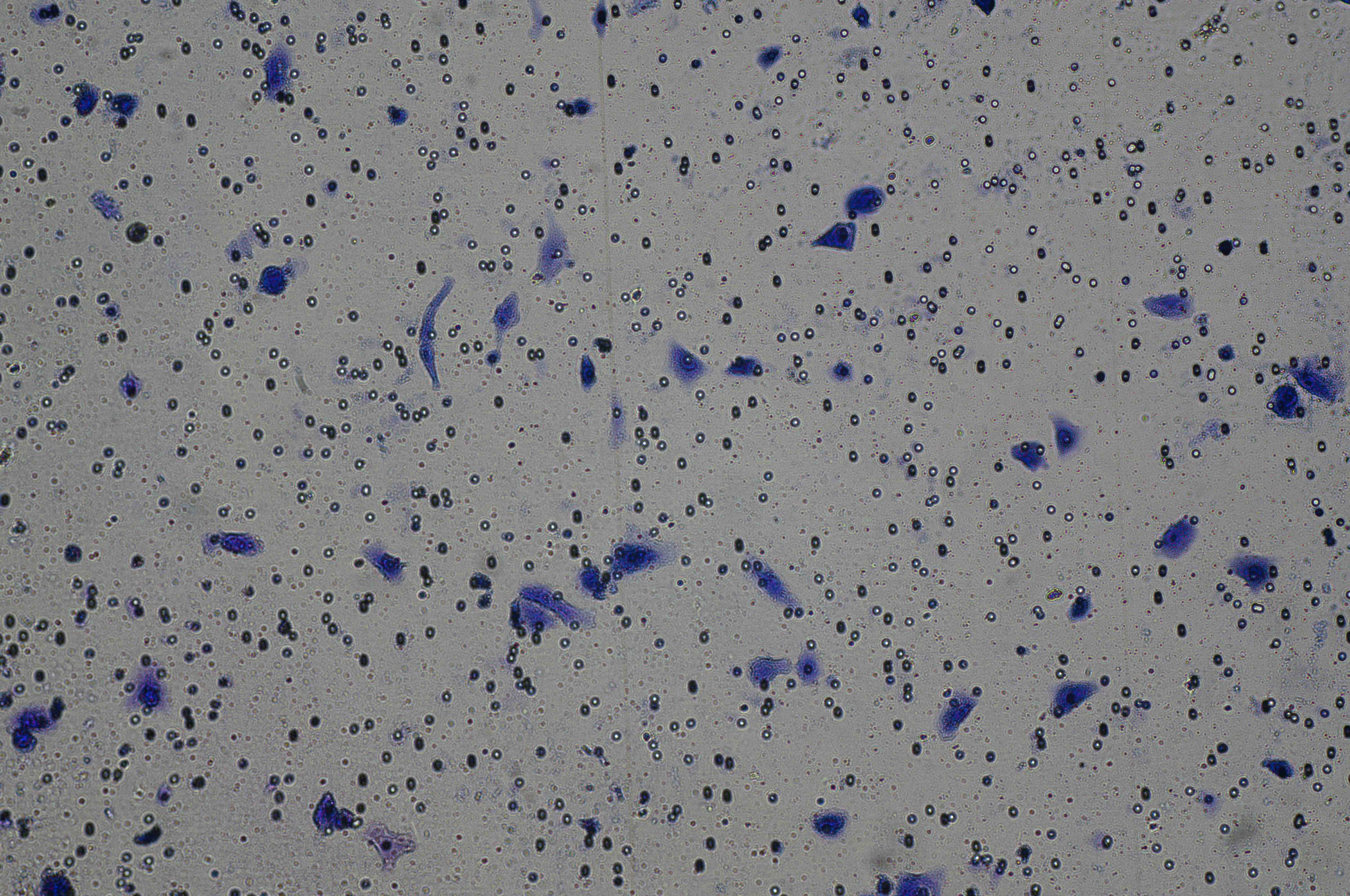

Supplement: Supplemental Information 14 [file peerj-12-18497-s014.zip › qbc939 functional experiment/control overexpression (nc oe)/qbc Invasion nc oe/picture/qbc939 oe 孔1 10倍02.jpg]

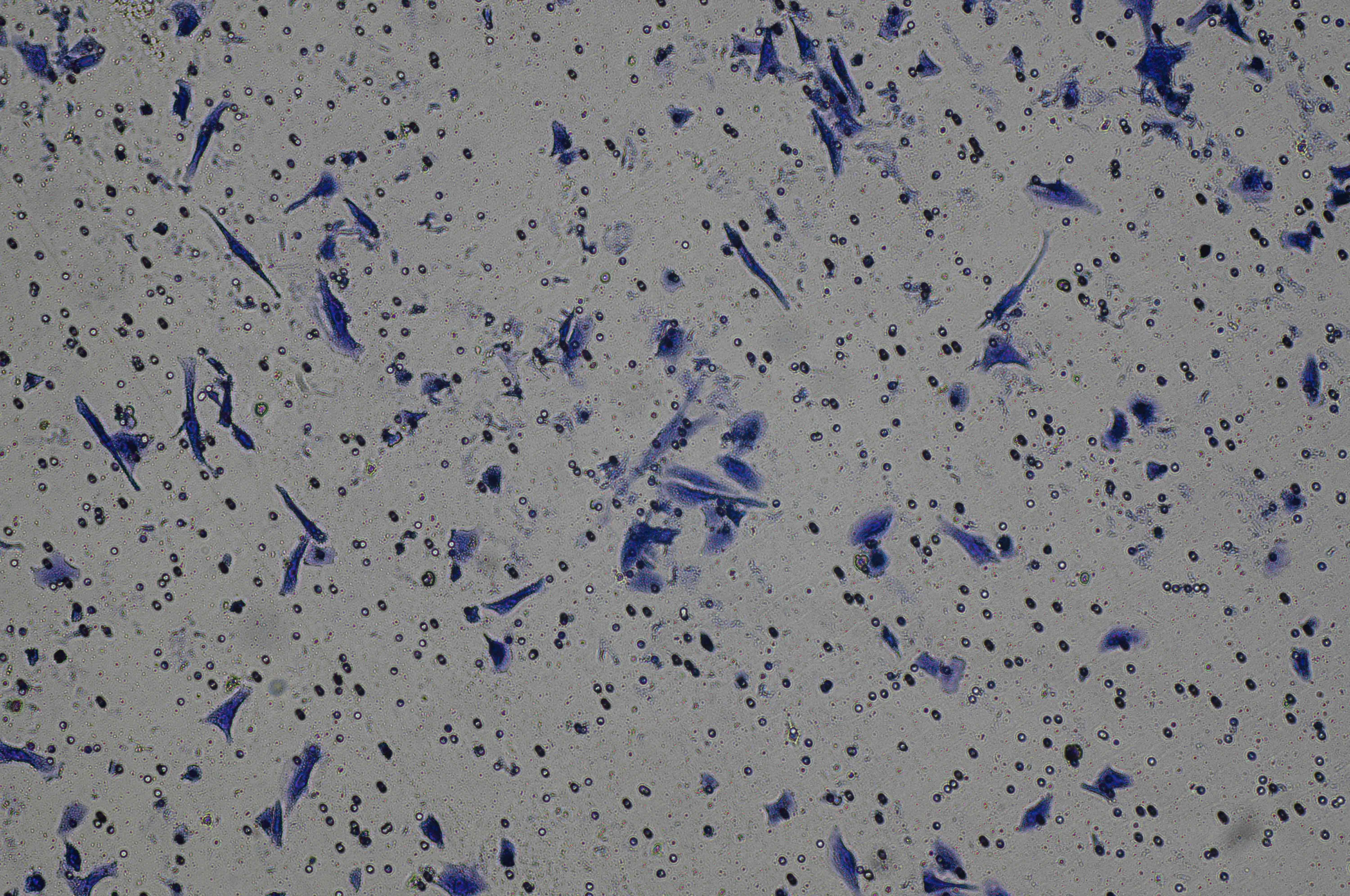

Supplement: Supplemental Information 14 [file peerj-12-18497-s014.zip › qbc939 functional experiment/control overexpression (nc oe)/qbc Invasion nc oe/picture/qbc939 oe 孔2 10倍02.jpg]

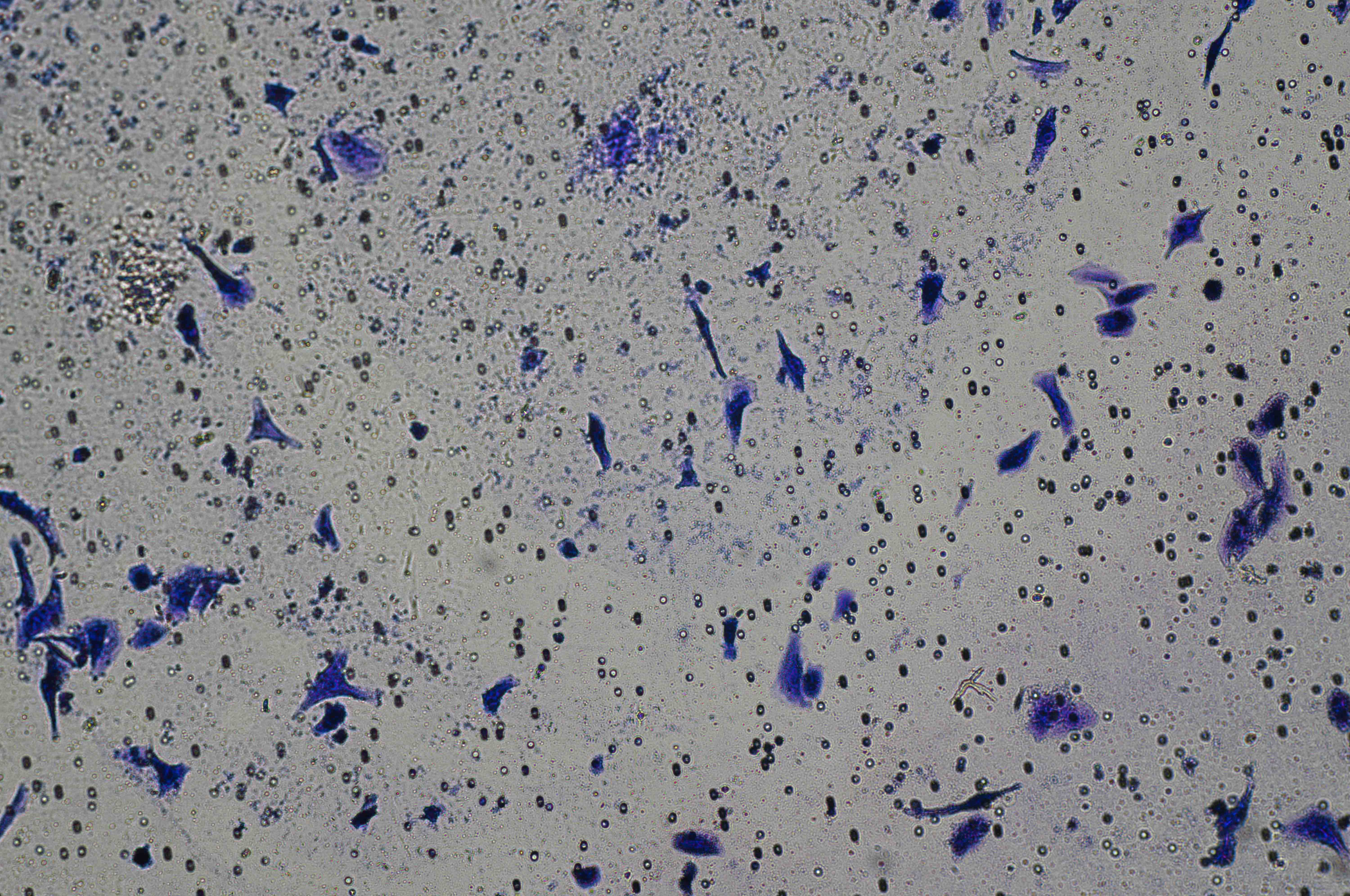

Supplement: Supplemental Information 14 [file peerj-12-18497-s014.zip › qbc939 functional experiment/control overexpression (nc oe)/qbc Invasion nc oe/picture/qbc939 oe 孔3 10倍01.jpg]

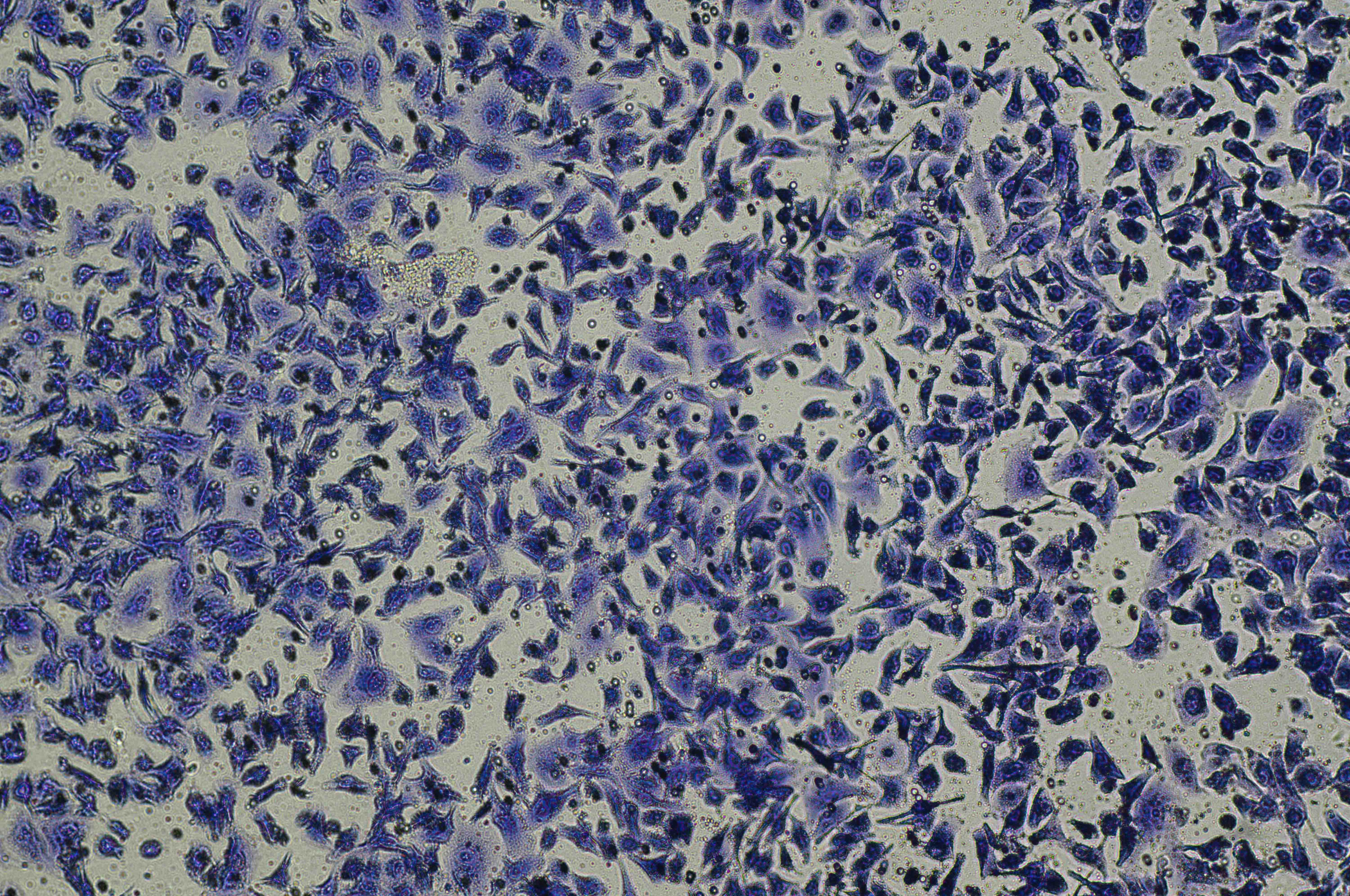

Supplement: Supplemental Information 14 [file peerj-12-18497-s014.zip › qbc939 functional experiment/control overexpression (nc oe)/qbc migration nc oe/picture/qbc939 nc 孔1 10倍01.jpg]

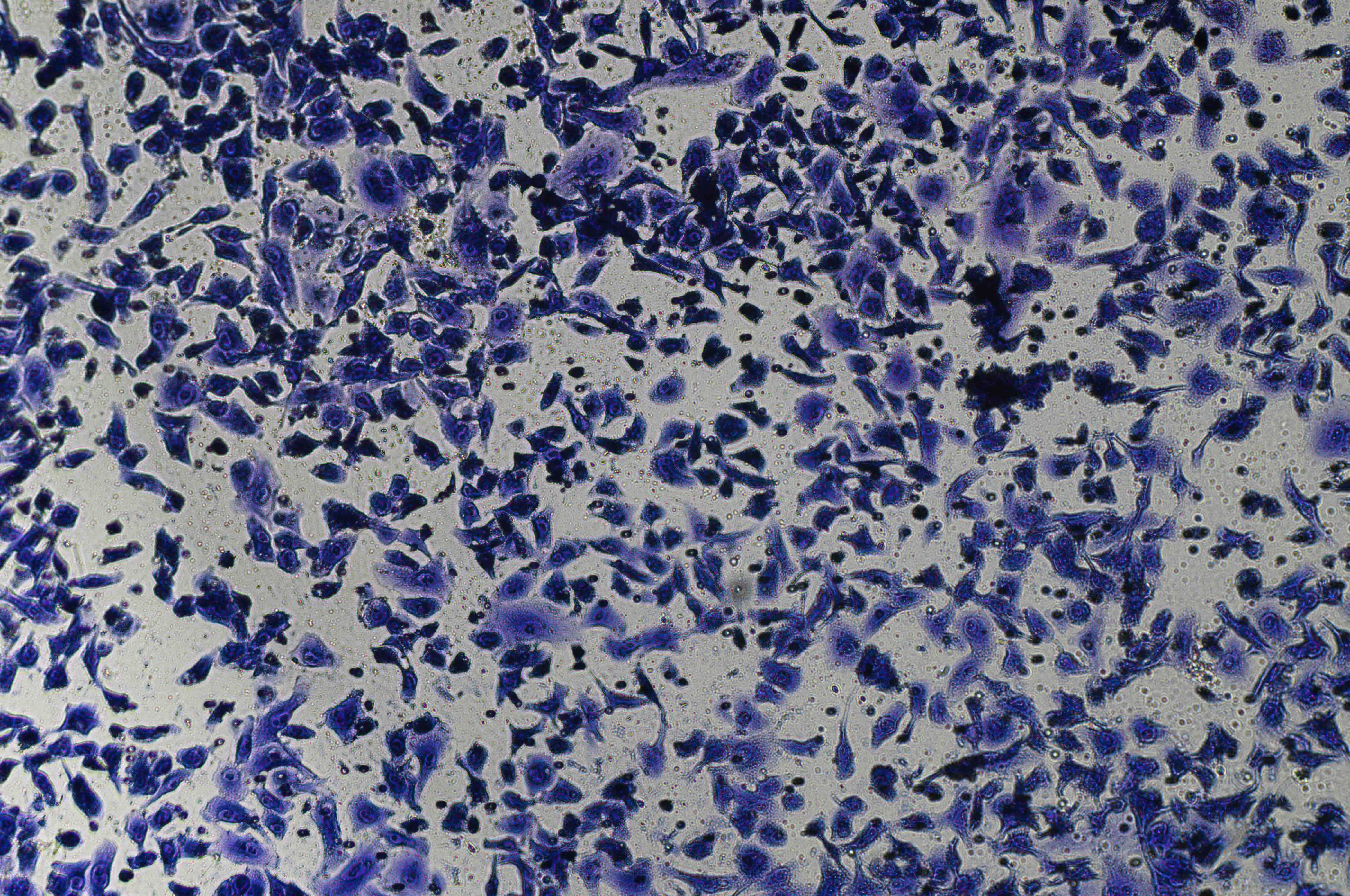

Supplement: Supplemental Information 14 [file peerj-12-18497-s014.zip › qbc939 functional experiment/control overexpression (nc oe)/qbc migration nc oe/picture/qbc939 nc 孔2 10倍05.jpg]

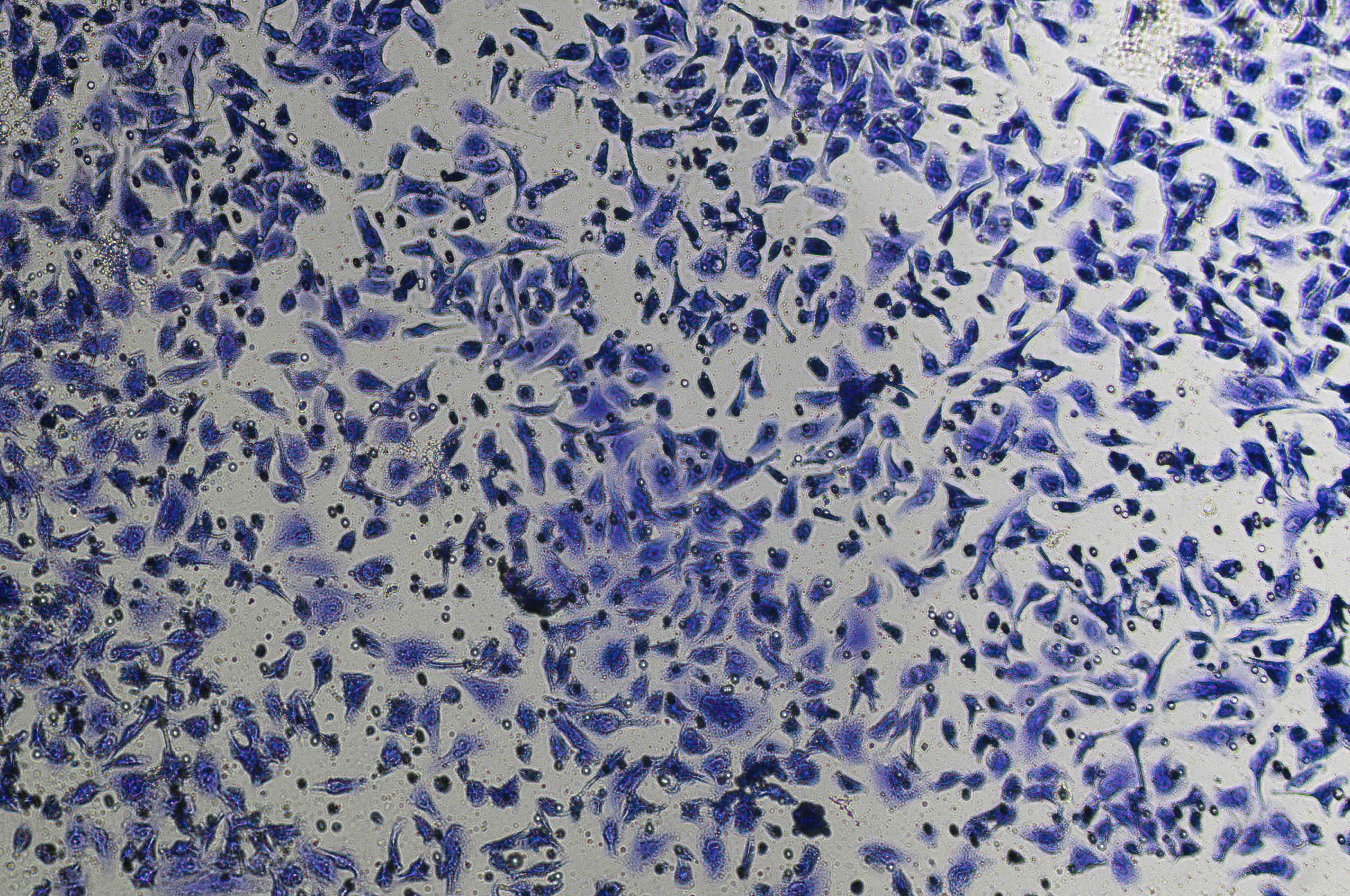

Supplement: Supplemental Information 14 [file peerj-12-18497-s014.zip › qbc939 functional experiment/control overexpression (nc oe)/qbc migration nc oe/picture/qbc939 nc 孔3 10倍06.jpg]

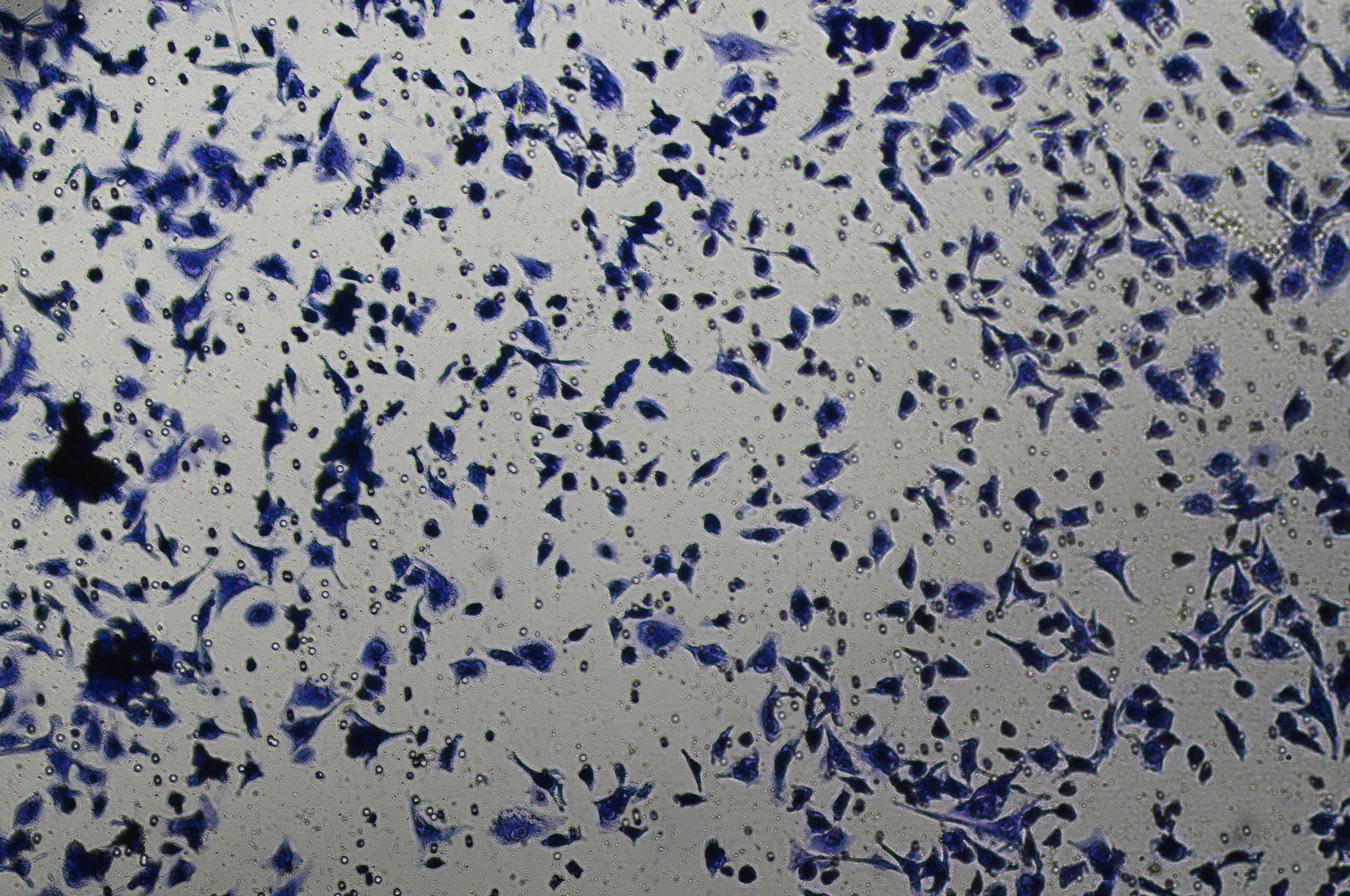

Supplement: Supplemental Information 14 [file peerj-12-18497-s014.zip › qbc939 functional experiment/control overexpression (nc oe)/qbc migration nc oe/picture/qbc939 oe 孔1 10倍01.jpg]

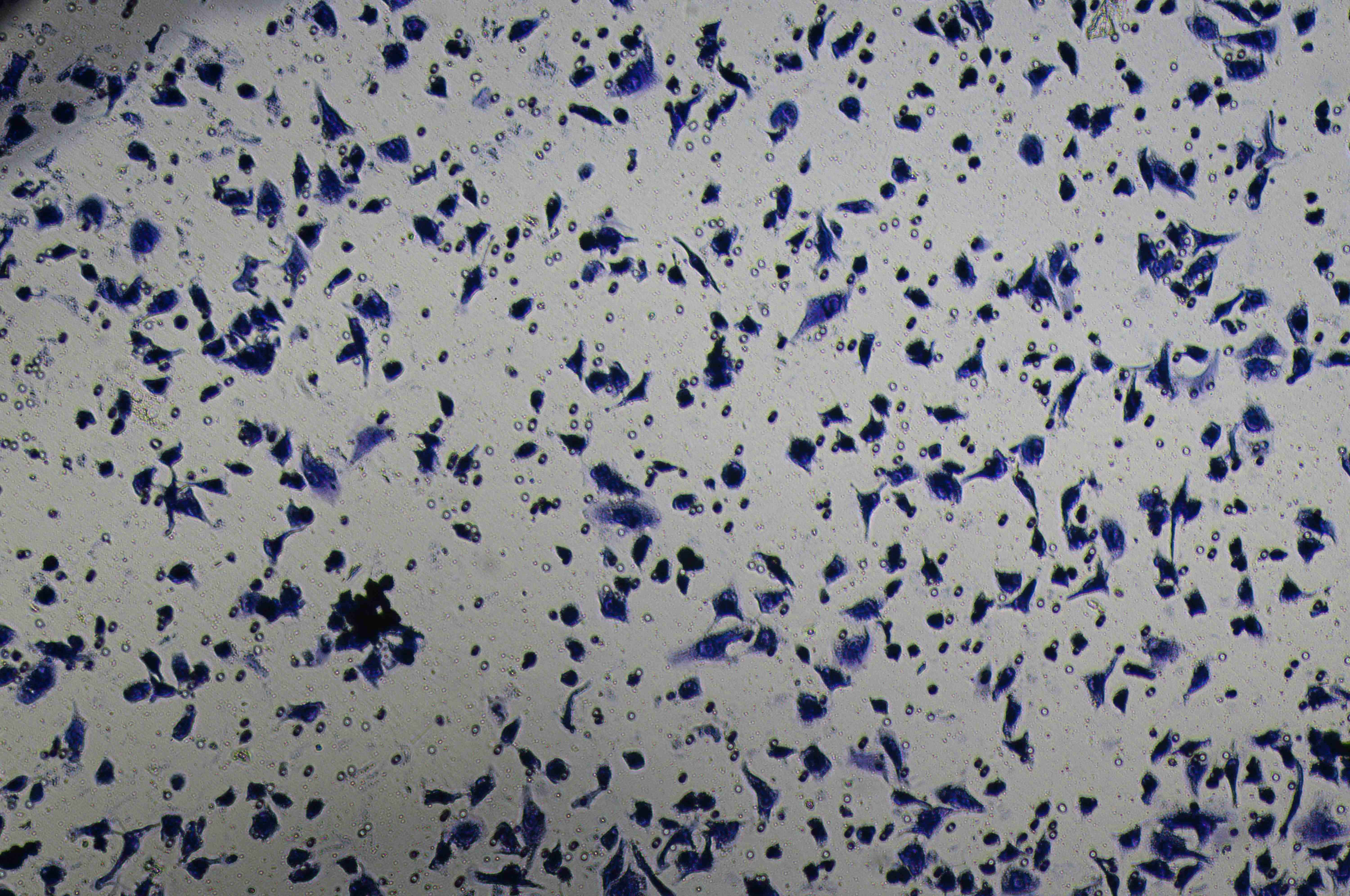

Supplement: Supplemental Information 14 [file peerj-12-18497-s014.zip › qbc939 functional experiment/control overexpression (nc oe)/qbc migration nc oe/picture/qbc939 oe 孔2 10倍02.jpg]

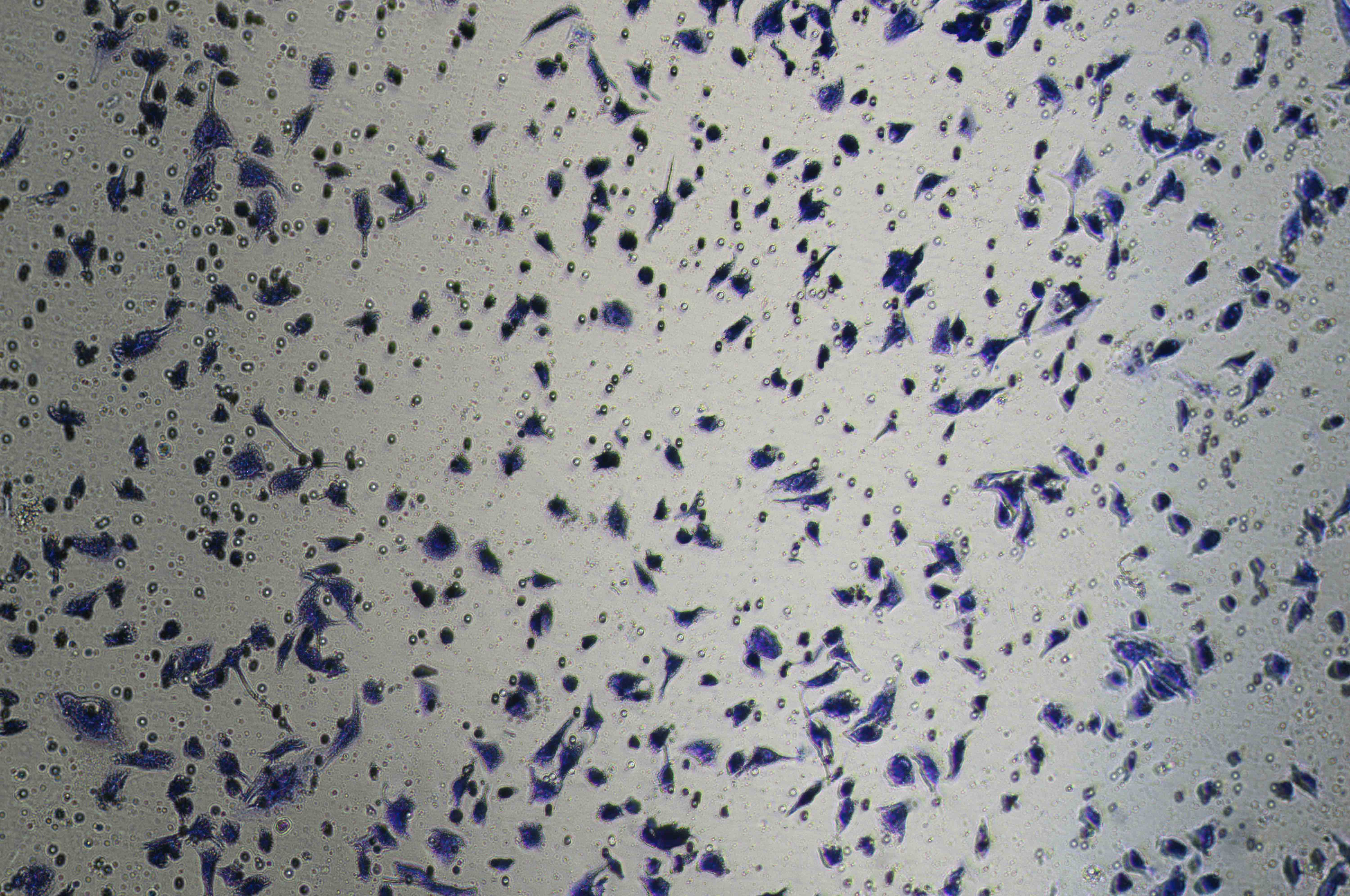

Supplement: Supplemental Information 14 [file peerj-12-18497-s014.zip › qbc939 functional experiment/control overexpression (nc oe)/qbc migration nc oe/picture/qbc939 oe 孔3 10倍09.jpg]

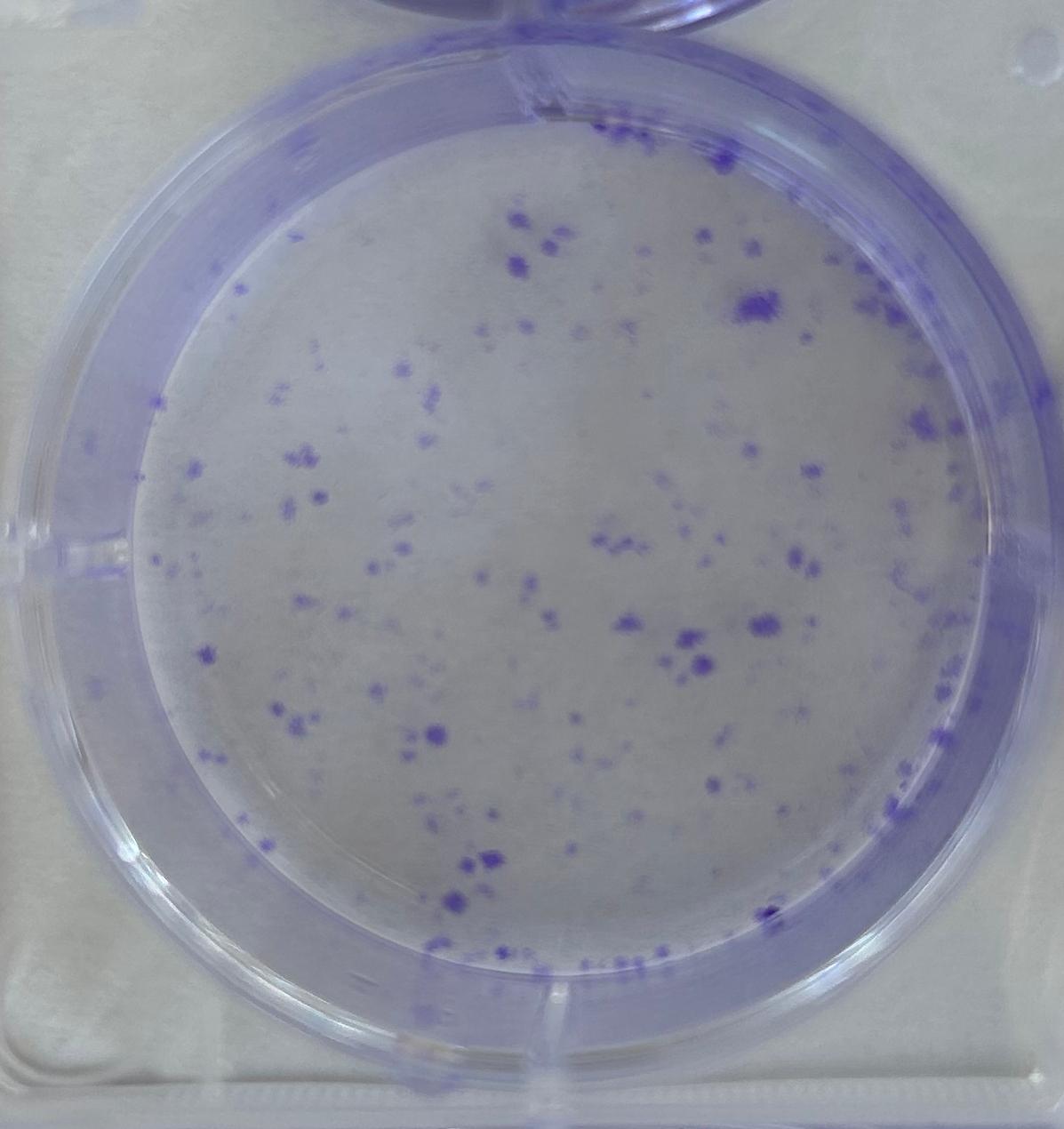

Supplement: Supplemental Information 14 [file peerj-12-18497-s014.zip › qbc939 functional experiment/control overexpression (nc oe)/qbc939 clone formation nc oe/clec3b 1.jpg]

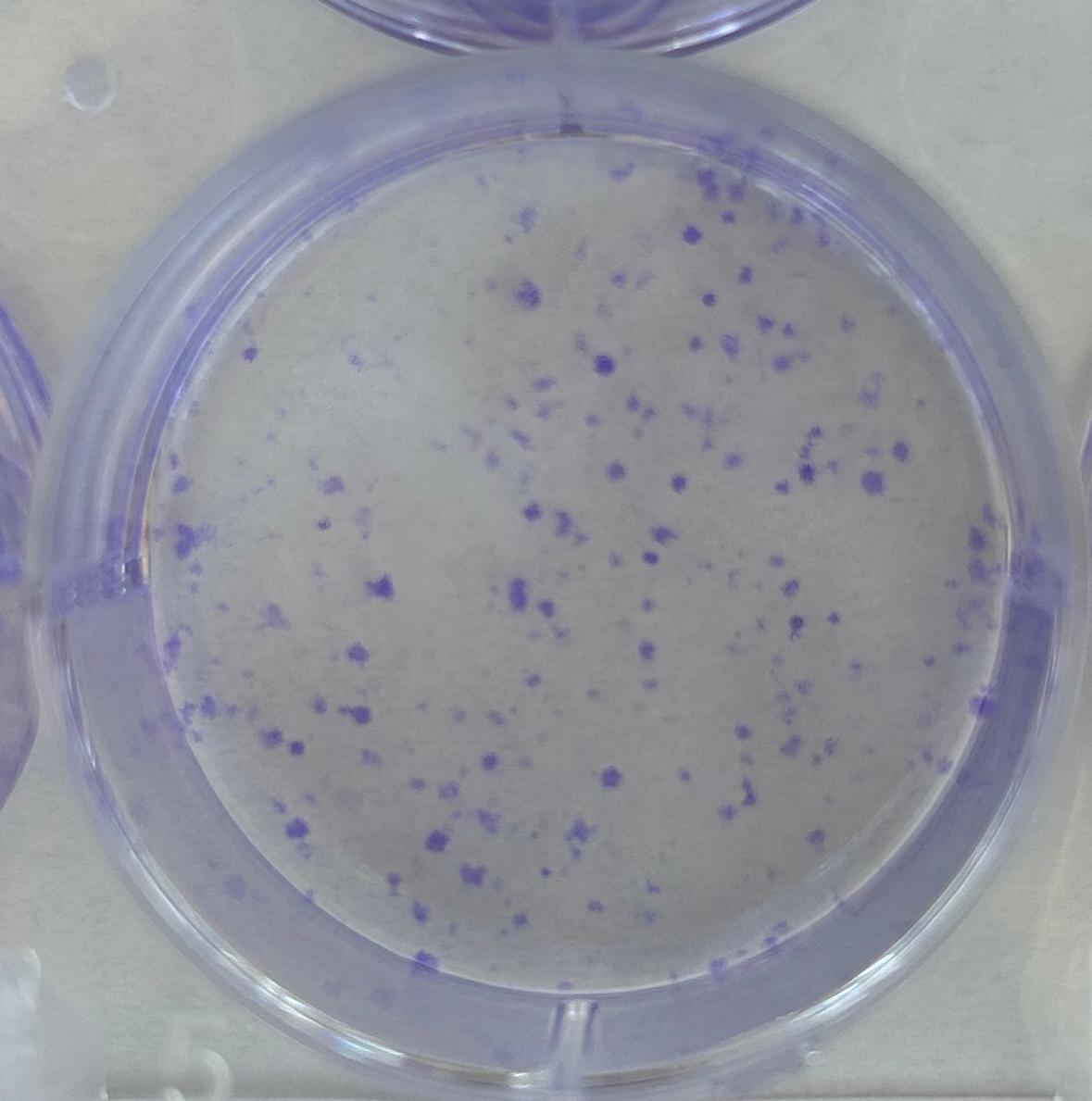

Supplement: Supplemental Information 14 [file peerj-12-18497-s014.zip › qbc939 functional experiment/control overexpression (nc oe)/qbc939 clone formation nc oe/clec3b 2.jpg]

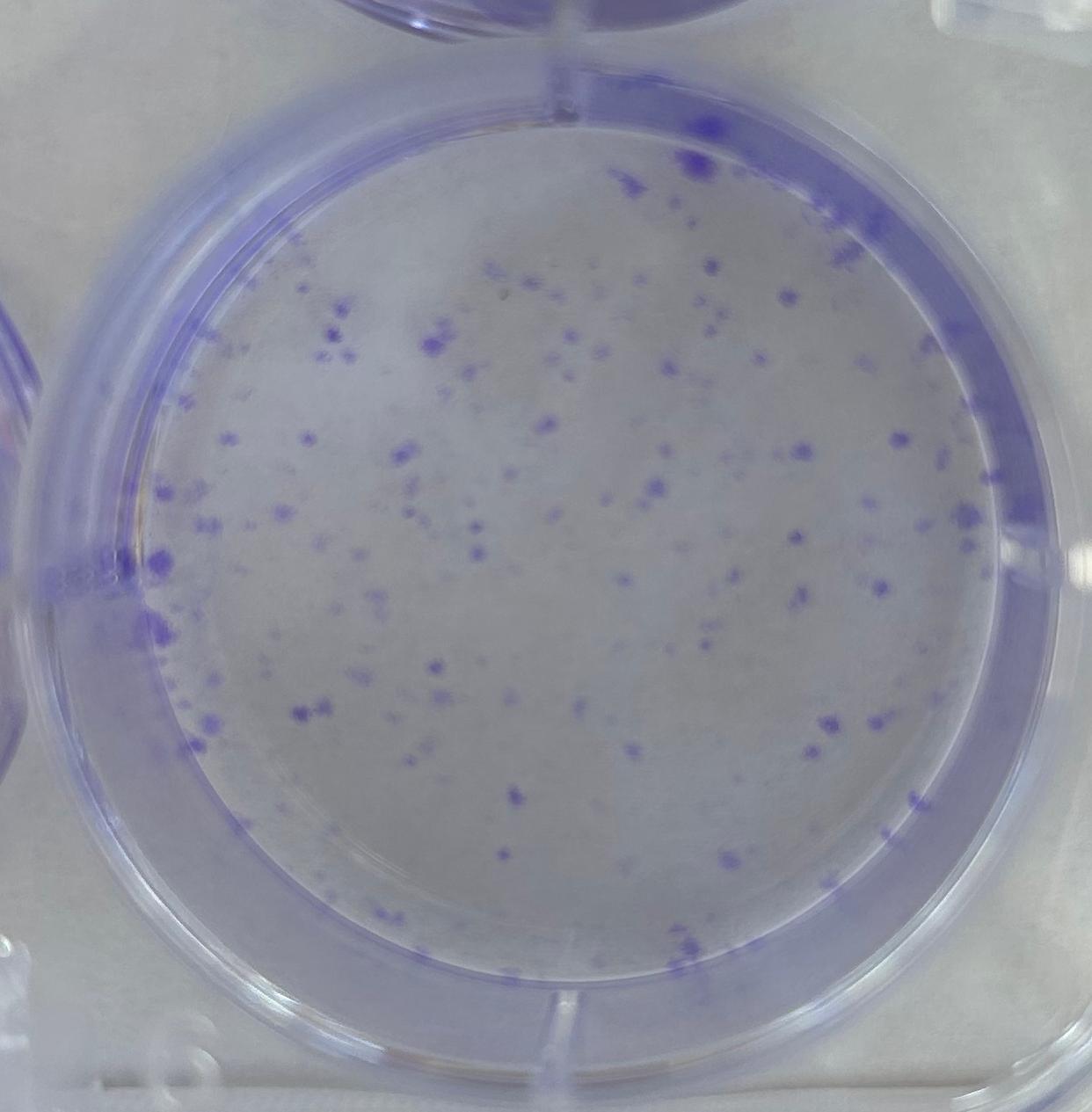

Supplement: Supplemental Information 14 [file peerj-12-18497-s014.zip › qbc939 functional experiment/control overexpression (nc oe)/qbc939 clone formation nc oe/clec3b 3.jpg]

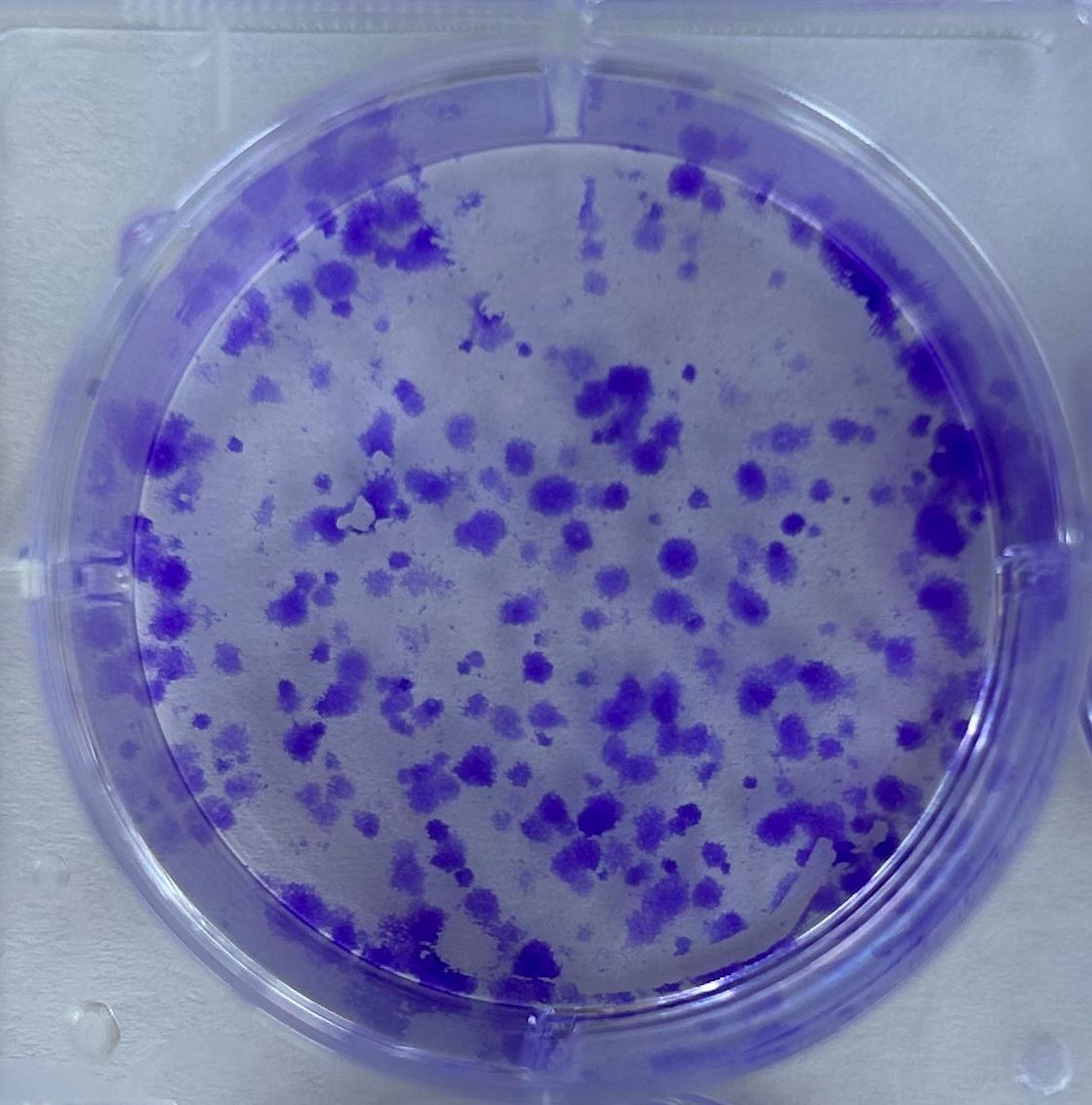

Supplement: Supplemental Information 14 [file peerj-12-18497-s014.zip › qbc939 functional experiment/control overexpression (nc oe)/qbc939 clone formation nc oe/nc1.jpg]

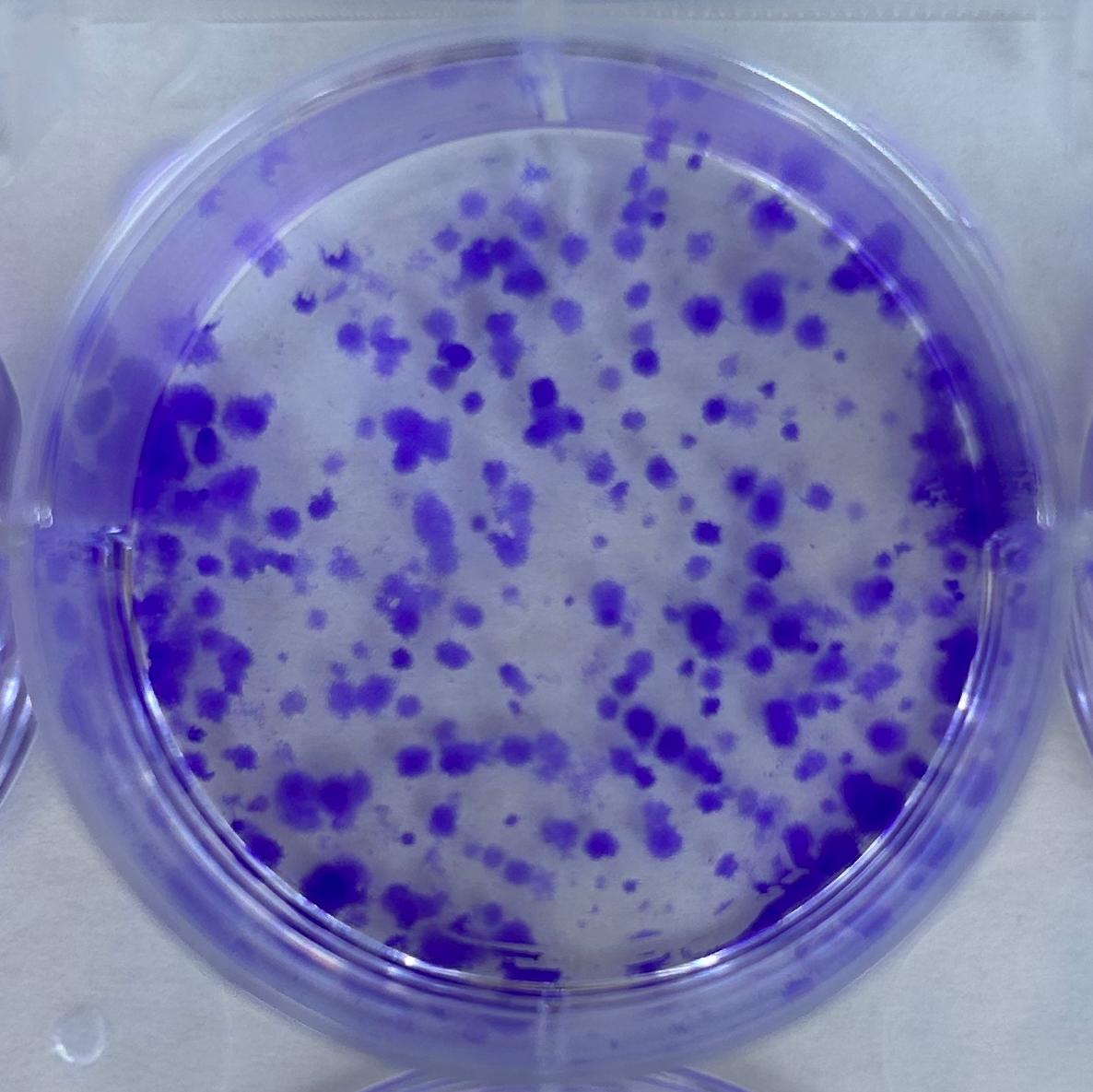

Supplement: Supplemental Information 14 [file peerj-12-18497-s014.zip › qbc939 functional experiment/control overexpression (nc oe)/qbc939 clone formation nc oe/nc2.jpg]

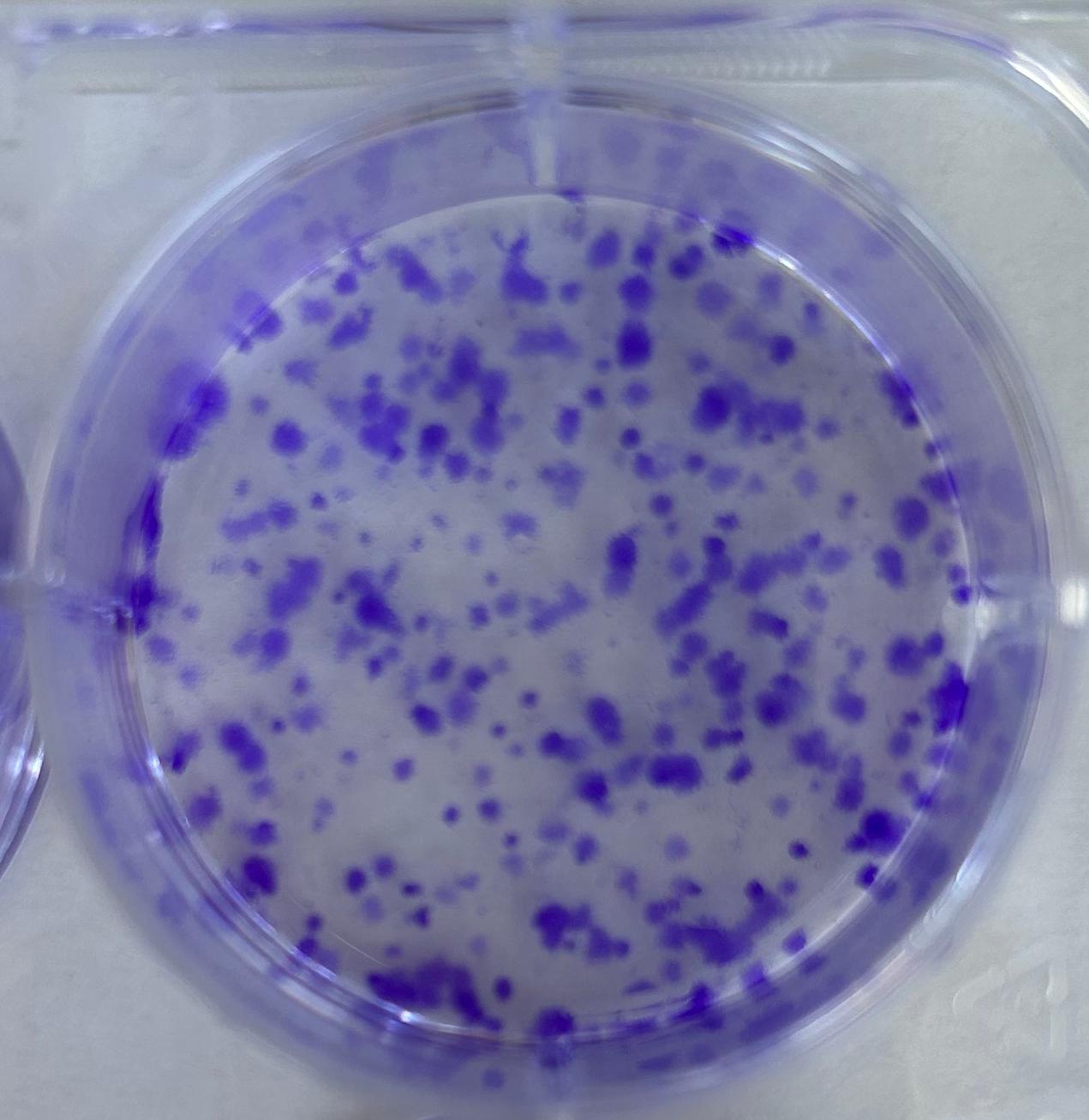

Supplement: Supplemental Information 14 [file peerj-12-18497-s014.zip › qbc939 functional experiment/control overexpression (nc oe)/qbc939 clone formation nc oe/nc3.jpg]

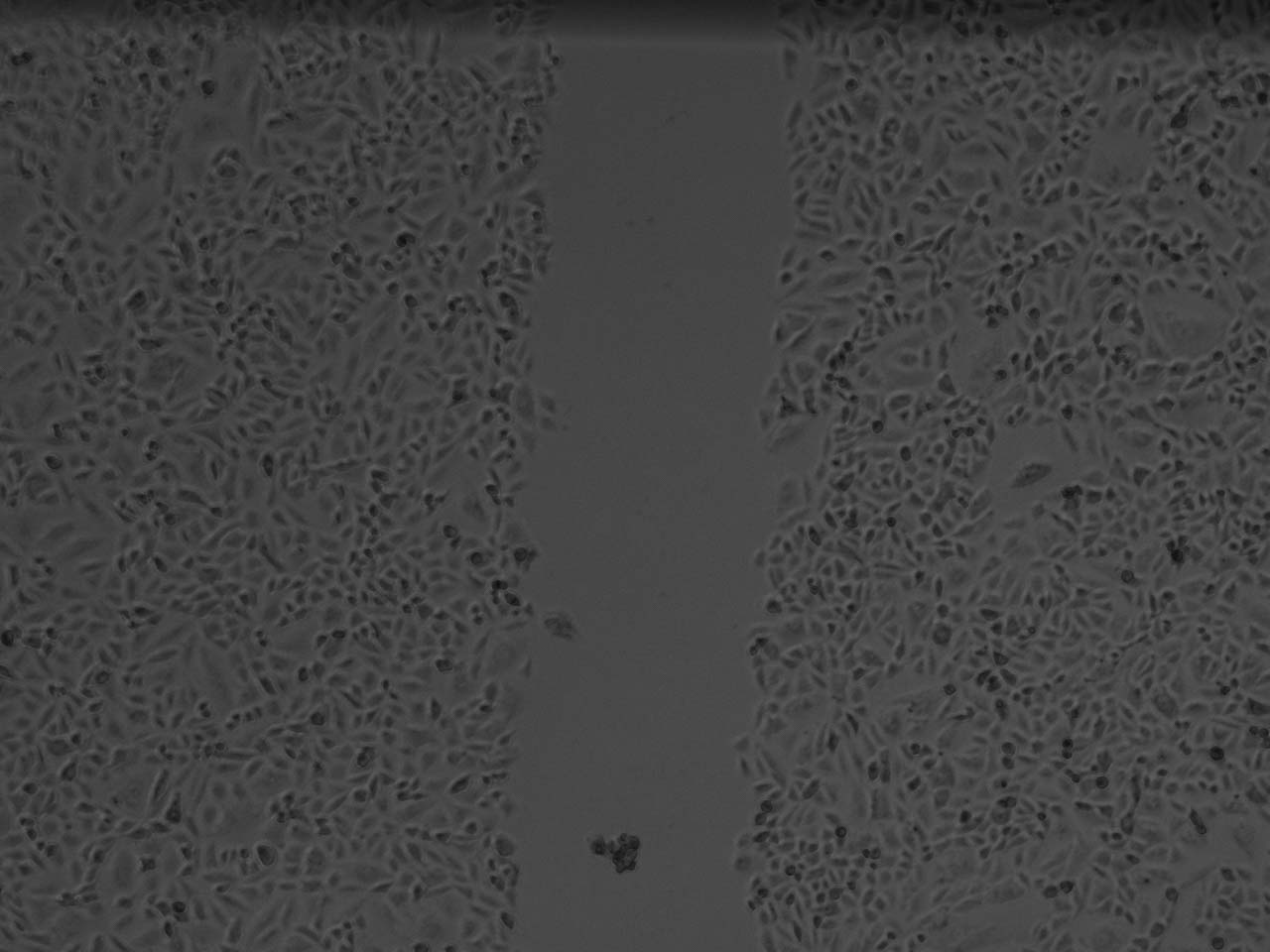

Supplement: Supplemental Information 14 [file peerj-12-18497-s014.zip › qbc939 functional experiment/control overexpression (nc oe)/QBC939 Wound Healing NC OE/picture/k1 b oe 0h.jpg]

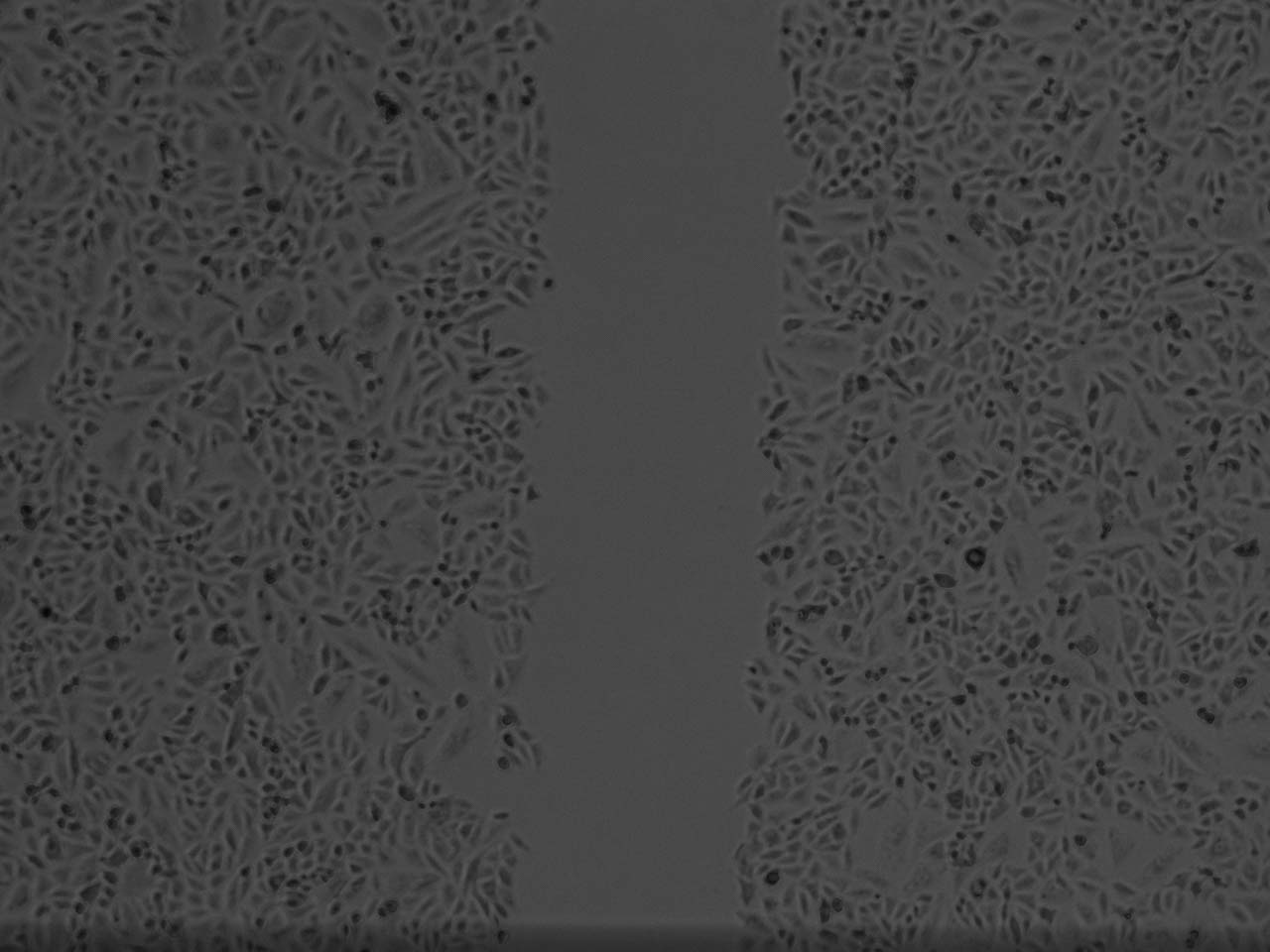

Supplement: Supplemental Information 14 [file peerj-12-18497-s014.zip › qbc939 functional experiment/control overexpression (nc oe)/QBC939 Wound Healing NC OE/picture/k1 c oe 0h.jpg]

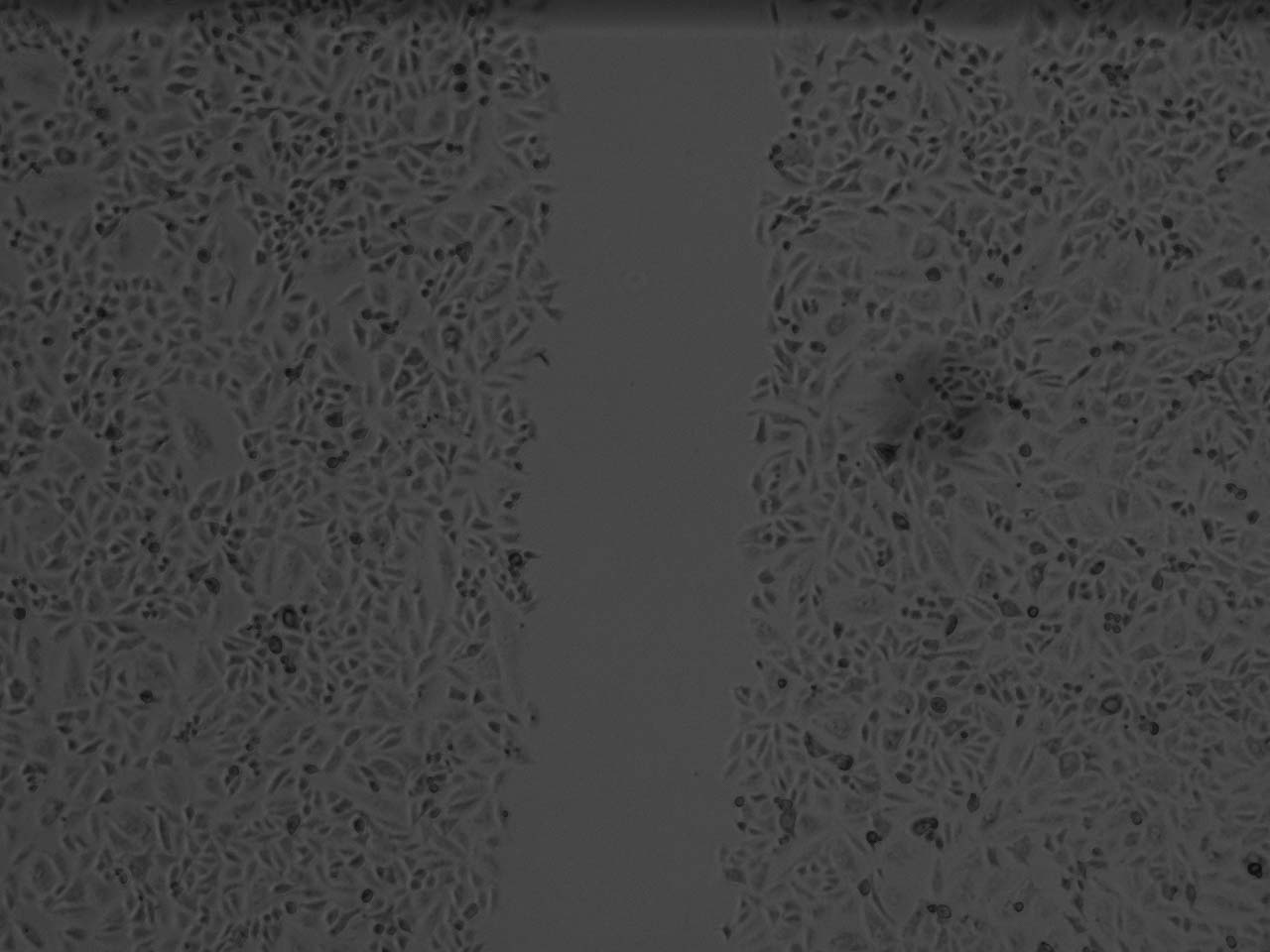

Supplement: Supplemental Information 14 [file peerj-12-18497-s014.zip › qbc939 functional experiment/control overexpression (nc oe)/QBC939 Wound Healing NC OE/picture/k1 d oe 0h.jpg]

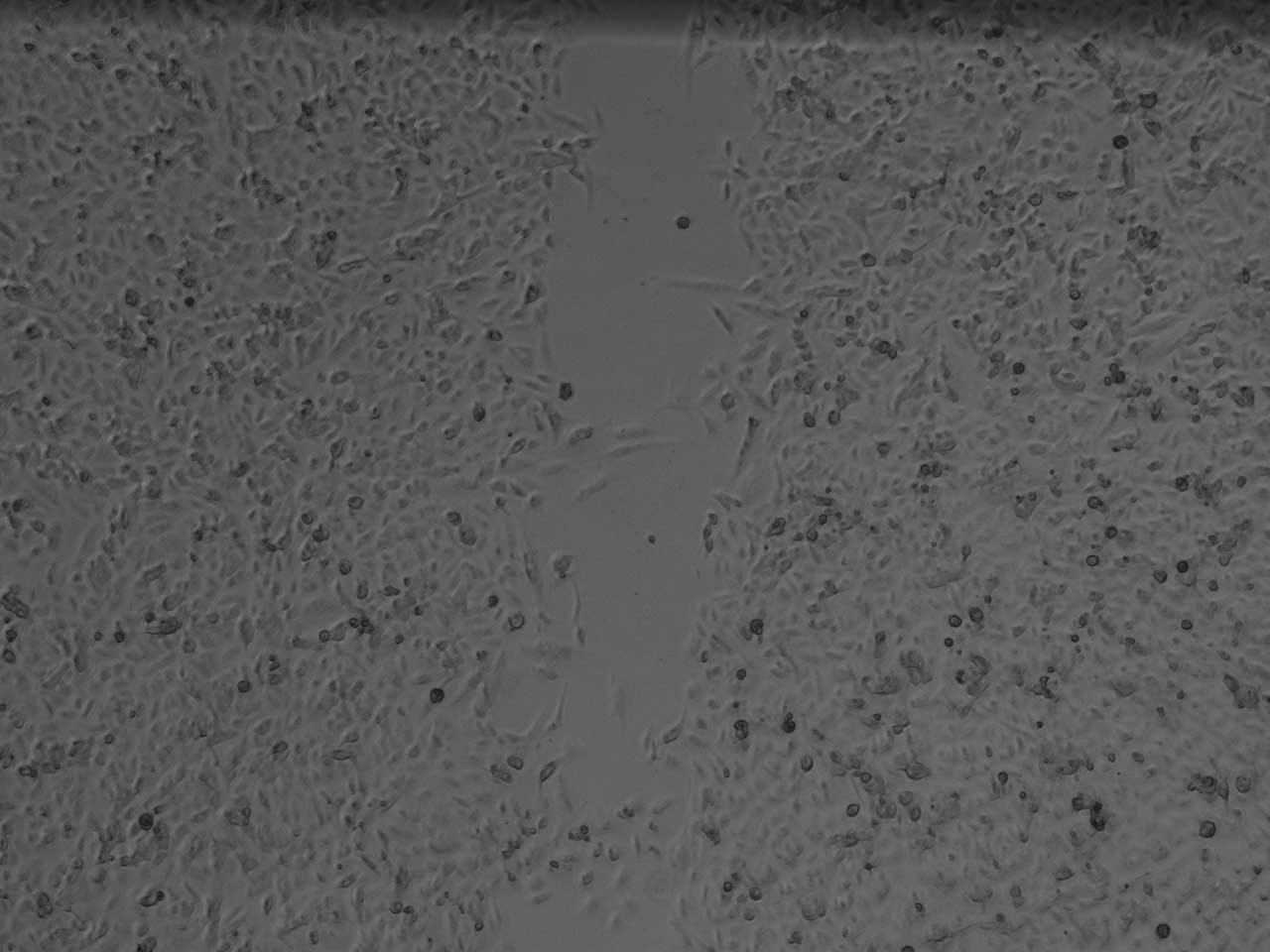

Supplement: Supplemental Information 14 [file peerj-12-18497-s014.zip › qbc939 functional experiment/control overexpression (nc oe)/QBC939 Wound Healing NC OE/picture/k1 b oe24h.jpg]

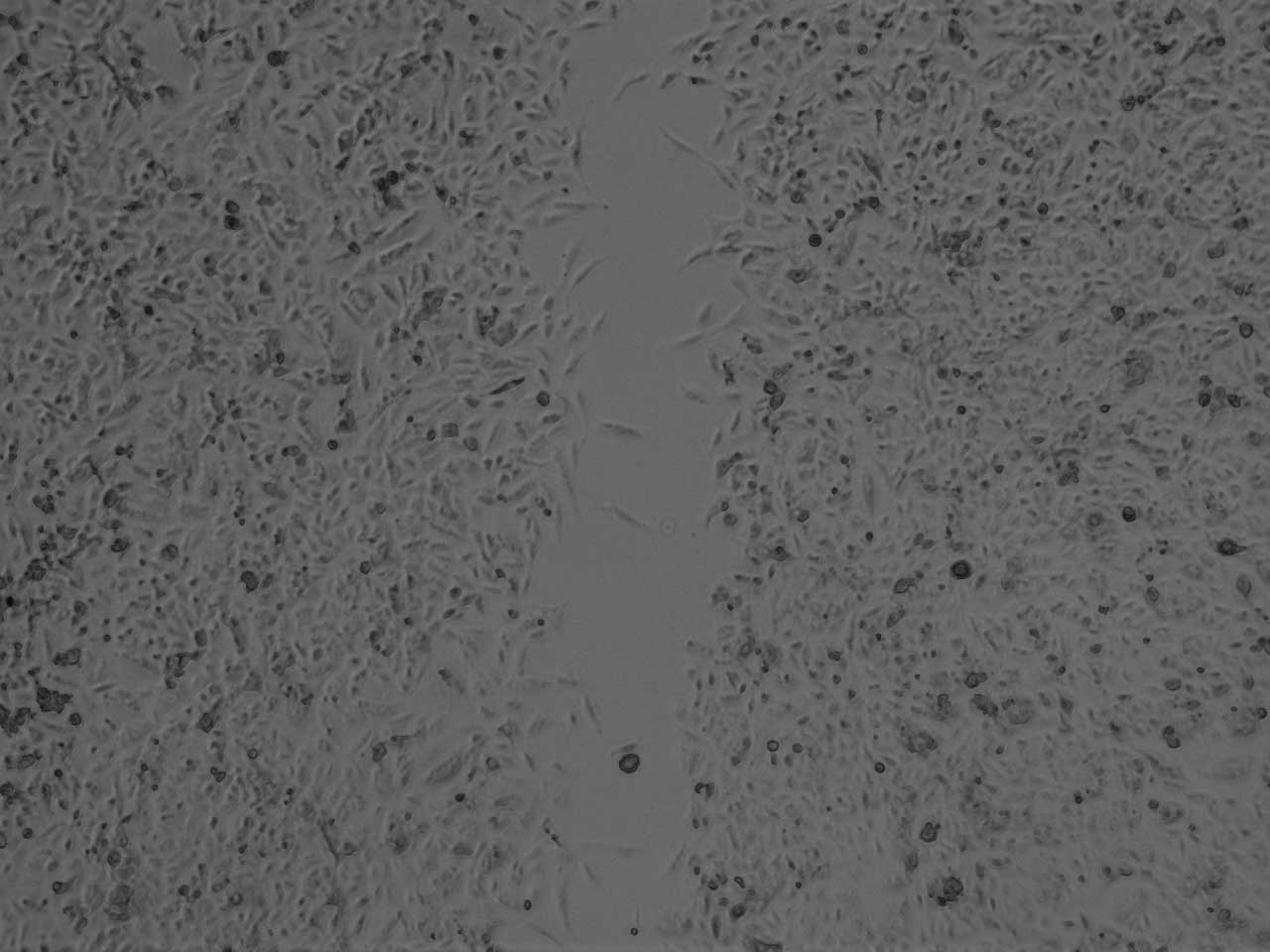

Supplement: Supplemental Information 14 [file peerj-12-18497-s014.zip › qbc939 functional experiment/control overexpression (nc oe)/QBC939 Wound Healing NC OE/picture/k1 c oe24h.jpg]

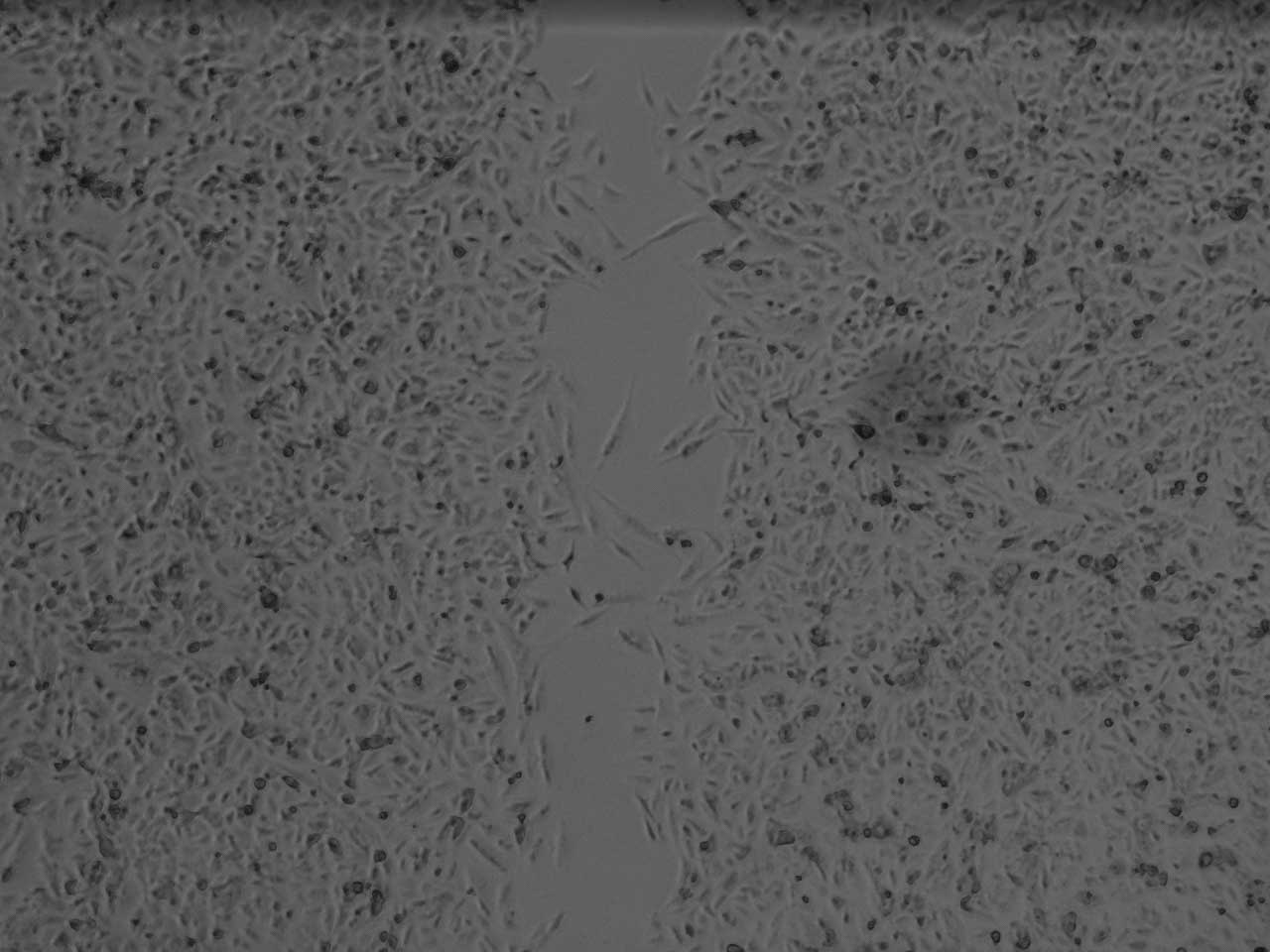

Supplement: Supplemental Information 14 [file peerj-12-18497-s014.zip › qbc939 functional experiment/control overexpression (nc oe)/QBC939 Wound Healing NC OE/picture/k1 d oe24h.jpg]

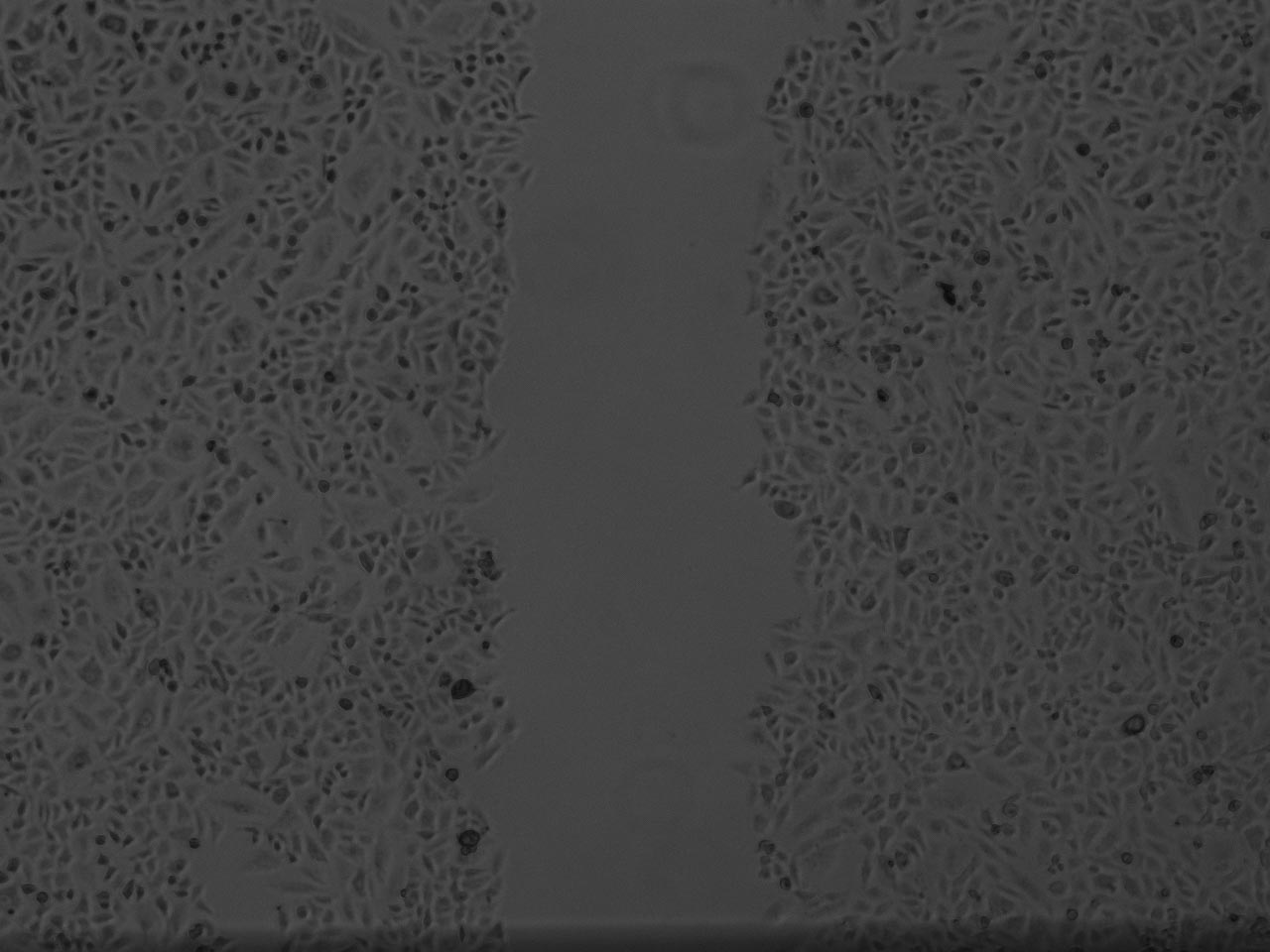

Supplement: Supplemental Information 14 [file peerj-12-18497-s014.zip › qbc939 functional experiment/control overexpression (nc oe)/QBC939 Wound Healing NC OE/picture/k2 q nc 0h.jpg]

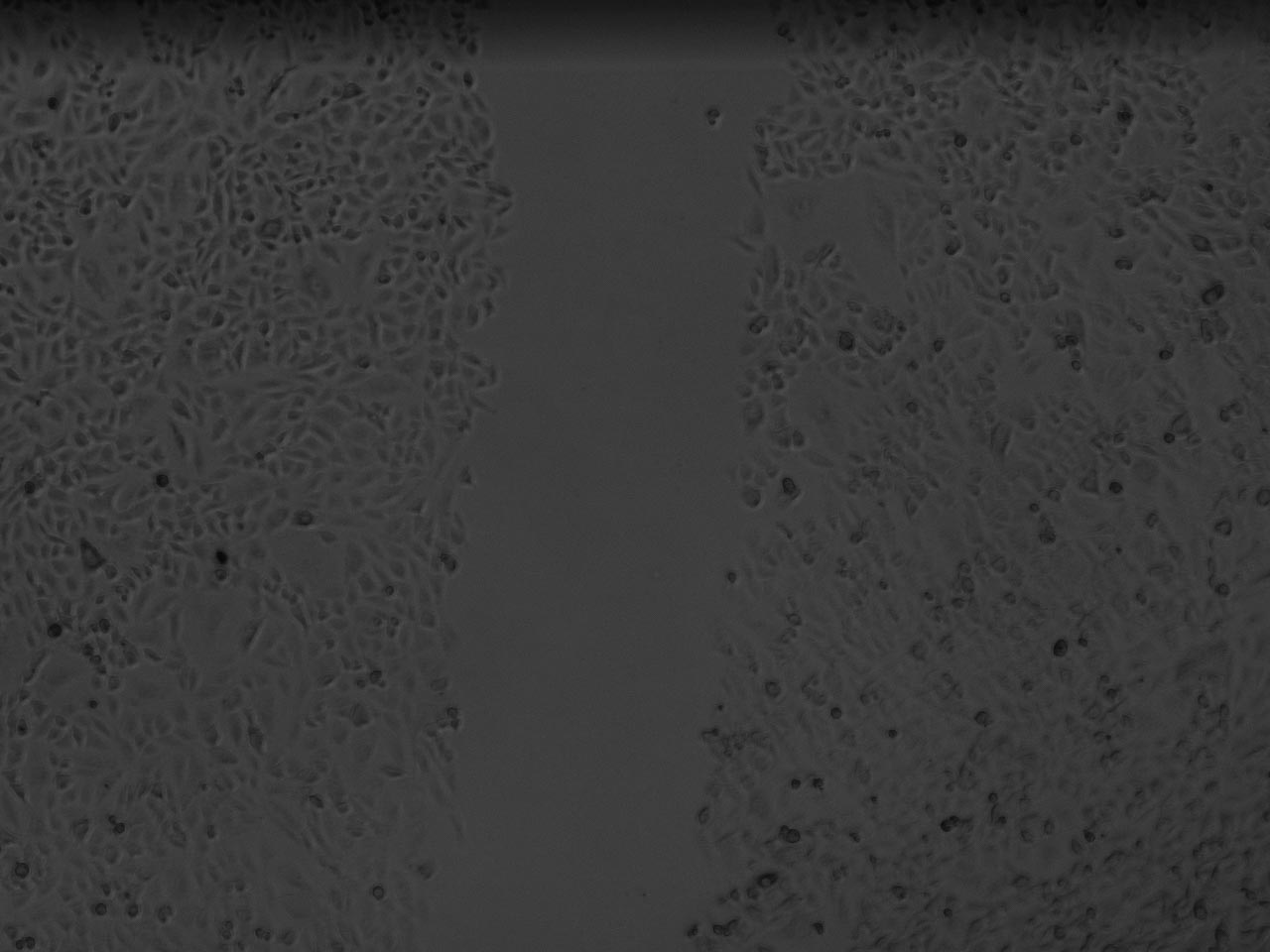

Supplement: Supplemental Information 14 [file peerj-12-18497-s014.zip › qbc939 functional experiment/control overexpression (nc oe)/QBC939 Wound Healing NC OE/picture/k2 r nc 0h.jpg]

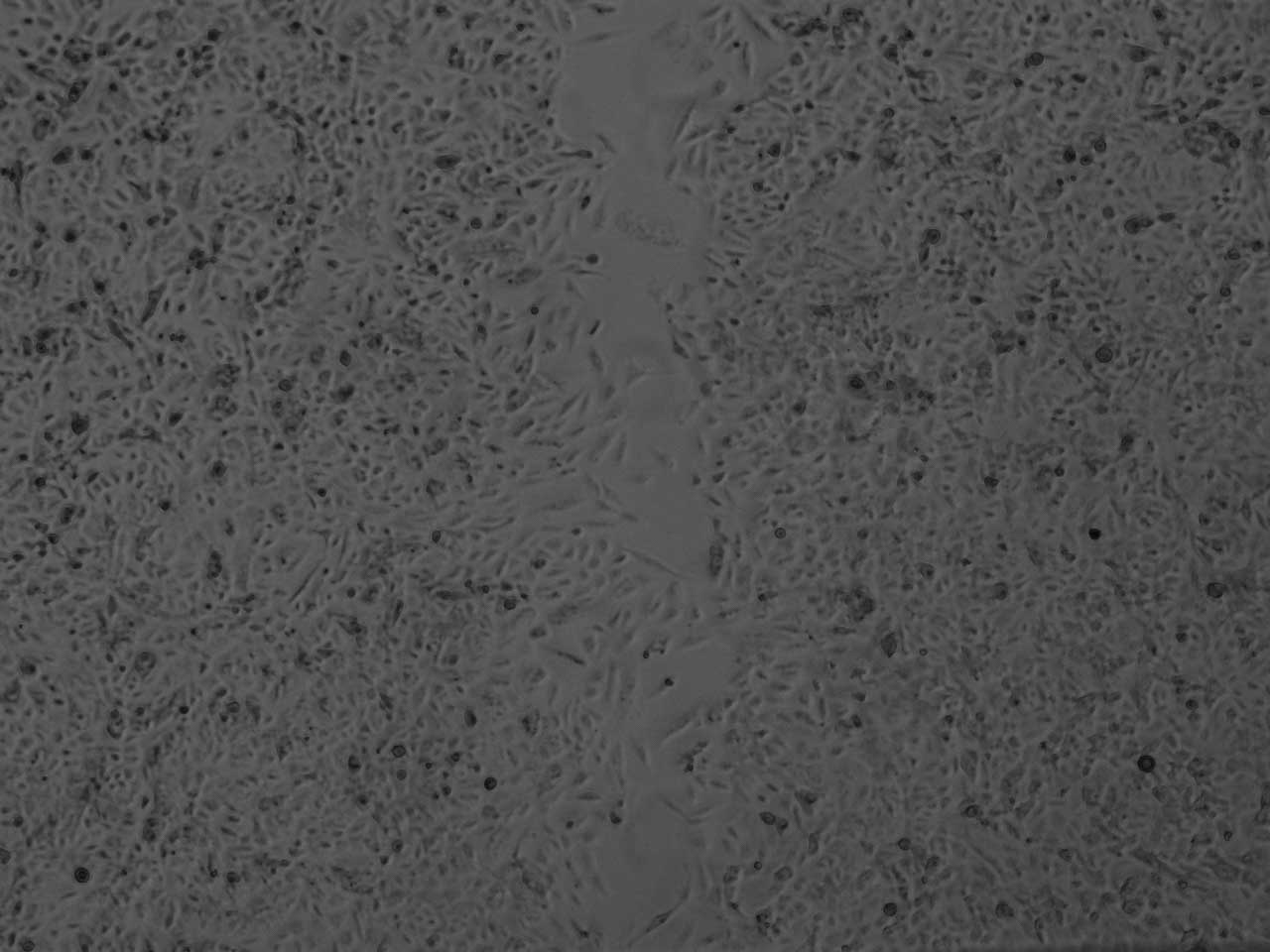

Supplement: Supplemental Information 14 [file peerj-12-18497-s014.zip › qbc939 functional experiment/control overexpression (nc oe)/QBC939 Wound Healing NC OE/picture/k2 q nc 24h.jpg]

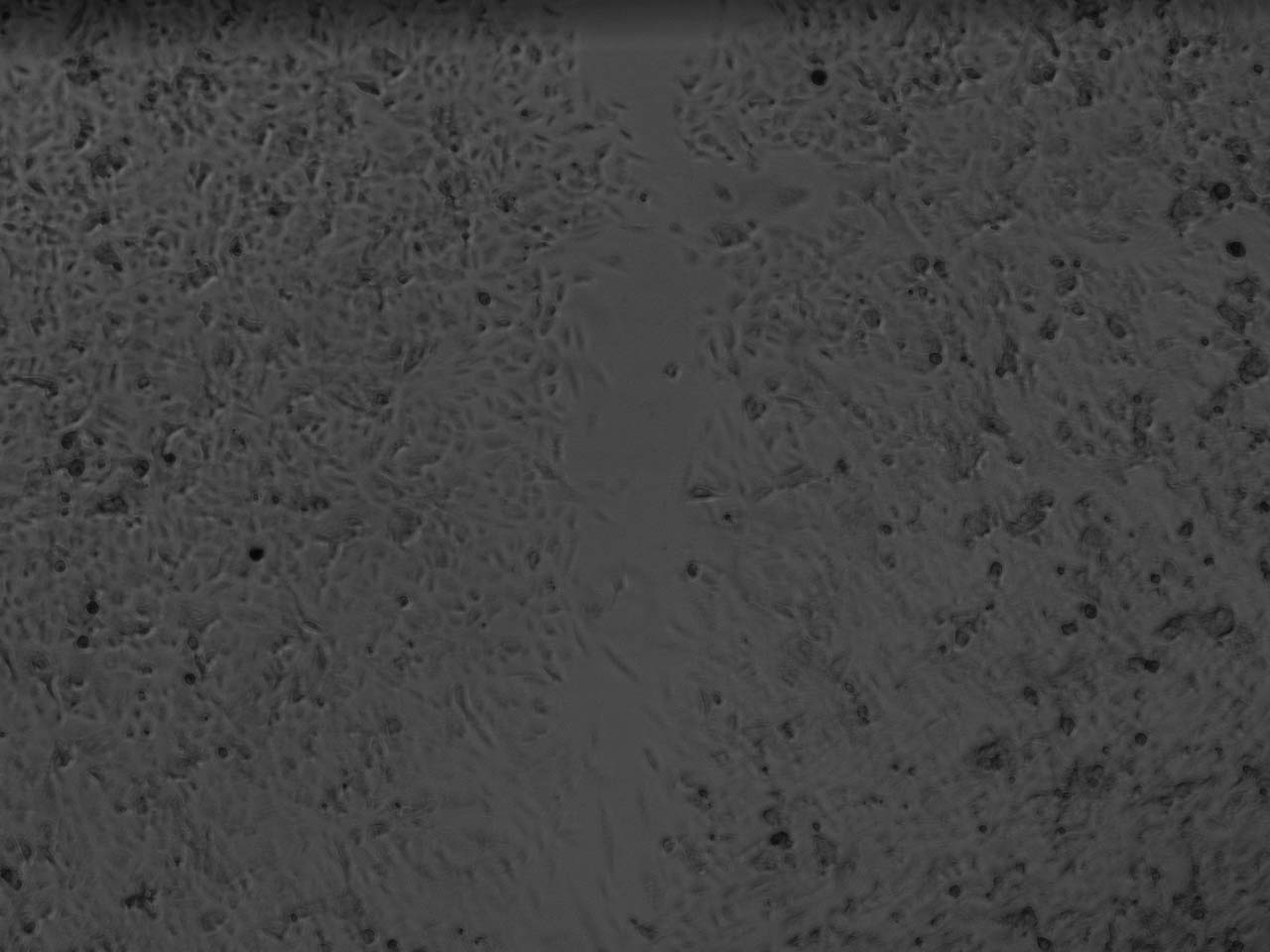

Supplement: Supplemental Information 14 [file peerj-12-18497-s014.zip › qbc939 functional experiment/control overexpression (nc oe)/QBC939 Wound Healing NC OE/picture/k2 r nc 24h.jpg]

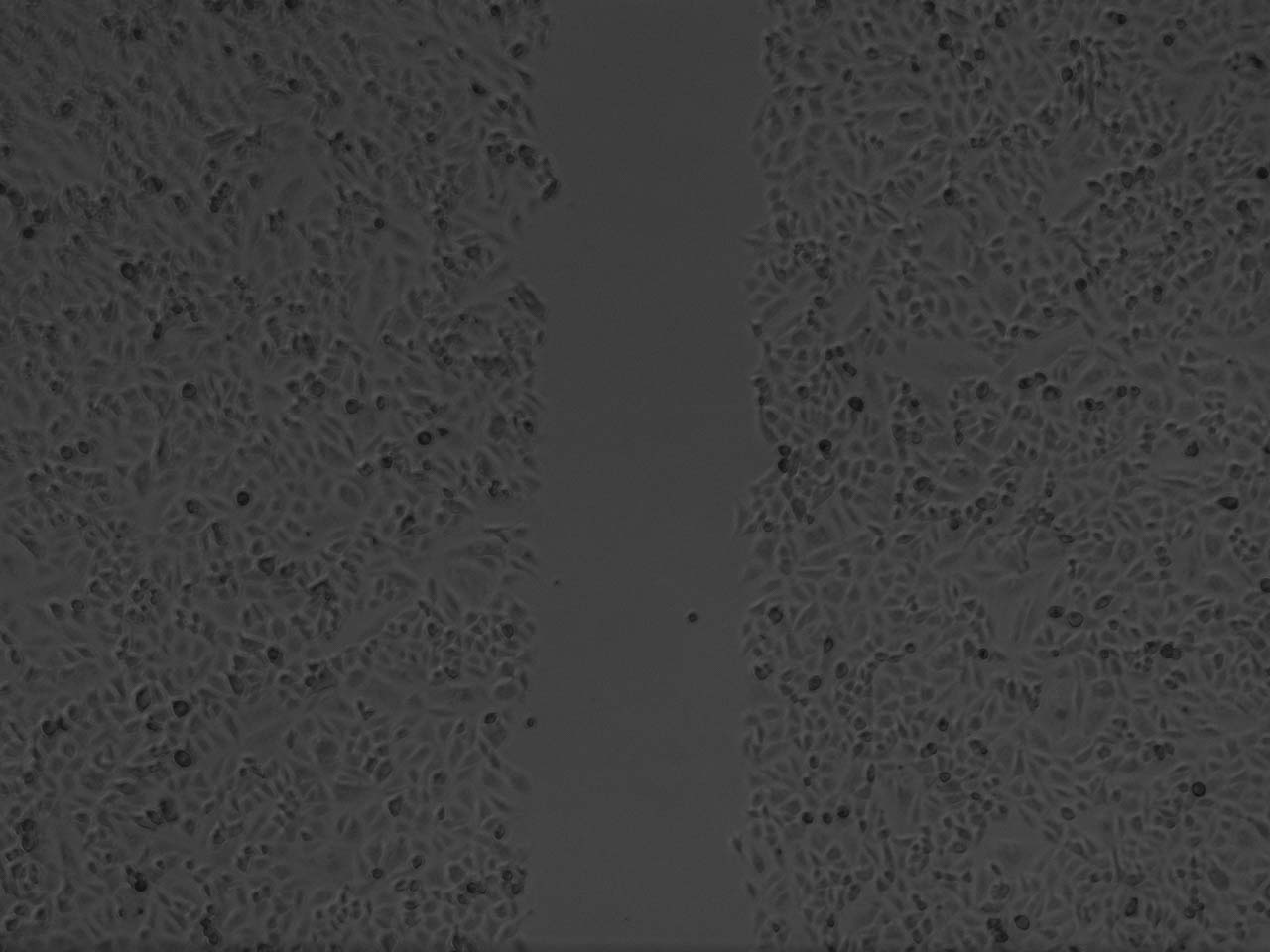

Supplement: Supplemental Information 14 [file peerj-12-18497-s014.zip › qbc939 functional experiment/control overexpression (nc oe)/QBC939 Wound Healing NC OE/picture/k3 a nc 0h.jpg]

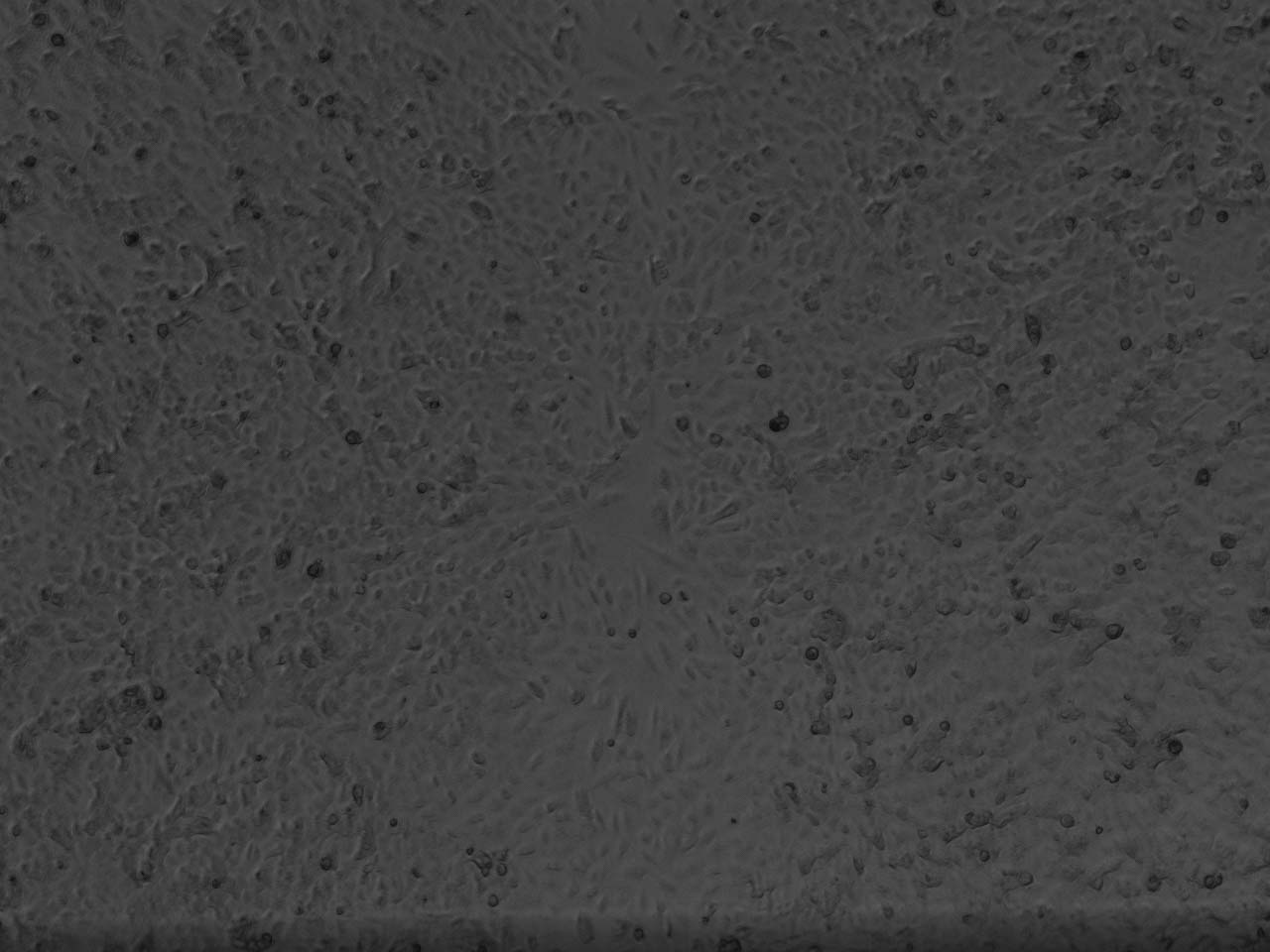

Supplement: Supplemental Information 14 [file peerj-12-18497-s014.zip › qbc939 functional experiment/control overexpression (nc oe)/QBC939 Wound Healing NC OE/picture/k3 a nc 24h.jpg]

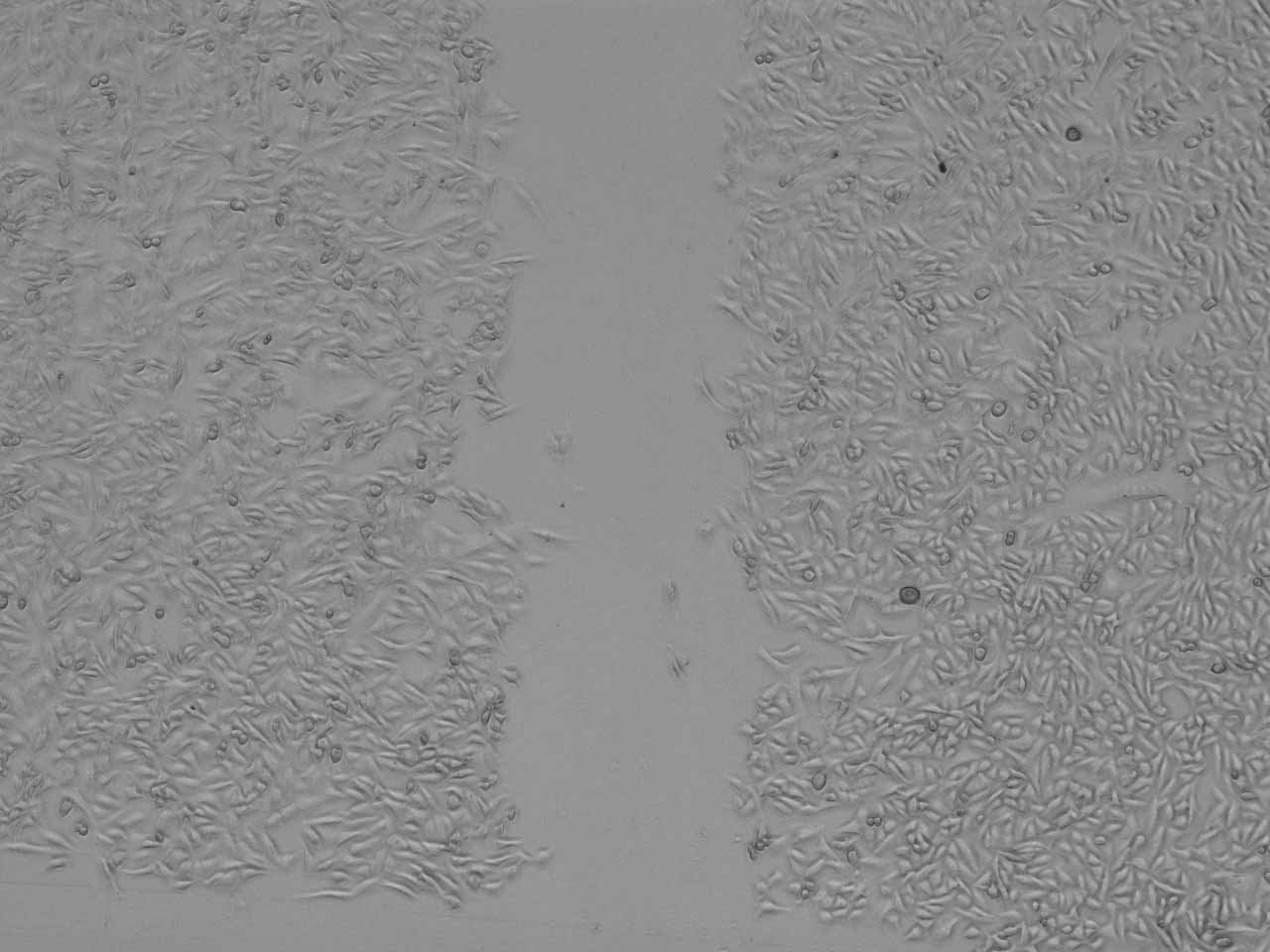

Supplement: Supplemental Information 14 [file peerj-12-18497-s014.zip › qbc939 functional experiment/nc knockdown /qbc 939 Wound Healing nc si/picture/nc 0h1_.jpg]

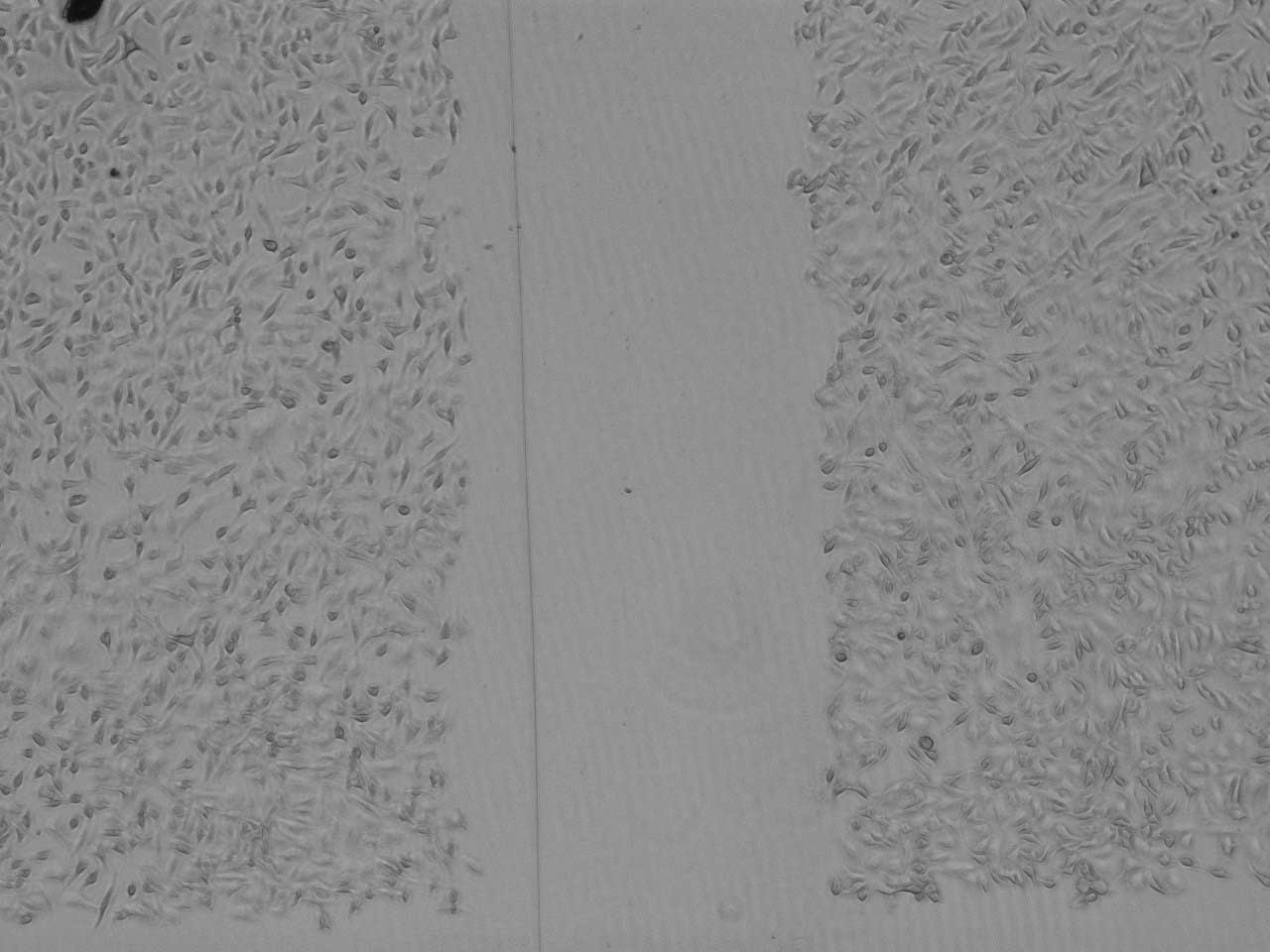

Supplement: Supplemental Information 14 [file peerj-12-18497-s014.zip › qbc939 functional experiment/nc knockdown /qbc 939 Wound Healing nc si/picture/nc 0h2.jpg]

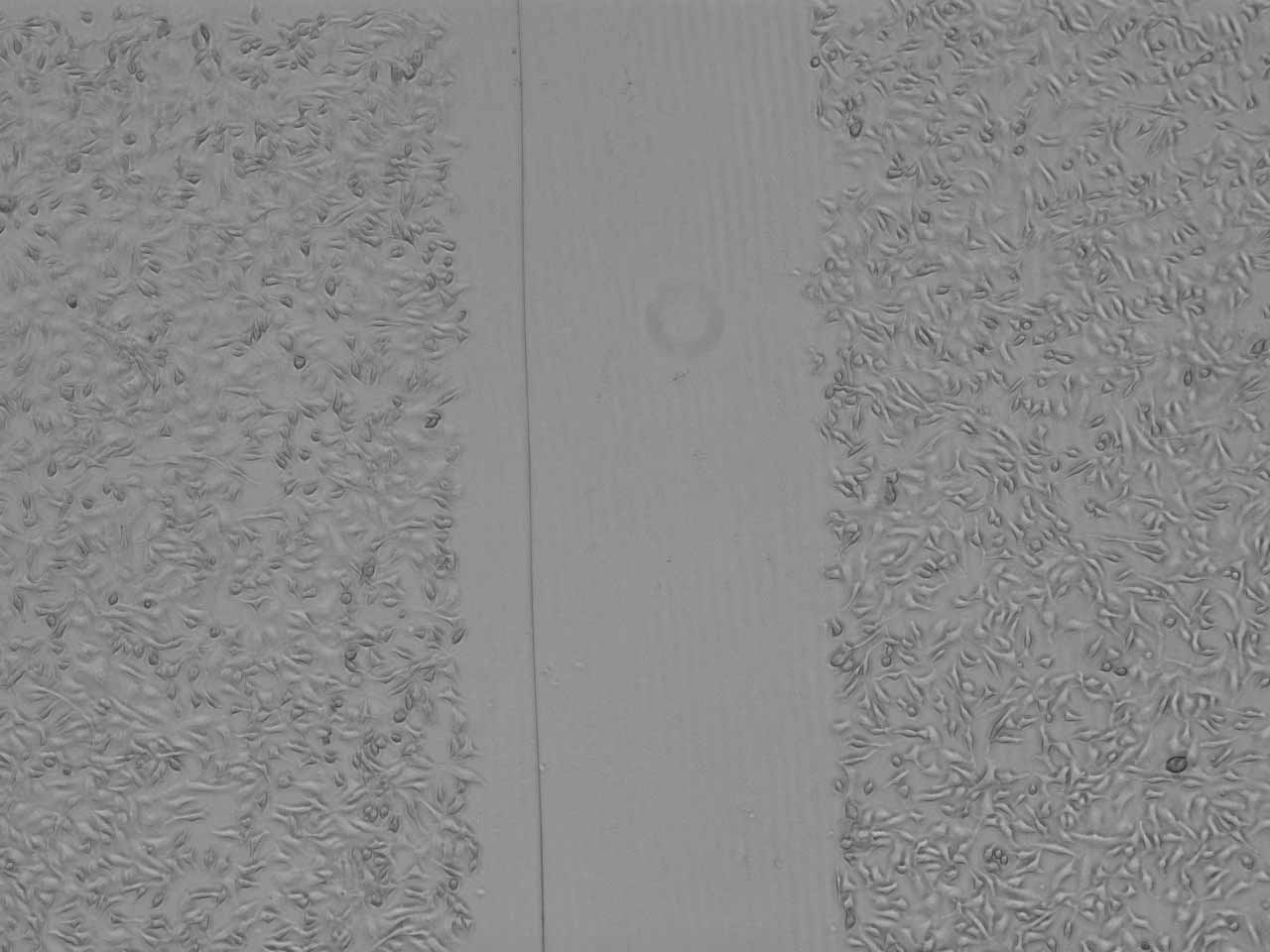

Supplement: Supplemental Information 14 [file peerj-12-18497-s014.zip › qbc939 functional experiment/nc knockdown /qbc 939 Wound Healing nc si/picture/nc 0h3.jpg]

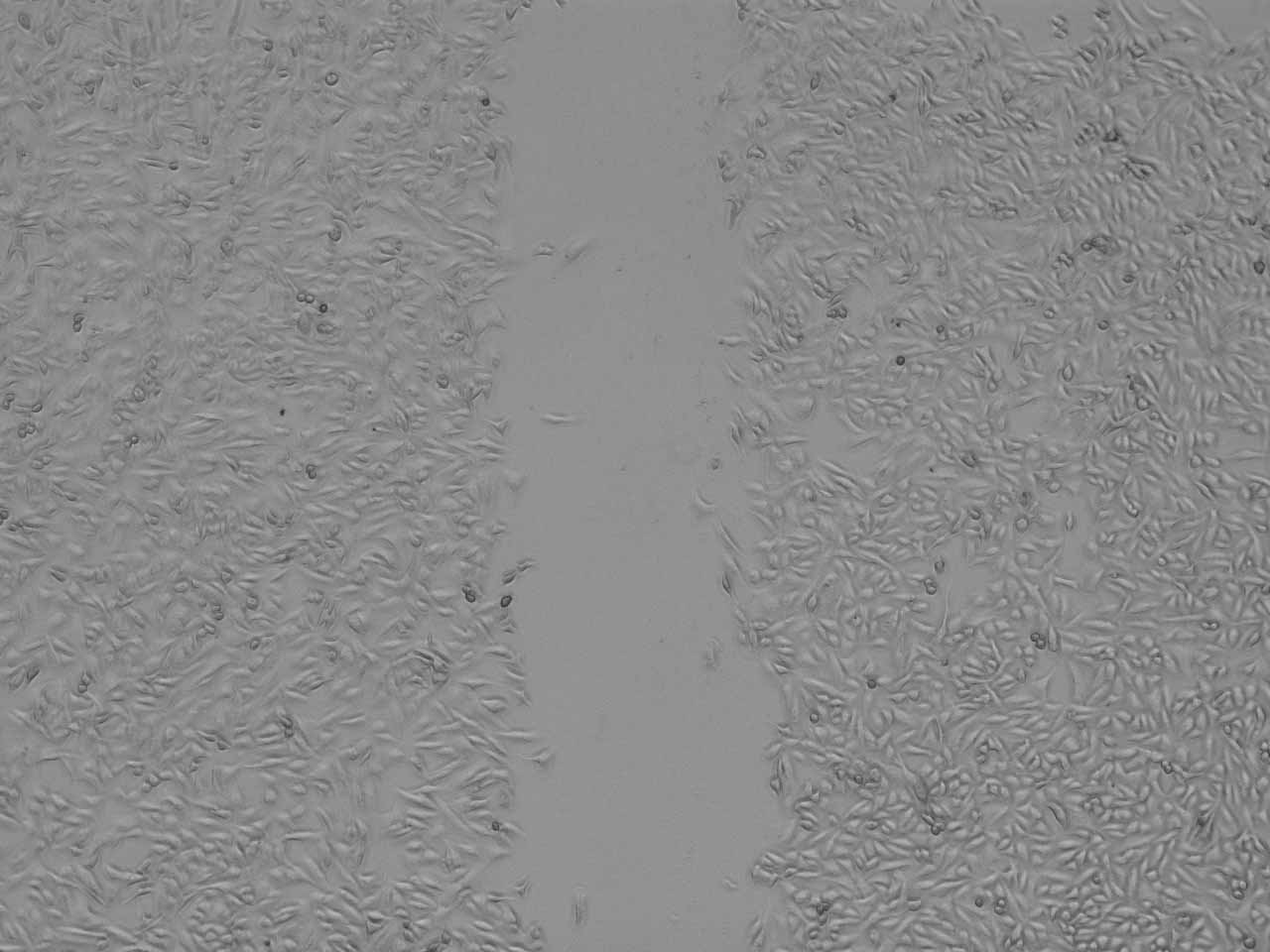

Supplement: Supplemental Information 14 [file peerj-12-18497-s014.zip › qbc939 functional experiment/nc knockdown /qbc 939 Wound Healing nc si/picture/nc 24h1_.jpg]

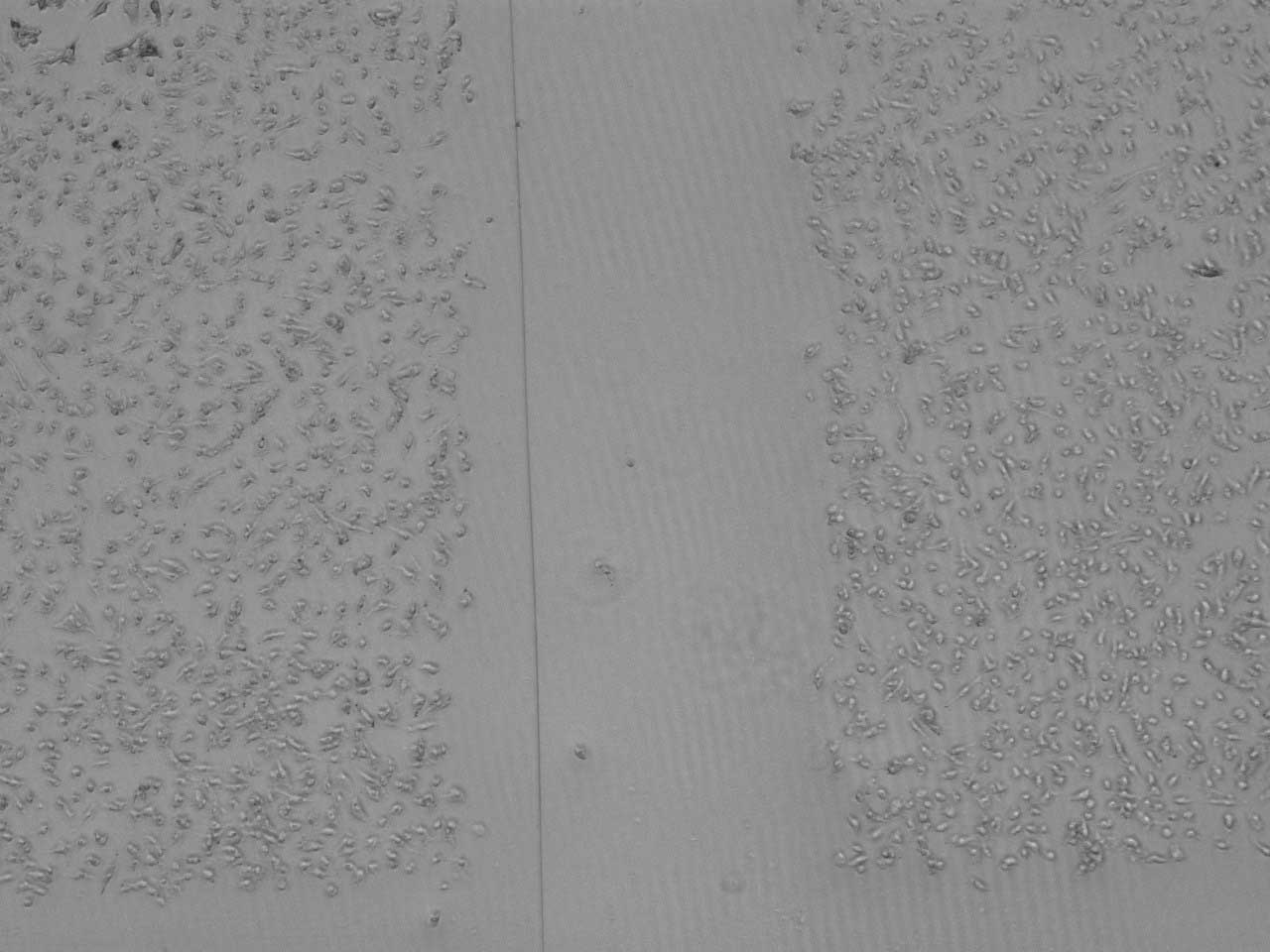

Supplement: Supplemental Information 14 [file peerj-12-18497-s014.zip › qbc939 functional experiment/nc knockdown /qbc 939 Wound Healing nc si/picture/nc 24h2.jpg]

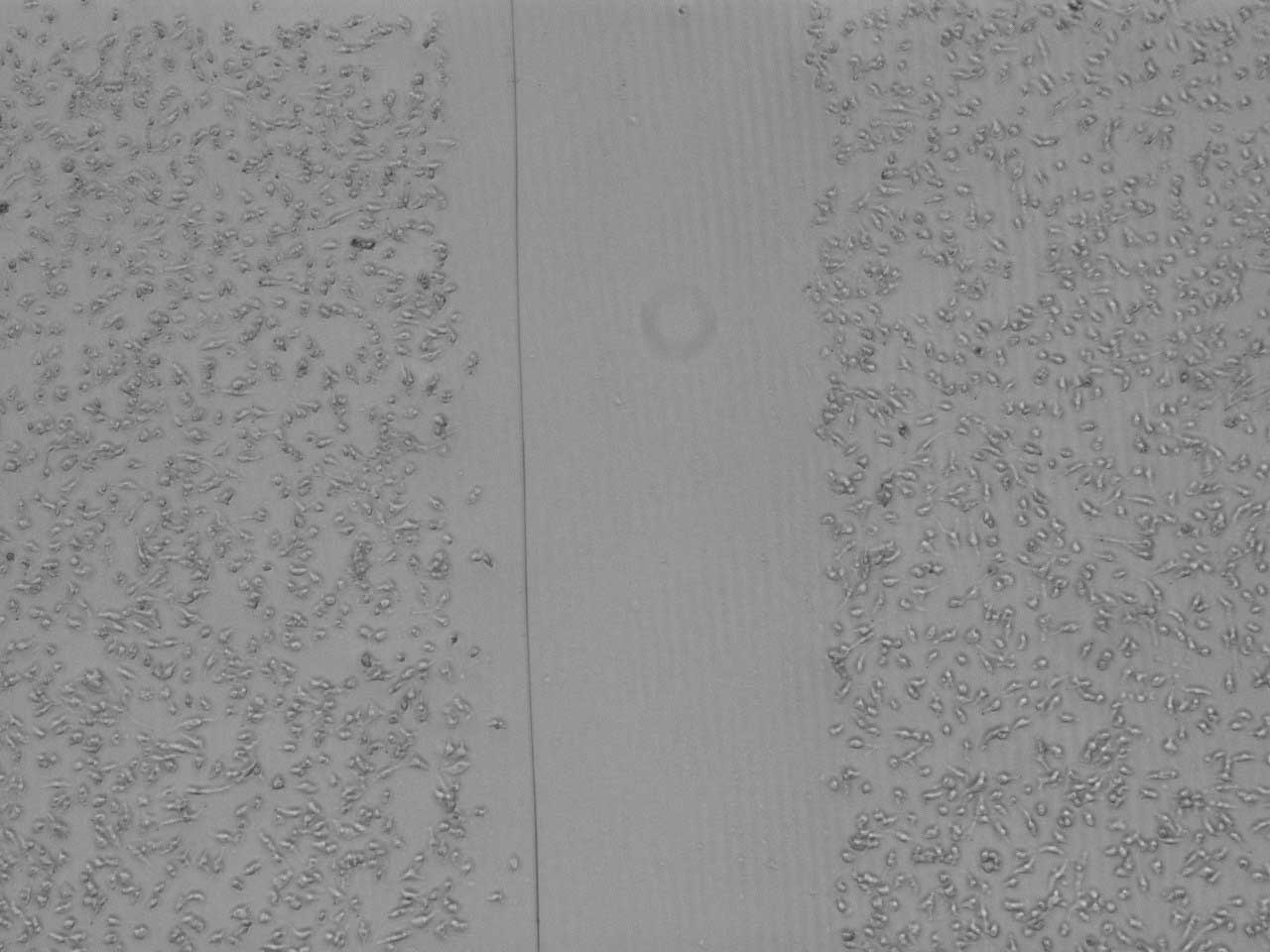

Supplement: Supplemental Information 14 [file peerj-12-18497-s014.zip › qbc939 functional experiment/nc knockdown /qbc 939 Wound Healing nc si/picture/nc 24h3.jpg]

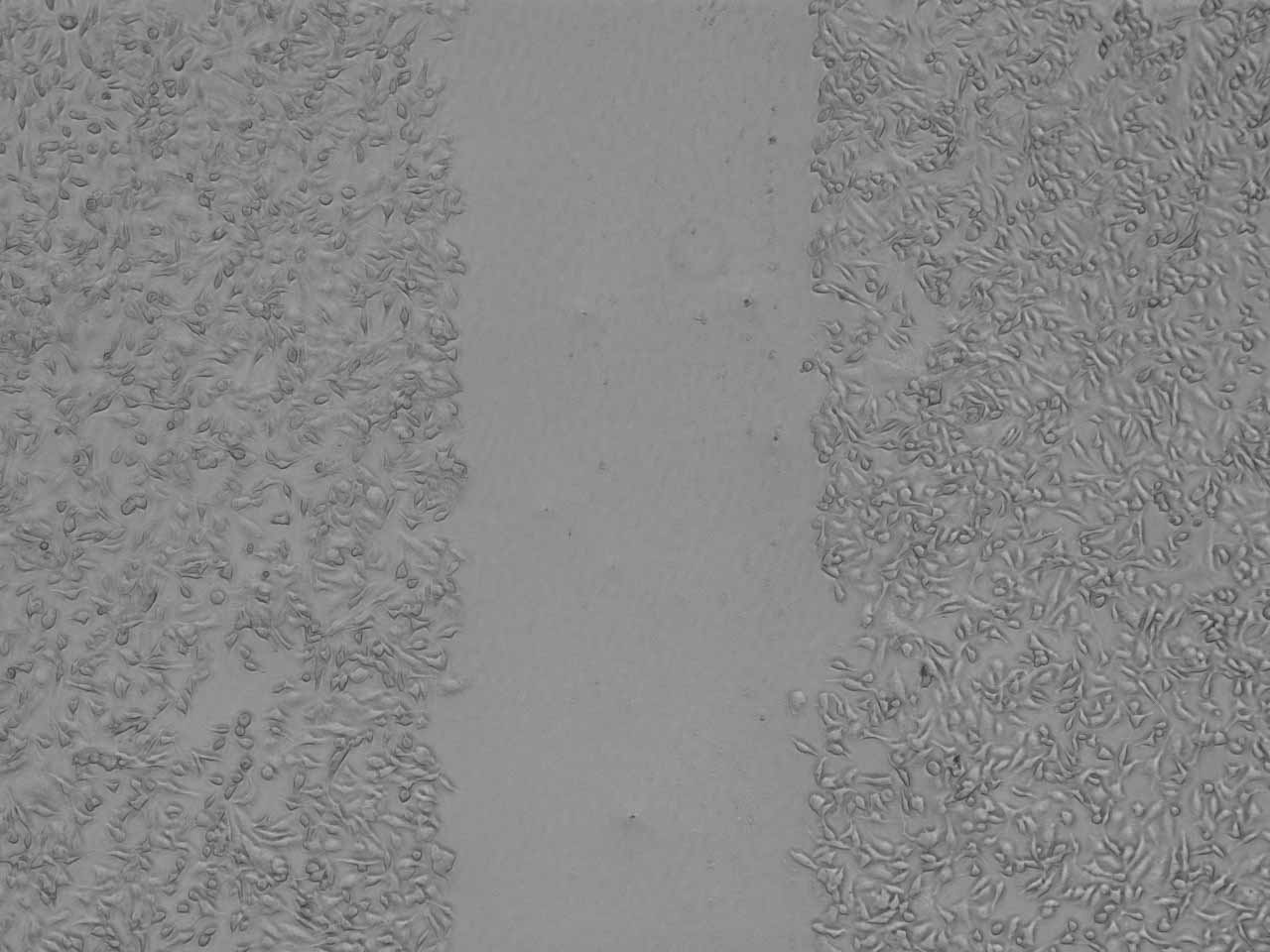

Supplement: Supplemental Information 14 [file peerj-12-18497-s014.zip › qbc939 functional experiment/nc knockdown /qbc 939 Wound Healing nc si/picture/si 0h a.jpg]

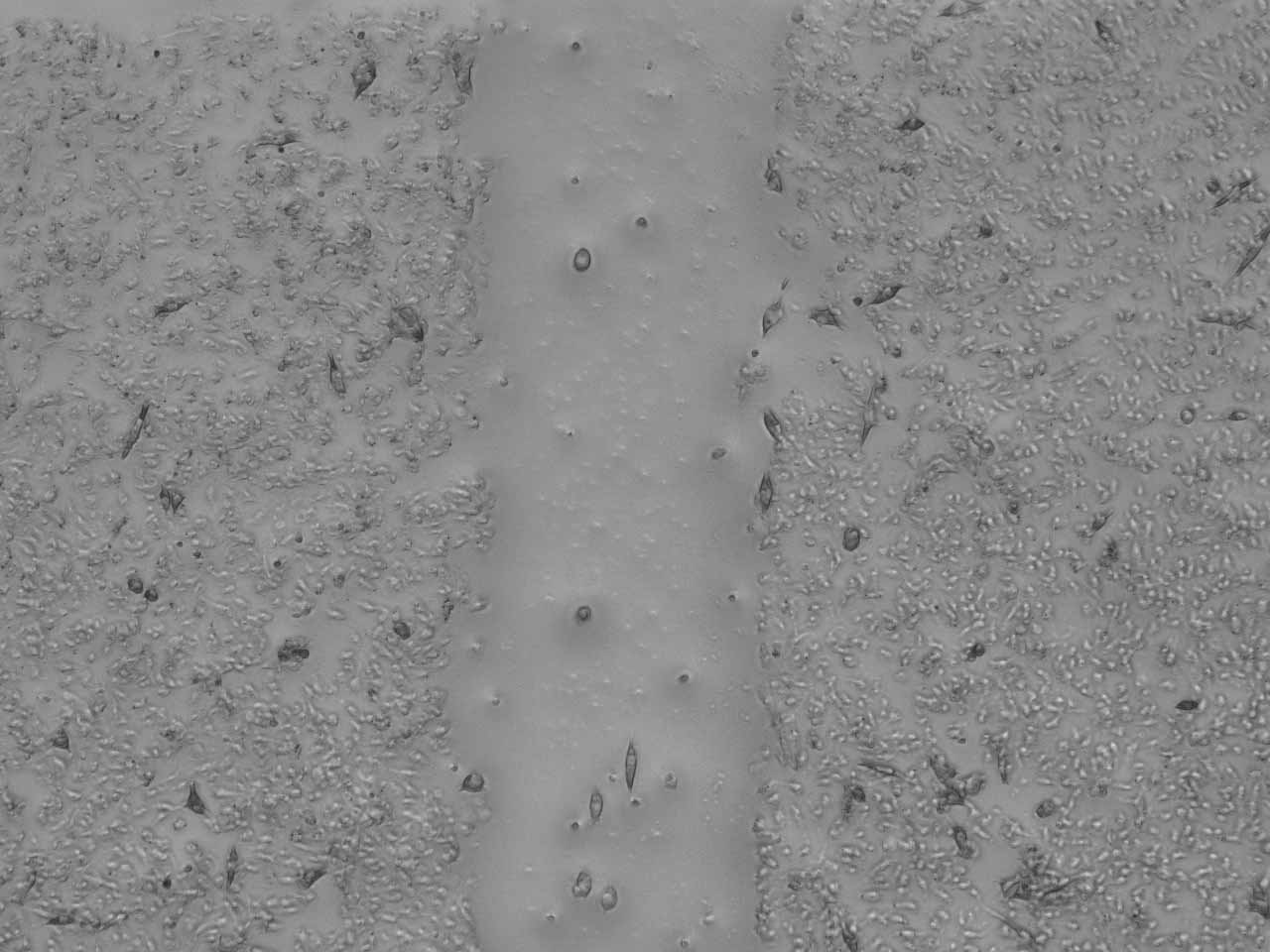

Supplement: Supplemental Information 14 [file peerj-12-18497-s014.zip › qbc939 functional experiment/nc knockdown /qbc 939 Wound Healing nc si/picture/si 24h a.jpg]

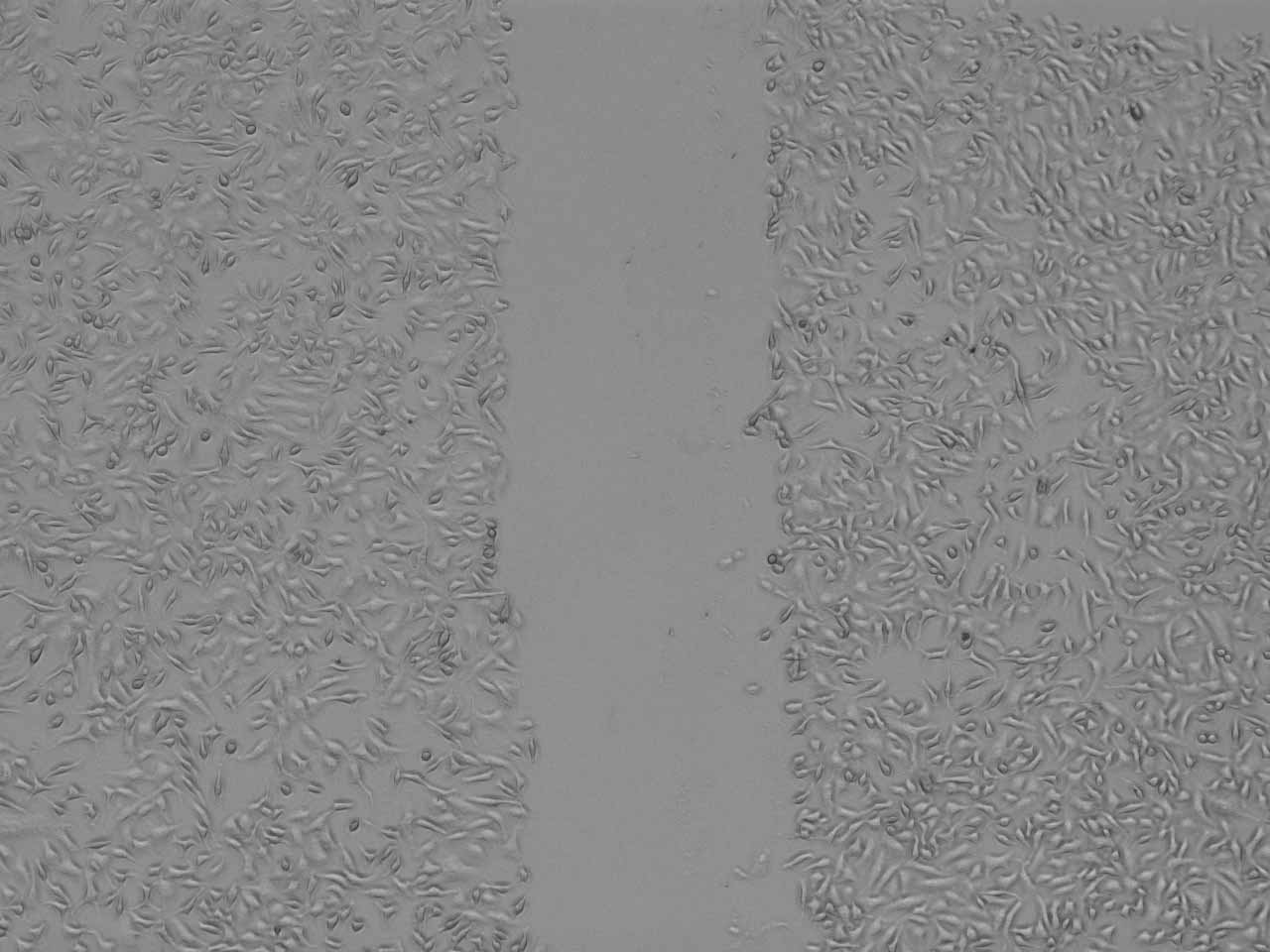

Supplement: Supplemental Information 14 [file peerj-12-18497-s014.zip › qbc939 functional experiment/nc knockdown /qbc 939 Wound Healing nc si/picture/si 0h b.jpg]

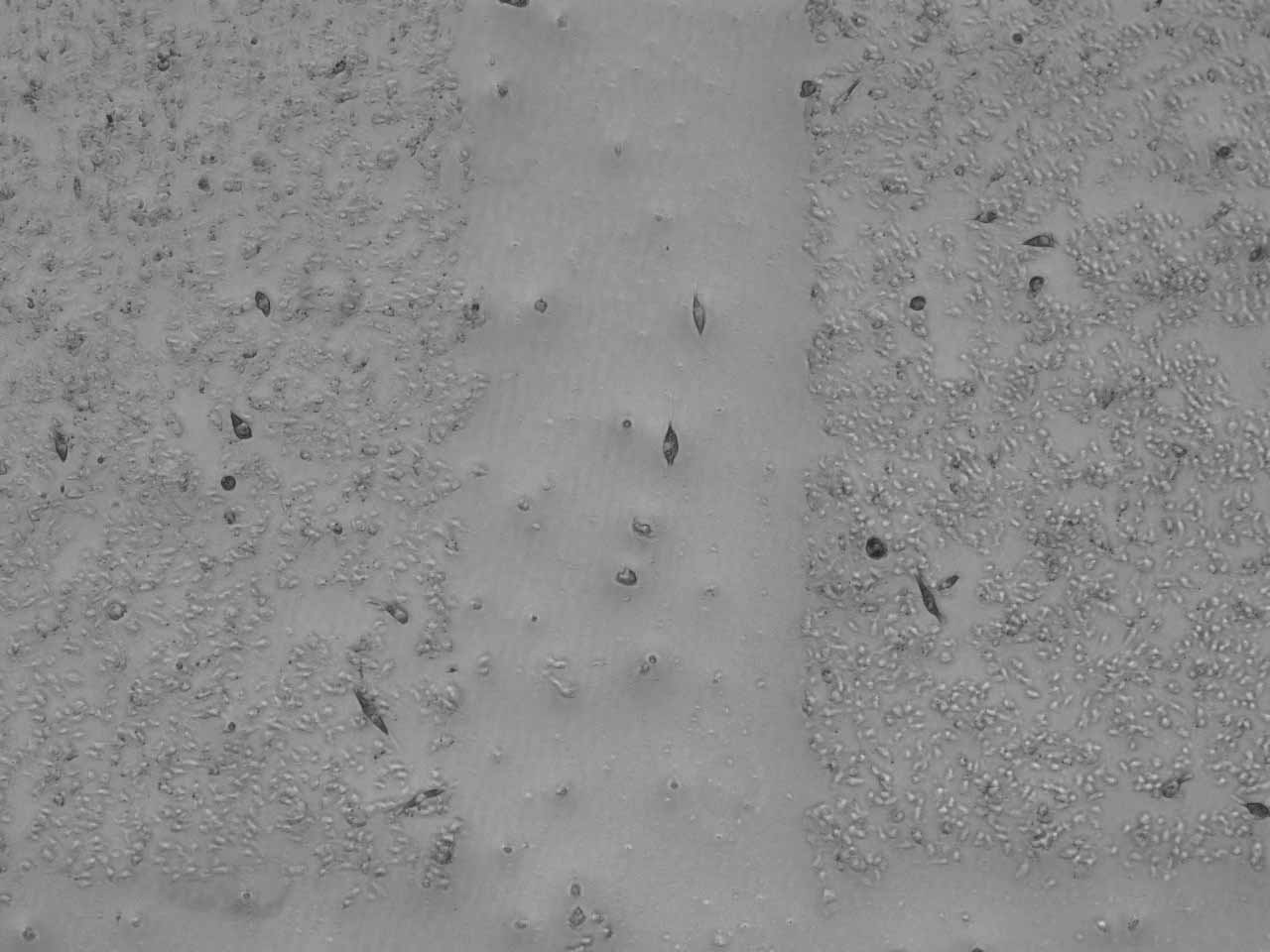

Supplement: Supplemental Information 14 [file peerj-12-18497-s014.zip › qbc939 functional experiment/nc knockdown /qbc 939 Wound Healing nc si/picture/si 0h c.jpg]

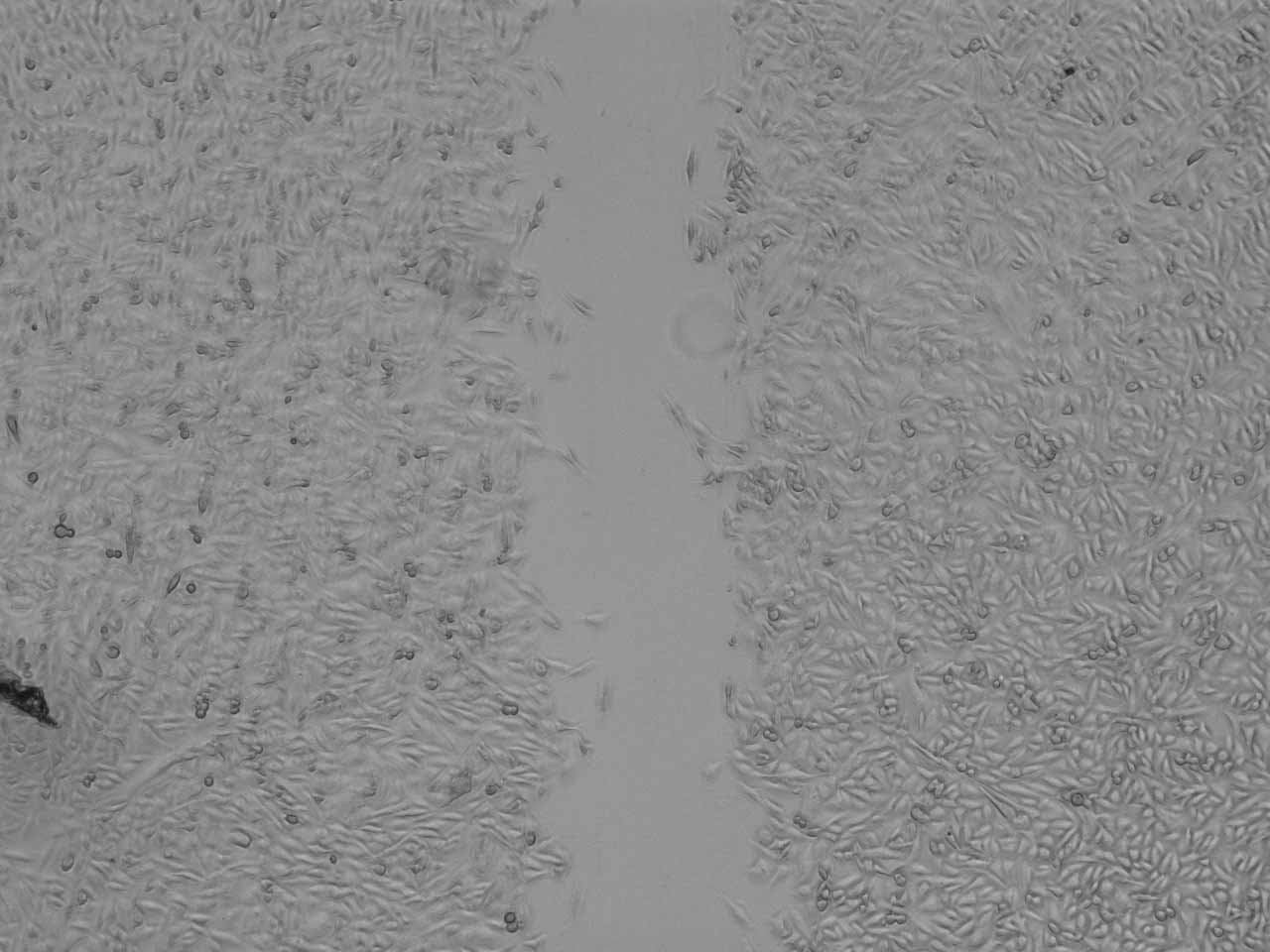

Supplement: Supplemental Information 14 [file peerj-12-18497-s014.zip › qbc939 functional experiment/nc knockdown /qbc 939 Wound Healing nc si/picture/si 24h b.jpg]

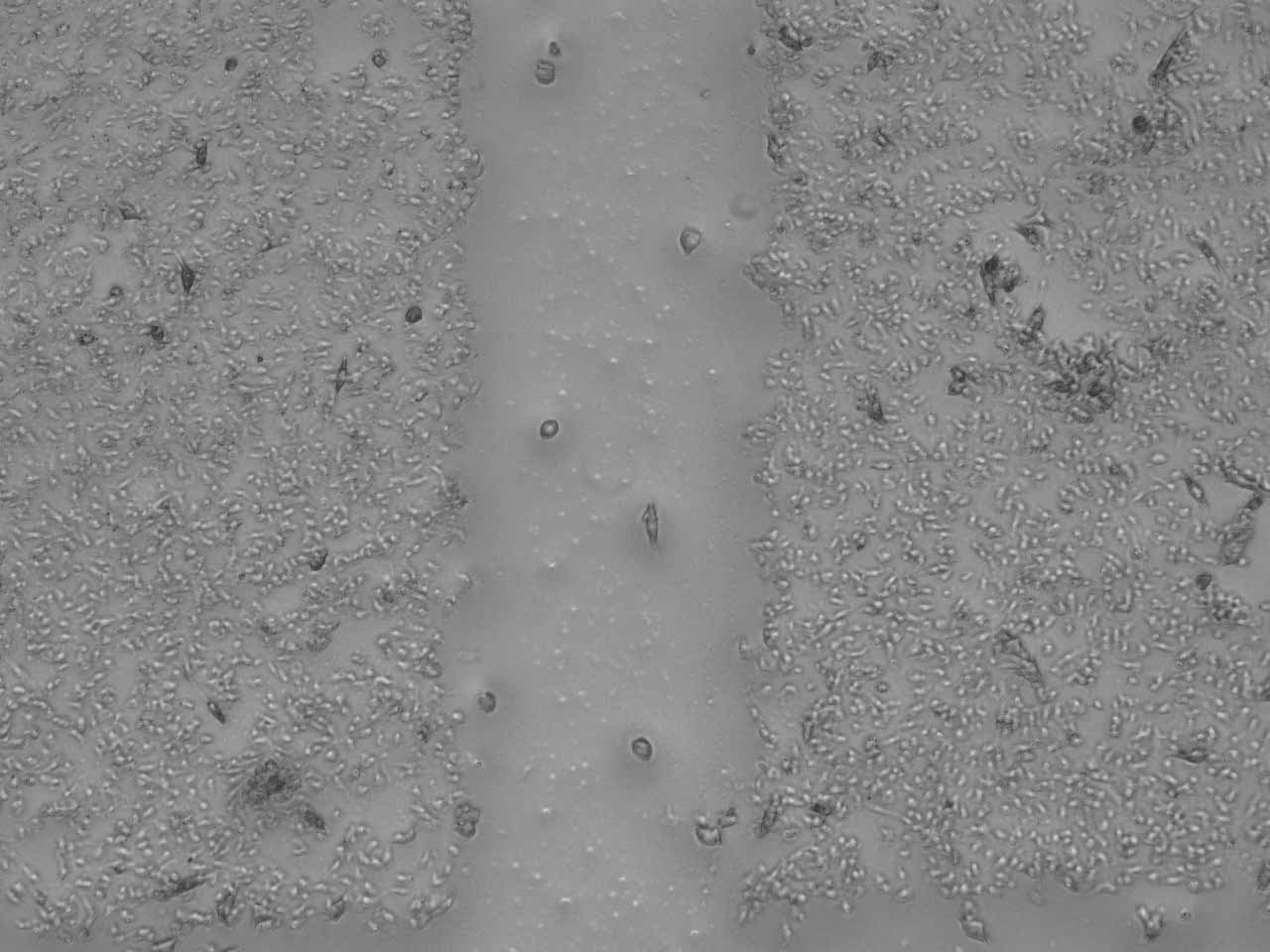

Supplement: Supplemental Information 14 [file peerj-12-18497-s014.zip › qbc939 functional experiment/nc knockdown /qbc 939 Wound Healing nc si/picture/si 24h c.jpg]

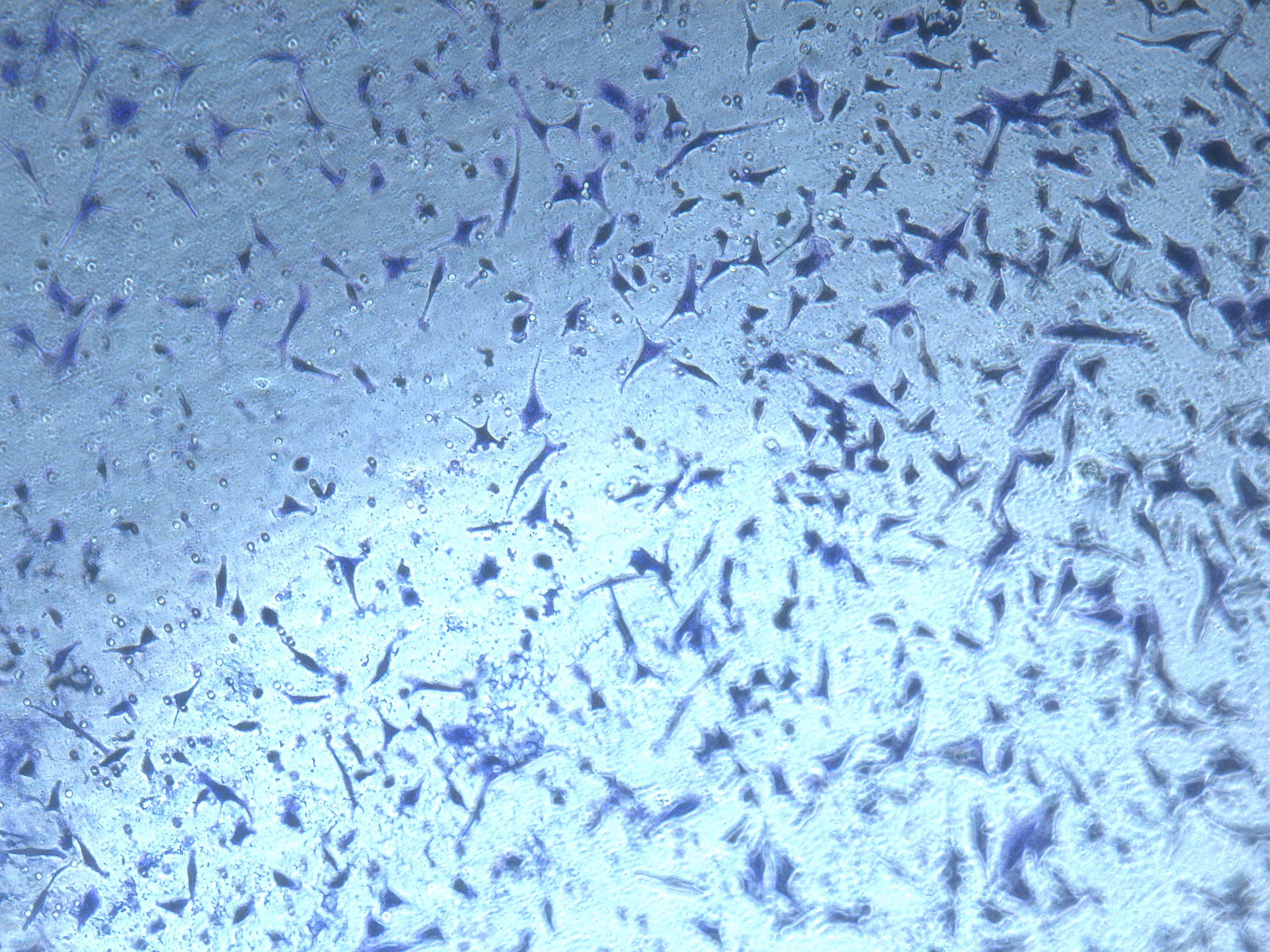

Supplement: Supplemental Information 14 [file peerj-12-18497-s014.zip › qbc939 functional experiment/nc knockdown /qbc Invasion nc si/picture/nc-1.jpg]

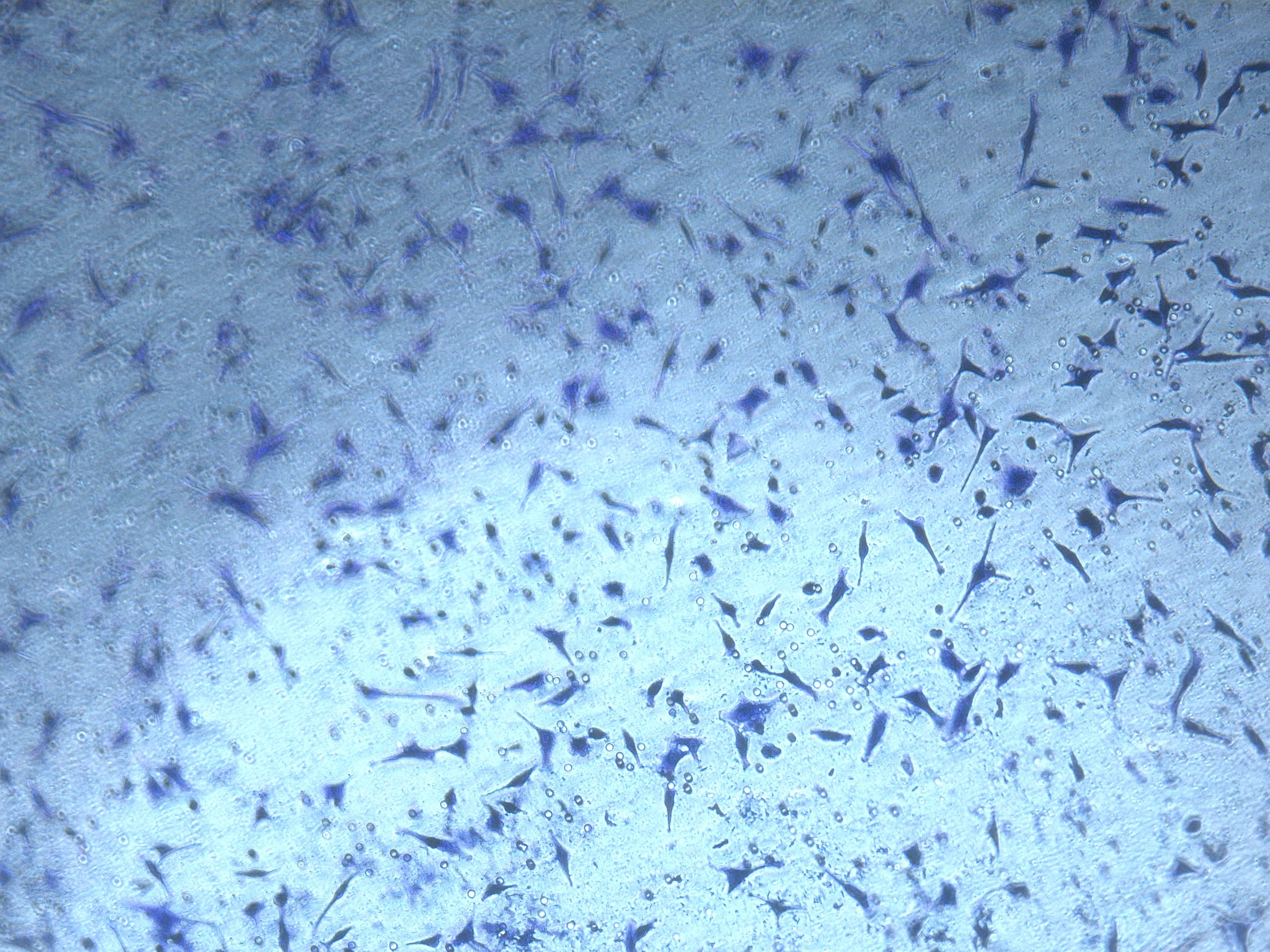

Supplement: Supplemental Information 14 [file peerj-12-18497-s014.zip › qbc939 functional experiment/nc knockdown /qbc Invasion nc si/picture/nc-2.jpg]

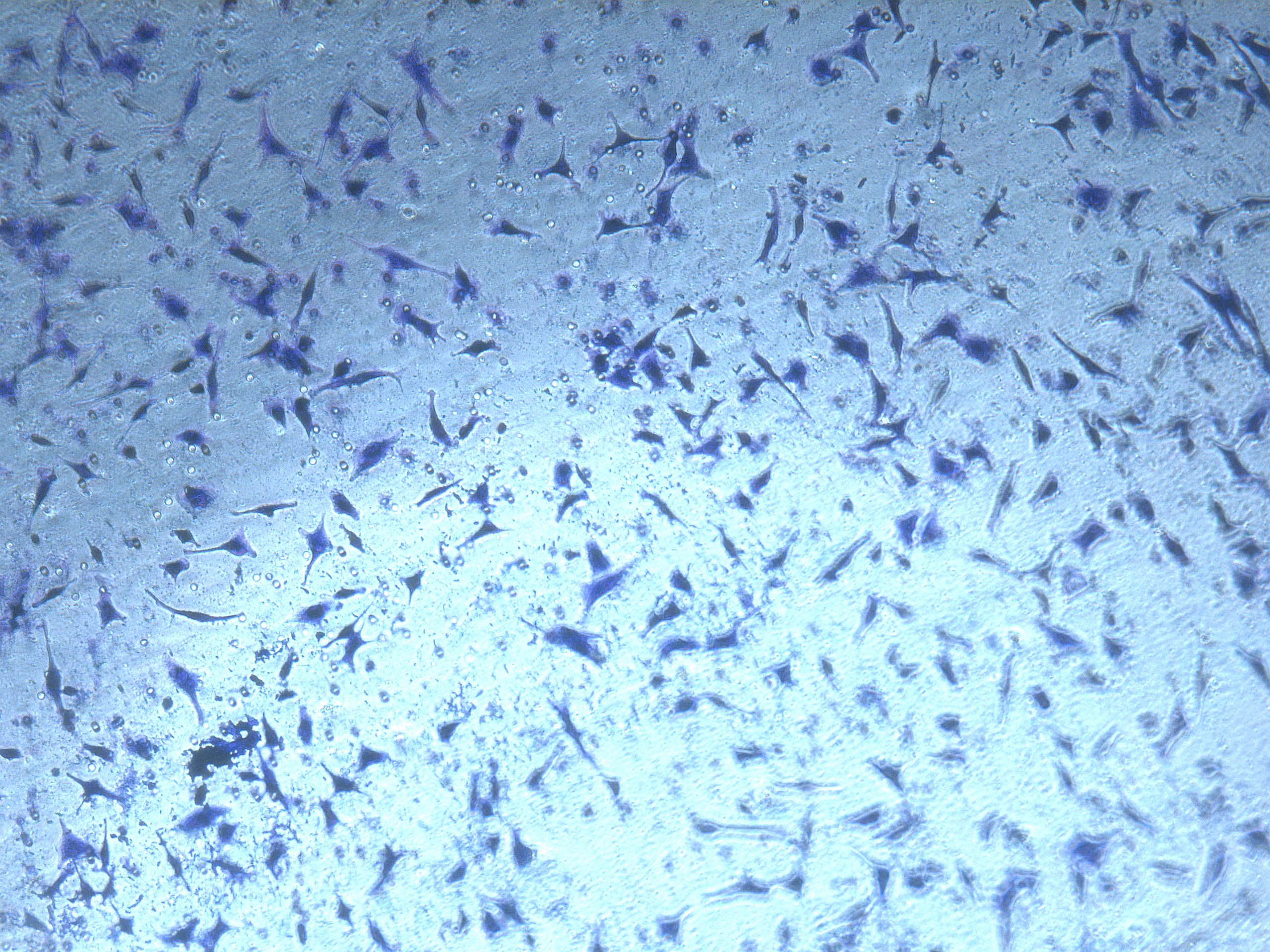

Supplement: Supplemental Information 14 [file peerj-12-18497-s014.zip › qbc939 functional experiment/nc knockdown /qbc Invasion nc si/picture/nc-3.jpg]

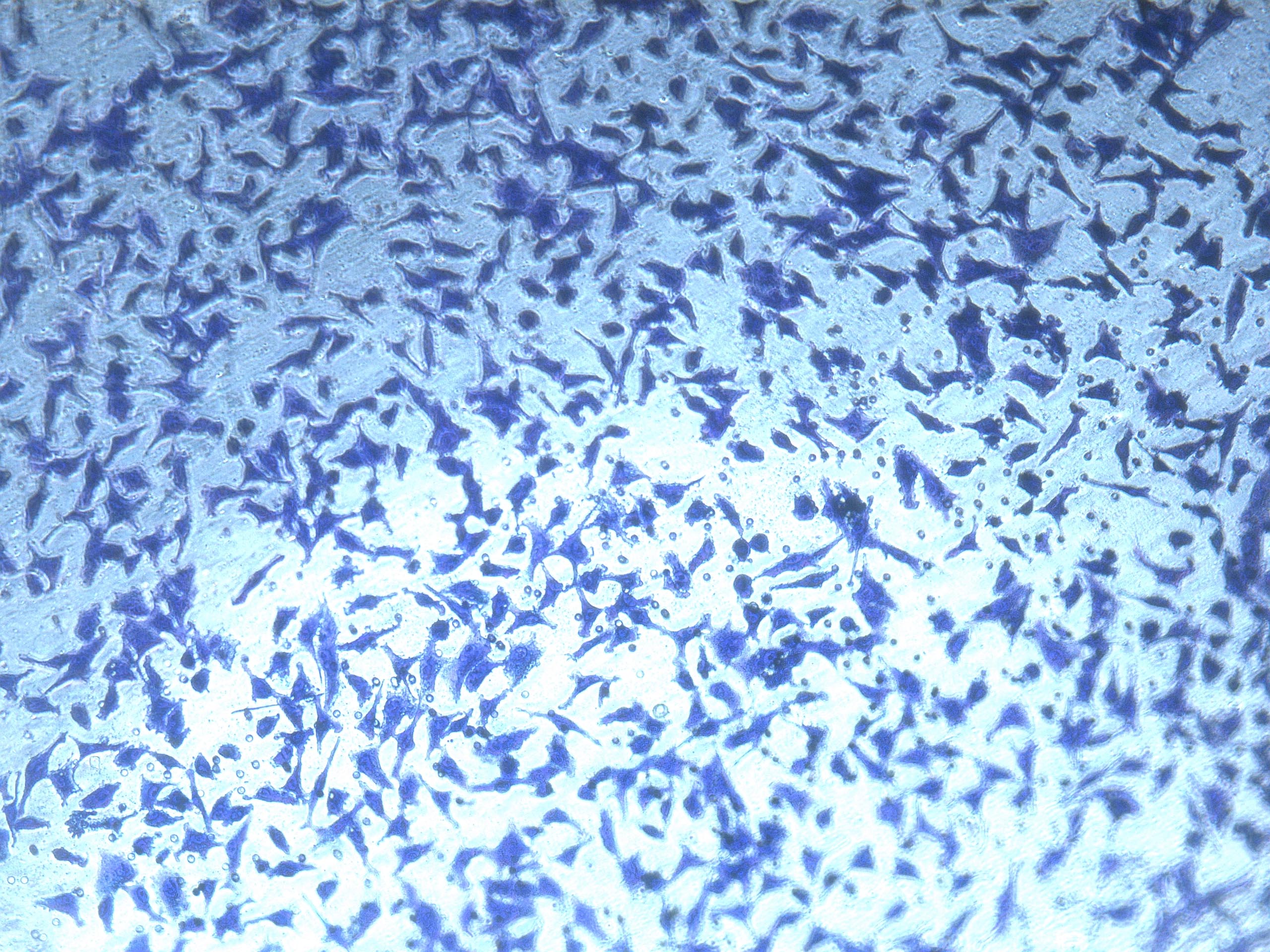

Supplement: Supplemental Information 14 [file peerj-12-18497-s014.zip › qbc939 functional experiment/nc knockdown /qbc Invasion nc si/picture/si-1.jpg]

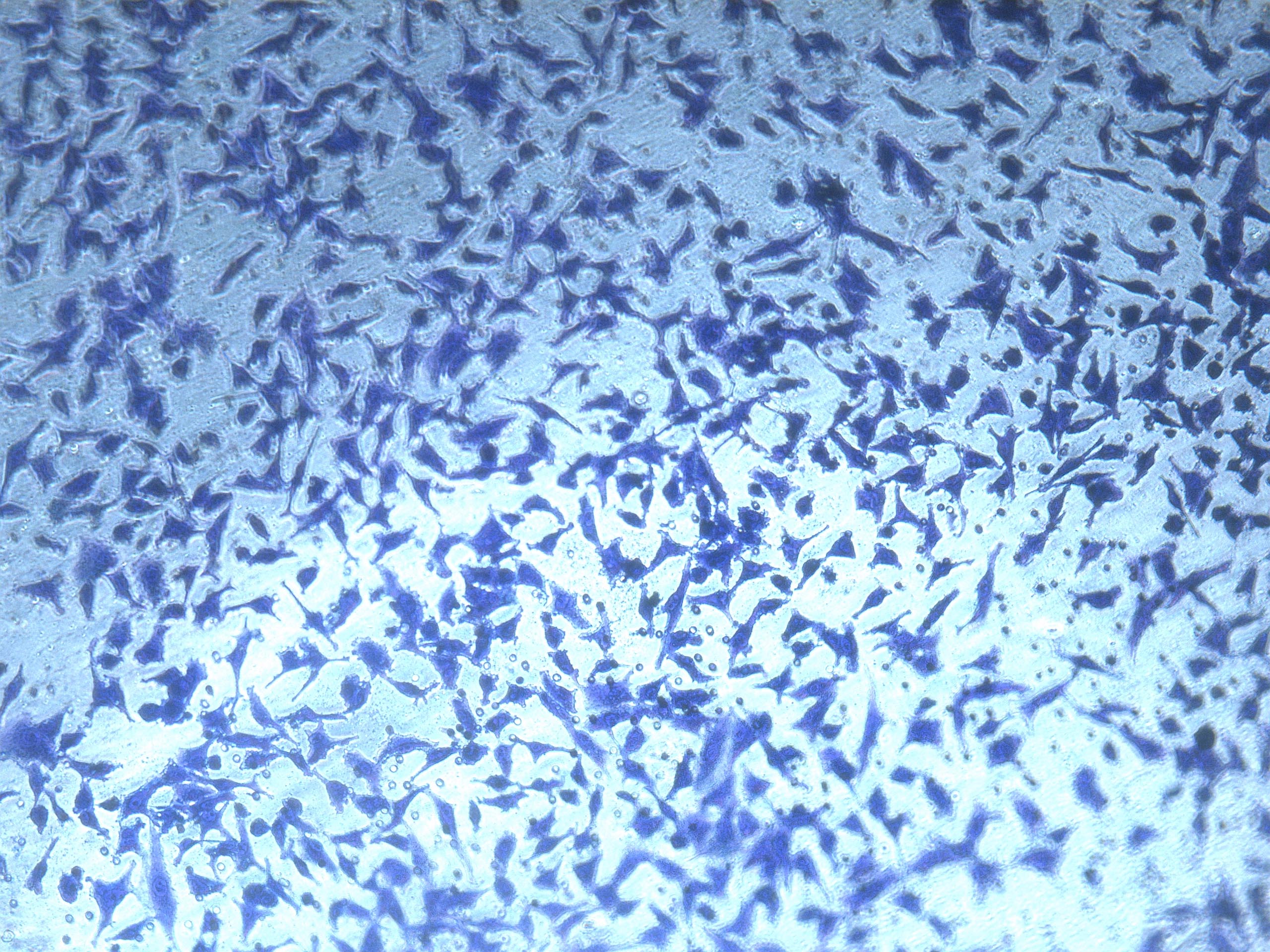

Supplement: Supplemental Information 14 [file peerj-12-18497-s014.zip › qbc939 functional experiment/nc knockdown /qbc Invasion nc si/picture/si-2.jpg]

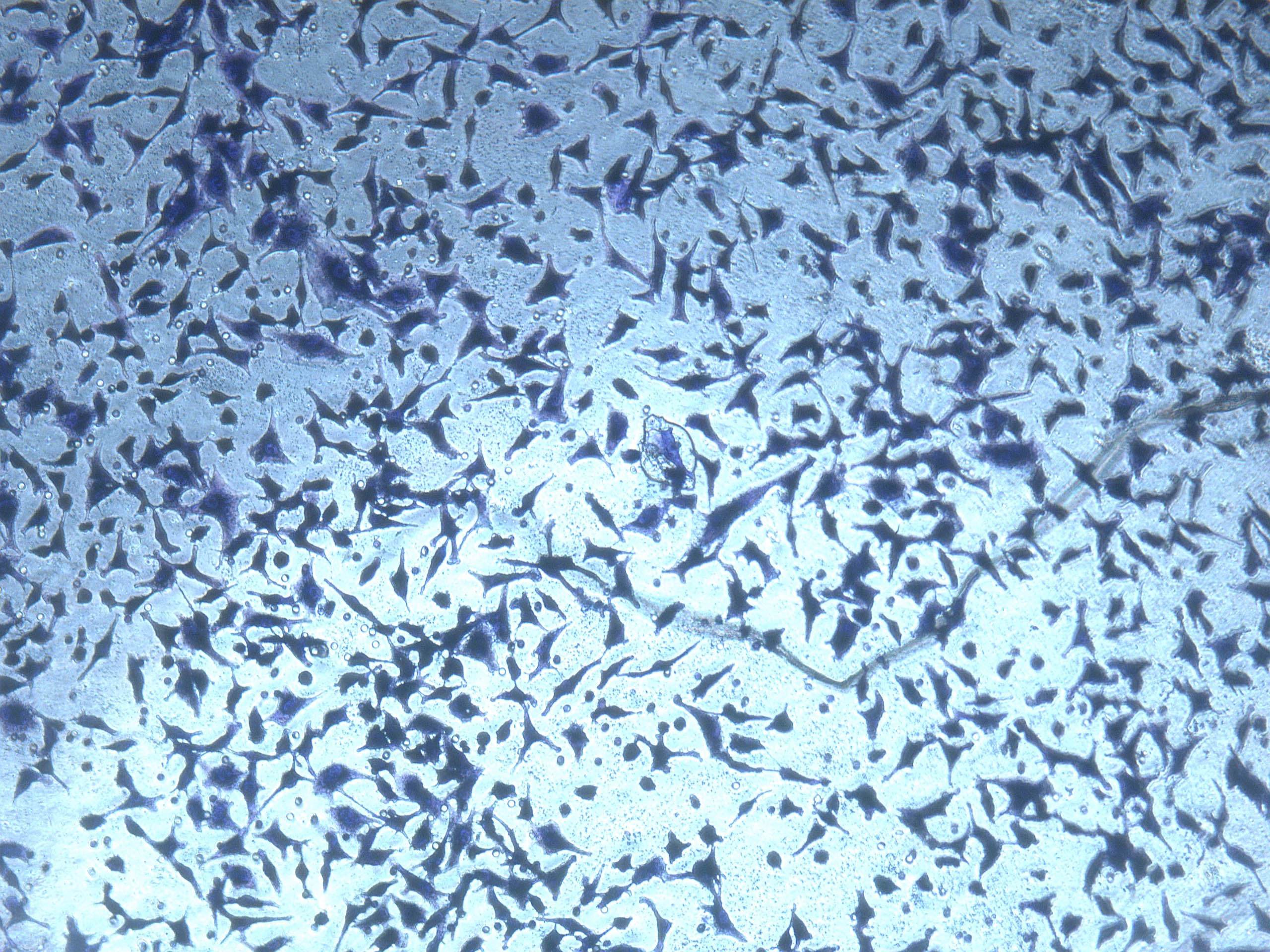

Supplement: Supplemental Information 14 [file peerj-12-18497-s014.zip › qbc939 functional experiment/nc knockdown /qbc Invasion nc si/picture/si-3.jpg]

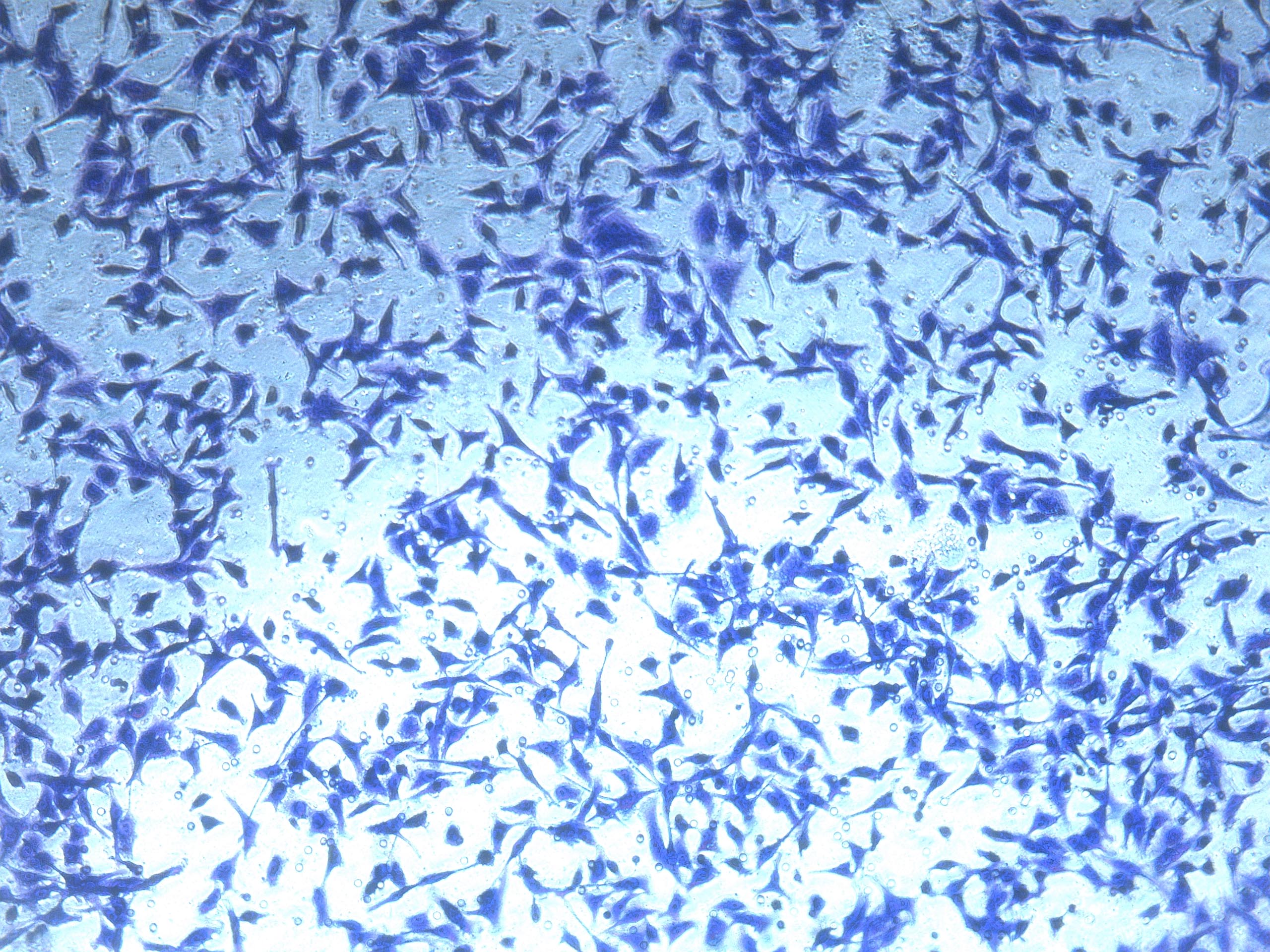

Supplement: Supplemental Information 14 [file peerj-12-18497-s014.zip › qbc939 functional experiment/nc knockdown /qbc migration nc si/picture/nc-1.jpg]

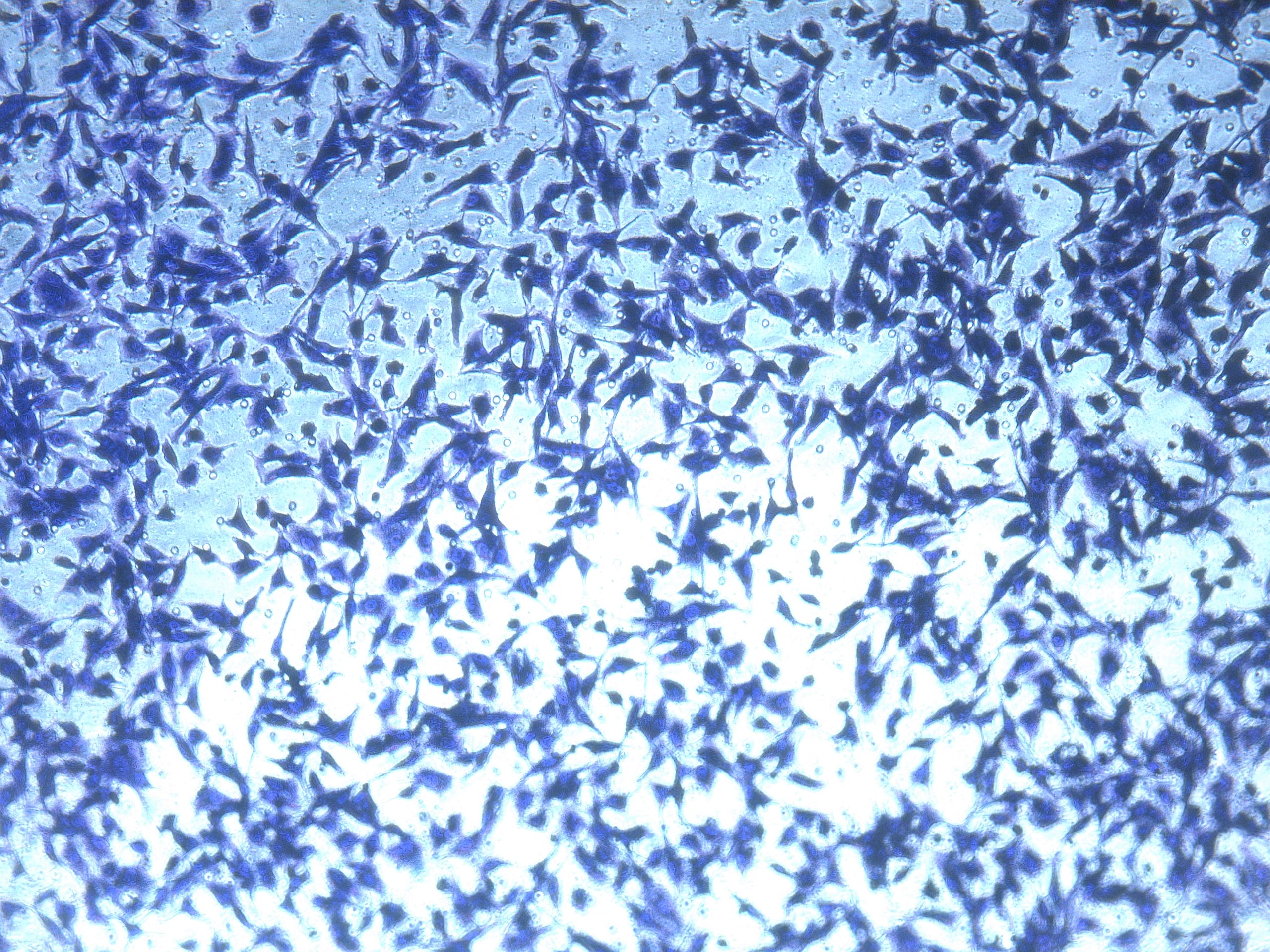

Supplement: Supplemental Information 14 [file peerj-12-18497-s014.zip › qbc939 functional experiment/nc knockdown /qbc migration nc si/picture/nc-2.jpg]

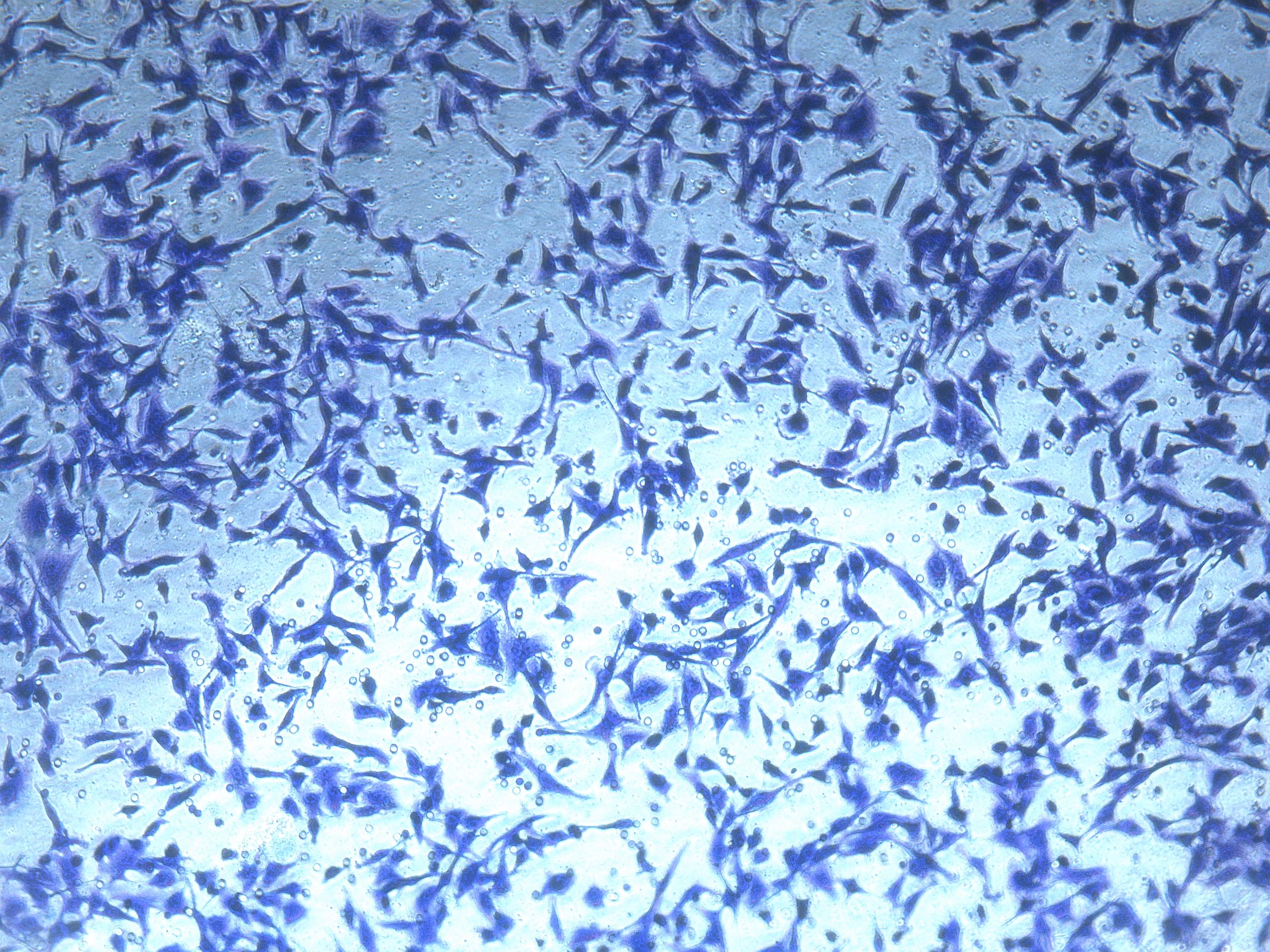

Supplement: Supplemental Information 14 [file peerj-12-18497-s014.zip › qbc939 functional experiment/nc knockdown /qbc migration nc si/picture/nc-3.jpg]

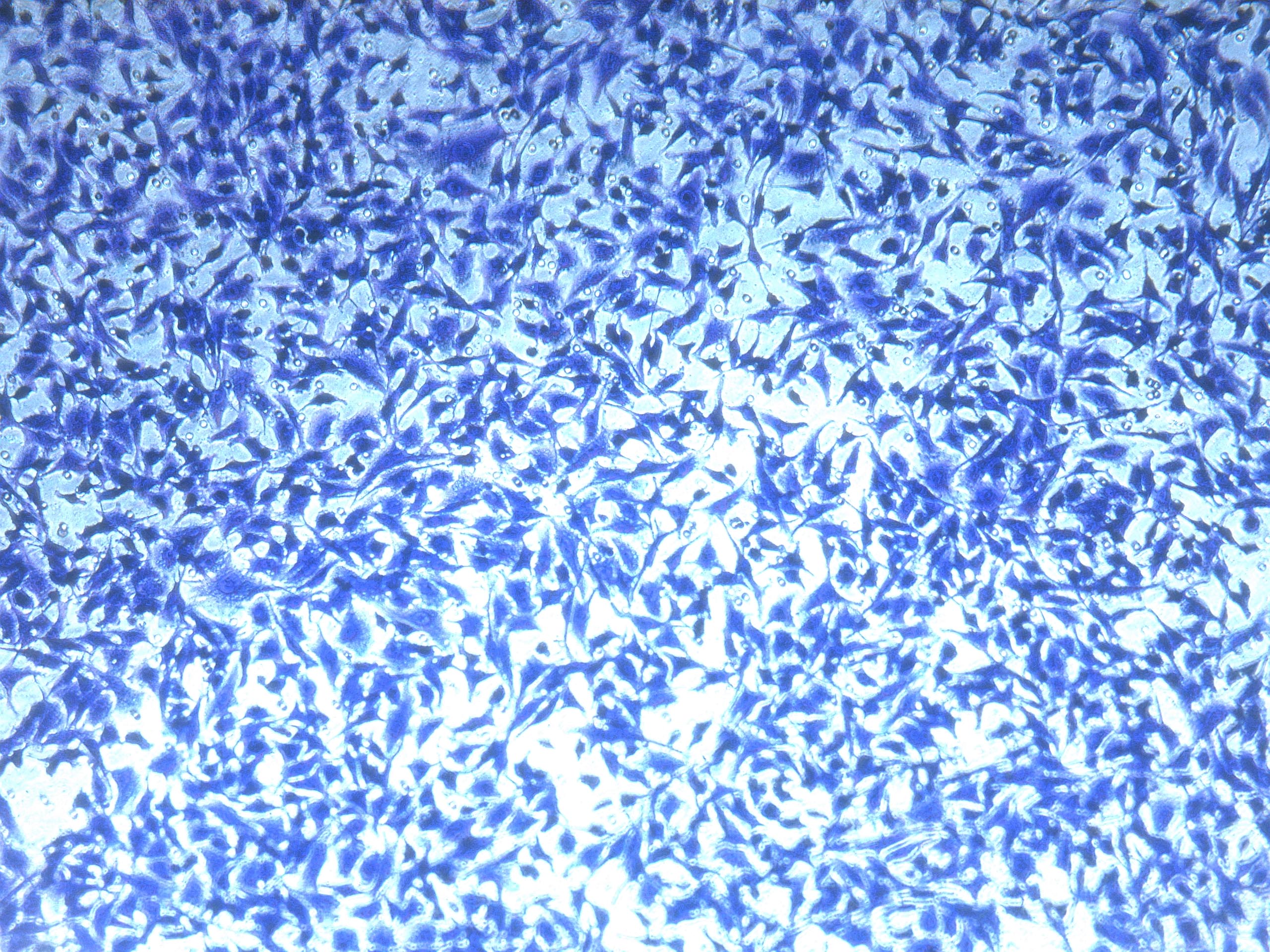

Supplement: Supplemental Information 14 [file peerj-12-18497-s014.zip › qbc939 functional experiment/nc knockdown /qbc migration nc si/picture/si-1.jpg]

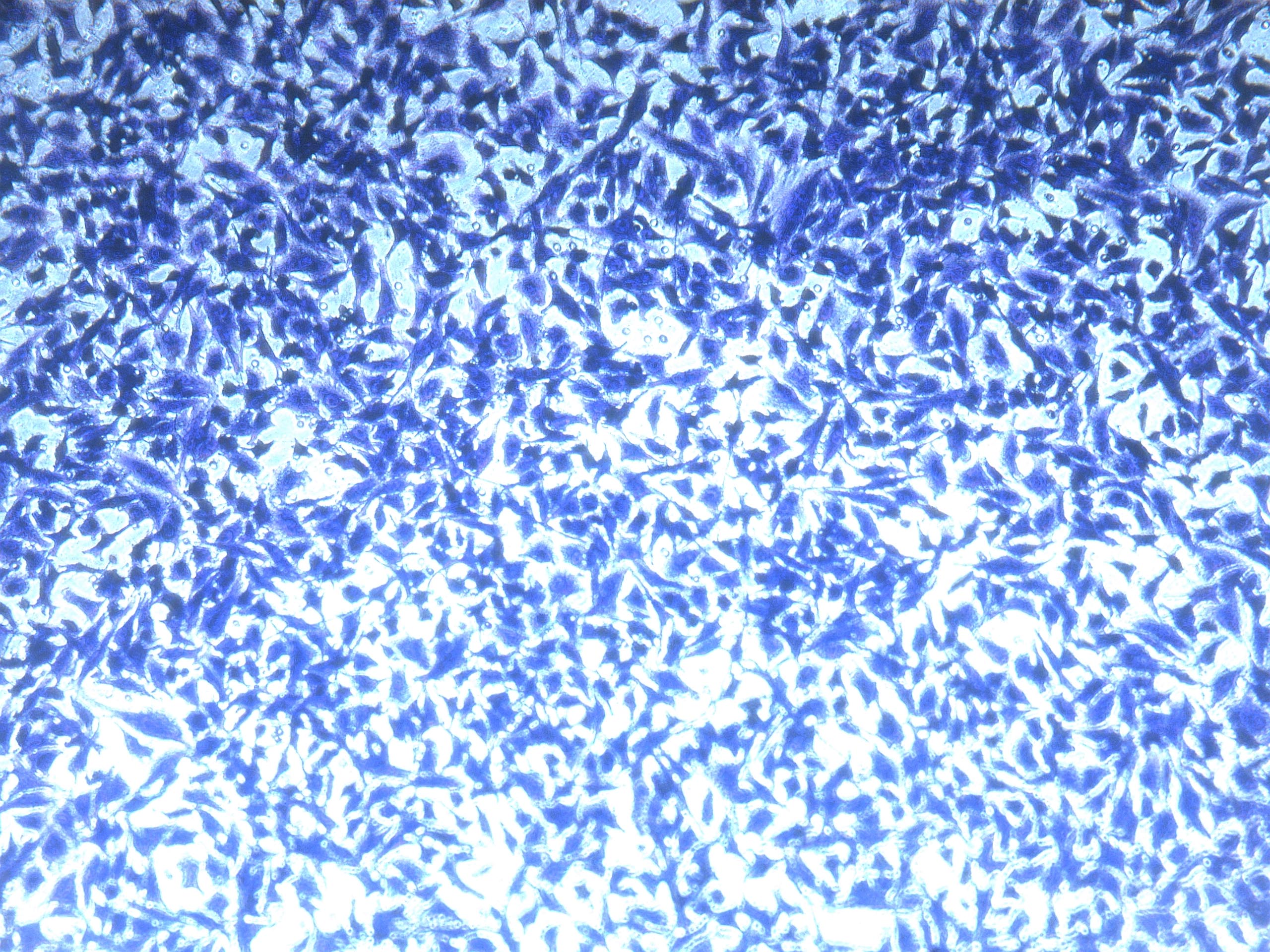

Supplement: Supplemental Information 14 [file peerj-12-18497-s014.zip › qbc939 functional experiment/nc knockdown /qbc migration nc si/picture/si-2.jpg]

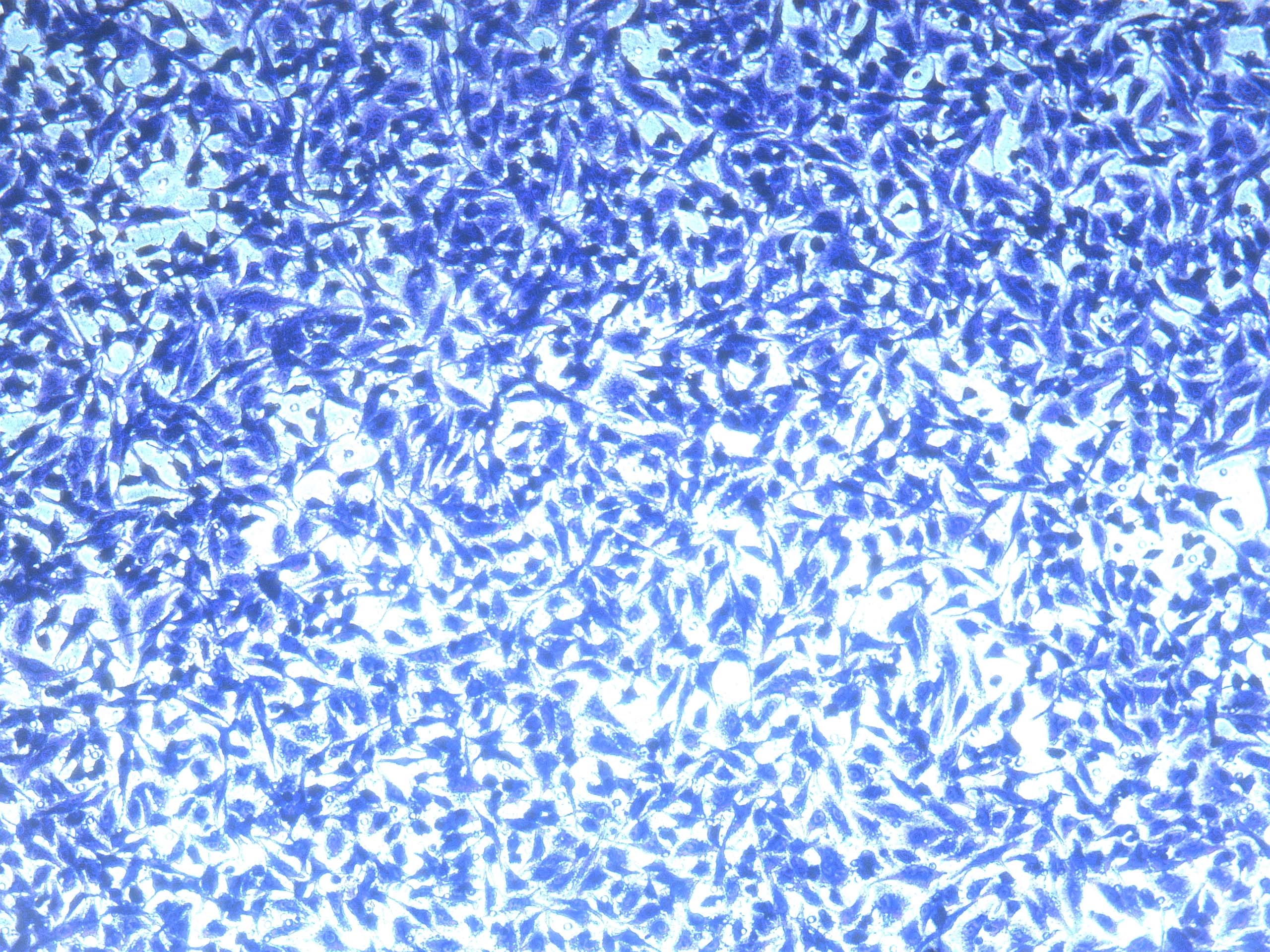

Supplement: Supplemental Information 14 [file peerj-12-18497-s014.zip › qbc939 functional experiment/nc knockdown /qbc migration nc si/picture/si-3.jpg]

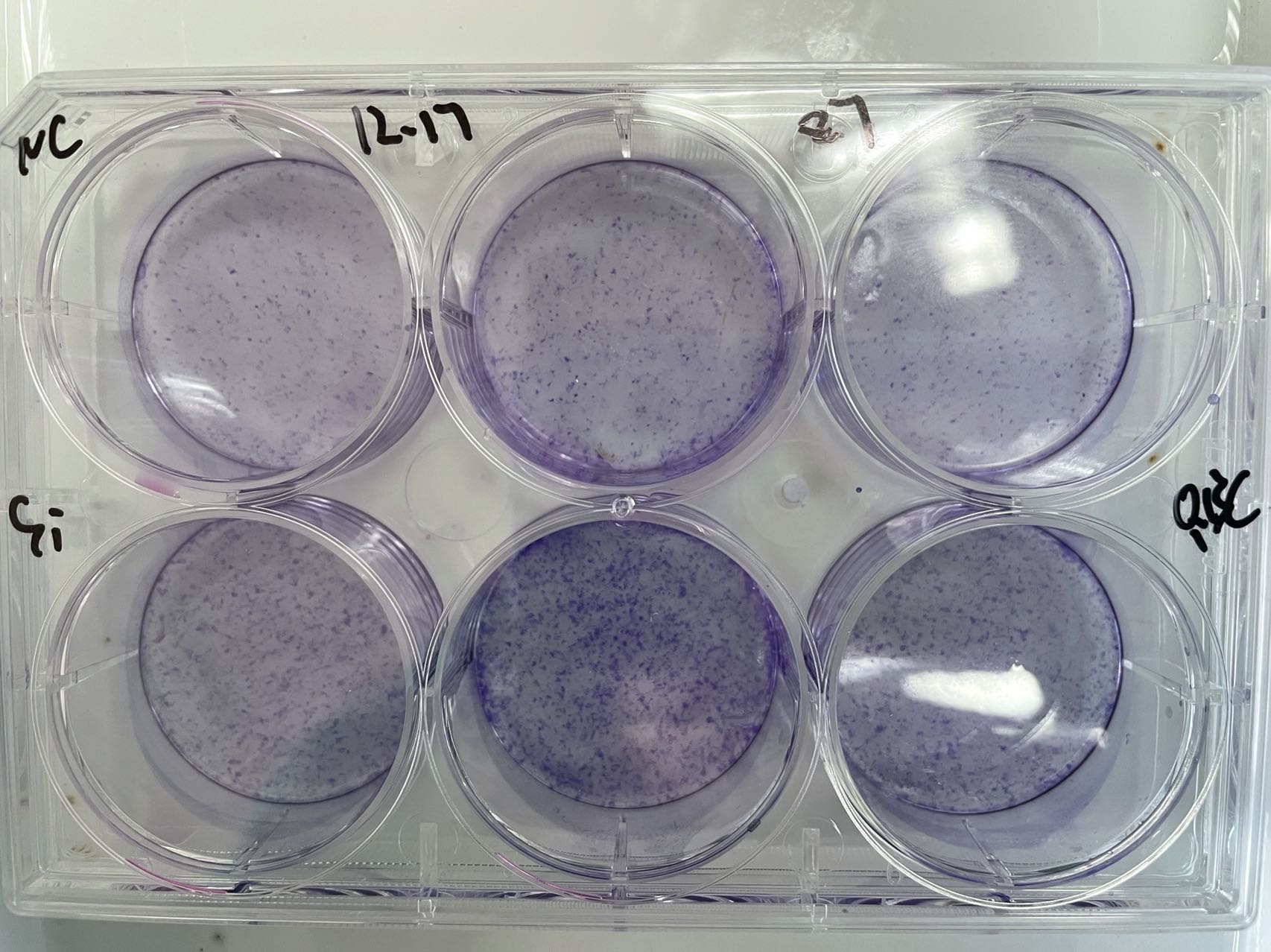

Supplement: Supplemental Information 14 [file peerj-12-18497-s014.zip › qbc939 functional experiment/nc knockdown /qbc939 clone formation nc si/QBC-克隆.jpg]
