# Supplementary material for: The CLEC3B inhibits cellular proliferation and metastasis of cholangiocarcinoma through Wnt/β-catenin pathway
Source: PeerJ. 2024 Nov 13;12:e18497. doi: 10.7717/peerj.18497 (PMC11568818; doi:10.7717/peerj.18497)
Supplement: Supplemental Information 16 [file peerj-12-18497-s016.docx]

**Highlights**

CLEC3B was different in the survival analysis of cholangiocarcinoma, and the mRNA expression level of cholangiocarcinoma patients was significantly lower.

For the first time, the combination of calcium ion and CLEC3B was used to explore the influence of calcium ion on the biological effect of CLEC3B.

Overexpression of CLEC3B could inhibit the proliferation, migration and invasion ability of bile duct cancer cells.

CLEC3B regulates the progression of bile duct cancer cells through the Wnt/ beta-catenin signaling pathway.

CLEC3B was found to be closely related to good prognosis of patients with cholangiocarcinoma.
